# Inducible Defenses Stay Up Late: Temporal Patterns of Immune Gene Expression in *Tenebrio molitor*

**DOI:** 10.1534/g3.113.008516

**Published:** 2014-06-01

**Authors:** Paul R Johnston, Olga Makarova, Jens Rolff

**Affiliations:** Evolutionary Biology, Institute for Biology, Free University of Berlin, 14195 Berlin, Germany

**Keywords:** RNAseq time course, costs of immunity, insect immunity, persistent infection, innate immunity, complex genetics, tolerance, complex immunity, infection, resistance

## Abstract

The course of microbial infection in insects is shaped by a two-stage process of immune defense. Constitutive defenses, such as engulfment and melanization, act immediately and are followed by inducible defenses, archetypically the production of antimicrobial peptides, which eliminate or suppress the remaining microbes. By applying RNAseq across a 7-day time course, we sought to characterize the long-lasting immune response to bacterial challenge in the mealworm beetle *Tenebrio molitor*, a model for the biochemistry of insect immunity and persistent bacterial infection. By annotating a hybrid *de novo* assembly of RNAseq data, we were able to identify putative orthologs for the majority of components of the conserved insect immune system. Compared with *Tribolium castaneum*, the most closely related species with a reference genome sequence and a manually curated immune system annotation, the *T. molitor* immune gene count was lower, with lineage-specific expansions of genes encoding serine proteases and their countervailing inhibitors accounting for the majority of the deficit. Quantitative mapping of RNAseq reads to the reference assembly showed that expression of genes with predicted functions in cellular immunity, wound healing, melanization, and the production of reactive oxygen species was transiently induced immediately after immune challenge. In contrast, expression of genes encoding antimicrobial peptides or components of the Toll signaling pathway and iron sequestration response remained elevated for at least 7 days. Numerous genes involved in metabolism and nutrient storage were repressed, indicating a possible cost of immune induction. Strikingly, the expression of almost all antibacterial peptides followed the same pattern of long-lasting induction, regardless of their spectra of activity, signaling possible interactive roles *in vivo*.

Because of the importance of insects as models for vertebrate immunity (Lemaitre *et al.* 1996) and as vectors of disease (Enayati and Hemingway 2010), insect immune defenses have been studied in great detail (Rolff and Reynolds 2009; Kounatidis and Ligoxygakis 2012) and the interplay between constitutive and, hence, fast-acting immune responses and inducible defenses has been elucidated. Similar to vertebrates, insect immunity comprises a suite of constitutive responses such as phagocytotic engulfment, melanization, and production of reactive oxygen, as well as inducible components such as antimicrobial peptides (Rolff and Reynolds 2009; Kounatidis and Ligoxygakis 2012).

Insect immune systems and, more generally, invertebrate immune systems, however, are devoid of B-cell–mediated and T-cell–mediated memory. Presumably, this perceived lack of a memory mechanism explains why most studies of insect immune gene expression capture only up to 48 hr after infection.

Yet, many parasites, such as *Plasmodium* (Michel and Kafatos 2005) or microsporidia (Schwarz and Evans 2013), are present in the host for several days. It has been frequently reported that bacterial infections can persist in insect hosts for several days to even weeks. Persistent infections can also be beneficial. Mutualistic relationships with microbes are often established for the lifetime of the host and interactions can be mediated by the insect immune system, for example, by antimicrobial peptides such as coleoptericins (Login *et al.* 2011).

Independent of persistent infection, elevated antimicrobial responses in insects can be long-lasting. Elevated antimicrobial activity has been reported for 9 d in the silk moth (Faye *et al.* 1975), for 11 d in *Rhodnius prolixus* (Azambuja *et al.* 1986), for 14 d in bumble bees (Korner and Schmid-Hempel 2004), for 21 d in our model *Tenebrio molitor* (Haine *et al.* 2008b), and for 44 d in dragonflies (Bulet *et al.* 1992). Hence, the duration of the elevated antimicrobial response can be a significant part of total life span in many insects.

On infection, insects utilize an array of recognition and effector systems adapted to bacterial, viral, and eukaryotic pathogens. Recognition of bacterial infection has been intensively studied in *Drosophila melanogaster* and also in *T. molitor* (Park *et al.* 2011), in which lysine-type peptidoglycan from Gram-positive bacteria and diaminopimelic-type peptidoglycan from Gram-negative bacteria activate signaling via the Toll and IMD pathways, respectively. After a breach of the cuticle, constitutive defenses including phenoloxidase, some lysozymes, and phagocytotic cells act quickly. Phagocytes are analogous to human macrophages and recognize microbes using receptors and opsonins such as scavenger receptors, thio-ester proteins (TEPs), or the highly variable, alternatively spliced Dscam (Cherry and Silverman 2006).

The insect equivalent to the liver, the fat body, not only is of great metabolic importance but also is pivotal in the production of inducible immune effectors, including antimicrobial peptides that follow constitutive responses over the course of an infection. The inducible antimicrobial defense responses are elicited by recognition of conserved microbe-associated molecular patterns by PGRPs and/or GNBPs, which induce the Toll and IMD signal transduction cascades, complemented by the Jak/Stat and JNK pathways, and activate the NF-kappaB transcription factors relish, dorsal, and dif, which induce expression of antimicrobial peptides (Kounatidis and Ligoxygakis 2012). These pathways are conserved in many insects including disease vectors such as mosquitoes (Kafatos *et al.* 2009) and the ancient odonates (Johnston and Rolff 2013).

Recent work suggested that the persistence of bacterial infections is shaped by a two-stage process of insect immune defenses (Schneider and Chambers 2008). Haine *et al.* (2008a) performed an infection experiment in *T. molitor* and reported that the majority of *Staphylococcus aureus* is cleared within 1 hr of injection, yet induced antimicrobial activity is only detected after 6 hr and peaks even later, at approximately day 4 (Haine *et al.* 2008a). Bacteria that survive the initial immune response are more resistant to host defenses on reinfection (Haine *et al.* 2008a). These observations led to the suggestion that fast-acting constitutive immune responses, for example, melanization, phagocytic engulfment, and generation of reactive oxygen species clear the majority of the infection and that the main function of the inducible immune response is to “mop up” remaining bacteria and to control persistent infections (Haine *et al.* 2008a; Schneider and Chambers 2008). This latter notion is based on the observation that elevated antimicrobial activity after challenge with living or dead bacteria can be observed for up to 21 d in *T. molitor* (Haine *et al.* 2008b). These observations are based on the functional zone of clearance assays that measure the overall antimicrobial activity of cell-free hemolymph. Hence, a molecular analysis is warranted that elucidates which components of the immune system are upregulated over the time course of an infection, especially during the start of the expression and peak activity at approximately days 3–5 after infection in *T. molitor* (Haine *et al.* 2008b).

*T. molitor* is an established model for the biochemistry of insect immunity (Park *et al.* 2011) despite the lack of a reference genome sequence. The biochemical activation of the Toll pathway has been elucidated in *T. molitor* (Roh *et al.* 2009), although the IMD pathway is not yet described (Chae *et al.* 2011). Several antimicrobial peptides have been characterized in detail, most recently tenecin 4, which bears similarity to *Drosophila* attacins (Chae *et al.* 2011). Recent work using *T. molitor* has also highlighted the role of PGRP-SA in the detection of peptidoglycan as well as D-alanylation–meditated evasion of PGRP recognition by *S. aureus* (Kurokawa *et al.* 2011).

Here, we present the first comprehensive RNAseq study of the temporal dynamics of an insect immune response, up to 7 d after immune challenge, using the model insect *T. molitor*. Based on the observations of long-lasting inducible immunity against *S. aureus* in *T. molitor* (Haine *et al.* 2008a; Haine *et al.* 2008b), we quantified gene expression 6 hr and 1, 3, 5, and 7 d after immune challenge to gain a comprehensive insight into the temporal dynamics of the insect immune system over the period of 1 wk. We show that genome-independent transcriptome analysis is effective, not only for annotation of the immune system but also for revealing temporal patterns of differential gene expression. The transcriptional dynamics of immune challenge are characterized by a striking separation of transient and long-lasting responses, with the latter dominated by induction of a suite of antimicrobial peptides.

We present the following: a reference transcriptome assembly derived from insects challenged with both Gram-positive and Gram-negative bacteria and utilizing data from multiple sequencing platforms; annotation of genes encoding components of the *T. molitor* immune system; and quantitative RNAseq analyses of the response to challenge with *S. aureus* that reveal transient as well as long-lasting induction and repression of gene expression.

## Materials and Methods

### Insect culturing

Final instar *T. molitor* larvae and Progrub formulated diet were purchased from a commercial supplier (Livefoods Direct, Sheffield, UK). Larvae were reared *en masse* under a 12:12-hr photoperiod at 25° with *ad libitum* access to food supplemented with apple. Pupae were collected daily and females were maintained individually in grid boxes. Newly eclosed beetles were provided with food and fresh apple was replaced daily. All experimental treatments were performed 7 d after adult eclosion.

### Bacterial preparations

*S. aureus* SH1000 and *Escherichia coli* K12 were grown overnight at 37° in Mueller-Hinton broth and Luria broth, respectively. Two bacterial preparations were produced for immune challenge experiments, the first with a 1:1 combination of *S. aureus* and *E. coli* and the second with *S. aureus* alone. Cultures were washed twice with sterile PBS, heat-killed at 95° for 30 min, and stored in 1-ml aliquots at −80° until further use.

### Immune challenge experiments

The 7-d-old beetles received 5-µl intrahemocoelic injections of heat-killed bacteria (approximately 10^6^ cells) (Haine *et al.* 2008a, 2008b) between the second and third abdominal sternites that were first swabbed with 96% ethanol. Control beetles received injections of sterile PBS. Beetles were maintained on a diet supplemented with 2-mm cubes of fresh apple.

We first performed an immune-challenge experiment with a combination of *S. aureus* and *E. coli* to obtain a comprehensive reference of immune genes expressed in response to both Gram-positive and Gram-negative bacteria using 454 GS FLX titanium sequencing. Five individuals were collected for RNA isolation at 6 hr after challenge and then every 24 hr for 7 d. For quantitative RNAseq analysis using Illumina HiSeq2000, a second challenge experiment was performed with *S. aureus* and 10 individuals were collected at 6 hr and 1, 3, 5, and 7 d after challenge. Additionally, five control individuals were collected at each time point. This experiment was performed twice on consecutive weeks.

### RNA isolation

Insects were decapitated with a sterile razor blade and the intestines and reproductive tract were removed with sterile forceps. From each individual, hemolymph and fat body were combined, suspended in cold Trizol (Sigma), and homogenized with a 5-mm steel bead (Qiagen) using a TissueLyser (Qiagen) twice at 20 Hz for 10 s. RNA was recovered from the individual homogenates by chloroform extraction and isopropanol precipitation according to the manufacturer’s instructions and re-dissolved in RNA storage solution (Ambion). Samples were subsequently incubated with 2 units of TurboDNase (Ambion) for 30 min at 37° and RNA was isolated using an RNeasy MinElute cleanup kit (Qiagen).

### 454 Sequencing

Full-length cDNA synthesis, GS FLX titanium library construction, and sequencing on the GS FLX titanium platform were performed by GATC Biotech (Konstanz, Germany). Briefly, polyadenylated RNA was isolated from a pool constructed using 2 µg total RNA from each individual in the combined *S. aureus* and *E. coli* immune challenge experiment. Full-length cDNA was constructed according to a SMART protocol. First-strand cDNA synthesis was primed with oligo(dT), followed by RNA hydrolysis and adaptor-primed second-strand synthesis. After hydroxyapatite normalization, cDNA was coligated, nebulized, and sequenced three times on a GS FLX instrument using titanium chemistry on a one-sixteenth plate, a one-fourth plate, and a full picotiter plate, respectively. Resulting sequence data are available from the NCBI sequence read archive (SRA) under BioSample accession SAMN02389790.

### Illumina sequencing

The construction of 12 barcoded, non-normalized TruSeq cDNA libraries and sequencing on the HiSeq2000 platform were performed by GATC Biotech. Briefly, polyadenylated RNA was isolated from total RNA pools representing each replicated time point as described above (using *S. aureus*–challenged beetles only). Pools of RNA representing 10 individuals from each replicated time point as well as two pools representing control individuals were created by combining equal quantities of total RNA. cDNA from each treatment was bar-coded with TruSeq universal adapters, pooled, and sequenced on a HiSeq2000 using two lanes of a single flow cell. Resulting sequence data are available from the NCBI SRA under BioSample accession numbers SAMN02389798–SAMN02389809.

### Hybrid transcriptome assembly and annotation

Raw 454 and Illumina reads were trimmed using cutadapt to remove sequencing barcodes and cDNA synthesis adaptors. Trimmed 454 reads were filtered by length to remove reads less than 50 bp. Illumina reads were combined into a single fastq file and normalized to a maximum of 20-fold coverage using k-mers of length 20 by khmer version 0.2 (Brown *et al.* 2012). Paired-end reads were simulated from 454 reads and normalized using simulate_illuminaPE_from_454ds.pl and normalize_by_kmer_coverage.pl, respectively, from Trinity assembler version r2013-02-25 (Grabherr *et al.* 2011; Haas *et al.* 2013). Both sets of digitally normalized reads were then combined and assembled using Trinity. Trinity assembly generates components that each comprises a group of contig sequences that are inferred to represent alternative splice forms or closely related paralogs (Grabherr *et al.* 2011). To eliminate possible artifacts, sequences representing less than 1% of the per-component expression across all mapped RNAseq reads were discarded. Annotation was performed following the trinotate annotation suite guidelines. Homology searches and predictions were performed locally and used to populate an sqlLITE database with the trinotate perl wrapper from Trinity assembler version r2013-02-25 at an e-value threshold of 1e−5. Briefly, peptide sequences were predicted from the assembly by Trinity transdecoder and used to query SwissProt with BLAST. Protein domains, signal peptides, and transmembrane domains were determined using HMMER (Finn *et al.* 2011), signalP (Petersen *et al.* 2011), and tmHMM (Krogh *et al.* 2001), respectively. Putative orthologs were predicted from reciprocal best BLAST hits with the *Tribolium castaneum* predicted proteome official gene set (http://beetlebase.org/) as described elsewhere (Johnston and Rolff 2013). The Insecta level of OrthoDB version 6 was downloaded and used to define both gene ontologies of the *T. castaneum* official gene set as well as ortholog relationships with other published insect genomes (Waterhouse *et al.* 2011). Antimicrobial peptide genes were identified by reciprocal best BLAST hits with *T. castaneum* antimicrobial peptides (AMPs) and annotation by BLAST and HMMER.

### RNASeq analysis

Trimmed Illumina reads from each replicate were mapped to the reference assembly using RSEM (Li and Dewey 2011) and Bowtie (Langmead *et al.* 2009). Choice of methodology for analysis of differential gene expression was informed by a recent comparison of 10 RNAseq analysis methods (all of which are implemented in R) that utilized real and simulated data and identified DESeq as the most conservative method with the lowest rates of type I error and false discovery and no method-specific signal (Soneson and Delorenzi 2013). Differential gene expression was determined using the R bioconductor package DESeq (Anders and Huber 2010) using the default sharing mode to estimate dispersions with false discovery rates at *p* < 0.05, following the procedures of Benjamini and Hochberg (Anders and Huber 2010). Transcripts with a minimum of four-fold change in expression at *p* < 0.05 were extracted and clustered using the R package DIRECT (Fu *et al.* 2013) according to median-centered log2 fragments per feature kilobase per million reads mapped. Briefly, a one-parameter Dirichlet process prior was used to induce a prior distribution and to estimate cluster number. Partitions were sampled using a Metropolis-Hastings Markov Chain Monte Carlo procedure. Resampling and relabeling were used to create an allocation probability matrix describing clusters of genes (Fu *et al.* 2013). Tests for over-representation of molecular function and biological process Gene Ontology (GO) terms associated with lists of genes from DIRECT clusters were performed using the R package GOstats (Falcon and Gentleman 2007) using a hypergeometric test with a *p*-value cut-off of 0.01 and a nonredundant list of GOs associated with the reference assembly annotation as the gene universe (Falcon and Gentleman 2007).

### Quantitative PCR

Total RNA was re-isolated from individual frozen Trizol homogenates as described. From each biological replicate, three independent pools were created using 100 ng total RNA from individual insects. cDNA was synthesized using a cDNA-Synthesis Kit H Plus (Peqlab). Relative gene expression was determined using a peqGOLD Hot Start-Mix kit (Peqlab) and a StepOne real-time thermocycler (Applied Biosystems) according to the manufacturer’s instructions. Relative expression was calculated using the comparative Ct method with the ribosomal protein gene RPL27a as the control (Chae *et al.* 2011).

### cDNA cloning of antimicrobial peptide sequences

To verify the accuracy of the reference assembly, DNA sequences corresponding to the mature peptide of the five previously described *T. molitor* AMP genes were cloned. Ten μl cDNA derived from each individual insect from the *S. aureus* challenge time course (synthesized as described) were pooled and used as the template for cloning. Primers were designed for regions with no sequence variation immediately upstream and downstream of the predicted mature peptide sequence to allow for amplification of potential sequence variants. Primer sequences can be found in Supporting Information, Table S17. PCR was performed in 25 μl reaction volume containing 12.5 μl Promega GoTaq 2× master mix, 10 μM primers, and 2 μl cDNA template. PCR conditions were as follows: 95° for 2 min, 30× (95° for 30 sec, 52° for 30 sec, 72° for 30 sec), 72° for 7 min, and 4° hold. Amplicons were purified using QIAquick PCR purification kit (Qiagen) and cloned into pGEM-T vector using pGEM-T vector system kit (Promega). White colonies were screened by PCR with SP6 and T7 primers. PCR product purification and Sanger sequencing of positive clones with SP6 and T7 primers were performed by Macrogen (Seoul, Korea). Sequences were assembled using DNA baser and trimmed to the mature peptide using CLC sequence viewer 6. In total, 34 (+4 truncated at the 5′ or 3′ end) clones were obtained for tenecin 1, 41 were obtained for tenecin 2, 31 (+12 truncated at the 5′ or 3′ end) were obtained for tenecin 3, 36 were obtained for tenecin 4, and 41 were obtained for attacin. Cloned DNA sequences of mature AMPs can be found in Table S17.

## Results and Discussion

### Reference assembly

To generate a comprehensive reference transcriptome, we performed a hybrid assembly of 454 GS FLX titanium sequencing reads from *T. molitor* adults challenged with a combination of Gram-positive and Gram-negative bacteria and Illumina HiSeq2000 sequencing reads from insects challenged with the Gram-positive bacterium *S. aureus* only. Multiple Trinity assemblies utilizing differently preprocessed combinations of 454 and Illumina data were compared to identify the most comprehensive reference transcriptome for subsequent annotation and RNAseq quantitation (Table S1 and Table S2). Assembly of digitally normalized Illumina reads together with 76-bp paired-end reads simulated from the 454 data resulted in the greatest number of components, transcripts, and putative orthologs of *T. castaneum* genes as well as universal single-copy orthologs (Table S1 and File S1). Filtering this assembly to retain transcripts representing at least 1% of the per-component expression produced a reference transcriptome of 44,516 components containing 90,956 sequences with N50 of 1644 bp and N90 of 393 bp (File S2). The transcriptome assembly is primarily derived from hemocytes and fat body tissue of adult females, yet only 20 of 112 existing *T. molitor* Sanger cDNA sequences failed to retrieve a near-identical reciprocal best blastn hit with the reference transcriptome assembly (Table S3). It is likely that these 20 sequences were not detected because they are expressed in a sex-specific, developmental stage-specific, and/or tissue-specific manner (Table S4). For example, seven of these sequences encode variants of a *T. molitor* antifreeze protein that is expressed in the midguts of overwintering larvae (Graham *et al.* 2000) (Table S4), a tissue and developmental stage not represented in our sampling. Reciprocal blast analysis of 77,118 predicted peptides (File S3) compared with the *T. castaneum* official gene set proteome (16,645 proteins) identified 9370 putative orthologs (Table S5). By assigning *T. molitor* sequences to ortholog groups based on *T. castaneum* reciprocal blast hits, 3120 of 3377 universal single-copy orthologs that are conserved across the arthropods were identified (Waterhouse *et al.* 2011). Despite the small deficit of universal single-copy orthologs and the observation that 11,758 genes in the *T. castaneum* official gene set are predicted to possess orthologs in at least one other published insect genome outside of the order coleoptera (Waterhouse *et al.* 2011), it is clear that the reference transcriptome assembly is comprehensive.

### Annotation of the *T. molitor* immune system

**Figure 1:**
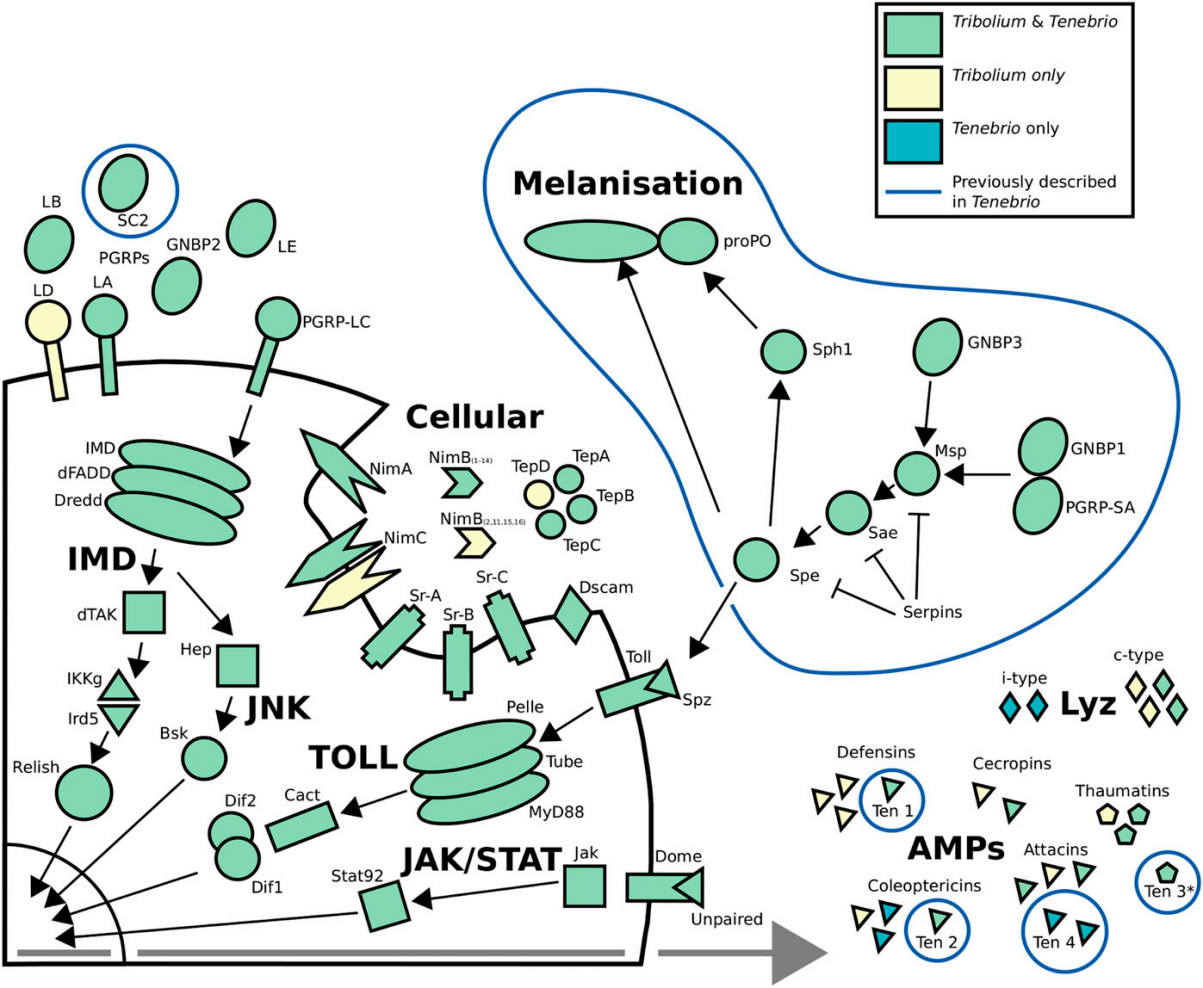
Comparison of immune genes and pathways annotated in *Tenebrio molitor* and *Tribolium castaneum*. Gene products are organized by pathway and cellular location according to Obbard *et al.* (2009). Green indicates genes that are annotated in both organisms. Yellow and blue indicate genes that have only been annotated in *T. castaneum* or *T. molitor*, respectively. Blue lines highlight genes that have been previously described in *T. molitor*.

Immune genes (Table S6) were defined as transcripts encoding putative orthologs of the manually curated *T. castaneum* immune system (Zou *et al.* 2007), previously described components of the *T. molitor* immune system (Figure 1), putative AMPs, and any predicted protein annotated with the biological process GO term “immune response” (GO:0006955).

For the 390 proteins previously defined as components of the *T. castaneum* immune system (Zou *et al.* 2007), 213 putative orthologs were identified (Table S6), including the conserved signaling pathways Toll, IMD, and JAK/STAT (Figure 1). Of the 177 remaining *T. castaneum* immune genes (Table S7) for which no *T. molitor* orthologs were found, the majority (124) were serine proteases (SPs), noncatalytic serine protease homologs (SPHs), or serine protease inhibitors (serpins). Together with SPHs, SPs regulate several aspects of the insect immune response, including proteolytic activation of the prophenoloxidase zymogen responsible for melanization as well as activation of the Toll pathway, which induces AMP synthesis (Figure 1) (Kounatidis and Ligoxygakis 2012). Lineage-specific expansions of SPs/SPHs are evident in many insect genomes, including *Anopheles* (Christophides *et al.* 2002), *Drosophila* (Ross *et al.* 2003), and *Tribolium* (Zou *et al.* 2007), in which repeated rounds of SP/SPH expansion (Zou *et al.* 2007) may account for the relative paucity of 1:1 orthology between *T. castanuem* and *T. molitor*. A similar pattern was observed in serpins, in which a recent major amplification within a 50-kb region of *T. castaneum* chromosome 8 produced a cluster of 16 closely related serpins (Zou *et al.* 2007) for which we found no *T. molitor* orthologs (Table S7). Another 40 putative immune system genes were identified by BLAST, HMMER, and/or immune response GO terms (Table S6). In this way, we identified members belonging to several functional classes that are under-represented in *T. castaneum*, such as chitotriosidases and the croquemort scavenger receptor family, as well as the i-type lysozymes, which are not annotated in *T. castaneum*. Differences in gene content between *Tribolium* and *Tenebrio* were also apparent within AMP gene families, which was expected given that AMPs are subject to rapid diversification with frequent duplication and turnover (Yang *et al.* 2011). Expansion and divergence are apparent in the *T. molitor* coleoptericin and attacin AMP families, with both possessing more members than in most other coleopterans, with the possible exception of the ladybird beetle *Harmonia axyridis* (Vilcinskas *et al.* 2013). In addition to the previously described tenecin 4 (Chae *et al.* 2011) and attacin C (Dobson *et al.* 2012), two attacins were identified that belong to divergent phylogenetic groups (Figure S1) along with their respective *T. castaneum* orthologs. In contrast, the coleoptericins formed species-specific groups (Figure S2) with two novel *T. molitor* coleoptercins clustering with the previously identified tenecin 2. A single novel cecropin was identified, the fourth member of this family to be reported from the coleoptera, supporting the notion that cecropins may be widespread in this order (Zou *et al.* 2007). As in *T. castaneum*, the cecropin possesses an atypical tyrosine-rich c-terminal extension (data not shown). Strikingly, we could reliably annotate only a single defensin, the previously identified tenecin 1 (Moon *et al.* 1994), which belongs to a coleoptera-specific clade of defensins (Zou *et al.* 2007). This is in contrast to *T. castaneum*, which possesses four defensins, one of which belongs to a clade of primitive defensins that is found in diverse arthropods (Zou *et al.* 2007). Two putative defensins were discarded because of low coverage (Table S8) but might represent transcripts of real genes with little or no expression in our target tissues. Defensin duplications are apparent in many insect species, including wasps (Gao and Zhu 2010), termites (Bulmer and Crozier 2004), ants (Zhang and Zhu 2012), and mosquitoes (Dassanayake *et al.* 2007), and we cannot exclude the possibility that transcripts from recently duplicated loci may have been collapsed during assembly, leading to an underestimation of copy number throughout the assembly.

### Gene expression

**Figure 2:**
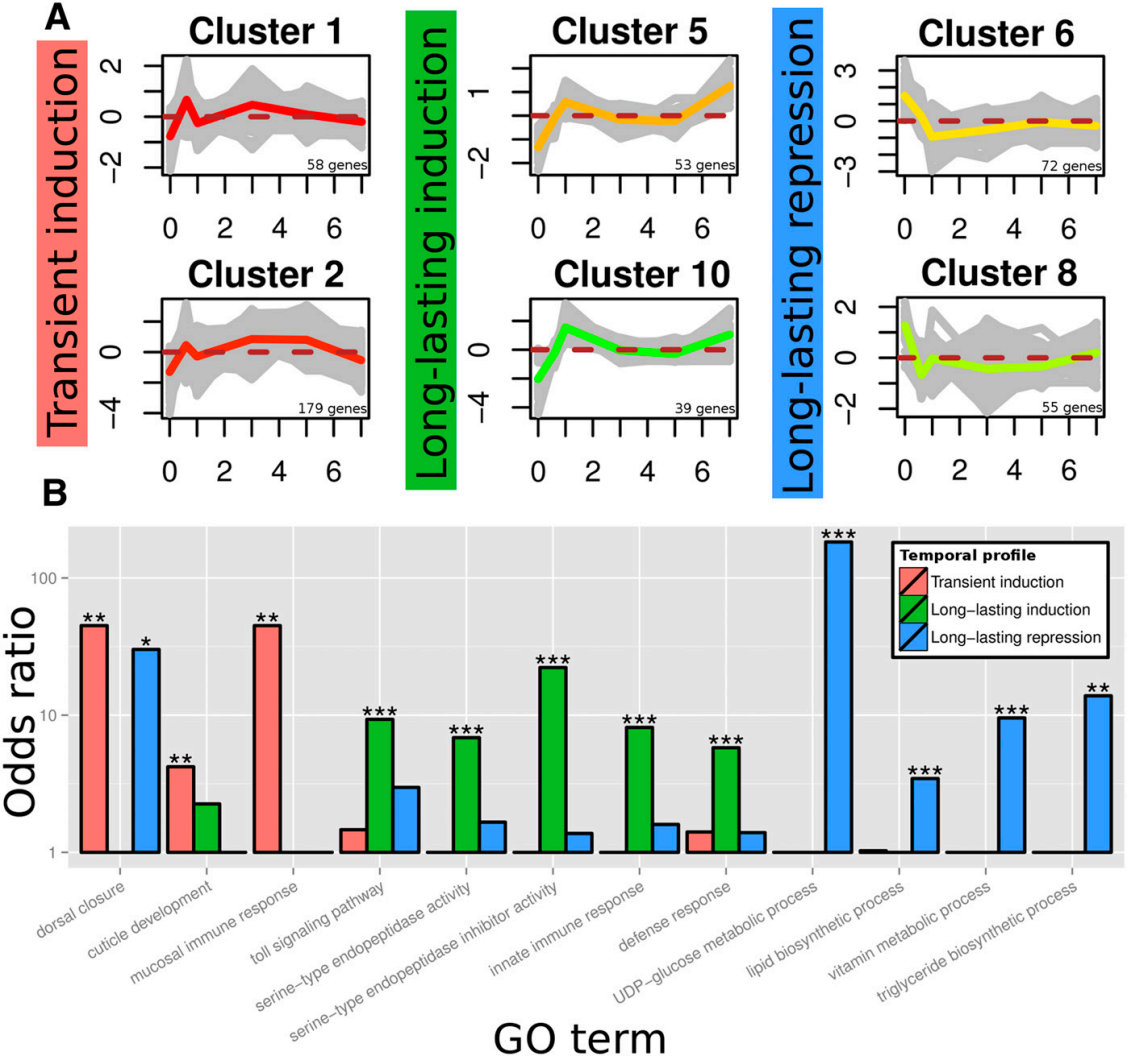
Contrasting profiles of differential gene expression after immune challenge. (A) Six clusters of differentially expressed genes showing three temporal profiles: transient induction; long-lasting induction; or long-lasting repression. Vertical axes represent median-centered log2 feature kilobase per million reads mapped (FPKM), whereas horizontal axes represent days after immune challenge. Colored lines depict the median profile for each cluster. (B) Significantly over-represented gene ontology in each temporal profile. *p < 0.05; **p < 0.01; ***p < 0.001.

The temporal response to immune challenge with heat-killed *S. aureus* was quantified by mapping approximately 8 million 100-bp Illumina reads per replicated time point to the reference assembly, followed by pairwise comparison of time points using DESeq (Anders and Huber 2010). DESeq identified 1050 components (of 44,516) as differentially expressed (DE) across the time course (Table S9) with a minimum of a four-fold change in expression at *p* < 0.05 after FDR correction. Bayesian clustering with a Dirichlet process prior was used to cluster DE genes by their temporal expression and to estimate the number of clusters within the data, following the procedures of Housden *et al.* (2013) and Fu *et al.* (2013). The process produced 27 clusters of DE genes reflecting temporal expression, magnitude of change, and variance (Figure S3, Table S10, and Table S18). Across the time course, differentially expressed immune effector genes belonged to six clusters displaying three general patterns: transient induction immediately after immune challenge; long-lasting induction; or long-lasting repression (Figure 2A).

### Transient response to immune challenge

The transient response immediately after immune challenge comprised induction of microbe recognition, signal transduction, and immune effector gene expression. This included genes encoding a previously described beta-1,3-glucan recognition protein (GNBP3) (Zhang *et al.* 2003) as well as the NF-kappaB transcription factor Relish, the ultimate target of the IMD signaling pathway, which is itself responsible for transcriptional activation of numerous immune genes (Kounatidis and Ligoxygakis 2012). A gene encoding phenoloxidase, the key enzyme in the melanization response, which produces cytotoxic melanin as well as oxidative intermediates with broad-spectrum antibacterial activity (Zhao *et al.* 2007), was also upregulated. Increased expression of genes encoding heme peroxidase, which participates in an alternative melanigenesis pathway (Nappi and Christensen 2005), and apolipophorins, which facilitate beta-1,3-glucan pattern recognition and phagocytosis (Whitten *et al.* 2004; Hanada *et al.* 2011), provide further evidence of a transient cellular response. Induction of FAD-glucose dehydrogenase, which enhances the encapsulation response by generating superoxide anions (Cox-Foster and Stehr 1994), and dual oxidase (duox) gene expression suggest a role for reactive oxygen–mediated killing in the early phase of the immune response. Recent work demonstrates a role for duox in activating the transcriptional response to wounding in *Drosophila* in addition to its role as an immune effector (Juarez *et al.* 2011). We also detected further transcriptional evidence for a transient wound healing response, including increased expression of genes encoding spectrin (scab) and integrin (karst), which are required for purse-string wound closure in *Drosophila* (Campos *et al.* 2010), as well as cohesin (fascin) and myospheroid, which mediate the wound-migratory response of plasmatocytes (Comber *et al.* 2013; Zanet *et al.* 2009). Genes encoding epidermal growth factor receptor and shark, which are components of the wound closure signaling pathway (Geiger *et al.* 2011), as well as a homolog of djub, which positively regulates epithelial proliferation via the hippo pathway in *Drosophila* (Das Thakur *et al.* 2010), were also upregulated. Supporting our inference of upregulation of genes involved in immunity and wound closure, the biological process GO terms “mucosal immune response” (GO:0002385; *p* = 0.003), “immune response in organ or tissue” (GO:0002251; *p* = 0.003), “immune system process” (GO:0002376; *p* = 0.0098), “dorsal closure” (GO:0007394; *p* = 0.0028), and “morphogenesis of an epithelial sheet” (GO:0002011; *p* = 0.0025) were over-represented in the transiently induced gene clusters (Figure 2B, Table S11, and Table S12).

### Long-lasting response to immune challenge

**Figure 3:**
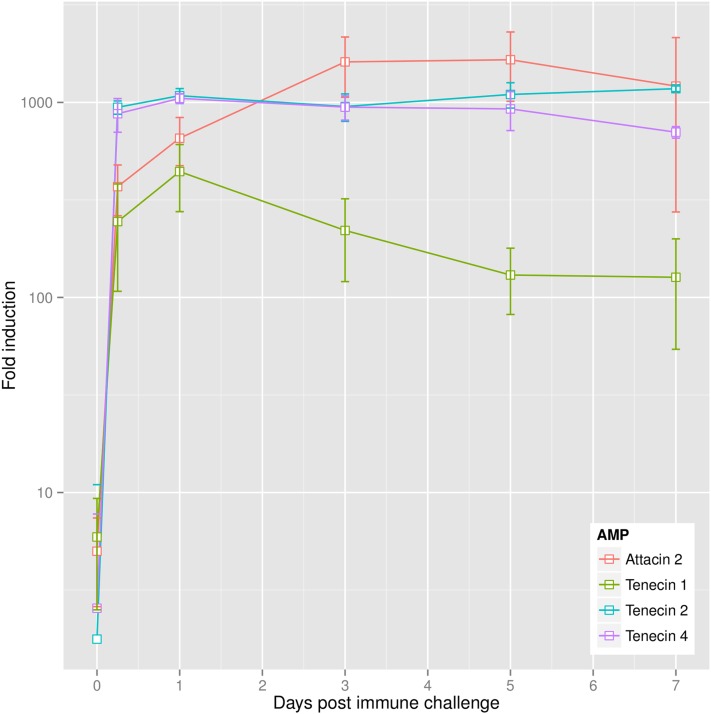
Quantification of antimicrobial peptide gene expression by relative qPCR. Error bars show SD across three biological replicates of pools of 8–10 individual insects.

Numerous immune-related biological process GO terms were over-represented in long-lasting response clusters, including “immune response” (GO:0006955; *p* = 6.151663e−04) and several child terms, as well as “Toll signaling pathway” (GO:0008063; *p* = 0.001) and the molecular function GO terms “serine-type endopeptidase inhibitor activity” (GO:0004867; *p* = 1.621524e−08) and “serine-type endopeptidase activity” (GO:0004252; *p* = 1.582472e−04) (Figure 2B, Table S13, and Table S14). Lasting induction of genes encoding components of the Toll signaling pathway was apparent, including the previously described Gram-negative binding protein 1 (GNBP1), which initiates the proteolytic activation cascade that converges on the Toll ligand Spaetzle (Kounatidis and Ligoxygakis 2012) (Figure 1), and an ortholog of *Tribolium* beta-1,3-glucan-binding protein 2. Six serine protease genes were upregulated, including the Spaetzle-processing enzyme (SPE) and SPE-activating enzyme (orthologs of *Drosophila* easter and snake, respectively), which are responsible for the final steps of Toll activation, as well as seven serine protease inhibitor genes. As in *Drosophila* (De Gregorio *et al.* 2001), expression of *Toll* itself was also induced after immune challenge. Strikingly, the only immune effector genes to show long-lasting induction were those encoding antibacterial peptides and the iron-sequestering protein ferritin. In contrast to the marked induction of antibacterial peptide gene expression, we detected no change in expression of genes encoding the thaumatins or tenecin 3, which are active against fungi and yeast (Kim *et al.* 1998; Altincicek *et al.* 2008), or the i-type and c-type lysozymes. A total of eight AMP genes were upregulated, including four attacins (two of which were previously described) (Dobson *et al.* 2012; Chae *et al.* 2011), three coleoptericins, including tenecin 2 (Roh *et al.* 2009), and the defensin tenecin 1 (Moon *et al.* 1994). To verify this result, fold induction of gene expression relative to procedural controls was determined by relative quantitative PCR for a subset of AMP genes across the time course (Figure 3).

The attacin tenecin 4 and the coleoptericin tenecin 2 show little to no antibacterial activity toward *S. aureus* and other Gram-positive bacteria *in vitro* (Roh *et al.* 2009; Chae *et al.* 2011). Furthermore, given that attacins kill Gram-negative bacteria via a nonlytic mechanism that involves lipopolysaccharide binding and inhibition of outer membrane protein synthesis (Carlsson *et al.* 1998), it is likely that attacin C and the two putative attacins identified here also possess negligible activity toward *S. aureus*. Long-lasting induction of almost all antibacterial peptides, regardless of their spectra of activity, is striking and may arise from a lack of specificity in the immune response. Alternatively, antibacterial peptides may interact when expressed in combination or may act in a sublethal, bacteriostatic manner.

### Metabolic repression

A single immune gene encoding a scavenger receptor class B member was repressed after immune challenge. Dramatic downregulation of numerous metabolic genes provides clear evidence of general metabolic repression. Of 81 over-represented biological process GO terms, 55 described genes with functions in metabolism or biosynthesis, including glucose metabolism, as well as lipid and vitamin biosynthesis (Figure 2B, Table S15, and Table S16). Expression of the hemolymph storage protein hexamerin, which forms a nutrient reservoir in many insects, was also repressed. Repression of dispensable metabolic pathways as a cost of intense immune gene expression was proposed after a genome-wide analysis of the immune response in *Drosophila* (De Gregorio *et al.* 2001). Recent work demonstrated that activation of the Toll pathway (but not IMD) in *Drosophila* suppresses insulin signaling within the fat body and thereby reduces nutrient storage (DiAngelo *et al.* 2009). Our results suggest that a trade-off between metabolism and immunity may be a general phenomenon.

## Conclusions

Our data show that a genome-independent comprehensive RNAseq annotation of most of the conserved insect immune system in *T. molitor* is feasible. In accordance with suggestions of a clear two-stage process of insect immunity, as proposed previously (Haine *et al.* 2008a; Schneider and Chambers 2008), we found distinct temporal profiles with clear groups of immune-responsive genes. Notably, antimicrobial peptide genes were clearly persistently upregulated, whereas genes involved in constitutive defense responses showed only transient upregulation, presumably partly to replenish zymogens such as Prophenoloxidase. This is consistent with the idea that antimicrobial peptides are upregulated for a long duration to “mop up” and control persistent infections (Haine *et al.* 2008a). Finally, we have shown the suppression of metabolic genes, consistent with the proposed physiological costs of immune defenses (Schmid-Hempel 2011), that have often been shown at the physiological level (Adamo *et al.* 2008; Freitak *et al.* 2003) but have not been studied much at the molecular level.

## Supplementary Material

Supporting Information
