# Supplementary material for: Inducible Defenses Stay Up Late: Temporal Patterns of Immune Gene Expression in Tenebrio molitor
Source: G3 (Bethesda). 2014 Jun 1;4(6):947–55. doi: 10.1534/g3.113.008516 (PMC4065263; doi:10.1534/g3.113.008516)
Supplement: Supporting Information [file supp_4_6_947__index.html]

Inducible Defenses Stay Up Late: Temporal Patterns of Immune Gene Expression in Tenebrio molitor — Supporting Information 

# Inducible Defenses Stay Up Late: Temporal Patterns of Immune Gene Expression in *Tenebrio molitor*

## Supporting Information for Johnston, Makarova, and Rolff, 2014

**Files in this Data Supplement:**

- Supporting Information - Figures S1-S3, Files S1-S3, and Tables S1-S18 (PDF, 1 MB)
- Table S14 - Results of a hypergeometric test for over-representation of molecular function gene ontology terms associated with the long-lasting induction temporal profile. (HTML, 83 KB)
- Table S15 - Results of a hypergeometric test for over-representation of biological process gene ontology terms associated with the long-lasting repression temporal profile. (HTML, 690 KB)
- Table S16 - Results of a hypergeometric test for over-representation of molecular function gene ontology terms associated with the long-lasting repression temporal profile. (HTML, 156 KB)
- Figure S1 - Maximum likelihood phylogenetic tree showing relationships among beetle attacins. (PDF, 345 KB)
- Figure S2 - Maximum likelihood phylogenetic tree showing relationships among beetle coleoptericins. (PDF, 404 KB)
- Figure S3 - Gene expression clusters produced by the R package DIRECT. (PDF, 544 KB)
- Table S1 - Comparison of trinity assemblies utilizing illumina and/or 454 reads. (PDF, 401 KB)
- Table S2 - Summary statistics for each of the data sets used in *de novo* assembly. (PDF, 393 KB)
- Table S11 - Results of a hypergeometric test for over-representation of biological process gene ontology terms associated with the transiently-induced temporal profile. (HTML, 1 MB)
- Table S12 - Results of a hypergeometric test for over-representation of molecular function gene ontology terms associated with the transiently-induced temporal profile. (HTML, 190 KB)
- Table S13 - Results of a hypergeometric test for over-representation of biological process gene ontology terms associated with the long-lasting induction temporal profile. (HTML, 389 KB)
- File S1 - Full fasta format output of trinity reference assembly (.zip, 27 MB)
- File S2 - Reference assembly after filtering to remove sequences representing less than 1% of the per-component expression across all mapped RNAseq reads (.zip, 22 MB)
- File S3 - Full annotation report for 77,118 predicted peptides derived from the reference assembly (.zip, 8 MB)
- Table S3 - Reciprocal best blastn hits between assembled contigs and 89 previously published *Tenebrio molitor* gene sequences from GenBank (.csv, 3 KB)
- Table S4 - GenBank accession numbers and metadata for 20 previously published *Tenebrio molitor* gene sequences which do not retreive a reciprocal best blastn hit from assembled contigs (.tsv, 1 KB)
- Table S5 - Reciprocal best blastp hits between predicted proteins derived from assembled contigs and the *Tribolium castaneum* predicted proteome official gene set (http://beetlebase.org/). (.csv, 167 KB)
- Table S6 - Details of annotated *Tenebrio molitor* contigs defined as immune genes (.csv, 10 KB)
- Table S7 - Details of *Tribolium castaneum* immune genes for which no putative *Tenebrio molitor* ortholog could be identified (.tsv, 5 KB)
- Table S8 - Details of two putative defensins which were discarded due to poor read support (.txt, 1 KB)
- Table S9 - Genes which were differentially expressed at one or more timepoints in the timecourse (.txt, 13 KB)
- Table S10 - Details of cluster allocation for each differentially expressed gene (.tsv, 35 KB)
- Table S17 - Primer sequences used to clone AMP sequences from cDNA (.csv, 1 KB)
- Table S18 - Combined details of cluster allocation for each differentially-expressed gene together with annotations (.tsv, 1 MB)
