# Supplementary material for: Inducible Defenses Stay Up Late: Temporal Patterns of Immune Gene Expression in Tenebrio molitor
Source: G3 (Bethesda). 2014 Jun 1;4(6):947–55. doi: 10.1534/g3.113.008516 (PMC4065263; doi:10.1534/g3.113.008516)
Supplement: Supporting Information [file supp_g3.113.008516_008516SI.pdf]

G3·Genes | Genomes | Genetics

Inducible defenses stay up late: temporal patterns of immune gene expression in *Tenebrio molitor*

Paul R Johnston, Olga Makarova, Jens Rolff

Evolutionary Biology, Institute for Biology, Free University Berlin, Berlin, Germany

Corresponding author: Paul R. Johnston. Evolutionary Biology, Institute for Biology, Free University Berlin, Koenigin-Luise-Strasse 1-3, 14195 Berlin, Germany. paul.johnston@fu-berlin.de Phone: +49(0)30 838 54692
Fax: +49(0)30 838 54869

DOI: 10.1534/g3.113.008516

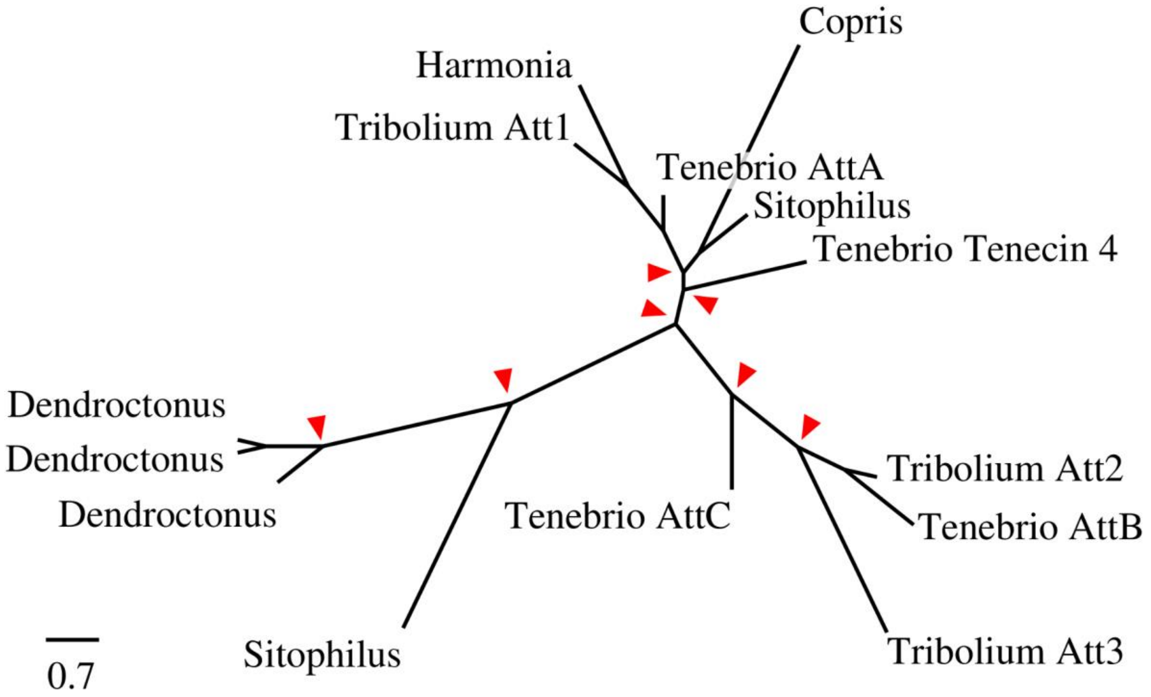

Figure S1 Maximum likelihood phylogenetic tree showing relationships among beetle attacins. Protein sequences were aligned using MUSCLE and Gblocks. Trees were constructed usin PhyML and TreeDyn using phylogeny.fr webserver. Red arrows indicate a confidence index > 0.8.

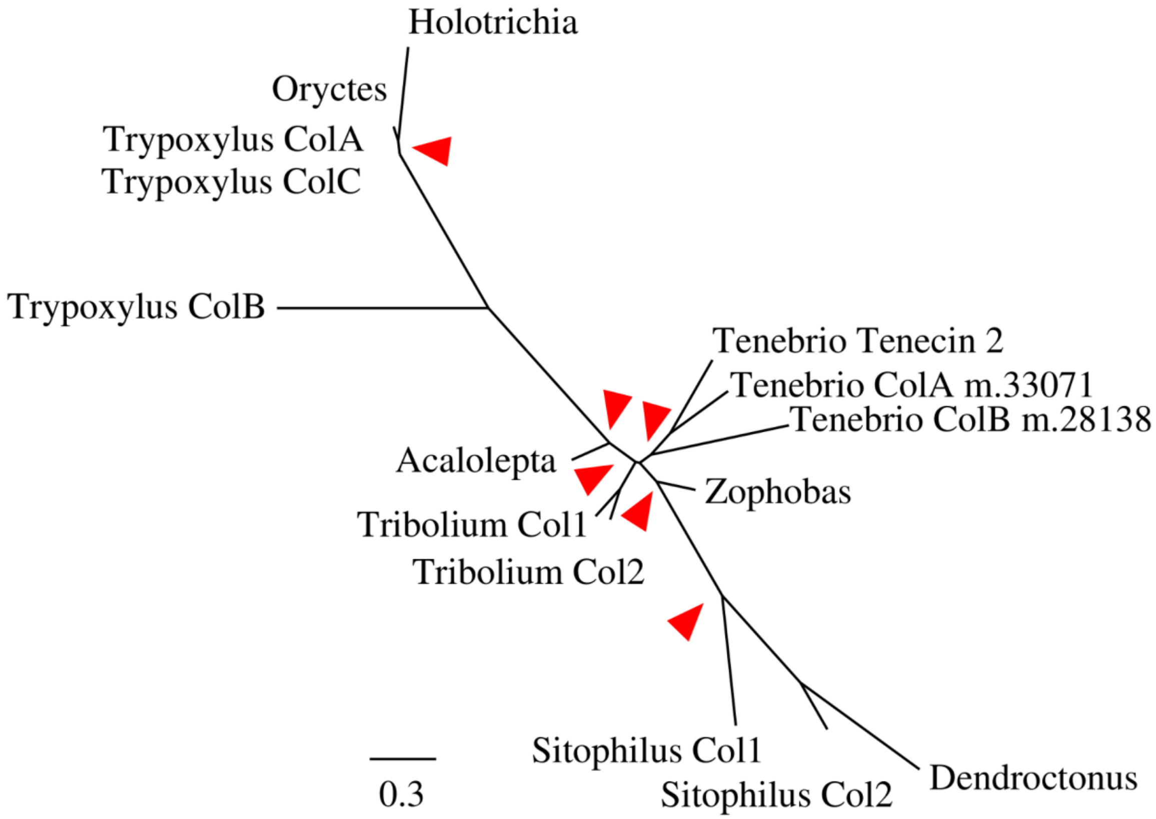

Figure S2 Maximum likelihood phylogenetic tree showing relationships among beetle coleoptericins. Protein sequences were aligned using MUSCLE and Gblocks. Trees were constructed usin PhyML and TreeDyn using phylogeny.fr webserver. Red arrows indicate a confidence index > 0.8.

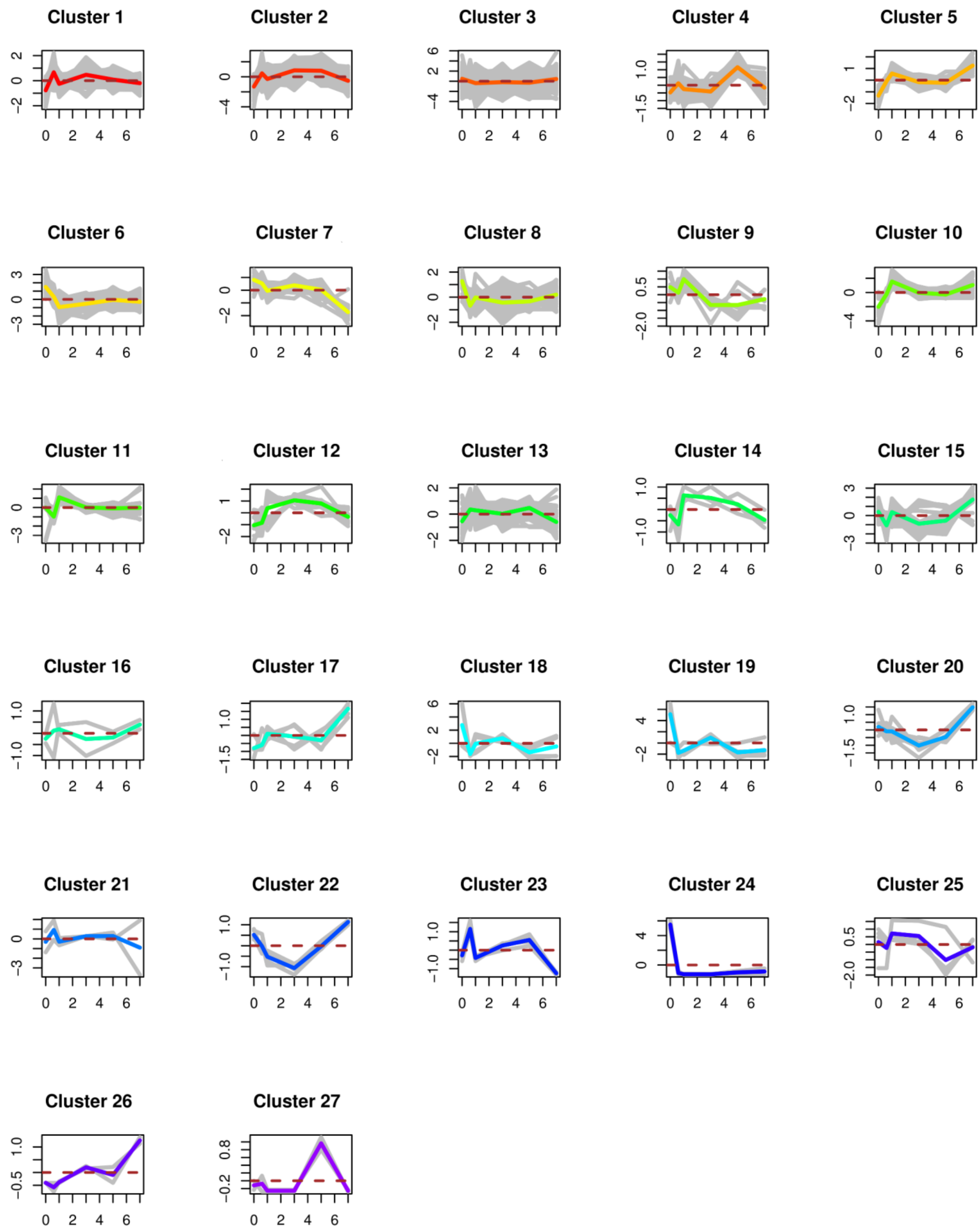

Figure S3 Gene expression clusters produced by the R package DIRECT. Vertical axes represent median centered log2 FPKM whereas horizontal axes represent days post immune challenge. Colored lines depict the median profile for each cluster.

Files S1-S3

Available for download at http://www.g3journal.org/lookup/suppl/doi:10.1534/g3.113.008516/-/DC1.

File S1 Full fasta format output of trinity reference assembly.

File S2 Reference assembly after filtering to remove sequences representing less than 1% of the per-component expression across all mapped RNAseq reads.

File S3 Full annotation report for 77,118 predicted peptides derived from the reference assembly.

Table S1 Comparison of trinity assemblies utilising illumina and/or 454 reads. sim PE, simulated paired-end; USCO, universal single-copy ortholog.

Assembly	Components	Transcripts	N50	N90	Orthologs	USCOs
Illumina	44811	96330	2545	571	8955	3071
454 (newbler)*	16647*	22507	1567	559	7628	2907
454 sim PE	25649	58060	696	306	6557	2465
Illumina + 454	45676	103597	2183	516	8830	2999
Illumina + 454 sim PE	49410	122521	1745	448	9370	3120
Illumina + 454 (newbler)*	54099*	118322	742	168	9057	3107
454	13724	25374	1097	434	6876	2646

*assembly newbler included for reference. Newbler does not compute components and the number of isogroups is reported instead. Orthologs, the number of putative orthologs between the assembly and the *Tribolium castaneum* proteome. In each assembly Illumina data was first digitally normalized.

Table S2 Summary statistics for each of the data sets used in *de novo* assembly. sim PE, simulated paired-end; diginorm, digitally normalized.

Data set	Reads	Mean length (bp)	File size (GB)
454	1,151,456	285	0.695[a]
Illumina*	95,339,034	101	22.8[a]
454 sim PE	7,367,318**	76	0.715[b]
Illumina diginorm	9,639,354	83	1.2[b]

*all assemblies utilized digitally normalized illulmina data. The properties of the original data are included here for reference.

**refers to number of read pairs

[a]fastq file

[b]fasta file

Tables S3-S18
Available for download at http://www.g3journal.org/lookup/suppl/doi:10.1534/g3.113.008516/-/DC1.

Table S3 Reciprocal best blastn hits between assembled contigs and 89 previously published *Tenebrio molitor* gene sequences from GenBank.

Table S4 GenBank accession numbers and metadata for 20 previously published *Tenebrio molitor* gene sequences which do not retreive a reciprocal best blastn hit from assembled contigs.

Table S5 Reciprocal best blastp hits between predicted proteins derived from assembled contigs and the *Tribolium castaneum* predicted proteome official gene set (http://beetlebase.org/).

Table S6 Details of annotated *Tenebrio molitor* contigs defined as immune genes.

Table S7 Details of *Tribolium castaneum* immune genes for which no putative *Tenebrio molitor* ortholog could be identified.

Table S8 Details of two putative defensins which were discarded due to poor read support.

Table S9 Genes which were differentially expressed at one or more timepoints in the timecourse.

Table S10 Details of cluster allocation for each differentially expressed gene.

Table S11 Results of a hypergeometric test for over-representation of biological process gene ontology terms associated with the transiently-induced temporal profile.

Table S12 Results of a hypergeometric test for over-representation of molecular function gene ontology terms associated with the transiently-induced temporal profile.

Table S13 Results of a hypergeometric test for over-representation of biological process gene ontology terms associated with the long-lasting induction temporal profile.

Table S14 Results of a hypergeometric test for over-representation of molecular function gene ontology terms associated with the long-lasting induction temporal profile.

Table S15 Results of a hypergeometric test for over-representation of biological process gene ontology terms associated with the long-lasting repression temporal profile.

Table S16 Results of a hypergeometric test for over-representation of molecular function gene ontology terms associated with the long-lasting repression temporal profile.

Table S17 Primer sequences used to clone AMP sequences from cDNA.

Table S18 Combined details of cluster allocation for each differentially-expressed gene together with annotations.
