# Supplementary material for: Inducible Defenses Stay Up Late: Temporal Patterns of Immune Gene Expression in Tenebrio molitor
Source: G3 (Bethesda). 2014 Jun 1;4(6):947–55. doi: 10.1534/g3.113.008516 (PMC4065263; doi:10.1534/g3.113.008516)
Supplement: Supporting Information [file supp_g3.113.008516_TableS14.html]

TableS14 

# Inducible Defenses Stay Up Late: Temporal Patterns of Immune Gene Expression in *Tenebrio molitor*

Gene to GO MF test for over-representation

| GOMFID | Pvalue | OddsRatio | ExpCount | Count | Size | Term |
| GO:0004867 | 0.000 | 22.228 | 0 | 8 | 64 | serine-type endopeptidase inhibitor activity |
| GO:0004866 | 0.000 | 16.784 | 1 | 8 | 82 | endopeptidase inhibitor activity |
| GO:0030414 | 0.000 | 16.558 | 1 | 8 | 83 | peptidase inhibitor activity |
| GO:0061135 | 0.000 | 15.915 | 1 | 8 | 86 | endopeptidase regulator activity |
| GO:0061134 | 0.000 | 14.253 | 1 | 8 | 95 | peptidase regulator activity |
| GO:0004857 | 0.000 | 10.938 | 1 | 8 | 121 | enzyme inhibitor activity |
| GO:0016714 | 0.000 | 280.897 | 0 | 2 | 3 | oxidoreductase activity, acting on paired donors, with incorporation or reduction of molecular oxygen, reduced pteridine as one donor, and incorporation of one atom of oxygen |
| GO:0004252 | 0.000 | 6.855 | 1 | 7 | 161 | serine-type endopeptidase activity |
| GO:0008236 | 0.000 | 6.124 | 1 | 7 | 179 | serine-type peptidase activity |
| GO:0017171 | 0.000 | 6.052 | 1 | 7 | 181 | serine hydrolase activity |
| GO:0051635 | 0.001 | 93.609 | 0 | 2 | 5 | bacterial cell surface binding |
| GO:0070279 | 0.001 | 11.120 | 0 | 4 | 56 | vitamin B6 binding |
| GO:0030170 | 0.001 | 11.120 | 0 | 4 | 56 | pyridoxal phosphate binding |
| GO:0019842 | 0.001 | 7.170 | 1 | 5 | 107 | vitamin binding |
| GO:0016831 | 0.001 | 16.439 | 0 | 3 | 29 | carboxy-lyase activity |
| GO:0043498 | 0.003 | 31.180 | 0 | 2 | 11 | cell surface binding |
| GO:0016830 | 0.003 | 11.536 | 0 | 3 | 40 | carbon-carbon lyase activity |
| GO:0004175 | 0.003 | 3.288 | 3 | 9 | 424 | endopeptidase activity |
| GO:0005537 | 0.004 | 25.505 | 0 | 2 | 13 | mannose binding |
| GO:0016829 | 0.004 | 5.088 | 1 | 5 | 148 | lyase activity |
| GO:0001163 | 0.007 | Inf | 0 | 1 | 1 | RNA polymerase I regulatory region sequence-specific DNA binding |
| GO:0001164 | 0.007 | Inf | 0 | 1 | 1 | RNA polymerase I CORE element sequence-specific DNA binding |
| GO:0001167 | 0.007 | Inf | 0 | 1 | 1 | sequence-specific DNA binding RNA polymerase I transcription factor activity |
| GO:0001169 | 0.007 | Inf | 0 | 1 | 1 | RNA polymerase I CORE element sequence-specific DNA binding transcription factor activity |
| GO:0001186 | 0.007 | Inf | 0 | 1 | 1 | RNA polymerase I transcription factor recruiting transcription factor activity |
| GO:0001187 | 0.007 | Inf | 0 | 1 | 1 | RNA polymerase I CORE element sequence-specific DNA binding transcription factor recruiting transcription factor activity |
| GO:0001134 | 0.007 | Inf | 0 | 1 | 1 | transcription factor recruiting transcription factor activity |
| GO:0047536 | 0.007 | Inf | 0 | 1 | 1 | 2-aminoadipate transaminase activity |
| GO:0070456 | 0.007 | Inf | 0 | 1 | 1 | galactose-1-phosphate phosphatase activity |
| GO:0031177 | 0.007 | Inf | 0 | 1 | 1 | phosphopantetheine binding |
| GO:0010347 | 0.007 | Inf | 0 | 1 | 1 | L-galactose-1-phosphate phosphatase activity |
| GO:0004492 | 0.007 | Inf | 0 | 1 | 1 | methylmalonyl-CoA decarboxylase activity |
| GO:0001082 | 0.007 | Inf | 0 | 1 | 1 | RNA polymerase I transcription factor binding transcription factor activity |
| GO:0001013 | 0.007 | Inf | 0 | 1 | 1 | RNA polymerase I regulatory region DNA binding |
| GO:0004510 | 0.007 | Inf | 0 | 1 | 1 | tryptophan 5-monooxygenase activity |
| GO:0004511 | 0.007 | Inf | 0 | 1 | 1 | tyrosine 3-monooxygenase activity |
| GO:0004505 | 0.007 | Inf | 0 | 1 | 1 | phenylalanine 4-monooxygenase activity |
| GO:0008793 | 0.007 | Inf | 0 | 1 | 1 | aromatic-amino-acid:2-oxoglutarate aminotransferase activity |
| GO:0030234 | 0.008 | 2.882 | 4 | 9 | 479 | enzyme regulator activity |
| GO:0048029 | 0.009 | 15.573 | 0 | 2 | 20 | monosaccharide binding |
| GO:0070011 | 0.010 | 2.595 | 4 | 10 | 593 | peptidase activity, acting on L-amino acid peptides |
| GO:0008233 | 0.012 | 2.511 | 4 | 10 | 611 | peptidase activity |
| GO:0005313 | 0.015 | 138.068 | 0 | 1 | 2 | L-glutamate transmembrane transporter activity |
| GO:0005138 | 0.015 | 138.068 | 0 | 1 | 2 | interleukin-6 receptor binding |
| GO:0015172 | 0.015 | 138.068 | 0 | 1 | 2 | acidic amino acid transmembrane transporter activity |
| GO:0004782 | 0.015 | 138.068 | 0 | 1 | 2 | sulfinoalanine decarboxylase activity |
| GO:0004530 | 0.015 | 138.068 | 0 | 1 | 2 | deoxyribonuclease I activity |
| GO:0004843 | 0.020 | 9.999 | 0 | 2 | 30 | ubiquitin-specific protease activity |
| GO:0070883 | 0.022 | 69.025 | 0 | 1 | 3 | pre-miRNA binding |
| GO:0016443 | 0.022 | 69.025 | 0 | 1 | 3 | bidentate ribonuclease III activity |
| GO:0004691 | 0.022 | 69.025 | 0 | 1 | 3 | cAMP-dependent protein kinase activity |
| GO:0019783 | 0.023 | 9.330 | 0 | 2 | 32 | small conjugating protein-specific protease activity |
| GO:0004058 | 0.029 | 46.011 | 0 | 1 | 4 | aromatic-L-amino-acid decarboxylase activity |
| GO:0052832 | 0.029 | 46.011 | 0 | 1 | 4 | inositol monophosphate 3-phosphatase activity |
| GO:0052833 | 0.029 | 46.011 | 0 | 1 | 4 | inositol monophosphate 4-phosphatase activity |
| GO:0052834 | 0.029 | 46.011 | 0 | 1 | 4 | inositol monophosphate phosphatase activity |
| GO:0008934 | 0.029 | 46.011 | 0 | 1 | 4 | inositol monophosphate 1-phosphatase activity |
| GO:0004499 | 0.029 | 46.011 | 0 | 1 | 4 | N,N-dimethylaniline monooxygenase activity |
| GO:0033784 | 0.029 | 46.011 | 0 | 1 | 4 | senecionine N-oxygenase activity |
| GO:0016597 | 0.034 | 7.358 | 0 | 2 | 40 | amino acid binding |
| GO:0080130 | 0.036 | 34.504 | 0 | 1 | 5 | L-phenylalanine:2-oxoglutarate aminotransferase activity |
| GO:0004553 | 0.039 | 3.332 | 1 | 4 | 175 | hydrolase activity, hydrolyzing O-glycosyl compounds |
| GO:0042393 | 0.043 | 6.499 | 0 | 2 | 45 | histone binding |
| GO:0030060 | 0.043 | 27.600 | 0 | 1 | 6 | L-malate dehydrogenase activity |
| GO:0050308 | 0.043 | 27.600 | 0 | 1 | 6 | sugar-phosphatase activity |
| GO:0019203 | 0.043 | 27.600 | 0 | 1 | 6 | carbohydrate phosphatase activity |
| GO:0016888 | 0.043 | 27.600 | 0 | 1 | 6 | endodeoxyribonuclease activity, producing 5'-phosphomonoesters |
| GO:0016853 | 0.044 | 4.031 | 1 | 3 | 108 | isomerase activity |
| GO:0004221 | 0.046 | 6.208 | 0 | 2 | 47 | ubiquitin thiolesterase activity |
| GO:0048037 | 0.050 | 2.652 | 2 | 5 | 275 | cofactor binding |
| GO:0015927 | 0.050 | 22.997 | 0 | 1 | 7 | trehalase activity |
| GO:0070546 | 0.050 | 22.997 | 0 | 1 | 7 | L-phenylalanine aminotransferase activity |
| GO:0005527 | 0.050 | 22.997 | 0 | 1 | 7 | macrolide binding |
| GO:0005528 | 0.050 | 22.997 | 0 | 1 | 7 | FK506 binding |
| GO:0004555 | 0.050 | 22.997 | 0 | 1 | 7 | alpha,alpha-trehalase activity |
| GO:0004525 | 0.050 | 22.997 | 0 | 1 | 7 | ribonuclease III activity |
| GO:0072341 | 0.050 | 22.997 | 0 | 1 | 7 | modified amino acid binding |
| GO:0016798 | 0.053 | 2.991 | 1 | 4 | 194 | hydrolase activity, acting on glycosyl bonds |
| GO:0036137 | 0.057 | 19.709 | 0 | 1 | 8 | kynurenine aminotransferase activity |
| GO:0016212 | 0.057 | 19.709 | 0 | 1 | 8 | kynurenine-oxoglutarate transaminase activity |
| GO:0003918 | 0.064 | 17.244 | 0 | 1 | 9 | DNA topoisomerase (ATP-hydrolyzing) activity |
| GO:0003916 | 0.078 | 13.792 | 0 | 1 | 11 | DNA topoisomerase activity |
| GO:0019955 | 0.078 | 13.792 | 0 | 1 | 11 | cytokine binding |
| GO:0070851 | 0.078 | 13.792 | 0 | 1 | 11 | growth factor receptor binding |
| GO:0008199 | 0.078 | 13.792 | 0 | 1 | 11 | ferric iron binding |
| GO:0005315 | 0.084 | 12.536 | 0 | 1 | 12 | inorganic phosphate transmembrane transporter activity |
| GO:0052745 | 0.084 | 12.536 | 0 | 1 | 12 | inositol phosphate phosphatase activity |
| GO:0016790 | 0.086 | 4.288 | 0 | 2 | 67 | thiolester hydrolase activity |
| GO:0031406 | 0.088 | 4.222 | 0 | 2 | 68 | carboxylic acid binding |
| GO:0035064 | 0.091 | 11.490 | 0 | 1 | 13 | methylated histone residue binding |
| GO:0004383 | 0.091 | 11.490 | 0 | 1 | 13 | guanylate cyclase activity |
| GO:0015114 | 0.091 | 11.490 | 0 | 1 | 13 | phosphate ion transmembrane transporter activity |
| GO:0004690 | 0.091 | 11.490 | 0 | 1 | 13 | cyclic nucleotide-dependent protein kinase activity |
| GO:0016709 | 0.098 | 10.605 | 0 | 1 | 14 | oxidoreductase activity, acting on paired donors, with incorporation or reduction of molecular oxygen, NADH or NADPH as one donor, and incorporation of one atom of oxygen |
| GO:0004520 | 0.098 | 10.605 | 0 | 1 | 14 | endodeoxyribonuclease activity |
| GO:0005126 | 0.104 | 9.846 | 0 | 1 | 15 | cytokine receptor binding |
| GO:0015179 | 0.111 | 9.189 | 0 | 1 | 16 | L-amino acid transmembrane transporter activity |
| GO:0016615 | 0.117 | 8.613 | 0 | 1 | 17 | malate dehydrogenase activity |
| GO:0001046 | 0.117 | 8.613 | 0 | 1 | 17 | core promoter sequence-specific DNA binding |
| GO:0004869 | 0.124 | 8.106 | 0 | 1 | 18 | cysteine-type endopeptidase inhibitor activity |
| GO:0004536 | 0.130 | 7.654 | 0 | 1 | 19 | deoxyribonuclease activity |
| GO:0004559 | 0.137 | 7.251 | 0 | 1 | 20 | alpha-mannosidase activity |
| GO:0005044 | 0.143 | 6.887 | 0 | 1 | 21 | scavenger receptor activity |
| GO:0001047 | 0.149 | 6.559 | 0 | 1 | 22 | core promoter binding |
| GO:0004842 | 0.151 | 2.316 | 1 | 3 | 184 | ubiquitin-protein ligase activity |
| GO:0003725 | 0.155 | 6.260 | 0 | 1 | 23 | double-stranded RNA binding |
| GO:0019787 | 0.163 | 2.228 | 1 | 3 | 191 | small conjugating protein ligase activity |
| GO:0008234 | 0.166 | 2.832 | 1 | 2 | 100 | cysteine-type peptidase activity |
| GO:0015923 | 0.168 | 5.737 | 0 | 1 | 25 | mannosidase activity |
| GO:0005506 | 0.169 | 1.928 | 2 | 4 | 295 | iron ion binding |
| GO:0030246 | 0.185 | 2.091 | 1 | 3 | 203 | carbohydrate binding |
| GO:0009975 | 0.186 | 5.097 | 0 | 1 | 28 | cyclase activity |
| GO:0015171 | 0.186 | 5.097 | 0 | 1 | 28 | amino acid transmembrane transporter activity |
| GO:0016769 | 0.186 | 5.097 | 0 | 1 | 28 | transferase activity, transferring nitrogenous groups |
| GO:0008483 | 0.186 | 5.097 | 0 | 1 | 28 | transaminase activity |
| GO:0016849 | 0.186 | 5.097 | 0 | 1 | 28 | phosphorus-oxygen lyase activity |
| GO:0015293 | 0.192 | 2.567 | 1 | 2 | 110 | symporter activity |
| GO:0003755 | 0.198 | 4.745 | 0 | 1 | 30 | peptidyl-prolyl cis-trans isomerase activity |
| GO:0016881 | 0.202 | 1.999 | 2 | 3 | 212 | acid-amino acid ligase activity |
| GO:0016787 | 0.202 | 1.306 | 17 | 20 | 2276 | hydrolase activity |
| GO:0043130 | 0.204 | 4.586 | 0 | 1 | 31 | ubiquitin binding |
| GO:0016859 | 0.204 | 4.586 | 0 | 1 | 31 | cis-trans isomerase activity |
| GO:0003727 | 0.215 | 4.298 | 0 | 1 | 33 | single-stranded RNA binding |
| GO:0008144 | 0.227 | 4.044 | 0 | 1 | 35 | drug binding |
| GO:0005275 | 0.233 | 3.928 | 0 | 1 | 36 | amine transmembrane transporter activity |
| GO:0038024 | 0.238 | 3.819 | 0 | 1 | 37 | cargo receptor activity |
| GO:0050661 | 0.238 | 3.819 | 0 | 1 | 37 | NADP binding |
| GO:0004497 | 0.240 | 1.820 | 2 | 3 | 232 | monooxygenase activity |
| GO:0015151 | 0.244 | 3.715 | 0 | 1 | 38 | alpha-glucoside transmembrane transporter activity |
| GO:0015574 | 0.244 | 3.715 | 0 | 1 | 38 | trehalose transmembrane transporter activity |
| GO:0042947 | 0.244 | 3.715 | 0 | 1 | 38 | glucoside transmembrane transporter activity |
| GO:0015154 | 0.249 | 3.617 | 0 | 1 | 39 | disaccharide transmembrane transporter activity |
| GO:0032182 | 0.255 | 3.524 | 0 | 1 | 40 | small conjugating protein binding |
| GO:0008235 | 0.260 | 3.435 | 0 | 1 | 41 | metalloexopeptidase activity |
| GO:0015291 | 0.273 | 2.001 | 1 | 2 | 140 | secondary active transmembrane transporter activity |
| GO:0003824 | 0.280 | 1.210 | 34 | 37 | 4686 | catalytic activity |
| GO:0016879 | 0.282 | 1.663 | 2 | 3 | 253 | ligase activity, forming carbon-nitrogen bonds |
| GO:0016705 | 0.284 | 1.656 | 2 | 3 | 254 | oxidoreductase activity, acting on paired donors, with incorporation or reduction of molecular oxygen |
| GO:0004197 | 0.292 | 2.985 | 0 | 1 | 47 | cysteine-type endopeptidase activity |
| GO:0080030 | 0.292 | 2.985 | 0 | 1 | 47 | methyl indole-3-acetate esterase activity |
| GO:0080031 | 0.292 | 2.985 | 0 | 1 | 47 | methyl salicylate esterase activity |
| GO:0080032 | 0.292 | 2.985 | 0 | 1 | 47 | methyl jasmonate esterase activity |
| GO:0016788 | 0.294 | 1.338 | 5 | 7 | 739 | hydrolase activity, acting on ester bonds |
| GO:0016874 | 0.332 | 1.406 | 3 | 4 | 398 | ligase activity |
| GO:0015144 | 0.343 | 2.449 | 0 | 1 | 57 | carbohydrate transmembrane transporter activity |
| GO:0016891 | 0.343 | 2.449 | 0 | 1 | 57 | endoribonuclease activity, producing 5'-phosphomonoesters |
| GO:0051119 | 0.343 | 2.449 | 0 | 1 | 57 | sugar transmembrane transporter activity |
| GO:0042277 | 0.348 | 2.406 | 0 | 1 | 58 | peptide binding |
| GO:0008565 | 0.357 | 2.323 | 0 | 1 | 60 | protein transporter activity |
| GO:0051539 | 0.357 | 2.323 | 0 | 1 | 60 | 4 iron, 4 sulfur cluster binding |
| GO:0004177 | 0.362 | 2.284 | 0 | 1 | 61 | aminopeptidase activity |
| GO:0016893 | 0.362 | 2.284 | 0 | 1 | 61 | endonuclease activity, active with either ribo- or deoxyribonucleic acids and producing 5'-phosphomonoesters |
| GO:0004091 | 0.376 | 2.175 | 0 | 1 | 64 | carboxylesterase activity |
| GO:0050253 | 0.376 | 2.175 | 0 | 1 | 64 | retinyl-palmitate esterase activity |
| GO:0005342 | 0.380 | 2.141 | 0 | 1 | 65 | organic acid transmembrane transporter activity |
| GO:0046943 | 0.380 | 2.141 | 0 | 1 | 65 | carboxylic acid transmembrane transporter activity |
| GO:0015103 | 0.380 | 2.141 | 0 | 1 | 65 | inorganic anion transmembrane transporter activity |
| GO:0004519 | 0.386 | 1.526 | 1 | 2 | 182 | endonuclease activity |
| GO:0008094 | 0.394 | 2.044 | 0 | 1 | 68 | DNA-dependent ATPase activity |
| GO:0008237 | 0.399 | 1.484 | 1 | 2 | 187 | metallopeptidase activity |
| GO:0004872 | 0.408 | 1.254 | 3 | 4 | 443 | receptor activity |
| GO:0004521 | 0.412 | 1.928 | 1 | 1 | 72 | endoribonuclease activity |
| GO:0008509 | 0.429 | 1.824 | 1 | 1 | 76 | anion transmembrane transporter activity |
| GO:0000976 | 0.433 | 1.800 | 1 | 1 | 77 | transcription regulatory region sequence-specific DNA binding |
| GO:0003682 | 0.452 | 1.329 | 2 | 2 | 208 | chromatin binding |
| GO:0051540 | 0.458 | 1.667 | 1 | 1 | 83 | metal cluster binding |
| GO:0051536 | 0.458 | 1.667 | 1 | 1 | 83 | iron-sulfur cluster binding |
| GO:0004222 | 0.485 | 1.535 | 1 | 1 | 90 | metalloendopeptidase activity |
| GO:0050660 | 0.518 | 1.392 | 1 | 1 | 99 | flavin adenine dinucleotide binding |
| GO:0004540 | 0.526 | 1.364 | 1 | 1 | 101 | ribonuclease activity |
| GO:0004672 | 0.532 | 1.082 | 3 | 3 | 381 | protein kinase activity |
| GO:0008238 | 0.572 | 1.194 | 1 | 1 | 115 | exopeptidase activity |
| GO:0008026 | 0.585 | 1.153 | 1 | 1 | 119 | ATP-dependent helicase activity |
| GO:0070035 | 0.585 | 1.153 | 1 | 1 | 119 | purine NTP-dependent helicase activity |
| GO:0004518 | 0.592 | 1.014 | 2 | 2 | 270 | nuclease activity |
| GO:0043565 | 0.604 | 0.991 | 2 | 2 | 276 | sequence-specific DNA binding |
| GO:0005102 | 0.612 | 1.070 | 1 | 1 | 128 | receptor binding |
| GO:0016758 | 0.612 | 1.070 | 1 | 1 | 128 | transferase activity, transferring hexosyl groups |
| GO:0044212 | 0.626 | 1.029 | 1 | 1 | 133 | transcription regulatory region DNA binding |
| GO:0001067 | 0.629 | 1.021 | 1 | 1 | 134 | regulatory region nucleic acid binding |
| GO:0000975 | 0.629 | 1.021 | 1 | 1 | 134 | regulatory region DNA binding |
| GO:0052689 | 0.634 | 1.006 | 1 | 1 | 136 | carboxylic ester hydrolase activity |
| GO:0016773 | 0.689 | 0.848 | 4 | 3 | 479 | phosphotransferase activity, alcohol group as acceptor |
| GO:0022804 | 0.690 | 0.841 | 2 | 2 | 323 | active transmembrane transporter activity |
| GO:0036094 | 0.692 | 0.894 | 16 | 15 | 2227 | small molecule binding |
| GO:0016616 | 0.697 | 0.846 | 1 | 1 | 161 | oxidoreductase activity, acting on the CH-OH group of donors, NAD or NADP as acceptor |
| GO:0016757 | 0.701 | 0.835 | 1 | 1 | 163 | transferase activity, transferring glycosyl groups |
| GO:0003723 | 0.706 | 0.843 | 6 | 5 | 798 | RNA binding |
| GO:0016301 | 0.720 | 0.807 | 4 | 3 | 502 | kinase activity |
| GO:0016791 | 0.741 | 0.746 | 1 | 1 | 182 | phosphatase activity |
| GO:0042623 | 0.751 | 0.744 | 3 | 2 | 363 | ATPase activity, coupled |
| GO:0000989 | 0.752 | 0.721 | 1 | 1 | 188 | transcription factor binding transcription factor activity |
| GO:0016614 | 0.758 | 0.710 | 1 | 1 | 191 | oxidoreductase activity, acting on CH-OH group of donors |
| GO:0004386 | 0.763 | 0.699 | 1 | 1 | 194 | helicase activity |
| GO:0000988 | 0.767 | 0.691 | 1 | 1 | 196 | protein binding transcription factor activity |
| GO:0050662 | 0.777 | 0.670 | 1 | 1 | 202 | coenzyme binding |
| GO:0005525 | 0.807 | 0.611 | 2 | 1 | 221 | GTP binding |
| GO:0003700 | 0.808 | 0.656 | 3 | 2 | 409 | sequence-specific DNA binding transcription factor activity |
| GO:0001071 | 0.808 | 0.656 | 3 | 2 | 409 | nucleic acid binding transcription factor activity |
| GO:0019001 | 0.825 | 0.576 | 2 | 1 | 234 | guanyl nucleotide binding |
| GO:0032561 | 0.825 | 0.576 | 2 | 1 | 234 | guanyl ribonucleotide binding |
| GO:0042578 | 0.826 | 0.573 | 2 | 1 | 235 | phosphoric ester hydrolase activity |
| GO:0020037 | 0.827 | 0.571 | 2 | 1 | 236 | heme binding |
| GO:0046906 | 0.833 | 0.561 | 2 | 1 | 240 | tetrapyrrole binding |
| GO:0008092 | 0.835 | 0.556 | 2 | 1 | 242 | cytoskeletal protein binding |
| GO:0022892 | 0.847 | 0.635 | 5 | 3 | 627 | substrate-specific transporter activity |
| GO:0016491 | 0.864 | 0.641 | 6 | 4 | 821 | oxidoreductase activity |
| GO:0046914 | 0.868 | 0.701 | 11 | 8 | 1474 | transition metal ion binding |
| GO:0016887 | 0.870 | 0.559 | 3 | 2 | 475 | ATPase activity |
| GO:0004674 | 0.874 | 0.483 | 2 | 1 | 277 | protein serine/threonine kinase activity |
| GO:0004888 | 0.890 | 0.453 | 2 | 1 | 295 | transmembrane signaling receptor activity |
| GO:0022857 | 0.896 | 0.563 | 5 | 3 | 699 | transmembrane transporter activity |
| GO:0005215 | 0.900 | 0.589 | 6 | 4 | 885 | transporter activity |
| GO:0046872 | 0.913 | 0.704 | 20 | 16 | 2792 | metal ion binding |
| GO:0038023 | 0.914 | 0.407 | 2 | 1 | 327 | signaling receptor activity |
| GO:0022891 | 0.914 | 0.485 | 4 | 2 | 543 | substrate-specific transmembrane transporter activity |
| GO:0005515 | 0.920 | 0.584 | 8 | 5 | 1102 | protein binding |
| GO:0043169 | 0.939 | 0.668 | 21 | 16 | 2887 | cation binding |
| GO:0043167 | 0.939 | 0.668 | 21 | 16 | 2888 | ion binding |
| GO:0016772 | 0.941 | 0.486 | 6 | 3 | 799 | transferase activity, transferring phosphorus-containing groups |
| GO:0003676 | 0.942 | 0.631 | 16 | 11 | 2149 | nucleic acid binding |
| GO:0005509 | 0.948 | 0.335 | 3 | 1 | 393 | calcium ion binding |
| GO:0016740 | 0.962 | 0.528 | 10 | 6 | 1423 | transferase activity |
| GO:0015075 | 0.963 | 0.300 | 3 | 1 | 437 | ion transmembrane transporter activity |
| GO:0003677 | 0.964 | 0.498 | 9 | 5 | 1262 | DNA binding |
| GO:0000166 | 0.965 | 0.577 | 15 | 10 | 2106 | nucleotide binding |
| GO:0097159 | 0.965 | 0.577 | 15 | 10 | 2106 | organic cyclic compound binding |
| GO:1901265 | 0.965 | 0.577 | 15 | 10 | 2106 | nucleoside phosphate binding |
| GO:0004871 | 0.967 | 0.290 | 3 | 1 | 451 | signal transducer activity |
| GO:0060089 | 0.967 | 0.290 | 3 | 1 | 451 | molecular transducer activity |
| GO:0005524 | 0.967 | 0.514 | 11 | 6 | 1454 | ATP binding |
| GO:0032559 | 0.970 | 0.508 | 11 | 6 | 1469 | adenyl ribonucleotide binding |
| GO:0030554 | 0.970 | 0.508 | 11 | 6 | 1469 | adenyl nucleotide binding |
| GO:0008270 | 0.974 | 0.443 | 8 | 4 | 1135 | zinc ion binding |
| GO:0017111 | 0.983 | 0.325 | 6 | 2 | 784 | nucleoside-triphosphatase activity |
| GO:0016462 | 0.985 | 0.316 | 6 | 2 | 804 | pyrophosphatase activity |
| GO:0016818 | 0.985 | 0.315 | 6 | 2 | 805 | hydrolase activity, acting on acid anhydrides, in phosphorus-containing anhydrides |
| GO:0016817 | 0.985 | 0.313 | 6 | 2 | 810 | hydrolase activity, acting on acid anhydrides |
| GO:0035639 | 0.989 | 0.439 | 12 | 6 | 1652 | purine ribonucleoside triphosphate binding |
| GO:0017076 | 0.990 | 0.433 | 12 | 6 | 1669 | purine nucleotide binding |
| GO:0032553 | 0.990 | 0.433 | 12 | 6 | 1669 | ribonucleotide binding |
| GO:0032555 | 0.990 | 0.433 | 12 | 6 | 1669 | purine ribonucleotide binding |
| GO:0005488 | 0.995 | 0.518 | 45 | 37 | 6201 | binding |
