# Supplementary material for: Inducible Defenses Stay Up Late: Temporal Patterns of Immune Gene Expression in Tenebrio molitor
Source: G3 (Bethesda). 2014 Jun 1;4(6):947–55. doi: 10.1534/g3.113.008516 (PMC4065263; doi:10.1534/g3.113.008516)
Supplement: Supporting Information [file supp_g3.113.008516_TableS15.html]

TableS15 

# Inducible Defenses Stay Up Late: Temporal Patterns of Immune Gene Expression in *Tenebrio molitor*

Gene to GO BP test for over-representation

| GOBPID | Pvalue | OddsRatio | ExpCount | Count | Size | Term |
| GO:0008033 | 0.000 | 11.420 | 1 | 6 | 56 | tRNA processing |
| GO:0042221 | 0.000 | 2.423 | 14 | 27 | 1234 | response to chemical stimulus |
| GO:0006011 | 0.000 | 182.876 | 0 | 2 | 3 | UDP-glucose metabolic process |
| GO:0050789 | 0.000 | 2.108 | 43 | 59 | 3856 | regulation of biological process |
| GO:0050794 | 0.000 | 2.085 | 40 | 56 | 3590 | regulation of cellular process |
| GO:0044255 | 0.000 | 3.028 | 6 | 15 | 513 | cellular lipid metabolic process |
| GO:0008610 | 0.001 | 3.452 | 4 | 12 | 355 | lipid biosynthetic process |
| GO:0006732 | 0.001 | 4.386 | 2 | 8 | 183 | coenzyme metabolic process |
| GO:0044281 | 0.001 | 2.152 | 16 | 29 | 1482 | small molecule metabolic process |
| GO:0050896 | 0.001 | 1.973 | 37 | 52 | 3334 | response to stimulus |
| GO:0009058 | 0.001 | 1.974 | 31 | 46 | 2823 | biosynthetic process |
| GO:0034470 | 0.001 | 4.336 | 2 | 8 | 185 | ncRNA processing |
| GO:0044249 | 0.001 | 1.958 | 30 | 45 | 2757 | cellular biosynthetic process |
| GO:0060716 | 0.001 | 60.944 | 0 | 2 | 5 | labyrinthine layer blood vessel development |
| GO:0006767 | 0.001 | 9.549 | 0 | 4 | 43 | water-soluble vitamin metabolic process |
| GO:0003151 | 0.001 | 16.287 | 0 | 3 | 20 | outflow tract morphogenesis |
| GO:0000902 | 0.001 | 2.520 | 8 | 17 | 697 | cell morphogenesis |
| GO:0006399 | 0.002 | 5.249 | 1 | 6 | 114 | tRNA metabolic process |
| GO:0000904 | 0.002 | 2.731 | 6 | 14 | 522 | cell morphogenesis involved in differentiation |
| GO:0061311 | 0.002 | 45.702 | 0 | 2 | 6 | cell surface receptor signaling pathway involved in heart development |
| GO:0090102 | 0.002 | 45.702 | 0 | 2 | 6 | cochlea development |
| GO:0006629 | 0.002 | 2.461 | 8 | 17 | 712 | lipid metabolic process |
| GO:0065007 | 0.002 | 1.919 | 46 | 60 | 4147 | biological regulation |
| GO:0006807 | 0.002 | 1.883 | 36 | 50 | 3249 | nitrogen compound metabolic process |
| GO:0019432 | 0.002 | 13.839 | 0 | 3 | 23 | triglyceride biosynthetic process |
| GO:0006637 | 0.002 | 13.839 | 0 | 3 | 23 | acyl-CoA metabolic process |
| GO:0035383 | 0.002 | 13.839 | 0 | 3 | 23 | thioester metabolic process |
| GO:0008152 | 0.002 | 2.245 | 65 | 77 | 5857 | metabolic process |
| GO:0048667 | 0.002 | 2.763 | 5 | 13 | 476 | cell morphogenesis involved in neuron differentiation |
| GO:0006631 | 0.002 | 3.768 | 2 | 8 | 211 | fatty acid metabolic process |
| GO:0031328 | 0.002 | 2.632 | 6 | 14 | 540 | positive regulation of cellular biosynthetic process |
| GO:0032989 | 0.002 | 2.346 | 9 | 18 | 792 | cellular component morphogenesis |
| GO:0051186 | 0.002 | 3.712 | 2 | 8 | 214 | cofactor metabolic process |
| GO:0060674 | 0.002 | 36.557 | 0 | 2 | 7 | placenta blood vessel development |
| GO:0010467 | 0.003 | 1.875 | 27 | 40 | 2440 | gene expression |
| GO:0009395 | 0.003 | 12.578 | 0 | 3 | 25 | phospholipid catabolic process |
| GO:0006766 | 0.003 | 5.713 | 1 | 5 | 87 | vitamin metabolic process |
| GO:0046460 | 0.003 | 12.030 | 0 | 3 | 26 | neutral lipid biosynthetic process |
| GO:0046463 | 0.003 | 12.030 | 0 | 3 | 26 | acylglycerol biosynthetic process |
| GO:0031325 | 0.003 | 2.294 | 9 | 18 | 808 | positive regulation of cellular metabolic process |
| GO:0010033 | 0.003 | 2.475 | 7 | 15 | 616 | response to organic substance |
| GO:0009891 | 0.003 | 2.554 | 6 | 14 | 555 | positive regulation of biosynthetic process |
| GO:0034641 | 0.003 | 1.820 | 36 | 49 | 3228 | cellular nitrogen compound metabolic process |
| GO:0042491 | 0.003 | 30.461 | 0 | 2 | 8 | auditory receptor cell differentiation |
| GO:0046475 | 0.003 | 30.461 | 0 | 2 | 8 | glycerophospholipid catabolic process |
| GO:0016125 | 0.003 | 5.444 | 1 | 5 | 91 | sterol metabolic process |
| GO:0019222 | 0.003 | 1.855 | 25 | 37 | 2232 | regulation of metabolic process |
| GO:0044242 | 0.004 | 5.319 | 1 | 5 | 93 | cellular lipid catabolic process |
| GO:0006066 | 0.004 | 3.469 | 3 | 8 | 228 | alcohol metabolic process |
| GO:0031323 | 0.004 | 1.870 | 22 | 34 | 2002 | regulation of cellular metabolic process |
| GO:0010043 | 0.004 | 26.106 | 0 | 2 | 9 | response to zinc ion |
| GO:0008210 | 0.004 | 26.106 | 0 | 2 | 9 | estrogen metabolic process |
| GO:0006644 | 0.004 | 3.706 | 2 | 7 | 186 | phospholipid metabolic process |
| GO:0051254 | 0.005 | 2.595 | 5 | 12 | 462 | positive regulation of RNA metabolic process |
| GO:0060113 | 0.005 | 22.840 | 0 | 2 | 10 | inner ear receptor cell differentiation |
| GO:0042490 | 0.005 | 22.840 | 0 | 2 | 10 | mechanoreceptor differentiation |
| GO:0006685 | 0.005 | 22.840 | 0 | 2 | 10 | sphingomyelin catabolic process |
| GO:0010866 | 0.005 | 22.840 | 0 | 2 | 10 | regulation of triglyceride biosynthetic process |
| GO:0010867 | 0.005 | 22.840 | 0 | 2 | 10 | positive regulation of triglyceride biosynthetic process |
| GO:0030148 | 0.005 | 9.534 | 0 | 3 | 32 | sphingolipid biosynthetic process |
| GO:0009893 | 0.006 | 2.137 | 10 | 18 | 860 | positive regulation of metabolic process |
| GO:0046394 | 0.006 | 3.172 | 3 | 8 | 248 | carboxylic acid biosynthetic process |
| GO:0016053 | 0.006 | 3.172 | 3 | 8 | 248 | organic acid biosynthetic process |
| GO:0001892 | 0.006 | 8.916 | 0 | 3 | 34 | embryonic placenta development |
| GO:0006139 | 0.006 | 1.729 | 34 | 46 | 3070 | nucleobase-containing compound metabolic process |
| GO:0046467 | 0.007 | 8.637 | 0 | 3 | 35 | membrane lipid biosynthetic process |
| GO:0044283 | 0.007 | 2.878 | 3 | 9 | 308 | small molecule biosynthetic process |
| GO:0044237 | 0.007 | 1.825 | 56 | 68 | 5100 | cellular metabolic process |
| GO:0031326 | 0.007 | 1.875 | 16 | 26 | 1457 | regulation of cellular biosynthetic process |
| GO:0034660 | 0.007 | 3.067 | 3 | 8 | 256 | ncRNA metabolic process |
| GO:0048732 | 0.007 | 3.360 | 2 | 7 | 204 | gland development |
| GO:0006665 | 0.007 | 5.624 | 1 | 4 | 70 | sphingolipid metabolic process |
| GO:0008209 | 0.007 | 18.267 | 0 | 2 | 12 | androgen metabolic process |
| GO:0009889 | 0.008 | 1.859 | 16 | 26 | 1467 | regulation of biosynthetic process |
| GO:0003007 | 0.008 | 5.539 | 1 | 4 | 71 | heart morphogenesis |
| GO:0001890 | 0.008 | 8.127 | 0 | 3 | 37 | placenta development |
| GO:0008202 | 0.008 | 3.684 | 2 | 6 | 159 | steroid metabolic process |
| GO:0044238 | 0.009 | 1.816 | 59 | 70 | 5339 | primary metabolic process |
| GO:0006641 | 0.009 | 7.673 | 0 | 3 | 39 | triglyceride metabolic process |
| GO:0045935 | 0.009 | 2.356 | 6 | 12 | 505 | positive regulation of nucleobase-containing compound metabolic process |
| GO:0032787 | 0.010 | 2.886 | 3 | 8 | 271 | monocarboxylic acid metabolic process |
| GO:0051173 | 0.010 | 2.331 | 6 | 12 | 510 | positive regulation of nitrogen compound metabolic process |
| GO:0060711 | 0.010 | 15.219 | 0 | 2 | 14 | labyrinthine layer development |
| GO:0006684 | 0.010 | 15.219 | 0 | 2 | 14 | sphingomyelin metabolic process |
| GO:0090207 | 0.010 | 15.219 | 0 | 2 | 14 | regulation of triglyceride metabolic process |
| GO:0090208 | 0.010 | 15.219 | 0 | 2 | 14 | positive regulation of triglyceride metabolic process |
| GO:0008203 | 0.010 | 5.080 | 1 | 4 | 77 | cholesterol metabolic process |
| GO:0016070 | 0.010 | 1.732 | 22 | 32 | 1973 | RNA metabolic process |
| GO:0006633 | 0.010 | 4.057 | 1 | 5 | 120 | fatty acid biosynthetic process |
| GO:0046128 | 0.010 | 3.116 | 2 | 7 | 219 | purine ribonucleoside metabolic process |
| GO:0071704 | 0.011 | 2.114 | 8 | 15 | 710 | organic substance metabolic process |
| GO:0042278 | 0.011 | 3.101 | 2 | 7 | 220 | purine nucleoside metabolic process |
| GO:0006638 | 0.011 | 7.080 | 0 | 3 | 42 | neutral lipid metabolic process |
| GO:0006639 | 0.011 | 7.080 | 0 | 3 | 42 | acylglycerol metabolic process |
| GO:0021707 | 0.011 | Inf | 0 | 1 | 1 | cerebellar granule cell differentiation |
| GO:0060842 | 0.011 | Inf | 0 | 1 | 1 | arterial endothelial cell differentiation |
| GO:0060837 | 0.011 | Inf | 0 | 1 | 1 | blood vessel endothelial cell differentiation |
| GO:0021683 | 0.011 | Inf | 0 | 1 | 1 | cerebellar granular layer morphogenesis |
| GO:0021684 | 0.011 | Inf | 0 | 1 | 1 | cerebellar granular layer formation |
| GO:0032053 | 0.011 | Inf | 0 | 1 | 1 | microtubule basal body organization |
| GO:0060633 | 0.011 | Inf | 0 | 1 | 1 | negative regulation of transcription initiation from RNA polymerase II promoter |
| GO:0042759 | 0.011 | Inf | 0 | 1 | 1 | long-chain fatty acid biosynthetic process |
| GO:0042640 | 0.011 | Inf | 0 | 1 | 1 | anagen |
| GO:0097054 | 0.011 | Inf | 0 | 1 | 1 | L-glutamate biosynthetic process |
| GO:0048076 | 0.011 | Inf | 0 | 1 | 1 | regulation of compound eye pigmentation |
| GO:0045299 | 0.011 | Inf | 0 | 1 | 1 | otolith mineralization |
| GO:0014898 | 0.011 | Inf | 0 | 1 | 1 | cardiac muscle hypertrophy in response to stress |
| GO:0006924 | 0.011 | Inf | 0 | 1 | 1 | activation-induced cell death of T cells |
| GO:0097359 | 0.011 | Inf | 0 | 1 | 1 | UDP-glucosylation |
| GO:0009750 | 0.011 | Inf | 0 | 1 | 1 | response to fructose stimulus |
| GO:0061027 | 0.011 | Inf | 0 | 1 | 1 | umbilical cord development |
| GO:0009854 | 0.011 | Inf | 0 | 1 | 1 | oxidative photosynthetic carbon pathway |
| GO:0097186 | 0.011 | Inf | 0 | 1 | 1 | amelogenesis |
| GO:0097187 | 0.011 | Inf | 0 | 1 | 1 | dentinogenesis |
| GO:0071895 | 0.011 | Inf | 0 | 1 | 1 | odontoblast differentiation |
| GO:0061314 | 0.011 | Inf | 0 | 1 | 1 | Notch signaling involved in heart development |
| GO:0030421 | 0.011 | Inf | 0 | 1 | 1 | defecation |
| GO:0048840 | 0.011 | Inf | 0 | 1 | 1 | otolith development |
| GO:0061156 | 0.011 | Inf | 0 | 1 | 1 | pulmonary artery morphogenesis |
| GO:0035743 | 0.011 | Inf | 0 | 1 | 1 | CD4-positive, alpha-beta T cell cytokine production |
| GO:0035745 | 0.011 | Inf | 0 | 1 | 1 | T-helper 2 cell cytokine production |
| GO:0007185 | 0.011 | Inf | 0 | 1 | 1 | transmembrane receptor protein tyrosine phosphatase signaling pathway |
| GO:0035912 | 0.011 | Inf | 0 | 1 | 1 | dorsal aorta morphogenesis |
| GO:0035907 | 0.011 | Inf | 0 | 1 | 1 | dorsal aorta development |
| GO:0007624 | 0.011 | Inf | 0 | 1 | 1 | ultradian rhythm |
| GO:0036071 | 0.011 | Inf | 0 | 1 | 1 | N-glycan fucosylation |
| GO:0036035 | 0.011 | Inf | 0 | 1 | 1 | osteoclast development |
| GO:0010460 | 0.011 | Inf | 0 | 1 | 1 | positive regulation of heart rate |
| GO:0051643 | 0.011 | Inf | 0 | 1 | 1 | endoplasmic reticulum localization |
| GO:0018307 | 0.011 | Inf | 0 | 1 | 1 | enzyme active site formation |
| GO:0036304 | 0.011 | Inf | 0 | 1 | 1 | umbilical cord morphogenesis |
| GO:0018192 | 0.011 | Inf | 0 | 1 | 1 | enzyme active site formation via L-cysteine persulfide |
| GO:0036179 | 0.011 | Inf | 0 | 1 | 1 | osteoclast maturation |
| GO:0070166 | 0.011 | Inf | 0 | 1 | 1 | enamel mineralization |
| GO:0070231 | 0.011 | Inf | 0 | 1 | 1 | T cell apoptotic process |
| GO:0070232 | 0.011 | Inf | 0 | 1 | 1 | regulation of T cell apoptotic process |
| GO:0070233 | 0.011 | Inf | 0 | 1 | 1 | negative regulation of T cell apoptotic process |
| GO:0070235 | 0.011 | Inf | 0 | 1 | 1 | regulation of activation-induced cell death of T cells |
| GO:0070236 | 0.011 | Inf | 0 | 1 | 1 | negative regulation of activation-induced cell death of T cells |
| GO:2000143 | 0.011 | Inf | 0 | 1 | 1 | negative regulation of DNA-dependent transcription, initiation |
| GO:0003195 | 0.011 | Inf | 0 | 1 | 1 | tricuspid valve formation |
| GO:0003199 | 0.011 | Inf | 0 | 1 | 1 | endocardial cushion to mesenchymal transition involved in heart valve formation |
| GO:0003184 | 0.011 | Inf | 0 | 1 | 1 | pulmonary valve morphogenesis |
| GO:0003186 | 0.011 | Inf | 0 | 1 | 1 | tricuspid valve morphogenesis |
| GO:0003175 | 0.011 | Inf | 0 | 1 | 1 | tricuspid valve development |
| GO:0003177 | 0.011 | Inf | 0 | 1 | 1 | pulmonary valve development |
| GO:0034162 | 0.011 | Inf | 0 | 1 | 1 | toll-like receptor 9 signaling pathway |
| GO:0034163 | 0.011 | Inf | 0 | 1 | 1 | regulation of toll-like receptor 9 signaling pathway |
| GO:0034165 | 0.011 | Inf | 0 | 1 | 1 | positive regulation of toll-like receptor 9 signaling pathway |
| GO:0034155 | 0.011 | Inf | 0 | 1 | 1 | regulation of toll-like receptor 7 signaling pathway |
| GO:0034157 | 0.011 | Inf | 0 | 1 | 1 | positive regulation of toll-like receptor 7 signaling pathway |
| GO:0034123 | 0.011 | Inf | 0 | 1 | 1 | positive regulation of toll-like receptor signaling pathway |
| GO:0003299 | 0.011 | Inf | 0 | 1 | 1 | muscle hypertrophy in response to stress |
| GO:2000820 | 0.011 | Inf | 0 | 1 | 1 | negative regulation of transcription from RNA polymerase II promoter involved in smooth muscle cell differentiation |
| GO:2000551 | 0.011 | Inf | 0 | 1 | 1 | regulation of T-helper 2 cell cytokine production |
| GO:2000553 | 0.011 | Inf | 0 | 1 | 1 | positive regulation of T-helper 2 cell cytokine production |
| GO:0006643 | 0.011 | 4.943 | 1 | 4 | 79 | membrane lipid metabolic process |
| GO:0009119 | 0.011 | 3.071 | 2 | 7 | 222 | ribonucleoside metabolic process |
| GO:0048518 | 0.012 | 1.785 | 17 | 26 | 1516 | positive regulation of biological process |
| GO:0007435 | 0.012 | 4.878 | 1 | 4 | 80 | salivary gland morphogenesis |
| GO:0048813 | 0.013 | 3.821 | 1 | 5 | 127 | dendrite morphogenesis |
| GO:0007431 | 0.013 | 3.821 | 1 | 5 | 127 | salivary gland development |
| GO:0046503 | 0.013 | 13.042 | 0 | 2 | 16 | glycerolipid catabolic process |
| GO:0007409 | 0.013 | 2.433 | 4 | 10 | 403 | axonogenesis |
| GO:0051259 | 0.013 | 3.789 | 1 | 5 | 128 | protein oligomerization |
| GO:0035272 | 0.014 | 3.727 | 1 | 5 | 130 | exocrine system development |
| GO:0009116 | 0.015 | 2.905 | 3 | 7 | 234 | nucleoside metabolic process |
| GO:0045463 | 0.015 | 12.171 | 0 | 2 | 17 | R8 cell development |
| GO:0045168 | 0.015 | 4.518 | 1 | 4 | 86 | cell-cell signaling involved in cell fate commitment |
| GO:0009605 | 0.015 | 2.018 | 8 | 15 | 740 | response to external stimulus |
| GO:0080090 | 0.015 | 1.682 | 21 | 31 | 1944 | regulation of primary metabolic process |
| GO:0019637 | 0.016 | 2.060 | 7 | 14 | 674 | organophosphate metabolic process |
| GO:0051716 | 0.016 | 1.644 | 25 | 35 | 2277 | cellular response to stimulus |
| GO:0051336 | 0.016 | 2.637 | 3 | 8 | 295 | regulation of hydrolase activity |
| GO:0065009 | 0.016 | 2.050 | 7 | 14 | 677 | regulation of molecular function |
| GO:0006516 | 0.016 | 11.409 | 0 | 2 | 18 | glycoprotein catabolic process |
| GO:0009225 | 0.016 | 11.409 | 0 | 2 | 18 | nucleotide-sugar metabolic process |
| GO:0016318 | 0.016 | 11.409 | 0 | 2 | 18 | ommatidial rotation |
| GO:0009653 | 0.017 | 1.734 | 16 | 25 | 1484 | anatomical structure morphogenesis |
| GO:0015980 | 0.017 | 3.582 | 1 | 5 | 135 | energy derivation by oxidation of organic compounds |
| GO:0030154 | 0.017 | 1.695 | 19 | 28 | 1719 | cell differentiation |
| GO:0048858 | 0.017 | 2.159 | 6 | 12 | 547 | cell projection morphogenesis |
| GO:0048812 | 0.017 | 2.234 | 5 | 11 | 483 | neuron projection morphogenesis |
| GO:0022612 | 0.018 | 4.255 | 1 | 4 | 91 | gland morphogenesis |
| GO:0051345 | 0.018 | 3.069 | 2 | 6 | 189 | positive regulation of hydrolase activity |
| GO:1901135 | 0.018 | 2.017 | 8 | 14 | 687 | carbohydrate derivative metabolic process |
| GO:0042078 | 0.018 | 10.736 | 0 | 2 | 19 | germ-line stem cell division |
| GO:0048522 | 0.018 | 1.747 | 15 | 23 | 1343 | positive regulation of cellular process |
| GO:0032990 | 0.019 | 2.125 | 6 | 12 | 555 | cell part morphogenesis |
| GO:0019752 | 0.019 | 2.057 | 7 | 13 | 623 | carboxylic acid metabolic process |
| GO:0043436 | 0.019 | 2.057 | 7 | 13 | 623 | oxoacid metabolic process |
| GO:0006367 | 0.020 | 5.628 | 1 | 3 | 52 | transcription initiation from RNA polymerase II promoter |
| GO:0006082 | 0.020 | 2.035 | 7 | 13 | 629 | organic acid metabolic process |
| GO:0010604 | 0.021 | 1.936 | 8 | 15 | 768 | positive regulation of macromolecule metabolic process |
| GO:0007411 | 0.021 | 2.510 | 3 | 8 | 309 | axon guidance |
| GO:0048666 | 0.021 | 2.025 | 7 | 13 | 632 | neuron development |
| GO:0048869 | 0.021 | 1.645 | 20 | 29 | 1831 | cellular developmental process |
| GO:0045893 | 0.021 | 2.241 | 5 | 10 | 435 | positive regulation of transcription, DNA-dependent |
| GO:0051262 | 0.022 | 5.406 | 1 | 3 | 54 | protein tetramerization |
| GO:0060237 | 0.022 | 90.422 | 0 | 1 | 2 | regulation of fungal-type cell wall organization |
| GO:0070970 | 0.022 | 90.422 | 0 | 1 | 2 | interleukin-2 secretion |
| GO:0021551 | 0.022 | 90.422 | 0 | 1 | 2 | central nervous system morphogenesis |
| GO:2000980 | 0.022 | 90.422 | 0 | 1 | 2 | regulation of inner ear receptor cell differentiation |
| GO:0021681 | 0.022 | 90.422 | 0 | 1 | 2 | cerebellar granular layer development |
| GO:0042104 | 0.022 | 90.422 | 0 | 1 | 2 | positive regulation of activated T cell proliferation |
| GO:0060658 | 0.022 | 90.422 | 0 | 1 | 2 | nipple morphogenesis |
| GO:0060618 | 0.022 | 90.422 | 0 | 1 | 2 | nipple development |
| GO:0006771 | 0.022 | 90.422 | 0 | 1 | 2 | riboflavin metabolic process |
| GO:0050798 | 0.022 | 90.422 | 0 | 1 | 2 | activated T cell proliferation |
| GO:0050783 | 0.022 | 90.422 | 0 | 1 | 2 | cocaine metabolic process |
| GO:0060947 | 0.022 | 90.422 | 0 | 1 | 2 | cardiac vascular smooth muscle cell differentiation |
| GO:0060948 | 0.022 | 90.422 | 0 | 1 | 2 | cardiac vascular smooth muscle cell development |
| GO:0006669 | 0.022 | 90.422 | 0 | 1 | 2 | sphinganine-1-phosphate biosynthetic process |
| GO:0009398 | 0.022 | 90.422 | 0 | 1 | 2 | FMN biosynthetic process |
| GO:0097084 | 0.022 | 90.422 | 0 | 1 | 2 | vascular smooth muscle cell development |
| GO:0032252 | 0.022 | 90.422 | 0 | 1 | 2 | secretory granule localization |
| GO:0048073 | 0.022 | 90.422 | 0 | 1 | 2 | regulation of eye pigmentation |
| GO:0014887 | 0.022 | 90.422 | 0 | 1 | 2 | cardiac muscle adaptation |
| GO:0009231 | 0.022 | 90.422 | 0 | 1 | 2 | riboflavin biosynthetic process |
| GO:0050910 | 0.022 | 90.422 | 0 | 1 | 2 | detection of mechanical stimulus involved in sensory perception of sound |
| GO:0043029 | 0.022 | 90.422 | 0 | 1 | 2 | T cell homeostasis |
| GO:2001212 | 0.022 | 90.422 | 0 | 1 | 2 | regulation of vasculogenesis |
| GO:0045631 | 0.022 | 90.422 | 0 | 1 | 2 | regulation of mechanoreceptor differentiation |
| GO:0045607 | 0.022 | 90.422 | 0 | 1 | 2 | regulation of auditory receptor cell differentiation |
| GO:0048820 | 0.022 | 90.422 | 0 | 1 | 2 | hair follicle maturation |
| GO:0007415 | 0.022 | 90.422 | 0 | 1 | 2 | defasciculation of motor neuron axon |
| GO:0046006 | 0.022 | 90.422 | 0 | 1 | 2 | regulation of activated T cell proliferation |
| GO:0033327 | 0.022 | 90.422 | 0 | 1 | 2 | Leydig cell differentiation |
| GO:0043569 | 0.022 | 90.422 | 0 | 1 | 2 | negative regulation of insulin-like growth factor receptor signaling pathway |
| GO:0035910 | 0.022 | 90.422 | 0 | 1 | 2 | ascending aorta morphogenesis |
| GO:0035905 | 0.022 | 90.422 | 0 | 1 | 2 | ascending aorta development |
| GO:0046322 | 0.022 | 90.422 | 0 | 1 | 2 | negative regulation of fatty acid oxidation |
| GO:0046448 | 0.022 | 90.422 | 0 | 1 | 2 | tropane alkaloid metabolic process |
| GO:0090163 | 0.022 | 90.422 | 0 | 1 | 2 | establishment of epithelial cell planar polarity |
| GO:0000083 | 0.022 | 90.422 | 0 | 1 | 2 | regulation of transcription involved in G1/S phase of mitotic cell cycle |
| GO:0010204 | 0.022 | 90.422 | 0 | 1 | 2 | defense response signaling pathway, resistance gene-independent |
| GO:0070229 | 0.022 | 90.422 | 0 | 1 | 2 | negative regulation of lymphocyte apoptotic process |
| GO:0003150 | 0.022 | 90.422 | 0 | 1 | 2 | muscular septum morphogenesis |
| GO:0003188 | 0.022 | 90.422 | 0 | 1 | 2 | heart valve formation |
| GO:0003171 | 0.022 | 90.422 | 0 | 1 | 2 | atrioventricular valve development |
| GO:0034154 | 0.022 | 90.422 | 0 | 1 | 2 | toll-like receptor 7 signaling pathway |
| GO:0003222 | 0.022 | 90.422 | 0 | 1 | 2 | ventricular trabecula myocardium morphogenesis |
| GO:0003214 | 0.022 | 90.422 | 0 | 1 | 2 | cardiac left ventricle morphogenesis |
| GO:0055012 | 0.022 | 90.422 | 0 | 1 | 2 | ventricular cardiac muscle cell differentiation |
| GO:0055015 | 0.022 | 90.422 | 0 | 1 | 2 | ventricular cardiac muscle cell development |
| GO:0046513 | 0.022 | 9.604 | 0 | 2 | 21 | ceramide biosynthetic process |
| GO:0007165 | 0.022 | 1.658 | 19 | 27 | 1678 | signal transduction |
| GO:0046483 | 0.022 | 2.007 | 7 | 13 | 637 | heterocycle metabolic process |
| GO:0007548 | 0.023 | 2.891 | 2 | 6 | 200 | sex differentiation |
| GO:0046890 | 0.024 | 5.201 | 1 | 3 | 56 | regulation of lipid biosynthetic process |
| GO:0035019 | 0.024 | 9.122 | 0 | 2 | 22 | somatic stem cell maintenance |
| GO:0046486 | 0.024 | 3.228 | 2 | 5 | 149 | glycerolipid metabolic process |
| GO:0048731 | 0.024 | 1.605 | 22 | 31 | 2013 | system development |
| GO:0048468 | 0.025 | 1.745 | 13 | 20 | 1151 | cell development |
| GO:0007154 | 0.025 | 1.603 | 22 | 31 | 2015 | cell communication |
| GO:0051171 | 0.025 | 1.659 | 17 | 25 | 1538 | regulation of nitrogen compound metabolic process |
| GO:0045017 | 0.026 | 3.772 | 1 | 4 | 102 | glycerolipid biosynthetic process |
| GO:0006541 | 0.026 | 8.687 | 0 | 2 | 23 | glutamine metabolic process |
| GO:0030433 | 0.026 | 8.687 | 0 | 2 | 23 | ER-associated protein catabolic process |
| GO:0042180 | 0.027 | 1.946 | 7 | 13 | 655 | cellular ketone metabolic process |
| GO:0016358 | 0.028 | 3.118 | 2 | 5 | 154 | dendrite development |
| GO:0042476 | 0.029 | 8.291 | 0 | 2 | 24 | odontogenesis |
| GO:0030182 | 0.029 | 1.879 | 8 | 14 | 732 | neuron differentiation |
| GO:0048513 | 0.029 | 1.657 | 16 | 23 | 1403 | organ development |
| GO:0023052 | 0.030 | 1.581 | 22 | 30 | 1961 | signaling |
| GO:0010038 | 0.030 | 3.587 | 1 | 4 | 107 | response to metal ion |
| GO:0043085 | 0.031 | 2.317 | 4 | 8 | 333 | positive regulation of catalytic activity |
| GO:0032968 | 0.031 | 7.930 | 0 | 2 | 25 | positive regulation of transcription elongation from RNA polymerase II promoter |
| GO:0002218 | 0.031 | 7.930 | 0 | 2 | 25 | activation of innate immune response |
| GO:0031638 | 0.031 | 7.930 | 0 | 2 | 25 | zymogen activation |
| GO:0008361 | 0.031 | 4.669 | 1 | 3 | 62 | regulation of cell size |
| GO:0031175 | 0.031 | 2.019 | 6 | 11 | 530 | neuron projection development |
| GO:0010628 | 0.032 | 2.080 | 5 | 10 | 466 | positive regulation of gene expression |
| GO:0031958 | 0.033 | 45.206 | 0 | 1 | 3 | corticosteroid receptor signaling pathway |
| GO:0032025 | 0.033 | 45.206 | 0 | 1 | 3 | response to cobalt ion |
| GO:0060628 | 0.033 | 45.206 | 0 | 1 | 3 | regulation of ER to Golgi vesicle-mediated transport |
| GO:0042780 | 0.033 | 45.206 | 0 | 1 | 3 | tRNA 3'-end processing |
| GO:0040036 | 0.033 | 45.206 | 0 | 1 | 3 | regulation of fibroblast growth factor receptor signaling pathway |
| GO:0019255 | 0.033 | 45.206 | 0 | 1 | 3 | glucose 1-phosphate metabolic process |
| GO:0019287 | 0.033 | 45.206 | 0 | 1 | 3 | isopentenyl diphosphate biosynthetic process, mevalonate pathway |
| GO:0006668 | 0.033 | 45.206 | 0 | 1 | 3 | sphinganine-1-phosphate metabolic process |
| GO:0009441 | 0.033 | 45.206 | 0 | 1 | 3 | glycolate metabolic process |
| GO:0042921 | 0.033 | 45.206 | 0 | 1 | 3 | glucocorticoid receptor signaling pathway |
| GO:0014888 | 0.033 | 45.206 | 0 | 1 | 3 | striated muscle adaptation |
| GO:0009240 | 0.033 | 45.206 | 0 | 1 | 3 | isopentenyl diphosphate biosynthetic process |
| GO:0009853 | 0.033 | 45.206 | 0 | 1 | 3 | photorespiration |
| GO:0007047 | 0.033 | 45.206 | 0 | 1 | 3 | cellular cell wall organization |
| GO:0035886 | 0.033 | 45.206 | 0 | 1 | 3 | vascular smooth muscle cell differentiation |
| GO:0002093 | 0.033 | 45.206 | 0 | 1 | 3 | auditory receptor cell morphogenesis |
| GO:0071907 | 0.033 | 45.206 | 0 | 1 | 3 | determination of digestive tract left/right asymmetry |
| GO:0010042 | 0.033 | 45.206 | 0 | 1 | 3 | response to manganese ion |
| GO:0046490 | 0.033 | 45.206 | 0 | 1 | 3 | isopentenyl diphosphate metabolic process |
| GO:0046498 | 0.033 | 45.206 | 0 | 1 | 3 | S-adenosylhomocysteine metabolic process |
| GO:0090381 | 0.033 | 45.206 | 0 | 1 | 3 | regulation of heart induction |
| GO:0046444 | 0.033 | 45.206 | 0 | 1 | 3 | FMN metabolic process |
| GO:0036065 | 0.033 | 45.206 | 0 | 1 | 3 | fucosylation |
| GO:0046296 | 0.033 | 45.206 | 0 | 1 | 3 | glycolate catabolic process |
| GO:0002828 | 0.033 | 45.206 | 0 | 1 | 3 | regulation of type 2 immune response |
| GO:0033578 | 0.033 | 45.206 | 0 | 1 | 3 | protein glycosylation in Golgi |
| GO:1901320 | 0.033 | 45.206 | 0 | 1 | 3 | negative regulation of heart induction |
| GO:0018198 | 0.033 | 45.206 | 0 | 1 | 3 | peptidyl-cysteine modification |
| GO:0060088 | 0.033 | 45.206 | 0 | 1 | 3 | auditory receptor cell stereocilium organization |
| GO:0060045 | 0.033 | 45.206 | 0 | 1 | 3 | positive regulation of cardiac muscle cell proliferation |
| GO:0070227 | 0.033 | 45.206 | 0 | 1 | 3 | lymphocyte apoptotic process |
| GO:0070228 | 0.033 | 45.206 | 0 | 1 | 3 | regulation of lymphocyte apoptotic process |
| GO:2000107 | 0.033 | 45.206 | 0 | 1 | 3 | negative regulation of leukocyte apoptotic process |
| GO:0003129 | 0.033 | 45.206 | 0 | 1 | 3 | heart induction |
| GO:0003136 | 0.033 | 45.206 | 0 | 1 | 3 | negative regulation of heart induction by canonical Wnt receptor signaling pathway |
| GO:0031505 | 0.033 | 45.206 | 0 | 1 | 3 | fungal-type cell wall organization |
| GO:0034121 | 0.033 | 45.206 | 0 | 1 | 3 | regulation of toll-like receptor signaling pathway |
| GO:0003215 | 0.033 | 45.206 | 0 | 1 | 3 | cardiac right ventricle morphogenesis |
| GO:0060413 | 0.033 | 45.206 | 0 | 1 | 3 | atrial septum morphogenesis |
| GO:0045465 | 0.033 | 7.598 | 0 | 2 | 26 | R8 cell differentiation |
| GO:0030149 | 0.033 | 7.598 | 0 | 2 | 26 | sphingolipid catabolic process |
| GO:0008354 | 0.033 | 7.598 | 0 | 2 | 26 | germ cell migration |
| GO:0008654 | 0.033 | 3.484 | 1 | 4 | 110 | phospholipid biosynthetic process |
| GO:0042330 | 0.034 | 2.158 | 4 | 9 | 403 | taxis |
| GO:0016485 | 0.034 | 3.451 | 1 | 4 | 111 | protein processing |
| GO:0045137 | 0.035 | 2.918 | 2 | 5 | 164 | development of primary sexual characteristics |
| GO:0043170 | 0.035 | 1.506 | 46 | 55 | 4154 | macromolecule metabolic process |
| GO:0050790 | 0.035 | 1.978 | 6 | 11 | 540 | regulation of catalytic activity |
| GO:0042558 | 0.035 | 7.293 | 0 | 2 | 27 | pteridine-containing compound metabolic process |
| GO:0032786 | 0.035 | 7.293 | 0 | 2 | 27 | positive regulation of DNA-dependent transcription, elongation |
| GO:0006084 | 0.036 | 4.370 | 1 | 3 | 66 | acetyl-CoA metabolic process |
| GO:0010557 | 0.037 | 2.033 | 5 | 10 | 476 | positive regulation of macromolecule biosynthetic process |
| GO:0007275 | 0.038 | 1.504 | 29 | 37 | 2584 | multicellular organismal development |
| GO:0042364 | 0.038 | 7.012 | 0 | 2 | 28 | water-soluble vitamin biosynthetic process |
| GO:0030879 | 0.038 | 7.012 | 0 | 2 | 28 | mammary gland development |
| GO:0046889 | 0.038 | 7.012 | 0 | 2 | 28 | positive regulation of lipid biosynthetic process |
| GO:0019219 | 0.038 | 1.595 | 17 | 24 | 1517 | regulation of nucleobase-containing compound metabolic process |
| GO:0006650 | 0.038 | 3.325 | 1 | 4 | 115 | glycerophospholipid metabolic process |
| GO:0009308 | 0.039 | 3.295 | 1 | 4 | 116 | amine metabolic process |
| GO:0009798 | 0.040 | 2.810 | 2 | 5 | 170 | axis specification |
| GO:0016042 | 0.040 | 2.810 | 2 | 5 | 170 | lipid catabolic process |
| GO:0048568 | 0.040 | 3.266 | 1 | 4 | 117 | embryonic organ development |
| GO:0051604 | 0.040 | 3.266 | 1 | 4 | 117 | protein maturation |
| GO:0034243 | 0.040 | 6.752 | 0 | 2 | 29 | regulation of transcription elongation from RNA polymerase II promoter |
| GO:0001817 | 0.042 | 4.107 | 1 | 3 | 70 | regulation of cytokine production |
| GO:0007399 | 0.042 | 1.629 | 13 | 20 | 1220 | nervous system development |
| GO:0030030 | 0.043 | 1.814 | 8 | 13 | 698 | cell projection organization |
| GO:0006396 | 0.043 | 1.912 | 6 | 11 | 557 | RNA processing |
| GO:0050708 | 0.043 | 6.510 | 0 | 2 | 30 | regulation of protein secretion |
| GO:0046466 | 0.043 | 6.510 | 0 | 2 | 30 | membrane lipid catabolic process |
| GO:0033865 | 0.043 | 6.510 | 0 | 2 | 30 | nucleoside bisphosphate metabolic process |
| GO:0009987 | 0.043 | 2.098 | 79 | 85 | 7174 | cellular process |
| GO:0060122 | 0.044 | 30.133 | 0 | 1 | 4 | inner ear receptor stereocilium organization |
| GO:0060117 | 0.044 | 30.133 | 0 | 1 | 4 | auditory receptor cell development |
| GO:0021702 | 0.044 | 30.133 | 0 | 1 | 4 | cerebellar Purkinje cell differentiation |
| GO:0034505 | 0.044 | 30.133 | 0 | 1 | 4 | tooth mineralization |
| GO:0021533 | 0.044 | 30.133 | 0 | 1 | 4 | cell differentiation in hindbrain |
| GO:0021692 | 0.044 | 30.133 | 0 | 1 | 4 | cerebellar Purkinje cell layer morphogenesis |
| GO:0021694 | 0.044 | 30.133 | 0 | 1 | 4 | cerebellar Purkinje cell layer formation |
| GO:0006122 | 0.044 | 30.133 | 0 | 1 | 4 | mitochondrial electron transport, ubiquinol to cytochrome c |
| GO:0042760 | 0.044 | 30.133 | 0 | 1 | 4 | very long-chain fatty acid catabolic process |
| GO:0006884 | 0.044 | 30.133 | 0 | 1 | 4 | cell volume homeostasis |
| GO:0060976 | 0.044 | 30.133 | 0 | 1 | 4 | coronary vasculature development |
| GO:0060977 | 0.044 | 30.133 | 0 | 1 | 4 | coronary vasculature morphogenesis |
| GO:0001748 | 0.044 | 30.133 | 0 | 1 | 4 | optic lobe placode development |
| GO:0014855 | 0.044 | 30.133 | 0 | 1 | 4 | striated muscle cell proliferation |
| GO:0045717 | 0.044 | 30.133 | 0 | 1 | 4 | negative regulation of fatty acid biosynthetic process |
| GO:0050974 | 0.044 | 30.133 | 0 | 1 | 4 | detection of mechanical stimulus involved in sensory perception |
| GO:0035309 | 0.044 | 30.133 | 0 | 1 | 4 | wing and notum subfield formation |
| GO:0048106 | 0.044 | 30.133 | 0 | 1 | 4 | establishment of thoracic bristle planar orientation |
| GO:0071852 | 0.044 | 30.133 | 0 | 1 | 4 | fungal-type cell wall organization or biogenesis |
| GO:0071887 | 0.044 | 30.133 | 0 | 1 | 4 | leukocyte apoptotic process |
| GO:0071555 | 0.044 | 30.133 | 0 | 1 | 4 | cell wall organization |
| GO:0007394 | 0.044 | 30.133 | 0 | 1 | 4 | dorsal closure, elongation of leading edge cells |
| GO:0002369 | 0.044 | 30.133 | 0 | 1 | 4 | T cell cytokine production |
| GO:0002724 | 0.044 | 30.133 | 0 | 1 | 4 | regulation of T cell cytokine production |
| GO:0002726 | 0.044 | 30.133 | 0 | 1 | 4 | positive regulation of T cell cytokine production |
| GO:0002711 | 0.044 | 30.133 | 0 | 1 | 4 | positive regulation of T cell mediated immunity |
| GO:0002709 | 0.044 | 30.133 | 0 | 1 | 4 | regulation of T cell mediated immunity |
| GO:0051593 | 0.044 | 30.133 | 0 | 1 | 4 | response to folic acid |
| GO:0046500 | 0.044 | 30.133 | 0 | 1 | 4 | S-adenosylmethionine metabolic process |
| GO:0000480 | 0.044 | 30.133 | 0 | 1 | 4 | endonucleolytic cleavage in 5'-ETS of tricistronic rRNA transcript (SSU-rRNA, 5.8S rRNA, LSU-rRNA) |
| GO:0000472 | 0.044 | 30.133 | 0 | 1 | 4 | endonucleolytic cleavage to generate mature 5'-end of SSU-rRNA from (SSU-rRNA, 5.8S rRNA, LSU-rRNA) |
| GO:0000478 | 0.044 | 30.133 | 0 | 1 | 4 | endonucleolytic cleavage involved in rRNA processing |
| GO:0000479 | 0.044 | 30.133 | 0 | 1 | 4 | endonucleolytic cleavage of tricistronic rRNA transcript (SSU-rRNA, 5.8S rRNA, LSU-rRNA) |
| GO:0000447 | 0.044 | 30.133 | 0 | 1 | 4 | endonucleolytic cleavage in ITS1 to separate SSU-rRNA from 5.8S rRNA and LSU-rRNA from tricistronic rRNA transcript (SSU-rRNA, 5.8S rRNA, LSU-rRNA) |
| GO:0010662 | 0.044 | 30.133 | 0 | 1 | 4 | regulation of striated muscle cell apoptotic process |
| GO:0010664 | 0.044 | 30.133 | 0 | 1 | 4 | negative regulation of striated muscle cell apoptotic process |
| GO:0010665 | 0.044 | 30.133 | 0 | 1 | 4 | regulation of cardiac muscle cell apoptotic process |
| GO:0010667 | 0.044 | 30.133 | 0 | 1 | 4 | negative regulation of cardiac muscle cell apoptotic process |
| GO:0010658 | 0.044 | 30.133 | 0 | 1 | 4 | striated muscle cell apoptotic process |
| GO:0010659 | 0.044 | 30.133 | 0 | 1 | 4 | cardiac muscle cell apoptotic process |
| GO:0060027 | 0.044 | 30.133 | 0 | 1 | 4 | convergent extension involved in gastrulation |
| GO:0060043 | 0.044 | 30.133 | 0 | 1 | 4 | regulation of cardiac muscle cell proliferation |
| GO:0060038 | 0.044 | 30.133 | 0 | 1 | 4 | cardiac muscle cell proliferation |
| GO:2000106 | 0.044 | 30.133 | 0 | 1 | 4 | regulation of leukocyte apoptotic process |
| GO:0034471 | 0.044 | 30.133 | 0 | 1 | 4 | ncRNA 5'-end processing |
| GO:0003170 | 0.044 | 30.133 | 0 | 1 | 4 | heart valve development |
| GO:0031585 | 0.044 | 30.133 | 0 | 1 | 4 | regulation of inositol 1,4,5-trisphosphate-sensitive calcium-release channel activity |
| GO:0003179 | 0.044 | 30.133 | 0 | 1 | 4 | heart valve morphogenesis |
| GO:0000967 | 0.044 | 30.133 | 0 | 1 | 4 | rRNA 5'-end processing |
| GO:0042092 | 0.044 | 30.133 | 0 | 1 | 4 | type 2 immune response |
| GO:0034227 | 0.044 | 30.133 | 0 | 1 | 4 | tRNA thio-modification |
| GO:0060427 | 0.044 | 30.133 | 0 | 1 | 4 | lung connective tissue development |
| GO:0071310 | 0.044 | 2.145 | 4 | 8 | 358 | cellular response to organic substance |
| GO:0009887 | 0.044 | 1.849 | 7 | 12 | 630 | organ morphogenesis |
| GO:2000112 | 0.044 | 1.591 | 15 | 22 | 1381 | regulation of cellular macromolecule biosynthetic process |
| GO:0006695 | 0.046 | 6.284 | 0 | 2 | 31 | cholesterol biosynthetic process |
| GO:0009110 | 0.046 | 6.284 | 0 | 2 | 31 | vitamin biosynthetic process |
| GO:0031290 | 0.046 | 6.284 | 0 | 2 | 31 | retinal ganglion cell axon guidance |
| GO:0048699 | 0.046 | 1.755 | 9 | 14 | 778 | generation of neurons |
| GO:0065003 | 0.047 | 1.940 | 5 | 10 | 497 | macromolecular complex assembly |
| GO:0010556 | 0.048 | 1.575 | 15 | 22 | 1392 | regulation of macromolecule biosynthetic process |
| GO:0007367 | 0.048 | 6.074 | 0 | 2 | 32 | segment polarity determination |
| GO:0048839 | 0.048 | 6.074 | 0 | 2 | 32 | inner ear development |
| GO:0007311 | 0.048 | 6.074 | 0 | 2 | 32 | maternal specification of dorsal/ventral axis, oocyte, germ-line encoded |
| GO:0051289 | 0.048 | 6.074 | 0 | 2 | 32 | protein homotetramerization |
| GO:0046686 | 0.048 | 6.074 | 0 | 2 | 32 | response to cadmium ion |
| GO:0030031 | 0.049 | 3.047 | 1 | 4 | 125 | cell projection assembly |
| GO:0006355 | 0.049 | 1.596 | 14 | 20 | 1241 | regulation of transcription, DNA-dependent |
| GO:2001141 | 0.050 | 1.593 | 14 | 20 | 1243 | regulation of RNA biosynthetic process |
| GO:0043933 | 0.050 | 1.854 | 6 | 11 | 573 | macromolecular complex subunit organization |
| GO:0006935 | 0.052 | 2.071 | 4 | 8 | 370 | chemotaxis |
| GO:0006352 | 0.052 | 3.767 | 1 | 3 | 76 | DNA-dependent transcription, initiation |
| GO:0009950 | 0.052 | 3.767 | 1 | 3 | 76 | dorsal/ventral axis specification |
| GO:0051260 | 0.052 | 3.767 | 1 | 3 | 76 | protein homooligomerization |
| GO:0090304 | 0.052 | 1.459 | 28 | 36 | 2556 | nucleic acid metabolic process |
| GO:0022008 | 0.053 | 1.660 | 10 | 16 | 943 | neurogenesis |
| GO:0009994 | 0.053 | 2.972 | 1 | 4 | 128 | oocyte differentiation |
| GO:0048583 | 0.053 | 1.634 | 11 | 17 | 1020 | regulation of response to stimulus |
| GO:0046331 | 0.053 | 3.715 | 1 | 3 | 77 | lateral inhibition |
| GO:0045089 | 0.054 | 5.693 | 0 | 2 | 34 | positive regulation of innate immune response |
| GO:0060119 | 0.054 | 22.597 | 0 | 1 | 5 | inner ear receptor cell development |
| GO:2000678 | 0.054 | 22.597 | 0 | 1 | 5 | negative regulation of transcription regulatory region DNA binding |
| GO:0042438 | 0.054 | 22.597 | 0 | 1 | 5 | melanin biosynthetic process |
| GO:0021697 | 0.054 | 22.597 | 0 | 1 | 5 | cerebellar cortex formation |
| GO:0042727 | 0.054 | 22.597 | 0 | 1 | 5 | flavin-containing compound biosynthetic process |
| GO:0032497 | 0.054 | 22.597 | 0 | 1 | 5 | detection of lipopolysaccharide |
| GO:0050709 | 0.054 | 22.597 | 0 | 1 | 5 | negative regulation of protein secretion |
| GO:0032480 | 0.054 | 22.597 | 0 | 1 | 5 | negative regulation of type I interferon production |
| GO:0006537 | 0.054 | 22.597 | 0 | 1 | 5 | glutamate biosynthetic process |
| GO:0097056 | 0.054 | 22.597 | 0 | 1 | 5 | selenocysteinyl-tRNA(Sec) biosynthetic process |
| GO:0050665 | 0.054 | 22.597 | 0 | 1 | 5 | hydrogen peroxide biosynthetic process |
| GO:0001702 | 0.054 | 22.597 | 0 | 1 | 5 | gastrulation with mouth forming second |
| GO:0001561 | 0.054 | 22.597 | 0 | 1 | 5 | fatty acid alpha-oxidation |
| GO:0001659 | 0.054 | 22.597 | 0 | 1 | 5 | temperature homeostasis |
| GO:0061316 | 0.054 | 22.597 | 0 | 1 | 5 | canonical Wnt receptor signaling pathway involved in heart development |
| GO:0035262 | 0.054 | 22.597 | 0 | 1 | 5 | gonad morphogenesis |
| GO:0045922 | 0.054 | 22.597 | 0 | 1 | 5 | negative regulation of fatty acid metabolic process |
| GO:0009997 | 0.054 | 22.597 | 0 | 1 | 5 | negative regulation of cardioblast cell fate specification |
| GO:0002097 | 0.054 | 22.597 | 0 | 1 | 5 | tRNA wobble base modification |
| GO:0002098 | 0.054 | 22.597 | 0 | 1 | 5 | tRNA wobble uridine modification |
| GO:0007220 | 0.054 | 22.597 | 0 | 1 | 5 | Notch receptor processing |
| GO:0002456 | 0.054 | 22.597 | 0 | 1 | 5 | T cell mediated immunity |
| GO:0007567 | 0.054 | 22.597 | 0 | 1 | 5 | parturition |
| GO:0002720 | 0.054 | 22.597 | 0 | 1 | 5 | positive regulation of cytokine production involved in immune response |
| GO:0051856 | 0.054 | 22.597 | 0 | 1 | 5 | adhesion to symbiont |
| GO:0002824 | 0.054 | 22.597 | 0 | 1 | 5 | positive regulation of adaptive immune response based on somatic recombination of immune receptors built from immunoglobulin superfamily domains |
| GO:0046655 | 0.054 | 22.597 | 0 | 1 | 5 | folic acid metabolic process |
| GO:0000462 | 0.054 | 22.597 | 0 | 1 | 5 | maturation of SSU-rRNA from tricistronic rRNA transcript (SSU-rRNA, 5.8S rRNA, LSU-rRNA) |
| GO:0000469 | 0.054 | 22.597 | 0 | 1 | 5 | cleavage involved in rRNA processing |
| GO:2000044 | 0.054 | 22.597 | 0 | 1 | 5 | negative regulation of cardiac cell fate specification |
| GO:0015920 | 0.054 | 22.597 | 0 | 1 | 5 | lipopolysaccharide transport |
| GO:0031146 | 0.054 | 22.597 | 0 | 1 | 5 | SCF-dependent proteasomal ubiquitin-dependent protein catabolic process |
| GO:0008205 | 0.054 | 22.597 | 0 | 1 | 5 | ecdysone metabolic process |
| GO:0003306 | 0.054 | 22.597 | 0 | 1 | 5 | Wnt receptor signaling pathway involved in heart development |
| GO:0055086 | 0.054 | 1.829 | 6 | 11 | 580 | nucleobase-containing small molecule metabolic process |
| GO:0044260 | 0.056 | 1.429 | 40 | 48 | 3617 | cellular macromolecule metabolic process |
| GO:0032868 | 0.057 | 3.617 | 1 | 3 | 79 | response to insulin stimulus |
| GO:0008406 | 0.057 | 2.901 | 1 | 4 | 131 | gonad development |
| GO:0032784 | 0.057 | 5.520 | 0 | 2 | 35 | regulation of DNA-dependent transcription, elongation |
| GO:0032501 | 0.058 | 1.424 | 36 | 44 | 3272 | multicellular organismal process |
| GO:0035023 | 0.058 | 3.569 | 1 | 3 | 80 | regulation of Rho protein signal transduction |
| GO:0034754 | 0.058 | 3.569 | 1 | 3 | 80 | cellular hormone metabolic process |
| GO:0021700 | 0.059 | 2.500 | 2 | 5 | 190 | developmental maturation |
| GO:0006357 | 0.059 | 1.920 | 5 | 9 | 449 | regulation of transcription from RNA polymerase II promoter |
| GO:0031349 | 0.060 | 5.357 | 0 | 2 | 36 | positive regulation of defense response |
| GO:0031214 | 0.060 | 5.357 | 0 | 2 | 36 | biomineral tissue development |
| GO:0006950 | 0.062 | 1.520 | 16 | 22 | 1433 | response to stress |
| GO:0043066 | 0.062 | 2.459 | 2 | 5 | 193 | negative regulation of apoptotic process |
| GO:0009117 | 0.064 | 1.828 | 6 | 10 | 525 | nucleotide metabolic process |
| GO:0006694 | 0.064 | 3.434 | 1 | 3 | 83 | steroid biosynthetic process |
| GO:0009108 | 0.064 | 3.434 | 1 | 3 | 83 | coenzyme biosynthetic process |
| GO:0021696 | 0.065 | 18.076 | 0 | 1 | 6 | cerebellar cortex morphogenesis |
| GO:0060696 | 0.065 | 18.076 | 0 | 1 | 6 | regulation of phospholipid catabolic process |
| GO:0006101 | 0.065 | 18.076 | 0 | 1 | 6 | citrate metabolic process |
| GO:0042726 | 0.065 | 18.076 | 0 | 1 | 6 | flavin-containing compound metabolic process |
| GO:0032490 | 0.065 | 18.076 | 0 | 1 | 6 | detection of molecule of bacterial origin |
| GO:0032770 | 0.065 | 18.076 | 0 | 1 | 6 | positive regulation of monooxygenase activity |
| GO:0014823 | 0.065 | 18.076 | 0 | 1 | 6 | response to activity |
| GO:0035338 | 0.065 | 18.076 | 0 | 1 | 6 | long-chain fatty-acyl-CoA biosynthetic process |
| GO:0048268 | 0.065 | 18.076 | 0 | 1 | 6 | clathrin coat assembly |
| GO:0030318 | 0.065 | 18.076 | 0 | 1 | 6 | melanocyte differentiation |
| GO:0043367 | 0.065 | 18.076 | 0 | 1 | 6 | CD4-positive, alpha-beta T cell differentiation |
| GO:0035710 | 0.065 | 18.076 | 0 | 1 | 6 | CD4-positive, alpha-beta T cell activation |
| GO:0007307 | 0.065 | 18.076 | 0 | 1 | 6 | eggshell chorion gene amplification |
| GO:0051000 | 0.065 | 18.076 | 0 | 1 | 6 | positive regulation of nitric-oxide synthase activity |
| GO:0030727 | 0.065 | 18.076 | 0 | 1 | 6 | germarium-derived female germ-line cyst formation |
| GO:0002367 | 0.065 | 18.076 | 0 | 1 | 6 | cytokine production involved in immune response |
| GO:0002260 | 0.065 | 18.076 | 0 | 1 | 6 | lymphocyte homeostasis |
| GO:0002718 | 0.065 | 18.076 | 0 | 1 | 6 | regulation of cytokine production involved in immune response |
| GO:0072350 | 0.065 | 18.076 | 0 | 1 | 6 | tricarboxylic acid metabolic process |
| GO:0002702 | 0.065 | 18.076 | 0 | 1 | 6 | positive regulation of production of molecular mediator of immune response |
| GO:0002705 | 0.065 | 18.076 | 0 | 1 | 6 | positive regulation of leukocyte mediated immunity |
| GO:0002708 | 0.065 | 18.076 | 0 | 1 | 6 | positive regulation of lymphocyte mediated immunity |
| GO:0002821 | 0.065 | 18.076 | 0 | 1 | 6 | positive regulation of adaptive immune response |
| GO:0033539 | 0.065 | 18.076 | 0 | 1 | 6 | fatty acid beta-oxidation using acyl-CoA dehydrogenase |
| GO:0010899 | 0.065 | 18.076 | 0 | 1 | 6 | regulation of phosphatidylcholine catabolic process |
| GO:0000466 | 0.065 | 18.076 | 0 | 1 | 6 | maturation of 5.8S rRNA from tricistronic rRNA transcript (SSU-rRNA, 5.8S rRNA, LSU-rRNA) |
| GO:0010660 | 0.065 | 18.076 | 0 | 1 | 6 | regulation of muscle cell apoptotic process |
| GO:0010656 | 0.065 | 18.076 | 0 | 1 | 6 | negative regulation of muscle cell apoptotic process |
| GO:0010657 | 0.065 | 18.076 | 0 | 1 | 6 | muscle cell apoptotic process |
| GO:0000966 | 0.065 | 18.076 | 0 | 1 | 6 | RNA 5'-end processing |
| GO:0060317 | 0.065 | 18.076 | 0 | 1 | 6 | cardiac epithelial to mesenchymal transition |
| GO:0060347 | 0.065 | 18.076 | 0 | 1 | 6 | heart trabecula formation |
| GO:0003229 | 0.065 | 18.076 | 0 | 1 | 6 | ventricular cardiac muscle tissue development |
| GO:0003209 | 0.065 | 18.076 | 0 | 1 | 6 | cardiac atrium morphogenesis |
| GO:0055089 | 0.065 | 18.076 | 0 | 1 | 6 | fatty acid homeostasis |
| GO:0055021 | 0.065 | 18.076 | 0 | 1 | 6 | regulation of cardiac muscle tissue growth |
| GO:0055010 | 0.065 | 18.076 | 0 | 1 | 6 | ventricular cardiac muscle tissue morphogenesis |
| GO:0055017 | 0.065 | 18.076 | 0 | 1 | 6 | cardiac muscle tissue growth |
| GO:0006085 | 0.065 | 18.076 | 0 | 1 | 6 | acetyl-CoA biosynthetic process |
| GO:0042439 | 0.066 | 5.058 | 0 | 2 | 38 | ethanolamine-containing compound metabolic process |
| GO:0006672 | 0.066 | 5.058 | 0 | 2 | 38 | ceramide metabolic process |
| GO:0048102 | 0.066 | 5.058 | 0 | 2 | 38 | autophagic cell death |
| GO:0007365 | 0.066 | 5.058 | 0 | 2 | 38 | periodic partitioning |
| GO:0001816 | 0.068 | 3.350 | 1 | 3 | 85 | cytokine production |
| GO:0010468 | 0.068 | 1.492 | 17 | 23 | 1527 | regulation of gene expression |
| GO:0043069 | 0.068 | 2.394 | 2 | 5 | 198 | negative regulation of programmed cell death |
| GO:0055114 | 0.069 | 2.182 | 3 | 6 | 261 | oxidation-reduction process |
| GO:0001764 | 0.069 | 4.921 | 0 | 2 | 39 | neuron migration |
| GO:0009743 | 0.069 | 4.921 | 0 | 2 | 39 | response to carbohydrate stimulus |
| GO:0048469 | 0.069 | 2.706 | 2 | 4 | 140 | cell maturation |
| GO:0072521 | 0.070 | 1.854 | 5 | 9 | 464 | purine-containing compound metabolic process |
| GO:0022604 | 0.070 | 2.369 | 2 | 5 | 200 | regulation of cell morphogenesis |
| GO:0040011 | 0.071 | 1.634 | 9 | 14 | 829 | locomotion |
| GO:0023051 | 0.072 | 1.604 | 10 | 15 | 907 | regulation of signaling |
| GO:0008407 | 0.072 | 4.791 | 0 | 2 | 40 | chaeta morphogenesis |
| GO:0051252 | 0.073 | 1.512 | 14 | 20 | 1298 | regulation of RNA metabolic process |
| GO:0008037 | 0.073 | 2.646 | 2 | 4 | 143 | cell recognition |
| GO:0006461 | 0.074 | 1.905 | 4 | 8 | 400 | protein complex assembly |
| GO:0003002 | 0.074 | 1.905 | 4 | 8 | 400 | regionalization |
| GO:2000677 | 0.075 | 15.061 | 0 | 1 | 7 | regulation of transcription regulatory region DNA binding |
| GO:0070922 | 0.075 | 15.061 | 0 | 1 | 7 | small RNA loading onto RISC |
| GO:0034638 | 0.075 | 15.061 | 0 | 1 | 7 | phosphatidylcholine catabolic process |
| GO:0006434 | 0.075 | 15.061 | 0 | 1 | 7 | seryl-tRNA aminoacylation |
| GO:0060914 | 0.075 | 15.061 | 0 | 1 | 7 | heart formation |
| GO:0032401 | 0.075 | 15.061 | 0 | 1 | 7 | establishment of melanosome localization |
| GO:0032402 | 0.075 | 15.061 | 0 | 1 | 7 | melanosome transport |
| GO:0048072 | 0.075 | 15.061 | 0 | 1 | 7 | compound eye pigmentation |
| GO:0001759 | 0.075 | 15.061 | 0 | 1 | 7 | organ induction |
| GO:0071353 | 0.075 | 15.061 | 0 | 1 | 7 | cellular response to interleukin-4 |
| GO:0035087 | 0.075 | 15.061 | 0 | 1 | 7 | siRNA loading onto RISC involved in RNA interference |
| GO:0032623 | 0.075 | 15.061 | 0 | 1 | 7 | interleukin-2 production |
| GO:0035382 | 0.075 | 15.061 | 0 | 1 | 7 | sterol transmembrane transport |
| GO:0035376 | 0.075 | 15.061 | 0 | 1 | 7 | sterol import |
| GO:0009595 | 0.075 | 15.061 | 0 | 1 | 7 | detection of biotic stimulus |
| GO:0048135 | 0.075 | 15.061 | 0 | 1 | 7 | female germ-line cyst formation |
| GO:0030490 | 0.075 | 15.061 | 0 | 1 | 7 | maturation of SSU-rRNA |
| GO:0061384 | 0.075 | 15.061 | 0 | 1 | 7 | heart trabecula morphogenesis |
| GO:0048730 | 0.075 | 15.061 | 0 | 1 | 7 | epidermis morphogenesis |
| GO:0046068 | 0.075 | 15.061 | 0 | 1 | 7 | cGMP metabolic process |
| GO:0043500 | 0.075 | 15.061 | 0 | 1 | 7 | muscle adaptation |
| GO:0007527 | 0.075 | 15.061 | 0 | 1 | 7 | adult somatic muscle development |
| GO:0002706 | 0.075 | 15.061 | 0 | 1 | 7 | regulation of lymphocyte mediated immunity |
| GO:0002822 | 0.075 | 15.061 | 0 | 1 | 7 | regulation of adaptive immune response based on somatic recombination of immune receptors built from immunoglobulin superfamily domains |
| GO:0051904 | 0.075 | 15.061 | 0 | 1 | 7 | pigment granule transport |
| GO:0051905 | 0.075 | 15.061 | 0 | 1 | 7 | establishment of pigment granule localization |
| GO:0033962 | 0.075 | 15.061 | 0 | 1 | 7 | cytoplasmic mRNA processing body assembly |
| GO:0044331 | 0.075 | 15.061 | 0 | 1 | 7 | cell-cell adhesion mediated by cadherin |
| GO:0060026 | 0.075 | 15.061 | 0 | 1 | 7 | convergent extension |
| GO:0016024 | 0.075 | 15.061 | 0 | 1 | 7 | CDP-diacylglycerol biosynthetic process |
| GO:0070882 | 0.075 | 15.061 | 0 | 1 | 7 | cellular cell wall organization or biogenesis |
| GO:2000826 | 0.075 | 15.061 | 0 | 1 | 7 | regulation of heart morphogenesis |
| GO:0070508 | 0.075 | 15.061 | 0 | 1 | 7 | cholesterol import |
| GO:0060420 | 0.075 | 15.061 | 0 | 1 | 7 | regulation of heart growth |
| GO:0060412 | 0.075 | 15.061 | 0 | 1 | 7 | ventricular septum morphogenesis |
| GO:0060419 | 0.075 | 15.061 | 0 | 1 | 7 | heart growth |
| GO:0030811 | 0.075 | 2.627 | 2 | 4 | 144 | regulation of nucleotide catabolic process |
| GO:0033121 | 0.075 | 2.627 | 2 | 4 | 144 | regulation of purine nucleotide catabolic process |
| GO:0016126 | 0.075 | 4.667 | 0 | 2 | 41 | sterol biosynthetic process |
| GO:0009612 | 0.075 | 4.667 | 0 | 2 | 41 | response to mechanical stimulus |
| GO:0051924 | 0.075 | 4.667 | 0 | 2 | 41 | regulation of calcium ion transport |
| GO:0002684 | 0.075 | 3.192 | 1 | 3 | 89 | positive regulation of immune system process |
| GO:0070271 | 0.077 | 1.890 | 4 | 8 | 403 | protein complex biogenesis |
| GO:0006368 | 0.078 | 4.550 | 0 | 2 | 42 | transcription elongation from RNA polymerase II promoter |
| GO:0009062 | 0.078 | 4.550 | 0 | 2 | 42 | fatty acid catabolic process |
| GO:0048066 | 0.078 | 4.550 | 0 | 2 | 42 | developmental pigmentation |
| GO:0010565 | 0.078 | 4.550 | 0 | 2 | 42 | regulation of cellular ketone metabolic process |
| GO:0048856 | 0.078 | 1.398 | 27 | 34 | 2468 | anatomical structure development |
| GO:0061458 | 0.081 | 2.261 | 2 | 5 | 209 | reproductive system development |
| GO:0048608 | 0.081 | 2.261 | 2 | 5 | 209 | reproductive structure development |
| GO:0007369 | 0.081 | 3.084 | 1 | 3 | 92 | gastrulation |
| GO:0009948 | 0.081 | 3.084 | 1 | 3 | 92 | anterior/posterior axis specification |
| GO:0042384 | 0.082 | 4.438 | 0 | 2 | 43 | cilium assembly |
| GO:0017145 | 0.082 | 4.438 | 0 | 2 | 43 | stem cell division |
| GO:0006753 | 0.082 | 1.734 | 6 | 10 | 551 | nucleoside phosphate metabolic process |
| GO:0060548 | 0.083 | 2.250 | 2 | 5 | 210 | negative regulation of cell death |
| GO:0044093 | 0.083 | 1.855 | 5 | 8 | 410 | positive regulation of molecular function |
| GO:0009953 | 0.084 | 2.517 | 2 | 4 | 150 | dorsal/ventral pattern formation |
| GO:0051240 | 0.084 | 2.517 | 2 | 4 | 150 | positive regulation of multicellular organismal process |
| GO:0045834 | 0.085 | 4.332 | 0 | 2 | 44 | positive regulation of lipid metabolic process |
| GO:0046546 | 0.085 | 4.332 | 0 | 2 | 44 | development of primary male sexual characteristics |
| GO:0016127 | 0.085 | 12.908 | 0 | 1 | 8 | sterol catabolic process |
| GO:0021680 | 0.085 | 12.908 | 0 | 1 | 8 | cerebellar Purkinje cell layer development |
| GO:0006111 | 0.085 | 12.908 | 0 | 1 | 8 | regulation of gluconeogenesis |
| GO:0006277 | 0.085 | 12.908 | 0 | 1 | 8 | DNA amplification |
| GO:0006702 | 0.085 | 12.908 | 0 | 1 | 8 | androgen biosynthetic process |
| GO:0006707 | 0.085 | 12.908 | 0 | 1 | 8 | cholesterol catabolic process |
| GO:0006670 | 0.085 | 12.908 | 0 | 1 | 8 | sphingosine metabolic process |
| GO:0001776 | 0.085 | 12.908 | 0 | 1 | 8 | leukocyte homeostasis |
| GO:0045823 | 0.085 | 12.908 | 0 | 1 | 8 | positive regulation of heart contraction |
| GO:0009820 | 0.085 | 12.908 | 0 | 1 | 8 | alkaloid metabolic process |
| GO:0048104 | 0.085 | 12.908 | 0 | 1 | 8 | establishment of body hair or bristle planar orientation |
| GO:0046085 | 0.085 | 12.908 | 0 | 1 | 8 | adenosine metabolic process |
| GO:0051353 | 0.085 | 12.908 | 0 | 1 | 8 | positive regulation of oxidoreductase activity |
| GO:0043567 | 0.085 | 12.908 | 0 | 1 | 8 | regulation of insulin-like growth factor receptor signaling pathway |
| GO:0043654 | 0.085 | 12.908 | 0 | 1 | 8 | recognition of apoptotic cell |
| GO:0051145 | 0.085 | 12.908 | 0 | 1 | 8 | smooth muscle cell differentiation |
| GO:0007506 | 0.085 | 12.908 | 0 | 1 | 8 | gonadal mesoderm development |
| GO:0046341 | 0.085 | 12.908 | 0 | 1 | 8 | CDP-diacylglycerol metabolic process |
| GO:0046520 | 0.085 | 12.908 | 0 | 1 | 8 | sphingoid biosynthetic process |
| GO:0010886 | 0.085 | 12.908 | 0 | 1 | 8 | positive regulation of cholesterol storage |
| GO:0034384 | 0.085 | 12.908 | 0 | 1 | 8 | high-density lipoprotein particle clearance |
| GO:0055024 | 0.085 | 12.908 | 0 | 1 | 8 | regulation of cardiac muscle tissue development |
| GO:0060411 | 0.085 | 12.908 | 0 | 1 | 8 | cardiac septum morphogenesis |
| GO:0019216 | 0.086 | 3.015 | 1 | 3 | 94 | regulation of lipid metabolic process |
| GO:0003006 | 0.087 | 1.713 | 6 | 10 | 557 | developmental process involved in reproduction |
| GO:0010941 | 0.088 | 1.831 | 5 | 8 | 415 | regulation of cell death |
| GO:0006576 | 0.088 | 2.982 | 1 | 3 | 95 | cellular biogenic amine metabolic process |
| GO:0007293 | 0.088 | 4.231 | 0 | 2 | 45 | germarium-derived egg chamber formation |
| GO:0051607 | 0.088 | 4.231 | 0 | 2 | 45 | defense response to virus |
| GO:0060255 | 0.090 | 1.406 | 21 | 27 | 1905 | regulation of macromolecule metabolic process |
| GO:0042110 | 0.092 | 4.134 | 1 | 2 | 46 | T cell activation |
| GO:0045088 | 0.092 | 4.134 | 1 | 2 | 46 | regulation of innate immune response |
| GO:0050920 | 0.092 | 4.134 | 1 | 2 | 46 | regulation of chemotaxis |
| GO:0048645 | 0.092 | 4.134 | 1 | 2 | 46 | organ formation |
| GO:0010817 | 0.092 | 2.432 | 2 | 4 | 155 | regulation of hormone levels |
| GO:0007040 | 0.095 | 4.042 | 1 | 2 | 47 | lysosome organization |
| GO:0043279 | 0.095 | 4.042 | 1 | 2 | 47 | response to alkaloid |
| GO:0007298 | 0.095 | 4.042 | 1 | 2 | 47 | border follicle cell migration |
| GO:0060260 | 0.095 | 11.293 | 0 | 1 | 9 | regulation of transcription initiation from RNA polymerase II promoter |
| GO:0042304 | 0.095 | 11.293 | 0 | 1 | 9 | regulation of fatty acid biosynthetic process |
| GO:0042428 | 0.095 | 11.293 | 0 | 1 | 9 | serotonin metabolic process |
| GO:0021587 | 0.095 | 11.293 | 0 | 1 | 9 | cerebellum morphogenesis |
| GO:0021575 | 0.095 | 11.293 | 0 | 1 | 9 | hindbrain morphogenesis |
| GO:0014073 | 0.095 | 11.293 | 0 | 1 | 9 | response to tropane |
| GO:0042102 | 0.095 | 11.293 | 0 | 1 | 9 | positive regulation of T cell proliferation |
| GO:0042220 | 0.095 | 11.293 | 0 | 1 | 9 | response to cocaine |
| GO:0006777 | 0.095 | 11.293 | 0 | 1 | 9 | Mo-molybdopterin cofactor biosynthetic process |
| GO:0032324 | 0.095 | 11.293 | 0 | 1 | 9 | molybdopterin cofactor biosynthetic process |
| GO:0032400 | 0.095 | 11.293 | 0 | 1 | 9 | melanosome localization |
| GO:0019720 | 0.095 | 11.293 | 0 | 1 | 9 | Mo-molybdopterin cofactor metabolic process |
| GO:0042987 | 0.095 | 11.293 | 0 | 1 | 9 | amyloid precursor protein catabolic process |
| GO:0045229 | 0.095 | 11.293 | 0 | 1 | 9 | external encapsulating structure organization |
| GO:0006910 | 0.095 | 11.293 | 0 | 1 | 9 | phagocytosis, recognition |
| GO:0050931 | 0.095 | 11.293 | 0 | 1 | 9 | pigment cell differentiation |
| GO:0035336 | 0.095 | 11.293 | 0 | 1 | 9 | long-chain fatty-acyl-CoA metabolic process |
| GO:0030422 | 0.095 | 11.293 | 0 | 1 | 9 | production of siRNA involved in RNA interference |
| GO:0007392 | 0.095 | 11.293 | 0 | 1 | 9 | initiation of dorsal closure |
| GO:0030212 | 0.095 | 11.293 | 0 | 1 | 9 | hyaluronan metabolic process |
| GO:0043545 | 0.095 | 11.293 | 0 | 1 | 9 | molybdopterin cofactor metabolic process |
| GO:0051189 | 0.095 | 11.293 | 0 | 1 | 9 | prosthetic group metabolic process |
| GO:0043628 | 0.095 | 11.293 | 0 | 1 | 9 | ncRNA 3'-end processing |
| GO:0007638 | 0.095 | 11.293 | 0 | 1 | 9 | mechanosensory behavior |
| GO:0051875 | 0.095 | 11.293 | 0 | 1 | 9 | pigment granule localization |
| GO:0002819 | 0.095 | 11.293 | 0 | 1 | 9 | regulation of adaptive immune response |
| GO:0031054 | 0.095 | 11.293 | 0 | 1 | 9 | pre-miRNA processing |
| GO:0010884 | 0.095 | 11.293 | 0 | 1 | 9 | positive regulation of lipid storage |
| GO:0031128 | 0.095 | 11.293 | 0 | 1 | 9 | developmental induction |
| GO:0003208 | 0.095 | 11.293 | 0 | 1 | 9 | cardiac ventricle morphogenesis |
| GO:0070670 | 0.095 | 11.293 | 0 | 1 | 9 | response to interleukin-4 |
| GO:0006351 | 0.097 | 1.438 | 16 | 21 | 1426 | transcription, DNA-dependent |
| GO:0008219 | 0.098 | 1.594 | 8 | 12 | 720 | cell death |
| GO:0009966 | 0.099 | 1.564 | 9 | 13 | 797 | regulation of signal transduction |
| GO:0045165 | 0.099 | 1.967 | 3 | 6 | 288 | cell fate commitment |
| GO:0032774 | 0.099 | 1.433 | 16 | 21 | 1430 | RNA biosynthetic process |
| GO:0016265 | 0.100 | 1.589 | 8 | 12 | 722 | death |
| GO:0071822 | 0.101 | 1.767 | 5 | 8 | 429 | protein complex subunit organization |
| GO:0006986 | 0.102 | 3.869 | 1 | 2 | 49 | response to unfolded protein |
| GO:0048675 | 0.102 | 3.869 | 1 | 2 | 49 | axon extension |
| GO:0043583 | 0.102 | 3.869 | 1 | 2 | 49 | ear development |
| GO:0032502 | 0.103 | 1.341 | 32 | 38 | 2873 | developmental process |
| GO:0045664 | 0.104 | 2.322 | 2 | 4 | 162 | regulation of neuron differentiation |
| GO:0046578 | 0.104 | 2.322 | 2 | 4 | 162 | regulation of Ras protein signal transduction |
| GO:0006509 | 0.105 | 10.037 | 0 | 1 | 10 | membrane protein ectodomain proteolysis |
| GO:0048060 | 0.105 | 10.037 | 0 | 1 | 10 | negative gravitaxis |
| GO:0045176 | 0.105 | 10.037 | 0 | 1 | 10 | apical protein localization |
| GO:0032862 | 0.105 | 10.037 | 0 | 1 | 10 | activation of Rho GTPase activity |
| GO:0030316 | 0.105 | 10.037 | 0 | 1 | 10 | osteoclast differentiation |
| GO:0017144 | 0.105 | 10.037 | 0 | 1 | 10 | drug metabolic process |
| GO:0030520 | 0.105 | 10.037 | 0 | 1 | 10 | intracellular estrogen receptor signaling pathway |
| GO:0046487 | 0.105 | 10.037 | 0 | 1 | 10 | glyoxylate metabolic process |
| GO:0046519 | 0.105 | 10.037 | 0 | 1 | 10 | sphingoid metabolic process |
| GO:0015936 | 0.105 | 10.037 | 0 | 1 | 10 | coenzyme A metabolic process |
| GO:0070328 | 0.105 | 10.037 | 0 | 1 | 10 | triglyceride homeostasis |
| GO:2000142 | 0.105 | 10.037 | 0 | 1 | 10 | regulation of DNA-dependent transcription, initiation |
| GO:0003281 | 0.105 | 10.037 | 0 | 1 | 10 | ventricular septum development |
| GO:0060343 | 0.105 | 10.037 | 0 | 1 | 10 | trabecula formation |
| GO:0070536 | 0.105 | 10.037 | 0 | 1 | 10 | protein K63-linked deubiquitination |
| GO:0006112 | 0.105 | 3.788 | 1 | 2 | 50 | energy reserve metabolic process |
| GO:0032320 | 0.105 | 3.788 | 1 | 2 | 50 | positive regulation of Ras GTPase activity |
| GO:0007309 | 0.105 | 2.741 | 1 | 3 | 103 | oocyte axis specification |
| GO:0043547 | 0.105 | 2.741 | 1 | 3 | 103 | positive regulation of GTPase activity |
| GO:0016579 | 0.109 | 3.710 | 1 | 2 | 51 | protein deubiquitination |
| GO:0050821 | 0.109 | 3.710 | 1 | 2 | 51 | protein stabilization |
| GO:0072329 | 0.109 | 3.710 | 1 | 2 | 51 | monocarboxylic acid catabolic process |
| GO:0035295 | 0.109 | 2.054 | 3 | 5 | 229 | tube development |
| GO:0006091 | 0.109 | 2.054 | 3 | 5 | 229 | generation of precursor metabolites and energy |
| GO:0044106 | 0.110 | 2.686 | 1 | 3 | 105 | cellular amine metabolic process |
| GO:0007308 | 0.112 | 2.660 | 1 | 3 | 106 | oocyte construction |
| GO:0008544 | 0.112 | 2.660 | 1 | 3 | 106 | epidermis development |
| GO:0006354 | 0.113 | 3.636 | 1 | 2 | 52 | DNA-dependent transcription, elongation |
| GO:0043281 | 0.113 | 3.636 | 1 | 2 | 52 | regulation of cysteine-type endopeptidase activity involved in apoptotic process |
| GO:0006140 | 0.113 | 2.250 | 2 | 4 | 167 | regulation of nucleotide metabolic process |
| GO:1900542 | 0.113 | 2.250 | 2 | 4 | 167 | regulation of purine nucleotide metabolic process |
| GO:0012501 | 0.115 | 1.574 | 7 | 11 | 665 | programmed cell death |
| GO:0042332 | 0.115 | 9.032 | 0 | 1 | 11 | gravitaxis |
| GO:0021695 | 0.115 | 9.032 | 0 | 1 | 11 | cerebellar cortex development |
| GO:0032088 | 0.115 | 9.032 | 0 | 1 | 11 | negative regulation of NF-kappaB transcription factor activity |
| GO:0006654 | 0.115 | 9.032 | 0 | 1 | 11 | phosphatidic acid biosynthetic process |
| GO:0050671 | 0.115 | 9.032 | 0 | 1 | 11 | positive regulation of lymphocyte proliferation |
| GO:0014896 | 0.115 | 9.032 | 0 | 1 | 11 | muscle hypertrophy |
| GO:0014897 | 0.115 | 9.032 | 0 | 1 | 11 | striated muscle hypertrophy |
| GO:0019695 | 0.115 | 9.032 | 0 | 1 | 11 | choline metabolic process |
| GO:0050999 | 0.115 | 9.032 | 0 | 1 | 11 | regulation of nitric-oxide synthase activity |
| GO:0009826 | 0.115 | 9.032 | 0 | 1 | 11 | unidimensional cell growth |
| GO:0061383 | 0.115 | 9.032 | 0 | 1 | 11 | trabecula morphogenesis |
| GO:0071554 | 0.115 | 9.032 | 0 | 1 | 11 | cell wall organization or biogenesis |
| GO:0032946 | 0.115 | 9.032 | 0 | 1 | 11 | positive regulation of mononuclear cell proliferation |
| GO:0007414 | 0.115 | 9.032 | 0 | 1 | 11 | axonal defasciculation |
| GO:0007280 | 0.115 | 9.032 | 0 | 1 | 11 | pole cell migration |
| GO:0030501 | 0.115 | 9.032 | 0 | 1 | 11 | positive regulation of bone mineralization |
| GO:0046320 | 0.115 | 9.032 | 0 | 1 | 11 | regulation of fatty acid oxidation |
| GO:0046473 | 0.115 | 9.032 | 0 | 1 | 11 | phosphatidic acid metabolic process |
| GO:0051825 | 0.115 | 9.032 | 0 | 1 | 11 | adhesion to other organism involved in symbiotic interaction |
| GO:0046632 | 0.115 | 9.032 | 0 | 1 | 11 | alpha-beta T cell differentiation |
| GO:0010885 | 0.115 | 9.032 | 0 | 1 | 11 | regulation of cholesterol storage |
| GO:0000460 | 0.115 | 9.032 | 0 | 1 | 11 | maturation of 5.8S rRNA |
| GO:0051923 | 0.115 | 9.032 | 0 | 1 | 11 | sulfation |
| GO:0015969 | 0.115 | 9.032 | 0 | 1 | 11 | guanosine tetraphosphate metabolic process |
| GO:0003300 | 0.115 | 9.032 | 0 | 1 | 11 | cardiac muscle hypertrophy |
| GO:0003156 | 0.115 | 9.032 | 0 | 1 | 11 | regulation of organ formation |
| GO:0031639 | 0.115 | 9.032 | 0 | 1 | 11 | plasminogen activation |
| GO:0070665 | 0.115 | 9.032 | 0 | 1 | 11 | positive regulation of leukocyte proliferation |
| GO:0019395 | 0.116 | 3.564 | 1 | 2 | 53 | fatty acid oxidation |
| GO:0009306 | 0.116 | 3.564 | 1 | 2 | 53 | protein secretion |
| GO:0007297 | 0.116 | 3.564 | 1 | 2 | 53 | ovarian follicle cell migration |
| GO:0035966 | 0.116 | 3.564 | 1 | 2 | 53 | response to topologically incorrect protein |
| GO:0010035 | 0.117 | 2.222 | 2 | 4 | 169 | response to inorganic substance |
| GO:0007507 | 0.118 | 1.999 | 3 | 5 | 235 | heart development |
| GO:0051188 | 0.120 | 2.584 | 1 | 3 | 109 | cofactor biosynthetic process |
| GO:0007310 | 0.120 | 3.495 | 1 | 2 | 54 | oocyte dorsal/ventral axis specification |
| GO:0030203 | 0.120 | 3.495 | 1 | 2 | 54 | glycosaminoglycan metabolic process |
| GO:0046661 | 0.120 | 3.495 | 1 | 2 | 54 | male sex differentiation |
| GO:0048646 | 0.121 | 1.635 | 6 | 9 | 521 | anatomical structure formation involved in morphogenesis |
| GO:0043434 | 0.122 | 2.559 | 1 | 3 | 110 | response to peptide hormone stimulus |
| GO:0030855 | 0.122 | 2.559 | 1 | 3 | 110 | epithelial cell differentiation |
| GO:0031347 | 0.122 | 2.559 | 1 | 3 | 110 | regulation of defense response |
| GO:0009719 | 0.122 | 1.844 | 3 | 6 | 306 | response to endogenous stimulus |
| GO:0050770 | 0.123 | 3.428 | 1 | 2 | 55 | regulation of axonogenesis |
| GO:0002253 | 0.123 | 3.428 | 1 | 2 | 55 | activation of immune response |
| GO:0055088 | 0.123 | 3.428 | 1 | 2 | 55 | lipid homeostasis |
| GO:0007417 | 0.124 | 1.745 | 4 | 7 | 378 | central nervous system development |
| GO:0060627 | 0.124 | 2.535 | 1 | 3 | 111 | regulation of vesicle-mediated transport |
| GO:0031329 | 0.125 | 1.964 | 3 | 5 | 239 | regulation of cellular catabolic process |
| GO:0042129 | 0.125 | 8.210 | 0 | 1 | 12 | regulation of T cell proliferation |
| GO:0042274 | 0.125 | 8.210 | 0 | 1 | 12 | ribosomal small subunit biogenesis |
| GO:0050774 | 0.125 | 8.210 | 0 | 1 | 12 | negative regulation of dendrite morphogenesis |
| GO:0016925 | 0.125 | 8.210 | 0 | 1 | 12 | protein sumoylation |
| GO:0006555 | 0.125 | 8.210 | 0 | 1 | 12 | methionine metabolic process |
| GO:0045455 | 0.125 | 8.210 | 0 | 1 | 12 | ecdysteroid metabolic process |
| GO:0048013 | 0.125 | 8.210 | 0 | 1 | 12 | ephrin receptor signaling pathway |
| GO:0032768 | 0.125 | 8.210 | 0 | 1 | 12 | regulation of monooxygenase activity |
| GO:0032781 | 0.125 | 8.210 | 0 | 1 | 12 | positive regulation of ATPase activity |
| GO:0032874 | 0.125 | 8.210 | 0 | 1 | 12 | positive regulation of stress-activated MAPK cascade |
| GO:0050982 | 0.125 | 8.210 | 0 | 1 | 12 | detection of mechanical stimulus |
| GO:0035384 | 0.125 | 8.210 | 0 | 1 | 12 | thioester biosynthetic process |
| GO:0035196 | 0.125 | 8.210 | 0 | 1 | 12 | production of miRNAs involved in gene silencing by miRNA |
| GO:0035146 | 0.125 | 8.210 | 0 | 1 | 12 | tube fusion |
| GO:0035147 | 0.125 | 8.210 | 0 | 1 | 12 | branch fusion, open tracheal system |
| GO:0071695 | 0.125 | 8.210 | 0 | 1 | 12 | anatomical structure maturation |
| GO:0071616 | 0.125 | 8.210 | 0 | 1 | 12 | acyl-CoA biosynthetic process |
| GO:0043154 | 0.125 | 8.210 | 0 | 1 | 12 | negative regulation of cysteine-type endopeptidase activity involved in apoptotic process |
| GO:0007271 | 0.125 | 8.210 | 0 | 1 | 12 | synaptic transmission, cholinergic |
| GO:0035909 | 0.125 | 8.210 | 0 | 1 | 12 | aorta morphogenesis |
| GO:0046330 | 0.125 | 8.210 | 0 | 1 | 12 | positive regulation of JNK cascade |
| GO:0033619 | 0.125 | 8.210 | 0 | 1 | 12 | membrane protein proteolysis |
| GO:0046949 | 0.125 | 8.210 | 0 | 1 | 12 | fatty-acyl-CoA biosynthetic process |
| GO:0070304 | 0.125 | 8.210 | 0 | 1 | 12 | positive regulation of stress-activated protein kinase signaling cascade |
| GO:0070169 | 0.125 | 8.210 | 0 | 1 | 12 | positive regulation of biomineral tissue development |
| GO:0034383 | 0.125 | 8.210 | 0 | 1 | 12 | low-density lipoprotein particle clearance |
| GO:0055013 | 0.125 | 8.210 | 0 | 1 | 12 | cardiac muscle cell development |
| GO:0060541 | 0.126 | 1.955 | 3 | 5 | 240 | respiratory system development |
| GO:0006909 | 0.127 | 2.511 | 1 | 3 | 112 | phagocytosis |
| GO:0006816 | 0.127 | 3.365 | 1 | 2 | 56 | calcium ion transport |
| GO:0002521 | 0.127 | 3.365 | 1 | 2 | 56 | leukocyte differentiation |
| GO:0006366 | 0.127 | 1.615 | 6 | 9 | 527 | transcription from RNA polymerase II promoter |
| GO:0006725 | 0.128 | 2.142 | 2 | 4 | 175 | cellular aromatic compound metabolic process |
| GO:0042981 | 0.129 | 1.725 | 4 | 7 | 382 | regulation of apoptotic process |
| GO:0009100 | 0.130 | 2.130 | 2 | 4 | 176 | glycoprotein metabolic process |
| GO:2000116 | 0.131 | 3.303 | 1 | 2 | 57 | regulation of cysteine-type endopeptidase activity |
| GO:0070646 | 0.131 | 3.303 | 1 | 2 | 57 | protein modification by small protein removal |
| GO:0007420 | 0.133 | 1.922 | 3 | 5 | 244 | brain development |
| GO:0042445 | 0.134 | 2.443 | 1 | 3 | 115 | hormone metabolic process |
| GO:0030099 | 0.135 | 3.244 | 1 | 2 | 58 | myeloid cell differentiation |
| GO:0030100 | 0.135 | 3.244 | 1 | 2 | 58 | regulation of endocytosis |
| GO:0022618 | 0.135 | 3.244 | 1 | 2 | 58 | ribonucleoprotein complex assembly |
| GO:0008356 | 0.135 | 3.244 | 1 | 2 | 58 | asymmetric cell division |
| GO:0010959 | 0.135 | 3.244 | 1 | 2 | 58 | regulation of metal ion transport |
| GO:0042360 | 0.135 | 7.525 | 0 | 1 | 13 | vitamin E metabolic process |
| GO:0042775 | 0.135 | 7.525 | 0 | 1 | 13 | mitochondrial ATP synthesis coupled electron transport |
| GO:0009083 | 0.135 | 7.525 | 0 | 1 | 13 | branched-chain amino acid catabolic process |
| GO:0006706 | 0.135 | 7.525 | 0 | 1 | 13 | steroid catabolic process |
| GO:0045778 | 0.135 | 7.525 | 0 | 1 | 13 | positive regulation of ossification |
| GO:0050924 | 0.135 | 7.525 | 0 | 1 | 13 | positive regulation of negative chemotaxis |
| GO:0050929 | 0.135 | 7.525 | 0 | 1 | 13 | induction of negative chemotaxis |
| GO:0035307 | 0.135 | 7.525 | 0 | 1 | 13 | positive regulation of protein dephosphorylation |
| GO:0051318 | 0.135 | 7.525 | 0 | 1 | 13 | G1 phase |
| GO:0007502 | 0.135 | 7.525 | 0 | 1 | 13 | digestive tract mesoderm development |
| GO:0002703 | 0.135 | 7.525 | 0 | 1 | 13 | regulation of leukocyte mediated immunity |
| GO:0090175 | 0.135 | 7.525 | 0 | 1 | 13 | regulation of establishment of planar polarity |
| GO:0010469 | 0.135 | 7.525 | 0 | 1 | 13 | regulation of receptor activity |
| GO:0000080 | 0.135 | 7.525 | 0 | 1 | 13 | G1 phase of mitotic cell cycle |
| GO:0090210 | 0.135 | 7.525 | 0 | 1 | 13 | regulation of establishment of blood-brain barrier |
| GO:0090212 | 0.135 | 7.525 | 0 | 1 | 13 | negative regulation of establishment of blood-brain barrier |
| GO:0033627 | 0.135 | 7.525 | 0 | 1 | 13 | cell adhesion mediated by integrin |
| GO:0060071 | 0.135 | 7.525 | 0 | 1 | 13 | Wnt receptor signaling pathway, planar cell polarity pathway |
| GO:0055091 | 0.135 | 7.525 | 0 | 1 | 13 | phospholipid homeostasis |
| GO:0055006 | 0.135 | 7.525 | 0 | 1 | 13 | cardiac cell development |
| GO:0022603 | 0.135 | 1.789 | 3 | 6 | 315 | regulation of anatomical structure morphogenesis |
| GO:0006508 | 0.136 | 1.468 | 9 | 13 | 843 | proteolysis |
| GO:0045944 | 0.136 | 1.905 | 3 | 5 | 246 | positive regulation of transcription from RNA polymerase II promoter |
| GO:0016477 | 0.137 | 1.697 | 4 | 7 | 388 | cell migration |
| GO:0009615 | 0.138 | 3.186 | 1 | 2 | 59 | response to virus |
| GO:0048588 | 0.138 | 3.186 | 1 | 2 | 59 | developmental cell growth |
| GO:0034440 | 0.138 | 3.186 | 1 | 2 | 59 | lipid oxidation |
| GO:0007389 | 0.140 | 1.624 | 5 | 8 | 464 | pattern specification process |
| GO:0006298 | 0.144 | 6.945 | 0 | 1 | 14 | mismatch repair |
| GO:0042773 | 0.144 | 6.945 | 0 | 1 | 14 | ATP synthesis coupled electron transport |
| GO:0006760 | 0.144 | 6.945 | 0 | 1 | 14 | folic acid-containing compound metabolic process |
| GO:0006890 | 0.144 | 6.945 | 0 | 1 | 14 | retrograde vesicle-mediated transport, Golgi to ER |
| GO:0050707 | 0.144 | 6.945 | 0 | 1 | 14 | regulation of cytokine secretion |
| GO:0060912 | 0.144 | 6.945 | 0 | 1 | 14 | cardiac cell fate specification |
| GO:0032479 | 0.144 | 6.945 | 0 | 1 | 14 | regulation of type I interferon production |
| GO:0042685 | 0.144 | 6.945 | 0 | 1 | 14 | cardioblast cell fate specification |
| GO:0042686 | 0.144 | 6.945 | 0 | 1 | 14 | regulation of cardioblast cell fate specification |
| GO:0048069 | 0.144 | 6.945 | 0 | 1 | 14 | eye pigmentation |
| GO:0042982 | 0.144 | 6.945 | 0 | 1 | 14 | amyloid precursor protein metabolic process |
| GO:0032856 | 0.144 | 6.945 | 0 | 1 | 14 | activation of Ras GTPase activity |
| GO:0043392 | 0.144 | 6.945 | 0 | 1 | 14 | negative regulation of DNA binding |
| GO:0030716 | 0.144 | 6.945 | 0 | 1 | 14 | oocyte fate determination |
| GO:0007294 | 0.144 | 6.945 | 0 | 1 | 14 | germarium-derived oocyte fate determination |
| GO:0035904 | 0.144 | 6.945 | 0 | 1 | 14 | aorta development |
| GO:0051290 | 0.144 | 6.945 | 0 | 1 | 14 | protein heterotetramerization |
| GO:0051890 | 0.144 | 6.945 | 0 | 1 | 14 | regulation of cardioblast differentiation |
| GO:0002699 | 0.144 | 6.945 | 0 | 1 | 14 | positive regulation of immune effector process |
| GO:0046631 | 0.144 | 6.945 | 0 | 1 | 14 | alpha-beta T cell activation |
| GO:0010878 | 0.144 | 6.945 | 0 | 1 | 14 | cholesterol storage |
| GO:2000043 | 0.144 | 6.945 | 0 | 1 | 14 | regulation of cardiac cell fate specification |
| GO:0042098 | 0.144 | 6.945 | 0 | 1 | 14 | T cell proliferation |
| GO:0009725 | 0.144 | 1.865 | 3 | 5 | 251 | response to hormone stimulus |
| GO:0051049 | 0.146 | 1.665 | 4 | 7 | 395 | regulation of transport |
| GO:0048599 | 0.147 | 2.337 | 1 | 3 | 120 | oocyte development |
| GO:0007166 | 0.150 | 1.420 | 10 | 14 | 938 | cell surface receptor signaling pathway |
| GO:0035303 | 0.150 | 3.026 | 1 | 2 | 62 | regulation of dephosphorylation |
| GO:0071826 | 0.150 | 3.026 | 1 | 2 | 62 | ribonucleoprotein complex subunit organization |
| GO:0035152 | 0.150 | 3.026 | 1 | 2 | 62 | regulation of tube architecture, open tracheal system |
| GO:0007266 | 0.150 | 3.026 | 1 | 2 | 62 | Rho protein signal transduction |
| GO:0007424 | 0.150 | 2.010 | 2 | 4 | 186 | open tracheal system development |
| GO:0043067 | 0.150 | 1.651 | 4 | 7 | 398 | regulation of programmed cell death |
| GO:0009888 | 0.152 | 1.454 | 9 | 12 | 782 | tissue development |
| GO:0007265 | 0.152 | 2.298 | 1 | 3 | 122 | Ras protein signal transduction |
| GO:0009064 | 0.154 | 2.976 | 1 | 2 | 63 | glutamine family amino acid metabolic process |
| GO:0035051 | 0.154 | 2.976 | 1 | 2 | 63 | cardiac cell differentiation |
| GO:0008063 | 0.154 | 2.976 | 1 | 2 | 63 | Toll signaling pathway |
| GO:0070918 | 0.154 | 6.448 | 0 | 1 | 15 | production of small RNA involved in gene silencing by RNA |
| GO:0032321 | 0.154 | 6.448 | 0 | 1 | 15 | positive regulation of Rho GTPase activity |
| GO:0050764 | 0.154 | 6.448 | 0 | 1 | 15 | regulation of phagocytosis |
| GO:0050728 | 0.154 | 6.448 | 0 | 1 | 15 | negative regulation of inflammatory response |
| GO:0045446 | 0.154 | 6.448 | 0 | 1 | 15 | endothelial cell differentiation |
| GO:0001570 | 0.154 | 6.448 | 0 | 1 | 15 | vasculogenesis |
| GO:0050923 | 0.154 | 6.448 | 0 | 1 | 15 | regulation of negative chemotaxis |
| GO:0032606 | 0.154 | 6.448 | 0 | 1 | 15 | type I interferon production |
| GO:0035337 | 0.154 | 6.448 | 0 | 1 | 15 | fatty-acyl-CoA metabolic process |
| GO:0035304 | 0.154 | 6.448 | 0 | 1 | 15 | regulation of protein dephosphorylation |
| GO:0035306 | 0.154 | 6.448 | 0 | 1 | 15 | positive regulation of dephosphorylation |
| GO:0048260 | 0.154 | 6.448 | 0 | 1 | 15 | positive regulation of receptor-mediated endocytosis |
| GO:0043462 | 0.154 | 6.448 | 0 | 1 | 15 | regulation of ATPase activity |
| GO:0048676 | 0.154 | 6.448 | 0 | 1 | 15 | axon extension involved in development |
| GO:0035567 | 0.154 | 6.448 | 0 | 1 | 15 | non-canonical Wnt receptor signaling pathway |
| GO:0051341 | 0.154 | 6.448 | 0 | 1 | 15 | regulation of oxidoreductase activity |
| GO:0007595 | 0.154 | 6.448 | 0 | 1 | 15 | lactation |
| GO:0043507 | 0.154 | 6.448 | 0 | 1 | 15 | positive regulation of JUN kinase activity |
| GO:0008078 | 0.154 | 6.448 | 0 | 1 | 15 | mesodermal cell migration |
| GO:0031050 | 0.154 | 6.448 | 0 | 1 | 15 | dsRNA fragmentation |
| GO:2000274 | 0.154 | 6.448 | 0 | 1 | 15 | regulation of epithelial cell migration, open tracheal system |
| GO:0003158 | 0.154 | 6.448 | 0 | 1 | 15 | endothelium development |
| GO:0016476 | 0.154 | 6.448 | 0 | 1 | 15 | regulation of embryonic cell shape |
| GO:0055008 | 0.154 | 6.448 | 0 | 1 | 15 | cardiac muscle tissue morphogenesis |
| GO:0032101 | 0.155 | 2.278 | 1 | 3 | 123 | regulation of response to external stimulus |
| GO:0060271 | 0.157 | 2.928 | 1 | 2 | 64 | cilium morphogenesis |
| GO:0023057 | 0.158 | 1.703 | 4 | 6 | 330 | negative regulation of signaling |
| GO:0051056 | 0.159 | 1.966 | 2 | 4 | 190 | regulation of small GTPase mediated signal transduction |
| GO:1901068 | 0.159 | 1.966 | 2 | 4 | 190 | guanosine-containing compound metabolic process |
| GO:0050778 | 0.161 | 2.881 | 1 | 2 | 65 | positive regulation of immune response |
| GO:0042475 | 0.163 | 6.018 | 0 | 1 | 16 | odontogenesis of dentin-containing tooth |
| GO:0050848 | 0.163 | 6.018 | 0 | 1 | 16 | regulation of calcium-mediated signaling |
| GO:0048070 | 0.163 | 6.018 | 0 | 1 | 16 | regulation of developmental pigmentation |
| GO:0050670 | 0.163 | 6.018 | 0 | 1 | 16 | regulation of lymphocyte proliferation |
| GO:0048009 | 0.163 | 6.018 | 0 | 1 | 16 | insulin-like growth factor receptor signaling pathway |
| GO:0071359 | 0.163 | 6.018 | 0 | 1 | 16 | cellular response to dsRNA |
| GO:0001676 | 0.163 | 6.018 | 0 | 1 | 16 | long-chain fatty acid metabolic process |
| GO:0048138 | 0.163 | 6.018 | 0 | 1 | 16 | germ-line cyst encapsulation |
| GO:0048139 | 0.163 | 6.018 | 0 | 1 | 16 | female germ-line cyst encapsulation |
| GO:0009629 | 0.163 | 6.018 | 0 | 1 | 16 | response to gravity |
| GO:0032944 | 0.163 | 6.018 | 0 | 1 | 16 | regulation of mononuclear cell proliferation |
| GO:0007412 | 0.163 | 6.018 | 0 | 1 | 16 | axon target recognition |
| GO:0030720 | 0.163 | 6.018 | 0 | 1 | 16 | oocyte localization involved in germarium-derived egg chamber formation |
| GO:0030706 | 0.163 | 6.018 | 0 | 1 | 16 | germarium-derived oocyte differentiation |
| GO:0030708 | 0.163 | 6.018 | 0 | 1 | 16 | germarium-derived female germ-line cyst encapsulation |
| GO:0043534 | 0.163 | 6.018 | 0 | 1 | 16 | blood vessel endothelial cell migration |
| GO:0043666 | 0.163 | 6.018 | 0 | 1 | 16 | regulation of phosphoprotein phosphatase activity |
| GO:0090181 | 0.163 | 6.018 | 0 | 1 | 16 | regulation of cholesterol metabolic process |
| GO:1901160 | 0.163 | 6.018 | 0 | 1 | 16 | primary amino compound metabolic process |
| GO:0008594 | 0.163 | 6.018 | 0 | 1 | 16 | photoreceptor cell morphogenesis |
| GO:0008584 | 0.163 | 6.018 | 0 | 1 | 16 | male gonad development |
| GO:0060415 | 0.163 | 6.018 | 0 | 1 | 16 | muscle tissue morphogenesis |
| GO:0019827 | 0.165 | 2.835 | 1 | 2 | 66 | stem cell maintenance |
| GO:0050954 | 0.165 | 2.835 | 1 | 2 | 66 | sensory perception of mechanical stimulus |
| GO:0007033 | 0.165 | 2.835 | 1 | 2 | 66 | vacuole organization |
| GO:0048871 | 0.165 | 2.835 | 1 | 2 | 66 | multicellular organismal homeostasis |
| GO:0043473 | 0.165 | 2.835 | 1 | 2 | 66 | pigmentation |
| GO:0070838 | 0.165 | 2.835 | 1 | 2 | 66 | divalent metal ion transport |
| GO:0001700 | 0.166 | 2.204 | 1 | 3 | 127 | embryonic development via the syncytial blastoderm |
| GO:0043087 | 0.168 | 2.186 | 1 | 3 | 128 | regulation of GTPase activity |
| GO:0010648 | 0.169 | 1.665 | 4 | 6 | 337 | negative regulation of cell communication |
| GO:0052548 | 0.169 | 2.791 | 1 | 2 | 67 | regulation of endopeptidase activity |
| GO:0006979 | 0.171 | 2.168 | 1 | 3 | 129 | response to oxidative stress |
| GO:0033124 | 0.171 | 2.168 | 1 | 3 | 129 | regulation of GTP catabolic process |
| GO:0006400 | 0.172 | 5.641 | 0 | 1 | 17 | tRNA modification |
| GO:0032069 | 0.172 | 5.641 | 0 | 1 | 17 | regulation of nuclease activity |
| GO:0032075 | 0.172 | 5.641 | 0 | 1 | 17 | positive regulation of nuclease activity |
| GO:0050885 | 0.172 | 5.641 | 0 | 1 | 17 | neuromuscular process controlling balance |
| GO:0019853 | 0.172 | 5.641 | 0 | 1 | 17 | L-ascorbic acid biosynthetic process |
| GO:0032231 | 0.172 | 5.641 | 0 | 1 | 17 | regulation of actin filament bundle assembly |
| GO:0045186 | 0.172 | 5.641 | 0 | 1 | 17 | zonula adherens assembly |
| GO:0071356 | 0.172 | 5.641 | 0 | 1 | 17 | cellular response to tumor necrosis factor |
| GO:0071322 | 0.172 | 5.641 | 0 | 1 | 17 | cellular response to carbohydrate stimulus |
| GO:0006987 | 0.172 | 5.641 | 0 | 1 | 17 | activation of signaling protein activity involved in unfolded protein response |
| GO:0048259 | 0.172 | 5.641 | 0 | 1 | 17 | regulation of receptor-mediated endocytosis |
| GO:0043331 | 0.172 | 5.641 | 0 | 1 | 17 | response to dsRNA |
| GO:0048644 | 0.172 | 5.641 | 0 | 1 | 17 | muscle organ morphogenesis |
| GO:0030866 | 0.172 | 5.641 | 0 | 1 | 17 | cortical actin cytoskeleton organization |
| GO:0000038 | 0.172 | 5.641 | 0 | 1 | 17 | very long-chain fatty acid metabolic process |
| GO:0008258 | 0.172 | 5.641 | 0 | 1 | 17 | head involution |
| GO:2000117 | 0.172 | 5.641 | 0 | 1 | 17 | negative regulation of cysteine-type endopeptidase activity |
| GO:0003283 | 0.172 | 5.641 | 0 | 1 | 17 | atrial septum development |
| GO:0003231 | 0.172 | 5.641 | 0 | 1 | 17 | cardiac ventricle development |
| GO:0070663 | 0.172 | 5.641 | 0 | 1 | 17 | regulation of leukocyte proliferation |
| GO:0009636 | 0.173 | 2.749 | 1 | 2 | 68 | response to toxin |
| GO:0072511 | 0.173 | 2.749 | 1 | 2 | 68 | divalent inorganic cation transport |
| GO:0050767 | 0.173 | 1.893 | 2 | 4 | 197 | regulation of neurogenesis |
| GO:0007350 | 0.174 | 2.151 | 1 | 3 | 130 | blastoderm segmentation |
| GO:0051707 | 0.175 | 1.741 | 3 | 5 | 268 | response to other organism |
| GO:0034645 | 0.175 | 1.271 | 24 | 28 | 2137 | cellular macromolecule biosynthetic process |
| GO:0052547 | 0.177 | 2.707 | 1 | 2 | 69 | regulation of peptidase activity |
| GO:0032869 | 0.177 | 2.707 | 1 | 2 | 69 | cellular response to insulin stimulus |
| GO:0048864 | 0.177 | 2.707 | 1 | 2 | 69 | stem cell development |
| GO:0007267 | 0.177 | 1.521 | 5 | 8 | 493 | cell-cell signaling |
| GO:0016043 | 0.178 | 1.254 | 28 | 33 | 2573 | cellular component organization |
| GO:0009894 | 0.178 | 1.728 | 3 | 5 | 270 | regulation of catabolic process |
| GO:0030707 | 0.179 | 2.117 | 1 | 3 | 132 | ovarian follicle cell development |
| GO:0031647 | 0.181 | 2.667 | 1 | 2 | 70 | regulation of protein stability |
| GO:0001818 | 0.182 | 5.308 | 0 | 1 | 18 | negative regulation of cytokine production |
| GO:0050994 | 0.182 | 5.308 | 0 | 1 | 18 | regulation of lipid catabolic process |
| GO:0035385 | 0.182 | 5.308 | 0 | 1 | 18 | Roundabout signaling pathway |
| GO:0009593 | 0.182 | 5.308 | 0 | 1 | 18 | detection of chemical stimulus |
| GO:0048134 | 0.182 | 5.308 | 0 | 1 | 18 | germ-line cyst formation |
| GO:0048841 | 0.182 | 5.308 | 0 | 1 | 18 | regulation of axon extension involved in axon guidance |
| GO:0007189 | 0.182 | 5.308 | 0 | 1 | 18 | adenylate cyclase-activating G-protein coupled receptor signaling pathway |
| GO:0030865 | 0.182 | 5.308 | 0 | 1 | 18 | cortical cytoskeleton organization |
| GO:0043588 | 0.182 | 5.308 | 0 | 1 | 18 | skin development |
| GO:0033227 | 0.182 | 5.308 | 0 | 1 | 18 | dsRNA transport |
| GO:0008038 | 0.182 | 2.100 | 1 | 3 | 133 | neuron recognition |
| GO:0009607 | 0.182 | 1.714 | 3 | 5 | 272 | response to biotic stimulus |
| GO:0044092 | 0.184 | 1.844 | 2 | 4 | 202 | negative regulation of molecular function |
| GO:0042493 | 0.185 | 2.084 | 1 | 3 | 134 | response to drug |
| GO:0001701 | 0.185 | 2.084 | 1 | 3 | 134 | in utero embryonic development |
| GO:0022416 | 0.185 | 2.628 | 1 | 2 | 71 | chaeta development |
| GO:0032879 | 0.186 | 1.463 | 6 | 9 | 577 | regulation of localization |
| GO:0046395 | 0.188 | 2.068 | 1 | 3 | 135 | carboxylic acid catabolic process |
| GO:0016054 | 0.188 | 2.068 | 1 | 3 | 135 | organic acid catabolic process |
| GO:0060284 | 0.188 | 1.694 | 3 | 5 | 275 | regulation of cell development |
| GO:0006163 | 0.188 | 1.543 | 5 | 7 | 424 | purine nucleotide metabolic process |
| GO:0051174 | 0.188 | 1.604 | 4 | 6 | 349 | regulation of phosphorus metabolic process |
| GO:0009059 | 0.189 | 1.255 | 24 | 28 | 2156 | macromolecule biosynthetic process |
| GO:0050795 | 0.189 | 2.590 | 1 | 2 | 72 | regulation of behavior |
| GO:0043269 | 0.189 | 2.590 | 1 | 2 | 72 | regulation of ion transport |
| GO:0042592 | 0.190 | 1.492 | 6 | 8 | 502 | homeostatic process |
| GO:0032535 | 0.190 | 2.052 | 2 | 3 | 136 | regulation of cellular component size |
| GO:0021549 | 0.191 | 5.013 | 0 | 1 | 19 | cerebellum development |
| GO:0016578 | 0.191 | 5.013 | 0 | 1 | 19 | histone deubiquitination |
| GO:0006730 | 0.191 | 5.013 | 0 | 1 | 19 | one-carbon metabolic process |
| GO:0042684 | 0.191 | 5.013 | 0 | 1 | 19 | cardioblast cell fate commitment |
| GO:0019852 | 0.191 | 5.013 | 0 | 1 | 19 | L-ascorbic acid metabolic process |
| GO:0006919 | 0.191 | 5.013 | 0 | 1 | 19 | activation of cysteine-type endopeptidase activity involved in apoptotic process |
| GO:0022405 | 0.191 | 5.013 | 0 | 1 | 19 | hair cycle process |
| GO:0097202 | 0.191 | 5.013 | 0 | 1 | 19 | activation of cysteine-type endopeptidase activity |
| GO:0035160 | 0.191 | 5.013 | 0 | 1 | 19 | maintenance of epithelial integrity, open tracheal system |
| GO:0001942 | 0.191 | 5.013 | 0 | 1 | 19 | hair follicle development |
| GO:0035725 | 0.191 | 5.013 | 0 | 1 | 19 | sodium ion transmembrane transport |
| GO:0051084 | 0.191 | 5.013 | 0 | 1 | 19 | 'de novo' posttranslational protein folding |
| GO:0007509 | 0.191 | 5.013 | 0 | 1 | 19 | mesoderm migration involved in gastrulation |
| GO:0046329 | 0.191 | 5.013 | 0 | 1 | 19 | negative regulation of JNK cascade |
| GO:0002224 | 0.191 | 5.013 | 0 | 1 | 19 | toll-like receptor signaling pathway |
| GO:0043968 | 0.191 | 5.013 | 0 | 1 | 19 | histone H2A acetylation |
| GO:0002573 | 0.191 | 5.013 | 0 | 1 | 19 | myeloid leukocyte differentiation |
| GO:0015914 | 0.191 | 5.013 | 0 | 1 | 19 | phospholipid transport |
| GO:0034381 | 0.191 | 5.013 | 0 | 1 | 19 | plasma lipoprotein particle clearance |
| GO:0034375 | 0.191 | 5.013 | 0 | 1 | 19 | high-density lipoprotein particle remodeling |
| GO:0003407 | 0.191 | 5.013 | 0 | 1 | 19 | neural retina development |
| GO:0003206 | 0.191 | 5.013 | 0 | 1 | 19 | cardiac chamber morphogenesis |
| GO:0055007 | 0.191 | 5.013 | 0 | 1 | 19 | cardiac muscle cell differentiation |
| GO:0006911 | 0.193 | 2.554 | 1 | 2 | 73 | phagocytosis, engulfment |
| GO:0046649 | 0.193 | 2.554 | 1 | 2 | 73 | lymphocyte activation |
| GO:0072358 | 0.194 | 1.590 | 4 | 6 | 352 | cardiovascular system development |
| GO:0072359 | 0.194 | 1.590 | 4 | 6 | 352 | circulatory system development |
| GO:0046903 | 0.195 | 1.585 | 4 | 6 | 353 | secretion |
| GO:0090066 | 0.196 | 1.797 | 2 | 4 | 207 | regulation of anatomical structure size |
| GO:0042326 | 0.197 | 2.518 | 1 | 2 | 74 | negative regulation of phosphorylation |
| GO:0016573 | 0.197 | 2.518 | 1 | 2 | 74 | histone acetylation |
| GO:0007423 | 0.197 | 1.520 | 5 | 7 | 430 | sensory organ development |
| GO:0042478 | 0.200 | 4.749 | 0 | 1 | 20 | regulation of eye photoreceptor cell development |
| GO:0006458 | 0.200 | 4.749 | 0 | 1 | 20 | 'de novo' protein folding |
| GO:0009084 | 0.200 | 4.749 | 0 | 1 | 20 | glutamine family amino acid biosynthetic process |
| GO:0060911 | 0.200 | 4.749 | 0 | 1 | 20 | cardiac cell fate commitment |
| GO:0019217 | 0.200 | 4.749 | 0 | 1 | 20 | regulation of fatty acid metabolic process |
| GO:0050870 | 0.200 | 4.749 | 0 | 1 | 20 | positive regulation of T cell activation |
| GO:0042633 | 0.200 | 4.749 | 0 | 1 | 20 | hair cycle |
| GO:0045314 | 0.200 | 4.749 | 0 | 1 | 20 | regulation of compound eye photoreceptor development |
| GO:0022037 | 0.200 | 4.749 | 0 | 1 | 20 | metencephalon development |
| GO:0001703 | 0.200 | 4.749 | 0 | 1 | 20 | gastrulation with mouth forming first |
| GO:0006901 | 0.200 | 4.749 | 0 | 1 | 20 | vesicle coating |
| GO:0032943 | 0.200 | 4.749 | 0 | 1 | 20 | mononuclear cell proliferation |
| GO:0033059 | 0.200 | 4.749 | 0 | 1 | 20 | cellular pigmentation |
| GO:0043277 | 0.200 | 4.749 | 0 | 1 | 20 | apoptotic cell clearance |
| GO:0007279 | 0.200 | 4.749 | 0 | 1 | 20 | pole cell formation |
| GO:0043691 | 0.200 | 4.749 | 0 | 1 | 20 | reverse cholesterol transport |
| GO:0010004 | 0.200 | 4.749 | 0 | 1 | 20 | gastrulation involving germ band extension |
| GO:0046470 | 0.200 | 4.749 | 0 | 1 | 20 | phosphatidylcholine metabolic process |
| GO:0033238 | 0.200 | 4.749 | 0 | 1 | 20 | regulation of cellular amine metabolic process |
| GO:1901019 | 0.200 | 4.749 | 0 | 1 | 20 | regulation of calcium ion transmembrane transporter activity |
| GO:0033875 | 0.200 | 4.749 | 0 | 1 | 20 | ribonucleoside bisphosphate metabolic process |
| GO:0046651 | 0.200 | 4.749 | 0 | 1 | 20 | lymphocyte proliferation |
| GO:0034032 | 0.200 | 4.749 | 0 | 1 | 20 | purine nucleoside bisphosphate metabolic process |
| GO:0034368 | 0.200 | 4.749 | 0 | 1 | 20 | protein-lipid complex remodeling |
| GO:0034369 | 0.200 | 4.749 | 0 | 1 | 20 | plasma lipoprotein particle remodeling |
| GO:0016319 | 0.200 | 4.749 | 0 | 1 | 20 | mushroom body development |
| GO:0003230 | 0.200 | 4.749 | 0 | 1 | 20 | cardiac atrium development |
| GO:0042074 | 0.200 | 4.749 | 0 | 1 | 20 | cell migration involved in gastrulation |
| GO:0043405 | 0.201 | 2.483 | 1 | 2 | 75 | regulation of MAP kinase activity |
| GO:0051606 | 0.201 | 2.483 | 1 | 2 | 75 | detection of stimulus |
| GO:0070887 | 0.203 | 1.463 | 6 | 8 | 511 | cellular response to chemical stimulus |
| GO:0048870 | 0.205 | 1.501 | 5 | 7 | 435 | cell motility |
| GO:0032318 | 0.205 | 2.449 | 1 | 2 | 76 | regulation of Ras GTPase activity |
| GO:2000027 | 0.205 | 2.449 | 1 | 2 | 76 | regulation of organ morphogenesis |
| GO:0043009 | 0.207 | 1.753 | 2 | 4 | 212 | chordate embryonic development |
| GO:0042430 | 0.208 | 4.511 | 0 | 1 | 21 | indole-containing compound metabolic process |
| GO:0009081 | 0.208 | 4.511 | 0 | 1 | 21 | branched-chain amino acid metabolic process |
| GO:0042559 | 0.208 | 4.511 | 0 | 1 | 21 | pteridine-containing compound biosynthetic process |
| GO:0006586 | 0.208 | 4.511 | 0 | 1 | 21 | indolalkylamine metabolic process |
| GO:0045448 | 0.208 | 4.511 | 0 | 1 | 21 | mitotic cell cycle, embryonic |
| GO:0001737 | 0.208 | 4.511 | 0 | 1 | 21 | establishment of imaginal disc-derived wing hair orientation |
| GO:0001837 | 0.208 | 4.511 | 0 | 1 | 21 | epithelial to mesenchymal transition |
| GO:0032873 | 0.208 | 4.511 | 0 | 1 | 21 | negative regulation of stress-activated MAPK cascade |
| GO:0050921 | 0.208 | 4.511 | 0 | 1 | 21 | positive regulation of chemotaxis |
| GO:2001257 | 0.208 | 4.511 | 0 | 1 | 21 | regulation of cation channel activity |
| GO:0035195 | 0.208 | 4.511 | 0 | 1 | 21 | gene silencing by miRNA |
| GO:0009996 | 0.208 | 4.511 | 0 | 1 | 21 | negative regulation of cell fate specification |
| GO:0043255 | 0.208 | 4.511 | 0 | 1 | 21 | regulation of carbohydrate biosynthetic process |
| GO:0043506 | 0.208 | 4.511 | 0 | 1 | 21 | regulation of JUN kinase activity |
| GO:0007588 | 0.208 | 4.511 | 0 | 1 | 21 | excretion |
| GO:0002221 | 0.208 | 4.511 | 0 | 1 | 21 | pattern recognition receptor signaling pathway |
| GO:0002758 | 0.208 | 4.511 | 0 | 1 | 21 | innate immune response-activating signal transduction |
| GO:0051702 | 0.208 | 4.511 | 0 | 1 | 21 | interaction with symbiont |
| GO:0010454 | 0.208 | 4.511 | 0 | 1 | 21 | negative regulation of cell fate commitment |
| GO:0010669 | 0.208 | 4.511 | 0 | 1 | 21 | epithelial structure maintenance |
| GO:0070303 | 0.208 | 4.511 | 0 | 1 | 21 | negative regulation of stress-activated protein kinase signaling cascade |
| GO:0034367 | 0.208 | 4.511 | 0 | 1 | 21 | macromolecular complex remodeling |
| GO:0070661 | 0.208 | 4.511 | 0 | 1 | 21 | leukocyte proliferation |
| GO:0006793 | 0.209 | 1.417 | 7 | 9 | 594 | phosphorus metabolic process |
| GO:0045936 | 0.209 | 2.416 | 1 | 2 | 77 | negative regulation of phosphate metabolic process |
| GO:0046474 | 0.209 | 2.416 | 1 | 2 | 77 | glycerophospholipid biosynthetic process |
| GO:0018393 | 0.209 | 2.416 | 1 | 2 | 77 | internal peptidyl-lysine acetylation |
| GO:0010563 | 0.209 | 2.416 | 1 | 2 | 77 | negative regulation of phosphorus metabolic process |
| GO:0051674 | 0.211 | 1.487 | 5 | 7 | 439 | localization of cell |
| GO:0001568 | 0.213 | 1.934 | 2 | 3 | 144 | blood vessel development |
| GO:0010324 | 0.213 | 2.384 | 1 | 2 | 78 | membrane invagination |
| GO:0042472 | 0.217 | 4.295 | 0 | 1 | 22 | inner ear morphogenesis |
| GO:0006119 | 0.217 | 4.295 | 0 | 1 | 22 | oxidative phosphorylation |
| GO:0050663 | 0.217 | 4.295 | 0 | 1 | 22 | cytokine secretion |
| GO:0045669 | 0.217 | 4.295 | 0 | 1 | 22 | positive regulation of osteoblast differentiation |
| GO:0007432 | 0.217 | 4.295 | 0 | 1 | 22 | salivary gland boundary specification |
| GO:0030500 | 0.217 | 4.295 | 0 | 1 | 22 | regulation of bone mineralization |
| GO:0044550 | 0.217 | 4.295 | 0 | 1 | 22 | secondary metabolite biosynthetic process |
| GO:0006475 | 0.217 | 2.353 | 1 | 2 | 79 | internal protein amino acid acetylation |
| GO:0007281 | 0.219 | 1.711 | 2 | 4 | 217 | germ cell development |
| GO:0018394 | 0.221 | 2.322 | 1 | 2 | 80 | peptidyl-lysine acetylation |
| GO:0045595 | 0.226 | 1.455 | 5 | 7 | 448 | regulation of cell differentiation |
| GO:0014031 | 0.226 | 4.099 | 0 | 1 | 23 | mesenchymal cell development |
| GO:0060606 | 0.226 | 4.099 | 0 | 1 | 23 | tube closure |
| GO:0001843 | 0.226 | 4.099 | 0 | 1 | 23 | neural tube closure |
| GO:0030032 | 0.226 | 4.099 | 0 | 1 | 23 | lamellipodium assembly |
| GO:0051055 | 0.226 | 4.099 | 0 | 1 | 23 | negative regulation of lipid biosynthetic process |
| GO:0007426 | 0.226 | 4.099 | 0 | 1 | 23 | tracheal outgrowth, open tracheal system |
| GO:0002027 | 0.226 | 4.099 | 0 | 1 | 23 | regulation of heart rate |
| GO:0007277 | 0.226 | 4.099 | 0 | 1 | 23 | pole cell development |
| GO:0070167 | 0.226 | 4.099 | 0 | 1 | 23 | regulation of biomineral tissue development |
| GO:0048519 | 0.229 | 1.252 | 16 | 19 | 1436 | negative regulation of biological process |
| GO:0006874 | 0.230 | 2.264 | 1 | 2 | 82 | cellular calcium ion homeostasis |
| GO:0006457 | 0.231 | 1.853 | 2 | 3 | 150 | protein folding |
| GO:0006915 | 0.233 | 1.374 | 7 | 9 | 611 | apoptotic process |
| GO:0051239 | 0.233 | 1.315 | 9 | 12 | 855 | regulation of multicellular organismal process |
| GO:0008284 | 0.233 | 1.841 | 2 | 3 | 151 | positive regulation of cell proliferation |
| GO:0045333 | 0.234 | 2.236 | 1 | 2 | 83 | cellular respiration |
| GO:0014020 | 0.234 | 3.921 | 0 | 1 | 24 | primary neural tube formation |
| GO:0006536 | 0.234 | 3.921 | 0 | 1 | 24 | glutamate metabolic process |
| GO:0006900 | 0.234 | 3.921 | 0 | 1 | 24 | membrane budding |
| GO:0043433 | 0.234 | 3.921 | 0 | 1 | 24 | negative regulation of sequence-specific DNA binding transcription factor activity |
| GO:0007413 | 0.234 | 3.921 | 0 | 1 | 24 | axonal fasciculation |
| GO:0007229 | 0.234 | 3.921 | 0 | 1 | 24 | integrin-mediated signaling pathway |
| GO:0022904 | 0.234 | 3.921 | 0 | 1 | 24 | respiratory electron transport chain |
| GO:0051251 | 0.234 | 3.921 | 0 | 1 | 24 | positive regulation of lymphocyte activation |
| GO:0046164 | 0.234 | 3.921 | 0 | 1 | 24 | alcohol catabolic process |
| GO:0002696 | 0.234 | 3.921 | 0 | 1 | 24 | positive regulation of leukocyte activation |
| GO:0006094 | 0.234 | 3.921 | 0 | 1 | 24 | gluconeogenesis |
| GO:0048523 | 0.235 | 1.259 | 14 | 17 | 1273 | negative regulation of cellular process |
| GO:0009150 | 0.236 | 1.482 | 4 | 6 | 376 | purine ribonucleotide metabolic process |
| GO:0001944 | 0.236 | 1.828 | 2 | 3 | 152 | vasculature development |
| GO:0010608 | 0.238 | 1.647 | 2 | 4 | 225 | posttranscriptional regulation of gene expression |
| GO:0048477 | 0.238 | 1.478 | 4 | 6 | 377 | oogenesis |
| GO:0090305 | 0.240 | 1.640 | 2 | 4 | 226 | nucleic acid phosphodiester bond hydrolysis |
| GO:0006473 | 0.242 | 2.181 | 1 | 2 | 85 | protein acetylation |
| GO:0071375 | 0.242 | 2.181 | 1 | 2 | 85 | cellular response to peptide hormone stimulus |
| GO:0072503 | 0.242 | 2.181 | 1 | 2 | 85 | cellular divalent inorganic cation homeostasis |
| GO:0001751 | 0.242 | 1.803 | 2 | 3 | 154 | compound eye photoreceptor cell differentiation |
| GO:0042743 | 0.243 | 3.757 | 0 | 1 | 25 | hydrogen peroxide metabolic process |
| GO:0050867 | 0.243 | 3.757 | 0 | 1 | 25 | positive regulation of cell activation |
| GO:0006506 | 0.243 | 3.757 | 0 | 1 | 25 | GPI anchor biosynthetic process |
| GO:0045746 | 0.243 | 3.757 | 0 | 1 | 25 | negative regulation of Notch signaling pathway |
| GO:0048103 | 0.243 | 3.757 | 0 | 1 | 25 | somatic stem cell division |
| GO:0048762 | 0.243 | 3.757 | 0 | 1 | 25 | mesenchymal cell differentiation |
| GO:0048844 | 0.243 | 3.757 | 0 | 1 | 25 | artery morphogenesis |
| GO:0043407 | 0.243 | 3.757 | 0 | 1 | 25 | negative regulation of MAP kinase activity |
| GO:0007157 | 0.243 | 3.757 | 0 | 1 | 25 | heterophilic cell-cell adhesion |
| GO:0007188 | 0.243 | 3.757 | 0 | 1 | 25 | adenylate cyclase-modulating G-protein coupled receptor signaling pathway |
| GO:0033002 | 0.243 | 3.757 | 0 | 1 | 25 | muscle cell proliferation |
| GO:0002460 | 0.243 | 3.757 | 0 | 1 | 25 | adaptive immune response based on somatic recombination of immune receptors built from immunoglobulin superfamily domains |
| GO:0051100 | 0.243 | 3.757 | 0 | 1 | 25 | negative regulation of binding |
| GO:0051101 | 0.243 | 3.757 | 0 | 1 | 25 | regulation of DNA binding |
| GO:0043966 | 0.243 | 3.757 | 0 | 1 | 25 | histone H3 acetylation |
| GO:0051568 | 0.243 | 3.757 | 0 | 1 | 25 | histone H3-K4 methylation |
| GO:0008299 | 0.243 | 3.757 | 0 | 1 | 25 | isoprenoid biosynthetic process |
| GO:0010906 | 0.243 | 3.757 | 0 | 1 | 25 | regulation of glucose metabolic process |
| GO:0008543 | 0.243 | 3.757 | 0 | 1 | 25 | fibroblast growth factor receptor signaling pathway |
| GO:0009880 | 0.245 | 1.791 | 2 | 3 | 155 | embryonic pattern specification |
| GO:0042067 | 0.246 | 2.155 | 1 | 2 | 86 | establishment of ommatidial planar polarity |
| GO:0010721 | 0.250 | 2.129 | 1 | 2 | 87 | negative regulation of cell development |
| GO:0055074 | 0.250 | 2.129 | 1 | 2 | 87 | calcium ion homeostasis |
| GO:0009952 | 0.251 | 1.768 | 2 | 3 | 157 | anterior/posterior pattern specification |
| GO:0060840 | 0.251 | 3.606 | 0 | 1 | 26 | artery development |
| GO:0050863 | 0.251 | 3.606 | 0 | 1 | 26 | regulation of T cell activation |
| GO:0050805 | 0.251 | 3.606 | 0 | 1 | 26 | negative regulation of synaptic transmission |
| GO:0009268 | 0.251 | 3.606 | 0 | 1 | 26 | response to pH |
| GO:0009746 | 0.251 | 3.606 | 0 | 1 | 26 | response to hexose stimulus |
| GO:0009749 | 0.251 | 3.606 | 0 | 1 | 26 | response to glucose stimulus |
| GO:0071774 | 0.251 | 3.606 | 0 | 1 | 26 | response to fibroblast growth factor stimulus |
| GO:0071825 | 0.251 | 3.606 | 0 | 1 | 26 | protein-lipid complex subunit organization |
| GO:0071827 | 0.251 | 3.606 | 0 | 1 | 26 | plasma lipoprotein particle organization |
| GO:0048520 | 0.251 | 3.606 | 0 | 1 | 26 | positive regulation of behavior |
| GO:0030516 | 0.251 | 3.606 | 0 | 1 | 26 | regulation of axon extension |
| GO:0002449 | 0.251 | 3.606 | 0 | 1 | 26 | lymphocyte mediated immunity |
| GO:0007589 | 0.251 | 3.606 | 0 | 1 | 26 | body fluid secretion |
| GO:0002250 | 0.251 | 3.606 | 0 | 1 | 26 | adaptive immune response |
| GO:0030902 | 0.251 | 3.606 | 0 | 1 | 26 | hindbrain development |
| GO:0046532 | 0.251 | 3.606 | 0 | 1 | 26 | regulation of photoreceptor cell differentiation |
| GO:0051970 | 0.251 | 3.606 | 0 | 1 | 26 | negative regulation of transmission of nerve impulse |
| GO:0044344 | 0.251 | 3.606 | 0 | 1 | 26 | cellular response to fibroblast growth factor stimulus |
| GO:0010951 | 0.251 | 3.606 | 0 | 1 | 26 | negative regulation of endopeptidase activity |
| GO:0003279 | 0.251 | 3.606 | 0 | 1 | 26 | cardiac septum development |
| GO:0034284 | 0.251 | 3.606 | 0 | 1 | 26 | response to monosaccharide stimulus |
| GO:0030097 | 0.254 | 1.756 | 2 | 3 | 158 | hemopoiesis |
| GO:0035239 | 0.254 | 1.756 | 2 | 3 | 158 | tube morphogenesis |
| GO:0051128 | 0.255 | 1.316 | 8 | 10 | 708 | regulation of cellular component organization |
| GO:1901136 | 0.258 | 1.433 | 4 | 6 | 388 | carbohydrate derivative catabolic process |
| GO:0016246 | 0.260 | 3.467 | 0 | 1 | 27 | RNA interference |
| GO:0019319 | 0.260 | 3.467 | 0 | 1 | 27 | hexose biosynthetic process |
| GO:0040018 | 0.260 | 3.467 | 0 | 1 | 27 | positive regulation of multicellular organism growth |
| GO:0001101 | 0.260 | 3.467 | 0 | 1 | 27 | response to acid |
| GO:0045198 | 0.260 | 3.467 | 0 | 1 | 27 | establishment of epithelial cell apical/basal polarity |
| GO:0007187 | 0.260 | 3.467 | 0 | 1 | 27 | G-protein coupled receptor signaling pathway, coupled to cyclic nucleotide second messenger |
| GO:0007205 | 0.260 | 3.467 | 0 | 1 | 27 | protein kinase C-activating G-protein coupled receptor signaling pathway |
| GO:0046364 | 0.260 | 3.467 | 0 | 1 | 27 | monosaccharide biosynthetic process |
| GO:0010466 | 0.260 | 3.467 | 0 | 1 | 27 | negative regulation of peptidase activity |
| GO:0010883 | 0.260 | 3.467 | 0 | 1 | 27 | regulation of lipid storage |
| GO:0031348 | 0.260 | 3.467 | 0 | 1 | 27 | negative regulation of defense response |
| GO:0009259 | 0.260 | 1.429 | 4 | 6 | 389 | ribonucleotide metabolic process |
| GO:0043543 | 0.262 | 2.056 | 1 | 2 | 90 | protein acylation |
| GO:0010769 | 0.262 | 2.056 | 1 | 2 | 90 | regulation of cell morphogenesis involved in differentiation |
| GO:0016044 | 0.262 | 1.483 | 3 | 5 | 312 | cellular membrane organization |
| GO:0007292 | 0.264 | 1.422 | 4 | 6 | 391 | female gamete generation |
| GO:0061024 | 0.265 | 1.478 | 3 | 5 | 313 | membrane organization |
| GO:0010646 | 0.265 | 1.322 | 7 | 9 | 633 | regulation of cell communication |
| GO:0044282 | 0.266 | 1.711 | 2 | 3 | 162 | small molecule catabolic process |
| GO:0010927 | 0.266 | 1.711 | 2 | 3 | 162 | cellular component assembly involved in morphogenesis |
| GO:0032940 | 0.267 | 1.473 | 3 | 5 | 314 | secretion by cell |
| GO:0042471 | 0.268 | 3.338 | 0 | 1 | 28 | ear morphogenesis |
| GO:0032319 | 0.268 | 3.338 | 0 | 1 | 28 | regulation of Rho GTPase activity |
| GO:0042659 | 0.268 | 3.338 | 0 | 1 | 28 | regulation of cell fate specification |
| GO:0006505 | 0.268 | 3.338 | 0 | 1 | 28 | GPI anchor metabolic process |
| GO:0035089 | 0.268 | 3.338 | 0 | 1 | 28 | establishment of apical/basal cell polarity |
| GO:0071897 | 0.268 | 3.338 | 0 | 1 | 28 | DNA biosynthetic process |
| GO:0061387 | 0.268 | 3.338 | 0 | 1 | 28 | regulation of extent of cell growth |
| GO:0048846 | 0.268 | 3.338 | 0 | 1 | 28 | axon extension involved in axon guidance |
| GO:0030217 | 0.268 | 3.338 | 0 | 1 | 28 | T cell differentiation |
| GO:0007218 | 0.268 | 3.338 | 0 | 1 | 28 | neuropeptide signaling pathway |
| GO:0010632 | 0.268 | 3.338 | 0 | 1 | 28 | regulation of epithelial cell migration |
| GO:0007264 | 0.269 | 1.553 | 3 | 4 | 238 | small GTPase mediated signal transduction |
| GO:0051960 | 0.269 | 1.553 | 3 | 4 | 238 | regulation of nervous system development |
| GO:0001501 | 0.275 | 1.987 | 1 | 2 | 93 | skeletal system development |
| GO:0072507 | 0.275 | 1.987 | 1 | 2 | 93 | divalent inorganic cation homeostasis |
| GO:0001754 | 0.275 | 1.679 | 2 | 3 | 165 | eye photoreceptor cell differentiation |
| GO:0032103 | 0.276 | 3.219 | 0 | 1 | 29 | positive regulation of response to external stimulus |
| GO:0045833 | 0.276 | 3.219 | 0 | 1 | 29 | negative regulation of lipid metabolic process |
| GO:0022409 | 0.276 | 3.219 | 0 | 1 | 29 | positive regulation of cell-cell adhesion |
| GO:0061339 | 0.276 | 3.219 | 0 | 1 | 29 | establishment or maintenance of monopolar cell polarity |
| GO:0001935 | 0.276 | 3.219 | 0 | 1 | 29 | endothelial cell proliferation |
| GO:0061162 | 0.276 | 3.219 | 0 | 1 | 29 | establishment of monopolar cell polarity |
| GO:0030282 | 0.276 | 3.219 | 0 | 1 | 29 | bone mineralization |
| GO:0030859 | 0.276 | 3.219 | 0 | 1 | 29 | polarized epithelial cell differentiation |
| GO:0030539 | 0.276 | 3.219 | 0 | 1 | 29 | male genitalia development |
| GO:0010453 | 0.276 | 3.219 | 0 | 1 | 29 | regulation of cell fate commitment |
| GO:0051928 | 0.276 | 3.219 | 0 | 1 | 29 | positive regulation of calcium ion transport |
| GO:0060446 | 0.276 | 3.219 | 0 | 1 | 29 | branching involved in open tracheal system development |
| GO:0006027 | 0.276 | 3.219 | 0 | 1 | 29 | glycosaminoglycan catabolic process |
| GO:0006281 | 0.277 | 1.449 | 4 | 5 | 319 | DNA repair |
| GO:0045321 | 0.279 | 1.966 | 1 | 2 | 94 | leukocyte activation |
| GO:0030258 | 0.279 | 1.966 | 1 | 2 | 94 | lipid modification |
| GO:0006184 | 0.281 | 1.658 | 2 | 3 | 167 | GTP catabolic process |
| GO:0006520 | 0.282 | 1.439 | 4 | 5 | 321 | cellular amino acid metabolic process |
| GO:0071840 | 0.283 | 1.160 | 30 | 33 | 2711 | cellular component organization or biogenesis |
| GO:0006144 | 0.284 | 3.107 | 0 | 1 | 30 | purine nucleobase metabolic process |
| GO:0040010 | 0.284 | 3.107 | 0 | 1 | 30 | positive regulation of growth rate |
| GO:0048190 | 0.284 | 3.107 | 0 | 1 | 30 | wing disc dorsal/ventral pattern formation |
| GO:0035159 | 0.284 | 3.107 | 0 | 1 | 30 | regulation of tube length, open tracheal system |
| GO:0007520 | 0.284 | 3.107 | 0 | 1 | 30 | myoblast fusion |
| GO:0007525 | 0.284 | 3.107 | 0 | 1 | 30 | somatic muscle development |
| GO:0030968 | 0.284 | 3.107 | 0 | 1 | 30 | endoplasmic reticulum unfolded protein response |
| GO:0000096 | 0.284 | 3.107 | 0 | 1 | 30 | sulfur amino acid metabolic process |
| GO:0031645 | 0.284 | 3.107 | 0 | 1 | 30 | negative regulation of neurological system process |
| GO:1901069 | 0.284 | 1.648 | 2 | 3 | 168 | guanosine-containing compound catabolic process |
| GO:0006195 | 0.286 | 1.430 | 4 | 5 | 323 | purine nucleotide catabolic process |
| GO:0045927 | 0.287 | 1.923 | 1 | 2 | 96 | positive regulation of growth |
| GO:0007498 | 0.287 | 1.923 | 1 | 2 | 96 | mesoderm development |
| GO:0048878 | 0.290 | 1.421 | 4 | 5 | 325 | chemical homeostasis |
| GO:0050804 | 0.291 | 1.903 | 1 | 2 | 97 | regulation of synaptic transmission |
| GO:0071345 | 0.291 | 1.903 | 1 | 2 | 97 | cellular response to cytokine stimulus |
| GO:0034620 | 0.292 | 3.003 | 0 | 1 | 31 | cellular response to unfolded protein |
| GO:0034612 | 0.292 | 3.003 | 0 | 1 | 31 | response to tumor necrosis factor |
| GO:0060856 | 0.292 | 3.003 | 0 | 1 | 31 | establishment of blood-brain barrier |
| GO:0006754 | 0.292 | 3.003 | 0 | 1 | 31 | ATP biosynthetic process |
| GO:0035050 | 0.292 | 3.003 | 0 | 1 | 31 | embryonic heart tube development |
| GO:0032851 | 0.292 | 3.003 | 0 | 1 | 31 | positive regulation of Rab GTPase activity |
| GO:0006904 | 0.292 | 3.003 | 0 | 1 | 31 | vesicle docking involved in exocytosis |
| GO:0051291 | 0.292 | 3.003 | 0 | 1 | 31 | protein heterooligomerization |
| GO:0051249 | 0.292 | 3.003 | 0 | 1 | 31 | regulation of lymphocyte activation |
| GO:0090162 | 0.292 | 3.003 | 0 | 1 | 31 | establishment of epithelial cell polarity |
| GO:0051650 | 0.292 | 3.003 | 0 | 1 | 31 | establishment of vesicle localization |
| GO:0000768 | 0.292 | 3.003 | 0 | 1 | 31 | syncytium formation by plasma membrane fusion |
| GO:0008105 | 0.292 | 3.003 | 0 | 1 | 31 | asymmetric protein localization |
| GO:0060485 | 0.292 | 3.003 | 0 | 1 | 31 | mesenchyme development |
| GO:0050808 | 0.293 | 1.617 | 2 | 3 | 171 | synapse organization |
| GO:0046039 | 0.293 | 1.617 | 2 | 3 | 171 | GTP metabolic process |
| GO:0048610 | 0.294 | 1.299 | 6 | 8 | 570 | cellular process involved in reproduction |
| GO:0030001 | 0.296 | 1.608 | 2 | 3 | 172 | metal ion transport |
| GO:0048534 | 0.296 | 1.608 | 2 | 3 | 172 | hemopoietic or lymphoid organ development |
| GO:0009792 | 0.298 | 1.355 | 5 | 6 | 409 | embryo development ending in birth or egg hatching |
| GO:0042454 | 0.299 | 1.598 | 2 | 3 | 173 | ribonucleoside catabolic process |
| GO:0006152 | 0.299 | 1.598 | 2 | 3 | 173 | purine nucleoside catabolic process |
| GO:0045087 | 0.299 | 1.598 | 2 | 3 | 173 | innate immune response |
| GO:0046130 | 0.299 | 1.598 | 2 | 3 | 173 | purine ribonucleoside catabolic process |
| GO:0016458 | 0.299 | 1.863 | 1 | 2 | 99 | gene silencing |
| GO:0032313 | 0.300 | 2.906 | 0 | 1 | 32 | regulation of Rab GTPase activity |
| GO:0050727 | 0.300 | 2.906 | 0 | 1 | 32 | regulation of inflammatory response |
| GO:0040009 | 0.300 | 2.906 | 0 | 1 | 32 | regulation of growth rate |
| GO:0032483 | 0.300 | 2.906 | 0 | 1 | 32 | regulation of Rab protein signal transduction |
| GO:0048011 | 0.300 | 2.906 | 0 | 1 | 32 | nerve growth factor receptor signaling pathway |
| GO:0009187 | 0.300 | 2.906 | 0 | 1 | 32 | cyclic nucleotide metabolic process |
| GO:0006949 | 0.300 | 2.906 | 0 | 1 | 32 | syncytium formation |
| GO:0030048 | 0.300 | 2.906 | 0 | 1 | 32 | actin filament-based movement |
| GO:0050919 | 0.300 | 2.906 | 0 | 1 | 32 | negative chemotaxis |
| GO:0050906 | 0.300 | 2.906 | 0 | 1 | 32 | detection of stimulus involved in sensory perception |
| GO:0045807 | 0.300 | 2.906 | 0 | 1 | 32 | positive regulation of endocytosis |
| GO:0007041 | 0.300 | 2.906 | 0 | 1 | 32 | lysosomal transport |
| GO:0048278 | 0.300 | 2.906 | 0 | 1 | 32 | vesicle docking |
| GO:0014902 | 0.300 | 2.906 | 0 | 1 | 32 | myotube differentiation |
| GO:0007450 | 0.300 | 2.906 | 0 | 1 | 32 | dorsal/ventral pattern formation, imaginal disc |
| GO:0023021 | 0.300 | 2.906 | 0 | 1 | 32 | termination of signal transduction |
| GO:0006952 | 0.303 | 1.393 | 4 | 5 | 331 | defense response |
| GO:0060560 | 0.303 | 1.844 | 1 | 2 | 100 | developmental growth involved in morphogenesis |
| GO:0048863 | 0.303 | 1.844 | 1 | 2 | 100 | stem cell differentiation |
| GO:0019538 | 0.304 | 1.153 | 24 | 27 | 2207 | protein metabolic process |
| GO:0022607 | 0.305 | 1.213 | 11 | 13 | 996 | cellular component assembly |
| GO:0007050 | 0.305 | 1.579 | 2 | 3 | 175 | cell cycle arrest |
| GO:0048638 | 0.308 | 1.825 | 1 | 2 | 101 | regulation of developmental growth |
| GO:0007584 | 0.308 | 1.825 | 1 | 2 | 101 | response to nutrient |
| GO:0009166 | 0.308 | 1.385 | 4 | 5 | 333 | nucleotide catabolic process |
| GO:0032102 | 0.308 | 2.815 | 0 | 1 | 33 | negative regulation of response to external stimulus |
| GO:0001841 | 0.308 | 2.815 | 0 | 1 | 33 | neural tube formation |
| GO:0043094 | 0.308 | 2.815 | 0 | 1 | 33 | cellular metabolic compound salvage |
| GO:0022406 | 0.308 | 2.815 | 0 | 1 | 33 | membrane docking |
| GO:0048738 | 0.308 | 2.815 | 0 | 1 | 33 | cardiac muscle tissue development |
| GO:0010675 | 0.308 | 2.815 | 0 | 1 | 33 | regulation of cellular carbohydrate metabolic process |
| GO:0007169 | 0.309 | 1.451 | 3 | 4 | 254 | transmembrane receptor protein tyrosine kinase signaling pathway |
| GO:0040008 | 0.310 | 1.380 | 4 | 5 | 334 | regulation of growth |
| GO:0009164 | 0.311 | 1.561 | 2 | 3 | 177 | nucleoside catabolic process |
| GO:0048592 | 0.311 | 1.445 | 3 | 4 | 255 | eye morphogenesis |
| GO:0006875 | 0.312 | 1.807 | 1 | 2 | 102 | cellular metal ion homeostasis |
| GO:1901292 | 0.312 | 1.376 | 4 | 5 | 335 | nucleoside phosphate catabolic process |
| GO:0072523 | 0.314 | 1.371 | 4 | 5 | 336 | purine-containing compound catabolic process |
| GO:0016570 | 0.315 | 1.551 | 2 | 3 | 178 | histone modification |
| GO:0006109 | 0.315 | 2.729 | 0 | 1 | 34 | regulation of carbohydrate metabolic process |
| GO:0009206 | 0.315 | 2.729 | 0 | 1 | 34 | purine ribonucleoside triphosphate biosynthetic process |
| GO:0002064 | 0.315 | 2.729 | 0 | 1 | 34 | epithelial cell development |
| GO:0010160 | 0.315 | 2.729 | 0 | 1 | 34 | formation of organ boundary |
| GO:0018205 | 0.316 | 1.788 | 1 | 2 | 103 | peptidyl-lysine modification |
| GO:0010975 | 0.316 | 1.788 | 1 | 2 | 103 | regulation of neuron projection development |
| GO:0048589 | 0.317 | 1.433 | 3 | 4 | 257 | developmental growth |
| GO:0022414 | 0.317 | 1.192 | 12 | 14 | 1091 | reproductive process |
| GO:0016569 | 0.318 | 1.542 | 2 | 3 | 179 | covalent chromatin modification |
| GO:0001654 | 0.319 | 1.363 | 4 | 5 | 338 | eye development |
| GO:0007276 | 0.319 | 1.263 | 6 | 8 | 585 | gamete generation |
| GO:0006022 | 0.320 | 1.771 | 1 | 2 | 104 | aminoglycan metabolic process |
| GO:0071495 | 0.321 | 1.534 | 2 | 3 | 180 | cellular response to endogenous stimulus |
| GO:0006497 | 0.323 | 2.649 | 0 | 1 | 35 | protein lipidation |
| GO:0042158 | 0.323 | 2.649 | 0 | 1 | 35 | lipoprotein biosynthetic process |
| GO:0006582 | 0.323 | 2.649 | 0 | 1 | 35 | melanin metabolic process |
| GO:0035071 | 0.323 | 2.649 | 0 | 1 | 35 | salivary gland cell autophagic cell death |
| GO:0009145 | 0.323 | 2.649 | 0 | 1 | 35 | purine nucleoside triphosphate biosynthetic process |
| GO:0001819 | 0.323 | 2.649 | 0 | 1 | 35 | positive regulation of cytokine production |
| GO:0043410 | 0.323 | 2.649 | 0 | 1 | 35 | positive regulation of MAPK cascade |
| GO:0043409 | 0.323 | 2.649 | 0 | 1 | 35 | negative regulation of MAPK cascade |
| GO:0030518 | 0.323 | 2.649 | 0 | 1 | 35 | intracellular steroid hormone receptor signaling pathway |
| GO:0033344 | 0.323 | 2.649 | 0 | 1 | 35 | cholesterol efflux |
| GO:0002443 | 0.323 | 2.649 | 0 | 1 | 35 | leukocyte mediated immunity |
| GO:0035967 | 0.323 | 2.649 | 0 | 1 | 35 | cellular response to topologically incorrect protein |
| GO:0043967 | 0.323 | 2.649 | 0 | 1 | 35 | histone H4 acetylation |
| GO:0031333 | 0.323 | 2.649 | 0 | 1 | 35 | negative regulation of protein complex assembly |
| GO:0009205 | 0.323 | 1.354 | 4 | 5 | 340 | purine ribonucleoside triphosphate metabolic process |
| GO:0043161 | 0.324 | 1.753 | 1 | 2 | 105 | proteasomal ubiquitin-dependent protein catabolic process |
| GO:0034655 | 0.325 | 1.350 | 4 | 5 | 341 | nucleobase-containing compound catabolic process |
| GO:0007626 | 0.327 | 1.516 | 2 | 3 | 182 | locomotory behavior |
| GO:0009144 | 0.327 | 1.346 | 4 | 5 | 342 | purine nucleoside triphosphate metabolic process |
| GO:0051969 | 0.328 | 1.736 | 1 | 2 | 106 | regulation of transmission of nerve impulse |
| GO:0007610 | 0.328 | 1.273 | 6 | 7 | 507 | behavior |
| GO:0050793 | 0.330 | 1.216 | 8 | 10 | 760 | regulation of developmental process |
| GO:0009066 | 0.330 | 2.573 | 0 | 1 | 36 | aspartate family amino acid metabolic process |
| GO:0006635 | 0.330 | 2.573 | 0 | 1 | 36 | fatty acid beta-oxidation |
| GO:0001707 | 0.330 | 2.573 | 0 | 1 | 36 | mesoderm formation |
| GO:0006984 | 0.330 | 2.573 | 0 | 1 | 36 | ER-nucleus signaling pathway |
| GO:0048332 | 0.330 | 2.573 | 0 | 1 | 36 | mesoderm morphogenesis |
| GO:0045667 | 0.330 | 2.573 | 0 | 1 | 36 | regulation of osteoblast differentiation |
| GO:0043542 | 0.330 | 2.573 | 0 | 1 | 36 | endothelial cell migration |
| GO:0007565 | 0.330 | 2.573 | 0 | 1 | 36 | female pregnancy |
| GO:0003205 | 0.330 | 2.573 | 0 | 1 | 36 | cardiac chamber development |
| GO:0048585 | 0.332 | 1.338 | 4 | 5 | 344 | negative regulation of response to stimulus |
| GO:0006796 | 0.333 | 1.245 | 7 | 8 | 593 | phosphate-containing compound metabolic process |
| GO:0035282 | 0.333 | 1.499 | 2 | 3 | 184 | segmentation |
| GO:0002520 | 0.333 | 1.499 | 2 | 3 | 184 | immune system development |
| GO:0046530 | 0.333 | 1.499 | 2 | 3 | 184 | photoreceptor cell differentiation |
| GO:0009199 | 0.334 | 1.334 | 4 | 5 | 345 | ribonucleoside triphosphate metabolic process |
| GO:0007219 | 0.336 | 1.703 | 1 | 2 | 108 | Notch signaling pathway |
| GO:0051241 | 0.336 | 1.703 | 1 | 2 | 108 | negative regulation of multicellular organismal process |
| GO:0031644 | 0.336 | 1.703 | 1 | 2 | 108 | regulation of neurological system process |
| GO:0034220 | 0.336 | 1.703 | 1 | 2 | 108 | ion transmembrane transport |
| GO:0046434 | 0.336 | 1.330 | 4 | 5 | 346 | organophosphate catabolic process |
| GO:0006275 | 0.338 | 2.501 | 0 | 1 | 37 | regulation of DNA replication |
| GO:0034968 | 0.338 | 2.501 | 0 | 1 | 37 | histone lysine methylation |
| GO:2001056 | 0.338 | 2.501 | 0 | 1 | 37 | positive regulation of cysteine-type endopeptidase activity |
| GO:0097006 | 0.338 | 2.501 | 0 | 1 | 37 | regulation of plasma lipoprotein particle levels |
| GO:0051048 | 0.338 | 2.501 | 0 | 1 | 37 | negative regulation of secretion |
| GO:0043280 | 0.338 | 2.501 | 0 | 1 | 37 | positive regulation of cysteine-type endopeptidase activity involved in apoptotic process |
| GO:0002700 | 0.338 | 2.501 | 0 | 1 | 37 | regulation of production of molecular mediator of immune response |
| GO:0010950 | 0.338 | 2.501 | 0 | 1 | 37 | positive regulation of endopeptidase activity |
| GO:0005977 | 0.338 | 2.501 | 0 | 1 | 37 | glycogen metabolic process |
| GO:0006081 | 0.338 | 2.501 | 0 | 1 | 37 | cellular aldehyde metabolic process |
| GO:0019220 | 0.341 | 1.321 | 4 | 5 | 348 | regulation of phosphate metabolic process |
| GO:0040007 | 0.341 | 1.254 | 6 | 7 | 514 | growth |
| GO:0006486 | 0.344 | 1.671 | 1 | 2 | 110 | protein glycosylation |
| GO:0043413 | 0.344 | 1.671 | 1 | 2 | 110 | macromolecule glycosylation |
| GO:0070085 | 0.344 | 1.671 | 1 | 2 | 110 | glycosylation |
| GO:0080134 | 0.344 | 1.371 | 3 | 4 | 268 | regulation of response to stress |
| GO:0044057 | 0.345 | 1.466 | 2 | 3 | 188 | regulation of system process |
| GO:0009141 | 0.345 | 1.313 | 4 | 5 | 350 | nucleoside triphosphate metabolic process |
| GO:0021953 | 0.345 | 2.433 | 0 | 1 | 38 | central nervous system neuron differentiation |
| GO:0032412 | 0.345 | 2.433 | 0 | 1 | 38 | regulation of ion transmembrane transporter activity |
| GO:0042632 | 0.345 | 2.433 | 0 | 1 | 38 | cholesterol homeostasis |
| GO:0035070 | 0.345 | 2.433 | 0 | 1 | 38 | salivary gland histolysis |
| GO:0048814 | 0.345 | 2.433 | 0 | 1 | 38 | regulation of dendrite morphogenesis |
| GO:0043270 | 0.345 | 2.433 | 0 | 1 | 38 | positive regulation of ion transport |
| GO:0007569 | 0.345 | 2.433 | 0 | 1 | 38 | cell aging |
| GO:0010952 | 0.345 | 2.433 | 0 | 1 | 38 | positive regulation of peptidase activity |
| GO:0006073 | 0.345 | 2.433 | 0 | 1 | 38 | cellular glucan metabolic process |
| GO:0051094 | 0.348 | 1.458 | 2 | 3 | 189 | positive regulation of developmental process |
| GO:0008015 | 0.348 | 1.655 | 1 | 2 | 111 | blood circulation |
| GO:0042051 | 0.352 | 1.640 | 1 | 2 | 112 | compound eye photoreceptor development |
| GO:0016271 | 0.353 | 2.369 | 0 | 1 | 39 | tissue death |
| GO:0030098 | 0.353 | 2.369 | 0 | 1 | 39 | lymphocyte differentiation |
| GO:0009201 | 0.353 | 2.369 | 0 | 1 | 39 | ribonucleoside triphosphate biosynthetic process |
| GO:0007306 | 0.353 | 2.369 | 0 | 1 | 39 | eggshell chorion assembly |
| GO:0071901 | 0.353 | 2.369 | 0 | 1 | 39 | negative regulation of protein serine/threonine kinase activity |
| GO:0007559 | 0.353 | 2.369 | 0 | 1 | 39 | histolysis |
| GO:0035914 | 0.353 | 2.369 | 0 | 1 | 39 | skeletal muscle cell differentiation |
| GO:0002694 | 0.353 | 2.369 | 0 | 1 | 39 | regulation of leukocyte activation |
| GO:0044042 | 0.353 | 2.369 | 0 | 1 | 39 | glucan metabolic process |
| GO:0031345 | 0.353 | 2.369 | 0 | 1 | 39 | negative regulation of cell projection organization |
| GO:0010498 | 0.356 | 1.625 | 1 | 2 | 113 | proteasomal protein catabolic process |
| GO:0045937 | 0.357 | 1.434 | 2 | 3 | 192 | positive regulation of phosphate metabolic process |
| GO:0010562 | 0.357 | 1.434 | 2 | 3 | 192 | positive regulation of phosphorus metabolic process |
| GO:0006888 | 0.360 | 2.308 | 0 | 1 | 40 | ER to Golgi vesicle-mediated transport |
| GO:0019218 | 0.360 | 2.308 | 0 | 1 | 40 | regulation of steroid metabolic process |
| GO:0022898 | 0.360 | 2.308 | 0 | 1 | 40 | regulation of transmembrane transporter activity |
| GO:0007455 | 0.360 | 2.308 | 0 | 1 | 40 | eye-antennal disc morphogenesis |
| GO:0007427 | 0.360 | 2.308 | 0 | 1 | 40 | epithelial cell migration, open tracheal system |
| GO:0010002 | 0.360 | 2.308 | 0 | 1 | 40 | cardioblast differentiation |
| GO:0019953 | 0.360 | 1.209 | 7 | 8 | 609 | sexual reproduction |
| GO:0045596 | 0.360 | 1.426 | 2 | 3 | 193 | negative regulation of cell differentiation |
| GO:0018130 | 0.360 | 1.426 | 2 | 3 | 193 | heterocycle biosynthetic process |
| GO:0008360 | 0.360 | 1.611 | 1 | 2 | 114 | regulation of cell shape |
| GO:0003013 | 0.360 | 1.611 | 1 | 2 | 114 | circulatory system process |
| GO:0055065 | 0.360 | 1.611 | 1 | 2 | 114 | metal ion homeostasis |
| GO:0016337 | 0.363 | 1.419 | 2 | 3 | 194 | cell-cell adhesion |
| GO:0030003 | 0.364 | 1.596 | 1 | 2 | 115 | cellular cation homeostasis |
| GO:0048609 | 0.364 | 1.189 | 8 | 9 | 697 | multicellular organismal reproductive process |
| GO:0048584 | 0.365 | 1.279 | 4 | 5 | 359 | positive regulation of response to stimulus |
| GO:0006928 | 0.367 | 1.200 | 7 | 8 | 613 | cellular component movement |
| GO:0042552 | 0.367 | 2.250 | 0 | 1 | 41 | myelination |
| GO:0032496 | 0.367 | 2.250 | 0 | 1 | 41 | response to lipopolysaccharide |
| GO:0030177 | 0.367 | 2.250 | 0 | 1 | 41 | positive regulation of Wnt receptor signaling pathway |
| GO:0033273 | 0.367 | 2.250 | 0 | 1 | 41 | response to vitamin |
| GO:0002764 | 0.367 | 2.250 | 0 | 1 | 41 | immune response-regulating signaling pathway |
| GO:0002757 | 0.367 | 2.250 | 0 | 1 | 41 | immune response-activating signal transduction |
| GO:0046148 | 0.367 | 2.250 | 0 | 1 | 41 | pigment biosynthetic process |
| GO:0055092 | 0.367 | 2.250 | 0 | 1 | 41 | sterol homeostasis |
| GO:0034765 | 0.367 | 2.250 | 0 | 1 | 41 | regulation of ion transmembrane transport |
| GO:0046700 | 0.367 | 1.275 | 4 | 5 | 360 | heterocycle catabolic process |
| GO:0016310 | 0.367 | 1.241 | 5 | 6 | 444 | phosphorylation |
| GO:0006464 | 0.368 | 1.143 | 13 | 14 | 1131 | cellular protein modification process |
| GO:0036211 | 0.368 | 1.143 | 13 | 14 | 1131 | protein modification process |
| GO:0032504 | 0.369 | 1.183 | 8 | 9 | 700 | multicellular organism reproduction |
| GO:0007167 | 0.369 | 1.271 | 4 | 5 | 361 | enzyme linked receptor protein signaling pathway |
| GO:0000003 | 0.371 | 1.141 | 13 | 14 | 1133 | reproduction |
| GO:0006342 | 0.374 | 2.195 | 0 | 1 | 42 | chromatin silencing |
| GO:0016571 | 0.374 | 2.195 | 0 | 1 | 42 | histone methylation |
| GO:0006775 | 0.374 | 2.195 | 0 | 1 | 42 | fat-soluble vitamin metabolic process |
| GO:0009112 | 0.374 | 2.195 | 0 | 1 | 42 | nucleobase metabolic process |
| GO:0035194 | 0.374 | 2.195 | 0 | 1 | 42 | posttranscriptional gene silencing by RNA |
| GO:0002237 | 0.374 | 2.195 | 0 | 1 | 42 | response to molecule of bacterial origin |
| GO:0046847 | 0.374 | 2.195 | 0 | 1 | 42 | filopodium assembly |
| GO:0016441 | 0.374 | 2.195 | 0 | 1 | 42 | posttranscriptional gene silencing |
| GO:0044270 | 0.378 | 1.256 | 4 | 5 | 365 | cellular nitrogen compound catabolic process |
| GO:0006790 | 0.380 | 1.541 | 1 | 2 | 119 | sulfur compound metabolic process |
| GO:0045860 | 0.380 | 1.541 | 1 | 2 | 119 | positive regulation of protein kinase activity |
| GO:0007611 | 0.380 | 1.541 | 1 | 2 | 119 | learning or memory |
| GO:0019438 | 0.381 | 2.142 | 0 | 1 | 43 | aromatic compound biosynthetic process |
| GO:0032409 | 0.381 | 2.142 | 0 | 1 | 43 | regulation of transporter activity |
| GO:0006661 | 0.381 | 2.142 | 0 | 1 | 43 | phosphatidylinositol biosynthetic process |
| GO:0009142 | 0.381 | 2.142 | 0 | 1 | 43 | nucleoside triphosphate biosynthetic process |
| GO:0001838 | 0.381 | 2.142 | 0 | 1 | 43 | embryonic epithelial tube formation |
| GO:0022412 | 0.381 | 2.142 | 0 | 1 | 43 | cellular process involved in reproduction in multicellular organism |
| GO:0022407 | 0.381 | 2.142 | 0 | 1 | 43 | regulation of cell-cell adhesion |
| GO:0072175 | 0.381 | 2.142 | 0 | 1 | 43 | epithelial tube formation |
| GO:0008016 | 0.381 | 2.142 | 0 | 1 | 43 | regulation of heart contraction |
| GO:0042462 | 0.384 | 1.528 | 1 | 2 | 120 | eye photoreceptor cell development |
| GO:0050776 | 0.384 | 1.528 | 1 | 2 | 120 | regulation of immune response |
| GO:0042060 | 0.384 | 1.367 | 2 | 3 | 201 | wound healing |
| GO:0045859 | 0.387 | 1.360 | 2 | 3 | 202 | regulation of protein kinase activity |
| GO:0034976 | 0.388 | 2.092 | 0 | 1 | 44 | response to endoplasmic reticulum stress |
| GO:0050865 | 0.388 | 2.092 | 0 | 1 | 44 | regulation of cell activation |
| GO:0009156 | 0.388 | 2.092 | 0 | 1 | 44 | ribonucleoside monophosphate biosynthetic process |
| GO:0030073 | 0.388 | 2.092 | 0 | 1 | 44 | insulin secretion |
| GO:0007034 | 0.388 | 2.092 | 0 | 1 | 44 | vacuolar transport |
| GO:0035151 | 0.388 | 2.092 | 0 | 1 | 44 | regulation of tube size, open tracheal system |
| GO:0007368 | 0.388 | 2.092 | 0 | 1 | 44 | determination of left/right symmetry |
| GO:0007304 | 0.388 | 2.092 | 0 | 1 | 44 | chorion-containing eggshell formation |
| GO:0051017 | 0.388 | 2.092 | 0 | 1 | 44 | actin filament bundle assembly |
| GO:0051224 | 0.388 | 2.092 | 0 | 1 | 44 | negative regulation of protein transport |
| GO:0008585 | 0.388 | 2.092 | 0 | 1 | 44 | female gonad development |
| GO:0040029 | 0.388 | 1.514 | 1 | 2 | 121 | regulation of gene expression, epigenetic |
| GO:0097285 | 0.388 | 1.514 | 1 | 2 | 121 | cell-type specific apoptotic process |
| GO:0050890 | 0.392 | 1.502 | 1 | 2 | 122 | cognition |
| GO:0071900 | 0.392 | 1.502 | 1 | 2 | 122 | regulation of protein serine/threonine kinase activity |
| GO:2000026 | 0.392 | 1.184 | 6 | 7 | 542 | regulation of multicellular organismal development |
| GO:0006892 | 0.394 | 2.044 | 0 | 1 | 45 | post-Golgi vesicle-mediated transport |
| GO:0019722 | 0.394 | 2.044 | 0 | 1 | 45 | calcium-mediated signaling |
| GO:0001704 | 0.394 | 2.044 | 0 | 1 | 45 | formation of primary germ layer |
| GO:0009124 | 0.394 | 2.044 | 0 | 1 | 45 | nucleoside monophosphate biosynthetic process |
| GO:0045814 | 0.394 | 2.044 | 0 | 1 | 45 | negative regulation of gene expression, epigenetic |
| GO:1901264 | 0.394 | 2.044 | 0 | 1 | 45 | carbohydrate derivative transport |
| GO:0010921 | 0.394 | 2.044 | 0 | 1 | 45 | regulation of phosphatase activity |
| GO:0060041 | 0.394 | 2.044 | 0 | 1 | 45 | retina development in camera-type eye |
| GO:0033674 | 0.396 | 1.489 | 1 | 2 | 123 | positive regulation of kinase activity |
| GO:0016568 | 0.400 | 1.262 | 3 | 4 | 290 | chromatin modification |
| GO:0006289 | 0.401 | 1.999 | 1 | 1 | 46 | nucleotide-excision repair |
| GO:0009161 | 0.401 | 1.999 | 1 | 1 | 46 | ribonucleoside monophosphate metabolic process |
| GO:0061448 | 0.401 | 1.999 | 1 | 1 | 46 | connective tissue development |
| GO:0051480 | 0.401 | 1.999 | 1 | 1 | 46 | cytosolic calcium ion homeostasis |
| GO:0010741 | 0.401 | 1.999 | 1 | 1 | 46 | negative regulation of intracellular protein kinase cascade |
| GO:0065004 | 0.401 | 1.999 | 1 | 1 | 46 | protein-DNA complex assembly |
| GO:0031572 | 0.401 | 1.999 | 1 | 1 | 46 | G2/M transition DNA damage checkpoint |
| GO:0031576 | 0.401 | 1.999 | 1 | 1 | 46 | G2/M transition checkpoint |
| GO:0031667 | 0.402 | 1.326 | 2 | 3 | 207 | response to nutrient levels |
| GO:0007596 | 0.404 | 1.465 | 1 | 2 | 125 | blood coagulation |
| GO:0001736 | 0.408 | 1.453 | 1 | 2 | 126 | establishment of planar polarity |
| GO:0007164 | 0.408 | 1.453 | 1 | 2 | 126 | establishment of tissue polarity |
| GO:0051347 | 0.408 | 1.453 | 1 | 2 | 126 | positive regulation of transferase activity |
| GO:0034097 | 0.408 | 1.453 | 1 | 2 | 126 | response to cytokine stimulus |
| GO:0042446 | 0.408 | 1.955 | 1 | 1 | 47 | hormone biosynthetic process |
| GO:0009451 | 0.408 | 1.955 | 1 | 1 | 47 | RNA modification |
| GO:0009123 | 0.408 | 1.955 | 1 | 1 | 47 | nucleoside monophosphate metabolic process |
| GO:0035317 | 0.408 | 1.955 | 1 | 1 | 47 | imaginal disc-derived wing hair organization |
| GO:0035148 | 0.408 | 1.955 | 1 | 1 | 47 | tube formation |
| GO:0043406 | 0.408 | 1.955 | 1 | 1 | 47 | positive regulation of MAP kinase activity |
| GO:0030703 | 0.408 | 1.955 | 1 | 1 | 47 | eggshell formation |
| GO:0008347 | 0.408 | 1.955 | 1 | 1 | 47 | glial cell migration |
| GO:0051726 | 0.414 | 1.200 | 4 | 5 | 381 | regulation of cell cycle |
| GO:0040014 | 0.414 | 1.913 | 1 | 1 | 48 | regulation of multicellular organism growth |
| GO:0001649 | 0.414 | 1.913 | 1 | 1 | 48 | osteoblast differentiation |
| GO:0048167 | 0.414 | 1.913 | 1 | 1 | 48 | regulation of synaptic plasticity |
| GO:0030324 | 0.414 | 1.913 | 1 | 1 | 48 | lung development |
| GO:0048872 | 0.414 | 1.913 | 1 | 1 | 48 | homeostasis of number of cells |
| GO:0042127 | 0.416 | 1.197 | 4 | 5 | 382 | regulation of cell proliferation |
| GO:0009991 | 0.417 | 1.293 | 2 | 3 | 212 | response to extracellular stimulus |
| GO:0043549 | 0.417 | 1.293 | 2 | 3 | 212 | regulation of kinase activity |
| GO:0044085 | 0.418 | 1.099 | 13 | 14 | 1169 | cellular component biogenesis |
| GO:0060562 | 0.419 | 1.418 | 1 | 2 | 129 | epithelial tube morphogenesis |
| GO:0006720 | 0.421 | 1.873 | 1 | 1 | 49 | isoprenoid metabolic process |
| GO:0050773 | 0.421 | 1.873 | 1 | 1 | 49 | regulation of dendrite development |
| GO:0045197 | 0.421 | 1.873 | 1 | 1 | 49 | establishment or maintenance of epithelial cell apical/basal polarity |
| GO:0035316 | 0.421 | 1.873 | 1 | 1 | 49 | non-sensory hair organization |
| GO:0035222 | 0.421 | 1.873 | 1 | 1 | 49 | wing disc pattern formation |
| GO:0030574 | 0.421 | 1.873 | 1 | 1 | 49 | collagen catabolic process |
| GO:0051648 | 0.421 | 1.873 | 1 | 1 | 49 | vesicle localization |
| GO:0072593 | 0.421 | 1.873 | 1 | 1 | 49 | reactive oxygen species metabolic process |
| GO:0051223 | 0.423 | 1.406 | 1 | 2 | 130 | regulation of protein transport |
| GO:0001745 | 0.423 | 1.281 | 2 | 3 | 214 | compound eye morphogenesis |
| GO:0042325 | 0.425 | 1.218 | 3 | 4 | 300 | regulation of phosphorylation |
| GO:0022613 | 0.426 | 1.275 | 2 | 3 | 215 | ribonucleoprotein complex biogenesis |
| GO:0009617 | 0.427 | 1.395 | 1 | 2 | 131 | response to bacterium |
| GO:0000075 | 0.427 | 1.395 | 1 | 2 | 131 | cell cycle checkpoint |
| GO:0009799 | 0.427 | 1.834 | 1 | 1 | 50 | specification of symmetry |
| GO:0009855 | 0.427 | 1.834 | 1 | 1 | 50 | determination of bilateral symmetry |
| GO:0030323 | 0.427 | 1.834 | 1 | 1 | 50 | respiratory tube development |
| GO:0030278 | 0.427 | 1.834 | 1 | 1 | 50 | regulation of ossification |
| GO:0000082 | 0.427 | 1.834 | 1 | 1 | 50 | G1/S transition of mitotic cell cycle |
| GO:0072659 | 0.427 | 1.834 | 1 | 1 | 50 | protein localization to plasma membrane |
| GO:0016339 | 0.427 | 1.834 | 1 | 1 | 50 | calcium-dependent cell-cell adhesion |
| GO:0009143 | 0.428 | 1.214 | 3 | 4 | 301 | nucleoside triphosphate catabolic process |
| GO:0009146 | 0.428 | 1.214 | 3 | 4 | 301 | purine nucleoside triphosphate catabolic process |
| GO:0009203 | 0.428 | 1.214 | 3 | 4 | 301 | ribonucleoside triphosphate catabolic process |
| GO:0009207 | 0.428 | 1.214 | 3 | 4 | 301 | purine ribonucleoside triphosphate catabolic process |
| GO:0009968 | 0.428 | 1.214 | 3 | 4 | 301 | negative regulation of signal transduction |
| GO:0051338 | 0.429 | 1.269 | 2 | 3 | 216 | regulation of transferase activity |
| GO:0044267 | 0.429 | 1.077 | 18 | 19 | 1621 | cellular protein metabolic process |
| GO:0051046 | 0.431 | 1.384 | 1 | 2 | 132 | regulation of secretion |
| GO:0009154 | 0.433 | 1.206 | 3 | 4 | 303 | purine ribonucleotide catabolic process |
| GO:0009261 | 0.433 | 1.206 | 3 | 4 | 303 | ribonucleotide catabolic process |
| GO:0006313 | 0.434 | 1.798 | 1 | 1 | 51 | transposition, DNA-mediated |
| GO:0006479 | 0.434 | 1.798 | 1 | 1 | 51 | protein methylation |
| GO:0045785 | 0.434 | 1.798 | 1 | 1 | 51 | positive regulation of cell adhesion |
| GO:0030072 | 0.434 | 1.798 | 1 | 1 | 51 | peptide hormone secretion |
| GO:0022404 | 0.434 | 1.798 | 1 | 1 | 51 | molting cycle process |
| GO:0035315 | 0.434 | 1.798 | 1 | 1 | 51 | hair cell differentiation |
| GO:0046545 | 0.434 | 1.798 | 1 | 1 | 51 | development of primary female sexual characteristics |
| GO:0044243 | 0.434 | 1.798 | 1 | 1 | 51 | multicellular organismal catabolic process |
| GO:0008213 | 0.434 | 1.798 | 1 | 1 | 51 | protein alkylation |
| GO:0045786 | 0.435 | 1.256 | 2 | 3 | 218 | negative regulation of cell cycle |
| GO:0042461 | 0.438 | 1.363 | 1 | 2 | 134 | photoreceptor cell development |
| GO:0050817 | 0.438 | 1.363 | 1 | 2 | 134 | coagulation |
| GO:0009101 | 0.438 | 1.363 | 1 | 2 | 134 | glycoprotein biosynthetic process |
| GO:0007599 | 0.438 | 1.363 | 1 | 2 | 134 | hemostasis |
| GO:0005976 | 0.438 | 1.363 | 1 | 2 | 134 | polysaccharide metabolic process |
| GO:0008283 | 0.439 | 1.128 | 6 | 7 | 567 | cell proliferation |
| GO:0019725 | 0.440 | 1.193 | 3 | 4 | 306 | cellular homeostasis |
| GO:0021915 | 0.440 | 1.762 | 1 | 1 | 52 | neural tube development |
| GO:0035088 | 0.440 | 1.762 | 1 | 1 | 52 | establishment or maintenance of apical/basal cell polarity |
| GO:0007314 | 0.440 | 1.762 | 1 | 1 | 52 | oocyte anterior/posterior axis specification |
| GO:0048562 | 0.440 | 1.762 | 1 | 1 | 52 | embryonic organ morphogenesis |
| GO:0009790 | 0.441 | 1.106 | 8 | 9 | 744 | embryo development |
| GO:0031987 | 0.446 | 1.728 | 1 | 1 | 53 | locomotion involved in locomotory behavior |
| GO:0016202 | 0.446 | 1.728 | 1 | 1 | 53 | regulation of striated muscle tissue development |
| GO:0009581 | 0.446 | 1.728 | 1 | 1 | 53 | detection of external stimulus |
| GO:0009582 | 0.446 | 1.728 | 1 | 1 | 53 | detection of abiotic stimulus |
| GO:0061245 | 0.446 | 1.728 | 1 | 1 | 53 | establishment or maintenance of bipolar cell polarity |
| GO:0048634 | 0.446 | 1.728 | 1 | 1 | 53 | regulation of muscle organ development |
| GO:0007272 | 0.446 | 1.728 | 1 | 1 | 53 | ensheathment of neurons |
| GO:0002440 | 0.446 | 1.728 | 1 | 1 | 53 | production of molecular mediator of immune response |
| GO:0090132 | 0.446 | 1.728 | 1 | 1 | 53 | epithelium migration |
| GO:0008366 | 0.446 | 1.728 | 1 | 1 | 53 | axon ensheathment |
| GO:0008358 | 0.446 | 1.728 | 1 | 1 | 53 | maternal determination of anterior/posterior axis, embryo |
| GO:0010631 | 0.446 | 1.728 | 1 | 1 | 53 | epithelial cell migration |
| GO:0009611 | 0.447 | 1.181 | 3 | 4 | 309 | response to wounding |
| GO:0002165 | 0.447 | 1.181 | 3 | 4 | 309 | instar larval or pupal development |
| GO:0071824 | 0.452 | 1.695 | 1 | 1 | 54 | protein-DNA complex subunit organization |
| GO:0031344 | 0.453 | 1.322 | 2 | 2 | 138 | regulation of cell projection organization |
| GO:0044248 | 0.453 | 1.073 | 12 | 13 | 1107 | cellular catabolic process |
| GO:0043412 | 0.455 | 1.069 | 13 | 14 | 1197 | macromolecule modification |
| GO:0006469 | 0.459 | 1.664 | 1 | 1 | 55 | negative regulation of protein kinase activity |
| GO:0019915 | 0.459 | 1.664 | 1 | 1 | 55 | lipid storage |
| GO:0007478 | 0.459 | 1.664 | 1 | 1 | 55 | leg disc morphogenesis |
| GO:0002790 | 0.459 | 1.664 | 1 | 1 | 55 | peptide secretion |
| GO:0090130 | 0.459 | 1.664 | 1 | 1 | 55 | tissue migration |
| GO:0046620 | 0.459 | 1.664 | 1 | 1 | 55 | regulation of organ growth |
| GO:0051704 | 0.459 | 1.104 | 6 | 7 | 578 | multi-organism process |
| GO:0048598 | 0.460 | 1.161 | 3 | 4 | 314 | embryonic morphogenesis |
| GO:0070201 | 0.461 | 1.303 | 2 | 2 | 140 | regulation of establishment of protein localization |
| GO:0006897 | 0.462 | 1.157 | 3 | 4 | 315 | endocytosis |
| GO:0071156 | 0.464 | 1.293 | 2 | 2 | 141 | regulation of cell cycle arrest |
| GO:0045471 | 0.465 | 1.633 | 1 | 1 | 56 | response to ethanol |
| GO:0001894 | 0.465 | 1.633 | 1 | 1 | 56 | tissue homeostasis |
| GO:0032963 | 0.465 | 1.633 | 1 | 1 | 56 | collagen metabolic process |
| GO:0035264 | 0.465 | 1.633 | 1 | 1 | 56 | multicellular organism growth |
| GO:0048859 | 0.465 | 1.633 | 1 | 1 | 56 | formation of anatomical boundary |
| GO:0030522 | 0.465 | 1.633 | 1 | 1 | 56 | intracellular receptor mediated signaling pathway |
| GO:0031047 | 0.465 | 1.633 | 1 | 1 | 56 | gene silencing by RNA |
| GO:0044264 | 0.465 | 1.633 | 1 | 1 | 56 | cellular polysaccharide metabolic process |
| GO:0006955 | 0.465 | 1.153 | 3 | 4 | 316 | immune response |
| GO:0032870 | 0.468 | 1.284 | 2 | 2 | 142 | cellular response to hormone stimulus |
| GO:1901293 | 0.468 | 1.284 | 2 | 2 | 142 | nucleoside phosphate biosynthetic process |
| GO:0044262 | 0.468 | 1.284 | 2 | 2 | 142 | cellular carbohydrate metabolic process |
| GO:0007155 | 0.469 | 1.104 | 5 | 6 | 495 | cell adhesion |
| GO:0009152 | 0.471 | 1.604 | 1 | 1 | 57 | purine ribonucleotide biosynthetic process |
| GO:0097305 | 0.471 | 1.604 | 1 | 1 | 57 | response to alcohol |
| GO:0030301 | 0.471 | 1.604 | 1 | 1 | 57 | cholesterol transport |
| GO:0048806 | 0.471 | 1.604 | 1 | 1 | 57 | genitalia development |
| GO:0046328 | 0.471 | 1.604 | 1 | 1 | 57 | regulation of JNK cascade |
| GO:0046879 | 0.471 | 1.604 | 1 | 1 | 57 | hormone secretion |
| GO:0046660 | 0.471 | 1.604 | 1 | 1 | 57 | female sex differentiation |
| GO:0016050 | 0.471 | 1.604 | 1 | 1 | 57 | vesicle organization |
| GO:0060047 | 0.471 | 1.604 | 1 | 1 | 57 | heart contraction |
| GO:0030163 | 0.474 | 1.138 | 4 | 4 | 320 | protein catabolic process |
| GO:0055080 | 0.475 | 1.266 | 2 | 2 | 144 | cation homeostasis |
| GO:0006418 | 0.476 | 1.575 | 1 | 1 | 58 | tRNA aminoacylation for protein translation |
| GO:0043038 | 0.476 | 1.575 | 1 | 1 | 58 | amino acid activation |
| GO:0043039 | 0.476 | 1.575 | 1 | 1 | 58 | tRNA aminoacylation |
| GO:0007349 | 0.476 | 1.575 | 1 | 1 | 58 | cellularization |
| GO:0051098 | 0.476 | 1.575 | 1 | 1 | 58 | regulation of binding |
| GO:0015833 | 0.476 | 1.575 | 1 | 1 | 58 | peptide transport |
| GO:0006873 | 0.478 | 1.172 | 3 | 3 | 233 | cellular ion homeostasis |
| GO:0002376 | 0.479 | 1.082 | 7 | 7 | 589 | immune system process |
| GO:0006325 | 0.479 | 1.131 | 4 | 4 | 322 | chromatin organization |
| GO:0007600 | 0.481 | 1.167 | 3 | 3 | 234 | sensory perception |
| GO:0034622 | 0.482 | 1.127 | 4 | 4 | 323 | cellular macromolecular complex assembly |
| GO:0007464 | 0.482 | 1.548 | 1 | 1 | 59 | R3/R4 cell fate commitment |
| GO:0009914 | 0.482 | 1.548 | 1 | 1 | 59 | hormone transport |
| GO:0043297 | 0.482 | 1.548 | 1 | 1 | 59 | apical junction assembly |
| GO:0033673 | 0.482 | 1.548 | 1 | 1 | 59 | negative regulation of kinase activity |
| GO:0003015 | 0.482 | 1.548 | 1 | 1 | 59 | heart process |
| GO:0031123 | 0.482 | 1.548 | 1 | 1 | 59 | RNA 3'-end processing |
| GO:0016333 | 0.482 | 1.548 | 1 | 1 | 59 | morphogenesis of follicular epithelium |
| GO:0001738 | 0.483 | 1.248 | 2 | 2 | 146 | morphogenesis of a polarized epithelium |
| GO:0022610 | 0.485 | 1.085 | 6 | 6 | 503 | biological adhesion |
| GO:0042787 | 0.488 | 1.522 | 1 | 1 | 60 | protein ubiquitination involved in ubiquitin-dependent protein catabolic process |
| GO:0048056 | 0.488 | 1.522 | 1 | 1 | 60 | R3/R4 cell differentiation |
| GO:0001708 | 0.488 | 1.522 | 1 | 1 | 60 | cell fate specification |
| GO:0032872 | 0.488 | 1.522 | 1 | 1 | 60 | regulation of stress-activated MAPK cascade |
| GO:0035218 | 0.488 | 1.522 | 1 | 1 | 60 | leg disc development |
| GO:0061138 | 0.488 | 1.522 | 1 | 1 | 60 | morphogenesis of a branching epithelium |
| GO:0051348 | 0.488 | 1.522 | 1 | 1 | 60 | negative regulation of transferase activity |
| GO:0007586 | 0.488 | 1.522 | 1 | 1 | 60 | digestion |
| GO:0008344 | 0.488 | 1.522 | 1 | 1 | 60 | adult locomotory behavior |
| GO:0070302 | 0.488 | 1.522 | 1 | 1 | 60 | regulation of stress-activated protein kinase signaling cascade |
| GO:0044259 | 0.488 | 1.522 | 1 | 1 | 60 | multicellular organismal macromolecule metabolic process |
| GO:0002252 | 0.490 | 1.230 | 2 | 2 | 148 | immune effector process |
| GO:0002682 | 0.490 | 1.152 | 3 | 3 | 237 | regulation of immune system process |
| GO:0031401 | 0.490 | 1.152 | 3 | 3 | 237 | positive regulation of protein modification process |
| GO:0090407 | 0.493 | 1.222 | 2 | 2 | 149 | organophosphate biosynthetic process |
| GO:0045467 | 0.494 | 1.496 | 1 | 1 | 61 | R7 cell development |
| GO:0035214 | 0.494 | 1.496 | 1 | 1 | 61 | eye-antennal disc development |
| GO:0051384 | 0.494 | 1.496 | 1 | 1 | 61 | response to glucocorticoid stimulus |
| GO:0007268 | 0.494 | 1.109 | 4 | 4 | 328 | synaptic transmission |
| GO:0007447 | 0.499 | 1.471 | 1 | 1 | 62 | imaginal disc pattern formation |
| GO:0071902 | 0.499 | 1.471 | 1 | 1 | 62 | positive regulation of protein serine/threonine kinase activity |
| GO:0007612 | 0.499 | 1.471 | 1 | 1 | 62 | learning |
| GO:0007605 | 0.499 | 1.471 | 1 | 1 | 62 | sensory perception of sound |
| GO:0015918 | 0.499 | 1.471 | 1 | 1 | 62 | sterol transport |
| GO:0044236 | 0.499 | 1.471 | 1 | 1 | 62 | multicellular organismal metabolic process |
| GO:0016199 | 0.499 | 1.471 | 1 | 1 | 62 | axon midline choice point recognition |
| GO:0034654 | 0.500 | 1.205 | 2 | 2 | 151 | nucleobase-containing compound biosynthetic process |
| GO:0055082 | 0.501 | 1.132 | 3 | 3 | 241 | cellular chemical homeostasis |
| GO:0001775 | 0.504 | 1.197 | 2 | 2 | 152 | cell activation |
| GO:0071363 | 0.505 | 1.447 | 1 | 1 | 63 | cellular response to growth factor stimulus |
| GO:0048754 | 0.505 | 1.447 | 1 | 1 | 63 | branching morphogenesis of a tube |
| GO:0016198 | 0.505 | 1.447 | 1 | 1 | 63 | axon choice point recognition |
| GO:0006511 | 0.507 | 1.122 | 3 | 3 | 243 | ubiquitin-dependent protein catabolic process |
| GO:0006812 | 0.509 | 1.117 | 3 | 3 | 244 | cation transport |
| GO:0019941 | 0.509 | 1.117 | 3 | 3 | 244 | modification-dependent protein catabolic process |
| GO:0031960 | 0.510 | 1.424 | 1 | 1 | 64 | response to corticosteroid stimulus |
| GO:0042303 | 0.510 | 1.424 | 1 | 1 | 64 | molting cycle |
| GO:0042157 | 0.510 | 1.424 | 1 | 1 | 64 | lipoprotein metabolic process |
| GO:0046488 | 0.510 | 1.424 | 1 | 1 | 64 | phosphatidylinositol metabolic process |
| GO:0070848 | 0.510 | 1.424 | 1 | 1 | 64 | response to growth factor stimulus |
| GO:0023061 | 0.511 | 1.181 | 2 | 2 | 154 | signal release |
| GO:0003001 | 0.511 | 1.181 | 2 | 2 | 154 | generation of a signal involved in cell-cell signaling |
| GO:0051301 | 0.511 | 1.066 | 5 | 5 | 426 | cell division |
| GO:0006310 | 0.512 | 1.112 | 3 | 3 | 245 | DNA recombination |
| GO:0043086 | 0.514 | 1.173 | 2 | 2 | 155 | negative regulation of catalytic activity |
| GO:0002697 | 0.516 | 1.402 | 1 | 1 | 65 | regulation of immune effector process |
| GO:0006026 | 0.516 | 1.402 | 1 | 1 | 65 | aminoglycan catabolic process |
| GO:0070647 | 0.518 | 1.074 | 4 | 4 | 338 | protein modification by small protein conjugation or removal |
| GO:0051129 | 0.518 | 1.165 | 2 | 2 | 156 | negative regulation of cellular component organization |
| GO:0016311 | 0.518 | 1.165 | 2 | 2 | 156 | dephosphorylation |
| GO:0015074 | 0.521 | 1.158 | 2 | 2 | 157 | DNA integration |
| GO:0048749 | 0.523 | 1.094 | 3 | 3 | 249 | compound eye development |
| GO:0043632 | 0.526 | 1.089 | 3 | 3 | 250 | modification-dependent macromolecule catabolic process |
| GO:0009913 | 0.527 | 1.359 | 1 | 1 | 67 | epidermal cell differentiation |
| GO:0007568 | 0.528 | 1.142 | 2 | 2 | 159 | aging |
| GO:0044271 | 0.531 | 1.080 | 3 | 3 | 252 | cellular nitrogen compound biosynthetic process |
| GO:0042742 | 0.532 | 1.339 | 1 | 1 | 68 | defense response to bacterium |
| GO:0008045 | 0.532 | 1.339 | 1 | 1 | 68 | motor axon guidance |
| GO:0071843 | 0.534 | 1.076 | 3 | 3 | 253 | cellular component biogenesis at cellular level |
| GO:0014070 | 0.537 | 1.319 | 1 | 1 | 69 | response to organic cyclic compound |
| GO:1901214 | 0.537 | 1.319 | 1 | 1 | 69 | regulation of neuron death |
| GO:0008593 | 0.537 | 1.319 | 1 | 1 | 69 | regulation of Notch signaling pathway |
| GO:0050878 | 0.538 | 1.121 | 2 | 2 | 162 | regulation of body fluid levels |
| GO:0050801 | 0.539 | 1.067 | 3 | 3 | 255 | ion homeostasis |
| GO:0050768 | 0.542 | 1.300 | 1 | 1 | 70 | negative regulation of neurogenesis |
| GO:0032196 | 0.542 | 1.300 | 1 | 1 | 70 | transposition |
| GO:0009260 | 0.542 | 1.300 | 1 | 1 | 70 | ribonucleotide biosynthetic process |
| GO:0001933 | 0.542 | 1.300 | 1 | 1 | 70 | negative regulation of protein phosphorylation |
| GO:0060341 | 0.545 | 1.058 | 3 | 3 | 257 | regulation of cellular localization |
| GO:0065008 | 0.547 | 0.998 | 14 | 14 | 1268 | regulation of biological quality |
| GO:0043010 | 0.547 | 1.281 | 1 | 1 | 71 | camera-type eye development |
| GO:0007043 | 0.547 | 1.281 | 1 | 1 | 71 | cell-cell junction assembly |
| GO:0007009 | 0.547 | 1.281 | 1 | 1 | 71 | plasma membrane organization |
| GO:0051346 | 0.547 | 1.281 | 1 | 1 | 71 | negative regulation of hydrolase activity |
| GO:0001934 | 0.551 | 1.093 | 2 | 2 | 166 | positive regulation of protein phosphorylation |
| GO:0051493 | 0.551 | 1.093 | 2 | 2 | 166 | regulation of cytoskeleton organization |
| GO:0001763 | 0.552 | 1.263 | 1 | 1 | 72 | morphogenesis of a branching structure |
| GO:0009408 | 0.552 | 1.263 | 1 | 1 | 72 | response to heat |
| GO:0019932 | 0.552 | 1.263 | 1 | 1 | 72 | second-messenger-mediated signaling |
| GO:0035150 | 0.552 | 1.263 | 1 | 1 | 72 | regulation of tube size |
| GO:0051603 | 0.553 | 1.046 | 3 | 3 | 260 | proteolysis involved in cellular protein catabolic process |
| GO:0009056 | 0.553 | 0.993 | 15 | 15 | 1364 | catabolic process |
| GO:0050673 | 0.557 | 1.245 | 1 | 1 | 73 | epithelial cell proliferation |
| GO:0030010 | 0.557 | 1.245 | 1 | 1 | 73 | establishment of cell polarity |
| GO:0045995 | 0.557 | 1.245 | 1 | 1 | 73 | regulation of embryonic development |
| GO:0000272 | 0.557 | 1.245 | 1 | 1 | 73 | polysaccharide catabolic process |
| GO:0060070 | 0.557 | 1.245 | 1 | 1 | 73 | canonical Wnt receptor signaling pathway |
| GO:0016049 | 0.561 | 1.073 | 2 | 2 | 169 | cell growth |
| GO:0019228 | 0.562 | 1.228 | 1 | 1 | 74 | regulation of action potential in neuron |
| GO:0031023 | 0.562 | 1.228 | 1 | 1 | 74 | microtubule organizing center organization |
| GO:0003012 | 0.562 | 1.228 | 1 | 1 | 74 | muscle system process |
| GO:0009967 | 0.563 | 1.029 | 3 | 3 | 264 | positive regulation of signal transduction |
| GO:0044257 | 0.563 | 1.029 | 3 | 3 | 264 | cellular protein catabolic process |
| GO:0046034 | 0.564 | 1.066 | 2 | 2 | 170 | ATP metabolic process |
| GO:0051270 | 0.564 | 1.066 | 2 | 2 | 170 | regulation of cellular component movement |
| GO:0006974 | 0.565 | 1.000 | 5 | 5 | 452 | response to DNA damage stimulus |
| GO:0048707 | 0.566 | 1.025 | 3 | 3 | 265 | instar larval or pupal morphogenesis |
| GO:0001667 | 0.567 | 1.211 | 1 | 1 | 75 | ameboidal cell migration |
| GO:0007613 | 0.567 | 1.211 | 1 | 1 | 75 | memory |
| GO:0009892 | 0.569 | 0.988 | 7 | 7 | 640 | negative regulation of metabolic process |
| GO:0007391 | 0.572 | 1.195 | 1 | 1 | 76 | dorsal closure |
| GO:0007160 | 0.572 | 1.195 | 1 | 1 | 76 | cell-matrix adhesion |
| GO:0051093 | 0.576 | 1.009 | 3 | 3 | 269 | negative regulation of developmental process |
| GO:0031324 | 0.576 | 0.984 | 6 | 6 | 551 | negative regulation of cellular metabolic process |
| GO:0006814 | 0.577 | 1.179 | 1 | 1 | 77 | sodium ion transport |
| GO:0035265 | 0.577 | 1.179 | 1 | 1 | 77 | organ growth |
| GO:0022900 | 0.577 | 1.179 | 1 | 1 | 77 | electron transport chain |
| GO:0009886 | 0.578 | 1.005 | 3 | 3 | 270 | post-embryonic morphogenesis |
| GO:0032880 | 0.580 | 1.035 | 2 | 2 | 175 | regulation of protein localization |
| GO:0016055 | 0.580 | 1.035 | 2 | 2 | 175 | Wnt receptor signaling pathway |
| GO:0055001 | 0.580 | 1.035 | 2 | 2 | 175 | muscle cell development |
| GO:0032270 | 0.581 | 1.001 | 3 | 3 | 271 | positive regulation of cellular protein metabolic process |
| GO:0019748 | 0.582 | 1.163 | 1 | 1 | 78 | secondary metabolic process |
| GO:0051090 | 0.582 | 1.163 | 1 | 1 | 78 | regulation of sequence-specific DNA binding transcription factor activity |
| GO:0007179 | 0.582 | 1.163 | 1 | 1 | 78 | transforming growth factor beta receptor signaling pathway |
| GO:0007552 | 0.586 | 0.994 | 3 | 3 | 273 | metamorphosis |
| GO:0070997 | 0.586 | 1.148 | 1 | 1 | 79 | neuron death |
| GO:0042706 | 0.586 | 1.148 | 1 | 1 | 79 | eye photoreceptor cell fate commitment |
| GO:0001752 | 0.586 | 1.148 | 1 | 1 | 79 | compound eye photoreceptor fate commitment |
| GO:0050905 | 0.586 | 1.148 | 1 | 1 | 79 | neuromuscular process |
| GO:0007093 | 0.586 | 1.148 | 1 | 1 | 79 | mitotic cell cycle checkpoint |
| GO:0007351 | 0.586 | 1.148 | 1 | 1 | 79 | tripartite regional subdivision |
| GO:0007528 | 0.586 | 1.148 | 1 | 1 | 79 | neuromuscular junction development |
| GO:0000077 | 0.586 | 1.148 | 1 | 1 | 79 | DNA damage checkpoint |
| GO:0046552 | 0.586 | 1.148 | 1 | 1 | 79 | photoreceptor cell fate commitment |
| GO:0008595 | 0.586 | 1.148 | 1 | 1 | 79 | anterior/posterior axis specification, embryo |
| GO:0042327 | 0.587 | 1.023 | 2 | 2 | 177 | positive regulation of phosphorylation |
| GO:0007186 | 0.587 | 1.023 | 2 | 2 | 177 | G-protein coupled receptor signaling pathway |
| GO:0007010 | 0.587 | 0.970 | 7 | 7 | 651 | cytoskeleton organization |
| GO:0001932 | 0.589 | 0.990 | 3 | 3 | 274 | regulation of protein phosphorylation |
| GO:0043065 | 0.590 | 1.017 | 2 | 2 | 178 | positive regulation of apoptotic process |
| GO:0042440 | 0.591 | 1.134 | 1 | 1 | 80 | pigment metabolic process |
| GO:0045466 | 0.591 | 1.134 | 1 | 1 | 80 | R7 cell differentiation |
| GO:0032844 | 0.591 | 1.134 | 1 | 1 | 80 | regulation of homeostatic process |
| GO:0007254 | 0.591 | 1.134 | 1 | 1 | 80 | JNK cascade |
| GO:0072657 | 0.591 | 1.134 | 1 | 1 | 80 | protein localization to membrane |
| GO:0006164 | 0.595 | 1.119 | 1 | 1 | 81 | purine nucleotide biosynthetic process |
| GO:0048729 | 0.597 | 0.968 | 4 | 4 | 373 | tissue morphogenesis |
| GO:0006468 | 0.599 | 0.965 | 4 | 4 | 374 | protein phosphorylation |
| GO:0006575 | 0.600 | 1.105 | 1 | 1 | 82 | cellular modified amino acid metabolic process |
| GO:0007156 | 0.600 | 1.105 | 1 | 1 | 82 | homophilic cell adhesion |
| GO:0008340 | 0.600 | 1.105 | 1 | 1 | 82 | determination of adult lifespan |
| GO:0006887 | 0.602 | 0.994 | 2 | 2 | 182 | exocytosis |
| GO:0009628 | 0.602 | 0.957 | 5 | 5 | 471 | response to abiotic stimulus |
| GO:0018193 | 0.603 | 0.968 | 3 | 3 | 280 | peptidyl-amino acid modification |
| GO:0001503 | 0.604 | 1.092 | 1 | 1 | 83 | ossification |
| GO:0030198 | 0.604 | 1.092 | 1 | 1 | 83 | extracellular matrix organization |
| GO:0000226 | 0.606 | 0.964 | 3 | 3 | 281 | microtubule cytoskeleton organization |
| GO:0071842 | 0.608 | 0.960 | 25 | 24 | 2236 | cellular component organization at cellular level |
| GO:0009063 | 0.609 | 1.078 | 1 | 1 | 84 | cellular amino acid catabolic process |
| GO:0019730 | 0.609 | 1.078 | 1 | 1 | 84 | antimicrobial humoral response |
| GO:0043414 | 0.609 | 1.078 | 1 | 1 | 84 | macromolecule methylation |
| GO:0051403 | 0.609 | 1.078 | 1 | 1 | 84 | stress-activated MAPK cascade |
| GO:0014706 | 0.611 | 0.977 | 2 | 2 | 185 | striated muscle tissue development |
| GO:0048741 | 0.613 | 1.065 | 1 | 1 | 85 | skeletal muscle fiber development |
| GO:0046777 | 0.613 | 1.065 | 1 | 1 | 85 | protein autophosphorylation |
| GO:0023056 | 0.615 | 0.950 | 3 | 3 | 285 | positive regulation of signaling |
| GO:0034621 | 0.616 | 0.944 | 4 | 4 | 382 | cellular macromolecular complex subunit organization |
| GO:0006470 | 0.618 | 1.053 | 1 | 1 | 86 | protein dephosphorylation |
| GO:0006261 | 0.618 | 1.053 | 1 | 1 | 86 | DNA-dependent DNA replication |
| GO:0045216 | 0.618 | 1.053 | 1 | 1 | 86 | cell-cell junction organization |
| GO:0007052 | 0.618 | 1.053 | 1 | 1 | 86 | mitotic spindle organization |
| GO:0031098 | 0.618 | 1.053 | 1 | 1 | 86 | stress-activated protein kinase signaling cascade |
| GO:0051247 | 0.618 | 0.946 | 3 | 3 | 286 | positive regulation of protein metabolic process |
| GO:0010647 | 0.618 | 0.946 | 3 | 3 | 286 | positive regulation of cell communication |
| GO:0008104 | 0.619 | 0.942 | 11 | 10 | 953 | protein localization |
| GO:0051052 | 0.622 | 1.040 | 1 | 1 | 87 | regulation of DNA metabolic process |
| GO:0031570 | 0.622 | 1.040 | 1 | 1 | 87 | DNA integrity checkpoint |
| GO:0051146 | 0.623 | 0.956 | 2 | 2 | 189 | striated muscle cell differentiation |
| GO:0032259 | 0.626 | 1.028 | 1 | 1 | 88 | methylation |
| GO:0008652 | 0.626 | 1.028 | 1 | 1 | 88 | cellular amino acid biosynthetic process |
| GO:0019226 | 0.628 | 0.929 | 4 | 4 | 388 | transmission of nerve impulse |
| GO:0060537 | 0.628 | 0.945 | 2 | 2 | 191 | muscle tissue development |
| GO:0010259 | 0.630 | 1.017 | 1 | 1 | 89 | multicellular organismal aging |
| GO:0043068 | 0.631 | 0.940 | 2 | 2 | 192 | positive regulation of programmed cell death |
| GO:0051050 | 0.631 | 0.940 | 2 | 2 | 192 | positive regulation of transport |
| GO:0006913 | 0.634 | 0.935 | 2 | 2 | 193 | nucleocytoplasmic transport |
| GO:1901137 | 0.634 | 0.935 | 2 | 2 | 193 | carbohydrate derivative biosynthetic process |
| GO:0010942 | 0.634 | 0.935 | 2 | 2 | 193 | positive regulation of cell death |
| GO:0048663 | 0.634 | 1.005 | 1 | 1 | 90 | neuron fate commitment |
| GO:0060429 | 0.636 | 0.918 | 4 | 4 | 392 | epithelium development |
| GO:0033036 | 0.638 | 0.930 | 13 | 12 | 1155 | macromolecule localization |
| GO:0001709 | 0.638 | 0.994 | 1 | 1 | 91 | cell fate determination |
| GO:0051169 | 0.640 | 0.925 | 2 | 2 | 195 | nuclear transport |
| GO:0007269 | 0.643 | 0.983 | 1 | 1 | 92 | neurotransmitter secretion |
| GO:0072522 | 0.643 | 0.983 | 1 | 1 | 92 | purine-containing compound biosynthetic process |
| GO:0009791 | 0.644 | 0.909 | 4 | 4 | 396 | post-embryonic development |
| GO:0001508 | 0.650 | 0.961 | 1 | 1 | 94 | regulation of action potential |
| GO:0043062 | 0.650 | 0.961 | 1 | 1 | 94 | extracellular structure organization |
| GO:0051051 | 0.654 | 0.951 | 1 | 1 | 95 | negative regulation of transport |
| GO:0010740 | 0.654 | 0.951 | 1 | 1 | 95 | positive regulation of intracellular protein kinase cascade |
| GO:0035637 | 0.656 | 0.894 | 4 | 4 | 402 | multicellular organismal signaling |
| GO:0006898 | 0.658 | 0.941 | 1 | 1 | 96 | receptor-mediated endocytosis |
| GO:0006917 | 0.658 | 0.941 | 1 | 1 | 96 | induction of apoptosis |
| GO:0000578 | 0.658 | 0.941 | 1 | 1 | 96 | embryonic axis specification |
| GO:0055085 | 0.659 | 0.888 | 3 | 3 | 304 | transmembrane transport |
| GO:0048565 | 0.662 | 0.931 | 1 | 1 | 97 | digestive tract development |
| GO:0001666 | 0.666 | 0.921 | 1 | 1 | 98 | response to hypoxia |
| GO:0036293 | 0.666 | 0.921 | 1 | 1 | 98 | response to decreased oxygen levels |
| GO:0070482 | 0.666 | 0.921 | 1 | 1 | 98 | response to oxygen levels |
| GO:0031589 | 0.666 | 0.921 | 1 | 1 | 98 | cell-substrate adhesion |
| GO:0040012 | 0.672 | 0.870 | 2 | 2 | 207 | regulation of locomotion |
| GO:0040017 | 0.673 | 0.902 | 1 | 1 | 100 | positive regulation of locomotion |
| GO:0032956 | 0.673 | 0.902 | 1 | 1 | 100 | regulation of actin cytoskeleton organization |
| GO:0048747 | 0.673 | 0.902 | 1 | 1 | 100 | muscle fiber development |
| GO:0043408 | 0.673 | 0.902 | 1 | 1 | 100 | regulation of MAPK cascade |
| GO:0016051 | 0.677 | 0.893 | 1 | 1 | 101 | carbohydrate biosynthetic process |
| GO:0060249 | 0.681 | 0.884 | 1 | 1 | 102 | anatomical structure homeostasis |
| GO:0006954 | 0.681 | 0.884 | 1 | 1 | 102 | inflammatory response |
| GO:0051656 | 0.681 | 0.884 | 1 | 1 | 102 | establishment of organelle localization |
| GO:0006260 | 0.681 | 0.858 | 3 | 3 | 314 | DNA replication |
| GO:0006811 | 0.681 | 0.858 | 3 | 3 | 314 | ion transport |
| GO:0055123 | 0.684 | 0.875 | 1 | 1 | 103 | digestive system development |
| GO:0030111 | 0.684 | 0.875 | 1 | 1 | 103 | regulation of Wnt receptor signaling pathway |
| GO:0009057 | 0.686 | 0.864 | 6 | 5 | 518 | macromolecule catabolic process |
| GO:0043254 | 0.688 | 0.867 | 1 | 1 | 104 | regulation of protein complex assembly |
| GO:0012502 | 0.688 | 0.867 | 1 | 1 | 104 | induction of programmed cell death |
| GO:0006869 | 0.691 | 0.858 | 1 | 1 | 105 | lipid transport |
| GO:0061061 | 0.691 | 0.844 | 4 | 3 | 319 | muscle structure development |
| GO:0006959 | 0.695 | 0.850 | 1 | 1 | 106 | humoral immune response |
| GO:0009266 | 0.695 | 0.850 | 1 | 1 | 106 | response to temperature stimulus |
| GO:0007444 | 0.695 | 0.838 | 4 | 3 | 321 | imaginal disc development |
| GO:0031327 | 0.699 | 0.843 | 5 | 4 | 425 | negative regulation of cellular biosynthetic process |
| GO:0031400 | 0.701 | 0.834 | 1 | 1 | 108 | negative regulation of protein modification process |
| GO:0044087 | 0.702 | 0.820 | 2 | 2 | 219 | regulation of cellular component biogenesis |
| GO:0009890 | 0.704 | 0.837 | 5 | 4 | 428 | negative regulation of biosynthetic process |
| GO:0048193 | 0.705 | 0.826 | 1 | 1 | 109 | Golgi vesicle transport |
| GO:0034762 | 0.705 | 0.826 | 1 | 1 | 109 | regulation of transmembrane transport |
| GO:0051649 | 0.708 | 0.865 | 10 | 9 | 925 | establishment of localization in cell |
| GO:0006412 | 0.714 | 0.824 | 5 | 4 | 434 | translation |
| GO:0010629 | 0.714 | 0.824 | 5 | 4 | 434 | negative regulation of gene expression |
| GO:0006836 | 0.715 | 0.804 | 1 | 1 | 112 | neurotransmitter transport |
| GO:0006364 | 0.718 | 0.796 | 1 | 1 | 113 | rRNA processing |
| GO:0034329 | 0.718 | 0.796 | 1 | 1 | 113 | cell junction assembly |
| GO:0045597 | 0.724 | 0.782 | 1 | 1 | 115 | positive regulation of cell differentiation |
| GO:0071844 | 0.725 | 0.845 | 9 | 8 | 841 | cellular component assembly at cellular level |
| GO:0042063 | 0.727 | 0.775 | 1 | 1 | 116 | gliogenesis |
| GO:0007163 | 0.730 | 0.776 | 3 | 2 | 231 | establishment or maintenance of cell polarity |
| GO:0032970 | 0.730 | 0.768 | 1 | 1 | 117 | regulation of actin filament-based process |
| GO:0016072 | 0.730 | 0.768 | 1 | 1 | 117 | rRNA metabolic process |
| GO:0030029 | 0.732 | 0.789 | 4 | 3 | 340 | actin filament-based process |
| GO:0030155 | 0.733 | 0.762 | 1 | 1 | 118 | regulation of cell adhesion |
| GO:0030534 | 0.733 | 0.762 | 1 | 1 | 118 | adult behavior |
| GO:0006006 | 0.733 | 0.762 | 1 | 1 | 118 | glucose metabolic process |
| GO:0048563 | 0.734 | 0.769 | 3 | 2 | 233 | post-embryonic organ morphogenesis |
| GO:0007560 | 0.734 | 0.769 | 3 | 2 | 233 | imaginal disc morphogenesis |
| GO:0071841 | 0.736 | 0.882 | 26 | 24 | 2374 | cellular component organization or biogenesis at cellular level |
| GO:0001558 | 0.736 | 0.755 | 1 | 1 | 119 | regulation of cell growth |
| GO:0007178 | 0.736 | 0.755 | 1 | 1 | 119 | transmembrane receptor protein serine/threonine kinase signaling pathway |
| GO:0007051 | 0.739 | 0.749 | 1 | 1 | 120 | spindle organization |
| GO:0005975 | 0.739 | 0.807 | 6 | 5 | 552 | carbohydrate metabolic process |
| GO:0007517 | 0.741 | 0.759 | 3 | 2 | 236 | muscle organ development |
| GO:0032386 | 0.742 | 0.743 | 1 | 1 | 121 | regulation of intracellular transport |
| GO:0006606 | 0.742 | 0.743 | 1 | 1 | 121 | protein import into nucleus |
| GO:0001505 | 0.742 | 0.743 | 1 | 1 | 121 | regulation of neurotransmitter levels |
| GO:0051170 | 0.742 | 0.743 | 1 | 1 | 121 | nuclear import |
| GO:0042692 | 0.743 | 0.756 | 3 | 2 | 237 | muscle cell differentiation |
| GO:0006259 | 0.744 | 0.821 | 8 | 7 | 757 | DNA metabolic process |
| GO:0035220 | 0.745 | 0.753 | 3 | 2 | 238 | wing disc development |
| GO:0007049 | 0.747 | 0.832 | 11 | 9 | 958 | cell cycle |
| GO:0006417 | 0.748 | 0.730 | 1 | 1 | 123 | regulation of translation |
| GO:0045892 | 0.748 | 0.768 | 4 | 3 | 349 | negative regulation of transcription, DNA-dependent |
| GO:0010564 | 0.753 | 0.740 | 3 | 2 | 242 | regulation of cell cycle process |
| GO:0050657 | 0.754 | 0.718 | 1 | 1 | 125 | nucleic acid transport |
| GO:0050658 | 0.754 | 0.718 | 1 | 1 | 125 | RNA transport |
| GO:0051236 | 0.754 | 0.718 | 1 | 1 | 125 | establishment of RNA localization |
| GO:0007601 | 0.756 | 0.712 | 1 | 1 | 126 | visual perception |
| GO:0006278 | 0.759 | 0.707 | 1 | 1 | 127 | RNA-dependent DNA replication |
| GO:0050953 | 0.759 | 0.707 | 1 | 1 | 127 | sensory perception of light stimulus |
| GO:0048545 | 0.759 | 0.707 | 1 | 1 | 127 | response to steroid hormone stimulus |
| GO:0034330 | 0.759 | 0.707 | 1 | 1 | 127 | cell junction organization |
| GO:0080135 | 0.764 | 0.695 | 1 | 1 | 129 | regulation of cellular response to stress |
| GO:0051276 | 0.766 | 0.764 | 5 | 4 | 466 | chromosome organization |
| GO:0048514 | 0.767 | 0.690 | 1 | 1 | 130 | blood vessel morphogenesis |
| GO:0048511 | 0.770 | 0.685 | 1 | 1 | 131 | rhythmic process |
| GO:0000910 | 0.772 | 0.679 | 1 | 1 | 132 | cytokinesis |
| GO:0035556 | 0.773 | 0.793 | 9 | 7 | 781 | intracellular signal transduction |
| GO:0000165 | 0.775 | 0.674 | 1 | 1 | 133 | MAPK cascade |
| GO:0031399 | 0.775 | 0.732 | 4 | 3 | 365 | regulation of protein modification process |
| GO:0006200 | 0.777 | 0.669 | 1 | 1 | 134 | ATP catabolic process |
| GO:0016331 | 0.777 | 0.669 | 1 | 1 | 134 | morphogenesis of embryonic epithelium |
| GO:0048569 | 0.779 | 0.700 | 3 | 2 | 255 | post-embryonic organ development |
| GO:0009165 | 0.780 | 0.664 | 1 | 1 | 135 | nucleotide biosynthetic process |
| GO:0015931 | 0.782 | 0.659 | 2 | 1 | 136 | nucleobase-containing compound transport |
| GO:0034504 | 0.785 | 0.654 | 2 | 1 | 137 | protein localization to nucleus |
| GO:0010876 | 0.790 | 0.644 | 2 | 1 | 139 | lipid localization |
| GO:0051253 | 0.792 | 0.710 | 4 | 3 | 376 | negative regulation of RNA metabolic process |
| GO:0044419 | 0.794 | 0.635 | 2 | 1 | 141 | interspecies interaction between organisms |
| GO:0044403 | 0.794 | 0.635 | 2 | 1 | 141 | symbiosis, encompassing mutualism through parasitism |
| GO:0030334 | 0.797 | 0.630 | 2 | 1 | 142 | regulation of cell migration |
| GO:0051640 | 0.801 | 0.621 | 2 | 1 | 144 | organelle localization |
| GO:2000145 | 0.808 | 0.608 | 2 | 1 | 147 | regulation of cell motility |
| GO:0007519 | 0.810 | 0.604 | 2 | 1 | 148 | skeletal muscle tissue development |
| GO:0044265 | 0.811 | 0.685 | 4 | 3 | 389 | cellular macromolecule catabolic process |
| GO:0015672 | 0.812 | 0.600 | 2 | 1 | 149 | monovalent inorganic cation transport |
| GO:0033043 | 0.817 | 0.643 | 3 | 2 | 277 | regulation of organelle organization |
| GO:0019318 | 0.818 | 0.588 | 2 | 1 | 152 | hexose metabolic process |
| GO:0055002 | 0.818 | 0.588 | 2 | 1 | 152 | striated muscle cell development |
| GO:0010605 | 0.820 | 0.718 | 7 | 5 | 615 | negative regulation of macromolecule metabolic process |
| GO:0016052 | 0.820 | 0.584 | 2 | 1 | 153 | carbohydrate catabolic process |
| GO:0042391 | 0.822 | 0.580 | 2 | 1 | 154 | regulation of membrane potential |
| GO:0033554 | 0.824 | 0.728 | 8 | 6 | 725 | cellular response to stress |
| GO:0032446 | 0.826 | 0.628 | 3 | 2 | 283 | protein modification by small protein conjugation |
| GO:2000113 | 0.826 | 0.665 | 4 | 3 | 400 | negative regulation of cellular macromolecule biosynthetic process |
| GO:0010558 | 0.830 | 0.660 | 4 | 3 | 403 | negative regulation of macromolecule biosynthetic process |
| GO:0006403 | 0.830 | 0.565 | 2 | 1 | 158 | RNA localization |
| GO:0017038 | 0.830 | 0.565 | 2 | 1 | 158 | protein import |
| GO:0045934 | 0.834 | 0.654 | 4 | 3 | 406 | negative regulation of nucleobase-containing compound metabolic process |
| GO:0051172 | 0.834 | 0.654 | 4 | 3 | 406 | negative regulation of nitrogen compound metabolic process |
| GO:0032268 | 0.834 | 0.681 | 6 | 4 | 519 | regulation of cellular protein metabolic process |
| GO:0008380 | 0.835 | 0.615 | 3 | 2 | 289 | RNA splicing |
| GO:0007017 | 0.835 | 0.653 | 5 | 3 | 407 | microtubule-based process |
| GO:0032269 | 0.836 | 0.554 | 2 | 1 | 161 | negative regulation of cellular protein metabolic process |
| GO:0046907 | 0.839 | 0.695 | 7 | 5 | 633 | intracellular transport |
| GO:0000398 | 0.845 | 0.537 | 2 | 1 | 166 | nuclear mRNA splicing, via spliceosome |
| GO:0000377 | 0.845 | 0.537 | 2 | 1 | 166 | RNA splicing, via transesterification reactions with bulged adenosine as nucleophile |
| GO:0042254 | 0.847 | 0.534 | 2 | 1 | 167 | ribosome biogenesis |
| GO:0007015 | 0.850 | 0.527 | 2 | 1 | 169 | actin filament organization |
| GO:0000375 | 0.850 | 0.527 | 2 | 1 | 169 | RNA splicing, via transesterification reactions |
| GO:0005996 | 0.850 | 0.527 | 2 | 1 | 169 | monosaccharide metabolic process |
| GO:0060538 | 0.854 | 0.521 | 2 | 1 | 171 | skeletal muscle organ development |
| GO:0051641 | 0.855 | 0.730 | 12 | 9 | 1073 | cellular localization |
| GO:0051248 | 0.857 | 0.515 | 2 | 1 | 173 | negative regulation of protein metabolic process |
| GO:0051329 | 0.860 | 0.509 | 2 | 1 | 175 | interphase of mitotic cell cycle |
| GO:0006996 | 0.860 | 0.755 | 16 | 13 | 1485 | organelle organization |
| GO:0051179 | 0.865 | 0.783 | 25 | 21 | 2275 | localization |
| GO:0051325 | 0.865 | 0.500 | 2 | 1 | 178 | interphase |
| GO:0016192 | 0.870 | 0.658 | 7 | 5 | 666 | vesicle-mediated transport |
| GO:0000278 | 0.871 | 0.633 | 6 | 4 | 555 | mitotic cell cycle |
| GO:0008285 | 0.872 | 0.486 | 2 | 1 | 183 | negative regulation of cell proliferation |
| GO:0007476 | 0.874 | 0.483 | 2 | 1 | 184 | imaginal disc-derived wing morphogenesis |
| GO:0007346 | 0.877 | 0.478 | 2 | 1 | 186 | regulation of mitotic cell cycle |
| GO:0007472 | 0.877 | 0.478 | 2 | 1 | 186 | wing disc morphogenesis |
| GO:0010627 | 0.881 | 0.470 | 2 | 1 | 189 | regulation of intracellular protein kinase cascade |
| GO:0030036 | 0.881 | 0.542 | 4 | 2 | 326 | actin cytoskeleton organization |
| GO:0048232 | 0.882 | 0.467 | 2 | 1 | 190 | male gamete generation |
| GO:0007283 | 0.882 | 0.467 | 2 | 1 | 190 | spermatogenesis |
| GO:0002009 | 0.882 | 0.540 | 4 | 2 | 327 | morphogenesis of an epithelium |
| GO:0015031 | 0.890 | 0.632 | 8 | 5 | 691 | protein transport |
| GO:0035120 | 0.891 | 0.450 | 2 | 1 | 197 | post-embryonic appendage morphogenesis |
| GO:0035114 | 0.895 | 0.443 | 2 | 1 | 200 | imaginal disc-derived appendage morphogenesis |
| GO:0000122 | 0.895 | 0.443 | 2 | 1 | 200 | negative regulation of transcription from RNA polymerase II promoter |
| GO:0048737 | 0.898 | 0.437 | 2 | 1 | 203 | imaginal disc-derived appendage development |
| GO:0045184 | 0.903 | 0.613 | 8 | 5 | 710 | establishment of protein localization |
| GO:0051246 | 0.904 | 0.586 | 7 | 4 | 596 | regulation of protein metabolic process |
| GO:0050877 | 0.906 | 0.609 | 8 | 5 | 714 | neurological system process |
| GO:0051130 | 0.906 | 0.422 | 2 | 1 | 210 | positive regulation of cellular component organization |
| GO:0006810 | 0.910 | 0.714 | 20 | 15 | 1778 | transport |
| GO:0006397 | 0.917 | 0.481 | 4 | 2 | 365 | mRNA processing |
| GO:0043623 | 0.921 | 0.393 | 2 | 1 | 225 | cellular protein complex assembly |
| GO:0022402 | 0.923 | 0.584 | 8 | 5 | 742 | cell cycle process |
| GO:0003008 | 0.925 | 0.602 | 10 | 6 | 860 | system process |
| GO:0071702 | 0.926 | 0.382 | 3 | 1 | 231 | organic substance transport |
| GO:0051234 | 0.928 | 0.693 | 20 | 15 | 1820 | establishment of localization |
| GO:0006886 | 0.932 | 0.453 | 4 | 2 | 387 | intracellular protein transport |
| GO:0006605 | 0.938 | 0.358 | 3 | 1 | 246 | protein targeting |
| GO:0035107 | 0.938 | 0.358 | 3 | 1 | 246 | appendage morphogenesis |
| GO:0034613 | 0.940 | 0.489 | 6 | 3 | 533 | cellular protein localization |
| GO:0048736 | 0.943 | 0.348 | 3 | 1 | 253 | appendage development |
| GO:0033365 | 0.944 | 0.345 | 3 | 1 | 255 | protein localization to organelle |
| GO:0007243 | 0.949 | 0.334 | 3 | 1 | 263 | intracellular protein kinase cascade |
| GO:0016567 | 0.952 | 0.328 | 3 | 1 | 268 | protein ubiquitination |
| GO:0070727 | 0.954 | 0.461 | 6 | 3 | 564 | cellular macromolecule localization |
| GO:0016071 | 0.957 | 0.400 | 5 | 2 | 435 | mRNA metabolic process |
| GO:0022403 | 0.992 | 0.285 | 7 | 2 | 596 | cell cycle phase |
| GO:0000279 | 0.995 | 0.185 | 5 | 1 | 462 | M phase |
