# Supplementary material for: Inducible Defenses Stay Up Late: Temporal Patterns of Immune Gene Expression in Tenebrio molitor
Source: G3 (Bethesda). 2014 Jun 1;4(6):947–55. doi: 10.1534/g3.113.008516 (PMC4065263; doi:10.1534/g3.113.008516)
Supplement: Supporting Information [file supp_g3.113.008516_TableS16.html]

TableS16 

# Inducible Defenses Stay Up Late: Temporal Patterns of Immune Gene Expression in *Tenebrio molitor*

Gene to GO MF test for over-representation

| GOMFID | Pvalue | OddsRatio | ExpCount | Count | Size | Term |
| GO:0045735 | 0.000 | 30.004 | 0 | 4 | 16 | nutrient reservoir activity |
| GO:0070402 | 0.001 | 58.768 | 0 | 2 | 5 | NADPH binding |
| GO:0016628 | 0.002 | 14.826 | 0 | 3 | 21 | oxidoreductase activity, acting on the CH-CH group of donors, NAD or NADP as acceptor |
| GO:0050662 | 0.002 | 3.797 | 2 | 8 | 202 | coenzyme binding |
| GO:0001540 | 0.003 | 35.252 | 0 | 2 | 7 | beta-amyloid binding |
| GO:0004320 | 0.003 | 35.252 | 0 | 2 | 7 | oleoyl-[acyl-carrier-protein] hydrolase activity |
| GO:0004313 | 0.003 | 35.252 | 0 | 2 | 7 | [acyl-carrier-protein] S-acetyltransferase activity |
| GO:0004317 | 0.003 | 35.252 | 0 | 2 | 7 | 3-hydroxypalmitoyl-[acyl-carrier-protein] dehydratase activity |
| GO:0004319 | 0.003 | 35.252 | 0 | 2 | 7 | enoyl-[acyl-carrier-protein] reductase (NADPH, B-specific) activity |
| GO:0047451 | 0.003 | 35.252 | 0 | 2 | 7 | 3-hydroxyoctanoyl-[acyl-carrier-protein] dehydratase activity |
| GO:0016295 | 0.003 | 35.252 | 0 | 2 | 7 | myristoyl-[acyl-carrier-protein] hydrolase activity |
| GO:0016296 | 0.003 | 35.252 | 0 | 2 | 7 | palmitoyl-[acyl-carrier-protein] hydrolase activity |
| GO:0016297 | 0.003 | 35.252 | 0 | 2 | 7 | acyl-[acyl-carrier-protein] hydrolase activity |
| GO:0019171 | 0.003 | 35.252 | 0 | 2 | 7 | 3-hydroxyacyl-[acyl-carrier-protein] dehydratase activity |
| GO:0005515 | 0.003 | 2.112 | 13 | 23 | 1102 | protein binding |
| GO:0016627 | 0.003 | 7.627 | 1 | 4 | 51 | oxidoreductase activity, acting on the CH-CH group of donors |
| GO:0042803 | 0.003 | 3.999 | 2 | 7 | 167 | protein homodimerization activity |
| GO:0004314 | 0.003 | 29.373 | 0 | 2 | 8 | [acyl-carrier-protein] S-malonyltransferase activity |
| GO:0016420 | 0.003 | 29.373 | 0 | 2 | 8 | malonyltransferase activity |
| GO:0016418 | 0.003 | 29.373 | 0 | 2 | 8 | S-acetyltransferase activity |
| GO:0016419 | 0.003 | 29.373 | 0 | 2 | 8 | S-malonyltransferase activity |
| GO:0048037 | 0.004 | 3.124 | 3 | 9 | 275 | cofactor binding |
| GO:0042802 | 0.004 | 3.737 | 2 | 7 | 178 | identical protein binding |
| GO:0004315 | 0.006 | 22.024 | 0 | 2 | 10 | 3-oxoacyl-[acyl-carrier-protein] synthase activity |
| GO:0004316 | 0.006 | 22.024 | 0 | 2 | 10 | 3-oxoacyl-[acyl-carrier-protein] reductase (NADPH) activity |
| GO:0004767 | 0.007 | 19.575 | 0 | 2 | 11 | sphingomyelin phosphodiesterase activity |
| GO:0008641 | 0.008 | 17.615 | 0 | 2 | 12 | small protein activating enzyme activity |
| GO:0019899 | 0.009 | 3.644 | 2 | 6 | 155 | enzyme binding |
| GO:0008762 | 0.009 | 16.012 | 0 | 2 | 13 | UDP-N-acetylmuramate dehydrogenase activity |
| GO:0046983 | 0.009 | 3.183 | 2 | 7 | 207 | protein dimerization activity |
| GO:0004312 | 0.011 | 14.676 | 0 | 2 | 14 | fatty acid synthase activity |
| GO:0008022 | 0.011 | 14.676 | 0 | 2 | 14 | protein C-terminus binding |
| GO:0016417 | 0.011 | 14.676 | 0 | 2 | 14 | S-acyltransferase activity |
| GO:0035939 | 0.011 | Inf | 0 | 1 | 1 | microsatellite binding |
| GO:0008709 | 0.011 | Inf | 0 | 1 | 1 | cholate 7-alpha-dehydrogenase activity |
| GO:0015930 | 0.011 | Inf | 0 | 1 | 1 | glutamate synthase activity |
| GO:0070300 | 0.011 | Inf | 0 | 1 | 1 | phosphatidic acid binding |
| GO:0046965 | 0.011 | Inf | 0 | 1 | 1 | retinoid X receptor binding |
| GO:0046921 | 0.011 | Inf | 0 | 1 | 1 | alpha-(1->6)-fucosyltransferase activity |
| GO:0016040 | 0.011 | Inf | 0 | 1 | 1 | glutamate synthase (NADH) activity |
| GO:0050290 | 0.011 | Inf | 0 | 1 | 1 | sphingomyelin phosphodiesterase D activity |
| GO:0030337 | 0.011 | Inf | 0 | 1 | 1 | DNA polymerase processivity factor activity |
| GO:0047969 | 0.011 | Inf | 0 | 1 | 1 | glyoxylate oxidase activity |
| GO:0030306 | 0.011 | Inf | 0 | 1 | 1 | ADP-ribosylation factor binding |
| GO:0052852 | 0.011 | Inf | 0 | 1 | 1 | very-long-chain-(S)-2-hydroxy-acid oxidase activity |
| GO:0052853 | 0.011 | Inf | 0 | 1 | 1 | long-chain-(S)-2-hydroxy-long-chain-acid oxidase activity |
| GO:0052854 | 0.011 | Inf | 0 | 1 | 1 | medium-chain-(S)-2-hydroxy-acid oxidase activity |
| GO:0042292 | 0.011 | Inf | 0 | 1 | 1 | URM1 activating enzyme activity |
| GO:0045181 | 0.011 | Inf | 0 | 1 | 1 | glutamate synthase activity, NADH or NADPH as acceptor |
| GO:0047022 | 0.011 | Inf | 0 | 1 | 1 | 7-beta-hydroxysteroid dehydrogenase (NADP+) activity |
| GO:0047015 | 0.011 | Inf | 0 | 1 | 1 | 3-hydroxy-2-methylbutyryl-CoA dehydrogenase activity |
| GO:0017099 | 0.011 | Inf | 0 | 1 | 1 | very-long-chain-acyl-CoA dehydrogenase activity |
| GO:0004355 | 0.011 | Inf | 0 | 1 | 1 | glutamate synthase (NADPH) activity |
| GO:0004496 | 0.011 | Inf | 0 | 1 | 1 | mevalonate kinase activity |
| GO:0051990 | 0.011 | Inf | 0 | 1 | 1 | (R)-2-hydroxyglutarate dehydrogenase activity |
| GO:0004466 | 0.011 | Inf | 0 | 1 | 1 | long-chain-acyl-CoA dehydrogenase activity |
| GO:0005536 | 0.011 | Inf | 0 | 1 | 1 | glucose binding |
| GO:0030899 | 0.011 | Inf | 0 | 1 | 1 | calcium-dependent ATPase activity |
| GO:0008424 | 0.011 | Inf | 0 | 1 | 1 | glycoprotein 6-alpha-L-fucosyltransferase activity |
| GO:0046875 | 0.011 | Inf | 0 | 1 | 1 | ephrin receptor binding |
| GO:0008418 | 0.011 | Inf | 0 | 1 | 1 | protein-N-terminal asparagine amidohydrolase activity |
| GO:0034722 | 0.011 | Inf | 0 | 1 | 1 | gamma-glutamyl-peptidase activity |
| GO:0060001 | 0.011 | Inf | 0 | 1 | 1 | minus-end directed microfilament motor activity |
| GO:0018454 | 0.011 | Inf | 0 | 1 | 1 | acetoacetyl-CoA reductase activity |
| GO:0010181 | 0.016 | 11.736 | 0 | 2 | 17 | FMN binding |
| GO:0019209 | 0.018 | 11.001 | 0 | 2 | 18 | kinase activator activity |
| GO:0008013 | 0.020 | 10.353 | 0 | 2 | 19 | beta-catenin binding |
| GO:0016614 | 0.022 | 2.922 | 2 | 6 | 191 | oxidoreductase activity, acting on CH-OH group of donors |
| GO:0016623 | 0.023 | 87.226 | 0 | 1 | 2 | oxidoreductase activity, acting on the aldehyde or oxo group of donors, oxygen as acceptor |
| GO:0003973 | 0.023 | 87.226 | 0 | 1 | 2 | (S)-2-hydroxy-acid oxidase activity |
| GO:0008891 | 0.023 | 87.226 | 0 | 1 | 2 | glycolate oxidase activity |
| GO:0015019 | 0.023 | 87.226 | 0 | 1 | 2 | heparan-alpha-glucosaminide N-acetyltransferase activity |
| GO:0008531 | 0.023 | 87.226 | 0 | 1 | 2 | riboflavin kinase activity |
| GO:0017050 | 0.023 | 87.226 | 0 | 1 | 2 | D-erythro-sphingosine kinase activity |
| GO:0050510 | 0.023 | 87.226 | 0 | 1 | 2 | N-acetylgalactosaminyl-proteoglycan 3-beta-glucuronosyltransferase activity |
| GO:0050614 | 0.023 | 87.226 | 0 | 1 | 2 | delta24-sterol reductase activity |
| GO:0045545 | 0.023 | 87.226 | 0 | 1 | 2 | syndecan binding |
| GO:0004792 | 0.023 | 87.226 | 0 | 1 | 2 | thiosulfate sulfurtransferase activity |
| GO:0003696 | 0.023 | 87.226 | 0 | 1 | 2 | satellite DNA binding |
| GO:0008481 | 0.023 | 87.226 | 0 | 1 | 2 | sphinganine kinase activity |
| GO:0017174 | 0.023 | 87.226 | 0 | 1 | 2 | glycine N-methyltransferase activity |
| GO:0016899 | 0.023 | 87.226 | 0 | 1 | 2 | oxidoreductase activity, acting on the CH-OH group of donors, oxygen as acceptor |
| GO:0047238 | 0.023 | 87.226 | 0 | 1 | 2 | glucuronosyl-N-acetylgalactosaminyl-proteoglycan 4-beta-N-acetylgalactosaminyltransferase activity |
| GO:0000979 | 0.023 | 87.226 | 0 | 1 | 2 | RNA polymerase II core promoter sequence-specific DNA binding |
| GO:0005543 | 0.025 | 2.595 | 3 | 7 | 251 | phospholipid binding |
| GO:0050660 | 0.027 | 3.751 | 1 | 4 | 99 | flavin adenine dinucleotide binding |
| GO:0016407 | 0.032 | 4.578 | 1 | 3 | 61 | acetyltransferase activity |
| GO:0003980 | 0.034 | 43.608 | 0 | 1 | 3 | UDP-glucose:glycoprotein glucosyltransferase activity |
| GO:0003983 | 0.034 | 43.608 | 0 | 1 | 3 | UTP:glucose-1-phosphate uridylyltransferase activity |
| GO:0051748 | 0.034 | 43.608 | 0 | 1 | 3 | UTP-monosaccharide-1-phosphate uridylyltransferase activity |
| GO:0001727 | 0.034 | 43.608 | 0 | 1 | 3 | lipid kinase activity |
| GO:0050294 | 0.034 | 43.608 | 0 | 1 | 3 | steroid sulfotransferase activity |
| GO:0070883 | 0.034 | 43.608 | 0 | 1 | 3 | pre-miRNA binding |
| GO:0004366 | 0.034 | 43.608 | 0 | 1 | 3 | glycerol-3-phosphate O-acyltransferase activity |
| GO:0004304 | 0.034 | 43.608 | 0 | 1 | 3 | estrone sulfotransferase activity |
| GO:0016443 | 0.034 | 43.608 | 0 | 1 | 3 | bidentate ribonuclease III activity |
| GO:0032557 | 0.034 | 43.608 | 0 | 1 | 3 | pyrimidine ribonucleotide binding |
| GO:0019103 | 0.034 | 43.608 | 0 | 1 | 3 | pyrimidine nucleotide binding |
| GO:0047894 | 0.034 | 43.608 | 0 | 1 | 3 | flavonol 3-sulfotransferase activity |
| GO:0016616 | 0.037 | 2.866 | 2 | 5 | 161 | oxidoreductase activity, acting on the CH-OH group of donors, NAD or NADP as acceptor |
| GO:0005549 | 0.038 | 7.033 | 0 | 2 | 27 | odorant binding |
| GO:0043565 | 0.039 | 2.346 | 3 | 7 | 276 | sequence-specific DNA binding |
| GO:0016788 | 0.041 | 1.783 | 8 | 14 | 739 | hydrolase activity, acting on ester bonds |
| GO:0016790 | 0.041 | 4.146 | 1 | 3 | 67 | thiolester hydrolase activity |
| GO:0016746 | 0.041 | 2.775 | 2 | 5 | 166 | transferase activity, transferring acyl groups |
| GO:0008289 | 0.043 | 2.153 | 4 | 8 | 344 | lipid binding |
| GO:0003700 | 0.044 | 2.042 | 5 | 9 | 409 | sequence-specific DNA binding transcription factor activity |
| GO:0001071 | 0.044 | 2.042 | 5 | 9 | 409 | nucleic acid binding transcription factor activity |
| GO:0050327 | 0.045 | 29.068 | 0 | 1 | 4 | testosterone 17-beta-dehydrogenase (NAD+) activity |
| GO:0051538 | 0.045 | 29.068 | 0 | 1 | 4 | 3 iron, 4 sulfur cluster binding |
| GO:0015450 | 0.045 | 29.068 | 0 | 1 | 4 | P-P-bond-hydrolysis-driven protein transmembrane transporter activity |
| GO:0047035 | 0.045 | 29.068 | 0 | 1 | 4 | testosterone dehydrogenase (NAD+) activity |
| GO:0004303 | 0.045 | 29.068 | 0 | 1 | 4 | estradiol 17-beta-dehydrogenase activity |
| GO:0008121 | 0.045 | 29.068 | 0 | 1 | 4 | ubiquinol-cytochrome-c reductase activity |
| GO:0042974 | 0.045 | 29.068 | 0 | 1 | 4 | retinoic acid receptor binding |
| GO:0000983 | 0.045 | 29.068 | 0 | 1 | 4 | RNA polymerase II core promoter sequence-specific DNA binding transcription factor activity |
| GO:0016681 | 0.045 | 29.068 | 0 | 1 | 4 | oxidoreductase activity, acting on diphenols and related substances as donors, cytochrome as acceptor |
| GO:0030234 | 0.047 | 1.940 | 5 | 10 | 479 | enzyme regulator activity |
| GO:0016740 | 0.049 | 1.553 | 16 | 23 | 1423 | transferase activity |
| GO:0031267 | 0.052 | 5.857 | 0 | 2 | 32 | small GTPase binding |
| GO:0017016 | 0.052 | 5.857 | 0 | 2 | 32 | Ras GTPase binding |
| GO:0016782 | 0.052 | 5.857 | 0 | 2 | 32 | transferase activity, transferring sulfur-containing groups |
| GO:0008270 | 0.054 | 1.588 | 13 | 19 | 1135 | zinc ion binding |
| GO:0016639 | 0.056 | 21.798 | 0 | 1 | 5 | oxidoreductase activity, acting on the CH-NH2 group of donors, NAD or NADP as acceptor |
| GO:0046966 | 0.056 | 21.798 | 0 | 1 | 5 | thyroid hormone receptor binding |
| GO:0004045 | 0.056 | 21.798 | 0 | 1 | 5 | aminoacyl-tRNA hydrolase activity |
| GO:0001875 | 0.056 | 21.798 | 0 | 1 | 5 | lipopolysaccharide receptor activity |
| GO:0008035 | 0.056 | 21.798 | 0 | 1 | 5 | high-density lipoprotein particle binding |
| GO:0030276 | 0.056 | 21.798 | 0 | 1 | 5 | clathrin binding |
| GO:0070506 | 0.056 | 21.798 | 0 | 1 | 5 | high-density lipoprotein particle receptor activity |
| GO:0033265 | 0.056 | 21.798 | 0 | 1 | 5 | choline binding |
| GO:0003878 | 0.056 | 21.798 | 0 | 1 | 5 | ATP citrate synthase activity |
| GO:0042605 | 0.056 | 21.798 | 0 | 1 | 5 | peptide antigen binding |
| GO:0016594 | 0.056 | 21.798 | 0 | 1 | 5 | glycine binding |
| GO:0001077 | 0.057 | 5.490 | 0 | 2 | 34 | RNA polymerase II core promoter proximal region sequence-specific DNA binding transcription factor activity involved in positive regulation of transcription |
| GO:0001228 | 0.061 | 5.323 | 0 | 2 | 35 | RNA polymerase II transcription regulatory region sequence-specific DNA binding transcription factor activity involved in positive regulation of transcription |
| GO:0051020 | 0.061 | 5.323 | 0 | 2 | 35 | GTPase binding |
| GO:0008144 | 0.061 | 5.323 | 0 | 2 | 35 | drug binding |
| GO:0016747 | 0.063 | 2.795 | 2 | 4 | 131 | transferase activity, transferring acyl groups other than amino-acyl groups |
| GO:0005496 | 0.064 | 5.166 | 0 | 2 | 36 | steroid binding |
| GO:0050661 | 0.067 | 5.017 | 0 | 2 | 37 | NADP binding |
| GO:0016836 | 0.067 | 5.017 | 0 | 2 | 37 | hydro-lyase activity |
| GO:0031418 | 0.067 | 17.437 | 0 | 1 | 6 | L-ascorbic acid binding |
| GO:0005388 | 0.067 | 17.437 | 0 | 1 | 6 | calcium-transporting ATPase activity |
| GO:0019905 | 0.067 | 17.437 | 0 | 1 | 6 | syntaxin binding |
| GO:0022884 | 0.067 | 17.437 | 0 | 1 | 6 | macromolecule transmembrane transporter activity |
| GO:0008320 | 0.067 | 17.437 | 0 | 1 | 6 | protein transmembrane transporter activity |
| GO:0016783 | 0.067 | 17.437 | 0 | 1 | 6 | sulfurtransferase activity |
| GO:0050997 | 0.067 | 17.437 | 0 | 1 | 6 | quaternary ammonium group binding |
| GO:0017137 | 0.067 | 17.437 | 0 | 1 | 6 | Rab GTPase binding |
| GO:0019900 | 0.070 | 4.877 | 0 | 2 | 38 | kinase binding |
| GO:0001786 | 0.077 | 14.529 | 0 | 1 | 7 | phosphatidylserine binding |
| GO:0030331 | 0.077 | 14.529 | 0 | 1 | 7 | estrogen receptor binding |
| GO:0000146 | 0.077 | 14.529 | 0 | 1 | 7 | microfilament motor activity |
| GO:0000149 | 0.077 | 14.529 | 0 | 1 | 7 | SNARE binding |
| GO:0035251 | 0.077 | 14.529 | 0 | 1 | 7 | UDP-glucosyltransferase activity |
| GO:0070569 | 0.077 | 14.529 | 0 | 1 | 7 | uridylyltransferase activity |
| GO:0005542 | 0.077 | 14.529 | 0 | 1 | 7 | folic acid binding |
| GO:0003857 | 0.077 | 14.529 | 0 | 1 | 7 | 3-hydroxyacyl-CoA dehydrogenase activity |
| GO:0008417 | 0.077 | 14.529 | 0 | 1 | 7 | fucosyltransferase activity |
| GO:0004828 | 0.077 | 14.529 | 0 | 1 | 7 | serine-tRNA ligase activity |
| GO:0046527 | 0.077 | 14.529 | 0 | 1 | 7 | glucosyltransferase activity |
| GO:0004525 | 0.077 | 14.529 | 0 | 1 | 7 | ribonuclease III activity |
| GO:0001012 | 0.080 | 4.501 | 0 | 2 | 41 | RNA polymerase II regulatory region DNA binding |
| GO:0000977 | 0.080 | 4.501 | 0 | 2 | 41 | RNA polymerase II regulatory region sequence-specific DNA binding |
| GO:0001530 | 0.088 | 12.452 | 0 | 1 | 8 | lipopolysaccharide binding |
| GO:0033764 | 0.088 | 12.452 | 0 | 1 | 8 | steroid dehydrogenase activity, acting on the CH-OH group of donors, NAD or NADP as acceptor |
| GO:0008329 | 0.088 | 12.452 | 0 | 1 | 8 | pattern recognition receptor activity |
| GO:0004775 | 0.088 | 12.452 | 0 | 1 | 8 | succinate-CoA ligase (ADP-forming) activity |
| GO:0030515 | 0.088 | 12.452 | 0 | 1 | 8 | snoRNA binding |
| GO:0005319 | 0.094 | 4.080 | 1 | 2 | 45 | lipid transporter activity |
| GO:0016679 | 0.099 | 10.894 | 0 | 1 | 9 | oxidoreductase activity, acting on diphenols and related substances as donors |
| GO:0046912 | 0.099 | 10.894 | 0 | 1 | 9 | transferase activity, transferring acyl groups, acyl groups converted into alkyl on transfer |
| GO:0005112 | 0.099 | 10.894 | 0 | 1 | 9 | Notch binding |
| GO:0003823 | 0.099 | 10.894 | 0 | 1 | 9 | antigen binding |
| GO:0001948 | 0.099 | 10.894 | 0 | 1 | 9 | glycoprotein binding |
| GO:0016229 | 0.099 | 10.894 | 0 | 1 | 9 | steroid dehydrogenase activity |
| GO:0004774 | 0.099 | 10.894 | 0 | 1 | 9 | succinate-CoA ligase activity |
| GO:0016835 | 0.108 | 3.731 | 1 | 2 | 49 | carbon-oxygen lyase activity |
| GO:0046914 | 0.108 | 1.402 | 17 | 22 | 1474 | transition metal ion binding |
| GO:0004341 | 0.109 | 9.682 | 0 | 1 | 10 | gluconolactonase activity |
| GO:0003990 | 0.109 | 9.682 | 0 | 1 | 10 | acetylcholinesterase activity |
| GO:0046872 | 0.114 | 1.320 | 32 | 38 | 2792 | metal ion binding |
| GO:0030169 | 0.119 | 8.713 | 0 | 1 | 11 | low-density lipoprotein particle binding |
| GO:0008242 | 0.119 | 8.713 | 0 | 1 | 11 | omega peptidase activity |
| GO:0043169 | 0.120 | 1.311 | 33 | 39 | 2887 | cation binding |
| GO:0043167 | 0.120 | 1.310 | 33 | 39 | 2888 | ion binding |
| GO:0008081 | 0.123 | 3.436 | 1 | 2 | 53 | phosphoric diester hydrolase activity |
| GO:0005089 | 0.123 | 3.436 | 1 | 2 | 53 | Rho guanyl-nucleotide exchange factor activity |
| GO:0019842 | 0.124 | 2.539 | 1 | 3 | 107 | vitamin binding |
| GO:0016638 | 0.129 | 7.920 | 0 | 1 | 12 | oxidoreductase activity, acting on the CH-NH2 group of donors |
| GO:0005520 | 0.129 | 7.920 | 0 | 1 | 12 | insulin-like growth factor binding |
| GO:0004104 | 0.129 | 7.920 | 0 | 1 | 12 | cholinesterase activity |
| GO:0008376 | 0.129 | 7.920 | 0 | 1 | 12 | acetylgalactosaminyltransferase activity |
| GO:0003680 | 0.129 | 7.920 | 0 | 1 | 12 | AT DNA binding |
| GO:0019207 | 0.131 | 3.306 | 1 | 2 | 55 | kinase regulator activity |
| GO:0008047 | 0.134 | 2.102 | 2 | 4 | 172 | enzyme activator activity |
| GO:0004620 | 0.135 | 3.244 | 1 | 2 | 56 | phospholipase activity |
| GO:0030295 | 0.139 | 7.259 | 0 | 1 | 13 | protein kinase activator activity |
| GO:0043178 | 0.139 | 7.259 | 0 | 1 | 13 | alcohol binding |
| GO:0000978 | 0.139 | 7.259 | 0 | 1 | 13 | RNA polymerase II core promoter proximal region sequence-specific DNA binding |
| GO:0030165 | 0.149 | 6.700 | 0 | 1 | 14 | PDZ domain binding |
| GO:0035258 | 0.149 | 6.700 | 0 | 1 | 14 | steroid hormone receptor binding |
| GO:0004143 | 0.149 | 6.700 | 0 | 1 | 14 | diacylglycerol kinase activity |
| GO:0030552 | 0.149 | 6.700 | 0 | 1 | 14 | cAMP binding |
| GO:0008431 | 0.149 | 6.700 | 0 | 1 | 14 | vitamin E binding |
| GO:0000981 | 0.153 | 2.293 | 1 | 3 | 118 | sequence-specific DNA binding RNA polymerase II transcription factor activity |
| GO:0050839 | 0.154 | 2.968 | 1 | 2 | 61 | cell adhesion molecule binding |
| GO:0000982 | 0.154 | 2.968 | 1 | 2 | 61 | RNA polymerase II core promoter proximal region sequence-specific DNA binding transcription factor activity |
| GO:0004519 | 0.155 | 1.981 | 2 | 4 | 182 | endonuclease activity |
| GO:0071813 | 0.159 | 6.220 | 0 | 1 | 15 | lipoprotein particle binding |
| GO:0071814 | 0.159 | 6.220 | 0 | 1 | 15 | protein-lipid complex binding |
| GO:0004298 | 0.159 | 6.220 | 0 | 1 | 15 | threonine-type endopeptidase activity |
| GO:0070003 | 0.159 | 6.220 | 0 | 1 | 15 | threonine-type peptidase activity |
| GO:0000062 | 0.159 | 6.220 | 0 | 1 | 15 | fatty-acyl-CoA binding |
| GO:0003995 | 0.159 | 6.220 | 0 | 1 | 15 | acyl-CoA dehydrogenase activity |
| GO:0008233 | 0.160 | 1.488 | 7 | 10 | 611 | peptidase activity |
| GO:0005509 | 0.163 | 1.611 | 5 | 7 | 393 | calcium ion binding |
| GO:0045296 | 0.168 | 5.805 | 0 | 1 | 16 | cadherin binding |
| GO:0016874 | 0.171 | 1.589 | 5 | 7 | 398 | ligase activity |
| GO:0001159 | 0.178 | 5.442 | 0 | 1 | 17 | core promoter proximal region DNA binding |
| GO:0030374 | 0.178 | 5.442 | 0 | 1 | 17 | ligand-dependent nuclear receptor transcription coactivator activity |
| GO:0030228 | 0.178 | 5.442 | 0 | 1 | 17 | lipoprotein particle receptor activity |
| GO:0001046 | 0.178 | 5.442 | 0 | 1 | 17 | core promoter sequence-specific DNA binding |
| GO:0043621 | 0.178 | 5.442 | 0 | 1 | 17 | protein self-association |
| GO:0000987 | 0.178 | 5.442 | 0 | 1 | 17 | core promoter proximal region sequence-specific DNA binding |
| GO:0003993 | 0.178 | 5.442 | 0 | 1 | 17 | acid phosphatase activity |
| GO:0016758 | 0.181 | 2.107 | 1 | 3 | 128 | transferase activity, transferring hexosyl groups |
| GO:0031406 | 0.183 | 2.651 | 1 | 2 | 68 | carboxylic acid binding |
| GO:0050136 | 0.187 | 5.121 | 0 | 1 | 18 | NADH dehydrogenase (quinone) activity |
| GO:0008137 | 0.187 | 5.121 | 0 | 1 | 18 | NADH dehydrogenase (ubiquinone) activity |
| GO:0015085 | 0.191 | 2.572 | 1 | 2 | 70 | calcium ion transmembrane transporter activity |
| GO:0008170 | 0.191 | 2.572 | 1 | 2 | 70 | N-methyltransferase activity |
| GO:0044212 | 0.195 | 2.024 | 2 | 3 | 133 | transcription regulatory region DNA binding |
| GO:0003954 | 0.197 | 4.836 | 0 | 1 | 19 | NADH dehydrogenase activity |
| GO:0051082 | 0.197 | 4.836 | 0 | 1 | 19 | unfolded protein binding |
| GO:0042562 | 0.197 | 4.836 | 0 | 1 | 19 | hormone binding |
| GO:0001067 | 0.198 | 2.009 | 2 | 3 | 134 | regulatory region nucleic acid binding |
| GO:0000975 | 0.198 | 2.009 | 2 | 3 | 134 | regulatory region DNA binding |
| GO:0052689 | 0.204 | 1.978 | 2 | 3 | 136 | carboxylic ester hydrolase activity |
| GO:0048029 | 0.206 | 4.581 | 0 | 1 | 20 | monosaccharide binding |
| GO:0019829 | 0.206 | 4.581 | 0 | 1 | 20 | cation-transporting ATPase activity |
| GO:0019838 | 0.206 | 4.581 | 0 | 1 | 20 | growth factor binding |
| GO:0005088 | 0.208 | 2.428 | 1 | 2 | 74 | Ras guanyl-nucleotide exchange factor activity |
| GO:0042800 | 0.215 | 4.351 | 0 | 1 | 21 | histone methyltransferase activity (H3-K4 specific) |
| GO:0016655 | 0.215 | 4.351 | 0 | 1 | 21 | oxidoreductase activity, acting on NADH or NADPH, quinone or similar compound as acceptor |
| GO:0016298 | 0.216 | 2.362 | 1 | 2 | 76 | lipase activity |
| GO:0000976 | 0.220 | 2.330 | 1 | 2 | 77 | transcription regulatory region sequence-specific DNA binding |
| GO:0035257 | 0.224 | 4.143 | 0 | 1 | 22 | nuclear hormone receptor binding |
| GO:0019198 | 0.224 | 4.143 | 0 | 1 | 22 | transmembrane receptor protein phosphatase activity |
| GO:0001047 | 0.224 | 4.143 | 0 | 1 | 22 | core promoter binding |
| GO:0080025 | 0.224 | 4.143 | 0 | 1 | 22 | phosphatidylinositol-3,5-bisphosphate binding |
| GO:0051427 | 0.224 | 4.143 | 0 | 1 | 22 | hormone receptor binding |
| GO:0005001 | 0.224 | 4.143 | 0 | 1 | 22 | transmembrane receptor protein tyrosine phosphatase activity |
| GO:0016491 | 0.228 | 1.321 | 9 | 12 | 821 | oxidoreductase activity |
| GO:0003725 | 0.233 | 3.955 | 0 | 1 | 23 | double-stranded RNA binding |
| GO:0016405 | 0.233 | 3.955 | 0 | 1 | 23 | CoA-ligase activity |
| GO:0070011 | 0.237 | 1.365 | 7 | 9 | 593 | peptidase activity, acting on L-amino acid peptides |
| GO:0036094 | 0.240 | 1.201 | 26 | 29 | 2227 | small molecule binding |
| GO:0072509 | 0.242 | 2.183 | 1 | 2 | 82 | divalent inorganic cation transmembrane transporter activity |
| GO:0016706 | 0.242 | 3.782 | 0 | 1 | 24 | oxidoreductase activity, acting on paired donors, with incorporation or reduction of molecular oxygen, 2-oxoglutarate as one donor, and incorporation of one atom each of oxygen into both donors |
| GO:0008146 | 0.242 | 3.782 | 0 | 1 | 24 | sulfotransferase activity |
| GO:0000049 | 0.242 | 3.782 | 0 | 1 | 24 | tRNA binding |
| GO:0003676 | 0.245 | 1.199 | 25 | 28 | 2149 | nucleic acid binding |
| GO:0051540 | 0.246 | 2.156 | 1 | 2 | 83 | metal cluster binding |
| GO:0051536 | 0.246 | 2.156 | 1 | 2 | 83 | iron-sulfur cluster binding |
| GO:0004364 | 0.251 | 3.624 | 0 | 1 | 25 | glutathione transferase activity |
| GO:0008194 | 0.259 | 2.078 | 1 | 2 | 86 | UDP-glycosyltransferase activity |
| GO:0030551 | 0.259 | 3.479 | 0 | 1 | 26 | cyclic nucleotide binding |
| GO:0005272 | 0.276 | 3.220 | 0 | 1 | 28 | sodium channel activity |
| GO:0016878 | 0.276 | 3.220 | 0 | 1 | 28 | acid-thiol ligase activity |
| GO:0035091 | 0.280 | 1.960 | 1 | 2 | 91 | phosphatidylinositol binding |
| GO:0004252 | 0.280 | 1.660 | 2 | 3 | 161 | serine-type endopeptidase activity |
| GO:0042578 | 0.283 | 1.516 | 3 | 4 | 235 | phosphoric ester hydrolase activity |
| GO:0008374 | 0.284 | 3.105 | 0 | 1 | 29 | O-acyltransferase activity |
| GO:0016757 | 0.287 | 1.639 | 2 | 3 | 163 | transferase activity, transferring glycosyl groups |
| GO:0019901 | 0.301 | 2.897 | 0 | 1 | 31 | protein kinase binding |
| GO:0005516 | 0.301 | 2.897 | 0 | 1 | 31 | calmodulin binding |
| GO:0008168 | 0.309 | 1.569 | 2 | 3 | 170 | methyltransferase activity |
| GO:0016773 | 0.309 | 1.303 | 5 | 7 | 479 | phosphotransferase activity, alcohol group as acceptor |
| GO:0016702 | 0.309 | 2.803 | 0 | 1 | 32 | oxidoreductase activity, acting on single donors with incorporation of molecular oxygen, incorporation of two atoms of oxygen |
| GO:0003727 | 0.317 | 2.715 | 0 | 1 | 33 | single-stranded RNA binding |
| GO:0016877 | 0.317 | 2.715 | 0 | 1 | 33 | ligase activity, forming carbon-sulfur bonds |
| GO:0008234 | 0.318 | 1.778 | 1 | 2 | 100 | cysteine-type peptidase activity |
| GO:0016741 | 0.321 | 1.531 | 2 | 3 | 174 | transferase activity, transferring one-carbon groups |
| GO:0022890 | 0.321 | 1.421 | 3 | 4 | 250 | inorganic cation transmembrane transporter activity |
| GO:0051213 | 0.325 | 2.633 | 0 | 1 | 34 | dioxygenase activity |
| GO:0015020 | 0.325 | 2.633 | 0 | 1 | 34 | glucuronosyltransferase activity |
| GO:0005097 | 0.325 | 2.633 | 0 | 1 | 34 | Rab GTPase activator activity |
| GO:0008757 | 0.331 | 1.724 | 1 | 2 | 103 | S-adenosylmethionine-dependent methyltransferase activity |
| GO:0005083 | 0.337 | 1.487 | 2 | 3 | 179 | small GTPase regulator activity |
| GO:0008236 | 0.337 | 1.487 | 2 | 3 | 179 | serine-type peptidase activity |
| GO:0004190 | 0.339 | 1.691 | 1 | 2 | 105 | aspartic-type endopeptidase activity |
| GO:0070001 | 0.339 | 1.691 | 1 | 2 | 105 | aspartic-type peptidase activity |
| GO:0017171 | 0.343 | 1.470 | 2 | 3 | 181 | serine hydrolase activity |
| GO:0038024 | 0.348 | 2.412 | 0 | 1 | 37 | cargo receptor activity |
| GO:0016301 | 0.352 | 1.238 | 6 | 7 | 502 | kinase activity |
| GO:0003824 | 0.352 | 1.109 | 54 | 56 | 4686 | catalytic activity |
| GO:0030695 | 0.353 | 1.353 | 3 | 4 | 262 | GTPase regulator activity |
| GO:0015151 | 0.355 | 2.347 | 0 | 1 | 38 | alpha-glucoside transmembrane transporter activity |
| GO:0015574 | 0.355 | 2.347 | 0 | 1 | 38 | trehalose transmembrane transporter activity |
| GO:0042947 | 0.355 | 2.347 | 0 | 1 | 38 | glucoside transmembrane transporter activity |
| GO:0004175 | 0.358 | 1.255 | 5 | 6 | 424 | endopeptidase activity |
| GO:0003713 | 0.360 | 1.611 | 1 | 2 | 110 | transcription coactivator activity |
| GO:0060589 | 0.361 | 1.337 | 3 | 4 | 265 | nucleoside-triphosphatase regulator activity |
| GO:0015154 | 0.363 | 2.285 | 0 | 1 | 39 | disaccharide transmembrane transporter activity |
| GO:0005488 | 0.368 | 1.126 | 71 | 73 | 6201 | binding |
| GO:0016597 | 0.370 | 2.226 | 0 | 1 | 40 | amino acid binding |
| GO:0004518 | 0.374 | 1.311 | 3 | 4 | 270 | nuclease activity |
| GO:0004721 | 0.377 | 1.553 | 1 | 2 | 114 | phosphoprotein phosphatase activity |
| GO:0005200 | 0.384 | 2.117 | 0 | 1 | 42 | structural constituent of cytoskeleton |
| GO:0000988 | 0.390 | 1.353 | 2 | 3 | 196 | protein binding transcription factor activity |
| GO:0016779 | 0.392 | 1.276 | 3 | 4 | 277 | nucleotidyltransferase activity |
| GO:0008080 | 0.398 | 2.018 | 1 | 1 | 44 | N-acetyltransferase activity |
| GO:0030246 | 0.412 | 1.304 | 2 | 3 | 203 | carbohydrate binding |
| GO:0019887 | 0.412 | 1.928 | 1 | 1 | 46 | protein kinase regulator activity |
| GO:0046873 | 0.418 | 1.291 | 2 | 3 | 205 | metal ion transmembrane transporter activity |
| GO:0019904 | 0.419 | 1.886 | 1 | 1 | 47 | protein domain specific binding |
| GO:0005249 | 0.419 | 1.886 | 1 | 1 | 47 | voltage-gated potassium channel activity |
| GO:0004221 | 0.419 | 1.886 | 1 | 1 | 47 | ubiquitin thiolesterase activity |
| GO:0080030 | 0.419 | 1.886 | 1 | 1 | 47 | methyl indole-3-acetate esterase activity |
| GO:0080031 | 0.419 | 1.886 | 1 | 1 | 47 | methyl salicylate esterase activity |
| GO:0080032 | 0.419 | 1.886 | 1 | 1 | 47 | methyl jasmonate esterase activity |
| GO:0016651 | 0.419 | 1.886 | 1 | 1 | 47 | oxidoreductase activity, acting on NADH or NADPH |
| GO:0003682 | 0.428 | 1.272 | 2 | 3 | 208 | chromatin binding |
| GO:0022892 | 0.429 | 1.126 | 7 | 8 | 627 | substrate-specific transporter activity |
| GO:0018024 | 0.432 | 1.807 | 1 | 1 | 49 | histone-lysine N-methyltransferase activity |
| GO:0005102 | 0.433 | 1.378 | 1 | 2 | 128 | receptor binding |
| GO:0016772 | 0.434 | 1.105 | 9 | 10 | 799 | transferase activity, transferring phosphorus-containing groups |
| GO:0005096 | 0.437 | 1.367 | 1 | 2 | 129 | GTPase activator activity |
| GO:0016410 | 0.439 | 1.770 | 1 | 1 | 50 | N-acyltransferase activity |
| GO:0016278 | 0.439 | 1.770 | 1 | 1 | 50 | lysine N-methyltransferase activity |
| GO:0016279 | 0.439 | 1.770 | 1 | 1 | 50 | protein-lysine N-methyltransferase activity |
| GO:0015662 | 0.439 | 1.770 | 1 | 1 | 50 | ATPase activity, coupled to transmembrane movement of ions, phosphorylative mechanism |
| GO:0016811 | 0.439 | 1.770 | 1 | 1 | 50 | hydrolase activity, acting on carbon-nitrogen (but not peptide) bonds, in linear amides |
| GO:0005085 | 0.441 | 1.356 | 1 | 2 | 130 | guanyl-nucleotide exchange factor activity |
| GO:0051287 | 0.445 | 1.734 | 1 | 1 | 51 | NAD binding |
| GO:0015078 | 0.445 | 1.734 | 1 | 1 | 51 | hydrogen ion transmembrane transporter activity |
| GO:0000166 | 0.457 | 1.050 | 24 | 25 | 2106 | nucleotide binding |
| GO:0097159 | 0.457 | 1.050 | 24 | 25 | 2106 | organic cyclic compound binding |
| GO:1901265 | 0.457 | 1.050 | 24 | 25 | 2106 | nucleoside phosphate binding |
| GO:0008201 | 0.458 | 1.667 | 1 | 1 | 53 | heparin binding |
| GO:0003743 | 0.464 | 1.635 | 1 | 1 | 54 | translation initiation factor activity |
| GO:0005099 | 0.470 | 1.605 | 1 | 1 | 55 | Ras GTPase activator activity |
| GO:0008324 | 0.477 | 1.134 | 4 | 4 | 310 | cation transmembrane transporter activity |
| GO:0005261 | 0.479 | 1.256 | 2 | 2 | 140 | cation channel activity |
| GO:0003677 | 0.482 | 1.045 | 14 | 15 | 1262 | DNA binding |
| GO:0005262 | 0.483 | 1.547 | 1 | 1 | 57 | calcium channel activity |
| GO:0015144 | 0.483 | 1.547 | 1 | 1 | 57 | carbohydrate transmembrane transporter activity |
| GO:0016765 | 0.483 | 1.547 | 1 | 1 | 57 | transferase activity, transferring alkyl or aryl (other than methyl) groups |
| GO:0016891 | 0.483 | 1.547 | 1 | 1 | 57 | endoribonuclease activity, producing 5'-phosphomonoesters |
| GO:0042054 | 0.483 | 1.547 | 1 | 1 | 57 | histone methyltransferase activity |
| GO:0051119 | 0.483 | 1.547 | 1 | 1 | 57 | sugar transmembrane transporter activity |
| GO:0042277 | 0.489 | 1.520 | 1 | 1 | 58 | peptide binding |
| GO:0008565 | 0.500 | 1.468 | 1 | 1 | 60 | protein transporter activity |
| GO:0051539 | 0.500 | 1.468 | 1 | 1 | 60 | 4 iron, 4 sulfur cluster binding |
| GO:0005267 | 0.500 | 1.468 | 1 | 1 | 60 | potassium channel activity |
| GO:0042625 | 0.500 | 1.468 | 1 | 1 | 60 | ATPase activity, coupled to transmembrane movement of ions |
| GO:0004812 | 0.500 | 1.468 | 1 | 1 | 60 | aminoacyl-tRNA ligase activity |
| GO:0016875 | 0.500 | 1.468 | 1 | 1 | 60 | ligase activity, forming carbon-oxygen bonds |
| GO:0016876 | 0.500 | 1.468 | 1 | 1 | 60 | ligase activity, forming aminoacyl-tRNA and related compounds |
| GO:0016701 | 0.506 | 1.443 | 1 | 1 | 61 | oxidoreductase activity, acting on single donors with incorporation of molecular oxygen |
| GO:0016893 | 0.506 | 1.443 | 1 | 1 | 61 | endonuclease activity, active with either ribo- or deoxyribonucleic acids and producing 5'-phosphomonoesters |
| GO:0016829 | 0.508 | 1.186 | 2 | 2 | 148 | lyase activity |
| GO:0003964 | 0.523 | 1.154 | 2 | 2 | 152 | RNA-directed DNA polymerase activity |
| GO:0004091 | 0.523 | 1.374 | 1 | 1 | 64 | carboxylesterase activity |
| GO:0050253 | 0.523 | 1.374 | 1 | 1 | 64 | retinyl-palmitate esterase activity |
| GO:0004867 | 0.523 | 1.374 | 1 | 1 | 64 | serine-type endopeptidase inhibitor activity |
| GO:0005539 | 0.528 | 1.352 | 1 | 1 | 65 | glycosaminoglycan binding |
| GO:0004527 | 0.539 | 1.311 | 1 | 1 | 67 | exonuclease activity |
| GO:0016903 | 0.560 | 1.235 | 1 | 1 | 71 | oxidoreductase activity, acting on the aldehyde or oxo group of donors |
| GO:0008276 | 0.560 | 1.235 | 1 | 1 | 71 | protein methyltransferase activity |
| GO:0004521 | 0.565 | 1.218 | 1 | 1 | 72 | endoribonuclease activity |
| GO:0005215 | 0.568 | 0.985 | 10 | 10 | 885 | transporter activity |
| GO:0004725 | 0.570 | 1.201 | 1 | 1 | 73 | protein tyrosine phosphatase activity |
| GO:0008134 | 0.570 | 1.201 | 1 | 1 | 73 | transcription factor binding |
| GO:0003723 | 0.571 | 0.983 | 9 | 9 | 798 | RNA binding |
| GO:0004872 | 0.579 | 0.984 | 5 | 5 | 443 | receptor activity |
| GO:0015081 | 0.580 | 1.168 | 1 | 1 | 75 | sodium ion transmembrane transporter activity |
| GO:0022843 | 0.580 | 1.168 | 1 | 1 | 75 | voltage-gated cation channel activity |
| GO:0015399 | 0.587 | 1.022 | 2 | 2 | 171 | primary active transmembrane transporter activity |
| GO:0015405 | 0.587 | 1.022 | 2 | 2 | 171 | P-P-bond-hydrolysis-driven transmembrane transporter activity |
| GO:0022891 | 0.596 | 0.962 | 6 | 6 | 543 | substrate-specific transmembrane transporter activity |
| GO:0016810 | 0.599 | 1.108 | 1 | 1 | 79 | hydrolase activity, acting on carbon-nitrogen (but not peptide) bonds |
| GO:0015077 | 0.603 | 0.992 | 2 | 2 | 176 | monovalent inorganic cation transmembrane transporter activity |
| GO:0015079 | 0.608 | 1.080 | 1 | 1 | 81 | potassium ion transmembrane transporter activity |
| GO:0003712 | 0.612 | 0.975 | 2 | 2 | 179 | transcription cofactor activity |
| GO:0004866 | 0.613 | 1.066 | 1 | 1 | 82 | endopeptidase inhibitor activity |
| GO:0030414 | 0.617 | 1.053 | 1 | 1 | 83 | peptidase inhibitor activity |
| GO:0008135 | 0.617 | 1.053 | 1 | 1 | 83 | translation factor activity, nucleic acid binding |
| GO:0004674 | 0.620 | 0.943 | 3 | 3 | 277 | protein serine/threonine kinase activity |
| GO:0016791 | 0.621 | 0.958 | 2 | 2 | 182 | phosphatase activity |
| GO:0004842 | 0.627 | 0.947 | 2 | 2 | 184 | ubiquitin-protein ligase activity |
| GO:0061135 | 0.631 | 1.016 | 1 | 1 | 86 | endopeptidase regulator activity |
| GO:0034061 | 0.636 | 0.932 | 2 | 2 | 187 | DNA polymerase activity |
| GO:0016787 | 0.636 | 0.944 | 26 | 25 | 2276 | hydrolase activity |
| GO:0000989 | 0.639 | 0.926 | 2 | 2 | 188 | transcription factor binding transcription factor activity |
| GO:0004672 | 0.641 | 0.912 | 4 | 4 | 381 | protein kinase activity |
| GO:0019787 | 0.648 | 0.911 | 2 | 2 | 191 | small conjugating protein ligase activity |
| GO:0004386 | 0.656 | 0.897 | 2 | 2 | 194 | helicase activity |
| GO:0005506 | 0.663 | 0.883 | 3 | 3 | 295 | iron ion binding |
| GO:0061134 | 0.667 | 0.917 | 1 | 1 | 95 | peptidase regulator activity |
| GO:0005244 | 0.690 | 0.862 | 1 | 1 | 101 | voltage-gated ion channel activity |
| GO:0022832 | 0.690 | 0.862 | 1 | 1 | 101 | voltage-gated channel activity |
| GO:0004540 | 0.690 | 0.862 | 1 | 1 | 101 | ribonuclease activity |
| GO:0016881 | 0.703 | 0.818 | 2 | 2 | 212 | acid-amino acid ligase activity |
| GO:0032559 | 0.730 | 0.870 | 17 | 15 | 1469 | adenyl ribonucleotide binding |
| GO:0030554 | 0.730 | 0.870 | 17 | 15 | 1469 | adenyl nucleotide binding |
| GO:0003735 | 0.730 | 0.768 | 1 | 1 | 113 | structural constituent of ribosome |
| GO:0008238 | 0.737 | 0.754 | 1 | 1 | 115 | exopeptidase activity |
| GO:0015075 | 0.745 | 0.788 | 5 | 4 | 437 | ion transmembrane transporter activity |
| GO:0001871 | 0.746 | 0.735 | 1 | 1 | 118 | pattern binding |
| GO:0030247 | 0.746 | 0.735 | 1 | 1 | 118 | polysaccharide binding |
| GO:0008026 | 0.749 | 0.729 | 1 | 1 | 119 | ATP-dependent helicase activity |
| GO:0070035 | 0.749 | 0.729 | 1 | 1 | 119 | purine NTP-dependent helicase activity |
| GO:0005216 | 0.750 | 0.745 | 3 | 2 | 232 | ion channel activity |
| GO:0022838 | 0.750 | 0.745 | 3 | 2 | 232 | substrate-specific channel activity |
| GO:0004857 | 0.754 | 0.716 | 1 | 1 | 121 | enzyme inhibitor activity |
| GO:0003779 | 0.757 | 0.710 | 1 | 1 | 122 | actin binding |
| GO:0015267 | 0.758 | 0.732 | 3 | 2 | 236 | channel activity |
| GO:0022803 | 0.758 | 0.732 | 3 | 2 | 236 | passive transmembrane transporter activity |
| GO:0004930 | 0.774 | 0.676 | 1 | 1 | 128 | G-protein coupled receptor activity |
| GO:0003774 | 0.789 | 0.645 | 2 | 1 | 134 | motor activity |
| GO:0016879 | 0.792 | 0.681 | 3 | 2 | 253 | ligase activity, forming carbon-nitrogen bonds |
| GO:0016705 | 0.793 | 0.678 | 3 | 2 | 254 | oxidoreductase activity, acting on paired donors, with incorporation or reduction of molecular oxygen |
| GO:0005524 | 0.802 | 0.811 | 17 | 14 | 1454 | ATP binding |
| GO:0022857 | 0.823 | 0.730 | 8 | 6 | 699 | transmembrane transporter activity |
| GO:0017076 | 0.824 | 0.802 | 19 | 16 | 1669 | purine nucleotide binding |
| GO:0032553 | 0.824 | 0.802 | 19 | 16 | 1669 | ribonucleotide binding |
| GO:0032555 | 0.824 | 0.802 | 19 | 16 | 1669 | purine ribonucleotide binding |
| GO:0022836 | 0.852 | 0.524 | 2 | 1 | 164 | gated channel activity |
| GO:0043492 | 0.857 | 0.515 | 2 | 1 | 167 | ATPase activity, coupled to movement of substances |
| GO:0042626 | 0.857 | 0.515 | 2 | 1 | 167 | ATPase activity, coupled to transmembrane movement of substances |
| GO:0004888 | 0.857 | 0.580 | 3 | 2 | 295 | transmembrane signaling receptor activity |
| GO:0016820 | 0.863 | 0.502 | 2 | 1 | 171 | hydrolase activity, acting on acid anhydrides, catalyzing transmembrane movement of substances |
| GO:0035639 | 0.876 | 0.751 | 19 | 15 | 1652 | purine ribonucleoside triphosphate binding |
| GO:0022804 | 0.890 | 0.528 | 4 | 2 | 323 | active transmembrane transporter activity |
| GO:0038023 | 0.894 | 0.521 | 4 | 2 | 327 | signaling receptor activity |
| GO:0016798 | 0.896 | 0.441 | 2 | 1 | 194 | hydrolase activity, acting on glycosyl bonds |
| GO:0004871 | 0.897 | 0.564 | 5 | 3 | 451 | signal transducer activity |
| GO:0060089 | 0.897 | 0.564 | 5 | 3 | 451 | molecular transducer activity |
| GO:0005525 | 0.924 | 0.386 | 3 | 1 | 221 | GTP binding |
| GO:0042623 | 0.925 | 0.467 | 4 | 2 | 363 | ATPase activity, coupled |
| GO:0005198 | 0.928 | 0.461 | 4 | 2 | 367 | structural molecule activity |
| GO:0009055 | 0.932 | 0.370 | 3 | 1 | 230 | electron carrier activity |
| GO:0004497 | 0.934 | 0.367 | 3 | 1 | 232 | monooxygenase activity |
| GO:0019001 | 0.935 | 0.364 | 3 | 1 | 234 | guanyl nucleotide binding |
| GO:0032561 | 0.935 | 0.364 | 3 | 1 | 234 | guanyl ribonucleotide binding |
| GO:0020037 | 0.937 | 0.360 | 3 | 1 | 236 | heme binding |
| GO:0046906 | 0.940 | 0.354 | 3 | 1 | 240 | tetrapyrrole binding |
| GO:0008092 | 0.941 | 0.351 | 3 | 1 | 242 | cytoskeletal protein binding |
| GO:0016887 | 0.976 | 0.351 | 5 | 2 | 475 | ATPase activity |
| GO:0017111 | 0.983 | 0.418 | 9 | 4 | 784 | nucleoside-triphosphatase activity |
| GO:0016462 | 0.986 | 0.406 | 9 | 4 | 804 | pyrophosphatase activity |
| GO:0016818 | 0.986 | 0.406 | 9 | 4 | 805 | hydrolase activity, acting on acid anhydrides, in phosphorus-containing anhydrides |
| GO:0016817 | 0.987 | 0.403 | 9 | 4 | 810 | hydrolase activity, acting on acid anhydrides |
