# Supplementary material for: Inducible Defenses Stay Up Late: Temporal Patterns of Immune Gene Expression in Tenebrio molitor
Source: G3 (Bethesda). 2014 Jun 1;4(6):947–55. doi: 10.1534/g3.113.008516 (PMC4065263; doi:10.1534/g3.113.008516)
Supplement: Supporting Information [file supp_g3.113.008516_FigureS1.pdf]

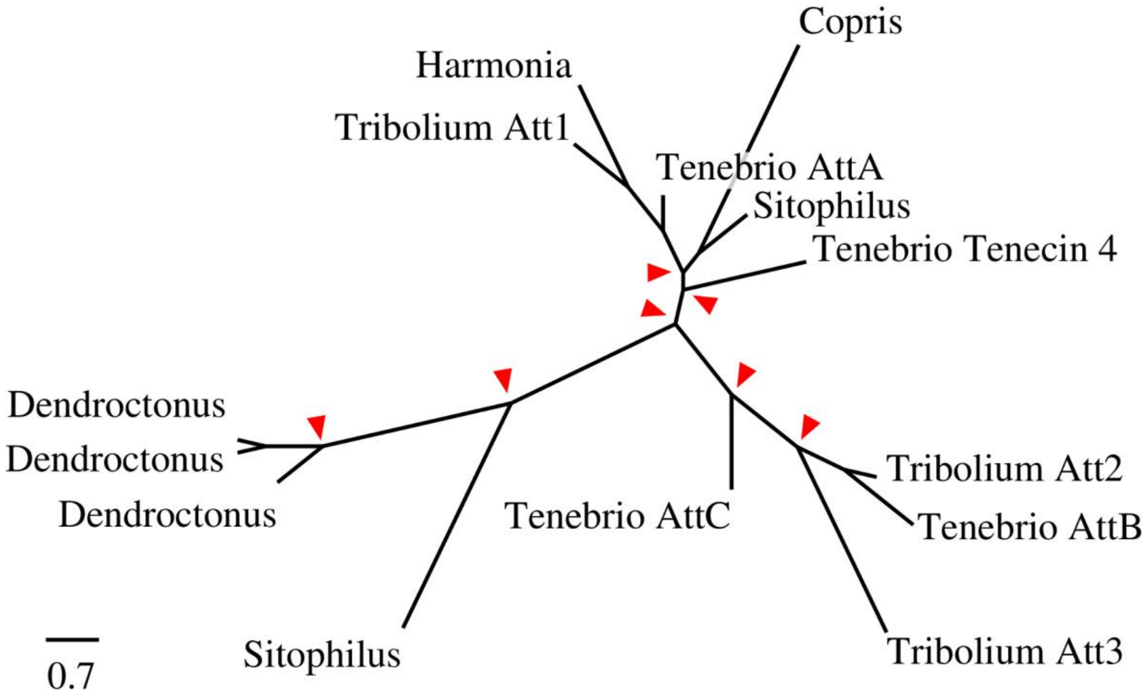

Figure S1 Maximum likelihood phylogenetic tree showing relationships among beetle attacins. Protein sequences were aligned using MUSCLE and Gblocks. Trees were constructed usin PhyML and TreeDyn using phylogeny.fr webserver. Red arrows indicate a confidence index > 0.8.
