# Supplementary material for: Inducible Defenses Stay Up Late: Temporal Patterns of Immune Gene Expression in Tenebrio molitor
Source: G3 (Bethesda). 2014 Jun 1;4(6):947–55. doi: 10.1534/g3.113.008516 (PMC4065263; doi:10.1534/g3.113.008516)
Supplement: Supporting Information [file supp_g3.113.008516_FigureS3.pdf]

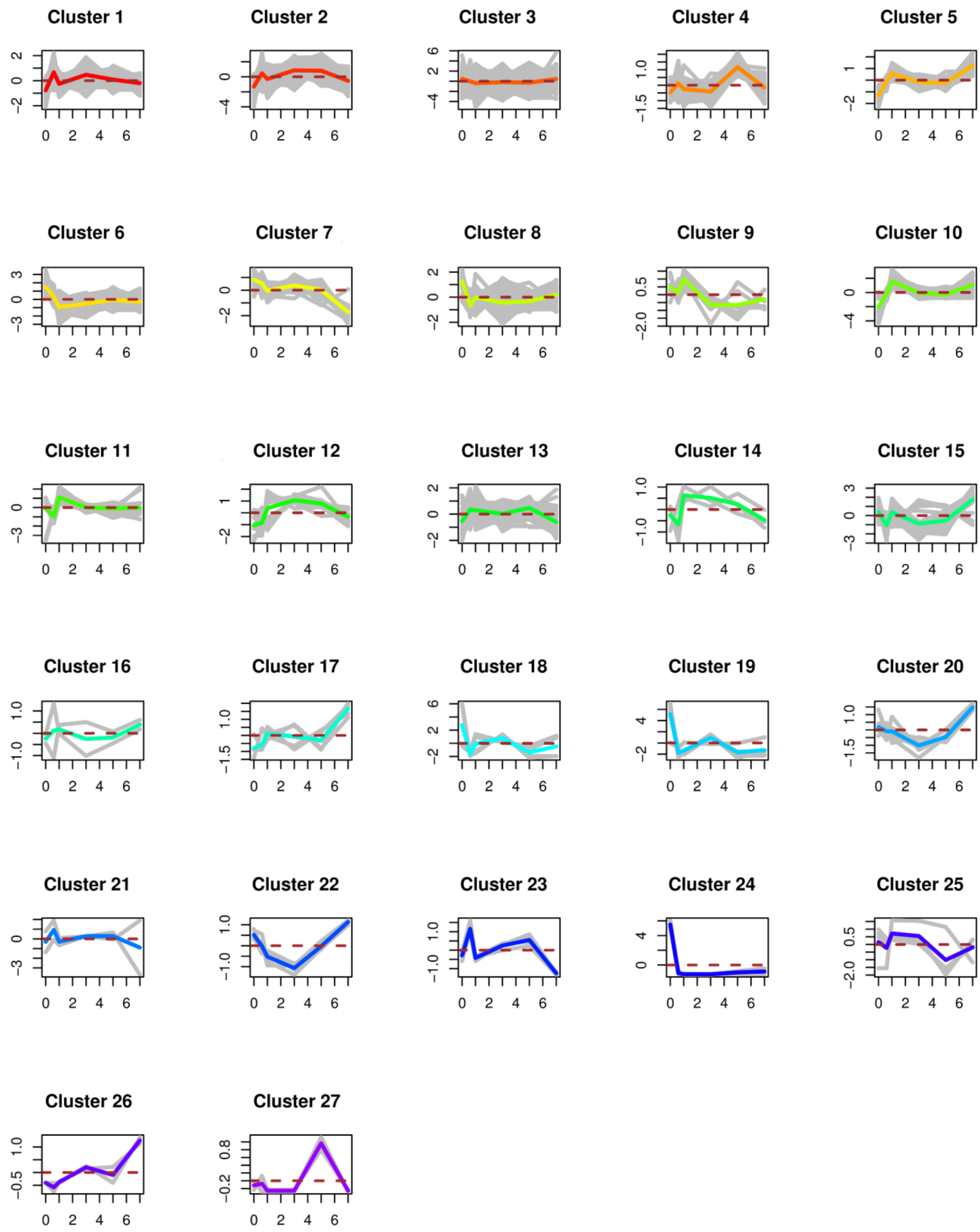

Figure S3 Gene expression clusters produced by the R package DIRECT. Vertical axes represent median centered log2 FPKM whereas horizontal axes represent days post immune challenge. Colored lines depict the median profile for each cluster.
