# Supplementary material for: Inducible Defenses Stay Up Late: Temporal Patterns of Immune Gene Expression in Tenebrio molitor
Source: G3 (Bethesda). 2014 Jun 1;4(6):947–55. doi: 10.1534/g3.113.008516 (PMC4065263; doi:10.1534/g3.113.008516)
Supplement: Supporting Information [file supp_g3.113.008516_TableS1.pdf]

Table S1 Comparison of trinity assemblies utilizing illumina and/or 454 reads. sim PE, simulated paired-end; USCO, universal single-copy ortholog.

Assembly	Components	Transcripts	N50	N90	Orthologs	USCOs
Illumina	44811	96330	2545	571	8955	3071
454 (newbler)*	16647*	22507	1567	559	7628	2907
454 sim PE	25649	58060	696	306	6557	2465
Illumina + 454	45676	103597	2183	516	8830	2999
Illumina + 454 sim PE	49410	122521	1745	448	9370	3120
Illumina + 454 (newbler)*	54099*	118322	742	168	9057	3107
454	13724	25374	1097	434	6876	2646

*assembly newbler included for reference. Newbler does not compute components and the number of isogroups is reported instead. Orthologs, the number of putative orthologs between the assembly and the *Tribolium castaneum* proteome. In each assembly Illumina data was first digitally normalized.
