# Supplementary material for: Inducible Defenses Stay Up Late: Temporal Patterns of Immune Gene Expression in Tenebrio molitor
Source: G3 (Bethesda). 2014 Jun 1;4(6):947–55. doi: 10.1534/g3.113.008516 (PMC4065263; doi:10.1534/g3.113.008516)
Supplement: Supporting Information [file supp_g3.113.008516_TableS2.pdf]

Table S2 Summary statistics for each of the data sets used in *de novo* assembly. sim PE, simulated paired-end; diginorm, digitally normalized.

Data set	Reads	Mean length (bp)	File size (GB)
454	1,151,456	285	0.695[a]
Illumina*	95,339,034	101	22.8[a]
454 sim PE	7,367,318**	76	0.715[b]
Illumina diginorm	9,639,354	83	1.2[b]

*all assemblies utilized digitally normalized illulmina data. The properties of the original data are included here for reference.
**refers to number of read pairs
[a]fastq file
[b]fasta file
