# Supplementary material for: Inducible Defenses Stay Up Late: Temporal Patterns of Immune Gene Expression in Tenebrio molitor
Source: G3 (Bethesda). 2014 Jun 1;4(6):947–55. doi: 10.1534/g3.113.008516 (PMC4065263; doi:10.1534/g3.113.008516)
Supplement: Supporting Information [file supp_g3.113.008516_TableS11.html]

TableS11 

# Inducible Defenses Stay Up Late: Temporal Patterns of Immune Gene Expression in *Tenebrio molitor*

Gene to GO BP test for over-representation

| GOBPID | Pvalue | OddsRatio | ExpCount | Count | Size | Term |
| GO:0050789 | 0.000 | 2.452 | 85 | 123 | 3856 | regulation of biological process |
| GO:0065007 | 0.000 | 2.422 | 91 | 128 | 4147 | biological regulation |
| GO:0019222 | 0.000 | 2.273 | 49 | 82 | 2232 | regulation of metabolic process |
| GO:0050794 | 0.000 | 2.238 | 79 | 114 | 3590 | regulation of cellular process |
| GO:0060255 | 0.000 | 2.240 | 42 | 72 | 1905 | regulation of macromolecule metabolic process |
| GO:0060042 | 0.000 | 27.562 | 0 | 6 | 16 | retina morphogenesis in camera-type eye |
| GO:0031323 | 0.000 | 2.144 | 44 | 73 | 2002 | regulation of cellular metabolic process |
| GO:0080090 | 0.000 | 2.128 | 43 | 71 | 1944 | regulation of primary metabolic process |
| GO:0010842 | 0.000 | 38.082 | 0 | 5 | 11 | retina layer formation |
| GO:0032268 | 0.000 | 2.943 | 11 | 29 | 519 | regulation of cellular protein metabolic process |
| GO:0010604 | 0.000 | 2.572 | 17 | 37 | 768 | positive regulation of macromolecule metabolic process |
| GO:0051246 | 0.000 | 2.738 | 13 | 31 | 596 | regulation of protein metabolic process |
| GO:0044267 | 0.000 | 2.114 | 36 | 61 | 1621 | cellular protein metabolic process |
| GO:0048518 | 0.000 | 2.132 | 33 | 58 | 1516 | positive regulation of biological process |
| GO:0009893 | 0.000 | 2.418 | 19 | 39 | 860 | positive regulation of metabolic process |
| GO:0048593 | 0.000 | 16.199 | 1 | 6 | 23 | camera-type eye morphogenesis |
| GO:0043412 | 0.000 | 2.167 | 26 | 48 | 1197 | macromolecule modification |
| GO:0048522 | 0.000 | 2.110 | 30 | 52 | 1343 | positive regulation of cellular process |
| GO:0031325 | 0.000 | 2.340 | 18 | 36 | 808 | positive regulation of cellular metabolic process |
| GO:0048523 | 0.000 | 2.070 | 28 | 49 | 1273 | negative regulation of cellular process |
| GO:0006464 | 0.000 | 2.121 | 25 | 45 | 1131 | cellular protein modification process |
| GO:0036211 | 0.000 | 2.121 | 25 | 45 | 1131 | protein modification process |
| GO:0003407 | 0.000 | 16.305 | 0 | 5 | 19 | neural retina development |
| GO:0031399 | 0.000 | 2.940 | 8 | 21 | 365 | regulation of protein modification process |
| GO:0048519 | 0.000 | 1.996 | 32 | 53 | 1436 | negative regulation of biological process |
| GO:0031401 | 0.000 | 3.435 | 5 | 16 | 237 | positive regulation of protein modification process |
| GO:0060560 | 0.000 | 5.172 | 2 | 10 | 100 | developmental growth involved in morphogenesis |
| GO:0032270 | 0.000 | 3.181 | 6 | 17 | 271 | positive regulation of cellular protein metabolic process |
| GO:0009892 | 0.000 | 2.323 | 14 | 29 | 640 | negative regulation of metabolic process |
| GO:0051171 | 0.000 | 1.881 | 34 | 54 | 1538 | regulation of nitrogen compound metabolic process |
| GO:0010605 | 0.000 | 2.326 | 14 | 28 | 615 | negative regulation of macromolecule metabolic process |
| GO:0051247 | 0.000 | 2.998 | 6 | 17 | 286 | positive regulation of protein metabolic process |
| GO:0010468 | 0.000 | 1.847 | 34 | 53 | 1527 | regulation of gene expression |
| GO:0010557 | 0.000 | 2.441 | 10 | 23 | 476 | positive regulation of macromolecule biosynthetic process |
| GO:0016070 | 0.000 | 1.759 | 43 | 64 | 1973 | RNA metabolic process |
| GO:0046209 | 0.000 | 15.136 | 0 | 4 | 16 | nitric oxide metabolic process |
| GO:0003344 | 0.000 | 33.897 | 0 | 3 | 7 | pericardium morphogenesis |
| GO:0009889 | 0.000 | 1.838 | 32 | 51 | 1467 | regulation of biosynthetic process |
| GO:0010556 | 0.000 | 1.854 | 31 | 49 | 1392 | regulation of macromolecule biosynthetic process |
| GO:0051147 | 0.000 | 7.228 | 1 | 6 | 44 | regulation of muscle cell differentiation |
| GO:0031324 | 0.000 | 2.292 | 12 | 25 | 551 | negative regulation of cellular metabolic process |
| GO:0060041 | 0.000 | 7.042 | 1 | 6 | 45 | retina development in camera-type eye |
| GO:0009891 | 0.000 | 2.274 | 12 | 25 | 555 | positive regulation of biosynthetic process |
| GO:0044260 | 0.000 | 1.665 | 80 | 102 | 3617 | cellular macromolecule metabolic process |
| GO:0048596 | 0.000 | Inf | 0 | 2 | 2 | embryonic camera-type eye morphogenesis |
| GO:0060059 | 0.000 | Inf | 0 | 2 | 2 | embryonic retina morphogenesis in camera-type eye |
| GO:0051252 | 0.000 | 1.850 | 29 | 46 | 1298 | regulation of RNA metabolic process |
| GO:0019538 | 0.001 | 1.703 | 49 | 69 | 2207 | protein metabolic process |
| GO:0048589 | 0.001 | 2.915 | 6 | 15 | 257 | developmental growth |
| GO:0048134 | 0.001 | 12.970 | 0 | 4 | 18 | germ-line cyst formation |
| GO:0043086 | 0.001 | 3.552 | 3 | 11 | 155 | negative regulation of catalytic activity |
| GO:0012501 | 0.001 | 2.129 | 15 | 28 | 665 | programmed cell death |
| GO:0008283 | 0.001 | 2.220 | 12 | 25 | 567 | cell proliferation |
| GO:0051130 | 0.001 | 3.084 | 5 | 13 | 210 | positive regulation of cellular component organization |
| GO:0010467 | 0.001 | 1.661 | 54 | 74 | 2440 | gene expression |
| GO:0070507 | 0.001 | 6.238 | 1 | 6 | 50 | regulation of microtubule cytoskeleton organization |
| GO:0019219 | 0.001 | 1.762 | 33 | 51 | 1517 | regulation of nucleobase-containing compound metabolic process |
| GO:0010559 | 0.001 | 22.593 | 0 | 3 | 9 | regulation of glycoprotein biosynthetic process |
| GO:0032502 | 0.001 | 1.633 | 63 | 84 | 2873 | developmental process |
| GO:0051094 | 0.001 | 3.158 | 4 | 12 | 189 | positive regulation of developmental process |
| GO:0008219 | 0.001 | 2.032 | 16 | 29 | 720 | cell death |
| GO:0007026 | 0.001 | 10.677 | 0 | 4 | 21 | negative regulation of microtubule depolymerization |
| GO:0016265 | 0.001 | 2.025 | 16 | 29 | 722 | death |
| GO:0016202 | 0.001 | 5.837 | 1 | 6 | 53 | regulation of striated muscle tissue development |
| GO:0048634 | 0.001 | 5.837 | 1 | 6 | 53 | regulation of muscle organ development |
| GO:0031326 | 0.001 | 1.751 | 32 | 49 | 1457 | regulation of cellular biosynthetic process |
| GO:2000112 | 0.001 | 1.766 | 30 | 47 | 1381 | regulation of cellular macromolecule biosynthetic process |
| GO:0030334 | 0.001 | 3.507 | 3 | 10 | 142 | regulation of cell migration |
| GO:0060039 | 0.001 | 19.363 | 0 | 3 | 10 | pericardium development |
| GO:0033043 | 0.001 | 2.686 | 6 | 15 | 277 | regulation of organelle organization |
| GO:0051270 | 0.001 | 3.211 | 4 | 11 | 170 | regulation of cellular component movement |
| GO:0010629 | 0.001 | 2.291 | 10 | 20 | 434 | negative regulation of gene expression |
| GO:0007160 | 0.001 | 4.653 | 2 | 7 | 76 | cell-matrix adhesion |
| GO:0007044 | 0.001 | 6.901 | 1 | 5 | 38 | cell-substrate junction assembly |
| GO:0031589 | 0.001 | 4.089 | 2 | 8 | 98 | cell-substrate adhesion |
| GO:0090141 | 0.001 | 89.922 | 0 | 2 | 3 | positive regulation of mitochondrial fission |
| GO:0042062 | 0.001 | 89.922 | 0 | 2 | 3 | long-term strengthening of neuromuscular junction |
| GO:2000145 | 0.001 | 3.377 | 3 | 10 | 147 | regulation of cell motility |
| GO:0051248 | 0.001 | 3.150 | 4 | 11 | 173 | negative regulation of protein metabolic process |
| GO:0000122 | 0.001 | 2.969 | 4 | 12 | 200 | negative regulation of transcription from RNA polymerase II promoter |
| GO:0023056 | 0.001 | 2.603 | 6 | 15 | 285 | positive regulation of signaling |
| GO:0051128 | 0.002 | 1.983 | 16 | 28 | 708 | regulation of cellular component organization |
| GO:0050790 | 0.002 | 2.121 | 12 | 23 | 540 | regulation of catalytic activity |
| GO:0048638 | 0.002 | 3.956 | 2 | 8 | 101 | regulation of developmental growth |
| GO:0031111 | 0.002 | 9.072 | 1 | 4 | 24 | negative regulation of microtubule polymerization or depolymerization |
| GO:0065009 | 0.002 | 1.996 | 15 | 27 | 677 | regulation of molecular function |
| GO:0032886 | 0.002 | 5.173 | 1 | 6 | 59 | regulation of microtubule-based process |
| GO:0072358 | 0.002 | 2.387 | 8 | 17 | 352 | cardiovascular system development |
| GO:0072359 | 0.002 | 2.387 | 8 | 17 | 352 | circulatory system development |
| GO:0043170 | 0.002 | 1.571 | 91 | 111 | 4154 | macromolecule metabolic process |
| GO:0045765 | 0.002 | 6.152 | 1 | 5 | 42 | regulation of angiogenesis |
| GO:0006355 | 0.002 | 1.726 | 27 | 42 | 1241 | regulation of transcription, DNA-dependent |
| GO:0031114 | 0.002 | 8.246 | 1 | 4 | 26 | regulation of microtubule depolymerization |
| GO:2001141 | 0.002 | 1.723 | 27 | 42 | 1243 | regulation of RNA biosynthetic process |
| GO:0031327 | 0.002 | 2.208 | 9 | 19 | 425 | negative regulation of cellular biosynthetic process |
| GO:0006376 | 0.003 | 13.549 | 0 | 3 | 13 | mRNA splice site selection |
| GO:0006809 | 0.003 | 13.549 | 0 | 3 | 13 | nitric oxide biosynthetic process |
| GO:0002011 | 0.003 | 13.549 | 0 | 3 | 13 | morphogenesis of an epithelial sheet |
| GO:0023052 | 0.003 | 1.604 | 43 | 60 | 1961 | signaling |
| GO:0006796 | 0.003 | 2.010 | 13 | 24 | 593 | phosphate-containing compound metabolic process |
| GO:0030154 | 0.003 | 1.630 | 38 | 54 | 1719 | cell differentiation |
| GO:0006793 | 0.003 | 2.006 | 13 | 24 | 594 | phosphorus metabolic process |
| GO:0018988 | 0.003 | 5.835 | 1 | 5 | 44 | molting cycle, protein-based cuticle |
| GO:0009890 | 0.003 | 2.191 | 9 | 19 | 428 | negative regulation of biosynthetic process |
| GO:0016556 | 0.003 | 44.955 | 0 | 2 | 4 | mRNA modification |
| GO:0042983 | 0.003 | 44.955 | 0 | 2 | 4 | amyloid precursor protein biosynthetic process |
| GO:0042984 | 0.003 | 44.955 | 0 | 2 | 4 | regulation of amyloid precursor protein biosynthetic process |
| GO:0007394 | 0.003 | 44.955 | 0 | 2 | 4 | dorsal closure, elongation of leading edge cells |
| GO:0030721 | 0.003 | 44.955 | 0 | 2 | 4 | spectrosome organization |
| GO:0002385 | 0.003 | 44.955 | 0 | 2 | 4 | mucosal immune response |
| GO:0002251 | 0.003 | 44.955 | 0 | 2 | 4 | organ or tissue specific immune response |
| GO:0090140 | 0.003 | 44.955 | 0 | 2 | 4 | regulation of mitochondrial fission |
| GO:0048856 | 0.003 | 1.558 | 54 | 72 | 2468 | anatomical structure development |
| GO:0032269 | 0.003 | 3.059 | 4 | 10 | 161 | negative regulation of cellular protein metabolic process |
| GO:2000113 | 0.003 | 2.216 | 9 | 18 | 400 | negative regulation of cellular macromolecule biosynthetic process |
| GO:0007049 | 0.003 | 1.784 | 21 | 34 | 958 | cell cycle |
| GO:0042982 | 0.003 | 12.316 | 0 | 3 | 14 | amyloid precursor protein metabolic process |
| GO:0030514 | 0.003 | 12.316 | 0 | 3 | 14 | negative regulation of BMP signaling pathway |
| GO:0002685 | 0.003 | 12.316 | 0 | 3 | 14 | regulation of leukocyte migration |
| GO:0031062 | 0.003 | 12.316 | 0 | 3 | 14 | positive regulation of histone methylation |
| GO:0010558 | 0.003 | 2.198 | 9 | 18 | 403 | negative regulation of macromolecule biosynthetic process |
| GO:0048041 | 0.003 | 7.253 | 1 | 4 | 29 | focal adhesion assembly |
| GO:0007019 | 0.003 | 7.253 | 1 | 4 | 29 | microtubule depolymerization |
| GO:0045934 | 0.004 | 2.180 | 9 | 18 | 406 | negative regulation of nucleobase-containing compound metabolic process |
| GO:0051172 | 0.004 | 2.180 | 9 | 18 | 406 | negative regulation of nitrogen compound metabolic process |
| GO:0010638 | 0.004 | 3.861 | 2 | 7 | 90 | positive regulation of organelle organization |
| GO:0016337 | 0.004 | 2.781 | 4 | 11 | 194 | cell-cell adhesion |
| GO:0042221 | 0.004 | 1.683 | 27 | 41 | 1234 | response to chemical stimulus |
| GO:0051253 | 0.004 | 2.220 | 8 | 17 | 376 | negative regulation of RNA metabolic process |
| GO:0032446 | 0.004 | 2.425 | 6 | 14 | 283 | protein modification by small protein conjugation |
| GO:0006915 | 0.004 | 1.943 | 13 | 24 | 611 | apoptotic process |
| GO:0048869 | 0.004 | 1.584 | 40 | 56 | 1831 | cellular developmental process |
| GO:0010639 | 0.004 | 3.769 | 2 | 7 | 92 | negative regulation of organelle organization |
| GO:0010035 | 0.004 | 2.902 | 4 | 10 | 169 | response to inorganic substance |
| GO:0010647 | 0.004 | 2.397 | 6 | 14 | 286 | positive regulation of cell communication |
| GO:0043069 | 0.004 | 2.720 | 4 | 11 | 198 | negative regulation of programmed cell death |
| GO:0045892 | 0.004 | 2.247 | 8 | 16 | 349 | negative regulation of transcription, DNA-dependent |
| GO:0051494 | 0.004 | 5.169 | 1 | 5 | 49 | negative regulation of cytoskeleton organization |
| GO:0030534 | 0.004 | 3.337 | 3 | 8 | 118 | adult behavior |
| GO:0050793 | 0.004 | 1.829 | 17 | 28 | 760 | regulation of developmental process |
| GO:0045595 | 0.004 | 2.083 | 10 | 19 | 448 | regulation of cell differentiation |
| GO:0061061 | 0.004 | 2.302 | 7 | 15 | 319 | muscle structure development |
| GO:0007591 | 0.004 | 6.714 | 1 | 4 | 31 | molting cycle, chitin-based cuticle |
| GO:0006357 | 0.005 | 2.078 | 10 | 19 | 449 | regulation of transcription from RNA polymerase II promoter |
| GO:0043010 | 0.005 | 4.211 | 2 | 6 | 71 | camera-type eye development |
| GO:0048048 | 0.005 | 29.966 | 0 | 2 | 5 | embryonic eye morphogenesis |
| GO:0048137 | 0.005 | 29.966 | 0 | 2 | 5 | spermatocyte division |
| GO:0061316 | 0.005 | 29.966 | 0 | 2 | 5 | canonical Wnt receptor signaling pathway involved in heart development |
| GO:0051574 | 0.005 | 29.966 | 0 | 2 | 5 | positive regulation of histone H3-K9 methylation |
| GO:0003306 | 0.005 | 29.966 | 0 | 2 | 5 | Wnt receptor signaling pathway involved in heart development |
| GO:0006067 | 0.005 | 29.966 | 0 | 2 | 5 | ethanol metabolic process |
| GO:0006996 | 0.005 | 1.613 | 33 | 47 | 1485 | organelle organization |
| GO:0016525 | 0.005 | 10.418 | 0 | 3 | 16 | negative regulation of angiogenesis |
| GO:0044092 | 0.005 | 2.662 | 4 | 11 | 202 | negative regulation of molecular function |
| GO:0007154 | 0.005 | 1.546 | 44 | 60 | 2015 | cell communication |
| GO:0006367 | 0.005 | 4.837 | 1 | 5 | 52 | transcription initiation from RNA polymerase II promoter |
| GO:0048738 | 0.006 | 6.250 | 1 | 4 | 33 | cardiac muscle tissue development |
| GO:0031110 | 0.006 | 6.250 | 1 | 4 | 33 | regulation of microtubule polymerization or depolymerization |
| GO:0022607 | 0.006 | 1.704 | 22 | 34 | 996 | cellular component assembly |
| GO:0055057 | 0.006 | 9.673 | 0 | 3 | 17 | neuroblast division |
| GO:0006366 | 0.006 | 1.957 | 12 | 21 | 527 | transcription from RNA polymerase II promoter |
| GO:0051240 | 0.006 | 2.935 | 3 | 9 | 150 | positive regulation of multicellular organismal process |
| GO:0007517 | 0.006 | 2.480 | 5 | 12 | 236 | muscle organ development |
| GO:0040012 | 0.006 | 2.593 | 5 | 11 | 207 | regulation of locomotion |
| GO:0030335 | 0.006 | 4.735 | 1 | 5 | 53 | positive regulation of cell migration |
| GO:0016567 | 0.006 | 2.365 | 6 | 13 | 268 | protein ubiquitination |
| GO:0009790 | 0.006 | 1.793 | 16 | 27 | 744 | embryo development |
| GO:0051093 | 0.006 | 2.356 | 6 | 13 | 269 | negative regulation of developmental process |
| GO:2000147 | 0.006 | 4.638 | 1 | 5 | 54 | positive regulation of cell motility |
| GO:0043067 | 0.007 | 2.086 | 9 | 17 | 398 | regulation of programmed cell death |
| GO:0060548 | 0.007 | 2.552 | 5 | 11 | 210 | negative regulation of cell death |
| GO:0090077 | 0.007 | 9.027 | 0 | 3 | 18 | foam cell differentiation |
| GO:0010742 | 0.007 | 9.027 | 0 | 3 | 18 | macrophage derived foam cell differentiation |
| GO:0045429 | 0.007 | 22.472 | 0 | 2 | 6 | positive regulation of nitric oxide biosynthetic process |
| GO:0048136 | 0.007 | 22.472 | 0 | 2 | 6 | male germ-line cyst formation |
| GO:0061311 | 0.007 | 22.472 | 0 | 2 | 6 | cell surface receptor signaling pathway involved in heart development |
| GO:0030727 | 0.007 | 22.472 | 0 | 2 | 6 | germarium-derived female germ-line cyst formation |
| GO:0007508 | 0.007 | 22.472 | 0 | 2 | 6 | larval heart development |
| GO:0031076 | 0.007 | 22.472 | 0 | 2 | 6 | embryonic camera-type eye development |
| GO:0034308 | 0.007 | 22.472 | 0 | 2 | 6 | primary alcohol metabolic process |
| GO:0016340 | 0.007 | 22.472 | 0 | 2 | 6 | calcium-dependent cell-matrix adhesion |
| GO:0050900 | 0.007 | 5.845 | 1 | 4 | 35 | leukocyte migration |
| GO:0048641 | 0.007 | 5.845 | 1 | 4 | 35 | regulation of skeletal muscle tissue development |
| GO:0043242 | 0.007 | 5.845 | 1 | 4 | 35 | negative regulation of protein complex disassembly |
| GO:0007165 | 0.007 | 1.548 | 37 | 51 | 1678 | signal transduction |
| GO:0045893 | 0.007 | 2.021 | 10 | 18 | 435 | positive regulation of transcription, DNA-dependent |
| GO:0051129 | 0.007 | 2.813 | 3 | 9 | 156 | negative regulation of cellular component organization |
| GO:0070647 | 0.007 | 2.161 | 7 | 15 | 338 | protein modification by small protein conjugation or removal |
| GO:0031328 | 0.008 | 1.904 | 12 | 21 | 540 | positive regulation of cellular biosynthetic process |
| GO:0045471 | 0.008 | 4.455 | 1 | 5 | 56 | response to ethanol |
| GO:0014706 | 0.008 | 2.631 | 4 | 10 | 185 | striated muscle tissue development |
| GO:0035335 | 0.008 | 5.662 | 1 | 4 | 36 | peptidyl-tyrosine dephosphorylation |
| GO:0042337 | 0.008 | 8.462 | 0 | 3 | 19 | cuticle development involved in chitin-based cuticle molting cycle |
| GO:0044238 | 0.008 | 1.513 | 117 | 133 | 5339 | primary metabolic process |
| GO:0043254 | 0.008 | 3.298 | 2 | 7 | 104 | regulation of protein complex assembly |
| GO:0097305 | 0.008 | 4.369 | 1 | 5 | 57 | response to alcohol |
| GO:0006468 | 0.008 | 2.083 | 8 | 16 | 374 | protein phosphorylation |
| GO:0009792 | 0.008 | 2.025 | 9 | 17 | 409 | embryo development ending in birth or egg hatching |
| GO:0097006 | 0.008 | 5.489 | 1 | 4 | 37 | regulation of plasma lipoprotein particle levels |
| GO:0031109 | 0.008 | 5.489 | 1 | 4 | 37 | microtubule polymerization or depolymerization |
| GO:0044093 | 0.009 | 2.019 | 9 | 17 | 410 | positive regulation of molecular function |
| GO:0051272 | 0.009 | 4.286 | 1 | 5 | 58 | positive regulation of cellular component movement |
| GO:0044087 | 0.009 | 2.439 | 5 | 11 | 219 | regulation of cellular component biogenesis |
| GO:0071842 | 0.009 | 1.480 | 49 | 64 | 2236 | cellular component organization at cellular level |
| GO:0032501 | 0.009 | 1.446 | 72 | 88 | 3272 | multicellular organismal process |
| GO:0040007 | 0.009 | 1.900 | 11 | 20 | 514 | growth |
| GO:0048149 | 0.009 | 7.963 | 0 | 3 | 20 | behavioral response to ethanol |
| GO:0031060 | 0.009 | 7.963 | 0 | 3 | 20 | regulation of histone methylation |
| GO:0031056 | 0.009 | 5.327 | 1 | 4 | 38 | regulation of histone modification |
| GO:0042335 | 0.009 | 4.206 | 1 | 5 | 59 | cuticle development |
| GO:0048588 | 0.009 | 4.206 | 1 | 5 | 59 | developmental cell growth |
| GO:0060537 | 0.009 | 2.542 | 4 | 10 | 191 | muscle tissue development |
| GO:0045428 | 0.009 | 17.975 | 0 | 2 | 7 | regulation of nitric oxide biosynthetic process |
| GO:0048135 | 0.009 | 17.975 | 0 | 2 | 7 | female germ-line cyst formation |
| GO:0045909 | 0.009 | 17.975 | 0 | 2 | 7 | positive regulation of vasodilation |
| GO:0051570 | 0.009 | 17.975 | 0 | 2 | 7 | regulation of histone H3-K9 methylation |
| GO:0010560 | 0.009 | 17.975 | 0 | 2 | 7 | positive regulation of glycoprotein biosynthetic process |
| GO:0072676 | 0.009 | 17.975 | 0 | 2 | 7 | lymphocyte migration |
| GO:0060052 | 0.009 | 17.975 | 0 | 2 | 7 | neurofilament cytoskeleton organization |
| GO:0060026 | 0.009 | 17.975 | 0 | 2 | 7 | convergent extension |
| GO:2000401 | 0.009 | 17.975 | 0 | 2 | 7 | regulation of lymphocyte migration |
| GO:2000826 | 0.009 | 17.975 | 0 | 2 | 7 | regulation of heart morphogenesis |
| GO:0030182 | 0.010 | 1.745 | 16 | 26 | 732 | neuron differentiation |
| GO:0019220 | 0.010 | 2.094 | 8 | 15 | 348 | regulation of phosphate metabolic process |
| GO:0048812 | 0.010 | 1.917 | 11 | 19 | 483 | neuron projection morphogenesis |
| GO:0007275 | 0.010 | 1.456 | 57 | 72 | 2584 | multicellular organismal development |
| GO:0010941 | 0.010 | 1.993 | 9 | 17 | 415 | regulation of cell death |
| GO:0002376 | 0.010 | 1.826 | 13 | 22 | 589 | immune system process |
| GO:0042127 | 0.010 | 2.036 | 8 | 16 | 382 | regulation of cell proliferation |
| GO:0042981 | 0.010 | 2.036 | 8 | 16 | 382 | regulation of apoptotic process |
| GO:0000902 | 0.010 | 1.759 | 15 | 25 | 697 | cell morphogenesis |
| GO:0051174 | 0.010 | 2.087 | 8 | 15 | 349 | regulation of phosphorus metabolic process |
| GO:0006351 | 0.010 | 1.549 | 31 | 44 | 1426 | transcription, DNA-dependent |
| GO:0051348 | 0.010 | 4.129 | 1 | 5 | 60 | negative regulation of transferase activity |
| GO:0043066 | 0.010 | 2.514 | 4 | 10 | 193 | negative regulation of apoptotic process |
| GO:0051153 | 0.010 | 5.174 | 1 | 4 | 39 | regulation of striated muscle cell differentiation |
| GO:0042336 | 0.010 | 7.520 | 0 | 3 | 21 | cuticle development involved in protein-based cuticle molting cycle |
| GO:0035195 | 0.010 | 7.520 | 0 | 3 | 21 | gene silencing by miRNA |
| GO:0001936 | 0.010 | 7.520 | 0 | 3 | 21 | regulation of endothelial cell proliferation |
| GO:0043255 | 0.010 | 7.520 | 0 | 3 | 21 | regulation of carbohydrate biosynthetic process |
| GO:0032774 | 0.010 | 1.544 | 31 | 44 | 1430 | RNA biosynthetic process |
| GO:0000904 | 0.011 | 1.868 | 11 | 20 | 522 | cell morphogenesis involved in differentiation |
| GO:0001816 | 0.011 | 3.459 | 2 | 6 | 85 | cytokine production |
| GO:0001934 | 0.011 | 2.630 | 4 | 9 | 166 | positive regulation of protein phosphorylation |
| GO:0009605 | 0.011 | 1.723 | 16 | 26 | 740 | response to external stimulus |
| GO:0040003 | 0.011 | 5.030 | 1 | 4 | 40 | chitin-based cuticle development |
| GO:0051963 | 0.011 | 5.030 | 1 | 4 | 40 | regulation of synapse assembly |
| GO:0031398 | 0.011 | 5.030 | 1 | 4 | 40 | positive regulation of protein ubiquitination |
| GO:0015918 | 0.011 | 3.983 | 1 | 5 | 62 | sterol transport |
| GO:0031058 | 0.012 | 7.123 | 0 | 3 | 22 | positive regulation of histone modification |
| GO:0009888 | 0.012 | 1.694 | 17 | 27 | 782 | tissue development |
| GO:0033273 | 0.012 | 4.894 | 1 | 4 | 41 | response to vitamin |
| GO:0035051 | 0.012 | 3.914 | 1 | 5 | 63 | cardiac cell differentiation |
| GO:0032925 | 0.012 | 14.978 | 0 | 2 | 8 | regulation of activin receptor signaling pathway |
| GO:0002479 | 0.012 | 14.978 | 0 | 2 | 8 | antigen processing and presentation of exogenous peptide antigen via MHC class I, TAP-dependent |
| GO:0046850 | 0.012 | 14.978 | 0 | 2 | 8 | regulation of bone remodeling |
| GO:0003401 | 0.012 | 14.978 | 0 | 2 | 8 | axis elongation |
| GO:0034103 | 0.012 | 14.978 | 0 | 2 | 8 | regulation of tissue remodeling |
| GO:0055024 | 0.012 | 14.978 | 0 | 2 | 8 | regulation of cardiac muscle tissue development |
| GO:0048584 | 0.013 | 2.024 | 8 | 15 | 359 | positive regulation of response to stimulus |
| GO:0044085 | 0.013 | 1.570 | 26 | 37 | 1169 | cellular component biogenesis |
| GO:0051254 | 0.013 | 1.891 | 10 | 18 | 462 | positive regulation of RNA metabolic process |
| GO:0042303 | 0.013 | 3.847 | 1 | 5 | 64 | molting cycle |
| GO:0031396 | 0.013 | 3.847 | 1 | 5 | 64 | regulation of protein ubiquitination |
| GO:0051336 | 0.013 | 2.131 | 6 | 13 | 295 | regulation of hydrolase activity |
| GO:0016043 | 0.013 | 1.431 | 57 | 71 | 2573 | cellular component organization |
| GO:0016571 | 0.013 | 4.764 | 1 | 4 | 42 | histone methylation |
| GO:0035194 | 0.013 | 4.764 | 1 | 4 | 42 | posttranscriptional gene silencing by RNA |
| GO:0010565 | 0.013 | 4.764 | 1 | 4 | 42 | regulation of cellular ketone metabolic process |
| GO:0016441 | 0.013 | 4.764 | 1 | 4 | 42 | posttranscriptional gene silencing |
| GO:0007494 | 0.013 | 6.766 | 1 | 3 | 23 | midgut development |
| GO:0000245 | 0.013 | 6.766 | 1 | 3 | 23 | spliceosomal complex assembly |
| GO:0009967 | 0.013 | 2.197 | 6 | 12 | 264 | positive regulation of signal transduction |
| GO:0010628 | 0.014 | 1.874 | 10 | 18 | 466 | positive regulation of gene expression |
| GO:0043933 | 0.014 | 1.783 | 13 | 21 | 573 | macromolecular complex subunit organization |
| GO:0017015 | 0.014 | 4.641 | 1 | 4 | 43 | regulation of transforming growth factor beta receptor signaling pathway |
| GO:0017145 | 0.014 | 4.641 | 1 | 4 | 43 | stem cell division |
| GO:0007507 | 0.014 | 2.260 | 5 | 11 | 235 | heart development |
| GO:0007399 | 0.015 | 1.544 | 27 | 38 | 1220 | nervous system development |
| GO:0006725 | 0.015 | 2.485 | 4 | 9 | 175 | cellular aromatic compound metabolic process |
| GO:0045935 | 0.015 | 1.825 | 11 | 19 | 505 | positive regulation of nucleobase-containing compound metabolic process |
| GO:0080134 | 0.015 | 2.162 | 6 | 12 | 268 | regulation of response to stress |
| GO:2001252 | 0.015 | 6.443 | 1 | 3 | 24 | positive regulation of chromosome organization |
| GO:0007592 | 0.015 | 6.443 | 1 | 3 | 24 | protein-based cuticle development |
| GO:0006006 | 0.015 | 2.877 | 3 | 7 | 118 | glucose metabolic process |
| GO:0002682 | 0.015 | 2.240 | 5 | 11 | 237 | regulation of immune system process |
| GO:0007010 | 0.016 | 1.720 | 14 | 23 | 651 | cytoskeleton organization |
| GO:0060260 | 0.016 | 12.836 | 0 | 2 | 9 | regulation of transcription initiation from RNA polymerase II promoter |
| GO:0040016 | 0.016 | 12.836 | 0 | 2 | 9 | embryonic cleavage |
| GO:0006521 | 0.016 | 12.836 | 0 | 2 | 9 | regulation of cellular amino acid metabolic process |
| GO:0001937 | 0.016 | 12.836 | 0 | 2 | 9 | negative regulation of endothelial cell proliferation |
| GO:0007392 | 0.016 | 12.836 | 0 | 2 | 9 | initiation of dorsal closure |
| GO:0046692 | 0.016 | 12.836 | 0 | 2 | 9 | sperm competition |
| GO:0046693 | 0.016 | 12.836 | 0 | 2 | 9 | sperm storage |
| GO:0031054 | 0.016 | 12.836 | 0 | 2 | 9 | pre-miRNA processing |
| GO:0010822 | 0.016 | 12.836 | 0 | 2 | 9 | positive regulation of mitochondrion organization |
| GO:0010596 | 0.016 | 12.836 | 0 | 2 | 9 | negative regulation of endothelial cell migration |
| GO:0008364 | 0.016 | 12.836 | 0 | 2 | 9 | pupal chitin-based cuticle development |
| GO:0031128 | 0.016 | 12.836 | 0 | 2 | 9 | developmental induction |
| GO:0042327 | 0.016 | 2.455 | 4 | 9 | 177 | positive regulation of phosphorylation |
| GO:0071844 | 0.016 | 1.629 | 18 | 28 | 841 | cellular component assembly at cellular level |
| GO:0051173 | 0.017 | 1.805 | 11 | 19 | 510 | positive regulation of nitrogen compound metabolic process |
| GO:0010921 | 0.017 | 4.414 | 1 | 4 | 45 | regulation of phosphatase activity |
| GO:0070887 | 0.017 | 1.801 | 11 | 19 | 511 | cellular response to chemical stimulus |
| GO:0048103 | 0.017 | 6.149 | 1 | 3 | 25 | somatic stem cell division |
| GO:0007157 | 0.017 | 6.149 | 1 | 3 | 25 | heterophilic cell-cell adhesion |
| GO:0048858 | 0.017 | 1.773 | 12 | 20 | 547 | cell projection morphogenesis |
| GO:0048667 | 0.017 | 1.830 | 10 | 18 | 476 | cell morphogenesis involved in neuron differentiation |
| GO:0006338 | 0.018 | 3.544 | 2 | 5 | 69 | chromatin remodeling |
| GO:0034623 | 0.018 | 3.544 | 2 | 5 | 69 | cellular macromolecular complex disassembly |
| GO:0001932 | 0.018 | 2.110 | 6 | 12 | 274 | regulation of protein phosphorylation |
| GO:0008306 | 0.018 | 4.308 | 1 | 4 | 46 | associative learning |
| GO:0031334 | 0.018 | 4.308 | 1 | 4 | 46 | positive regulation of protein complex assembly |
| GO:0016310 | 0.018 | 1.850 | 10 | 17 | 444 | phosphorylation |
| GO:0001817 | 0.019 | 3.490 | 2 | 5 | 70 | regulation of cytokine production |
| GO:0007420 | 0.019 | 2.171 | 5 | 11 | 244 | brain development |
| GO:0030516 | 0.019 | 5.881 | 1 | 3 | 26 | regulation of axon extension |
| GO:0019318 | 0.019 | 2.539 | 3 | 8 | 152 | hexose metabolic process |
| GO:0007417 | 0.019 | 1.913 | 8 | 15 | 378 | central nervous system development |
| GO:0042312 | 0.019 | 11.230 | 0 | 2 | 10 | regulation of vasodilation |
| GO:0007320 | 0.019 | 11.230 | 0 | 2 | 10 | insemination |
| GO:2000142 | 0.019 | 11.230 | 0 | 2 | 10 | regulation of DNA-dependent transcription, initiation |
| GO:0050678 | 0.019 | 4.208 | 1 | 4 | 47 | regulation of epithelial cell proliferation |
| GO:0043406 | 0.019 | 4.208 | 1 | 4 | 47 | positive regulation of MAP kinase activity |
| GO:0043244 | 0.019 | 4.208 | 1 | 4 | 47 | regulation of protein complex disassembly |
| GO:0032990 | 0.019 | 1.745 | 12 | 20 | 555 | cell part morphogenesis |
| GO:0009653 | 0.020 | 1.474 | 33 | 44 | 1484 | anatomical structure morphogenesis |
| GO:0032984 | 0.020 | 3.436 | 2 | 5 | 71 | macromolecular complex disassembly |
| GO:0045944 | 0.020 | 2.152 | 5 | 11 | 246 | positive regulation of transcription from RNA polymerase II promoter |
| GO:0048699 | 0.020 | 1.628 | 17 | 26 | 778 | generation of neurons |
| GO:0051239 | 0.020 | 1.598 | 19 | 28 | 855 | regulation of multicellular organismal process |
| GO:0018193 | 0.021 | 2.062 | 6 | 12 | 280 | peptidyl-amino acid modification |
| GO:0051726 | 0.021 | 1.897 | 8 | 15 | 381 | regulation of cell cycle |
| GO:0001525 | 0.021 | 2.965 | 2 | 6 | 98 | angiogenesis |
| GO:0051338 | 0.021 | 2.226 | 5 | 10 | 216 | regulation of transferase activity |
| GO:0031331 | 0.021 | 4.111 | 1 | 4 | 48 | positive regulation of cellular catabolic process |
| GO:0042558 | 0.021 | 5.636 | 1 | 3 | 27 | pteridine-containing compound metabolic process |
| GO:0030510 | 0.021 | 5.636 | 1 | 3 | 27 | regulation of BMP signaling pathway |
| GO:0000209 | 0.021 | 3.384 | 2 | 5 | 72 | protein polyubiquitination |
| GO:0048666 | 0.021 | 1.687 | 14 | 22 | 632 | neuron development |
| GO:0048598 | 0.021 | 1.992 | 7 | 13 | 314 | embryonic morphogenesis |
| GO:0000226 | 0.021 | 2.054 | 6 | 12 | 281 | microtubule cytoskeleton organization |
| GO:0022603 | 0.021 | 1.985 | 7 | 13 | 315 | regulation of anatomical structure morphogenesis |
| GO:0051321 | 0.022 | 2.469 | 3 | 8 | 156 | meiotic cell cycle |
| GO:0016311 | 0.022 | 2.469 | 3 | 8 | 156 | dephosphorylation |
| GO:0007618 | 0.022 | 2.933 | 2 | 6 | 99 | mating |
| GO:0001700 | 0.022 | 2.658 | 3 | 7 | 127 | embryonic development via the syncytial blastoderm |
| GO:0050673 | 0.022 | 3.334 | 2 | 5 | 73 | epithelial cell proliferation |
| GO:2000682 | 0.022 | Inf | 0 | 1 | 1 | positive regulation of rubidium ion transport |
| GO:2000686 | 0.022 | Inf | 0 | 1 | 1 | regulation of rubidium ion transmembrane transporter activity |
| GO:2000688 | 0.022 | Inf | 0 | 1 | 1 | positive regulation of rubidium ion transmembrane transporter activity |
| GO:0060267 | 0.022 | Inf | 0 | 1 | 1 | positive regulation of respiratory burst |
| GO:0060235 | 0.022 | Inf | 0 | 1 | 1 | lens induction in camera-type eye |
| GO:0021861 | 0.022 | Inf | 0 | 1 | 1 | forebrain radial glial cell differentiation |
| GO:0021874 | 0.022 | Inf | 0 | 1 | 1 | Wnt receptor signaling pathway involved in forebrain neuroblast division |
| GO:0019087 | 0.022 | Inf | 0 | 1 | 1 | transformation of host cell by virus |
| GO:0021535 | 0.022 | Inf | 0 | 1 | 1 | cell migration in hindbrain |
| GO:0006451 | 0.022 | Inf | 0 | 1 | 1 | translational readthrough |
| GO:0060592 | 0.022 | Inf | 0 | 1 | 1 | mammary gland formation |
| GO:0060596 | 0.022 | Inf | 0 | 1 | 1 | mammary placode formation |
| GO:2000983 | 0.022 | Inf | 0 | 1 | 1 | regulation of ATP citrate synthase activity |
| GO:2000984 | 0.022 | Inf | 0 | 1 | 1 | negative regulation of ATP citrate synthase activity |
| GO:0060558 | 0.022 | Inf | 0 | 1 | 1 | regulation of calcidiol 1-monooxygenase activity |
| GO:0006407 | 0.022 | Inf | 0 | 1 | 1 | rRNA export from nucleus |
| GO:0060534 | 0.022 | Inf | 0 | 1 | 1 | trachea cartilage development |
| GO:0060535 | 0.022 | Inf | 0 | 1 | 1 | trachea cartilage morphogenesis |
| GO:0042795 | 0.022 | Inf | 0 | 1 | 1 | snRNA transcription from RNA polymerase II promoter |
| GO:0042796 | 0.022 | Inf | 0 | 1 | 1 | snRNA transcription from RNA polymerase III promoter |
| GO:0019343 | 0.022 | Inf | 0 | 1 | 1 | cysteine biosynthetic process via cystathionine |
| GO:0042758 | 0.022 | Inf | 0 | 1 | 1 | long-chain fatty acid catabolic process |
| GO:0034983 | 0.022 | Inf | 0 | 1 | 1 | peptidyl-lysine deacetylation |
| GO:0045083 | 0.022 | Inf | 0 | 1 | 1 | negative regulation of interleukin-12 biosynthetic process |
| GO:0021943 | 0.022 | Inf | 0 | 1 | 1 | formation of radial glial scaffolds |
| GO:0021932 | 0.022 | Inf | 0 | 1 | 1 | hindbrain radial glia guided cell migration |
| GO:0050882 | 0.022 | Inf | 0 | 1 | 1 | voluntary musculoskeletal movement |
| GO:0006535 | 0.022 | Inf | 0 | 1 | 1 | cysteine biosynthetic process from serine |
| GO:1900037 | 0.022 | Inf | 0 | 1 | 1 | regulation of cellular response to hypoxia |
| GO:0045341 | 0.022 | Inf | 0 | 1 | 1 | MHC class I biosynthetic process |
| GO:0045343 | 0.022 | Inf | 0 | 1 | 1 | regulation of MHC class I biosynthetic process |
| GO:0045345 | 0.022 | Inf | 0 | 1 | 1 | positive regulation of MHC class I biosynthetic process |
| GO:0045162 | 0.022 | Inf | 0 | 1 | 1 | clustering of voltage-gated sodium channels |
| GO:0071397 | 0.022 | Inf | 0 | 1 | 1 | cellular response to cholesterol |
| GO:0014719 | 0.022 | Inf | 0 | 1 | 1 | satellite cell activation |
| GO:0042985 | 0.022 | Inf | 0 | 1 | 1 | negative regulation of amyloid precursor protein biosynthetic process |
| GO:0014816 | 0.022 | Inf | 0 | 1 | 1 | satellite cell differentiation |
| GO:0014841 | 0.022 | Inf | 0 | 1 | 1 | satellite cell proliferation |
| GO:0001514 | 0.022 | Inf | 0 | 1 | 1 | selenocysteine incorporation |
| GO:0030034 | 0.022 | Inf | 0 | 1 | 1 | microvillar actin bundle assembly |
| GO:0032514 | 0.022 | Inf | 0 | 1 | 1 | positive regulation of protein phosphatase type 2B activity |
| GO:1900125 | 0.022 | Inf | 0 | 1 | 1 | regulation of hyaluronan biosynthetic process |
| GO:1900127 | 0.022 | Inf | 0 | 1 | 1 | positive regulation of hyaluronan biosynthetic process |
| GO:1900112 | 0.022 | Inf | 0 | 1 | 1 | regulation of histone H3-K9 trimethylation |
| GO:1900114 | 0.022 | Inf | 0 | 1 | 1 | positive regulation of histone H3-K9 trimethylation |
| GO:0061072 | 0.022 | Inf | 0 | 1 | 1 | iris morphogenesis |
| GO:0071244 | 0.022 | Inf | 0 | 1 | 1 | cellular response to carbon dioxide |
| GO:0061350 | 0.022 | Inf | 0 | 1 | 1 | planar cell polarity pathway involved in cardiac muscle tissue morphogenesis |
| GO:0061354 | 0.022 | Inf | 0 | 1 | 1 | planar cell polarity pathway involved in pericardium morphogenesis |
| GO:0071593 | 0.022 | Inf | 0 | 1 | 1 | lymphocyte aggregation |
| GO:0061324 | 0.022 | Inf | 0 | 1 | 1 | canonical Wnt receptor signaling pathway involved in positive regulation of cardiac outflow tract cell proliferation |
| GO:0061325 | 0.022 | Inf | 0 | 1 | 1 | cell proliferation involved in outflow tract morphogenesis |
| GO:0061310 | 0.022 | Inf | 0 | 1 | 1 | canonical Wnt receptor signaling pathway involved in cardiac neural crest cell differentiation involved in heart development |
| GO:0061341 | 0.022 | Inf | 0 | 1 | 1 | non-canonical Wnt receptor signaling pathway involved in heart development |
| GO:0061346 | 0.022 | Inf | 0 | 1 | 1 | planar cell polarity pathway involved in heart morphogenesis |
| GO:0061347 | 0.022 | Inf | 0 | 1 | 1 | planar cell polarity pathway involved in outflow tract morphogenesis |
| GO:0061348 | 0.022 | Inf | 0 | 1 | 1 | planar cell polarity pathway involved in ventricular septum morphogenesis |
| GO:0061349 | 0.022 | Inf | 0 | 1 | 1 | planar cell polarity pathway involved in cardiac right atrium morphogenesis |
| GO:0061307 | 0.022 | Inf | 0 | 1 | 1 | cardiac neural crest cell differentiation involved in heart development |
| GO:0071603 | 0.022 | Inf | 0 | 1 | 1 | endothelial cell-cell adhesion |
| GO:0035733 | 0.022 | Inf | 0 | 1 | 1 | hepatic stellate cell activation |
| GO:0030213 | 0.022 | Inf | 0 | 1 | 1 | hyaluronan biosynthetic process |
| GO:0002051 | 0.022 | Inf | 0 | 1 | 1 | osteoblast fate commitment |
| GO:0007182 | 0.022 | Inf | 0 | 1 | 1 | common-partner SMAD protein phosphorylation |
| GO:0030578 | 0.022 | Inf | 0 | 1 | 1 | PML body organization |
| GO:0051389 | 0.022 | Inf | 0 | 1 | 1 | inactivation of MAPKK activity |
| GO:0090009 | 0.022 | Inf | 0 | 1 | 1 | primitive streak formation |
| GO:0010037 | 0.022 | Inf | 0 | 1 | 1 | response to carbon dioxide |
| GO:0051132 | 0.022 | Inf | 0 | 1 | 1 | NK T cell activation |
| GO:0051133 | 0.022 | Inf | 0 | 1 | 1 | regulation of NK T cell activation |
| GO:0051135 | 0.022 | Inf | 0 | 1 | 1 | positive regulation of NK T cell activation |
| GO:0030997 | 0.022 | Inf | 0 | 1 | 1 | regulation of centriole-centriole cohesion |
| GO:0090245 | 0.022 | Inf | 0 | 1 | 1 | axis elongation involved in somitogenesis |
| GO:0018119 | 0.022 | Inf | 0 | 1 | 1 | peptidyl-cysteine S-nitrosylation |
| GO:0046600 | 0.022 | Inf | 0 | 1 | 1 | negative regulation of centriole replication |
| GO:0046606 | 0.022 | Inf | 0 | 1 | 1 | negative regulation of centrosome cycle |
| GO:0051974 | 0.022 | Inf | 0 | 1 | 1 | negative regulation of telomerase activity |
| GO:0036109 | 0.022 | Inf | 0 | 1 | 1 | alpha-linolenic acid metabolic process |
| GO:0072537 | 0.022 | Inf | 0 | 1 | 1 | fibroblast activation |
| GO:1901219 | 0.022 | Inf | 0 | 1 | 1 | regulation of cardiac chamber morphogenesis |
| GO:0000117 | 0.022 | Inf | 0 | 1 | 1 | regulation of transcription involved in G2/M-phase of mitotic cell cycle |
| GO:0044340 | 0.022 | Inf | 0 | 1 | 1 | canonical Wnt receptor signaling pathway involved in regulation of cell proliferation |
| GO:0044332 | 0.022 | Inf | 0 | 1 | 1 | Wnt receptor signaling pathway involved in dorsal/ventral axis specification |
| GO:2000051 | 0.022 | Inf | 0 | 1 | 1 | negative regulation of non-canonical Wnt receptor signaling pathway |
| GO:2000053 | 0.022 | Inf | 0 | 1 | 1 | regulation of Wnt receptor signaling pathway involved in dorsal/ventral axis specification |
| GO:2000055 | 0.022 | Inf | 0 | 1 | 1 | positive regulation of Wnt receptor signaling pathway involved in dorsal/ventral axis specification |
| GO:0010610 | 0.022 | Inf | 0 | 1 | 1 | regulation of mRNA stability involved in response to stress |
| GO:0015919 | 0.022 | Inf | 0 | 1 | 1 | peroxisomal membrane transport |
| GO:0070486 | 0.022 | Inf | 0 | 1 | 1 | leukocyte aggregation |
| GO:0010956 | 0.022 | Inf | 0 | 1 | 1 | negative regulation of calcidiol 1-monooxygenase activity |
| GO:0010915 | 0.022 | Inf | 0 | 1 | 1 | regulation of very-low-density lipoprotein particle clearance |
| GO:0010916 | 0.022 | Inf | 0 | 1 | 1 | negative regulation of very-low-density lipoprotein particle clearance |
| GO:0031293 | 0.022 | Inf | 0 | 1 | 1 | membrane protein intracellular domain proteolysis |
| GO:2000491 | 0.022 | Inf | 0 | 1 | 1 | positive regulation of hepatic stellate cell activation |
| GO:2000489 | 0.022 | Inf | 0 | 1 | 1 | regulation of hepatic stellate cell activation |
| GO:0060028 | 0.022 | Inf | 0 | 1 | 1 | convergent extension involved in axis elongation |
| GO:0060019 | 0.022 | Inf | 0 | 1 | 1 | radial glial cell differentiation |
| GO:2000160 | 0.022 | Inf | 0 | 1 | 1 | negative regulation of planar cell polarity pathway involved in heart morphogenesis |
| GO:2000161 | 0.022 | Inf | 0 | 1 | 1 | regulation of planar cell polarity pathway involved in cardiac right atrium morphogenesis |
| GO:2000162 | 0.022 | Inf | 0 | 1 | 1 | negative regulation of planar cell polarity pathway involved in cardiac right atrium morphogenesis |
| GO:2000163 | 0.022 | Inf | 0 | 1 | 1 | regulation of planar cell polarity pathway involved in outflow tract morphogenesis |
| GO:2000164 | 0.022 | Inf | 0 | 1 | 1 | negative regulation of planar cell polarity pathway involved in outflow tract morphogenesis |
| GO:2000165 | 0.022 | Inf | 0 | 1 | 1 | regulation of planar cell polarity pathway involved in pericardium morphogenesis |
| GO:2000166 | 0.022 | Inf | 0 | 1 | 1 | negative regulation of planar cell polarity pathway involved in pericardium morphogenesis |
| GO:2000167 | 0.022 | Inf | 0 | 1 | 1 | regulation of planar cell polarity pathway involved in neural tube closure |
| GO:2000168 | 0.022 | Inf | 0 | 1 | 1 | negative regulation of planar cell polarity pathway involved in neural tube closure |
| GO:2000169 | 0.022 | Inf | 0 | 1 | 1 | regulation of peptidyl-cysteine S-nitrosylation |
| GO:2000150 | 0.022 | Inf | 0 | 1 | 1 | regulation of planar cell polarity pathway involved in cardiac muscle tissue morphogenesis |
| GO:2000151 | 0.022 | Inf | 0 | 1 | 1 | negative regulation of planar cell polarity pathway involved in cardiac muscle tissue morphogenesis |
| GO:2000159 | 0.022 | Inf | 0 | 1 | 1 | regulation of planar cell polarity pathway involved in heart morphogenesis |
| GO:0003307 | 0.022 | Inf | 0 | 1 | 1 | regulation of Wnt receptor signaling pathway involved in heart development |
| GO:0003308 | 0.022 | Inf | 0 | 1 | 1 | negative regulation of Wnt receptor signaling pathway involved in heart development |
| GO:2000148 | 0.022 | Inf | 0 | 1 | 1 | regulation of planar cell polarity pathway involved in ventricular septum morphogenesis |
| GO:2000149 | 0.022 | Inf | 0 | 1 | 1 | negative regulation of planar cell polarity pathway involved in ventricular septum morphogenesis |
| GO:0070846 | 0.022 | Inf | 0 | 1 | 1 | Hsp90 deacetylation |
| GO:0003213 | 0.022 | Inf | 0 | 1 | 1 | cardiac right atrium morphogenesis |
| GO:0006069 | 0.022 | Inf | 0 | 1 | 1 | ethanol oxidation |
| GO:0048675 | 0.022 | 4.020 | 1 | 4 | 49 | axon extension |
| GO:0006030 | 0.022 | 4.020 | 1 | 4 | 49 | chitin metabolic process |
| GO:0071841 | 0.022 | 1.393 | 52 | 65 | 2374 | cellular component organization or biogenesis at cellular level |
| GO:0061387 | 0.023 | 5.409 | 1 | 3 | 28 | regulation of extent of cell growth |
| GO:0035239 | 0.023 | 2.435 | 3 | 8 | 158 | tube morphogenesis |
| GO:0035099 | 0.023 | 9.981 | 0 | 2 | 11 | hemocyte migration |
| GO:0009826 | 0.023 | 9.981 | 0 | 2 | 11 | unidimensional cell growth |
| GO:0032924 | 0.023 | 9.981 | 0 | 2 | 11 | activin receptor signaling pathway |
| GO:0007614 | 0.023 | 9.981 | 0 | 2 | 11 | short-term memory |
| GO:0000266 | 0.023 | 9.981 | 0 | 2 | 11 | mitochondrial fission |
| GO:0016075 | 0.023 | 9.981 | 0 | 2 | 11 | rRNA catabolic process |
| GO:0034433 | 0.023 | 9.981 | 0 | 2 | 11 | steroid esterification |
| GO:0034434 | 0.023 | 9.981 | 0 | 2 | 11 | sterol esterification |
| GO:0034435 | 0.023 | 9.981 | 0 | 2 | 11 | cholesterol esterification |
| GO:0006979 | 0.023 | 2.614 | 3 | 7 | 129 | response to oxidative stress |
| GO:0010627 | 0.023 | 2.288 | 4 | 9 | 189 | regulation of intracellular protein kinase cascade |
| GO:0031175 | 0.024 | 1.730 | 12 | 19 | 530 | neuron projection development |
| GO:0016477 | 0.024 | 1.860 | 9 | 15 | 388 | cell migration |
| GO:0030278 | 0.024 | 3.932 | 1 | 4 | 50 | regulation of ossification |
| GO:0008152 | 0.024 | 1.439 | 129 | 141 | 5857 | metabolic process |
| GO:0007155 | 0.024 | 1.753 | 11 | 18 | 495 | cell adhesion |
| GO:0032989 | 0.024 | 1.595 | 17 | 26 | 792 | cellular component morphogenesis |
| GO:0006325 | 0.025 | 1.938 | 7 | 13 | 322 | chromatin organization |
| GO:0001935 | 0.025 | 5.201 | 1 | 3 | 29 | endothelial cell proliferation |
| GO:0043393 | 0.025 | 5.201 | 1 | 3 | 29 | regulation of protein binding |
| GO:0008355 | 0.025 | 5.201 | 1 | 3 | 29 | olfactory learning |
| GO:0008582 | 0.025 | 5.201 | 1 | 3 | 29 | regulation of synaptic growth at neuromuscular junction |
| GO:0006479 | 0.025 | 3.848 | 1 | 4 | 51 | protein methylation |
| GO:0022404 | 0.025 | 3.848 | 1 | 4 | 51 | molting cycle process |
| GO:0051261 | 0.025 | 3.848 | 1 | 4 | 51 | protein depolymerization |
| GO:0018108 | 0.025 | 3.848 | 1 | 4 | 51 | peptidyl-tyrosine phosphorylation |
| GO:0008213 | 0.025 | 3.848 | 1 | 4 | 51 | protein alkylation |
| GO:0006352 | 0.026 | 3.192 | 2 | 5 | 76 | DNA-dependent transcription, initiation |
| GO:0007391 | 0.026 | 3.192 | 2 | 5 | 76 | dorsal closure |
| GO:0045937 | 0.026 | 2.249 | 4 | 9 | 192 | positive regulation of phosphate metabolic process |
| GO:0010562 | 0.026 | 2.249 | 4 | 9 | 192 | positive regulation of phosphorus metabolic process |
| GO:0016568 | 0.026 | 1.985 | 6 | 12 | 290 | chromatin modification |
| GO:0044237 | 0.027 | 1.379 | 112 | 125 | 5100 | cellular metabolic process |
| GO:0048731 | 0.027 | 1.395 | 44 | 56 | 2013 | system development |
| GO:0018212 | 0.027 | 3.767 | 1 | 4 | 52 | peptidyl-tyrosine modification |
| GO:0016925 | 0.027 | 8.982 | 0 | 2 | 12 | protein sumoylation |
| GO:0032768 | 0.027 | 8.982 | 0 | 2 | 12 | regulation of monooxygenase activity |
| GO:0045103 | 0.027 | 8.982 | 0 | 2 | 12 | intermediate filament-based process |
| GO:0045104 | 0.027 | 8.982 | 0 | 2 | 12 | intermediate filament cytoskeleton organization |
| GO:0032885 | 0.027 | 8.982 | 0 | 2 | 12 | regulation of polysaccharide biosynthetic process |
| GO:0035196 | 0.027 | 8.982 | 0 | 2 | 12 | production of miRNAs involved in gene silencing by miRNA |
| GO:0035209 | 0.027 | 8.982 | 0 | 2 | 12 | pupal development |
| GO:0002040 | 0.027 | 8.982 | 0 | 2 | 12 | sprouting angiogenesis |
| GO:0030511 | 0.027 | 8.982 | 0 | 2 | 12 | positive regulation of transforming growth factor beta receptor signaling pathway |
| GO:0051567 | 0.027 | 8.982 | 0 | 2 | 12 | histone H3-K9 methylation |
| GO:0050680 | 0.027 | 5.007 | 1 | 3 | 30 | negative regulation of epithelial cell proliferation |
| GO:0007167 | 0.028 | 1.861 | 8 | 14 | 361 | enzyme linked receptor protein signaling pathway |
| GO:0022610 | 0.028 | 1.722 | 11 | 18 | 503 | biological adhesion |
| GO:0006869 | 0.028 | 2.753 | 2 | 6 | 105 | lipid transport |
| GO:0002009 | 0.028 | 1.906 | 7 | 13 | 327 | morphogenesis of an epithelium |
| GO:0009101 | 0.028 | 2.509 | 3 | 7 | 134 | glycoprotein biosynthetic process |
| GO:0016331 | 0.028 | 2.509 | 3 | 7 | 134 | morphogenesis of embryonic epithelium |
| GO:0051090 | 0.028 | 3.104 | 2 | 5 | 78 | regulation of sequence-specific DNA binding transcription factor activity |
| GO:0007179 | 0.028 | 3.104 | 2 | 5 | 78 | transforming growth factor beta receptor signaling pathway |
| GO:0071845 | 0.029 | 2.725 | 2 | 6 | 106 | cellular component disassembly at cellular level |
| GO:0051493 | 0.030 | 2.310 | 4 | 8 | 166 | regulation of cytoskeleton organization |
| GO:0000398 | 0.030 | 2.310 | 4 | 8 | 166 | nuclear mRNA splicing, via spliceosome |
| GO:0000377 | 0.030 | 2.310 | 4 | 8 | 166 | RNA splicing, via transesterification reactions with bulged adenosine as nucleophile |
| GO:0031290 | 0.030 | 4.828 | 1 | 3 | 31 | retinal ganglion cell axon guidance |
| GO:0071840 | 0.030 | 1.354 | 60 | 72 | 2711 | cellular component organization or biogenesis |
| GO:0048513 | 0.030 | 1.438 | 31 | 41 | 1403 | organ development |
| GO:0032535 | 0.030 | 2.470 | 3 | 7 | 136 | regulation of cellular component size |
| GO:0007243 | 0.030 | 2.002 | 6 | 11 | 263 | intracellular protein kinase cascade |
| GO:0048024 | 0.030 | 3.615 | 1 | 4 | 54 | regulation of nuclear mRNA splicing, via spliceosome |
| GO:0009059 | 0.031 | 1.373 | 47 | 59 | 2156 | macromolecule biosynthetic process |
| GO:0007254 | 0.031 | 3.020 | 2 | 5 | 80 | JNK cascade |
| GO:0022411 | 0.032 | 2.671 | 2 | 6 | 108 | cellular component disassembly |
| GO:0043085 | 0.032 | 1.869 | 7 | 13 | 333 | positive regulation of catalytic activity |
| GO:0021885 | 0.032 | 8.165 | 0 | 2 | 13 | forebrain cell migration |
| GO:0032092 | 0.032 | 8.165 | 0 | 2 | 13 | positive regulation of protein binding |
| GO:0042590 | 0.032 | 8.165 | 0 | 2 | 13 | antigen processing and presentation of exogenous peptide antigen via MHC class I |
| GO:0045478 | 0.032 | 8.165 | 0 | 2 | 13 | fusome organization |
| GO:0022029 | 0.032 | 8.165 | 0 | 2 | 13 | telencephalon cell migration |
| GO:0032881 | 0.032 | 8.165 | 0 | 2 | 13 | regulation of polysaccharide metabolic process |
| GO:0006977 | 0.032 | 8.165 | 0 | 2 | 13 | DNA damage response, signal transduction by p53 class mediator resulting in cell cycle arrest |
| GO:0045913 | 0.032 | 8.165 | 0 | 2 | 13 | positive regulation of carbohydrate metabolic process |
| GO:0072474 | 0.032 | 8.165 | 0 | 2 | 13 | signal transduction involved in mitotic cell cycle G1/S checkpoint |
| GO:0072413 | 0.032 | 8.165 | 0 | 2 | 13 | signal transduction involved in mitotic cell cycle checkpoint |
| GO:0072431 | 0.032 | 8.165 | 0 | 2 | 13 | signal transduction involved in mitotic cell cycle G1/S transition DNA damage checkpoint |
| GO:0072404 | 0.032 | 8.165 | 0 | 2 | 13 | signal transduction involved in G1/S transition checkpoint |
| GO:0051674 | 0.032 | 1.748 | 10 | 16 | 439 | localization of cell |
| GO:0043624 | 0.032 | 3.544 | 1 | 4 | 55 | cellular protein complex disassembly |
| GO:0040008 | 0.032 | 1.863 | 7 | 13 | 334 | regulation of growth |
| GO:0050727 | 0.033 | 4.661 | 1 | 3 | 32 | regulation of inflammatory response |
| GO:0048011 | 0.033 | 4.661 | 1 | 3 | 32 | nerve growth factor receptor signaling pathway |
| GO:0048742 | 0.033 | 4.661 | 1 | 3 | 32 | regulation of skeletal muscle fiber development |
| GO:0051438 | 0.033 | 4.661 | 1 | 3 | 32 | regulation of ubiquitin-protein ligase activity |
| GO:0042325 | 0.033 | 1.913 | 7 | 12 | 300 | regulation of phosphorylation |
| GO:0000375 | 0.033 | 2.266 | 4 | 8 | 169 | RNA splicing, via transesterification reactions |
| GO:0005996 | 0.033 | 2.266 | 4 | 8 | 169 | monosaccharide metabolic process |
| GO:0006935 | 0.033 | 1.812 | 8 | 14 | 370 | chemotaxis |
| GO:0045859 | 0.034 | 2.130 | 4 | 9 | 202 | regulation of protein kinase activity |
| GO:0031347 | 0.034 | 2.619 | 2 | 6 | 110 | regulation of defense response |
| GO:0008340 | 0.034 | 2.941 | 2 | 5 | 82 | determination of adult lifespan |
| GO:0033044 | 0.034 | 3.475 | 1 | 4 | 56 | regulation of chromosome organization |
| GO:0031047 | 0.034 | 3.475 | 1 | 4 | 56 | gene silencing by RNA |
| GO:0022402 | 0.035 | 1.561 | 16 | 24 | 742 | cell cycle process |
| GO:0090304 | 0.035 | 1.345 | 56 | 68 | 2556 | nucleic acid metabolic process |
| GO:0007274 | 0.035 | 4.505 | 1 | 3 | 33 | neuromuscular synaptic transmission |
| GO:0051340 | 0.035 | 4.505 | 1 | 3 | 33 | regulation of ligase activity |
| GO:0048729 | 0.035 | 1.796 | 8 | 14 | 373 | tissue morphogenesis |
| GO:0001503 | 0.036 | 2.903 | 2 | 5 | 83 | ossification |
| GO:0009057 | 0.036 | 1.667 | 11 | 18 | 518 | macromolecule catabolic process |
| GO:0042692 | 0.036 | 2.015 | 5 | 10 | 237 | muscle cell differentiation |
| GO:0030301 | 0.036 | 3.409 | 1 | 4 | 57 | cholesterol transport |
| GO:0043241 | 0.036 | 3.409 | 1 | 4 | 57 | protein complex disassembly |
| GO:0046328 | 0.036 | 3.409 | 1 | 4 | 57 | regulation of JNK cascade |
| GO:0032479 | 0.037 | 7.483 | 0 | 2 | 14 | regulation of type I interferon production |
| GO:0006525 | 0.037 | 7.483 | 0 | 2 | 14 | arginine metabolic process |
| GO:0007016 | 0.037 | 7.483 | 0 | 2 | 14 | cytoskeletal anchoring at plasma membrane |
| GO:0030716 | 0.037 | 7.483 | 0 | 2 | 14 | oocyte fate determination |
| GO:0007294 | 0.037 | 7.483 | 0 | 2 | 14 | germarium-derived oocyte fate determination |
| GO:0051893 | 0.037 | 7.483 | 0 | 2 | 14 | regulation of focal adhesion assembly |
| GO:0090109 | 0.037 | 7.483 | 0 | 2 | 14 | regulation of cell-substrate junction assembly |
| GO:0000216 | 0.037 | 7.483 | 0 | 2 | 14 | M/G1 transition of mitotic cell cycle |
| GO:0031571 | 0.037 | 7.483 | 0 | 2 | 14 | mitotic cell cycle G1/S transition DNA damage checkpoint |
| GO:0055059 | 0.037 | 7.483 | 0 | 2 | 14 | asymmetric neuroblast division |
| GO:0043414 | 0.037 | 2.866 | 2 | 5 | 84 | macromolecule methylation |
| GO:0051403 | 0.037 | 2.866 | 2 | 5 | 84 | stress-activated MAPK cascade |
| GO:0010646 | 0.037 | 1.595 | 14 | 21 | 633 | regulation of cell communication |
| GO:0022008 | 0.038 | 1.489 | 21 | 29 | 943 | neurogenesis |
| GO:0006109 | 0.038 | 4.359 | 1 | 3 | 34 | regulation of carbohydrate metabolic process |
| GO:0034329 | 0.038 | 2.545 | 2 | 6 | 113 | cell junction assembly |
| GO:0022618 | 0.038 | 3.346 | 1 | 4 | 58 | ribonucleoprotein complex assembly |
| GO:0090068 | 0.038 | 3.346 | 1 | 4 | 58 | positive regulation of cell cycle process |
| GO:0042048 | 0.038 | 3.346 | 1 | 4 | 58 | olfactory behavior |
| GO:0046777 | 0.039 | 2.830 | 2 | 5 | 85 | protein autophosphorylation |
| GO:0008360 | 0.039 | 2.521 | 3 | 6 | 114 | regulation of cell shape |
| GO:0023051 | 0.040 | 1.493 | 20 | 28 | 907 | regulation of signaling |
| GO:0009100 | 0.040 | 2.169 | 4 | 8 | 176 | glycoprotein metabolic process |
| GO:0040013 | 0.040 | 3.285 | 1 | 4 | 59 | negative regulation of locomotion |
| GO:0033673 | 0.040 | 3.285 | 1 | 4 | 59 | negative regulation of kinase activity |
| GO:0031098 | 0.041 | 2.795 | 2 | 5 | 86 | stress-activated protein kinase signaling cascade |
| GO:0051054 | 0.041 | 4.222 | 1 | 3 | 35 | positive regulation of DNA metabolic process |
| GO:0045597 | 0.041 | 2.498 | 3 | 6 | 115 | positive regulation of cell differentiation |
| GO:0034661 | 0.042 | 6.907 | 0 | 2 | 15 | ncRNA catabolic process |
| GO:0070918 | 0.042 | 6.907 | 0 | 2 | 15 | production of small RNA involved in gene silencing by RNA |
| GO:0042107 | 0.042 | 6.907 | 0 | 2 | 15 | cytokine metabolic process |
| GO:0035017 | 0.042 | 6.907 | 0 | 2 | 15 | cuticle pattern formation |
| GO:0032606 | 0.042 | 6.907 | 0 | 2 | 15 | type I interferon production |
| GO:0071779 | 0.042 | 6.907 | 0 | 2 | 15 | G1/S transition checkpoint |
| GO:0051341 | 0.042 | 6.907 | 0 | 2 | 15 | regulation of oxidoreductase activity |
| GO:0072395 | 0.042 | 6.907 | 0 | 2 | 15 | signal transduction involved in cell cycle checkpoint |
| GO:0072422 | 0.042 | 6.907 | 0 | 2 | 15 | signal transduction involved in DNA damage checkpoint |
| GO:0072401 | 0.042 | 6.907 | 0 | 2 | 15 | signal transduction involved in DNA integrity checkpoint |
| GO:0051693 | 0.042 | 6.907 | 0 | 2 | 15 | actin filament capping |
| GO:0010743 | 0.042 | 6.907 | 0 | 2 | 15 | regulation of macrophage derived foam cell differentiation |
| GO:0031050 | 0.042 | 6.907 | 0 | 2 | 15 | dsRNA fragmentation |
| GO:0000186 | 0.042 | 6.907 | 0 | 2 | 15 | activation of MAPKK activity |
| GO:0031575 | 0.042 | 6.907 | 0 | 2 | 15 | mitotic cell cycle G1/S transition checkpoint |
| GO:0042089 | 0.042 | 6.907 | 0 | 2 | 15 | cytokine biosynthetic process |
| GO:0042035 | 0.042 | 6.907 | 0 | 2 | 15 | regulation of cytokine biosynthetic process |
| GO:0016476 | 0.042 | 6.907 | 0 | 2 | 15 | regulation of embryonic cell shape |
| GO:0055008 | 0.042 | 6.907 | 0 | 2 | 15 | cardiac muscle tissue morphogenesis |
| GO:0051716 | 0.042 | 1.338 | 50 | 61 | 2277 | cellular response to stimulus |
| GO:0016570 | 0.042 | 2.143 | 4 | 8 | 178 | histone modification |
| GO:0032872 | 0.043 | 3.226 | 1 | 4 | 60 | regulation of stress-activated MAPK cascade |
| GO:0061138 | 0.043 | 3.226 | 1 | 4 | 60 | morphogenesis of a branching epithelium |
| GO:0007635 | 0.043 | 3.226 | 1 | 4 | 60 | chemosensory behavior |
| GO:0000302 | 0.043 | 3.226 | 1 | 4 | 60 | response to reactive oxygen species |
| GO:0070302 | 0.043 | 3.226 | 1 | 4 | 60 | regulation of stress-activated protein kinase signaling cascade |
| GO:0007126 | 0.043 | 2.273 | 3 | 7 | 147 | meiosis |
| GO:0051327 | 0.043 | 2.273 | 3 | 7 | 147 | M phase of meiotic cell cycle |
| GO:2000680 | 0.044 | 44.711 | 0 | 1 | 2 | regulation of rubidium ion transport |
| GO:0060263 | 0.044 | 44.711 | 0 | 1 | 2 | regulation of respiratory burst |
| GO:0042368 | 0.044 | 44.711 | 0 | 1 | 2 | vitamin D biosynthetic process |
| GO:0021873 | 0.044 | 44.711 | 0 | 1 | 2 | forebrain neuroblast division |
| GO:0060740 | 0.044 | 44.711 | 0 | 1 | 2 | prostate gland epithelium morphogenesis |
| GO:0006382 | 0.044 | 44.711 | 0 | 1 | 2 | adenosine to inosine editing |
| GO:0070934 | 0.044 | 44.711 | 0 | 1 | 2 | CRD-mediated mRNA stabilization |
| GO:0060556 | 0.044 | 44.711 | 0 | 1 | 2 | regulation of vitamin D biosynthetic process |
| GO:0016553 | 0.044 | 44.711 | 0 | 1 | 2 | base conversion or substitution editing |
| GO:0060512 | 0.044 | 44.711 | 0 | 1 | 2 | prostate gland morphogenesis |
| GO:0060536 | 0.044 | 44.711 | 0 | 1 | 2 | cartilage morphogenesis |
| GO:0032000 | 0.044 | 44.711 | 0 | 1 | 2 | positive regulation of fatty acid beta-oxidation |
| GO:0060669 | 0.044 | 44.711 | 0 | 1 | 2 | embryonic placenta morphogenesis |
| GO:0019227 | 0.044 | 44.711 | 0 | 1 | 2 | neuronal action potential propagation |
| GO:0006691 | 0.044 | 44.711 | 0 | 1 | 2 | leukotriene metabolic process |
| GO:0009301 | 0.044 | 44.711 | 0 | 1 | 2 | snRNA transcription |
| GO:0045161 | 0.044 | 44.711 | 0 | 1 | 2 | neuronal ion channel clustering |
| GO:0071396 | 0.044 | 44.711 | 0 | 1 | 2 | cellular response to lipid |
| GO:0045110 | 0.044 | 44.711 | 0 | 1 | 2 | intermediate filament bundle assembly |
| GO:0017014 | 0.044 | 44.711 | 0 | 1 | 2 | protein nitrosylation |
| GO:0019544 | 0.044 | 44.711 | 0 | 1 | 2 | arginine catabolic process to glutamate |
| GO:0045780 | 0.044 | 44.711 | 0 | 1 | 2 | positive regulation of bone resorption |
| GO:0045730 | 0.044 | 44.711 | 0 | 1 | 2 | respiratory burst |
| GO:0030033 | 0.044 | 44.711 | 0 | 1 | 2 | microvillus assembly |
| GO:0032512 | 0.044 | 44.711 | 0 | 1 | 2 | regulation of protein phosphatase type 2B activity |
| GO:0045899 | 0.044 | 44.711 | 0 | 1 | 2 | positive regulation of RNA polymerase II transcriptional preinitiation complex assembly |
| GO:0061050 | 0.044 | 44.711 | 0 | 1 | 2 | regulation of cell growth involved in cardiac muscle cell development |
| GO:0061051 | 0.044 | 44.711 | 0 | 1 | 2 | positive regulation of cell growth involved in cardiac muscle cell development |
| GO:0061049 | 0.044 | 44.711 | 0 | 1 | 2 | cell growth involved in cardiac muscle cell development |
| GO:0035313 | 0.044 | 44.711 | 0 | 1 | 2 | wound healing, spreading of epidermal cells |
| GO:0035331 | 0.044 | 44.711 | 0 | 1 | 2 | negative regulation of hippo signaling cascade |
| GO:0038004 | 0.044 | 44.711 | 0 | 1 | 2 | epidermal growth factor receptor ligand maturation |
| GO:0061323 | 0.044 | 44.711 | 0 | 1 | 2 | cell proliferation involved in heart morphogenesis |
| GO:0071542 | 0.044 | 44.711 | 0 | 1 | 2 | dopaminergic neuron differentiation |
| GO:0048845 | 0.044 | 44.711 | 0 | 1 | 2 | venous blood vessel morphogenesis |
| GO:1900673 | 0.044 | 44.711 | 0 | 1 | 2 | olefin metabolic process |
| GO:0043449 | 0.044 | 44.711 | 0 | 1 | 2 | cellular alkene metabolic process |
| GO:0045916 | 0.044 | 44.711 | 0 | 1 | 2 | negative regulation of complement activation |
| GO:0007421 | 0.044 | 44.711 | 0 | 1 | 2 | stomatogastric nervous system development |
| GO:0035826 | 0.044 | 44.711 | 0 | 1 | 2 | rubidium ion transport |
| GO:0002089 | 0.044 | 44.711 | 0 | 1 | 2 | lens morphogenesis in camera-type eye |
| GO:0071985 | 0.044 | 44.711 | 0 | 1 | 2 | multivesicular body sorting pathway |
| GO:0035638 | 0.044 | 44.711 | 0 | 1 | 2 | signal maturation |
| GO:0030656 | 0.044 | 44.711 | 0 | 1 | 2 | regulation of vitamin metabolic process |
| GO:0090034 | 0.044 | 44.711 | 0 | 1 | 2 | regulation of chaperone-mediated protein complex assembly |
| GO:0090035 | 0.044 | 44.711 | 0 | 1 | 2 | positive regulation of chaperone-mediated protein complex assembly |
| GO:0007529 | 0.044 | 44.711 | 0 | 1 | 2 | establishment of synaptic specificity at neuromuscular junction |
| GO:0043651 | 0.044 | 44.711 | 0 | 1 | 2 | linoleic acid metabolic process |
| GO:0051148 | 0.044 | 44.711 | 0 | 1 | 2 | negative regulation of muscle cell differentiation |
| GO:0046321 | 0.044 | 44.711 | 0 | 1 | 2 | positive regulation of fatty acid oxidation |
| GO:0033173 | 0.044 | 44.711 | 0 | 1 | 2 | calcineurin-NFAT signaling cascade |
| GO:0030917 | 0.044 | 44.711 | 0 | 1 | 2 | midbrain-hindbrain boundary development |
| GO:0046137 | 0.044 | 44.711 | 0 | 1 | 2 | negative regulation of vitamin metabolic process |
| GO:0010310 | 0.044 | 44.711 | 0 | 1 | 2 | regulation of hydrogen peroxide metabolic process |
| GO:0090118 | 0.044 | 44.711 | 0 | 1 | 2 | receptor-mediated endocytosis of low-density lipoprotein particle involved in cholesterol transport |
| GO:0090244 | 0.044 | 44.711 | 0 | 1 | 2 | Wnt receptor signaling pathway involved in somitogenesis |
| GO:0046852 | 0.044 | 44.711 | 0 | 1 | 2 | positive regulation of bone remodeling |
| GO:0046599 | 0.044 | 44.711 | 0 | 1 | 2 | regulation of centriole replication |
| GO:0010737 | 0.044 | 44.711 | 0 | 1 | 2 | protein kinase A signaling cascade |
| GO:0010738 | 0.044 | 44.711 | 0 | 1 | 2 | regulation of protein kinase A signaling cascade |
| GO:0080164 | 0.044 | 44.711 | 0 | 1 | 2 | regulation of nitric oxide metabolic process |
| GO:0036124 | 0.044 | 44.711 | 0 | 1 | 2 | histone H3-K9 trimethylation |
| GO:0010561 | 0.044 | 44.711 | 0 | 1 | 2 | negative regulation of glycoprotein biosynthetic process |
| GO:0018206 | 0.044 | 44.711 | 0 | 1 | 2 | peptidyl-methionine modification |
| GO:0036245 | 0.044 | 44.711 | 0 | 1 | 2 | cellular response to menadione |
| GO:0010635 | 0.044 | 44.711 | 0 | 1 | 2 | regulation of mitochondrial fusion |
| GO:0010637 | 0.044 | 44.711 | 0 | 1 | 2 | negative regulation of mitochondrial fusion |
| GO:0008315 | 0.044 | 44.711 | 0 | 1 | 2 | meiotic G2/MI transition |
| GO:0005513 | 0.044 | 44.711 | 0 | 1 | 2 | detection of calcium ion |
| GO:0010957 | 0.044 | 44.711 | 0 | 1 | 2 | negative regulation of vitamin D biosynthetic process |
| GO:0034390 | 0.044 | 44.711 | 0 | 1 | 2 | smooth muscle cell apoptotic process |
| GO:0034391 | 0.044 | 44.711 | 0 | 1 | 2 | regulation of smooth muscle cell apoptotic process |
| GO:0034392 | 0.044 | 44.711 | 0 | 1 | 2 | negative regulation of smooth muscle cell apoptotic process |
| GO:0003301 | 0.044 | 44.711 | 0 | 1 | 2 | physiological cardiac muscle hypertrophy |
| GO:2000136 | 0.044 | 44.711 | 0 | 1 | 2 | regulation of cell proliferation involved in heart morphogenesis |
| GO:2000258 | 0.044 | 44.711 | 0 | 1 | 2 | negative regulation of protein activation cascade |
| GO:0070884 | 0.044 | 44.711 | 0 | 1 | 2 | regulation of calcineurin-NFAT signaling cascade |
| GO:0070841 | 0.044 | 44.711 | 0 | 1 | 2 | inclusion body assembly |
| GO:0070842 | 0.044 | 44.711 | 0 | 1 | 2 | aggresome assembly |
| GO:0070843 | 0.044 | 44.711 | 0 | 1 | 2 | misfolded protein transport |
| GO:0070844 | 0.044 | 44.711 | 0 | 1 | 2 | polyubiquitinated protein transport |
| GO:0070845 | 0.044 | 44.711 | 0 | 1 | 2 | polyubiquitinated misfolded protein transport |
| GO:0003298 | 0.044 | 44.711 | 0 | 1 | 2 | physiological muscle hypertrophy |
| GO:0034105 | 0.044 | 44.711 | 0 | 1 | 2 | positive regulation of tissue remodeling |
| GO:0060421 | 0.044 | 44.711 | 0 | 1 | 2 | positive regulation of heart growth |
| GO:0055023 | 0.044 | 44.711 | 0 | 1 | 2 | positive regulation of cardiac muscle tissue growth |
| GO:0055025 | 0.044 | 44.711 | 0 | 1 | 2 | positive regulation of cardiac muscle tissue development |
| GO:0016569 | 0.044 | 2.130 | 4 | 8 | 179 | covalent chromatin modification |
| GO:0048568 | 0.044 | 2.452 | 3 | 6 | 117 | embryonic organ development |
| GO:0045667 | 0.044 | 4.094 | 1 | 3 | 36 | regulation of osteoblast differentiation |
| GO:0007405 | 0.044 | 4.094 | 1 | 3 | 36 | neuroblast proliferation |
| GO:0030509 | 0.044 | 4.094 | 1 | 3 | 36 | BMP signaling pathway |
| GO:0018958 | 0.044 | 4.094 | 1 | 3 | 36 | phenol-containing compound metabolic process |
| GO:0006032 | 0.044 | 4.094 | 1 | 3 | 36 | chitin catabolic process |
| GO:0043549 | 0.044 | 2.022 | 5 | 9 | 212 | regulation of kinase activity |
| GO:0032259 | 0.044 | 2.727 | 2 | 5 | 88 | methylation |
| GO:0050896 | 0.044 | 1.308 | 73 | 85 | 3334 | response to stimulus |
| GO:0006139 | 0.045 | 1.310 | 68 | 79 | 3070 | nucleobase-containing compound metabolic process |
| GO:0006807 | 0.045 | 1.306 | 71 | 83 | 3249 | nitrogen compound metabolic process |
| GO:0010259 | 0.046 | 2.694 | 2 | 5 | 89 | multicellular organismal aging |
| GO:0065003 | 0.046 | 1.635 | 11 | 17 | 497 | macromolecular complex assembly |
| GO:0045860 | 0.047 | 2.408 | 3 | 6 | 119 | positive regulation of protein kinase activity |
| GO:0007178 | 0.047 | 2.408 | 3 | 6 | 119 | transmembrane receptor protein serine/threonine kinase signaling pathway |
| GO:0009896 | 0.047 | 3.114 | 1 | 4 | 62 | positive regulation of catabolic process |
| GO:0035303 | 0.047 | 3.114 | 1 | 4 | 62 | regulation of dephosphorylation |
| GO:0071826 | 0.047 | 3.114 | 1 | 4 | 62 | ribonucleoprotein complex subunit organization |
| GO:0071902 | 0.047 | 3.114 | 1 | 4 | 62 | positive regulation of protein serine/threonine kinase activity |
| GO:0007612 | 0.047 | 3.114 | 1 | 4 | 62 | learning |
| GO:0008361 | 0.047 | 3.114 | 1 | 4 | 62 | regulation of cell size |
| GO:0034968 | 0.047 | 3.973 | 1 | 3 | 37 | histone lysine methylation |
| GO:0042311 | 0.047 | 6.413 | 0 | 2 | 16 | vasodilation |
| GO:0071359 | 0.047 | 6.413 | 0 | 2 | 16 | cellular response to dsRNA |
| GO:0035001 | 0.047 | 6.413 | 0 | 2 | 16 | dorsal trunk growth, open tracheal system |
| GO:0071158 | 0.047 | 6.413 | 0 | 2 | 16 | positive regulation of cell cycle arrest |
| GO:0032642 | 0.047 | 6.413 | 0 | 2 | 16 | regulation of chemokine production |
| GO:0032602 | 0.047 | 6.413 | 0 | 2 | 16 | chemokine production |
| GO:0030706 | 0.047 | 6.413 | 0 | 2 | 16 | germarium-derived oocyte differentiation |
| GO:0030835 | 0.047 | 6.413 | 0 | 2 | 16 | negative regulation of actin filament depolymerization |
| GO:0030595 | 0.047 | 6.413 | 0 | 2 | 16 | leukocyte chemotaxis |
| GO:0051437 | 0.047 | 6.413 | 0 | 2 | 16 | positive regulation of ubiquitin-protein ligase activity involved in mitotic cell cycle |
| GO:0043666 | 0.047 | 6.413 | 0 | 2 | 16 | regulation of phosphoprotein phosphatase activity |
| GO:0051781 | 0.047 | 6.413 | 0 | 2 | 16 | positive regulation of cell division |
| GO:0002532 | 0.047 | 6.413 | 0 | 2 | 16 | production of molecular mediator involved in inflammatory response |
| GO:0046849 | 0.047 | 6.413 | 0 | 2 | 16 | bone remodeling |
| GO:0010811 | 0.047 | 6.413 | 0 | 2 | 16 | positive regulation of cell-substrate adhesion |
| GO:0008594 | 0.047 | 6.413 | 0 | 2 | 16 | photoreceptor cell morphogenesis |
| GO:0060415 | 0.047 | 6.413 | 0 | 2 | 16 | muscle tissue morphogenesis |
| GO:0006457 | 0.047 | 2.224 | 3 | 7 | 150 | protein folding |
| GO:0044265 | 0.047 | 1.716 | 9 | 14 | 389 | cellular macromolecule catabolic process |
| GO:0000279 | 0.047 | 1.653 | 10 | 16 | 462 | M phase |
| GO:0051301 | 0.048 | 1.679 | 9 | 15 | 426 | cell division |
| GO:0008285 | 0.049 | 2.081 | 4 | 8 | 183 | negative regulation of cell proliferation |
| GO:0007051 | 0.049 | 2.386 | 3 | 6 | 120 | spindle organization |
| GO:0048754 | 0.049 | 3.060 | 1 | 4 | 63 | branching morphogenesis of a tube |
| GO:0001709 | 0.050 | 2.630 | 2 | 5 | 91 | cell fate determination |
| GO:0060429 | 0.050 | 1.701 | 9 | 14 | 392 | epithelium development |
| GO:0040029 | 0.050 | 2.365 | 3 | 6 | 121 | regulation of gene expression, epigenetic |
| GO:1901072 | 0.050 | 3.859 | 1 | 3 | 38 | glucosamine-containing compound catabolic process |
| GO:0090101 | 0.050 | 3.859 | 1 | 3 | 38 | negative regulation of transmembrane receptor protein serine/threonine kinase signaling pathway |
| GO:0000380 | 0.050 | 3.859 | 1 | 3 | 38 | alternative nuclear mRNA splicing, via spliceosome |
| GO:0000381 | 0.050 | 3.859 | 1 | 3 | 38 | regulation of alternative nuclear mRNA splicing, via spliceosome |
| GO:0010033 | 0.050 | 1.553 | 14 | 20 | 616 | response to organic substance |
| GO:0009058 | 0.051 | 1.302 | 62 | 73 | 2823 | biosynthetic process |
| GO:0090092 | 0.052 | 3.009 | 1 | 4 | 64 | regulation of transmembrane receptor protein serine/threonine kinase signaling pathway |
| GO:0007631 | 0.052 | 3.009 | 1 | 4 | 64 | feeding behavior |
| GO:0071900 | 0.052 | 2.345 | 3 | 6 | 122 | regulation of protein serine/threonine kinase activity |
| GO:0043331 | 0.053 | 5.984 | 0 | 2 | 17 | response to dsRNA |
| GO:0048644 | 0.053 | 5.984 | 0 | 2 | 17 | muscle organ morphogenesis |
| GO:0051439 | 0.053 | 5.984 | 0 | 2 | 17 | regulation of ubiquitin-protein ligase activity involved in mitotic cell cycle |
| GO:0060021 | 0.053 | 5.984 | 0 | 2 | 17 | palate development |
| GO:0016321 | 0.053 | 5.984 | 0 | 2 | 17 | female meiosis chromosome segregation |
| GO:0045132 | 0.054 | 3.751 | 1 | 3 | 39 | meiotic chromosome segregation |
| GO:0032101 | 0.054 | 2.324 | 3 | 6 | 123 | regulation of response to external stimulus |
| GO:0033674 | 0.054 | 2.324 | 3 | 6 | 123 | positive regulation of kinase activity |
| GO:0034645 | 0.054 | 1.319 | 47 | 57 | 2137 | cellular macromolecule biosynthetic process |
| GO:0030030 | 0.054 | 1.509 | 15 | 22 | 698 | cell projection organization |
| GO:0032147 | 0.054 | 2.959 | 1 | 4 | 65 | activation of protein kinase activity |
| GO:0048870 | 0.056 | 1.641 | 10 | 15 | 435 | cell motility |
| GO:0016071 | 0.056 | 1.641 | 10 | 15 | 435 | mRNA metabolic process |
| GO:0019216 | 0.056 | 2.541 | 2 | 5 | 94 | regulation of lipid metabolic process |
| GO:0007166 | 0.056 | 1.436 | 21 | 28 | 938 | cell surface receptor signaling pathway |
| GO:0051345 | 0.057 | 2.010 | 4 | 8 | 189 | positive regulation of hydrolase activity |
| GO:0051146 | 0.057 | 2.010 | 4 | 8 | 189 | striated muscle cell differentiation |
| GO:0050807 | 0.057 | 2.911 | 1 | 4 | 66 | regulation of synapse organization |
| GO:0043484 | 0.057 | 2.911 | 1 | 4 | 66 | regulation of RNA splicing |
| GO:0048583 | 0.058 | 1.417 | 22 | 30 | 1020 | regulation of response to stimulus |
| GO:0010740 | 0.058 | 2.512 | 2 | 5 | 95 | positive regulation of intracellular protein kinase cascade |
| GO:0001818 | 0.058 | 5.610 | 0 | 2 | 18 | negative regulation of cytokine production |
| GO:0048841 | 0.058 | 5.610 | 0 | 2 | 18 | regulation of axon extension involved in axon guidance |
| GO:0033227 | 0.058 | 5.610 | 0 | 2 | 18 | dsRNA transport |
| GO:0010821 | 0.058 | 5.610 | 0 | 2 | 18 | regulation of mitochondrion organization |
| GO:0033993 | 0.058 | 5.610 | 0 | 2 | 18 | response to lipid |
| GO:0034109 | 0.058 | 5.610 | 0 | 2 | 18 | homotypic cell-cell adhesion |
| GO:0051347 | 0.059 | 2.265 | 3 | 6 | 126 | positive regulation of transferase activity |
| GO:0005975 | 0.060 | 1.554 | 12 | 18 | 552 | carbohydrate metabolic process |
| GO:0010608 | 0.060 | 1.898 | 5 | 9 | 225 | posttranscriptional regulation of gene expression |
| GO:0042330 | 0.060 | 1.651 | 9 | 14 | 403 | taxis |
| GO:0007409 | 0.060 | 1.651 | 9 | 14 | 403 | axonogenesis |
| GO:0034614 | 0.061 | 3.553 | 1 | 3 | 41 | cellular response to reactive oxygen species |
| GO:0061351 | 0.061 | 3.553 | 1 | 3 | 41 | neural precursor cell proliferation |
| GO:0034330 | 0.061 | 2.246 | 3 | 6 | 127 | cell junction organization |
| GO:0007568 | 0.061 | 2.090 | 3 | 7 | 159 | aging |
| GO:0050684 | 0.062 | 2.820 | 1 | 4 | 68 | regulation of mRNA processing |
| GO:0045596 | 0.063 | 1.966 | 4 | 8 | 193 | negative regulation of cell differentiation |
| GO:0071704 | 0.063 | 1.480 | 16 | 22 | 710 | organic substance metabolic process |
| GO:0051259 | 0.063 | 2.228 | 3 | 6 | 128 | protein oligomerization |
| GO:0048468 | 0.063 | 1.382 | 25 | 33 | 1151 | cell development |
| GO:0021537 | 0.064 | 3.462 | 1 | 3 | 42 | telencephalon development |
| GO:0010810 | 0.064 | 3.462 | 1 | 3 | 42 | regulation of cell-substrate adhesion |
| GO:0032366 | 0.064 | 5.279 | 0 | 2 | 19 | intracellular sterol transport |
| GO:0032367 | 0.064 | 5.279 | 0 | 2 | 19 | intracellular cholesterol transport |
| GO:0050731 | 0.064 | 5.279 | 0 | 2 | 19 | positive regulation of peptidyl-tyrosine phosphorylation |
| GO:0035160 | 0.064 | 5.279 | 0 | 2 | 19 | maintenance of epithelial integrity, open tracheal system |
| GO:0048790 | 0.064 | 5.279 | 0 | 2 | 19 | maintenance of presynaptic active zone structure |
| GO:0033559 | 0.064 | 5.279 | 0 | 2 | 19 | unsaturated fatty acid metabolic process |
| GO:0034381 | 0.064 | 5.279 | 0 | 2 | 19 | plasma lipoprotein particle clearance |
| GO:0055007 | 0.064 | 5.279 | 0 | 2 | 19 | cardiac muscle cell differentiation |
| GO:0060148 | 0.065 | 22.353 | 0 | 1 | 3 | positive regulation of posttranscriptional gene silencing |
| GO:0060135 | 0.065 | 22.353 | 0 | 1 | 3 | maternal process involved in female pregnancy |
| GO:0021794 | 0.065 | 22.353 | 0 | 1 | 3 | thalamus development |
| GO:2000637 | 0.065 | 22.353 | 0 | 1 | 3 | positive regulation of gene silencing by miRNA |
| GO:0060219 | 0.065 | 22.353 | 0 | 1 | 3 | camera-type eye photoreceptor cell differentiation |
| GO:0042362 | 0.065 | 22.353 | 0 | 1 | 3 | fat-soluble vitamin biosynthetic process |
| GO:0060841 | 0.065 | 22.353 | 0 | 1 | 3 | venous blood vessel development |
| GO:0014019 | 0.065 | 22.353 | 0 | 1 | 3 | neuroblast development |
| GO:0034969 | 0.065 | 22.353 | 0 | 1 | 3 | histone arginine methylation |
| GO:0045075 | 0.065 | 22.353 | 0 | 1 | 3 | regulation of interleukin-12 biosynthetic process |
| GO:0045023 | 0.065 | 22.353 | 0 | 1 | 3 | G0 to G1 transition |
| GO:0006703 | 0.065 | 22.353 | 0 | 1 | 3 | estrogen biosynthetic process |
| GO:0021903 | 0.065 | 22.353 | 0 | 1 | 3 | rostrocaudal neural tube patterning |
| GO:0042554 | 0.065 | 22.353 | 0 | 1 | 3 | superoxide anion generation |
| GO:0032148 | 0.065 | 22.353 | 0 | 1 | 3 | activation of protein kinase B activity |
| GO:0014742 | 0.065 | 22.353 | 0 | 1 | 3 | positive regulation of muscle hypertrophy |
| GO:0032769 | 0.065 | 22.353 | 0 | 1 | 3 | negative regulation of monooxygenase activity |
| GO:0042986 | 0.065 | 22.353 | 0 | 1 | 3 | positive regulation of amyloid precursor protein biosynthetic process |
| GO:0006964 | 0.065 | 22.353 | 0 | 1 | 3 | positive regulation of biosynthetic process of antibacterial peptides active against Gram-negative bacteria |
| GO:0032528 | 0.065 | 22.353 | 0 | 1 | 3 | microvillus organization |
| GO:0032516 | 0.065 | 22.353 | 0 | 1 | 3 | positive regulation of phosphoprotein phosphatase activity |
| GO:0045898 | 0.065 | 22.353 | 0 | 1 | 3 | regulation of RNA polymerase II transcriptional preinitiation complex assembly |
| GO:0009853 | 0.065 | 22.353 | 0 | 1 | 3 | photorespiration |
| GO:0035115 | 0.065 | 22.353 | 0 | 1 | 3 | embryonic forelimb morphogenesis |
| GO:0035116 | 0.065 | 22.353 | 0 | 1 | 3 | embryonic hindlimb morphogenesis |
| GO:0035136 | 0.065 | 22.353 | 0 | 1 | 3 | forelimb morphogenesis |
| GO:0035137 | 0.065 | 22.353 | 0 | 1 | 3 | hindlimb morphogenesis |
| GO:0001946 | 0.065 | 22.353 | 0 | 1 | 3 | lymphangiogenesis |
| GO:0032965 | 0.065 | 22.353 | 0 | 1 | 3 | regulation of collagen biosynthetic process |
| GO:0032967 | 0.065 | 22.353 | 0 | 1 | 3 | positive regulation of collagen biosynthetic process |
| GO:0030449 | 0.065 | 22.353 | 0 | 1 | 3 | regulation of complement activation |
| GO:0035261 | 0.065 | 22.353 | 0 | 1 | 3 | external genitalia morphogenesis |
| GO:0048865 | 0.065 | 22.353 | 0 | 1 | 3 | stem cell fate commitment |
| GO:0048843 | 0.065 | 22.353 | 0 | 1 | 3 | negative regulation of axon extension involved in axon guidance |
| GO:0048526 | 0.065 | 22.353 | 0 | 1 | 3 | imaginal disc-derived wing expansion |
| GO:0043147 | 0.065 | 22.353 | 0 | 1 | 3 | meiotic spindle stabilization |
| GO:0007159 | 0.065 | 22.353 | 0 | 1 | 3 | leukocyte cell-cell adhesion |
| GO:0030575 | 0.065 | 22.353 | 0 | 1 | 3 | nuclear body organization |
| GO:0090023 | 0.065 | 22.353 | 0 | 1 | 3 | positive regulation of neutrophil chemotaxis |
| GO:0051451 | 0.065 | 22.353 | 0 | 1 | 3 | myoblast migration |
| GO:0010042 | 0.065 | 22.353 | 0 | 1 | 3 | response to manganese ion |
| GO:0046498 | 0.065 | 22.353 | 0 | 1 | 3 | S-adenosylhomocysteine metabolic process |
| GO:0090381 | 0.065 | 22.353 | 0 | 1 | 3 | regulation of heart induction |
| GO:0002812 | 0.065 | 22.353 | 0 | 1 | 3 | biosynthetic process of antibacterial peptides active against Gram-negative bacteria |
| GO:0002813 | 0.065 | 22.353 | 0 | 1 | 3 | regulation of biosynthetic process of antibacterial peptides active against Gram-negative bacteria |
| GO:0090178 | 0.065 | 22.353 | 0 | 1 | 3 | regulation of establishment of planar polarity involved in neural tube closure |
| GO:0090179 | 0.065 | 22.353 | 0 | 1 | 3 | planar cell polarity pathway involved in neural tube closure |
| GO:0010457 | 0.065 | 22.353 | 0 | 1 | 3 | centriole-centriole cohesion |
| GO:1901320 | 0.065 | 22.353 | 0 | 1 | 3 | negative regulation of heart induction |
| GO:0036303 | 0.065 | 22.353 | 0 | 1 | 3 | lymph vessel morphogenesis |
| GO:0010712 | 0.065 | 22.353 | 0 | 1 | 3 | regulation of collagen metabolic process |
| GO:0010714 | 0.065 | 22.353 | 0 | 1 | 3 | positive regulation of collagen metabolic process |
| GO:0010744 | 0.065 | 22.353 | 0 | 1 | 3 | positive regulation of macrophage derived foam cell differentiation |
| GO:0018198 | 0.065 | 22.353 | 0 | 1 | 3 | peptidyl-cysteine modification |
| GO:0031053 | 0.065 | 22.353 | 0 | 1 | 3 | primary miRNA processing |
| GO:0031017 | 0.065 | 22.353 | 0 | 1 | 3 | exocrine pancreas development |
| GO:0010870 | 0.065 | 22.353 | 0 | 1 | 3 | positive regulation of receptor biosynthetic process |
| GO:0051972 | 0.065 | 22.353 | 0 | 1 | 3 | regulation of telomerase activity |
| GO:0010845 | 0.065 | 22.353 | 0 | 1 | 3 | positive regulation of reciprocal meiotic recombination |
| GO:0036213 | 0.065 | 22.353 | 0 | 1 | 3 | contractile ring contraction |
| GO:0044335 | 0.065 | 22.353 | 0 | 1 | 3 | canonical Wnt receptor signaling pathway involved in neural crest cell differentiation |
| GO:0010613 | 0.065 | 22.353 | 0 | 1 | 3 | positive regulation of cardiac muscle hypertrophy |
| GO:0070316 | 0.065 | 22.353 | 0 | 1 | 3 | regulation of G0 to G1 transition |
| GO:0070317 | 0.065 | 22.353 | 0 | 1 | 3 | negative regulation of G0 to G1 transition |
| GO:0010985 | 0.065 | 22.353 | 0 | 1 | 3 | negative regulation of lipoprotein particle clearance |
| GO:2000257 | 0.065 | 22.353 | 0 | 1 | 3 | regulation of protein activation cascade |
| GO:0034474 | 0.065 | 22.353 | 0 | 1 | 3 | U2 snRNA 3'-end processing |
| GO:0003129 | 0.065 | 22.353 | 0 | 1 | 3 | heart induction |
| GO:0034198 | 0.065 | 22.353 | 0 | 1 | 3 | cellular response to amino acid starvation |
| GO:0070813 | 0.065 | 22.353 | 0 | 1 | 3 | hydrogen sulfide metabolic process |
| GO:0070814 | 0.065 | 22.353 | 0 | 1 | 3 | hydrogen sulfide biosynthetic process |
| GO:0003136 | 0.065 | 22.353 | 0 | 1 | 3 | negative regulation of heart induction by canonical Wnt receptor signaling pathway |
| GO:0000916 | 0.065 | 22.353 | 0 | 1 | 3 | actomyosin contractile ring contraction |
| GO:0042090 | 0.065 | 22.353 | 0 | 1 | 3 | interleukin-12 biosynthetic process |
| GO:0055060 | 0.065 | 22.353 | 0 | 1 | 3 | asymmetric neuroblast division resulting in ganglion mother cell formation |
| GO:0080135 | 0.065 | 2.209 | 3 | 6 | 129 | regulation of cellular response to stress |
| GO:0051641 | 0.066 | 1.390 | 24 | 31 | 1073 | cellular localization |
| GO:0048646 | 0.066 | 1.552 | 11 | 17 | 521 | anatomical structure formation involved in morphogenesis |
| GO:0048514 | 0.067 | 2.191 | 3 | 6 | 130 | blood vessel morphogenesis |
| GO:0006401 | 0.067 | 2.404 | 2 | 5 | 99 | RNA catabolic process |
| GO:0016458 | 0.067 | 2.404 | 2 | 5 | 99 | gene silencing |
| GO:0046348 | 0.068 | 3.375 | 1 | 3 | 43 | amino sugar catabolic process |
| GO:0040017 | 0.069 | 2.379 | 2 | 5 | 100 | positive regulation of locomotion |
| GO:0048863 | 0.069 | 2.379 | 2 | 5 | 100 | stem cell differentiation |
| GO:0006383 | 0.071 | 4.985 | 0 | 2 | 20 | transcription from RNA polymerase III promoter |
| GO:0050714 | 0.071 | 4.985 | 0 | 2 | 20 | positive regulation of protein secretion |
| GO:0048660 | 0.071 | 4.985 | 0 | 2 | 20 | regulation of smooth muscle cell proliferation |
| GO:0048639 | 0.071 | 4.985 | 0 | 2 | 20 | positive regulation of developmental growth |
| GO:0043277 | 0.071 | 4.985 | 0 | 2 | 20 | apoptotic cell clearance |
| GO:0030834 | 0.071 | 4.985 | 0 | 2 | 20 | regulation of actin filament depolymerization |
| GO:0007279 | 0.071 | 4.985 | 0 | 2 | 20 | pole cell formation |
| GO:0033238 | 0.071 | 4.985 | 0 | 2 | 20 | regulation of cellular amine metabolic process |
| GO:0090263 | 0.071 | 4.985 | 0 | 2 | 20 | positive regulation of canonical Wnt receptor signaling pathway |
| GO:0003007 | 0.071 | 2.692 | 2 | 4 | 71 | heart morphogenesis |
| GO:0050865 | 0.072 | 3.292 | 1 | 3 | 44 | regulation of cell activation |
| GO:0045834 | 0.072 | 3.292 | 1 | 3 | 44 | positive regulation of lipid metabolic process |
| GO:0051017 | 0.072 | 3.292 | 1 | 3 | 44 | actin filament bundle assembly |
| GO:0034641 | 0.072 | 1.262 | 71 | 81 | 3228 | cellular nitrogen compound metabolic process |
| GO:0001763 | 0.074 | 2.652 | 2 | 4 | 72 | morphogenesis of a branching structure |
| GO:1901071 | 0.074 | 2.652 | 2 | 4 | 72 | glucosamine-containing compound metabolic process |
| GO:0006954 | 0.074 | 2.329 | 2 | 5 | 102 | inflammatory response |
| GO:0005976 | 0.075 | 2.122 | 3 | 6 | 134 | polysaccharide metabolic process |
| GO:0007293 | 0.076 | 3.213 | 1 | 3 | 45 | germarium-derived egg chamber formation |
| GO:0043524 | 0.076 | 3.213 | 1 | 3 | 45 | negative regulation of neuron apoptotic process |
| GO:1901135 | 0.076 | 1.455 | 15 | 21 | 687 | carbohydrate derivative metabolic process |
| GO:0055123 | 0.076 | 2.305 | 2 | 5 | 103 | digestive system development |
| GO:0048585 | 0.076 | 1.650 | 8 | 12 | 344 | negative regulation of response to stimulus |
| GO:0045995 | 0.077 | 2.614 | 2 | 4 | 73 | regulation of embryonic development |
| GO:0021987 | 0.077 | 4.722 | 0 | 2 | 21 | cerebral cortex development |
| GO:0032355 | 0.077 | 4.722 | 0 | 2 | 21 | response to estradiol stimulus |
| GO:0042559 | 0.077 | 4.722 | 0 | 2 | 21 | pteridine-containing compound biosynthetic process |
| GO:0001837 | 0.077 | 4.722 | 0 | 2 | 21 | epithelial to mesenchymal transition |
| GO:0048659 | 0.077 | 4.722 | 0 | 2 | 21 | smooth muscle cell proliferation |
| GO:0002474 | 0.077 | 4.722 | 0 | 2 | 21 | antigen processing and presentation of peptide antigen via MHC class I |
| GO:0007620 | 0.077 | 4.722 | 0 | 2 | 21 | copulation |
| GO:0010669 | 0.077 | 4.722 | 0 | 2 | 21 | epithelial structure maintenance |
| GO:0034613 | 0.078 | 1.513 | 12 | 17 | 533 | cellular protein localization |
| GO:0034621 | 0.078 | 1.611 | 8 | 13 | 382 | cellular macromolecular complex subunit organization |
| GO:0022414 | 0.078 | 1.363 | 24 | 31 | 1091 | reproductive process |
| GO:0007411 | 0.078 | 1.683 | 7 | 11 | 309 | axon guidance |
| GO:0006022 | 0.079 | 2.281 | 2 | 5 | 104 | aminoglycan metabolic process |
| GO:0016049 | 0.079 | 1.959 | 4 | 7 | 169 | cell growth |
| GO:0003012 | 0.080 | 2.576 | 2 | 4 | 74 | muscle system process |
| GO:0050920 | 0.080 | 3.138 | 1 | 3 | 46 | regulation of chemotaxis |
| GO:0051091 | 0.080 | 3.138 | 1 | 3 | 46 | positive regulation of sequence-specific DNA binding transcription factor activity |
| GO:0044249 | 0.080 | 1.258 | 61 | 70 | 2757 | cellular biosynthetic process |
| GO:0031329 | 0.081 | 1.779 | 5 | 9 | 239 | regulation of cellular catabolic process |
| GO:0043161 | 0.081 | 2.258 | 2 | 5 | 105 | proteasomal ubiquitin-dependent protein catabolic process |
| GO:0044106 | 0.081 | 2.258 | 2 | 5 | 105 | cellular amine metabolic process |
| GO:0043405 | 0.083 | 2.539 | 2 | 4 | 75 | regulation of MAP kinase activity |
| GO:0060538 | 0.083 | 1.934 | 4 | 7 | 171 | skeletal muscle organ development |
| GO:0050808 | 0.083 | 1.934 | 4 | 7 | 171 | synapse organization |
| GO:0032879 | 0.083 | 1.480 | 13 | 18 | 577 | regulation of localization |
| GO:0006119 | 0.083 | 4.485 | 0 | 2 | 22 | oxidative phosphorylation |
| GO:0071453 | 0.083 | 4.485 | 0 | 2 | 22 | cellular response to oxygen levels |
| GO:0071456 | 0.083 | 4.485 | 0 | 2 | 22 | cellular response to hypoxia |
| GO:0045669 | 0.083 | 4.485 | 0 | 2 | 22 | positive regulation of osteoblast differentiation |
| GO:0051099 | 0.083 | 4.485 | 0 | 2 | 22 | positive regulation of binding |
| GO:0090100 | 0.083 | 4.485 | 0 | 2 | 22 | positive regulation of transmembrane receptor protein serine/threonine kinase signaling pathway |
| GO:0036294 | 0.083 | 4.485 | 0 | 2 | 22 | cellular response to decreased oxygen levels |
| GO:0044550 | 0.083 | 4.485 | 0 | 2 | 22 | secondary metabolite biosynthetic process |
| GO:0042446 | 0.084 | 3.066 | 1 | 3 | 47 | hormone biosynthetic process |
| GO:0009451 | 0.084 | 3.066 | 1 | 3 | 47 | RNA modification |
| GO:0030330 | 0.084 | 3.066 | 1 | 3 | 47 | DNA damage response, signal transduction by p53 class mediator |
| GO:0035148 | 0.084 | 3.066 | 1 | 3 | 47 | tube formation |
| GO:0042359 | 0.085 | 14.900 | 0 | 1 | 4 | vitamin D metabolic process |
| GO:0042119 | 0.085 | 14.900 | 0 | 1 | 4 | neutrophil activation |
| GO:0060587 | 0.085 | 14.900 | 0 | 1 | 4 | regulation of lipoprotein lipid oxidation |
| GO:0060588 | 0.085 | 14.900 | 0 | 1 | 4 | negative regulation of lipoprotein lipid oxidation |
| GO:0014029 | 0.085 | 14.900 | 0 | 1 | 4 | neural crest formation |
| GO:0014012 | 0.085 | 14.900 | 0 | 1 | 4 | peripheral nervous system axon regeneration |
| GO:0042760 | 0.085 | 14.900 | 0 | 1 | 4 | very long-chain fatty acid catabolic process |
| GO:0032372 | 0.085 | 14.900 | 0 | 1 | 4 | negative regulation of sterol transport |
| GO:0032375 | 0.085 | 14.900 | 0 | 1 | 4 | negative regulation of cholesterol transport |
| GO:0050748 | 0.085 | 14.900 | 0 | 1 | 4 | negative regulation of lipoprotein metabolic process |
| GO:0032418 | 0.085 | 14.900 | 0 | 1 | 4 | lysosome localization |
| GO:1900015 | 0.085 | 14.900 | 0 | 1 | 4 | regulation of cytokine production involved in inflammatory response |
| GO:1900016 | 0.085 | 14.900 | 0 | 1 | 4 | negative regulation of cytokine production involved in inflammatory response |
| GO:0006610 | 0.085 | 14.900 | 0 | 1 | 4 | ribosomal protein import into nucleus |
| GO:0050691 | 0.085 | 14.900 | 0 | 1 | 4 | regulation of defense response to virus by host |
| GO:0048016 | 0.085 | 14.900 | 0 | 1 | 4 | inositol phosphate-mediated signaling |
| GO:0014743 | 0.085 | 14.900 | 0 | 1 | 4 | regulation of muscle hypertrophy |
| GO:0045109 | 0.085 | 14.900 | 0 | 1 | 4 | intermediate filament organization |
| GO:1900372 | 0.085 | 14.900 | 0 | 1 | 4 | negative regulation of purine nucleotide biosynthetic process |
| GO:0001867 | 0.085 | 14.900 | 0 | 1 | 4 | complement activation, lectin pathway |
| GO:0045725 | 0.085 | 14.900 | 0 | 1 | 4 | positive regulation of glycogen biosynthetic process |
| GO:0001661 | 0.085 | 14.900 | 0 | 1 | 4 | conditioned taste aversion |
| GO:0045821 | 0.085 | 14.900 | 0 | 1 | 4 | positive regulation of glycolysis |
| GO:0045844 | 0.085 | 14.900 | 0 | 1 | 4 | positive regulation of striated muscle tissue development |
| GO:0045836 | 0.085 | 14.900 | 0 | 1 | 4 | positive regulation of meiosis |
| GO:0032655 | 0.085 | 14.900 | 0 | 1 | 4 | regulation of interleukin-12 production |
| GO:0032682 | 0.085 | 14.900 | 0 | 1 | 4 | negative regulation of chemokine production |
| GO:0032615 | 0.085 | 14.900 | 0 | 1 | 4 | interleukin-12 production |
| GO:0048194 | 0.085 | 14.900 | 0 | 1 | 4 | Golgi vesicle budding |
| GO:0001967 | 0.085 | 14.900 | 0 | 1 | 4 | suckling behavior |
| GO:0035149 | 0.085 | 14.900 | 0 | 1 | 4 | lumen formation, open tracheal system |
| GO:0048200 | 0.085 | 14.900 | 0 | 1 | 4 | Golgi transport vesicle coating |
| GO:0048205 | 0.085 | 14.900 | 0 | 1 | 4 | COPI coating of Golgi vesicle |
| GO:0001945 | 0.085 | 14.900 | 0 | 1 | 4 | lymph vessel development |
| GO:0032964 | 0.085 | 14.900 | 0 | 1 | 4 | collagen biosynthetic process |
| GO:1900543 | 0.085 | 14.900 | 0 | 1 | 4 | negative regulation of purine nucleotide metabolic process |
| GO:0071637 | 0.085 | 14.900 | 0 | 1 | 4 | regulation of monocyte chemotactic protein-1 production |
| GO:0071638 | 0.085 | 14.900 | 0 | 1 | 4 | negative regulation of monocyte chemotactic protein-1 production |
| GO:0071605 | 0.085 | 14.900 | 0 | 1 | 4 | monocyte chemotactic protein-1 production |
| GO:0030299 | 0.085 | 14.900 | 0 | 1 | 4 | intestinal cholesterol absorption |
| GO:0045911 | 0.085 | 14.900 | 0 | 1 | 4 | positive regulation of DNA recombination |
| GO:0051029 | 0.085 | 14.900 | 0 | 1 | 4 | rRNA transport |
| GO:0002076 | 0.085 | 14.900 | 0 | 1 | 4 | osteoblast development |
| GO:0035521 | 0.085 | 14.900 | 0 | 1 | 4 | monoubiquitinated histone deubiquitination |
| GO:0035522 | 0.085 | 14.900 | 0 | 1 | 4 | monoubiquitinated histone H2A deubiquitination |
| GO:0030815 | 0.085 | 14.900 | 0 | 1 | 4 | negative regulation of cAMP metabolic process |
| GO:0030818 | 0.085 | 14.900 | 0 | 1 | 4 | negative regulation of cAMP biosynthetic process |
| GO:0030800 | 0.085 | 14.900 | 0 | 1 | 4 | negative regulation of cyclic nucleotide metabolic process |
| GO:0030803 | 0.085 | 14.900 | 0 | 1 | 4 | negative regulation of cyclic nucleotide biosynthetic process |
| GO:0030809 | 0.085 | 14.900 | 0 | 1 | 4 | negative regulation of nucleotide biosynthetic process |
| GO:0030593 | 0.085 | 14.900 | 0 | 1 | 4 | neutrophil chemotaxis |
| GO:0051354 | 0.085 | 14.900 | 0 | 1 | 4 | negative regulation of oxidoreductase activity |
| GO:0090022 | 0.085 | 14.900 | 0 | 1 | 4 | regulation of neutrophil chemotaxis |
| GO:0090004 | 0.085 | 14.900 | 0 | 1 | 4 | positive regulation of establishment of protein localization to plasma membrane |
| GO:0051412 | 0.085 | 14.900 | 0 | 1 | 4 | response to corticosterone stimulus |
| GO:0048934 | 0.085 | 14.900 | 0 | 1 | 4 | peripheral nervous system neuron differentiation |
| GO:0048935 | 0.085 | 14.900 | 0 | 1 | 4 | peripheral nervous system neuron development |
| GO:0035964 | 0.085 | 14.900 | 0 | 1 | 4 | COPI-coated vesicle budding |
| GO:0090344 | 0.085 | 14.900 | 0 | 1 | 4 | negative regulation of cell aging |
| GO:0051894 | 0.085 | 14.900 | 0 | 1 | 4 | positive regulation of focal adhesion assembly |
| GO:0051593 | 0.085 | 14.900 | 0 | 1 | 4 | response to folic acid |
| GO:0002534 | 0.085 | 14.900 | 0 | 1 | 4 | cytokine production involved in inflammatory response |
| GO:0090231 | 0.085 | 14.900 | 0 | 1 | 4 | regulation of spindle checkpoint |
| GO:0046845 | 0.085 | 14.900 | 0 | 1 | 4 | branched duct epithelial cell fate determination, open tracheal system |
| GO:0046500 | 0.085 | 14.900 | 0 | 1 | 4 | S-adenosylmethionine metabolic process |
| GO:0000320 | 0.085 | 14.900 | 0 | 1 | 4 | re-entry into mitotic cell cycle |
| GO:0031063 | 0.085 | 14.900 | 0 | 1 | 4 | regulation of histone deacetylation |
| GO:0046688 | 0.085 | 14.900 | 0 | 1 | 4 | response to copper ion |
| GO:0010869 | 0.085 | 14.900 | 0 | 1 | 4 | regulation of receptor biosynthetic process |
| GO:0010824 | 0.085 | 14.900 | 0 | 1 | 4 | regulation of centrosome duplication |
| GO:0002921 | 0.085 | 14.900 | 0 | 1 | 4 | negative regulation of humoral immune response |
| GO:0044319 | 0.085 | 14.900 | 0 | 1 | 4 | wound healing, spreading of cells |
| GO:2000095 | 0.085 | 14.900 | 0 | 1 | 4 | regulation of Wnt receptor signaling pathway, planar cell polarity pathway |
| GO:2000097 | 0.085 | 14.900 | 0 | 1 | 4 | regulation of smooth muscle cell-matrix adhesion |
| GO:2000098 | 0.085 | 14.900 | 0 | 1 | 4 | negative regulation of smooth muscle cell-matrix adhesion |
| GO:2000050 | 0.085 | 14.900 | 0 | 1 | 4 | regulation of non-canonical Wnt receptor signaling pathway |
| GO:0000290 | 0.085 | 14.900 | 0 | 1 | 4 | deadenylation-dependent decapping of nuclear-transcribed mRNA |
| GO:0010611 | 0.085 | 14.900 | 0 | 1 | 4 | regulation of cardiac muscle hypertrophy |
| GO:0044253 | 0.085 | 14.900 | 0 | 1 | 4 | positive regulation of multicellular organismal metabolic process |
| GO:0044241 | 0.085 | 14.900 | 0 | 1 | 4 | lipid digestion |
| GO:0044246 | 0.085 | 14.900 | 0 | 1 | 4 | regulation of multicellular organismal metabolic process |
| GO:0010922 | 0.085 | 14.900 | 0 | 1 | 4 | positive regulation of phosphatase activity |
| GO:2000402 | 0.085 | 14.900 | 0 | 1 | 4 | negative regulation of lymphocyte migration |
| GO:2000405 | 0.085 | 14.900 | 0 | 1 | 4 | negative regulation of T cell migration |
| GO:0034379 | 0.085 | 14.900 | 0 | 1 | 4 | very-low-density lipoprotein particle assembly |
| GO:0070723 | 0.085 | 14.900 | 0 | 1 | 4 | response to cholesterol |
| GO:0034472 | 0.085 | 14.900 | 0 | 1 | 4 | snRNA 3'-end processing |
| GO:0034442 | 0.085 | 14.900 | 0 | 1 | 4 | regulation of lipoprotein oxidation |
| GO:0034443 | 0.085 | 14.900 | 0 | 1 | 4 | negative regulation of lipoprotein oxidation |
| GO:0034447 | 0.085 | 14.900 | 0 | 1 | 4 | very-low-density lipoprotein particle clearance |
| GO:0034439 | 0.085 | 14.900 | 0 | 1 | 4 | lipoprotein lipid oxidation |
| GO:0070875 | 0.085 | 14.900 | 0 | 1 | 4 | positive regulation of glycogen metabolic process |
| GO:2000725 | 0.085 | 14.900 | 0 | 1 | 4 | regulation of cardiac muscle cell differentiation |
| GO:0034227 | 0.085 | 14.900 | 0 | 1 | 4 | tRNA thio-modification |
| GO:0006068 | 0.085 | 14.900 | 0 | 1 | 4 | ethanol catabolic process |
| GO:0048609 | 0.086 | 1.432 | 15 | 21 | 697 | multicellular organismal reproductive process |
| GO:0010876 | 0.086 | 2.041 | 3 | 6 | 139 | lipid localization |
| GO:0006040 | 0.086 | 2.504 | 2 | 4 | 76 | amino sugar metabolic process |
| GO:0090066 | 0.086 | 1.824 | 5 | 8 | 207 | regulation of anatomical structure size |
| GO:0031667 | 0.086 | 1.824 | 5 | 8 | 207 | response to nutrient levels |
| GO:0010038 | 0.087 | 2.213 | 2 | 5 | 107 | response to metal ion |
| GO:2000026 | 0.087 | 1.486 | 12 | 17 | 542 | regulation of multicellular organismal development |
| GO:0006511 | 0.087 | 1.748 | 5 | 9 | 243 | ubiquitin-dependent protein catabolic process |
| GO:0001649 | 0.088 | 2.998 | 1 | 3 | 48 | osteoblast differentiation |
| GO:0048872 | 0.088 | 2.998 | 1 | 3 | 48 | homeostasis of number of cells |
| GO:1901215 | 0.088 | 2.998 | 1 | 3 | 48 | negative regulation of neuron death |
| GO:0003008 | 0.089 | 1.385 | 19 | 25 | 860 | system process |
| GO:0032504 | 0.089 | 1.425 | 15 | 21 | 700 | multicellular organism reproduction |
| GO:0019941 | 0.089 | 1.740 | 5 | 9 | 244 | modification-dependent protein catabolic process |
| GO:0031400 | 0.089 | 2.192 | 2 | 5 | 108 | negative regulation of protein modification process |
| GO:0071822 | 0.090 | 1.542 | 9 | 14 | 429 | protein complex subunit organization |
| GO:0014031 | 0.090 | 4.271 | 1 | 2 | 23 | mesenchymal cell development |
| GO:0060606 | 0.090 | 4.271 | 1 | 2 | 23 | tube closure |
| GO:0001843 | 0.090 | 4.271 | 1 | 2 | 23 | neural tube closure |
| GO:0045766 | 0.090 | 4.271 | 1 | 2 | 23 | positive regulation of angiogenesis |
| GO:0030042 | 0.090 | 4.271 | 1 | 2 | 23 | actin filament depolymerization |
| GO:0032526 | 0.090 | 4.271 | 1 | 2 | 23 | response to retinoic acid |
| GO:0030837 | 0.090 | 4.271 | 1 | 2 | 23 | negative regulation of actin filament polymerization |
| GO:0007277 | 0.090 | 4.271 | 1 | 2 | 23 | pole cell development |
| GO:0033189 | 0.090 | 4.271 | 1 | 2 | 23 | response to vitamin A |
| GO:0031145 | 0.090 | 4.271 | 1 | 2 | 23 | anaphase-promoting complex-dependent proteasomal ubiquitin-dependent protein catabolic process |
| GO:0070585 | 0.090 | 4.271 | 1 | 2 | 23 | protein localization to mitochondrion |
| GO:0016055 | 0.091 | 1.887 | 4 | 7 | 175 | Wnt receptor signaling pathway |
| GO:0007423 | 0.091 | 1.538 | 9 | 14 | 430 | sensory organ development |
| GO:0007610 | 0.092 | 1.493 | 11 | 16 | 507 | behavior |
| GO:0019098 | 0.092 | 2.170 | 2 | 5 | 109 | reproductive behavior |
| GO:0042594 | 0.092 | 2.170 | 2 | 5 | 109 | response to starvation |
| GO:0043009 | 0.095 | 1.778 | 5 | 8 | 212 | chordate embryonic development |
| GO:0009991 | 0.095 | 1.778 | 5 | 8 | 212 | response to extracellular stimulus |
| GO:0007528 | 0.096 | 2.403 | 2 | 4 | 79 | neuromuscular junction development |
| GO:0007619 | 0.096 | 2.403 | 2 | 4 | 79 | courtship behavior |
| GO:0071310 | 0.096 | 1.581 | 8 | 12 | 358 | cellular response to organic substance |
| GO:0006412 | 0.096 | 1.523 | 10 | 14 | 434 | translation |
| GO:0014020 | 0.097 | 4.077 | 1 | 2 | 24 | primary neural tube formation |
| GO:0032365 | 0.097 | 4.077 | 1 | 2 | 24 | intracellular lipid transport |
| GO:0032436 | 0.097 | 4.077 | 1 | 2 | 24 | positive regulation of proteasomal ubiquitin-dependent protein catabolic process |
| GO:0050806 | 0.097 | 4.077 | 1 | 2 | 24 | positive regulation of synaptic transmission |
| GO:0007229 | 0.097 | 4.077 | 1 | 2 | 24 | integrin-mediated signaling pathway |
| GO:0051971 | 0.097 | 4.077 | 1 | 2 | 24 | positive regulation of transmission of nerve impulse |
| GO:0010594 | 0.097 | 4.077 | 1 | 2 | 24 | regulation of endothelial cell migration |
| GO:2000045 | 0.097 | 4.077 | 1 | 2 | 24 | regulation of G1/S transition of mitotic cell cycle |
| GO:0006094 | 0.097 | 4.077 | 1 | 2 | 24 | gluconeogenesis |
| GO:0030336 | 0.097 | 2.869 | 1 | 3 | 50 | negative regulation of cell migration |
| GO:0016339 | 0.097 | 2.869 | 1 | 3 | 50 | calcium-dependent cell-cell adhesion |
| GO:0001568 | 0.098 | 1.965 | 3 | 6 | 144 | blood vessel development |
| GO:0009887 | 0.098 | 1.428 | 14 | 19 | 630 | organ morphogenesis |
| GO:0043632 | 0.100 | 1.695 | 5 | 9 | 250 | modification-dependent macromolecule catabolic process |
| GO:0072331 | 0.101 | 2.809 | 1 | 3 | 51 | signal transduction by p53 class mediator |
| GO:0022613 | 0.101 | 1.752 | 5 | 8 | 215 | ribonucleoprotein complex biogenesis |
| GO:0006461 | 0.102 | 1.532 | 9 | 13 | 400 | protein complex assembly |
| GO:0006164 | 0.103 | 2.340 | 2 | 4 | 81 | purine nucleotide biosynthetic process |
| GO:0010498 | 0.103 | 2.089 | 2 | 5 | 113 | proteasomal protein catabolic process |
| GO:0021543 | 0.104 | 3.899 | 1 | 2 | 25 | pallium development |
| GO:0042743 | 0.104 | 3.899 | 1 | 2 | 25 | hydrogen peroxide metabolic process |
| GO:0050867 | 0.104 | 3.899 | 1 | 2 | 25 | positive regulation of cell activation |
| GO:0048762 | 0.104 | 3.899 | 1 | 2 | 25 | mesenchymal cell differentiation |
| GO:0033002 | 0.104 | 3.899 | 1 | 2 | 25 | muscle cell proliferation |
| GO:0051443 | 0.104 | 3.899 | 1 | 2 | 25 | positive regulation of ubiquitin-protein ligase activity |
| GO:0070301 | 0.104 | 3.899 | 1 | 2 | 25 | cellular response to hydrogen peroxide |
| GO:0010906 | 0.104 | 3.899 | 1 | 2 | 25 | regulation of glucose metabolic process |
| GO:0008380 | 0.104 | 1.629 | 6 | 10 | 289 | RNA splicing |
| GO:0022403 | 0.105 | 1.427 | 13 | 18 | 596 | cell cycle phase |
| GO:0006396 | 0.105 | 1.441 | 12 | 17 | 557 | RNA processing |
| GO:0060147 | 0.105 | 11.174 | 0 | 1 | 5 | regulation of posttranscriptional gene silencing |
| GO:0060261 | 0.105 | 11.174 | 0 | 1 | 5 | positive regulation of transcription initiation from RNA polymerase II promoter |
| GO:0006388 | 0.105 | 11.174 | 0 | 1 | 5 | tRNA splicing, via endonucleolytic cleavage and ligation |
| GO:0042438 | 0.105 | 11.174 | 0 | 1 | 5 | melanin biosynthetic process |
| GO:0006488 | 0.105 | 11.174 | 0 | 1 | 5 | dolichol-linked oligosaccharide biosynthetic process |
| GO:0042160 | 0.105 | 11.174 | 0 | 1 | 5 | lipoprotein modification |
| GO:0042161 | 0.105 | 11.174 | 0 | 1 | 5 | lipoprotein oxidation |
| GO:0006120 | 0.105 | 11.174 | 0 | 1 | 5 | mitochondrial electron transport, NADH to ubiquinone |
| GO:0032007 | 0.105 | 11.174 | 0 | 1 | 5 | negative regulation of TOR signaling cascade |
| GO:0019344 | 0.105 | 11.174 | 0 | 1 | 5 | cysteine biosynthetic process |
| GO:0042772 | 0.105 | 11.174 | 0 | 1 | 5 | DNA damage response, signal transduction resulting in transcription |
| GO:0006729 | 0.105 | 11.174 | 0 | 1 | 5 | tetrahydrobiopterin biosynthetic process |
| GO:0032369 | 0.105 | 11.174 | 0 | 1 | 5 | negative regulation of lipid transport |
| GO:0060964 | 0.105 | 11.174 | 0 | 1 | 5 | regulation of gene silencing by miRNA |
| GO:0006590 | 0.105 | 11.174 | 0 | 1 | 5 | thyroid hormone generation |
| GO:0032410 | 0.105 | 11.174 | 0 | 1 | 5 | negative regulation of transporter activity |
| GO:0050881 | 0.105 | 11.174 | 0 | 1 | 5 | musculoskeletal movement |
| GO:0050879 | 0.105 | 11.174 | 0 | 1 | 5 | multicellular organismal movement |
| GO:0006655 | 0.105 | 11.174 | 0 | 1 | 5 | phosphatidylglycerol biosynthetic process |
| GO:0022028 | 0.105 | 11.174 | 0 | 1 | 5 | tangential migration from the subventricular zone to the olfactory bulb |
| GO:0050665 | 0.105 | 11.174 | 0 | 1 | 5 | hydrogen peroxide biosynthetic process |
| GO:0001702 | 0.105 | 11.174 | 0 | 1 | 5 | gastrulation with mouth forming second |
| GO:0009115 | 0.105 | 11.174 | 0 | 1 | 5 | xanthine catabolic process |
| GO:0006978 | 0.105 | 11.174 | 0 | 1 | 5 | DNA damage response, signal transduction by p53 class mediator resulting in transcription of p21 class mediator |
| GO:0001569 | 0.105 | 11.174 | 0 | 1 | 5 | patterning of blood vessels |
| GO:0032800 | 0.105 | 11.174 | 0 | 1 | 5 | receptor biosynthetic process |
| GO:0050922 | 0.105 | 11.174 | 0 | 1 | 5 | negative regulation of chemotaxis |
| GO:0071260 | 0.105 | 11.174 | 0 | 1 | 5 | cellular response to mechanical stimulus |
| GO:0071218 | 0.105 | 11.174 | 0 | 1 | 5 | cellular response to misfolded protein |
| GO:0045599 | 0.105 | 11.174 | 0 | 1 | 5 | negative regulation of fat cell differentiation |
| GO:0048255 | 0.105 | 11.174 | 0 | 1 | 5 | mRNA stabilization |
| GO:0061302 | 0.105 | 11.174 | 0 | 1 | 5 | smooth muscle cell-matrix adhesion |
| GO:0035262 | 0.105 | 11.174 | 0 | 1 | 5 | gonad morphogenesis |
| GO:0032926 | 0.105 | 11.174 | 0 | 1 | 5 | negative regulation of activin receptor signaling pathway |
| GO:0043487 | 0.105 | 11.174 | 0 | 1 | 5 | regulation of RNA stability |
| GO:0043488 | 0.105 | 11.174 | 0 | 1 | 5 | regulation of mRNA stability |
| GO:0043489 | 0.105 | 11.174 | 0 | 1 | 5 | RNA stabilization |
| GO:0045980 | 0.105 | 11.174 | 0 | 1 | 5 | negative regulation of nucleotide metabolic process |
| GO:0045923 | 0.105 | 11.174 | 0 | 1 | 5 | positive regulation of fatty acid metabolic process |
| GO:0009997 | 0.105 | 11.174 | 0 | 1 | 5 | negative regulation of cardioblast cell fate specification |
| GO:0048521 | 0.105 | 11.174 | 0 | 1 | 5 | negative regulation of behavior |
| GO:0030238 | 0.105 | 11.174 | 0 | 1 | 5 | male sex determination |
| GO:0002097 | 0.105 | 11.174 | 0 | 1 | 5 | tRNA wobble base modification |
| GO:0002098 | 0.105 | 11.174 | 0 | 1 | 5 | tRNA wobble uridine modification |
| GO:0002053 | 0.105 | 11.174 | 0 | 1 | 5 | positive regulation of mesenchymal cell proliferation |
| GO:0048636 | 0.105 | 11.174 | 0 | 1 | 5 | positive regulation of muscle organ development |
| GO:0007110 | 0.105 | 11.174 | 0 | 1 | 5 | cytokinesis after meiosis I |
| GO:0007111 | 0.105 | 11.174 | 0 | 1 | 5 | cytokinesis after meiosis II |
| GO:0030517 | 0.105 | 11.174 | 0 | 1 | 5 | negative regulation of axon extension |
| GO:0051385 | 0.105 | 11.174 | 0 | 1 | 5 | response to mineralocorticoid stimulus |
| GO:0043578 | 0.105 | 11.174 | 0 | 1 | 5 | nuclear matrix organization |
| GO:0002446 | 0.105 | 11.174 | 0 | 1 | 5 | neutrophil mediated immunity |
| GO:0051155 | 0.105 | 11.174 | 0 | 1 | 5 | positive regulation of striated muscle cell differentiation |
| GO:0007625 | 0.105 | 11.174 | 0 | 1 | 5 | grooming behavior |
| GO:0090311 | 0.105 | 11.174 | 0 | 1 | 5 | regulation of protein deacetylation |
| GO:0030949 | 0.105 | 11.174 | 0 | 1 | 5 | positive regulation of vascular endothelial growth factor receptor signaling pathway |
| GO:0051788 | 0.105 | 11.174 | 0 | 1 | 5 | response to misfolded protein |
| GO:0046110 | 0.105 | 11.174 | 0 | 1 | 5 | xanthine metabolic process |
| GO:0046146 | 0.105 | 11.174 | 0 | 1 | 5 | tetrahydrobiopterin metabolic process |
| GO:0010463 | 0.105 | 11.174 | 0 | 1 | 5 | mesenchymal cell proliferation |
| GO:0010464 | 0.105 | 11.174 | 0 | 1 | 5 | regulation of mesenchymal cell proliferation |
| GO:0090136 | 0.105 | 11.174 | 0 | 1 | 5 | epithelial cell-cell adhesion |
| GO:0090292 | 0.105 | 11.174 | 0 | 1 | 5 | nuclear matrix anchoring at nuclear membrane |
| GO:0090286 | 0.105 | 11.174 | 0 | 1 | 5 | cytoskeletal anchoring at nuclear membrane |
| GO:0002548 | 0.105 | 11.174 | 0 | 1 | 5 | monocyte chemotaxis |
| GO:0033563 | 0.105 | 11.174 | 0 | 1 | 5 | dorsal/ventral axon guidance |
| GO:0002673 | 0.105 | 11.174 | 0 | 1 | 5 | regulation of acute inflammatory response |
| GO:0000394 | 0.105 | 11.174 | 0 | 1 | 5 | RNA splicing, via endonucleolytic cleavage and ligation |
| GO:0008053 | 0.105 | 11.174 | 0 | 1 | 5 | mitochondrial fusion |
| GO:0046655 | 0.105 | 11.174 | 0 | 1 | 5 | folic acid metabolic process |
| GO:0010823 | 0.105 | 11.174 | 0 | 1 | 5 | negative regulation of mitochondrion organization |
| GO:0010586 | 0.105 | 11.174 | 0 | 1 | 5 | miRNA metabolic process |
| GO:0010520 | 0.105 | 11.174 | 0 | 1 | 5 | regulation of reciprocal meiotic recombination |
| GO:2000044 | 0.105 | 11.174 | 0 | 1 | 5 | negative regulation of cardiac cell fate specification |
| GO:0010955 | 0.105 | 11.174 | 0 | 1 | 5 | negative regulation of protein processing |
| GO:0034389 | 0.105 | 11.174 | 0 | 1 | 5 | lipid particle organization |
| GO:0034310 | 0.105 | 11.174 | 0 | 1 | 5 | primary alcohol catabolic process |
| GO:2000144 | 0.105 | 11.174 | 0 | 1 | 5 | positive regulation of DNA-dependent transcription, initiation |
| GO:0060395 | 0.105 | 11.174 | 0 | 1 | 5 | SMAD protein signal transduction |
| GO:0042036 | 0.105 | 11.174 | 0 | 1 | 5 | negative regulation of cytokine biosynthetic process |
| GO:0071843 | 0.105 | 1.674 | 6 | 9 | 253 | cellular component biogenesis at cellular level |
| GO:0048736 | 0.105 | 1.674 | 6 | 9 | 253 | appendage development |
| GO:0006997 | 0.106 | 2.752 | 1 | 3 | 52 | nucleus organization |
| GO:0048562 | 0.106 | 2.752 | 1 | 3 | 52 | embryonic organ morphogenesis |
| GO:0043281 | 0.106 | 2.752 | 1 | 3 | 52 | regulation of cysteine-type endopeptidase activity involved in apoptotic process |
| GO:2000146 | 0.106 | 2.752 | 1 | 3 | 52 | negative regulation of cell motility |
| GO:0009966 | 0.106 | 1.368 | 18 | 23 | 797 | regulation of signal transduction |
| GO:0050803 | 0.106 | 2.309 | 2 | 4 | 82 | regulation of synapse structure and activity |
| GO:0006397 | 0.107 | 1.548 | 8 | 12 | 365 | mRNA processing |
| GO:0070271 | 0.107 | 1.520 | 9 | 13 | 403 | protein complex biogenesis |
| GO:0045786 | 0.108 | 1.726 | 5 | 8 | 218 | negative regulation of cell cycle |
| GO:0007519 | 0.108 | 1.909 | 3 | 6 | 148 | skeletal muscle tissue development |
| GO:0048592 | 0.109 | 1.660 | 6 | 9 | 255 | eye morphogenesis |
| GO:0033365 | 0.109 | 1.660 | 6 | 9 | 255 | protein localization to organelle |
| GO:0007127 | 0.111 | 2.696 | 1 | 3 | 53 | meiosis I |
| GO:0035966 | 0.111 | 2.696 | 1 | 3 | 53 | response to topologically incorrect protein |
| GO:0014017 | 0.111 | 3.736 | 1 | 2 | 26 | neuroblast fate commitment |
| GO:0050863 | 0.111 | 3.736 | 1 | 2 | 26 | regulation of T cell activation |
| GO:0032272 | 0.111 | 3.736 | 1 | 2 | 26 | negative regulation of protein polymerization |
| GO:0071825 | 0.111 | 3.736 | 1 | 2 | 26 | protein-lipid complex subunit organization |
| GO:0071827 | 0.111 | 3.736 | 1 | 2 | 26 | plasma lipoprotein particle organization |
| GO:0035162 | 0.111 | 3.736 | 1 | 2 | 26 | embryonic hemopoiesis |
| GO:0051351 | 0.111 | 3.736 | 1 | 2 | 26 | positive regulation of ligase activity |
| GO:0060348 | 0.111 | 3.736 | 1 | 2 | 26 | bone development |
| GO:0023057 | 0.111 | 1.568 | 7 | 11 | 330 | negative regulation of signaling |
| GO:0009308 | 0.112 | 2.032 | 3 | 5 | 116 | amine metabolic process |
| GO:0007017 | 0.113 | 1.503 | 9 | 13 | 407 | microtubule-based process |
| GO:0034470 | 0.113 | 1.779 | 4 | 7 | 185 | ncRNA processing |
| GO:0000003 | 0.114 | 1.303 | 25 | 31 | 1133 | reproduction |
| GO:0070727 | 0.114 | 1.422 | 12 | 17 | 564 | cellular macromolecule localization |
| GO:0051262 | 0.115 | 2.643 | 1 | 3 | 54 | protein tetramerization |
| GO:0008284 | 0.115 | 1.869 | 3 | 6 | 151 | positive regulation of cell proliferation |
| GO:0033554 | 0.116 | 1.369 | 16 | 21 | 725 | cellular response to stress |
| GO:0048741 | 0.117 | 2.223 | 2 | 4 | 85 | skeletal muscle fiber development |
| GO:0016246 | 0.118 | 3.586 | 1 | 2 | 27 | RNA interference |
| GO:0014016 | 0.118 | 3.586 | 1 | 2 | 27 | neuroblast differentiation |
| GO:0032006 | 0.118 | 3.586 | 1 | 2 | 27 | regulation of TOR signaling cascade |
| GO:0019319 | 0.118 | 3.586 | 1 | 2 | 27 | hexose biosynthetic process |
| GO:0042733 | 0.118 | 3.586 | 1 | 2 | 27 | embryonic digit morphogenesis |
| GO:0042595 | 0.118 | 3.586 | 1 | 2 | 27 | behavioral response to starvation |
| GO:0050880 | 0.118 | 3.586 | 1 | 2 | 27 | regulation of blood vessel size |
| GO:0035112 | 0.118 | 3.586 | 1 | 2 | 27 | genitalia morphogenesis |
| GO:0001952 | 0.118 | 3.586 | 1 | 2 | 27 | regulation of cell-matrix adhesion |
| GO:0046364 | 0.118 | 3.586 | 1 | 2 | 27 | monosaccharide biosynthetic process |
| GO:0046667 | 0.118 | 3.586 | 1 | 2 | 27 | compound eye retinal cell programmed cell death |
| GO:0010883 | 0.118 | 3.586 | 1 | 2 | 27 | regulation of lipid storage |
| GO:0060326 | 0.118 | 3.586 | 1 | 2 | 27 | cell chemotaxis |
| GO:0031646 | 0.118 | 3.586 | 1 | 2 | 27 | positive regulation of neurological system process |
| GO:0001775 | 0.118 | 1.856 | 3 | 6 | 152 | cell activation |
| GO:0001944 | 0.118 | 1.856 | 3 | 6 | 152 | vasculature development |
| GO:0051603 | 0.119 | 1.626 | 6 | 9 | 260 | proteolysis involved in cellular protein catabolic process |
| GO:0034599 | 0.120 | 2.592 | 1 | 3 | 55 | cellular response to oxidative stress |
| GO:0006469 | 0.120 | 2.592 | 1 | 3 | 55 | negative regulation of protein kinase activity |
| GO:0050770 | 0.120 | 2.592 | 1 | 3 | 55 | regulation of axonogenesis |
| GO:0019915 | 0.120 | 2.592 | 1 | 3 | 55 | lipid storage |
| GO:0051271 | 0.120 | 2.592 | 1 | 3 | 55 | negative regulation of cellular component movement |
| GO:0006470 | 0.121 | 2.196 | 2 | 4 | 86 | protein dephosphorylation |
| GO:0055114 | 0.121 | 1.619 | 6 | 9 | 261 | oxidation-reduction process |
| GO:0001558 | 0.121 | 1.977 | 3 | 5 | 119 | regulation of cell growth |
| GO:0065008 | 0.123 | 1.277 | 28 | 34 | 1268 | regulation of biological quality |
| GO:0010648 | 0.123 | 1.533 | 7 | 11 | 337 | negative regulation of cell communication |
| GO:0007617 | 0.124 | 2.169 | 2 | 4 | 87 | mating behavior |
| GO:0001894 | 0.125 | 2.543 | 1 | 3 | 56 | tissue homeostasis |
| GO:0021772 | 0.125 | 8.938 | 0 | 1 | 6 | olfactory bulb development |
| GO:0031998 | 0.125 | 8.938 | 0 | 1 | 6 | regulation of fatty acid beta-oxidation |
| GO:0060232 | 0.125 | 8.938 | 0 | 1 | 6 | delamination |
| GO:0021591 | 0.125 | 8.938 | 0 | 1 | 6 | ventricular system development |
| GO:0042403 | 0.125 | 8.938 | 0 | 1 | 6 | thyroid hormone metabolic process |
| GO:0060631 | 0.125 | 8.938 | 0 | 1 | 6 | regulation of meiosis I |
| GO:0042810 | 0.125 | 8.938 | 0 | 1 | 6 | pheromone metabolic process |
| GO:0042811 | 0.125 | 8.938 | 0 | 1 | 6 | pheromone biosynthetic process |
| GO:0006563 | 0.125 | 8.938 | 0 | 1 | 6 | L-serine metabolic process |
| GO:0045124 | 0.125 | 8.938 | 0 | 1 | 6 | regulation of bone resorption |
| GO:0014823 | 0.125 | 8.938 | 0 | 1 | 6 | response to activity |
| GO:0045668 | 0.125 | 8.938 | 0 | 1 | 6 | negative regulation of osteoblast differentiation |
| GO:0035293 | 0.125 | 8.938 | 0 | 1 | 6 | chitin-based larval cuticle pattern formation |
| GO:0030423 | 0.125 | 8.938 | 0 | 1 | 6 | targeting of mRNA for destruction involved in RNA interference |
| GO:0051085 | 0.125 | 8.938 | 0 | 1 | 6 | chaperone mediated protein folding requiring cofactor |
| GO:0043162 | 0.125 | 8.938 | 0 | 1 | 6 | ubiquitin-dependent protein catabolic process via the multivesicular body sorting pathway |
| GO:0030854 | 0.125 | 8.938 | 0 | 1 | 6 | positive regulation of granulocyte differentiation |
| GO:0090003 | 0.125 | 8.938 | 0 | 1 | 6 | regulation of establishment of protein localization to plasma membrane |
| GO:0043502 | 0.125 | 8.938 | 0 | 1 | 6 | regulation of muscle adaptation |
| GO:0051131 | 0.125 | 8.938 | 0 | 1 | 6 | chaperone-mediated protein complex assembly |
| GO:0051895 | 0.125 | 8.938 | 0 | 1 | 6 | negative regulation of focal adhesion assembly |
| GO:0002690 | 0.125 | 8.938 | 0 | 1 | 6 | positive regulation of leukocyte chemotaxis |
| GO:0002687 | 0.125 | 8.938 | 0 | 1 | 6 | positive regulation of leukocyte migration |
| GO:0000389 | 0.125 | 8.938 | 0 | 1 | 6 | nuclear mRNA 3'-splice site recognition |
| GO:0010660 | 0.125 | 8.938 | 0 | 1 | 6 | regulation of muscle cell apoptotic process |
| GO:0010656 | 0.125 | 8.938 | 0 | 1 | 6 | negative regulation of muscle cell apoptotic process |
| GO:0010657 | 0.125 | 8.938 | 0 | 1 | 6 | muscle cell apoptotic process |
| GO:0072678 | 0.125 | 8.938 | 0 | 1 | 6 | T cell migration |
| GO:0065005 | 0.125 | 8.938 | 0 | 1 | 6 | protein-lipid complex assembly |
| GO:2000404 | 0.125 | 8.938 | 0 | 1 | 6 | regulation of T cell migration |
| GO:0034377 | 0.125 | 8.938 | 0 | 1 | 6 | plasma lipoprotein particle assembly |
| GO:0003209 | 0.125 | 8.938 | 0 | 1 | 6 | cardiac atrium morphogenesis |
| GO:0055021 | 0.125 | 8.938 | 0 | 1 | 6 | regulation of cardiac muscle tissue growth |
| GO:0055017 | 0.125 | 8.938 | 0 | 1 | 6 | cardiac muscle tissue growth |
| GO:0060444 | 0.125 | 8.938 | 0 | 1 | 6 | branching involved in mammary gland duct morphogenesis |
| GO:0001654 | 0.125 | 1.528 | 7 | 11 | 338 | eye development |
| GO:0007267 | 0.125 | 1.431 | 11 | 15 | 493 | cell-cell signaling |
| GO:0032319 | 0.125 | 3.448 | 1 | 2 | 28 | regulation of Rho GTPase activity |
| GO:0050730 | 0.125 | 3.448 | 1 | 2 | 28 | regulation of peptidyl-tyrosine phosphorylation |
| GO:0048846 | 0.125 | 3.448 | 1 | 2 | 28 | axon extension involved in axon guidance |
| GO:0030879 | 0.125 | 3.448 | 1 | 2 | 28 | mammary gland development |
| GO:0046666 | 0.125 | 3.448 | 1 | 2 | 28 | retinal cell programmed cell death |
| GO:0010827 | 0.125 | 3.448 | 1 | 2 | 28 | regulation of glucose transport |
| GO:0010817 | 0.126 | 1.818 | 3 | 6 | 155 | regulation of hormone levels |
| GO:0009968 | 0.127 | 1.559 | 7 | 10 | 301 | negative regulation of signal transduction |
| GO:0044257 | 0.128 | 1.599 | 6 | 9 | 264 | cellular protein catabolic process |
| GO:0044248 | 0.129 | 1.286 | 24 | 30 | 1107 | cellular catabolic process |
| GO:0006066 | 0.130 | 1.646 | 5 | 8 | 228 | alcohol metabolic process |
| GO:0009152 | 0.130 | 2.495 | 1 | 3 | 57 | purine ribonucleotide biosynthetic process |
| GO:0048806 | 0.130 | 2.495 | 1 | 3 | 57 | genitalia development |
| GO:2000116 | 0.130 | 2.495 | 1 | 3 | 57 | regulation of cysteine-type endopeptidase activity |
| GO:0035295 | 0.132 | 1.638 | 5 | 8 | 229 | tube development |
| GO:0002684 | 0.132 | 2.117 | 2 | 4 | 89 | positive regulation of immune system process |
| GO:0006584 | 0.133 | 3.320 | 1 | 2 | 29 | catecholamine metabolic process |
| GO:0009712 | 0.133 | 3.320 | 1 | 2 | 29 | catechol-containing compound metabolic process |
| GO:0045833 | 0.133 | 3.320 | 1 | 2 | 29 | negative regulation of lipid metabolic process |
| GO:0015758 | 0.133 | 3.320 | 1 | 2 | 29 | glucose transport |
| GO:0015749 | 0.133 | 3.320 | 1 | 2 | 29 | monosaccharide transport |
| GO:0034311 | 0.133 | 3.320 | 1 | 2 | 29 | diol metabolic process |
| GO:0008645 | 0.133 | 3.320 | 1 | 2 | 29 | hexose transport |
| GO:0060446 | 0.133 | 3.320 | 1 | 2 | 29 | branching involved in open tracheal system development |
| GO:1901137 | 0.133 | 1.701 | 4 | 7 | 193 | carbohydrate derivative biosynthetic process |
| GO:0009056 | 0.134 | 1.257 | 30 | 36 | 1364 | catabolic process |
| GO:0006417 | 0.134 | 1.909 | 3 | 5 | 123 | regulation of translation |
| GO:0007349 | 0.135 | 2.450 | 1 | 3 | 58 | cellularization |
| GO:0051098 | 0.135 | 2.450 | 1 | 3 | 58 | regulation of binding |
| GO:0008356 | 0.135 | 2.450 | 1 | 3 | 58 | asymmetric cell division |
| GO:0035556 | 0.135 | 1.329 | 17 | 22 | 781 | intracellular signal transduction |
| GO:0033036 | 0.136 | 1.273 | 25 | 31 | 1155 | macromolecule localization |
| GO:0033057 | 0.136 | 2.092 | 2 | 4 | 90 | multicellular organismal reproductive behavior |
| GO:0071702 | 0.137 | 1.623 | 5 | 8 | 231 | organic substance transport |
| GO:0030900 | 0.140 | 2.406 | 1 | 3 | 59 | forebrain development |
| GO:0006144 | 0.140 | 3.201 | 1 | 2 | 30 | purine nucleobase metabolic process |
| GO:0040010 | 0.140 | 3.201 | 1 | 2 | 30 | positive regulation of growth rate |
| GO:0050708 | 0.140 | 3.201 | 1 | 2 | 30 | regulation of protein secretion |
| GO:0048771 | 0.140 | 3.201 | 1 | 2 | 30 | tissue remodeling |
| GO:0000096 | 0.140 | 3.201 | 1 | 2 | 30 | sulfur amino acid metabolic process |
| GO:0018107 | 0.140 | 3.201 | 1 | 2 | 30 | peptidyl-threonine phosphorylation |
| GO:0008343 | 0.140 | 3.201 | 1 | 2 | 30 | adult feeding behavior |
| GO:0003018 | 0.140 | 3.201 | 1 | 2 | 30 | vascular process in circulatory system |
| GO:0060191 | 0.140 | 3.201 | 1 | 2 | 30 | regulation of lipase activity |
| GO:0009894 | 0.141 | 1.561 | 6 | 9 | 270 | regulation of catabolic process |
| GO:0072521 | 0.142 | 1.416 | 10 | 14 | 464 | purine-containing compound metabolic process |
| GO:0009116 | 0.144 | 1.601 | 5 | 8 | 234 | nucleoside metabolic process |
| GO:0072522 | 0.144 | 2.044 | 2 | 4 | 92 | purine-containing compound biosynthetic process |
| GO:0060788 | 0.144 | 7.447 | 0 | 1 | 7 | ectodermal placode formation |
| GO:0070922 | 0.144 | 7.447 | 0 | 1 | 7 | small RNA loading onto RISC |
| GO:0014033 | 0.144 | 7.447 | 0 | 1 | 7 | neural crest cell differentiation |
| GO:0006110 | 0.144 | 7.447 | 0 | 1 | 7 | regulation of glycolysis |
| GO:0042766 | 0.144 | 7.447 | 0 | 1 | 7 | nucleosome mobilization |
| GO:0050746 | 0.144 | 7.447 | 0 | 1 | 7 | regulation of lipoprotein metabolic process |
| GO:0060966 | 0.144 | 7.447 | 0 | 1 | 7 | regulation of gene silencing by RNA |
| GO:0040006 | 0.144 | 7.447 | 0 | 1 | 7 | protein-based cuticle attachment to epithelium |
| GO:0060914 | 0.144 | 7.447 | 0 | 1 | 7 | heart formation |
| GO:0050892 | 0.144 | 7.447 | 0 | 1 | 7 | intestinal absorption |
| GO:0071025 | 0.144 | 7.447 | 0 | 1 | 7 | RNA surveillance |
| GO:0071027 | 0.144 | 7.447 | 0 | 1 | 7 | nuclear RNA surveillance |
| GO:0006527 | 0.144 | 7.447 | 0 | 1 | 7 | arginine catabolic process |
| GO:0006528 | 0.144 | 7.447 | 0 | 1 | 7 | asparagine metabolic process |
| GO:0006515 | 0.144 | 7.447 | 0 | 1 | 7 | misfolded or incompletely synthesized protein catabolic process |
| GO:0048085 | 0.144 | 7.447 | 0 | 1 | 7 | adult chitin-containing cuticle pigmentation |
| GO:0048072 | 0.144 | 7.447 | 0 | 1 | 7 | compound eye pigmentation |
| GO:0001759 | 0.144 | 7.447 | 0 | 1 | 7 | organ induction |
| GO:0035087 | 0.144 | 7.447 | 0 | 1 | 7 | siRNA loading onto RISC involved in RNA interference |
| GO:0001833 | 0.144 | 7.447 | 0 | 1 | 7 | inner cell mass cell proliferation |
| GO:0006930 | 0.144 | 7.447 | 0 | 1 | 7 | substrate-dependent cell migration, cell extension |
| GO:0061057 | 0.144 | 7.447 | 0 | 1 | 7 | peptidoglycan recognition protein signaling pathway |
| GO:1900115 | 0.144 | 7.447 | 0 | 1 | 7 | extracellular regulation of signal transduction |
| GO:1900116 | 0.144 | 7.447 | 0 | 1 | 7 | extracellular negative regulation of signal transduction |
| GO:0007099 | 0.144 | 7.447 | 0 | 1 | 7 | centriole replication |
| GO:0001954 | 0.144 | 7.447 | 0 | 1 | 7 | positive regulation of cell-matrix adhesion |
| GO:0071696 | 0.144 | 7.447 | 0 | 1 | 7 | ectodermal placode development |
| GO:0071697 | 0.144 | 7.447 | 0 | 1 | 7 | ectodermal placode morphogenesis |
| GO:0043470 | 0.144 | 7.447 | 0 | 1 | 7 | regulation of carbohydrate catabolic process |
| GO:0043471 | 0.144 | 7.447 | 0 | 1 | 7 | regulation of cellular carbohydrate catabolic process |
| GO:0007458 | 0.144 | 7.447 | 0 | 1 | 7 | progression of morphogenetic furrow involved in compound eye morphogenesis |
| GO:0035520 | 0.144 | 7.447 | 0 | 1 | 7 | monoubiquitinated protein deubiquitination |
| GO:0030852 | 0.144 | 7.447 | 0 | 1 | 7 | regulation of granulocyte differentiation |
| GO:0043551 | 0.144 | 7.447 | 0 | 1 | 7 | regulation of phosphatidylinositol 3-kinase activity |
| GO:0043552 | 0.144 | 7.447 | 0 | 1 | 7 | positive regulation of phosphatidylinositol 3-kinase activity |
| GO:0007593 | 0.144 | 7.447 | 0 | 1 | 7 | chitin-based cuticle tanning |
| GO:0043500 | 0.144 | 7.447 | 0 | 1 | 7 | muscle adaptation |
| GO:0046326 | 0.144 | 7.447 | 0 | 1 | 7 | positive regulation of glucose import |
| GO:0030901 | 0.144 | 7.447 | 0 | 1 | 7 | midbrain development |
| GO:0018023 | 0.144 | 7.447 | 0 | 1 | 7 | peptidyl-lysine trimethylation |
| GO:0072499 | 0.144 | 7.447 | 0 | 1 | 7 | photoreceptor cell axon guidance |
| GO:0000059 | 0.144 | 7.447 | 0 | 1 | 7 | protein import into nucleus, docking |
| GO:0090218 | 0.144 | 7.447 | 0 | 1 | 7 | positive regulation of lipid kinase activity |
| GO:0033540 | 0.144 | 7.447 | 0 | 1 | 7 | fatty acid beta-oxidation using acyl-CoA oxidase |
| GO:0002686 | 0.144 | 7.447 | 0 | 1 | 7 | negative regulation of leukocyte migration |
| GO:0044003 | 0.144 | 7.447 | 0 | 1 | 7 | modification by symbiont of host morphology or physiology |
| GO:0046635 | 0.144 | 7.447 | 0 | 1 | 7 | positive regulation of alpha-beta T cell activation |
| GO:0010828 | 0.144 | 7.447 | 0 | 1 | 7 | positive regulation of glucose transport |
| GO:0008365 | 0.144 | 7.447 | 0 | 1 | 7 | adult chitin-based cuticle development |
| GO:0010984 | 0.144 | 7.447 | 0 | 1 | 7 | regulation of lipoprotein particle clearance |
| GO:0060325 | 0.144 | 7.447 | 0 | 1 | 7 | face morphogenesis |
| GO:0055099 | 0.144 | 7.447 | 0 | 1 | 7 | response to high density lipoprotein particle stimulus |
| GO:0060420 | 0.144 | 7.447 | 0 | 1 | 7 | regulation of heart growth |
| GO:0060412 | 0.144 | 7.447 | 0 | 1 | 7 | ventricular septum morphogenesis |
| GO:0060419 | 0.144 | 7.447 | 0 | 1 | 7 | heart growth |
| GO:0007005 | 0.144 | 1.861 | 3 | 5 | 126 | mitochondrion organization |
| GO:0007622 | 0.145 | 2.363 | 1 | 3 | 60 | rhythmic behavior |
| GO:0008344 | 0.145 | 2.363 | 1 | 3 | 60 | adult locomotory behavior |
| GO:0051276 | 0.146 | 1.409 | 10 | 14 | 466 | chromosome organization |
| GO:0006754 | 0.148 | 3.090 | 1 | 2 | 31 | ATP biosynthetic process |
| GO:0032371 | 0.148 | 3.090 | 1 | 2 | 31 | regulation of sterol transport |
| GO:0032374 | 0.148 | 3.090 | 1 | 2 | 31 | regulation of cholesterol transport |
| GO:0030218 | 0.148 | 3.090 | 1 | 2 | 31 | erythrocyte differentiation |
| GO:0051291 | 0.148 | 3.090 | 1 | 2 | 31 | protein heterooligomerization |
| GO:0051249 | 0.148 | 3.090 | 1 | 2 | 31 | regulation of lymphocyte activation |
| GO:0034101 | 0.148 | 3.090 | 1 | 2 | 31 | erythrocyte homeostasis |
| GO:0060485 | 0.148 | 3.090 | 1 | 2 | 31 | mesenchyme development |
| GO:0001501 | 0.148 | 2.021 | 2 | 4 | 93 | skeletal system development |
| GO:0043087 | 0.151 | 1.831 | 3 | 5 | 128 | regulation of GTPase activity |
| GO:0022604 | 0.151 | 1.638 | 4 | 7 | 200 | regulation of cell morphogenesis |
| GO:0060284 | 0.152 | 1.531 | 6 | 9 | 275 | regulation of cell development |
| GO:0030258 | 0.152 | 1.998 | 2 | 4 | 94 | lipid modification |
| GO:0035220 | 0.154 | 1.572 | 5 | 8 | 238 | wing disc development |
| GO:0051960 | 0.154 | 1.572 | 5 | 8 | 238 | regulation of nervous system development |
| GO:0060562 | 0.155 | 1.816 | 3 | 5 | 129 | epithelial tube morphogenesis |
| GO:0033124 | 0.155 | 1.816 | 3 | 5 | 129 | regulation of GTP catabolic process |
| GO:0042770 | 0.155 | 2.282 | 1 | 3 | 62 | signal transduction in response to DNA damage |
| GO:0035152 | 0.155 | 2.282 | 1 | 3 | 62 | regulation of tube architecture, open tracheal system |
| GO:0021510 | 0.156 | 2.987 | 1 | 2 | 32 | spinal cord development |
| GO:0016575 | 0.156 | 2.987 | 1 | 2 | 32 | histone deacetylation |
| GO:0040009 | 0.156 | 2.987 | 1 | 2 | 32 | regulation of growth rate |
| GO:0050810 | 0.156 | 2.987 | 1 | 2 | 32 | regulation of steroid biosynthetic process |
| GO:0071241 | 0.156 | 2.987 | 1 | 2 | 32 | cellular response to inorganic substance |
| GO:0007367 | 0.156 | 2.987 | 1 | 2 | 32 | segment polarity determination |
| GO:0051289 | 0.156 | 2.987 | 1 | 2 | 32 | protein homotetramerization |
| GO:0046425 | 0.156 | 2.987 | 1 | 2 | 32 | regulation of JAK-STAT cascade |
| GO:0046686 | 0.156 | 2.987 | 1 | 2 | 32 | response to cadmium ion |
| GO:0018210 | 0.156 | 2.987 | 1 | 2 | 32 | peptidyl-threonine modification |
| GO:0051051 | 0.156 | 1.976 | 2 | 4 | 95 | negative regulation of transport |
| GO:0045927 | 0.161 | 1.955 | 2 | 4 | 96 | positive regulation of growth |
| GO:0009064 | 0.161 | 2.244 | 1 | 3 | 63 | glutamine family amino acid metabolic process |
| GO:0051649 | 0.161 | 1.273 | 20 | 25 | 925 | establishment of localization in cell |
| GO:0006950 | 0.161 | 1.225 | 32 | 37 | 1433 | response to stress |
| GO:0006140 | 0.162 | 1.680 | 4 | 6 | 167 | regulation of nucleotide metabolic process |
| GO:1900542 | 0.162 | 1.680 | 4 | 6 | 167 | regulation of purine nucleotide metabolic process |
| GO:2000651 | 0.163 | 6.383 | 0 | 1 | 8 | positive regulation of sodium ion transmembrane transporter activity |
| GO:2000649 | 0.163 | 6.383 | 0 | 1 | 8 | regulation of sodium ion transmembrane transporter activity |
| GO:0014003 | 0.163 | 6.383 | 0 | 1 | 8 | oligodendrocyte development |
| GO:0006111 | 0.163 | 6.383 | 0 | 1 | 8 | regulation of gluconeogenesis |
| GO:0006702 | 0.163 | 6.383 | 0 | 1 | 8 | androgen biosynthetic process |
| GO:0032481 | 0.163 | 6.383 | 0 | 1 | 8 | positive regulation of type I interferon production |
| GO:0050829 | 0.163 | 6.383 | 0 | 1 | 8 | defense response to Gram-negative bacterium |
| GO:0048025 | 0.163 | 6.383 | 0 | 1 | 8 | negative regulation of nuclear mRNA splicing, via spliceosome |
| GO:0001776 | 0.163 | 6.383 | 0 | 1 | 8 | leukocyte homeostasis |
| GO:0009113 | 0.163 | 6.383 | 0 | 1 | 8 | purine nucleobase biosynthetic process |
| GO:0006940 | 0.163 | 6.383 | 0 | 1 | 8 | regulation of smooth muscle contraction |
| GO:0045760 | 0.163 | 6.383 | 0 | 1 | 8 | positive regulation of action potential |
| GO:0045742 | 0.163 | 6.383 | 0 | 1 | 8 | positive regulation of epidermal growth factor receptor signaling pathway |
| GO:0048384 | 0.163 | 6.383 | 0 | 1 | 8 | retinoic acid receptor signaling pathway |
| GO:0050996 | 0.163 | 6.383 | 0 | 1 | 8 | positive regulation of lipid catabolic process |
| GO:0045840 | 0.163 | 6.383 | 0 | 1 | 8 | positive regulation of mitosis |
| GO:0035279 | 0.163 | 6.383 | 0 | 1 | 8 | mRNA cleavage involved in gene silencing by miRNA |
| GO:0043388 | 0.163 | 6.383 | 0 | 1 | 8 | positive regulation of DNA binding |
| GO:0007370 | 0.163 | 6.383 | 0 | 1 | 8 | ventral furrow formation |
| GO:0048662 | 0.163 | 6.383 | 0 | 1 | 8 | negative regulation of smooth muscle cell proliferation |
| GO:0030851 | 0.163 | 6.383 | 0 | 1 | 8 | granulocyte differentiation |
| GO:0043654 | 0.163 | 6.383 | 0 | 1 | 8 | recognition of apoptotic cell |
| GO:0007506 | 0.163 | 6.383 | 0 | 1 | 8 | gonadal mesoderm development |
| GO:0046471 | 0.163 | 6.383 | 0 | 1 | 8 | phosphatidylglycerol metabolic process |
| GO:0090342 | 0.163 | 6.383 | 0 | 1 | 8 | regulation of cell aging |
| GO:0030947 | 0.163 | 6.383 | 0 | 1 | 8 | regulation of vascular endothelial growth factor receptor signaling pathway |
| GO:0051785 | 0.163 | 6.383 | 0 | 1 | 8 | positive regulation of nuclear division |
| GO:0051898 | 0.163 | 6.383 | 0 | 1 | 8 | negative regulation of protein kinase B signaling cascade |
| GO:0002688 | 0.163 | 6.383 | 0 | 1 | 8 | regulation of leukocyte chemotaxis |
| GO:0010765 | 0.163 | 6.383 | 0 | 1 | 8 | positive regulation of sodium ion transport |
| GO:0031061 | 0.163 | 6.383 | 0 | 1 | 8 | negative regulation of histone methylation |
| GO:0010872 | 0.163 | 6.383 | 0 | 1 | 8 | regulation of cholesterol esterification |
| GO:1901186 | 0.163 | 6.383 | 0 | 1 | 8 | positive regulation of ERBB signaling pathway |
| GO:0010907 | 0.163 | 6.383 | 0 | 1 | 8 | positive regulation of glucose metabolic process |
| GO:0034374 | 0.163 | 6.383 | 0 | 1 | 8 | low-density lipoprotein particle remodeling |
| GO:0034130 | 0.163 | 6.383 | 0 | 1 | 8 | toll-like receptor 1 signaling pathway |
| GO:0060323 | 0.163 | 6.383 | 0 | 1 | 8 | head morphogenesis |
| GO:0060411 | 0.163 | 6.383 | 0 | 1 | 8 | cardiac septum morphogenesis |
| GO:0070613 | 0.163 | 6.383 | 0 | 1 | 8 | regulation of protein processing |
| GO:0031929 | 0.163 | 2.890 | 1 | 2 | 33 | TOR signaling cascade |
| GO:0006476 | 0.163 | 2.890 | 1 | 2 | 33 | protein deacetylation |
| GO:0001841 | 0.163 | 2.890 | 1 | 2 | 33 | neural tube formation |
| GO:0045862 | 0.163 | 2.890 | 1 | 2 | 33 | positive regulation of proteolysis |
| GO:0043123 | 0.163 | 2.890 | 1 | 2 | 33 | positive regulation of I-kappaB kinase/NF-kappaB cascade |
| GO:0007162 | 0.163 | 2.890 | 1 | 2 | 33 | negative regulation of cell adhesion |
| GO:0002478 | 0.163 | 2.890 | 1 | 2 | 33 | antigen processing and presentation of exogenous peptide antigen |
| GO:0051896 | 0.163 | 2.890 | 1 | 2 | 33 | regulation of protein kinase B signaling cascade |
| GO:0010675 | 0.163 | 2.890 | 1 | 2 | 33 | regulation of cellular carbohydrate metabolic process |
| GO:0010923 | 0.163 | 2.890 | 1 | 2 | 33 | negative regulation of phosphatase activity |
| GO:0010564 | 0.164 | 1.544 | 5 | 8 | 242 | regulation of cell cycle process |
| GO:0048565 | 0.165 | 1.933 | 2 | 4 | 97 | digestive tract development |
| GO:0030833 | 0.166 | 2.207 | 1 | 3 | 64 | regulation of actin filament polymerization |
| GO:0043523 | 0.166 | 2.207 | 1 | 3 | 64 | regulation of neuron apoptotic process |
| GO:0070848 | 0.166 | 2.207 | 1 | 3 | 64 | response to growth factor stimulus |
| GO:0031669 | 0.166 | 2.207 | 1 | 3 | 64 | cellular response to nutrient levels |
| GO:0030163 | 0.167 | 1.460 | 7 | 10 | 320 | protein catabolic process |
| GO:0007015 | 0.168 | 1.659 | 4 | 6 | 169 | actin filament organization |
| GO:0000165 | 0.169 | 1.758 | 3 | 5 | 133 | MAPK cascade |
| GO:0001666 | 0.169 | 1.912 | 2 | 4 | 98 | response to hypoxia |
| GO:0036293 | 0.169 | 1.912 | 2 | 4 | 98 | response to decreased oxygen levels |
| GO:0070482 | 0.169 | 1.912 | 2 | 4 | 98 | response to oxygen levels |
| GO:0006520 | 0.169 | 1.455 | 7 | 10 | 321 | cellular amino acid metabolic process |
| GO:0016032 | 0.171 | 1.579 | 5 | 7 | 207 | viral reproduction |
| GO:0032434 | 0.171 | 2.799 | 1 | 2 | 34 | regulation of proteasomal ubiquitin-dependent protein catabolic process |
| GO:0019884 | 0.171 | 2.799 | 1 | 2 | 34 | antigen processing and presentation of exogenous antigen |
| GO:0009206 | 0.171 | 2.799 | 1 | 2 | 34 | purine ribonucleoside triphosphate biosynthetic process |
| GO:0035601 | 0.171 | 2.799 | 1 | 2 | 34 | protein deacylation |
| GO:0090257 | 0.171 | 2.799 | 1 | 2 | 34 | regulation of muscle system process |
| GO:0051705 | 0.171 | 1.648 | 4 | 6 | 170 | behavioral interaction between organisms |
| GO:0002697 | 0.172 | 2.171 | 1 | 3 | 65 | regulation of immune effector process |
| GO:0006026 | 0.172 | 2.171 | 1 | 3 | 65 | aminoglycan catabolic process |
| GO:0042493 | 0.172 | 1.744 | 3 | 5 | 134 | response to drug |
| GO:0007416 | 0.173 | 1.892 | 2 | 4 | 99 | synapse assembly |
| GO:0034622 | 0.174 | 1.445 | 7 | 10 | 323 | cellular macromolecular complex assembly |
| GO:0035107 | 0.174 | 1.518 | 5 | 8 | 246 | appendage morphogenesis |
| GO:0006936 | 0.177 | 2.136 | 1 | 3 | 66 | muscle contraction |
| GO:0048871 | 0.177 | 2.136 | 1 | 3 | 66 | multicellular organismal homeostasis |
| GO:0032956 | 0.178 | 1.872 | 2 | 4 | 100 | regulation of actin cytoskeleton organization |
| GO:0048747 | 0.178 | 1.872 | 2 | 4 | 100 | muscle fiber development |
| GO:0043408 | 0.178 | 1.872 | 2 | 4 | 100 | regulation of MAPK cascade |
| GO:0051179 | 0.179 | 1.177 | 50 | 56 | 2275 | localization |
| GO:0009145 | 0.179 | 2.714 | 1 | 2 | 35 | purine nucleoside triphosphate biosynthetic process |
| GO:0001819 | 0.179 | 2.714 | 1 | 2 | 35 | positive regulation of cytokine production |
| GO:0017148 | 0.179 | 2.714 | 1 | 2 | 35 | negative regulation of translation |
| GO:0043491 | 0.179 | 2.714 | 1 | 2 | 35 | protein kinase B signaling cascade |
| GO:0043410 | 0.179 | 2.714 | 1 | 2 | 35 | positive regulation of MAPK cascade |
| GO:0043409 | 0.179 | 2.714 | 1 | 2 | 35 | negative regulation of MAPK cascade |
| GO:0033344 | 0.179 | 2.714 | 1 | 2 | 35 | cholesterol efflux |
| GO:0035967 | 0.179 | 2.714 | 1 | 2 | 35 | cellular response to topologically incorrect protein |
| GO:0002920 | 0.179 | 2.714 | 1 | 2 | 35 | regulation of humoral immune response |
| GO:0010623 | 0.179 | 2.714 | 1 | 2 | 35 | developmental programmed cell death |
| GO:0031333 | 0.179 | 2.714 | 1 | 2 | 35 | negative regulation of protein complex assembly |
| GO:0070932 | 0.181 | 5.584 | 0 | 1 | 9 | histone H3 deacetylation |
| GO:0021587 | 0.181 | 5.584 | 0 | 1 | 9 | cerebellum morphogenesis |
| GO:0021575 | 0.181 | 5.584 | 0 | 1 | 9 | hindbrain morphogenesis |
| GO:0021536 | 0.181 | 5.584 | 0 | 1 | 9 | diencephalon development |
| GO:0006471 | 0.181 | 5.584 | 0 | 1 | 9 | protein ADP-ribosylation |
| GO:0006189 | 0.181 | 5.584 | 0 | 1 | 9 | 'de novo' IMP biosynthetic process |
| GO:0006145 | 0.181 | 5.584 | 0 | 1 | 9 | purine nucleobase catabolic process |
| GO:0060603 | 0.181 | 5.584 | 0 | 1 | 9 | mammary gland duct morphogenesis |
| GO:0006777 | 0.181 | 5.584 | 0 | 1 | 9 | Mo-molybdopterin cofactor biosynthetic process |
| GO:0032324 | 0.181 | 5.584 | 0 | 1 | 9 | molybdopterin cofactor biosynthetic process |
| GO:0019720 | 0.181 | 5.584 | 0 | 1 | 9 | Mo-molybdopterin cofactor metabolic process |
| GO:0042987 | 0.181 | 5.584 | 0 | 1 | 9 | amyloid precursor protein catabolic process |
| GO:0001836 | 0.181 | 5.584 | 0 | 1 | 9 | release of cytochrome c from mitochondria |
| GO:0006910 | 0.181 | 5.584 | 0 | 1 | 9 | phagocytosis, recognition |
| GO:0045740 | 0.181 | 5.584 | 0 | 1 | 9 | positive regulation of DNA replication |
| GO:0032648 | 0.181 | 5.584 | 0 | 1 | 9 | regulation of interferon-beta production |
| GO:0035330 | 0.181 | 5.584 | 0 | 1 | 9 | regulation of hippo signaling cascade |
| GO:0030422 | 0.181 | 5.584 | 0 | 1 | 9 | production of siRNA involved in RNA interference |
| GO:0030212 | 0.181 | 5.584 | 0 | 1 | 9 | hyaluronan metabolic process |
| GO:0002088 | 0.181 | 5.584 | 0 | 1 | 9 | lens development in camera-type eye |
| GO:0007406 | 0.181 | 5.584 | 0 | 1 | 9 | negative regulation of neuroblast proliferation |
| GO:0030850 | 0.181 | 5.584 | 0 | 1 | 9 | prostate gland development |
| GO:0035666 | 0.181 | 5.584 | 0 | 1 | 9 | TRIF-dependent toll-like receptor signaling pathway |
| GO:0007257 | 0.181 | 5.584 | 0 | 1 | 9 | activation of JUN kinase activity |
| GO:0043550 | 0.181 | 5.584 | 0 | 1 | 9 | regulation of lipid kinase activity |
| GO:0043545 | 0.181 | 5.584 | 0 | 1 | 9 | molybdopterin cofactor metabolic process |
| GO:0051189 | 0.181 | 5.584 | 0 | 1 | 9 | prosthetic group metabolic process |
| GO:0051149 | 0.181 | 5.584 | 0 | 1 | 9 | positive regulation of muscle cell differentiation |
| GO:0043628 | 0.181 | 5.584 | 0 | 1 | 9 | ncRNA 3'-end processing |
| GO:0033119 | 0.181 | 5.584 | 0 | 1 | 9 | negative regulation of RNA splicing |
| GO:0002763 | 0.181 | 5.584 | 0 | 1 | 9 | positive regulation of myeloid leukocyte differentiation |
| GO:0051571 | 0.181 | 5.584 | 0 | 1 | 9 | positive regulation of histone H3-K4 methylation |
| GO:0000042 | 0.181 | 5.584 | 0 | 1 | 9 | protein targeting to Golgi |
| GO:0090287 | 0.181 | 5.584 | 0 | 1 | 9 | regulation of cellular response to growth factor stimulus |
| GO:0046664 | 0.181 | 5.584 | 0 | 1 | 9 | dorsal closure, amnioserosa morphology change |
| GO:0046622 | 0.181 | 5.584 | 0 | 1 | 9 | positive regulation of organ growth |
| GO:0046634 | 0.181 | 5.584 | 0 | 1 | 9 | regulation of alpha-beta T cell activation |
| GO:0010884 | 0.181 | 5.584 | 0 | 1 | 9 | positive regulation of lipid storage |
| GO:0010839 | 0.181 | 5.584 | 0 | 1 | 9 | negative regulation of keratinocyte proliferation |
| GO:0010634 | 0.181 | 5.584 | 0 | 1 | 9 | positive regulation of epithelial cell migration |
| GO:0031365 | 0.181 | 5.584 | 0 | 1 | 9 | N-terminal protein amino acid modification |
| GO:0008210 | 0.181 | 5.584 | 0 | 1 | 9 | estrogen metabolic process |
| GO:2000178 | 0.181 | 5.584 | 0 | 1 | 9 | negative regulation of neural precursor cell proliferation |
| GO:0034134 | 0.181 | 5.584 | 0 | 1 | 9 | toll-like receptor 2 signaling pathway |
| GO:0060324 | 0.181 | 5.584 | 0 | 1 | 9 | face development |
| GO:0060443 | 0.181 | 5.584 | 0 | 1 | 9 | mammary gland morphogenesis |
| GO:0060439 | 0.181 | 5.584 | 0 | 1 | 9 | trachea morphogenesis |
| GO:0007584 | 0.182 | 1.853 | 2 | 4 | 101 | response to nutrient |
| GO:0016051 | 0.182 | 1.853 | 2 | 4 | 101 | carbohydrate biosynthetic process |
| GO:0052548 | 0.182 | 2.103 | 1 | 3 | 67 | regulation of endopeptidase activity |
| GO:0045165 | 0.183 | 1.457 | 6 | 9 | 288 | cell fate commitment |
| GO:0034504 | 0.184 | 1.704 | 3 | 5 | 137 | protein localization to nucleus |
| GO:0009066 | 0.187 | 2.634 | 1 | 2 | 36 | aspartate family amino acid metabolic process |
| GO:0032368 | 0.187 | 2.634 | 1 | 2 | 36 | regulation of lipid transport |
| GO:0048015 | 0.187 | 2.634 | 1 | 2 | 36 | phosphatidylinositol-mediated signaling |
| GO:0048017 | 0.187 | 2.634 | 1 | 2 | 36 | inositol lipid-mediated signaling |
| GO:0043542 | 0.187 | 2.634 | 1 | 2 | 36 | endothelial cell migration |
| GO:0000187 | 0.187 | 2.634 | 1 | 2 | 36 | activation of MAPK activity |
| GO:0007050 | 0.188 | 1.599 | 4 | 6 | 175 | cell cycle arrest |
| GO:0055001 | 0.188 | 1.599 | 4 | 6 | 175 | muscle cell development |
| GO:0042742 | 0.188 | 2.070 | 1 | 3 | 68 | defense response to bacterium |
| GO:0009636 | 0.188 | 2.070 | 1 | 3 | 68 | response to toxin |
| GO:0006928 | 0.191 | 1.296 | 13 | 17 | 613 | cellular component movement |
| GO:0006952 | 0.193 | 1.408 | 7 | 10 | 331 | defense response |
| GO:0052547 | 0.194 | 2.039 | 2 | 3 | 69 | regulation of peptidase activity |
| GO:1901214 | 0.194 | 2.039 | 2 | 3 | 69 | regulation of neuron death |
| GO:0031668 | 0.194 | 2.039 | 2 | 3 | 69 | cellular response to extracellular stimulus |
| GO:0042542 | 0.195 | 2.558 | 1 | 2 | 37 | response to hydrogen peroxide |
| GO:2001056 | 0.195 | 2.558 | 1 | 2 | 37 | positive regulation of cysteine-type endopeptidase activity |
| GO:0045444 | 0.195 | 2.558 | 1 | 2 | 37 | fat cell differentiation |
| GO:0043280 | 0.195 | 2.558 | 1 | 2 | 37 | positive regulation of cysteine-type endopeptidase activity involved in apoptotic process |
| GO:0007143 | 0.195 | 2.558 | 1 | 2 | 37 | female meiosis |
| GO:0051298 | 0.195 | 2.558 | 1 | 2 | 37 | centrosome duplication |
| GO:0010950 | 0.195 | 2.558 | 1 | 2 | 37 | positive regulation of endopeptidase activity |
| GO:0005977 | 0.195 | 2.558 | 1 | 2 | 37 | glycogen metabolic process |
| GO:0007169 | 0.196 | 1.467 | 6 | 8 | 254 | transmembrane receptor protein tyrosine kinase signaling pathway |
| GO:0043065 | 0.198 | 1.570 | 4 | 6 | 178 | positive regulation of apoptotic process |
| GO:0009260 | 0.199 | 2.008 | 2 | 3 | 70 | ribonucleotide biosynthetic process |
| GO:0001933 | 0.199 | 2.008 | 2 | 3 | 70 | negative regulation of protein phosphorylation |
| GO:0072594 | 0.199 | 2.008 | 2 | 3 | 70 | establishment of protein localization to organelle |
| GO:0021795 | 0.199 | 4.963 | 0 | 1 | 10 | cerebral cortex cell migration |
| GO:0014068 | 0.199 | 4.963 | 0 | 1 | 10 | positive regulation of phosphatidylinositol 3-kinase cascade |
| GO:0034959 | 0.199 | 4.963 | 0 | 1 | 10 | endothelin maturation |
| GO:0021988 | 0.199 | 4.963 | 0 | 1 | 10 | olfactory lobe development |
| GO:0032314 | 0.199 | 4.963 | 0 | 1 | 10 | regulation of Rac GTPase activity |
| GO:0006534 | 0.199 | 4.963 | 0 | 1 | 10 | cysteine metabolic process |
| GO:0009303 | 0.199 | 4.963 | 0 | 1 | 10 | rRNA transcription |
| GO:0048067 | 0.199 | 4.963 | 0 | 1 | 10 | cuticle pigmentation |
| GO:0071347 | 0.199 | 4.963 | 0 | 1 | 10 | cellular response to interleukin-1 |
| GO:0071300 | 0.199 | 4.963 | 0 | 1 | 10 | cellular response to retinoic acid |
| GO:0032862 | 0.199 | 4.963 | 0 | 1 | 10 | activation of Rho GTPase activity |
| GO:0006956 | 0.199 | 4.963 | 0 | 1 | 10 | complement activation |
| GO:0071299 | 0.199 | 4.963 | 0 | 1 | 10 | cellular response to vitamin A |
| GO:0032608 | 0.199 | 4.963 | 0 | 1 | 10 | interferon-beta production |
| GO:0048199 | 0.199 | 4.963 | 0 | 1 | 10 | vesicle targeting, to, from or within Golgi |
| GO:0045542 | 0.199 | 4.963 | 0 | 1 | 10 | positive regulation of cholesterol biosynthetic process |
| GO:0001953 | 0.199 | 4.963 | 0 | 1 | 10 | negative regulation of cell-matrix adhesion |
| GO:0035225 | 0.199 | 4.963 | 0 | 1 | 10 | determination of genital disc primordium |
| GO:0007428 | 0.199 | 4.963 | 0 | 1 | 10 | primary branching, open tracheal system |
| GO:0007445 | 0.199 | 4.963 | 0 | 1 | 10 | determination of imaginal disc primordium |
| GO:0051004 | 0.199 | 4.963 | 0 | 1 | 10 | regulation of lipoprotein lipase activity |
| GO:0007184 | 0.199 | 4.963 | 0 | 1 | 10 | SMAD protein import into nucleus |
| GO:0007128 | 0.199 | 4.963 | 0 | 1 | 10 | meiotic prophase I |
| GO:0002444 | 0.199 | 4.963 | 0 | 1 | 10 | myeloid leukocyte mediated immunity |
| GO:0051123 | 0.199 | 4.963 | 0 | 1 | 10 | RNA polymerase II transcriptional preinitiation complex assembly |
| GO:0002755 | 0.199 | 4.963 | 0 | 1 | 10 | MyD88-dependent toll-like receptor signaling pathway |
| GO:0046113 | 0.199 | 4.963 | 0 | 1 | 10 | nucleobase catabolic process |
| GO:0090195 | 0.199 | 4.963 | 0 | 1 | 10 | chemokine secretion |
| GO:0090196 | 0.199 | 4.963 | 0 | 1 | 10 | regulation of chemokine secretion |
| GO:0090197 | 0.199 | 4.963 | 0 | 1 | 10 | positive regulation of chemokine secretion |
| GO:0033700 | 0.199 | 4.963 | 0 | 1 | 10 | phospholipid efflux |
| GO:0000301 | 0.199 | 4.963 | 0 | 1 | 10 | retrograde transport, vesicle recycling within Golgi |
| GO:0051961 | 0.199 | 4.963 | 0 | 1 | 10 | negative regulation of nervous system development |
| GO:0051964 | 0.199 | 4.963 | 0 | 1 | 10 | negative regulation of synapse assembly |
| GO:0010888 | 0.199 | 4.963 | 0 | 1 | 10 | negative regulation of lipid storage |
| GO:0010813 | 0.199 | 4.963 | 0 | 1 | 10 | neuropeptide catabolic process |
| GO:0010814 | 0.199 | 4.963 | 0 | 1 | 10 | substance P catabolic process |
| GO:0010816 | 0.199 | 4.963 | 0 | 1 | 10 | calcitonin catabolic process |
| GO:0008363 | 0.199 | 4.963 | 0 | 1 | 10 | larval chitin-based cuticle development |
| GO:0015936 | 0.199 | 4.963 | 0 | 1 | 10 | coenzyme A metabolic process |
| GO:0034436 | 0.199 | 4.963 | 0 | 1 | 10 | glycoprotein transport |
| GO:0034138 | 0.199 | 4.963 | 0 | 1 | 10 | toll-like receptor 3 signaling pathway |
| GO:0003281 | 0.199 | 4.963 | 0 | 1 | 10 | ventricular septum development |
| GO:0055094 | 0.199 | 4.963 | 0 | 1 | 10 | response to lipoprotein particle stimulus |
| GO:0060416 | 0.199 | 4.963 | 0 | 1 | 10 | response to growth hormone stimulus |
| GO:0042632 | 0.203 | 2.487 | 1 | 2 | 38 | cholesterol homeostasis |
| GO:0048002 | 0.203 | 2.487 | 1 | 2 | 38 | antigen processing and presentation of peptide antigen |
| GO:0045732 | 0.203 | 2.487 | 1 | 2 | 38 | positive regulation of protein catabolic process |
| GO:0007365 | 0.203 | 2.487 | 1 | 2 | 38 | periodic partitioning |
| GO:0043270 | 0.203 | 2.487 | 1 | 2 | 38 | positive regulation of ion transport |
| GO:0051225 | 0.203 | 2.487 | 1 | 2 | 38 | spindle assembly |
| GO:0010952 | 0.203 | 2.487 | 1 | 2 | 38 | positive regulation of peptidase activity |
| GO:0006073 | 0.203 | 2.487 | 1 | 2 | 38 | cellular glucan metabolic process |
| GO:0040011 | 0.204 | 1.242 | 18 | 22 | 829 | locomotion |
| GO:0007009 | 0.205 | 1.978 | 2 | 3 | 71 | plasma membrane organization |
| GO:0030832 | 0.205 | 1.978 | 2 | 3 | 71 | regulation of actin filament length |
| GO:0051346 | 0.205 | 1.978 | 2 | 3 | 71 | negative regulation of hydrolase activity |
| GO:0008064 | 0.205 | 1.978 | 2 | 3 | 71 | regulation of actin polymerization or depolymerization |
| GO:0008049 | 0.205 | 1.978 | 2 | 3 | 71 | male courtship behavior |
| GO:0046128 | 0.207 | 1.487 | 5 | 7 | 219 | purine ribonucleoside metabolic process |
| GO:0019752 | 0.209 | 1.273 | 14 | 17 | 623 | carboxylic acid metabolic process |
| GO:0043436 | 0.209 | 1.273 | 14 | 17 | 623 | oxoacid metabolic process |
| GO:0042278 | 0.210 | 1.480 | 5 | 7 | 220 | purine nucleoside metabolic process |
| GO:0030811 | 0.211 | 1.617 | 3 | 5 | 144 | regulation of nucleotide catabolic process |
| GO:0033121 | 0.211 | 1.617 | 3 | 5 | 144 | regulation of purine nucleotide catabolic process |
| GO:0051640 | 0.211 | 1.617 | 3 | 5 | 144 | organelle localization |
| GO:0050795 | 0.211 | 1.949 | 2 | 3 | 72 | regulation of behavior |
| GO:0032271 | 0.211 | 1.949 | 2 | 3 | 72 | regulation of protein polymerization |
| GO:0035150 | 0.211 | 1.949 | 2 | 3 | 72 | regulation of tube size |
| GO:0006813 | 0.211 | 2.419 | 1 | 2 | 39 | potassium ion transport |
| GO:0009201 | 0.211 | 2.419 | 1 | 2 | 39 | ribonucleoside triphosphate biosynthetic process |
| GO:0030326 | 0.211 | 2.419 | 1 | 2 | 39 | embryonic limb morphogenesis |
| GO:0035113 | 0.211 | 2.419 | 1 | 2 | 39 | embryonic appendage morphogenesis |
| GO:0007259 | 0.211 | 2.419 | 1 | 2 | 39 | JAK-STAT cascade |
| GO:0071901 | 0.211 | 2.419 | 1 | 2 | 39 | negative regulation of protein serine/threonine kinase activity |
| GO:0090002 | 0.211 | 2.419 | 1 | 2 | 39 | establishment of protein localization to plasma membrane |
| GO:0043627 | 0.211 | 2.419 | 1 | 2 | 39 | response to estrogen stimulus |
| GO:0090150 | 0.211 | 2.419 | 1 | 2 | 39 | establishment of protein localization to membrane |
| GO:0002694 | 0.211 | 2.419 | 1 | 2 | 39 | regulation of leukocyte activation |
| GO:0044042 | 0.211 | 2.419 | 1 | 2 | 39 | glucan metabolic process |
| GO:0042592 | 0.214 | 1.299 | 11 | 14 | 502 | homeostatic process |
| GO:0071496 | 0.216 | 1.921 | 2 | 3 | 73 | cellular response to external stimulus |
| GO:0051402 | 0.216 | 1.921 | 2 | 3 | 73 | neuron apoptotic process |
| GO:0000272 | 0.216 | 1.921 | 2 | 3 | 73 | polysaccharide catabolic process |
| GO:0060070 | 0.216 | 1.921 | 2 | 3 | 73 | canonical Wnt receptor signaling pathway |
| GO:0009119 | 0.217 | 1.466 | 5 | 7 | 222 | ribonucleoside metabolic process |
| GO:0021766 | 0.217 | 4.466 | 0 | 1 | 11 | hippocampus development |
| GO:0021872 | 0.217 | 4.466 | 0 | 1 | 11 | forebrain generation of neurons |
| GO:0006337 | 0.217 | 4.466 | 0 | 1 | 11 | nucleosome disassembly |
| GO:0006309 | 0.217 | 4.466 | 0 | 1 | 11 | apoptotic DNA fragmentation |
| GO:0032091 | 0.217 | 4.466 | 0 | 1 | 11 | negative regulation of protein binding |
| GO:0006188 | 0.217 | 4.466 | 0 | 1 | 11 | IMP biosynthetic process |
| GO:0040034 | 0.217 | 4.466 | 0 | 1 | 11 | regulation of development, heterochronic |
| GO:0006801 | 0.217 | 4.466 | 0 | 1 | 11 | superoxide metabolic process |
| GO:0006654 | 0.217 | 4.466 | 0 | 1 | 11 | phosphatidic acid biosynthetic process |
| GO:0032722 | 0.217 | 4.466 | 0 | 1 | 11 | positive regulation of chemokine production |
| GO:0014896 | 0.217 | 4.466 | 0 | 1 | 11 | muscle hypertrophy |
| GO:0014897 | 0.217 | 4.466 | 0 | 1 | 11 | striated muscle hypertrophy |
| GO:0001832 | 0.217 | 4.466 | 0 | 1 | 11 | blastocyst growth |
| GO:0050999 | 0.217 | 4.466 | 0 | 1 | 11 | regulation of nitric-oxide synthase activity |
| GO:0061077 | 0.217 | 4.466 | 0 | 1 | 11 | chaperone-mediated protein folding |
| GO:0022408 | 0.217 | 4.466 | 0 | 1 | 11 | negative regulation of cell-cell adhesion |
| GO:0045598 | 0.217 | 4.466 | 0 | 1 | 11 | regulation of fat cell differentiation |
| GO:0001921 | 0.217 | 4.466 | 0 | 1 | 11 | positive regulation of receptor recycling |
| GO:0048793 | 0.217 | 4.466 | 0 | 1 | 11 | pronephros development |
| GO:0032986 | 0.217 | 4.466 | 0 | 1 | 11 | protein-DNA complex disassembly |
| GO:0061180 | 0.217 | 4.466 | 0 | 1 | 11 | mammary gland epithelium development |
| GO:0043146 | 0.217 | 4.466 | 0 | 1 | 11 | spindle stabilization |
| GO:0046040 | 0.217 | 4.466 | 0 | 1 | 11 | IMP metabolic process |
| GO:0051324 | 0.217 | 4.466 | 0 | 1 | 11 | prophase |
| GO:0051457 | 0.217 | 4.466 | 0 | 1 | 11 | maintenance of protein location in nucleus |
| GO:0046320 | 0.217 | 4.466 | 0 | 1 | 11 | regulation of fatty acid oxidation |
| GO:0046473 | 0.217 | 4.466 | 0 | 1 | 11 | phosphatidic acid metabolic process |
| GO:0002756 | 0.217 | 4.466 | 0 | 1 | 11 | MyD88-independent toll-like receptor signaling pathway |
| GO:0090205 | 0.217 | 4.466 | 0 | 1 | 11 | positive regulation of cholesterol metabolic process |
| GO:0010893 | 0.217 | 4.466 | 0 | 1 | 11 | positive regulation of steroid biosynthetic process |
| GO:0072600 | 0.217 | 4.466 | 0 | 1 | 11 | establishment of protein localization to Golgi |
| GO:0031498 | 0.217 | 4.466 | 0 | 1 | 11 | chromatin disassembly |
| GO:0010962 | 0.217 | 4.466 | 0 | 1 | 11 | regulation of glucan biosynthetic process |
| GO:0031103 | 0.217 | 4.466 | 0 | 1 | 11 | axon regeneration |
| GO:0003300 | 0.217 | 4.466 | 0 | 1 | 11 | cardiac muscle hypertrophy |
| GO:0005979 | 0.217 | 4.466 | 0 | 1 | 11 | regulation of glycogen biosynthetic process |
| GO:0070897 | 0.217 | 4.466 | 0 | 1 | 11 | DNA-dependent transcriptional preinitiation complex assembly |
| GO:0003156 | 0.217 | 4.466 | 0 | 1 | 11 | regulation of organ formation |
| GO:0060438 | 0.217 | 4.466 | 0 | 1 | 11 | trachea development |
| GO:0007476 | 0.219 | 1.516 | 4 | 6 | 184 | imaginal disc-derived wing morphogenesis |
| GO:0019218 | 0.219 | 2.355 | 1 | 2 | 40 | regulation of steroid metabolic process |
| GO:0035215 | 0.219 | 2.355 | 1 | 2 | 40 | genital disc development |
| GO:0061136 | 0.219 | 2.355 | 1 | 2 | 40 | regulation of proteasomal protein catabolic process |
| GO:0007608 | 0.219 | 2.355 | 1 | 2 | 40 | sensory perception of smell |
| GO:0002683 | 0.219 | 2.355 | 1 | 2 | 40 | negative regulation of immune system process |
| GO:0006082 | 0.220 | 1.260 | 14 | 17 | 629 | organic acid metabolic process |
| GO:0042326 | 0.222 | 1.894 | 2 | 3 | 74 | negative regulation of phosphorylation |
| GO:0030041 | 0.222 | 1.894 | 2 | 3 | 74 | actin filament polymerization |
| GO:0006486 | 0.223 | 1.693 | 2 | 4 | 110 | protein glycosylation |
| GO:0043413 | 0.223 | 1.693 | 2 | 4 | 110 | macromolecule glycosylation |
| GO:0043434 | 0.223 | 1.693 | 2 | 4 | 110 | response to peptide hormone stimulus |
| GO:0070085 | 0.223 | 1.693 | 2 | 4 | 110 | glycosylation |
| GO:0008654 | 0.223 | 1.693 | 2 | 4 | 110 | phospholipid biosynthetic process |
| GO:0006644 | 0.226 | 1.499 | 4 | 6 | 186 | phospholipid metabolic process |
| GO:0007472 | 0.226 | 1.499 | 4 | 6 | 186 | wing disc morphogenesis |
| GO:0007424 | 0.226 | 1.499 | 4 | 6 | 186 | open tracheal system development |
| GO:0019882 | 0.228 | 2.295 | 1 | 2 | 41 | antigen processing and presentation |
| GO:0007062 | 0.228 | 2.295 | 1 | 2 | 41 | sister chromatid cohesion |
| GO:0030177 | 0.228 | 2.295 | 1 | 2 | 41 | positive regulation of Wnt receptor signaling pathway |
| GO:0043122 | 0.228 | 2.295 | 1 | 2 | 41 | regulation of I-kappaB kinase/NF-kappaB cascade |
| GO:0051302 | 0.228 | 2.295 | 1 | 2 | 41 | regulation of cell division |
| GO:0046148 | 0.228 | 2.295 | 1 | 2 | 41 | pigment biosynthetic process |
| GO:0010171 | 0.228 | 2.295 | 1 | 2 | 41 | body morphogenesis |
| GO:0055092 | 0.228 | 2.295 | 1 | 2 | 41 | sterol homeostasis |
| GO:0046907 | 0.228 | 1.251 | 14 | 17 | 633 | intracellular transport |
| GO:0060179 | 0.228 | 1.867 | 2 | 3 | 75 | male mating behavior |
| GO:0001667 | 0.228 | 1.867 | 2 | 3 | 75 | ameboidal cell migration |
| GO:0032507 | 0.228 | 1.867 | 2 | 3 | 75 | maintenance of protein location in cell |
| GO:0006886 | 0.232 | 1.320 | 9 | 11 | 387 | intracellular protein transport |
| GO:0006909 | 0.232 | 1.662 | 2 | 4 | 112 | phagocytosis |
| GO:0002164 | 0.232 | 1.662 | 2 | 4 | 112 | larval development |
| GO:0044057 | 0.233 | 1.482 | 4 | 6 | 188 | regulation of system process |
| GO:0032318 | 0.234 | 1.841 | 2 | 3 | 76 | regulation of Ras GTPase activity |
| GO:0009950 | 0.234 | 1.841 | 2 | 3 | 76 | dorsal/ventral axis specification |
| GO:0051260 | 0.234 | 1.841 | 2 | 3 | 76 | protein homooligomerization |
| GO:0042381 | 0.234 | 4.060 | 0 | 1 | 12 | hemolymph coagulation |
| GO:0021532 | 0.234 | 4.060 | 0 | 1 | 12 | neural tube patterning |
| GO:0042129 | 0.234 | 4.060 | 0 | 1 | 12 | regulation of T cell proliferation |
| GO:0032008 | 0.234 | 4.060 | 0 | 1 | 12 | positive regulation of TOR signaling cascade |
| GO:0050715 | 0.234 | 4.060 | 0 | 1 | 12 | positive regulation of cytokine secretion |
| GO:0006555 | 0.234 | 4.060 | 0 | 1 | 12 | methionine metabolic process |
| GO:0006614 | 0.234 | 4.060 | 0 | 1 | 12 | SRP-dependent cotranslational protein targeting to membrane |
| GO:0045199 | 0.234 | 4.060 | 0 | 1 | 12 | maintenance of epithelial cell apical/basal polarity |
| GO:0050686 | 0.234 | 4.060 | 0 | 1 | 12 | negative regulation of mRNA processing |
| GO:0045292 | 0.234 | 4.060 | 0 | 1 | 12 | nuclear mRNA cis splicing, via spliceosome |
| GO:0032874 | 0.234 | 4.060 | 0 | 1 | 12 | positive regulation of stress-activated MAPK cascade |
| GO:0045792 | 0.234 | 4.060 | 0 | 1 | 12 | negative regulation of cell size |
| GO:1900274 | 0.234 | 4.060 | 0 | 1 | 12 | regulation of phospholipase C activity |
| GO:0045639 | 0.234 | 4.060 | 0 | 1 | 12 | positive regulation of myeloid cell differentiation |
| GO:0035146 | 0.234 | 4.060 | 0 | 1 | 12 | tube fusion |
| GO:0035147 | 0.234 | 4.060 | 0 | 1 | 12 | branch fusion, open tracheal system |
| GO:0001947 | 0.234 | 4.060 | 0 | 1 | 12 | heart looping |
| GO:0045940 | 0.234 | 4.060 | 0 | 1 | 12 | positive regulation of steroid metabolic process |
| GO:0043154 | 0.234 | 4.060 | 0 | 1 | 12 | negative regulation of cysteine-type endopeptidase activity involved in apoptotic process |
| GO:0007402 | 0.234 | 4.060 | 0 | 1 | 12 | ganglion mother cell fate determination |
| GO:0048661 | 0.234 | 4.060 | 0 | 1 | 12 | positive regulation of smooth muscle cell proliferation |
| GO:0022600 | 0.234 | 4.060 | 0 | 1 | 12 | digestive system process |
| GO:0007112 | 0.234 | 4.060 | 0 | 1 | 12 | male meiosis cytokinesis |
| GO:0046323 | 0.234 | 4.060 | 0 | 1 | 12 | glucose import |
| GO:0046324 | 0.234 | 4.060 | 0 | 1 | 12 | regulation of glucose import |
| GO:0046330 | 0.234 | 4.060 | 0 | 1 | 12 | positive regulation of JNK cascade |
| GO:0007628 | 0.234 | 4.060 | 0 | 1 | 12 | adult walking behavior |
| GO:0046112 | 0.234 | 4.060 | 0 | 1 | 12 | nucleobase biosynthetic process |
| GO:0033574 | 0.234 | 4.060 | 0 | 1 | 12 | response to testosterone stimulus |
| GO:0033619 | 0.234 | 4.060 | 0 | 1 | 12 | membrane protein proteolysis |
| GO:0010799 | 0.234 | 4.060 | 0 | 1 | 12 | regulation of peptidyl-threonine phosphorylation |
| GO:0010745 | 0.234 | 4.060 | 0 | 1 | 12 | negative regulation of macrophage derived foam cell differentiation |
| GO:0010863 | 0.234 | 4.060 | 0 | 1 | 12 | positive regulation of phospholipase C activity |
| GO:0010837 | 0.234 | 4.060 | 0 | 1 | 12 | regulation of keratinocyte proliferation |
| GO:0010800 | 0.234 | 4.060 | 0 | 1 | 12 | positive regulation of peptidyl-threonine phosphorylation |
| GO:0010518 | 0.234 | 4.060 | 0 | 1 | 12 | positive regulation of phospholipase activity |
| GO:0010676 | 0.234 | 4.060 | 0 | 1 | 12 | positive regulation of cellular carbohydrate metabolic process |
| GO:0034067 | 0.234 | 4.060 | 0 | 1 | 12 | protein localization to Golgi apparatus |
| GO:0070304 | 0.234 | 4.060 | 0 | 1 | 12 | positive regulation of stress-activated protein kinase signaling cascade |
| GO:0008209 | 0.234 | 4.060 | 0 | 1 | 12 | androgen metabolic process |
| GO:0034383 | 0.234 | 4.060 | 0 | 1 | 12 | low-density lipoprotein particle clearance |
| GO:0070873 | 0.234 | 4.060 | 0 | 1 | 12 | regulation of glycogen metabolic process |
| GO:0003143 | 0.234 | 4.060 | 0 | 1 | 12 | embryonic heart tube morphogenesis |
| GO:0006090 | 0.234 | 4.060 | 0 | 1 | 12 | pyruvate metabolic process |
| GO:0055013 | 0.234 | 4.060 | 0 | 1 | 12 | cardiac muscle cell development |
| GO:0019226 | 0.234 | 1.317 | 9 | 11 | 388 | transmission of nerve impulse |
| GO:0048066 | 0.236 | 2.237 | 1 | 2 | 42 | developmental pigmentation |
| GO:0009112 | 0.236 | 2.237 | 1 | 2 | 42 | nucleobase metabolic process |
| GO:0006096 | 0.236 | 2.237 | 1 | 2 | 42 | glycolysis |
| GO:0044255 | 0.238 | 1.268 | 11 | 14 | 513 | cellular lipid metabolic process |
| GO:0007059 | 0.239 | 1.538 | 3 | 5 | 151 | chromosome segregation |
| GO:0006091 | 0.239 | 1.418 | 5 | 7 | 229 | generation of precursor metabolites and energy |
| GO:0045936 | 0.240 | 1.816 | 2 | 3 | 77 | negative regulation of phosphate metabolic process |
| GO:0046474 | 0.240 | 1.816 | 2 | 3 | 77 | glycerophospholipid biosynthetic process |
| GO:0051651 | 0.240 | 1.816 | 2 | 3 | 77 | maintenance of location in cell |
| GO:0010563 | 0.240 | 1.816 | 2 | 3 | 77 | negative regulation of phosphorus metabolic process |
| GO:0009611 | 0.240 | 1.352 | 7 | 9 | 309 | response to wounding |
| GO:0055002 | 0.243 | 1.527 | 3 | 5 | 152 | striated muscle cell development |
| GO:0006405 | 0.244 | 2.182 | 1 | 2 | 43 | RNA export from nucleus |
| GO:0006767 | 0.244 | 2.182 | 1 | 2 | 43 | water-soluble vitamin metabolic process |
| GO:0019438 | 0.244 | 2.182 | 1 | 2 | 43 | aromatic compound biosynthetic process |
| GO:0032409 | 0.244 | 2.182 | 1 | 2 | 43 | regulation of transporter activity |
| GO:0009142 | 0.244 | 2.182 | 1 | 2 | 43 | nucleoside triphosphate biosynthetic process |
| GO:0001838 | 0.244 | 2.182 | 1 | 2 | 43 | embryonic epithelial tube formation |
| GO:0072175 | 0.244 | 2.182 | 1 | 2 | 43 | epithelial tube formation |
| GO:0008286 | 0.244 | 2.182 | 1 | 2 | 43 | insulin receptor signaling pathway |
| GO:0008643 | 0.244 | 2.182 | 1 | 2 | 43 | carbohydrate transport |
| GO:0048285 | 0.246 | 1.342 | 7 | 9 | 311 | organelle fission |
| GO:0042445 | 0.247 | 1.616 | 3 | 4 | 115 | hormone metabolic process |
| GO:0006650 | 0.247 | 1.616 | 3 | 4 | 115 | glycerophospholipid metabolic process |
| GO:0016052 | 0.247 | 1.517 | 3 | 5 | 153 | carbohydrate catabolic process |
| GO:0043068 | 0.248 | 1.449 | 4 | 6 | 192 | positive regulation of programmed cell death |
| GO:0051050 | 0.248 | 1.449 | 4 | 6 | 192 | positive regulation of transport |
| GO:0016044 | 0.249 | 1.338 | 7 | 9 | 312 | cellular membrane organization |
| GO:0023061 | 0.251 | 1.506 | 3 | 5 | 154 | signal release |
| GO:0003001 | 0.251 | 1.506 | 3 | 5 | 154 | generation of a signal involved in cell-cell signaling |
| GO:0060177 | 0.251 | 3.721 | 0 | 1 | 13 | regulation of angiotensin metabolic process |
| GO:0016180 | 0.251 | 3.721 | 0 | 1 | 13 | snRNA processing |
| GO:0021761 | 0.251 | 3.721 | 0 | 1 | 13 | limbic system development |
| GO:0042417 | 0.251 | 3.721 | 0 | 1 | 13 | dopamine metabolic process |
| GO:0042447 | 0.251 | 3.721 | 0 | 1 | 13 | hormone catabolic process |
| GO:0060612 | 0.251 | 3.721 | 0 | 1 | 13 | adipose tissue development |
| GO:0042775 | 0.251 | 3.721 | 0 | 1 | 13 | mitochondrial ATP synthesis coupled electron transport |
| GO:0045453 | 0.251 | 3.721 | 0 | 1 | 13 | bone resorption |
| GO:0006963 | 0.251 | 3.721 | 0 | 1 | 13 | positive regulation of antibacterial peptide biosynthetic process |
| GO:0045778 | 0.251 | 3.721 | 0 | 1 | 13 | positive regulation of ossification |
| GO:0045736 | 0.251 | 3.721 | 0 | 1 | 13 | negative regulation of cyclin-dependent protein kinase activity |
| GO:0045540 | 0.251 | 3.721 | 0 | 1 | 13 | regulation of cholesterol biosynthetic process |
| GO:0001991 | 0.251 | 3.721 | 0 | 1 | 13 | regulation of systemic arterial blood pressure by circulatory renin-angiotensin |
| GO:0001938 | 0.251 | 3.721 | 0 | 1 | 13 | positive regulation of endothelial cell proliferation |
| GO:0030262 | 0.251 | 3.721 | 0 | 1 | 13 | apoptotic nuclear change |
| GO:0002002 | 0.251 | 3.721 | 0 | 1 | 13 | regulation of angiotensin levels in blood |
| GO:0002780 | 0.251 | 3.721 | 0 | 1 | 13 | antibacterial peptide biosynthetic process |
| GO:0002786 | 0.251 | 3.721 | 0 | 1 | 13 | regulation of antibacterial peptide production |
| GO:0002778 | 0.251 | 3.721 | 0 | 1 | 13 | antibacterial peptide production |
| GO:0018022 | 0.251 | 3.721 | 0 | 1 | 13 | peptidyl-lysine methylation |
| GO:0090175 | 0.251 | 3.721 | 0 | 1 | 13 | regulation of establishment of planar polarity |
| GO:0010469 | 0.251 | 3.721 | 0 | 1 | 13 | regulation of receptor activity |
| GO:0002808 | 0.251 | 3.721 | 0 | 1 | 13 | regulation of antibacterial peptide biosynthetic process |
| GO:0090224 | 0.251 | 3.721 | 0 | 1 | 13 | regulation of spindle organization |
| GO:0002698 | 0.251 | 3.721 | 0 | 1 | 13 | negative regulation of immune effector process |
| GO:0046605 | 0.251 | 3.721 | 0 | 1 | 13 | regulation of centrosome cycle |
| GO:0010815 | 0.251 | 3.721 | 0 | 1 | 13 | bradykinin catabolic process |
| GO:0000212 | 0.251 | 3.721 | 0 | 1 | 13 | meiotic spindle organization |
| GO:0031102 | 0.251 | 3.721 | 0 | 1 | 13 | neuron projection regeneration |
| GO:0060071 | 0.251 | 3.721 | 0 | 1 | 13 | Wnt receptor signaling pathway, planar cell polarity pathway |
| GO:0018991 | 0.251 | 3.721 | 0 | 1 | 13 | oviposition |
| GO:0055091 | 0.251 | 3.721 | 0 | 1 | 13 | phospholipid homeostasis |
| GO:0055006 | 0.251 | 3.721 | 0 | 1 | 13 | cardiac cell development |
| GO:0018130 | 0.251 | 1.441 | 4 | 6 | 193 | heterocycle biosynthetic process |
| GO:0010942 | 0.251 | 1.441 | 4 | 6 | 193 | positive regulation of cell death |
| GO:0061024 | 0.251 | 1.333 | 7 | 9 | 313 | membrane organization |
| GO:0070997 | 0.252 | 1.768 | 2 | 3 | 79 | neuron death |
| GO:0032868 | 0.252 | 1.768 | 2 | 3 | 79 | response to insulin stimulus |
| GO:0000077 | 0.252 | 1.768 | 2 | 3 | 79 | DNA damage checkpoint |
| GO:0030073 | 0.252 | 2.130 | 1 | 2 | 44 | insulin secretion |
| GO:0007030 | 0.252 | 2.130 | 1 | 2 | 44 | Golgi organization |
| GO:0032970 | 0.256 | 1.587 | 3 | 4 | 117 | regulation of actin filament-based process |
| GO:0051604 | 0.256 | 1.587 | 3 | 4 | 117 | protein maturation |
| GO:0016072 | 0.256 | 1.587 | 3 | 4 | 117 | rRNA metabolic process |
| GO:0035023 | 0.257 | 1.745 | 2 | 3 | 80 | regulation of Rho protein signal transduction |
| GO:0006892 | 0.260 | 2.080 | 1 | 2 | 45 | post-Golgi vesicle-mediated transport |
| GO:0051607 | 0.260 | 2.080 | 1 | 2 | 45 | defense response to virus |
| GO:0030155 | 0.261 | 1.573 | 3 | 4 | 118 | regulation of cell adhesion |
| GO:0019953 | 0.264 | 1.219 | 13 | 16 | 609 | sexual reproduction |
| GO:0035120 | 0.266 | 1.411 | 4 | 6 | 197 | post-embryonic appendage morphogenesis |
| GO:0007611 | 0.266 | 1.559 | 3 | 4 | 119 | learning or memory |
| GO:0006403 | 0.268 | 1.466 | 3 | 5 | 158 | RNA localization |
| GO:0017038 | 0.268 | 1.466 | 3 | 5 | 158 | protein import |
| GO:0030097 | 0.268 | 1.466 | 3 | 5 | 158 | hemopoiesis |
| GO:0014066 | 0.268 | 3.434 | 0 | 1 | 14 | regulation of phosphatidylinositol 3-kinase cascade |
| GO:0042773 | 0.268 | 3.434 | 0 | 1 | 14 | ATP synthesis coupled electron transport |
| GO:0045047 | 0.268 | 3.434 | 0 | 1 | 14 | protein targeting to ER |
| GO:0006760 | 0.268 | 3.434 | 0 | 1 | 14 | folic acid-containing compound metabolic process |
| GO:0006890 | 0.268 | 3.434 | 0 | 1 | 14 | retrograde vesicle-mediated transport, Golgi to ER |
| GO:0050777 | 0.268 | 3.434 | 0 | 1 | 14 | negative regulation of immune response |
| GO:0050707 | 0.268 | 3.434 | 0 | 1 | 14 | regulation of cytokine secretion |
| GO:0060912 | 0.268 | 3.434 | 0 | 1 | 14 | cardiac cell fate specification |
| GO:0042685 | 0.268 | 3.434 | 0 | 1 | 14 | cardioblast cell fate specification |
| GO:0042686 | 0.268 | 3.434 | 0 | 1 | 14 | regulation of cardioblast cell fate specification |
| GO:0048069 | 0.268 | 3.434 | 0 | 1 | 14 | eye pigmentation |
| GO:0032856 | 0.268 | 3.434 | 0 | 1 | 14 | activation of Ras GTPase activity |
| GO:0006929 | 0.268 | 3.434 | 0 | 1 | 14 | substrate-dependent cell migration |
| GO:0045861 | 0.268 | 3.434 | 0 | 1 | 14 | negative regulation of proteolysis |
| GO:0071295 | 0.268 | 3.434 | 0 | 1 | 14 | cellular response to vitamin |
| GO:0071709 | 0.268 | 3.434 | 0 | 1 | 14 | membrane assembly |
| GO:0045666 | 0.268 | 3.434 | 0 | 1 | 14 | positive regulation of neuron differentiation |
| GO:0035153 | 0.268 | 3.434 | 0 | 1 | 14 | epithelial cell type specification, open tracheal system |
| GO:0061371 | 0.268 | 3.434 | 0 | 1 | 14 | determination of heart left/right asymmetry |
| GO:0051352 | 0.268 | 3.434 | 0 | 1 | 14 | negative regulation of ligase activity |
| GO:0051492 | 0.268 | 3.434 | 0 | 1 | 14 | regulation of stress fiber assembly |
| GO:0051444 | 0.268 | 3.434 | 0 | 1 | 14 | negative regulation of ubiquitin-protein ligase activity |
| GO:0051436 | 0.268 | 3.434 | 0 | 1 | 14 | negative regulation of ubiquitin-protein ligase activity involved in mitotic cell cycle |
| GO:0051290 | 0.268 | 3.434 | 0 | 1 | 14 | protein heterotetramerization |
| GO:0033280 | 0.268 | 3.434 | 0 | 1 | 14 | response to vitamin D |
| GO:0051890 | 0.268 | 3.434 | 0 | 1 | 14 | regulation of cardioblast differentiation |
| GO:0072376 | 0.268 | 3.434 | 0 | 1 | 14 | protein activation cascade |
| GO:0000097 | 0.268 | 3.434 | 0 | 1 | 14 | sulfur amino acid biosynthetic process |
| GO:0031057 | 0.268 | 3.434 | 0 | 1 | 14 | negative regulation of histone modification |
| GO:0046631 | 0.268 | 3.434 | 0 | 1 | 14 | alpha-beta T cell activation |
| GO:0010878 | 0.268 | 3.434 | 0 | 1 | 14 | cholesterol storage |
| GO:0072595 | 0.268 | 3.434 | 0 | 1 | 14 | maintenance of protein localization in organelle |
| GO:0072599 | 0.268 | 3.434 | 0 | 1 | 14 | establishment of protein localization to endoplasmic reticulum |
| GO:0010640 | 0.268 | 3.434 | 0 | 1 | 14 | regulation of platelet-derived growth factor receptor signaling pathway |
| GO:0010642 | 0.268 | 3.434 | 0 | 1 | 14 | negative regulation of platelet-derived growth factor receptor signaling pathway |
| GO:2000043 | 0.268 | 3.434 | 0 | 1 | 14 | regulation of cardiac cell fate specification |
| GO:0003081 | 0.268 | 3.434 | 0 | 1 | 14 | regulation of systemic arterial blood pressure by renin-angiotensin |
| GO:2000377 | 0.268 | 3.434 | 0 | 1 | 14 | regulation of reactive oxygen species metabolic process |
| GO:0060037 | 0.268 | 3.434 | 0 | 1 | 14 | pharyngeal system development |
| GO:0008542 | 0.268 | 3.434 | 0 | 1 | 14 | visual learning |
| GO:0034142 | 0.268 | 3.434 | 0 | 1 | 14 | toll-like receptor 4 signaling pathway |
| GO:0042098 | 0.268 | 3.434 | 0 | 1 | 14 | T cell proliferation |
| GO:0060193 | 0.268 | 3.434 | 0 | 1 | 14 | positive regulation of lipase activity |
| GO:0042110 | 0.269 | 2.033 | 1 | 2 | 46 | T cell activation |
| GO:0045088 | 0.269 | 2.033 | 1 | 2 | 46 | regulation of innate immune response |
| GO:0009267 | 0.269 | 2.033 | 1 | 2 | 46 | cellular response to starvation |
| GO:0061448 | 0.269 | 2.033 | 1 | 2 | 46 | connective tissue development |
| GO:0043112 | 0.269 | 2.033 | 1 | 2 | 46 | receptor metabolic process |
| GO:0010741 | 0.269 | 2.033 | 1 | 2 | 46 | negative regulation of intracellular protein kinase cascade |
| GO:0006575 | 0.269 | 1.700 | 2 | 3 | 82 | cellular modified amino acid metabolic process |
| GO:0035637 | 0.270 | 1.267 | 9 | 11 | 402 | multicellular organismal signaling |
| GO:0042180 | 0.272 | 1.204 | 14 | 17 | 655 | cellular ketone metabolic process |
| GO:0006694 | 0.275 | 1.679 | 2 | 3 | 83 | steroid biosynthetic process |
| GO:0009987 | 0.276 | 1.189 | 158 | 161 | 7174 | cellular process |
| GO:0006606 | 0.276 | 1.532 | 3 | 4 | 121 | protein import into nucleus |
| GO:0097285 | 0.276 | 1.532 | 3 | 4 | 121 | cell-type specific apoptotic process |
| GO:0051170 | 0.276 | 1.532 | 3 | 4 | 121 | nuclear import |
| GO:0060541 | 0.277 | 1.350 | 5 | 7 | 240 | respiratory system development |
| GO:0035114 | 0.277 | 1.388 | 4 | 6 | 200 | imaginal disc-derived appendage morphogenesis |
| GO:0044281 | 0.280 | 1.134 | 33 | 36 | 1482 | small molecule metabolic process |
| GO:0050890 | 0.281 | 1.519 | 3 | 4 | 122 | cognition |
| GO:0042060 | 0.281 | 1.381 | 4 | 6 | 201 | wound healing |
| GO:0016226 | 0.284 | 3.188 | 0 | 1 | 15 | iron-sulfur cluster assembly |
| GO:0070936 | 0.284 | 3.188 | 0 | 1 | 15 | protein K48-linked ubiquitination |
| GO:0006750 | 0.284 | 3.188 | 0 | 1 | 15 | glutathione biosynthetic process |
| GO:0009070 | 0.284 | 3.188 | 0 | 1 | 15 | serine family amino acid biosynthetic process |
| GO:0032321 | 0.284 | 3.188 | 0 | 1 | 15 | positive regulation of Rho GTPase activity |
| GO:0040020 | 0.284 | 3.188 | 0 | 1 | 15 | regulation of meiosis |
| GO:0060968 | 0.284 | 3.188 | 0 | 1 | 15 | regulation of gene silencing |
| GO:0006568 | 0.284 | 3.188 | 0 | 1 | 15 | tryptophan metabolic process |
| GO:0019731 | 0.284 | 3.188 | 0 | 1 | 15 | antibacterial humoral response |
| GO:0006613 | 0.284 | 3.188 | 0 | 1 | 15 | cotranslational protein targeting to membrane |
| GO:0006967 | 0.284 | 3.188 | 0 | 1 | 15 | positive regulation of antifungal peptide biosynthetic process |
| GO:0032846 | 0.284 | 3.188 | 0 | 1 | 15 | positive regulation of homeostatic process |
| GO:1900150 | 0.284 | 3.188 | 0 | 1 | 15 | regulation of defense response to fungus |
| GO:0061331 | 0.284 | 3.188 | 0 | 1 | 15 | epithelial cell proliferation involved in Malpighian tubule morphogenesis |
| GO:0043149 | 0.284 | 3.188 | 0 | 1 | 15 | stress fiber assembly |
| GO:0002028 | 0.284 | 3.188 | 0 | 1 | 15 | regulation of sodium ion transport |
| GO:0048676 | 0.284 | 3.188 | 0 | 1 | 15 | axon extension involved in development |
| GO:0035567 | 0.284 | 3.188 | 0 | 1 | 15 | non-canonical Wnt receptor signaling pathway |
| GO:0043268 | 0.284 | 3.188 | 0 | 1 | 15 | positive regulation of potassium ion transport |
| GO:0051445 | 0.284 | 3.188 | 0 | 1 | 15 | regulation of meiotic cell cycle |
| GO:0043574 | 0.284 | 3.188 | 0 | 1 | 15 | peroxisomal transport |
| GO:0043507 | 0.284 | 3.188 | 0 | 1 | 15 | positive regulation of JUN kinase activity |
| GO:0002781 | 0.284 | 3.188 | 0 | 1 | 15 | antifungal peptide production |
| GO:0002783 | 0.284 | 3.188 | 0 | 1 | 15 | antifungal peptide biosynthetic process |
| GO:0002788 | 0.284 | 3.188 | 0 | 1 | 15 | regulation of antifungal peptide production |
| GO:0002810 | 0.284 | 3.188 | 0 | 1 | 15 | regulation of antifungal peptide biosynthetic process |
| GO:0051591 | 0.284 | 3.188 | 0 | 1 | 15 | response to cAMP |
| GO:0000018 | 0.284 | 3.188 | 0 | 1 | 15 | regulation of DNA recombination |
| GO:0010812 | 0.284 | 3.188 | 0 | 1 | 15 | negative regulation of cell-substrate adhesion |
| GO:0031163 | 0.284 | 3.188 | 0 | 1 | 15 | metallo-sulfur cluster assembly |
| GO:2000177 | 0.284 | 3.188 | 0 | 1 | 15 | regulation of neural precursor cell proliferation |
| GO:0046578 | 0.285 | 1.428 | 4 | 5 | 162 | regulation of Ras protein signal transduction |
| GO:0043043 | 0.285 | 1.944 | 1 | 2 | 48 | peptide biosynthetic process |
| GO:0034728 | 0.285 | 1.944 | 1 | 2 | 48 | nucleosome organization |
| GO:0008154 | 0.287 | 1.638 | 2 | 3 | 85 | actin polymerization or depolymerization |
| GO:0048737 | 0.289 | 1.367 | 4 | 6 | 203 | imaginal disc-derived appendage development |
| GO:0051704 | 0.289 | 1.202 | 13 | 15 | 578 | multi-organism process |
| GO:0030036 | 0.290 | 1.276 | 7 | 9 | 326 | actin cytoskeleton organization |
| GO:0009411 | 0.293 | 1.902 | 1 | 2 | 49 | response to UV |
| GO:0006986 | 0.293 | 1.902 | 1 | 2 | 49 | response to unfolded protein |
| GO:0030308 | 0.293 | 1.902 | 1 | 2 | 49 | negative regulation of cell growth |
| GO:0072593 | 0.293 | 1.902 | 1 | 2 | 49 | reactive oxygen species metabolic process |
| GO:0045168 | 0.293 | 1.618 | 2 | 3 | 86 | cell-cell signaling involved in cell fate commitment |
| GO:0007052 | 0.293 | 1.618 | 2 | 3 | 86 | mitotic spindle organization |
| GO:0007422 | 0.293 | 1.618 | 2 | 3 | 86 | peripheral nervous system development |
| GO:0016192 | 0.295 | 1.182 | 15 | 17 | 666 | vesicle-mediated transport |
| GO:0050657 | 0.296 | 1.481 | 3 | 4 | 125 | nucleic acid transport |
| GO:0050658 | 0.296 | 1.481 | 3 | 4 | 125 | RNA transport |
| GO:0030031 | 0.296 | 1.481 | 3 | 4 | 125 | cell projection assembly |
| GO:0051236 | 0.296 | 1.481 | 3 | 4 | 125 | establishment of RNA localization |
| GO:0007268 | 0.296 | 1.268 | 7 | 9 | 328 | synaptic transmission |
| GO:0006605 | 0.297 | 1.315 | 5 | 7 | 246 | protein targeting |
| GO:0006766 | 0.299 | 1.598 | 2 | 3 | 87 | vitamin metabolic process |
| GO:0006518 | 0.299 | 1.598 | 2 | 3 | 87 | peptide metabolic process |
| GO:0051052 | 0.299 | 1.598 | 2 | 3 | 87 | regulation of DNA metabolic process |
| GO:0000086 | 0.299 | 1.598 | 2 | 3 | 87 | G2/M transition of mitotic cell cycle |
| GO:0031570 | 0.299 | 1.598 | 2 | 3 | 87 | DNA integrity checkpoint |
| GO:0042475 | 0.300 | 2.976 | 0 | 1 | 16 | odontogenesis of dentin-containing tooth |
| GO:0032011 | 0.300 | 2.976 | 0 | 1 | 16 | ARF protein signal transduction |
| GO:0006206 | 0.300 | 2.976 | 0 | 1 | 16 | pyrimidine nucleobase metabolic process |
| GO:0050771 | 0.300 | 2.976 | 0 | 1 | 16 | negative regulation of axonogenesis |
| GO:0050848 | 0.300 | 2.976 | 0 | 1 | 16 | regulation of calcium-mediated signaling |
| GO:0006692 | 0.300 | 2.976 | 0 | 1 | 16 | prostanoid metabolic process |
| GO:0006693 | 0.300 | 2.976 | 0 | 1 | 16 | prostaglandin metabolic process |
| GO:0050688 | 0.300 | 2.976 | 0 | 1 | 16 | regulation of defense response to virus |
| GO:0050670 | 0.300 | 2.976 | 0 | 1 | 16 | regulation of lymphocyte proliferation |
| GO:1900424 | 0.300 | 2.976 | 0 | 1 | 16 | regulation of defense response to bacterium |
| GO:0001578 | 0.300 | 2.976 | 0 | 1 | 16 | microtubule bundle formation |
| GO:0032506 | 0.300 | 2.976 | 0 | 1 | 16 | cytokinetic process |
| GO:0001676 | 0.300 | 2.976 | 0 | 1 | 16 | long-chain fatty acid metabolic process |
| GO:0001990 | 0.300 | 2.976 | 0 | 1 | 16 | regulation of systemic arterial blood pressure by hormone |
| GO:0035176 | 0.300 | 2.976 | 0 | 1 | 16 | social behavior |
| GO:0032944 | 0.300 | 2.976 | 0 | 1 | 16 | regulation of mononuclear cell proliferation |
| GO:0048800 | 0.300 | 2.976 | 0 | 1 | 16 | antennal morphogenesis |
| GO:0007412 | 0.300 | 2.976 | 0 | 1 | 16 | axon target recognition |
| GO:0030720 | 0.300 | 2.976 | 0 | 1 | 16 | oocyte localization involved in germarium-derived egg chamber formation |
| GO:0043616 | 0.300 | 2.976 | 0 | 1 | 16 | keratinocyte proliferation |
| GO:0046427 | 0.300 | 2.976 | 0 | 1 | 16 | positive regulation of JAK-STAT cascade |
| GO:0033206 | 0.300 | 2.976 | 0 | 1 | 16 | cytokinesis after meiosis |
| GO:0043928 | 0.300 | 2.976 | 0 | 1 | 16 | exonucleolytic nuclear-transcribed mRNA catabolic process involved in deadenylation-dependent decay |
| GO:0090181 | 0.300 | 2.976 | 0 | 1 | 16 | regulation of cholesterol metabolic process |
| GO:0051569 | 0.300 | 2.976 | 0 | 1 | 16 | regulation of histone H3-K4 methylation |
| GO:0000132 | 0.300 | 2.976 | 0 | 1 | 16 | establishment of mitotic spindle orientation |
| GO:0000291 | 0.300 | 2.976 | 0 | 1 | 16 | nuclear-transcribed mRNA catabolic process, exonucleolytic |
| GO:0008637 | 0.300 | 2.976 | 0 | 1 | 16 | apoptotic mitochondrial changes |
| GO:0070555 | 0.300 | 2.976 | 0 | 1 | 16 | response to interleukin-1 |
| GO:0007601 | 0.301 | 1.468 | 3 | 4 | 126 | visual perception |
| GO:0034097 | 0.301 | 1.468 | 3 | 4 | 126 | response to cytokine stimulus |
| GO:0006112 | 0.301 | 1.862 | 1 | 2 | 50 | energy reserve metabolic process |
| GO:0032320 | 0.301 | 1.862 | 1 | 2 | 50 | positive regulation of Ras GTPase activity |
| GO:0045787 | 0.301 | 1.862 | 1 | 2 | 50 | positive regulation of cell cycle |
| GO:0072659 | 0.301 | 1.862 | 1 | 2 | 50 | protein localization to plasma membrane |
| GO:0006629 | 0.302 | 1.170 | 16 | 18 | 712 | lipid metabolic process |
| GO:0007276 | 0.305 | 1.186 | 13 | 15 | 585 | gamete generation |
| GO:0050953 | 0.306 | 1.456 | 3 | 4 | 127 | sensory perception of light stimulus |
| GO:0048813 | 0.306 | 1.456 | 3 | 4 | 127 | dendrite morphogenesis |
| GO:0006184 | 0.306 | 1.383 | 4 | 5 | 167 | GTP catabolic process |
| GO:0050877 | 0.306 | 1.167 | 16 | 18 | 714 | neurological system process |
| GO:0045785 | 0.309 | 1.824 | 1 | 2 | 51 | positive regulation of cell adhesion |
| GO:0030072 | 0.309 | 1.824 | 1 | 2 | 51 | peptide hormone secretion |
| GO:0007249 | 0.309 | 1.824 | 1 | 2 | 51 | I-kappaB kinase/NF-kappaB cascade |
| GO:1901069 | 0.310 | 1.374 | 4 | 5 | 168 | guanosine-containing compound catabolic process |
| GO:0009416 | 0.311 | 1.444 | 3 | 4 | 128 | response to light stimulus |
| GO:0009994 | 0.311 | 1.444 | 3 | 4 | 128 | oocyte differentiation |
| GO:0045185 | 0.312 | 1.561 | 2 | 3 | 89 | maintenance of protein location |
| GO:0009150 | 0.314 | 1.228 | 8 | 10 | 376 | purine ribonucleotide metabolic process |
| GO:0006400 | 0.315 | 2.789 | 0 | 1 | 17 | tRNA modification |
| GO:0032069 | 0.315 | 2.789 | 0 | 1 | 17 | regulation of nuclease activity |
| GO:0032075 | 0.315 | 2.789 | 0 | 1 | 17 | positive regulation of nuclease activity |
| GO:0006108 | 0.315 | 2.789 | 0 | 1 | 17 | malate metabolic process |
| GO:0050852 | 0.315 | 2.789 | 0 | 1 | 17 | T cell receptor signaling pathway |
| GO:0050830 | 0.315 | 2.789 | 0 | 1 | 17 | defense response to Gram-positive bacterium |
| GO:0032197 | 0.315 | 2.789 | 0 | 1 | 17 | transposition, RNA-mediated |
| GO:0019732 | 0.315 | 2.789 | 0 | 1 | 17 | antifungal humoral response |
| GO:0032231 | 0.315 | 2.789 | 0 | 1 | 17 | regulation of actin filament bundle assembly |
| GO:0048008 | 0.315 | 2.789 | 0 | 1 | 17 | platelet-derived growth factor receptor signaling pathway |
| GO:0045186 | 0.315 | 2.789 | 0 | 1 | 17 | zonula adherens assembly |
| GO:0006987 | 0.315 | 2.789 | 0 | 1 | 17 | activation of signaling protein activity involved in unfolded protein response |
| GO:0001919 | 0.315 | 2.789 | 0 | 1 | 17 | regulation of receptor recycling |
| GO:0030866 | 0.315 | 2.789 | 0 | 1 | 17 | cortical actin cytoskeleton organization |
| GO:0002761 | 0.315 | 2.789 | 0 | 1 | 17 | regulation of myeloid leukocyte differentiation |
| GO:0051865 | 0.315 | 2.789 | 0 | 1 | 17 | protein autoubiquitination |
| GO:0000038 | 0.315 | 2.789 | 0 | 1 | 17 | very long-chain fatty acid metabolic process |
| GO:0010875 | 0.315 | 2.789 | 0 | 1 | 17 | positive regulation of cholesterol efflux |
| GO:0000737 | 0.315 | 2.789 | 0 | 1 | 17 | DNA catabolic process, endonucleolytic |
| GO:0003044 | 0.315 | 2.789 | 0 | 1 | 17 | regulation of systemic arterial blood pressure mediated by a chemical signal |
| GO:2000117 | 0.315 | 2.789 | 0 | 1 | 17 | negative regulation of cysteine-type endopeptidase activity |
| GO:0003231 | 0.315 | 2.789 | 0 | 1 | 17 | cardiac ventricle development |
| GO:0070663 | 0.315 | 2.789 | 0 | 1 | 17 | regulation of leukocyte proliferation |
| GO:0048477 | 0.317 | 1.224 | 8 | 10 | 377 | oogenesis |
| GO:0010769 | 0.318 | 1.542 | 2 | 3 | 90 | regulation of cell morphogenesis involved in differentiation |
| GO:0060828 | 0.318 | 1.787 | 1 | 2 | 52 | regulation of canonical Wnt receptor signaling pathway |
| GO:0021915 | 0.318 | 1.787 | 1 | 2 | 52 | neural tube development |
| GO:0019221 | 0.318 | 1.787 | 1 | 2 | 52 | cytokine-mediated signaling pathway |
| GO:0002831 | 0.318 | 1.787 | 1 | 2 | 52 | regulation of response to biotic stimulus |
| GO:0044271 | 0.319 | 1.281 | 6 | 7 | 252 | cellular nitrogen compound biosynthetic process |
| GO:0051234 | 0.323 | 1.100 | 40 | 43 | 1820 | establishment of localization |
| GO:0007389 | 0.323 | 1.193 | 10 | 12 | 464 | pattern specification process |
| GO:0046039 | 0.323 | 1.349 | 4 | 5 | 171 | GTP metabolic process |
| GO:0007623 | 0.324 | 1.525 | 2 | 3 | 91 | circadian rhythm |
| GO:0001655 | 0.326 | 1.410 | 3 | 4 | 131 | urogenital system development |
| GO:0048511 | 0.326 | 1.410 | 3 | 4 | 131 | rhythmic process |
| GO:0000075 | 0.326 | 1.410 | 3 | 4 | 131 | cell cycle checkpoint |
| GO:0009306 | 0.326 | 1.752 | 1 | 2 | 53 | protein secretion |
| GO:0007098 | 0.326 | 1.752 | 1 | 2 | 53 | centrosome cycle |
| GO:0007606 | 0.326 | 1.752 | 1 | 2 | 53 | sensory perception of chemical stimulus |
| GO:0090132 | 0.326 | 1.752 | 1 | 2 | 53 | epithelium migration |
| GO:0010631 | 0.326 | 1.752 | 1 | 2 | 53 | epithelial cell migration |
| GO:0006810 | 0.326 | 1.099 | 39 | 42 | 1778 | transport |
| GO:0046483 | 0.326 | 1.160 | 14 | 16 | 637 | heterocycle metabolic process |
| GO:0048534 | 0.328 | 1.341 | 4 | 5 | 172 | hemopoietic or lymphoid organ development |
| GO:0048569 | 0.329 | 1.265 | 6 | 7 | 255 | post-embryonic organ development |
| GO:0030162 | 0.330 | 1.507 | 2 | 3 | 92 | regulation of proteolysis |
| GO:0007269 | 0.330 | 1.507 | 2 | 3 | 92 | neurotransmitter secretion |
| GO:0006163 | 0.330 | 1.196 | 9 | 11 | 424 | purine nucleotide metabolic process |
| GO:0014065 | 0.330 | 2.625 | 0 | 1 | 18 | phosphatidylinositol 3-kinase cascade |
| GO:0042551 | 0.330 | 2.625 | 0 | 1 | 18 | neuron maturation |
| GO:0006516 | 0.330 | 2.625 | 0 | 1 | 18 | glycoprotein catabolic process |
| GO:0006690 | 0.330 | 2.625 | 0 | 1 | 18 | icosanoid metabolic process |
| GO:0050679 | 0.330 | 2.625 | 0 | 1 | 18 | positive regulation of epithelial cell proliferation |
| GO:0035090 | 0.330 | 2.625 | 0 | 1 | 18 | maintenance of apical/basal cell polarity |
| GO:0006939 | 0.330 | 2.625 | 0 | 1 | 18 | smooth muscle contraction |
| GO:0045739 | 0.330 | 2.625 | 0 | 1 | 18 | positive regulation of DNA repair |
| GO:0050994 | 0.330 | 2.625 | 0 | 1 | 18 | regulation of lipid catabolic process |
| GO:0009593 | 0.330 | 2.625 | 0 | 1 | 18 | detection of chemical stimulus |
| GO:0048709 | 0.330 | 2.625 | 0 | 1 | 18 | oligodendrocyte differentiation |
| GO:0007475 | 0.330 | 2.625 | 0 | 1 | 18 | apposition of dorsal and ventral imaginal disc-derived wing surfaces |
| GO:0043171 | 0.330 | 2.625 | 0 | 1 | 18 | peptide catabolic process |
| GO:0030865 | 0.330 | 2.625 | 0 | 1 | 18 | cortical cytoskeleton organization |
| GO:0010332 | 0.330 | 2.625 | 0 | 1 | 18 | response to gamma radiation |
| GO:0010894 | 0.330 | 2.625 | 0 | 1 | 18 | negative regulation of steroid biosynthetic process |
| GO:0016073 | 0.330 | 2.625 | 0 | 1 | 18 | snRNA metabolic process |
| GO:0060349 | 0.330 | 2.625 | 0 | 1 | 18 | bone morphogenesis |
| GO:0016318 | 0.330 | 2.625 | 0 | 1 | 18 | ommatidial rotation |
| GO:0031670 | 0.330 | 2.625 | 0 | 1 | 18 | cellular response to nutrient |
| GO:0042454 | 0.332 | 1.333 | 4 | 5 | 173 | ribonucleoside catabolic process |
| GO:0006152 | 0.332 | 1.333 | 4 | 5 | 173 | purine nucleoside catabolic process |
| GO:0046130 | 0.332 | 1.333 | 4 | 5 | 173 | purine ribonucleoside catabolic process |
| GO:0009205 | 0.333 | 1.220 | 7 | 9 | 340 | purine ribonucleoside triphosphate metabolic process |
| GO:0030029 | 0.333 | 1.220 | 7 | 9 | 340 | actin filament-based process |
| GO:0034660 | 0.333 | 1.260 | 6 | 7 | 256 | ncRNA metabolic process |
| GO:0071824 | 0.334 | 1.718 | 1 | 2 | 54 | protein-DNA complex subunit organization |
| GO:0051124 | 0.334 | 1.718 | 1 | 2 | 54 | synaptic growth at neuromuscular junction |
| GO:0006007 | 0.334 | 1.718 | 1 | 2 | 54 | glucose catabolic process |
| GO:0060173 | 0.336 | 1.490 | 2 | 3 | 93 | limb development |
| GO:0000278 | 0.336 | 1.163 | 12 | 14 | 555 | mitotic cell cycle |
| GO:0009144 | 0.339 | 1.212 | 8 | 9 | 342 | purine nucleoside triphosphate metabolic process |
| GO:0001701 | 0.341 | 1.377 | 3 | 4 | 134 | in utero embryonic development |
| GO:0051329 | 0.341 | 1.317 | 4 | 5 | 175 | interphase of mitotic cell cycle |
| GO:0045321 | 0.342 | 1.474 | 2 | 3 | 94 | leukocyte activation |
| GO:0048705 | 0.342 | 1.686 | 1 | 2 | 55 | skeletal system morphogenesis |
| GO:0002253 | 0.342 | 1.686 | 1 | 2 | 55 | activation of immune response |
| GO:0002790 | 0.342 | 1.686 | 1 | 2 | 55 | peptide secretion |
| GO:0090130 | 0.342 | 1.686 | 1 | 2 | 55 | tissue migration |
| GO:0046620 | 0.342 | 1.686 | 1 | 2 | 55 | regulation of organ growth |
| GO:0055088 | 0.342 | 1.686 | 1 | 2 | 55 | lipid homeostasis |
| GO:0007281 | 0.343 | 1.274 | 5 | 6 | 217 | germ cell development |
| GO:0021549 | 0.345 | 2.479 | 0 | 1 | 19 | cerebellum development |
| GO:0016578 | 0.345 | 2.479 | 0 | 1 | 19 | histone deubiquitination |
| GO:0006171 | 0.345 | 2.479 | 0 | 1 | 19 | cAMP biosynthetic process |
| GO:0042744 | 0.345 | 2.479 | 0 | 1 | 19 | hydrogen peroxide catabolic process |
| GO:0009065 | 0.345 | 2.479 | 0 | 1 | 19 | glutamine family amino acid catabolic process |
| GO:0006730 | 0.345 | 2.479 | 0 | 1 | 19 | one-carbon metabolic process |
| GO:0042684 | 0.345 | 2.479 | 0 | 1 | 19 | cardioblast cell fate commitment |
| GO:0006626 | 0.345 | 2.479 | 0 | 1 | 19 | protein targeting to mitochondrion |
| GO:0045475 | 0.345 | 2.479 | 0 | 1 | 19 | locomotor rhythm |
| GO:0006919 | 0.345 | 2.479 | 0 | 1 | 19 | activation of cysteine-type endopeptidase activity involved in apoptotic process |
| GO:0097202 | 0.345 | 2.479 | 0 | 1 | 19 | activation of cysteine-type endopeptidase activity |
| GO:0045939 | 0.345 | 2.479 | 0 | 1 | 19 | negative regulation of steroid metabolic process |
| GO:0051084 | 0.345 | 2.479 | 0 | 1 | 19 | 'de novo' posttranslational protein folding |
| GO:0007140 | 0.345 | 2.479 | 0 | 1 | 19 | male meiosis |
| GO:0030817 | 0.345 | 2.479 | 0 | 1 | 19 | regulation of cAMP biosynthetic process |
| GO:0046329 | 0.345 | 2.479 | 0 | 1 | 19 | negative regulation of JNK cascade |
| GO:0051294 | 0.345 | 2.479 | 0 | 1 | 19 | establishment of spindle orientation |
| GO:0002224 | 0.345 | 2.479 | 0 | 1 | 19 | toll-like receptor signaling pathway |
| GO:0002807 | 0.345 | 2.479 | 0 | 1 | 19 | positive regulation of antimicrobial peptide biosynthetic process |
| GO:0002573 | 0.345 | 2.479 | 0 | 1 | 19 | myeloid leukocyte differentiation |
| GO:0002526 | 0.345 | 2.479 | 0 | 1 | 19 | acute inflammatory response |
| GO:0051646 | 0.345 | 2.479 | 0 | 1 | 19 | mitochondrion localization |
| GO:0044091 | 0.345 | 2.479 | 0 | 1 | 19 | membrane biogenesis |
| GO:0010874 | 0.345 | 2.479 | 0 | 1 | 19 | regulation of cholesterol efflux |
| GO:0015914 | 0.345 | 2.479 | 0 | 1 | 19 | phospholipid transport |
| GO:0034375 | 0.345 | 2.479 | 0 | 1 | 19 | high-density lipoprotein particle remodeling |
| GO:0060322 | 0.345 | 2.479 | 0 | 1 | 19 | head development |
| GO:0003206 | 0.345 | 2.479 | 0 | 1 | 19 | cardiac chamber morphogenesis |
| GO:0042078 | 0.345 | 2.479 | 0 | 1 | 19 | germ-line stem cell division |
| GO:0009165 | 0.346 | 1.366 | 3 | 4 | 135 | nucleotide biosynthetic process |
| GO:0015980 | 0.346 | 1.366 | 3 | 4 | 135 | energy derivation by oxidation of organic compounds |
| GO:0006576 | 0.348 | 1.458 | 2 | 3 | 95 | cellular biogenic amine metabolic process |
| GO:0009199 | 0.348 | 1.201 | 8 | 9 | 345 | ribonucleoside triphosphate metabolic process |
| GO:1901136 | 0.349 | 1.187 | 9 | 10 | 388 | carbohydrate derivative catabolic process |
| GO:0009164 | 0.350 | 1.301 | 4 | 5 | 177 | nucleoside catabolic process |
| GO:0032963 | 0.350 | 1.654 | 1 | 2 | 56 | collagen metabolic process |
| GO:0030522 | 0.350 | 1.654 | 1 | 2 | 56 | intracellular receptor mediated signaling pathway |
| GO:0046890 | 0.350 | 1.654 | 1 | 2 | 56 | regulation of lipid biosynthetic process |
| GO:0008033 | 0.350 | 1.654 | 1 | 2 | 56 | tRNA processing |
| GO:0000271 | 0.350 | 1.654 | 1 | 2 | 56 | polysaccharide biosynthetic process |
| GO:0044264 | 0.350 | 1.654 | 1 | 2 | 56 | cellular polysaccharide metabolic process |
| GO:0015931 | 0.351 | 1.355 | 3 | 4 | 136 | nucleobase-containing compound transport |
| GO:0009259 | 0.352 | 1.183 | 9 | 10 | 389 | ribonucleotide metabolic process |
| GO:0006917 | 0.354 | 1.442 | 2 | 3 | 96 | induction of apoptosis |
| GO:0051325 | 0.354 | 1.293 | 4 | 5 | 178 | interphase |
| GO:0046879 | 0.358 | 1.624 | 1 | 2 | 57 | hormone secretion |
| GO:0044272 | 0.358 | 1.624 | 1 | 2 | 57 | sulfur compound biosynthetic process |
| GO:0007292 | 0.358 | 1.177 | 9 | 10 | 391 | female gamete generation |
| GO:0006458 | 0.359 | 2.348 | 0 | 1 | 20 | 'de novo' protein folding |
| GO:0042771 | 0.359 | 2.348 | 0 | 1 | 20 | DNA damage response, signal transduction by p53 class mediator resulting in induction of apoptosis |
| GO:0060911 | 0.359 | 2.348 | 0 | 1 | 20 | cardiac cell fate commitment |
| GO:0019217 | 0.359 | 2.348 | 0 | 1 | 20 | regulation of fatty acid metabolic process |
| GO:0050851 | 0.359 | 2.348 | 0 | 1 | 20 | antigen receptor-mediated signaling pathway |
| GO:0050886 | 0.359 | 2.348 | 0 | 1 | 20 | endocrine process |
| GO:0050870 | 0.359 | 2.348 | 0 | 1 | 20 | positive regulation of T cell activation |
| GO:0050832 | 0.359 | 2.348 | 0 | 1 | 20 | defense response to fungus |
| GO:0022037 | 0.359 | 2.348 | 0 | 1 | 20 | metencephalon development |
| GO:0001703 | 0.359 | 2.348 | 0 | 1 | 20 | gastrulation with mouth forming first |
| GO:0035020 | 0.359 | 2.348 | 0 | 1 | 20 | regulation of Rac protein signal transduction |
| GO:0014812 | 0.359 | 2.348 | 0 | 1 | 20 | muscle cell migration |
| GO:0006901 | 0.359 | 2.348 | 0 | 1 | 20 | vesicle coating |
| GO:0032943 | 0.359 | 2.348 | 0 | 1 | 20 | mononuclear cell proliferation |
| GO:0051092 | 0.359 | 2.348 | 0 | 1 | 20 | positive regulation of NF-kappaB transcription factor activity |
| GO:0048678 | 0.359 | 2.348 | 0 | 1 | 20 | response to axon injury |
| GO:0043691 | 0.359 | 2.348 | 0 | 1 | 20 | reverse cholesterol transport |
| GO:0010004 | 0.359 | 2.348 | 0 | 1 | 20 | gastrulation involving germ band extension |
| GO:0090278 | 0.359 | 2.348 | 0 | 1 | 20 | negative regulation of peptide hormone secretion |
| GO:0046651 | 0.359 | 2.348 | 0 | 1 | 20 | lymphocyte proliferation |
| GO:0046676 | 0.359 | 2.348 | 0 | 1 | 20 | negative regulation of insulin secretion |
| GO:0031016 | 0.359 | 2.348 | 0 | 1 | 20 | pancreas development |
| GO:0010517 | 0.359 | 2.348 | 0 | 1 | 20 | regulation of phospholipase activity |
| GO:0003073 | 0.359 | 2.348 | 0 | 1 | 20 | regulation of systemic arterial blood pressure |
| GO:0016081 | 0.359 | 2.348 | 0 | 1 | 20 | synaptic vesicle docking involved in exocytosis |
| GO:0034368 | 0.359 | 2.348 | 0 | 1 | 20 | protein-lipid complex remodeling |
| GO:0034369 | 0.359 | 2.348 | 0 | 1 | 20 | plasma lipoprotein particle remodeling |
| GO:0003151 | 0.359 | 2.348 | 0 | 1 | 20 | outflow tract morphogenesis |
| GO:0003230 | 0.359 | 2.348 | 0 | 1 | 20 | cardiac atrium development |
| GO:0042074 | 0.359 | 2.348 | 0 | 1 | 20 | cell migration involved in gastrulation |
| GO:0071345 | 0.360 | 1.426 | 2 | 3 | 97 | cellular response to cytokine stimulus |
| GO:0009141 | 0.364 | 1.183 | 8 | 9 | 350 | nucleoside triphosphate metabolic process |
| GO:0030099 | 0.366 | 1.595 | 1 | 2 | 58 | myeloid cell differentiation |
| GO:0033205 | 0.366 | 1.595 | 1 | 2 | 58 | cell cycle cytokinesis |
| GO:0043900 | 0.366 | 1.595 | 1 | 2 | 58 | regulation of multi-organism process |
| GO:0015833 | 0.366 | 1.595 | 1 | 2 | 58 | peptide transport |
| GO:0010959 | 0.366 | 1.595 | 1 | 2 | 58 | regulation of metal ion transport |
| GO:0002165 | 0.370 | 1.190 | 7 | 8 | 309 | instar larval or pupal development |
| GO:0070201 | 0.371 | 1.315 | 3 | 4 | 140 | regulation of establishment of protein localization |
| GO:0009791 | 0.373 | 1.161 | 9 | 10 | 396 | post-embryonic development |
| GO:0070933 | 0.373 | 2.230 | 0 | 1 | 21 | histone H4 deacetylation |
| GO:0034644 | 0.373 | 2.230 | 0 | 1 | 21 | cellular response to UV |
| GO:0042430 | 0.373 | 2.230 | 0 | 1 | 21 | indole-containing compound metabolic process |
| GO:0006414 | 0.373 | 2.230 | 0 | 1 | 21 | translational elongation |
| GO:0042246 | 0.373 | 2.230 | 0 | 1 | 21 | tissue regeneration |
| GO:0040035 | 0.373 | 2.230 | 0 | 1 | 21 | hermaphrodite genitalia development |
| GO:0006586 | 0.373 | 2.230 | 0 | 1 | 21 | indolalkylamine metabolic process |
| GO:0006662 | 0.373 | 2.230 | 0 | 1 | 21 | glycerol ether metabolic process |
| GO:2001022 | 0.373 | 2.230 | 0 | 1 | 21 | positive regulation of response to DNA damage stimulus |
| GO:0045448 | 0.373 | 2.230 | 0 | 1 | 21 | mitotic cell cycle, embryonic |
| GO:0048010 | 0.373 | 2.230 | 0 | 1 | 21 | vascular endothelial growth factor receptor signaling pathway |
| GO:0001756 | 0.373 | 2.230 | 0 | 1 | 21 | somitogenesis |
| GO:1900371 | 0.373 | 2.230 | 0 | 1 | 21 | regulation of purine nucleotide biosynthetic process |
| GO:0001824 | 0.373 | 2.230 | 0 | 1 | 21 | blastocyst development |
| GO:0032873 | 0.373 | 2.230 | 0 | 1 | 21 | negative regulation of stress-activated MAPK cascade |
| GO:0006921 | 0.373 | 2.230 | 0 | 1 | 21 | cellular component disassembly involved in apoptotic process |
| GO:0050921 | 0.373 | 2.230 | 0 | 1 | 21 | positive regulation of chemotaxis |
| GO:0035329 | 0.373 | 2.230 | 0 | 1 | 21 | hippo signaling cascade |
| GO:2001251 | 0.373 | 2.230 | 0 | 1 | 21 | negative regulation of chromosome organization |
| GO:0019933 | 0.373 | 2.230 | 0 | 1 | 21 | cAMP-mediated signaling |
| GO:0035126 | 0.373 | 2.230 | 0 | 1 | 21 | post-embryonic genitalia morphogenesis |
| GO:0009620 | 0.373 | 2.230 | 0 | 1 | 21 | response to fungus |
| GO:0048803 | 0.373 | 2.230 | 0 | 1 | 21 | imaginal disc-derived male genitalia morphogenesis |
| GO:0048805 | 0.373 | 2.230 | 0 | 1 | 21 | imaginal disc-derived genitalia morphogenesis |
| GO:0045921 | 0.373 | 2.230 | 0 | 1 | 21 | positive regulation of exocytosis |
| GO:0009996 | 0.373 | 2.230 | 0 | 1 | 21 | negative regulation of cell fate specification |
| GO:0030814 | 0.373 | 2.230 | 0 | 1 | 21 | regulation of cAMP metabolic process |
| GO:0030802 | 0.373 | 2.230 | 0 | 1 | 21 | regulation of cyclic nucleotide biosynthetic process |
| GO:0030808 | 0.373 | 2.230 | 0 | 1 | 21 | regulation of nucleotide biosynthetic process |
| GO:0043506 | 0.373 | 2.230 | 0 | 1 | 21 | regulation of JUN kinase activity |
| GO:0007629 | 0.373 | 2.230 | 0 | 1 | 21 | flight behavior |
| GO:0002221 | 0.373 | 2.230 | 0 | 1 | 21 | pattern recognition receptor signaling pathway |
| GO:0002792 | 0.373 | 2.230 | 0 | 1 | 21 | negative regulation of peptide secretion |
| GO:0002758 | 0.373 | 2.230 | 0 | 1 | 21 | innate immune response-activating signal transduction |
| GO:0010454 | 0.373 | 2.230 | 0 | 1 | 21 | negative regulation of cell fate commitment |
| GO:0046888 | 0.373 | 2.230 | 0 | 1 | 21 | negative regulation of hormone secretion |
| GO:0070303 | 0.373 | 2.230 | 0 | 1 | 21 | negative regulation of stress-activated protein kinase signaling cascade |
| GO:0034367 | 0.373 | 2.230 | 0 | 1 | 21 | macromolecular complex remodeling |
| GO:0018904 | 0.373 | 2.230 | 0 | 1 | 21 | organic ether metabolic process |
| GO:0031577 | 0.373 | 2.230 | 0 | 1 | 21 | spindle checkpoint |
| GO:0016486 | 0.373 | 2.230 | 0 | 1 | 21 | peptide hormone processing |
| GO:0070661 | 0.373 | 2.230 | 0 | 1 | 21 | leukocyte proliferation |
| GO:0019320 | 0.374 | 1.567 | 1 | 2 | 59 | hexose catabolic process |
| GO:0009615 | 0.374 | 1.567 | 1 | 2 | 59 | response to virus |
| GO:0009914 | 0.374 | 1.567 | 1 | 2 | 59 | hormone transport |
| GO:0034440 | 0.374 | 1.567 | 1 | 2 | 59 | lipid oxidation |
| GO:0071156 | 0.376 | 1.305 | 3 | 4 | 141 | regulation of cell cycle arrest |
| GO:0006732 | 0.376 | 1.256 | 4 | 5 | 183 | coenzyme metabolic process |
| GO:0002520 | 0.380 | 1.249 | 4 | 5 | 184 | immune system development |
| GO:0046530 | 0.380 | 1.249 | 4 | 5 | 184 | photoreceptor cell differentiation |
| GO:1901293 | 0.381 | 1.296 | 3 | 4 | 142 | nucleoside phosphate biosynthetic process |
| GO:0007586 | 0.382 | 1.539 | 1 | 2 | 60 | digestion |
| GO:0000084 | 0.382 | 1.539 | 1 | 2 | 60 | S phase of mitotic cell cycle |
| GO:0044259 | 0.382 | 1.539 | 1 | 2 | 60 | multicellular organismal macromolecule metabolic process |
| GO:0008037 | 0.386 | 1.286 | 3 | 4 | 143 | cell recognition |
| GO:0032940 | 0.387 | 1.170 | 7 | 8 | 314 | secretion by cell |
| GO:0014074 | 0.387 | 2.124 | 0 | 1 | 22 | response to purine-containing compound |
| GO:0032373 | 0.387 | 2.124 | 0 | 1 | 22 | positive regulation of sterol transport |
| GO:0032376 | 0.387 | 2.124 | 0 | 1 | 22 | positive regulation of cholesterol transport |
| GO:0050663 | 0.387 | 2.124 | 0 | 1 | 22 | cytokine secretion |
| GO:0009190 | 0.387 | 2.124 | 0 | 1 | 22 | cyclic nucleotide biosynthetic process |
| GO:0035019 | 0.387 | 2.124 | 0 | 1 | 22 | somatic stem cell maintenance |
| GO:0001881 | 0.387 | 2.124 | 0 | 1 | 22 | receptor recycling |
| GO:0030011 | 0.387 | 2.124 | 0 | 1 | 22 | maintenance of cell polarity |
| GO:0019935 | 0.387 | 2.124 | 0 | 1 | 22 | cyclic-nucleotide-mediated signaling |
| GO:0007006 | 0.387 | 2.124 | 0 | 1 | 22 | mitochondrial membrane organization |
| GO:0007483 | 0.387 | 2.124 | 0 | 1 | 22 | genital disc morphogenesis |
| GO:0002274 | 0.387 | 2.124 | 0 | 1 | 22 | myeloid leukocyte activation |
| GO:0051897 | 0.387 | 2.124 | 0 | 1 | 22 | positive regulation of protein kinase B signaling cascade |
| GO:0031000 | 0.387 | 2.124 | 0 | 1 | 22 | response to caffeine |
| GO:0072655 | 0.387 | 2.124 | 0 | 1 | 22 | establishment of protein localization to mitochondrion |
| GO:0000236 | 0.387 | 2.124 | 0 | 1 | 22 | mitotic prometaphase |
| GO:0005978 | 0.387 | 2.124 | 0 | 1 | 22 | glycogen biosynthetic process |
| GO:0051047 | 0.390 | 1.513 | 1 | 2 | 61 | positive regulation of secretion |
| GO:0051384 | 0.390 | 1.513 | 1 | 2 | 61 | response to glucocorticoid stimulus |
| GO:0060249 | 0.390 | 1.353 | 2 | 3 | 102 | anatomical structure homeostasis |
| GO:0045017 | 0.390 | 1.353 | 2 | 3 | 102 | glycerolipid biosynthetic process |
| GO:0051258 | 0.390 | 1.353 | 2 | 3 | 102 | protein polymerization |
| GO:0051656 | 0.390 | 1.353 | 2 | 3 | 102 | establishment of organelle localization |
| GO:0006897 | 0.390 | 1.166 | 7 | 8 | 315 | endocytosis |
| GO:0030111 | 0.396 | 1.340 | 2 | 3 | 103 | regulation of Wnt receptor signaling pathway |
| GO:0043547 | 0.396 | 1.340 | 2 | 3 | 103 | positive regulation of GTPase activity |
| GO:0018205 | 0.396 | 1.340 | 2 | 3 | 103 | peptidyl-lysine modification |
| GO:0010975 | 0.396 | 1.340 | 2 | 3 | 103 | regulation of neuron projection development |
| GO:0044236 | 0.397 | 1.488 | 1 | 2 | 62 | multicellular organismal metabolic process |
| GO:0016199 | 0.397 | 1.488 | 1 | 2 | 62 | axon midline choice point recognition |
| GO:0055086 | 0.399 | 1.108 | 13 | 14 | 580 | nucleobase-containing small molecule metabolic process |
| GO:0016236 | 0.401 | 2.027 | 1 | 1 | 23 | macroautophagy |
| GO:0019069 | 0.401 | 2.027 | 1 | 1 | 23 | viral capsid assembly |
| GO:0006379 | 0.401 | 2.027 | 1 | 1 | 23 | mRNA cleavage |
| GO:0006891 | 0.401 | 2.027 | 1 | 1 | 23 | intra-Golgi vesicle-mediated transport |
| GO:0032370 | 0.401 | 2.027 | 1 | 1 | 23 | positive regulation of lipid transport |
| GO:0006541 | 0.401 | 2.027 | 1 | 1 | 23 | glutamine metabolic process |
| GO:0009250 | 0.401 | 2.027 | 1 | 1 | 23 | glucan biosynthetic process |
| GO:0030032 | 0.401 | 2.027 | 1 | 1 | 23 | lamellipodium assembly |
| GO:0043001 | 0.401 | 2.027 | 1 | 1 | 23 | Golgi to plasma membrane protein transport |
| GO:0007469 | 0.401 | 2.027 | 1 | 1 | 23 | antennal development |
| GO:0051055 | 0.401 | 2.027 | 1 | 1 | 23 | negative regulation of lipid biosynthetic process |
| GO:0007426 | 0.401 | 2.027 | 1 | 1 | 23 | tracheal outgrowth, open tracheal system |
| GO:0030799 | 0.401 | 2.027 | 1 | 1 | 23 | regulation of cyclic nucleotide metabolic process |
| GO:0002429 | 0.401 | 2.027 | 1 | 1 | 23 | immune response-activating cell surface receptor signaling pathway |
| GO:0002768 | 0.401 | 2.027 | 1 | 1 | 23 | immune response-regulating cell surface receptor signaling pathway |
| GO:0051817 | 0.401 | 2.027 | 1 | 1 | 23 | modification of morphology or physiology of other organism involved in symbiotic interaction |
| GO:0046797 | 0.401 | 2.027 | 1 | 1 | 23 | viral procapsid maturation |
| GO:0000281 | 0.401 | 2.027 | 1 | 1 | 23 | cytokinesis after mitosis |
| GO:0015991 | 0.401 | 2.027 | 1 | 1 | 23 | ATP hydrolysis coupled proton transport |
| GO:0015988 | 0.401 | 2.027 | 1 | 1 | 23 | energy coupled proton transport, against electrochemical gradient |
| GO:0012502 | 0.402 | 1.326 | 2 | 3 | 104 | induction of programmed cell death |
| GO:0070925 | 0.405 | 1.463 | 1 | 2 | 63 | organelle assembly |
| GO:0071363 | 0.405 | 1.463 | 1 | 2 | 63 | cellular response to growth factor stimulus |
| GO:0046365 | 0.405 | 1.463 | 1 | 2 | 63 | monosaccharide catabolic process |
| GO:0008063 | 0.405 | 1.463 | 1 | 2 | 63 | Toll signaling pathway |
| GO:0016198 | 0.405 | 1.463 | 1 | 2 | 63 | axon choice point recognition |
| GO:0048563 | 0.406 | 1.181 | 5 | 6 | 233 | post-embryonic organ morphogenesis |
| GO:0007560 | 0.406 | 1.181 | 5 | 6 | 233 | imaginal disc morphogenesis |
| GO:0021700 | 0.407 | 1.208 | 4 | 5 | 190 | developmental maturation |
| GO:0051056 | 0.407 | 1.208 | 4 | 5 | 190 | regulation of small GTPase mediated signal transduction |
| GO:1901068 | 0.407 | 1.208 | 4 | 5 | 190 | guanosine-containing compound metabolic process |
| GO:0007444 | 0.410 | 1.143 | 7 | 8 | 321 | imaginal disc development |
| GO:0002252 | 0.411 | 1.241 | 3 | 4 | 148 | immune effector process |
| GO:0019637 | 0.413 | 1.089 | 15 | 16 | 674 | organophosphate metabolic process |
| GO:0031960 | 0.413 | 1.439 | 1 | 2 | 64 | response to corticosteroid stimulus |
| GO:0042157 | 0.413 | 1.439 | 1 | 2 | 64 | lipoprotein metabolic process |
| GO:0051222 | 0.413 | 1.439 | 1 | 2 | 64 | positive regulation of protein transport |
| GO:0042476 | 0.414 | 1.939 | 1 | 1 | 24 | odontogenesis |
| GO:0006305 | 0.414 | 1.939 | 1 | 1 | 24 | DNA alkylation |
| GO:0006306 | 0.414 | 1.939 | 1 | 1 | 24 | DNA methylation |
| GO:0042593 | 0.414 | 1.939 | 1 | 1 | 24 | glucose homeostasis |
| GO:0040001 | 0.414 | 1.939 | 1 | 1 | 24 | establishment of mitotic spindle localization |
| GO:0006536 | 0.414 | 1.939 | 1 | 1 | 24 | glutamate metabolic process |
| GO:0006900 | 0.414 | 1.939 | 1 | 1 | 24 | membrane budding |
| GO:0006903 | 0.414 | 1.939 | 1 | 1 | 24 | vesicle targeting |
| GO:0043467 | 0.414 | 1.939 | 1 | 1 | 24 | regulation of generation of precursor metabolites and energy |
| GO:0048808 | 0.414 | 1.939 | 1 | 1 | 24 | male genitalia morphogenesis |
| GO:0043433 | 0.414 | 1.939 | 1 | 1 | 24 | negative regulation of sequence-specific DNA binding transcription factor activity |
| GO:0007485 | 0.414 | 1.939 | 1 | 1 | 24 | imaginal disc-derived male genitalia development |
| GO:0007413 | 0.414 | 1.939 | 1 | 1 | 24 | axonal fasciculation |
| GO:0043266 | 0.414 | 1.939 | 1 | 1 | 24 | regulation of potassium ion transport |
| GO:0022904 | 0.414 | 1.939 | 1 | 1 | 24 | respiratory electron transport chain |
| GO:0051251 | 0.414 | 1.939 | 1 | 1 | 24 | positive regulation of lymphocyte activation |
| GO:0007632 | 0.414 | 1.939 | 1 | 1 | 24 | visual behavior |
| GO:0046164 | 0.414 | 1.939 | 1 | 1 | 24 | alcohol catabolic process |
| GO:0002696 | 0.414 | 1.939 | 1 | 1 | 24 | positive regulation of leukocyte activation |
| GO:0046843 | 0.414 | 1.939 | 1 | 1 | 24 | dorsal appendage formation |
| GO:0046486 | 0.416 | 1.232 | 3 | 4 | 149 | glycerolipid metabolic process |
| GO:0090407 | 0.416 | 1.232 | 3 | 4 | 149 | organophosphate biosynthetic process |
| GO:0015672 | 0.416 | 1.232 | 3 | 4 | 149 | monovalent inorganic cation transport |
| GO:0006913 | 0.420 | 1.188 | 4 | 5 | 193 | nucleocytoplasmic transport |
| GO:0050778 | 0.421 | 1.416 | 1 | 2 | 65 | positive regulation of immune response |
| GO:0051320 | 0.421 | 1.416 | 1 | 2 | 65 | S phase |
| GO:0010948 | 0.421 | 1.416 | 1 | 2 | 65 | negative regulation of cell cycle process |
| GO:0051241 | 0.426 | 1.275 | 2 | 3 | 108 | negative regulation of multicellular organismal process |
| GO:0034654 | 0.426 | 1.215 | 3 | 4 | 151 | nucleobase-containing compound biosynthetic process |
| GO:0019068 | 0.427 | 1.858 | 1 | 1 | 25 | virion assembly |
| GO:0006506 | 0.427 | 1.858 | 1 | 1 | 25 | GPI anchor biosynthetic process |
| GO:0009395 | 0.427 | 1.858 | 1 | 1 | 25 | phospholipid catabolic process |
| GO:0071482 | 0.427 | 1.858 | 1 | 1 | 25 | cellular response to light stimulus |
| GO:0061053 | 0.427 | 1.858 | 1 | 1 | 25 | somite development |
| GO:0007031 | 0.427 | 1.858 | 1 | 1 | 25 | peroxisome organization |
| GO:0030431 | 0.427 | 1.858 | 1 | 1 | 25 | sleep |
| GO:0035821 | 0.427 | 1.858 | 1 | 1 | 25 | modification of morphology or physiology of other organism |
| GO:0030838 | 0.427 | 1.858 | 1 | 1 | 25 | positive regulation of actin filament polymerization |
| GO:0051100 | 0.427 | 1.858 | 1 | 1 | 25 | negative regulation of binding |
| GO:0051101 | 0.427 | 1.858 | 1 | 1 | 25 | regulation of DNA binding |
| GO:0002218 | 0.427 | 1.858 | 1 | 1 | 25 | activation of innate immune response |
| GO:0051568 | 0.427 | 1.858 | 1 | 1 | 25 | histone H3-K4 methylation |
| GO:0033500 | 0.427 | 1.858 | 1 | 1 | 25 | carbohydrate homeostasis |
| GO:0031397 | 0.427 | 1.858 | 1 | 1 | 25 | negative regulation of protein ubiquitination |
| GO:0008299 | 0.427 | 1.858 | 1 | 1 | 25 | isoprenoid biosynthetic process |
| GO:0019827 | 0.428 | 1.394 | 1 | 2 | 66 | stem cell maintenance |
| GO:0043473 | 0.428 | 1.394 | 1 | 2 | 66 | pigmentation |
| GO:0051169 | 0.429 | 1.175 | 4 | 5 | 195 | nuclear transport |
| GO:0048193 | 0.431 | 1.263 | 2 | 3 | 109 | Golgi vesicle transport |
| GO:0051168 | 0.436 | 1.372 | 1 | 2 | 67 | nuclear export |
| GO:0008104 | 0.438 | 1.058 | 21 | 22 | 953 | protein localization |
| GO:0009072 | 0.440 | 1.783 | 1 | 1 | 26 | aromatic amino acid family metabolic process |
| GO:0040023 | 0.440 | 1.783 | 1 | 1 | 26 | establishment of nucleus localization |
| GO:0032414 | 0.440 | 1.783 | 1 | 1 | 26 | positive regulation of ion transmembrane transporter activity |
| GO:0009746 | 0.440 | 1.783 | 1 | 1 | 26 | response to hexose stimulus |
| GO:0009749 | 0.440 | 1.783 | 1 | 1 | 26 | response to glucose stimulus |
| GO:0048520 | 0.440 | 1.783 | 1 | 1 | 26 | positive regulation of behavior |
| GO:0007484 | 0.440 | 1.783 | 1 | 1 | 26 | imaginal disc-derived genitalia development |
| GO:0046058 | 0.440 | 1.783 | 1 | 1 | 26 | cAMP metabolic process |
| GO:0030902 | 0.440 | 1.783 | 1 | 1 | 26 | hindbrain development |
| GO:0072332 | 0.440 | 1.783 | 1 | 1 | 26 | signal transduction by p53 class mediator resulting in induction of apoptosis |
| GO:0018279 | 0.440 | 1.783 | 1 | 1 | 26 | protein N-linked glycosylation via asparagine |
| GO:0010951 | 0.440 | 1.783 | 1 | 1 | 26 | negative regulation of endopeptidase activity |
| GO:0034394 | 0.440 | 1.783 | 1 | 1 | 26 | protein localization to cell surface |
| GO:0003279 | 0.440 | 1.783 | 1 | 1 | 26 | cardiac septum development |
| GO:0034284 | 0.440 | 1.783 | 1 | 1 | 26 | response to monosaccharide stimulus |
| GO:0006024 | 0.440 | 1.783 | 1 | 1 | 26 | glycosaminoglycan biosynthetic process |
| GO:0016358 | 0.440 | 1.190 | 3 | 4 | 154 | dendrite development |
| GO:0019048 | 0.443 | 1.239 | 2 | 3 | 111 | virus-host interaction |
| GO:0008015 | 0.443 | 1.239 | 2 | 3 | 111 | blood circulation |
| GO:0016485 | 0.443 | 1.239 | 2 | 3 | 111 | protein processing |
| GO:0008045 | 0.443 | 1.351 | 1 | 2 | 68 | motor axon guidance |
| GO:0006836 | 0.449 | 1.228 | 2 | 3 | 112 | neurotransmitter transport |
| GO:0006612 | 0.451 | 1.331 | 2 | 2 | 69 | protein targeting to membrane |
| GO:0032869 | 0.451 | 1.331 | 2 | 2 | 69 | cellular response to insulin stimulus |
| GO:0048864 | 0.451 | 1.331 | 2 | 2 | 69 | stem cell development |
| GO:0051297 | 0.451 | 1.331 | 2 | 2 | 69 | centrosome organization |
| GO:0032012 | 0.452 | 1.714 | 1 | 1 | 27 | regulation of ARF protein signal transduction |
| GO:0001101 | 0.452 | 1.714 | 1 | 1 | 27 | response to acid |
| GO:0009168 | 0.452 | 1.714 | 1 | 1 | 27 | purine ribonucleoside monophosphate biosynthetic process |
| GO:0009127 | 0.452 | 1.714 | 1 | 1 | 27 | purine nucleoside monophosphate biosynthetic process |
| GO:0001889 | 0.452 | 1.714 | 1 | 1 | 27 | liver development |
| GO:0071248 | 0.452 | 1.714 | 1 | 1 | 27 | cellular response to metal ion |
| GO:0048704 | 0.452 | 1.714 | 1 | 1 | 27 | embryonic skeletal system morphogenesis |
| GO:0048706 | 0.452 | 1.714 | 1 | 1 | 27 | embryonic skeletal system development |
| GO:0030512 | 0.452 | 1.714 | 1 | 1 | 27 | negative regulation of transforming growth factor beta receptor signaling pathway |
| GO:0010466 | 0.452 | 1.714 | 1 | 1 | 27 | negative regulation of peptidase activity |
| GO:0018196 | 0.452 | 1.714 | 1 | 1 | 27 | peptidyl-asparagine modification |
| GO:0044275 | 0.452 | 1.714 | 1 | 1 | 27 | cellular carbohydrate catabolic process |
| GO:0003006 | 0.454 | 1.068 | 12 | 13 | 557 | developmental process involved in reproduction |
| GO:0006364 | 0.455 | 1.216 | 2 | 3 | 113 | rRNA processing |
| GO:0045926 | 0.455 | 1.216 | 2 | 3 | 113 | negative regulation of growth |
| GO:0051235 | 0.455 | 1.216 | 2 | 3 | 113 | maintenance of location |
| GO:0050768 | 0.458 | 1.311 | 2 | 2 | 70 | negative regulation of neurogenesis |
| GO:0003013 | 0.460 | 1.205 | 3 | 3 | 114 | circulatory system process |
| GO:0072523 | 0.461 | 1.089 | 7 | 8 | 336 | purine-containing compound catabolic process |
| GO:0019067 | 0.464 | 1.651 | 1 | 1 | 28 | viral assembly, maturation, egress, and release |
| GO:0009069 | 0.464 | 1.651 | 1 | 1 | 28 | serine family amino acid metabolic process |
| GO:0032411 | 0.464 | 1.651 | 1 | 1 | 28 | positive regulation of transporter activity |
| GO:0042659 | 0.464 | 1.651 | 1 | 1 | 28 | regulation of cell fate specification |
| GO:0006505 | 0.464 | 1.651 | 1 | 1 | 28 | GPI anchor metabolic process |
| GO:0009167 | 0.464 | 1.651 | 1 | 1 | 28 | purine ribonucleoside monophosphate metabolic process |
| GO:0009126 | 0.464 | 1.651 | 1 | 1 | 28 | purine nucleoside monophosphate metabolic process |
| GO:0006937 | 0.464 | 1.651 | 1 | 1 | 28 | regulation of muscle contraction |
| GO:0061008 | 0.464 | 1.651 | 1 | 1 | 28 | hepaticobiliary system development |
| GO:0007530 | 0.464 | 1.651 | 1 | 1 | 28 | sex determination |
| GO:0046889 | 0.464 | 1.651 | 1 | 1 | 28 | positive regulation of lipid biosynthetic process |
| GO:0010632 | 0.464 | 1.651 | 1 | 1 | 28 | regulation of epithelial cell migration |
| GO:0006023 | 0.464 | 1.651 | 1 | 1 | 28 | aminoglycan biosynthetic process |
| GO:0008202 | 0.465 | 1.151 | 3 | 4 | 159 | steroid metabolic process |
| GO:0022416 | 0.465 | 1.292 | 2 | 2 | 71 | chaeta development |
| GO:0007043 | 0.465 | 1.292 | 2 | 2 | 71 | cell-cell junction assembly |
| GO:0009408 | 0.473 | 1.274 | 2 | 2 | 72 | response to heat |
| GO:0019932 | 0.473 | 1.274 | 2 | 2 | 72 | second-messenger-mediated signaling |
| GO:0043269 | 0.473 | 1.274 | 2 | 2 | 72 | regulation of ion transport |
| GO:0042398 | 0.476 | 1.591 | 1 | 1 | 29 | cellular modified amino acid biosynthetic process |
| GO:0042308 | 0.476 | 1.591 | 1 | 1 | 29 | negative regulation of protein import into nucleus |
| GO:0042752 | 0.476 | 1.591 | 1 | 1 | 29 | regulation of circadian rhythm |
| GO:0032103 | 0.476 | 1.591 | 1 | 1 | 29 | positive regulation of response to external stimulus |
| GO:0048284 | 0.476 | 1.591 | 1 | 1 | 29 | organelle fusion |
| GO:0035206 | 0.476 | 1.591 | 1 | 1 | 29 | regulation of hemocyte proliferation |
| GO:0030282 | 0.476 | 1.591 | 1 | 1 | 29 | bone mineralization |
| GO:0007474 | 0.476 | 1.591 | 1 | 1 | 29 | imaginal disc-derived wing vein specification |
| GO:0030539 | 0.476 | 1.591 | 1 | 1 | 29 | male genitalia development |
| GO:0051293 | 0.476 | 1.591 | 1 | 1 | 29 | establishment of spindle localization |
| GO:0051216 | 0.476 | 1.591 | 1 | 1 | 29 | cartilage development |
| GO:0007602 | 0.476 | 1.591 | 1 | 1 | 29 | phototransduction |
| GO:0002784 | 0.476 | 1.591 | 1 | 1 | 29 | regulation of antimicrobial peptide production |
| GO:0002775 | 0.476 | 1.591 | 1 | 1 | 29 | antimicrobial peptide production |
| GO:0002777 | 0.476 | 1.591 | 1 | 1 | 29 | antimicrobial peptide biosynthetic process |
| GO:0010453 | 0.476 | 1.591 | 1 | 1 | 29 | regulation of cell fate commitment |
| GO:0002805 | 0.476 | 1.591 | 1 | 1 | 29 | regulation of antimicrobial peptide biosynthetic process |
| GO:0007067 | 0.477 | 1.080 | 7 | 7 | 296 | mitosis |
| GO:0006911 | 0.480 | 1.255 | 2 | 2 | 73 | phagocytosis, engulfment |
| GO:0046649 | 0.480 | 1.255 | 2 | 2 | 73 | lymphocyte activation |
| GO:0031023 | 0.487 | 1.238 | 2 | 2 | 74 | microtubule organizing center organization |
| GO:0006893 | 0.487 | 1.536 | 1 | 1 | 30 | Golgi to plasma membrane transport |
| GO:0032273 | 0.487 | 1.536 | 1 | 1 | 30 | positive regulation of protein polymerization |
| GO:0045637 | 0.487 | 1.536 | 1 | 1 | 30 | regulation of myeloid cell differentiation |
| GO:0030307 | 0.487 | 1.536 | 1 | 1 | 30 | positive regulation of cell growth |
| GO:0002168 | 0.487 | 1.536 | 1 | 1 | 30 | instar larval development |
| GO:0030968 | 0.487 | 1.536 | 1 | 1 | 30 | endoplasmic reticulum unfolded protein response |
| GO:0033865 | 0.487 | 1.536 | 1 | 1 | 30 | nucleoside bisphosphate metabolic process |
| GO:0031532 | 0.487 | 1.536 | 1 | 1 | 30 | actin cytoskeleton reorganization |
| GO:0048610 | 0.488 | 1.041 | 13 | 13 | 570 | cellular process involved in reproduction |
| GO:0000280 | 0.488 | 1.069 | 7 | 7 | 299 | nuclear division |
| GO:0006790 | 0.489 | 1.153 | 3 | 3 | 119 | sulfur compound metabolic process |
| GO:0051701 | 0.489 | 1.153 | 3 | 3 | 119 | interaction with host |
| GO:0061458 | 0.489 | 1.092 | 5 | 5 | 209 | reproductive system development |
| GO:0048608 | 0.489 | 1.092 | 5 | 5 | 209 | reproductive structure development |
| GO:0022415 | 0.489 | 1.114 | 4 | 4 | 164 | viral reproductive process |
| GO:0042176 | 0.494 | 1.221 | 2 | 2 | 75 | regulation of protein catabolic process |
| GO:0007613 | 0.494 | 1.221 | 2 | 2 | 75 | memory |
| GO:0051606 | 0.494 | 1.221 | 2 | 2 | 75 | detection of stimulus |
| GO:0050776 | 0.494 | 1.143 | 3 | 3 | 120 | regulation of immune response |
| GO:0048599 | 0.494 | 1.143 | 3 | 3 | 120 | oocyte development |
| GO:0009143 | 0.495 | 1.061 | 7 | 7 | 301 | nucleoside triphosphate catabolic process |
| GO:0009146 | 0.495 | 1.061 | 7 | 7 | 301 | purine nucleoside triphosphate catabolic process |
| GO:0009203 | 0.495 | 1.061 | 7 | 7 | 301 | ribonucleoside triphosphate catabolic process |
| GO:0009207 | 0.495 | 1.061 | 7 | 7 | 301 | purine ribonucleoside triphosphate catabolic process |
| GO:0045184 | 0.498 | 1.028 | 16 | 16 | 710 | establishment of protein localization |
| GO:0016203 | 0.499 | 1.485 | 1 | 1 | 31 | muscle attachment |
| GO:0034620 | 0.499 | 1.485 | 1 | 1 | 31 | cellular response to unfolded protein |
| GO:0034612 | 0.499 | 1.485 | 1 | 1 | 31 | response to tumor necrosis factor |
| GO:0019439 | 0.499 | 1.485 | 1 | 1 | 31 | aromatic compound catabolic process |
| GO:0006695 | 0.499 | 1.485 | 1 | 1 | 31 | cholesterol biosynthetic process |
| GO:0035050 | 0.499 | 1.485 | 1 | 1 | 31 | embryonic heart tube development |
| GO:0009110 | 0.499 | 1.485 | 1 | 1 | 31 | vitamin biosynthetic process |
| GO:0032851 | 0.499 | 1.485 | 1 | 1 | 31 | positive regulation of Rab GTPase activity |
| GO:0006904 | 0.499 | 1.485 | 1 | 1 | 31 | vesicle docking involved in exocytosis |
| GO:0048512 | 0.499 | 1.485 | 1 | 1 | 31 | circadian behavior |
| GO:0035825 | 0.499 | 1.485 | 1 | 1 | 31 | reciprocal DNA recombination |
| GO:0007131 | 0.499 | 1.485 | 1 | 1 | 31 | reciprocal meiotic recombination |
| GO:0002759 | 0.499 | 1.485 | 1 | 1 | 31 | regulation of antimicrobial humoral response |
| GO:0051650 | 0.499 | 1.485 | 1 | 1 | 31 | establishment of vesicle localization |
| GO:0051653 | 0.499 | 1.485 | 1 | 1 | 31 | spindle localization |
| GO:0008217 | 0.499 | 1.485 | 1 | 1 | 31 | regulation of blood pressure |
| GO:0008630 | 0.499 | 1.485 | 1 | 1 | 31 | DNA damage response, signal transduction resulting in induction of apoptosis |
| GO:0034763 | 0.499 | 1.485 | 1 | 1 | 31 | negative regulation of transmembrane transport |
| GO:0001505 | 0.500 | 1.133 | 3 | 3 | 121 | regulation of neurotransmitter levels |
| GO:2000027 | 0.501 | 1.204 | 2 | 2 | 76 | regulation of organ morphogenesis |
| GO:0009154 | 0.502 | 1.054 | 7 | 7 | 303 | purine ribonucleotide catabolic process |
| GO:0009261 | 0.502 | 1.054 | 7 | 7 | 303 | ribonucleotide catabolic process |
| GO:0000087 | 0.502 | 1.054 | 7 | 7 | 303 | M phase of mitotic cell cycle |
| GO:0042254 | 0.503 | 1.093 | 4 | 4 | 167 | ribosome biogenesis |
| GO:0007265 | 0.505 | 1.123 | 3 | 3 | 122 | Ras protein signal transduction |
| GO:0051049 | 0.506 | 1.039 | 9 | 9 | 395 | regulation of transport |
| GO:0001822 | 0.508 | 1.188 | 2 | 2 | 77 | kidney development |
| GO:0071214 | 0.508 | 1.188 | 2 | 2 | 77 | cellular response to abiotic stimulus |
| GO:0035265 | 0.508 | 1.188 | 2 | 2 | 77 | organ growth |
| GO:0022900 | 0.508 | 1.188 | 2 | 2 | 77 | electron transport chain |
| GO:0008203 | 0.508 | 1.188 | 2 | 2 | 77 | cholesterol metabolic process |
| GO:0051186 | 0.510 | 1.066 | 5 | 5 | 214 | cofactor metabolic process |
| GO:0006282 | 0.510 | 1.437 | 1 | 1 | 32 | regulation of DNA repair |
| GO:0050796 | 0.510 | 1.437 | 1 | 1 | 32 | regulation of insulin secretion |
| GO:0032313 | 0.510 | 1.437 | 1 | 1 | 32 | regulation of Rab GTPase activity |
| GO:0032483 | 0.510 | 1.437 | 1 | 1 | 32 | regulation of Rab protein signal transduction |
| GO:0009187 | 0.510 | 1.437 | 1 | 1 | 32 | cyclic nucleotide metabolic process |
| GO:0050919 | 0.510 | 1.437 | 1 | 1 | 32 | negative chemotaxis |
| GO:0007091 | 0.510 | 1.437 | 1 | 1 | 32 | mitotic metaphase/anaphase transition |
| GO:0007076 | 0.510 | 1.437 | 1 | 1 | 32 | mitotic chromosome condensation |
| GO:0048278 | 0.510 | 1.437 | 1 | 1 | 32 | vesicle docking |
| GO:0007311 | 0.510 | 1.437 | 1 | 1 | 32 | maternal specification of dorsal/ventral axis, oocyte, germ-line encoded |
| GO:0051592 | 0.510 | 1.437 | 1 | 1 | 32 | response to calcium ion |
| GO:0008088 | 0.510 | 1.437 | 1 | 1 | 32 | axon cargo transport |
| GO:0072527 | 0.510 | 1.437 | 1 | 1 | 32 | pyrimidine-containing compound metabolic process |
| GO:0072661 | 0.510 | 1.437 | 1 | 1 | 32 | protein targeting to plasma membrane |
| GO:0072001 | 0.511 | 1.114 | 3 | 3 | 123 | renal system development |
| GO:0019748 | 0.515 | 1.172 | 2 | 2 | 78 | secondary metabolic process |
| GO:0010324 | 0.515 | 1.172 | 2 | 2 | 78 | membrane invagination |
| GO:0046903 | 0.517 | 1.033 | 8 | 8 | 353 | secretion |
| GO:0046034 | 0.517 | 1.073 | 4 | 4 | 170 | ATP metabolic process |
| GO:0016042 | 0.517 | 1.073 | 4 | 4 | 170 | lipid catabolic process |
| GO:0070972 | 0.521 | 1.392 | 1 | 1 | 33 | protein localization to endoplasmic reticulum |
| GO:0006308 | 0.521 | 1.392 | 1 | 1 | 33 | DNA catabolic process |
| GO:0006487 | 0.521 | 1.392 | 1 | 1 | 33 | protein N-linked glycosylation |
| GO:0006749 | 0.521 | 1.392 | 1 | 1 | 33 | glutathione metabolic process |
| GO:0032102 | 0.521 | 1.392 | 1 | 1 | 33 | negative regulation of response to external stimulus |
| GO:0043094 | 0.521 | 1.392 | 1 | 1 | 33 | cellular metabolic compound salvage |
| GO:0022406 | 0.521 | 1.392 | 1 | 1 | 33 | membrane docking |
| GO:0007443 | 0.521 | 1.392 | 1 | 1 | 33 | Malpighian tubule morphogenesis |
| GO:0003002 | 0.521 | 1.025 | 9 | 9 | 400 | regionalization |
| GO:0007093 | 0.522 | 1.157 | 2 | 2 | 79 | mitotic cell cycle checkpoint |
| GO:0008610 | 0.524 | 1.026 | 8 | 8 | 355 | lipid biosynthetic process |
| GO:0030001 | 0.526 | 1.060 | 4 | 4 | 172 | metal ion transport |
| GO:0042440 | 0.529 | 1.142 | 2 | 2 | 80 | pigment metabolic process |
| GO:0072657 | 0.529 | 1.142 | 2 | 2 | 80 | protein localization to membrane |
| GO:0034754 | 0.529 | 1.142 | 2 | 2 | 80 | cellular hormone metabolic process |
| GO:0048707 | 0.530 | 1.031 | 6 | 6 | 265 | instar larval or pupal morphogenesis |
| GO:0045089 | 0.531 | 1.349 | 1 | 1 | 34 | positive regulation of innate immune response |
| GO:0001892 | 0.531 | 1.349 | 1 | 1 | 34 | embryonic placenta development |
| GO:0002064 | 0.531 | 1.349 | 1 | 1 | 34 | epithelial cell development |
| GO:0030718 | 0.531 | 1.349 | 1 | 1 | 34 | germ-line stem cell maintenance |
| GO:0046823 | 0.531 | 1.349 | 1 | 1 | 34 | negative regulation of nucleocytoplasmic transport |
| GO:0006914 | 0.535 | 1.127 | 2 | 2 | 81 | autophagy |
| GO:0046700 | 0.540 | 1.011 | 8 | 8 | 360 | heterocycle catabolic process |
| GO:0032880 | 0.540 | 1.041 | 4 | 4 | 175 | regulation of protein localization |
| GO:0006304 | 0.542 | 1.310 | 1 | 1 | 35 | DNA modification |
| GO:0006497 | 0.542 | 1.310 | 1 | 1 | 35 | protein lipidation |
| GO:0042158 | 0.542 | 1.310 | 1 | 1 | 35 | lipoprotein biosynthetic process |
| GO:0006582 | 0.542 | 1.310 | 1 | 1 | 35 | melanin metabolic process |
| GO:0002443 | 0.542 | 1.310 | 1 | 1 | 35 | leukocyte mediated immunity |
| GO:0046621 | 0.542 | 1.310 | 1 | 1 | 35 | negative regulation of organ growth |
| GO:0007156 | 0.542 | 1.113 | 2 | 2 | 82 | homophilic cell adhesion |
| GO:0006955 | 0.547 | 1.008 | 7 | 7 | 316 | immune response |
| GO:0035272 | 0.548 | 1.051 | 3 | 3 | 130 | exocrine system development |
| GO:0051223 | 0.548 | 1.051 | 3 | 3 | 130 | regulation of protein transport |
| GO:0009886 | 0.549 | 1.011 | 6 | 6 | 270 | post-embryonic morphogenesis |
| GO:0045333 | 0.549 | 1.099 | 2 | 2 | 83 | cellular respiration |
| GO:0009108 | 0.549 | 1.099 | 2 | 2 | 83 | coenzyme biosynthetic process |
| GO:0007186 | 0.549 | 1.029 | 4 | 4 | 177 | G-protein coupled receptor signaling pathway |
| GO:0006635 | 0.552 | 1.272 | 1 | 1 | 36 | fatty acid beta-oxidation |
| GO:0006984 | 0.552 | 1.272 | 1 | 1 | 36 | ER-nucleus signaling pathway |
| GO:0007565 | 0.552 | 1.272 | 1 | 1 | 36 | female pregnancy |
| GO:0090317 | 0.552 | 1.272 | 1 | 1 | 36 | negative regulation of intracellular protein transport |
| GO:0002576 | 0.552 | 1.272 | 1 | 1 | 36 | platelet degranulation |
| GO:0031349 | 0.552 | 1.272 | 1 | 1 | 36 | positive regulation of defense response |
| GO:0031214 | 0.552 | 1.272 | 1 | 1 | 36 | biomineral tissue development |
| GO:0003205 | 0.552 | 1.272 | 1 | 1 | 36 | cardiac chamber development |
| GO:0009617 | 0.554 | 1.043 | 3 | 3 | 131 | response to bacterium |
| GO:0043623 | 0.555 | 1.011 | 5 | 5 | 225 | cellular protein complex assembly |
| GO:0044270 | 0.556 | 0.996 | 8 | 8 | 365 | cellular nitrogen compound catabolic process |
| GO:0006753 | 0.558 | 0.989 | 12 | 12 | 551 | nucleoside phosphate metabolic process |
| GO:0030707 | 0.559 | 1.035 | 3 | 3 | 132 | ovarian follicle cell development |
| GO:0000910 | 0.559 | 1.035 | 3 | 3 | 132 | cytokinesis |
| GO:0007552 | 0.559 | 0.999 | 6 | 6 | 273 | metamorphosis |
| GO:0015031 | 0.561 | 0.986 | 15 | 15 | 691 | protein transport |
| GO:0071375 | 0.562 | 1.072 | 2 | 2 | 85 | cellular response to peptide hormone stimulus |
| GO:0006275 | 0.562 | 1.237 | 1 | 1 | 37 | regulation of DNA replication |
| GO:0032387 | 0.562 | 1.237 | 1 | 1 | 37 | negative regulation of intracellular transport |
| GO:0001890 | 0.562 | 1.237 | 1 | 1 | 37 | placenta development |
| GO:0009583 | 0.562 | 1.237 | 1 | 1 | 37 | detection of light stimulus |
| GO:0051048 | 0.562 | 1.237 | 1 | 1 | 37 | negative regulation of secretion |
| GO:0048640 | 0.562 | 1.237 | 1 | 1 | 37 | negative regulation of developmental growth |
| GO:0002700 | 0.562 | 1.237 | 1 | 1 | 37 | regulation of production of molecular mediator of immune response |
| GO:0000079 | 0.562 | 1.237 | 1 | 1 | 37 | regulation of cyclin-dependent protein kinase activity |
| GO:0051647 | 0.562 | 1.237 | 1 | 1 | 37 | nucleus localization |
| GO:0008629 | 0.562 | 1.237 | 1 | 1 | 37 | induction of apoptosis by intracellular signals |
| GO:0008038 | 0.564 | 1.027 | 3 | 3 | 133 | neuron recognition |
| GO:0045216 | 0.568 | 1.059 | 2 | 2 | 86 | cell-cell junction organization |
| GO:0042461 | 0.569 | 1.019 | 3 | 3 | 134 | photoreceptor cell development |
| GO:0006195 | 0.571 | 0.984 | 7 | 7 | 323 | purine nucleotide catabolic process |
| GO:0006446 | 0.571 | 1.203 | 1 | 1 | 38 | regulation of translational initiation |
| GO:0032412 | 0.571 | 1.203 | 1 | 1 | 38 | regulation of ion transmembrane transporter activity |
| GO:0048102 | 0.571 | 1.203 | 1 | 1 | 38 | autophagic cell death |
| GO:0007569 | 0.571 | 1.203 | 1 | 1 | 38 | cell aging |
| GO:0090276 | 0.571 | 1.203 | 1 | 1 | 38 | regulation of peptide hormone secretion |
| GO:1901184 | 0.571 | 1.203 | 1 | 1 | 38 | regulation of ERBB signaling pathway |
| GO:0000288 | 0.571 | 1.203 | 1 | 1 | 38 | nuclear-transcribed mRNA catabolic process, deadenylation-dependent decay |
| GO:0015992 | 0.571 | 1.203 | 1 | 1 | 38 | proton transport |
| GO:0042058 | 0.571 | 1.203 | 1 | 1 | 38 | regulation of epidermal growth factor receptor signaling pathway |
| GO:0006887 | 0.571 | 0.999 | 4 | 4 | 182 | exocytosis |
| GO:0010721 | 0.574 | 1.047 | 2 | 2 | 87 | negative regulation of cell development |
| GO:0048878 | 0.577 | 0.978 | 7 | 7 | 325 | chemical homeostasis |
| GO:0007163 | 0.578 | 0.983 | 5 | 5 | 231 | establishment or maintenance of cell polarity |
| GO:0009314 | 0.580 | 0.988 | 4 | 4 | 184 | response to radiation |
| GO:0035282 | 0.580 | 0.988 | 4 | 4 | 184 | segmentation |
| GO:0008652 | 0.581 | 1.035 | 2 | 2 | 88 | cellular amino acid biosynthetic process |
| GO:0006818 | 0.581 | 1.171 | 1 | 1 | 39 | hydrogen transport |
| GO:0001764 | 0.581 | 1.171 | 1 | 1 | 39 | neuron migration |
| GO:0071478 | 0.581 | 1.171 | 1 | 1 | 39 | cellular response to radiation |
| GO:0006944 | 0.581 | 1.171 | 1 | 1 | 39 | cellular membrane fusion |
| GO:0009743 | 0.581 | 1.171 | 1 | 1 | 39 | response to carbohydrate stimulus |
| GO:0007306 | 0.581 | 1.171 | 1 | 1 | 39 | eggshell chorion assembly |
| GO:0048619 | 0.581 | 1.171 | 1 | 1 | 39 | embryonic hindgut morphogenesis |
| GO:0035914 | 0.581 | 1.171 | 1 | 1 | 39 | skeletal muscle cell differentiation |
| GO:0031345 | 0.581 | 1.171 | 1 | 1 | 39 | negative regulation of cell projection organization |
| GO:0035108 | 0.587 | 1.023 | 2 | 2 | 89 | limb morphogenesis |
| GO:0031344 | 0.589 | 0.988 | 3 | 3 | 138 | regulation of cell projection organization |
| GO:0007600 | 0.590 | 0.970 | 5 | 5 | 234 | sensory perception |
| GO:0021782 | 0.590 | 1.141 | 1 | 1 | 40 | glial cell development |
| GO:0061025 | 0.590 | 1.141 | 1 | 1 | 40 | membrane fusion |
| GO:0022898 | 0.590 | 1.141 | 1 | 1 | 40 | regulation of transmembrane transporter activity |
| GO:0007455 | 0.590 | 1.141 | 1 | 1 | 40 | eye-antennal disc morphogenesis |
| GO:0007427 | 0.590 | 1.141 | 1 | 1 | 40 | epithelial cell migration, open tracheal system |
| GO:0090087 | 0.590 | 1.141 | 1 | 1 | 40 | regulation of peptide transport |
| GO:0010002 | 0.590 | 1.141 | 1 | 1 | 40 | cardioblast differentiation |
| GO:0002791 | 0.590 | 1.141 | 1 | 1 | 40 | regulation of peptide secretion |
| GO:0033692 | 0.590 | 1.141 | 1 | 1 | 40 | cellular polysaccharide biosynthetic process |
| GO:0008407 | 0.590 | 1.141 | 1 | 1 | 40 | chaeta morphogenesis |
| GO:0006508 | 0.590 | 0.967 | 19 | 18 | 843 | proteolysis |
| GO:0009628 | 0.593 | 0.963 | 10 | 10 | 471 | response to abiotic stimulus |
| GO:0006323 | 0.593 | 1.011 | 2 | 2 | 90 | DNA packaging |
| GO:0016125 | 0.599 | 0.999 | 2 | 2 | 91 | sterol metabolic process |
| GO:0022612 | 0.599 | 0.999 | 2 | 2 | 91 | gland morphogenesis |
| GO:0048469 | 0.599 | 0.973 | 3 | 3 | 140 | cell maturation |
| GO:0016126 | 0.599 | 1.112 | 1 | 1 | 41 | sterol biosynthetic process |
| GO:0006406 | 0.599 | 1.112 | 1 | 1 | 41 | mRNA export from nucleus |
| GO:0042552 | 0.599 | 1.112 | 1 | 1 | 41 | myelination |
| GO:0009612 | 0.599 | 1.112 | 1 | 1 | 41 | response to mechanical stimulus |
| GO:0002764 | 0.599 | 1.112 | 1 | 1 | 41 | immune response-regulating signaling pathway |
| GO:0002757 | 0.599 | 1.112 | 1 | 1 | 41 | immune response-activating signal transduction |
| GO:0034765 | 0.599 | 1.112 | 1 | 1 | 41 | regulation of ion transmembrane transport |
| GO:0009166 | 0.603 | 0.953 | 7 | 7 | 333 | nucleotide catabolic process |
| GO:0044419 | 0.604 | 0.966 | 3 | 3 | 141 | interspecies interaction between organisms |
| GO:0044403 | 0.604 | 0.966 | 3 | 3 | 141 | symbiosis, encompassing mutualism through parasitism |
| GO:0007369 | 0.605 | 0.988 | 2 | 2 | 92 | gastrulation |
| GO:0048232 | 0.606 | 0.955 | 4 | 4 | 190 | male gamete generation |
| GO:0007283 | 0.606 | 0.955 | 4 | 4 | 190 | spermatogenesis |
| GO:0006342 | 0.608 | 1.085 | 1 | 1 | 42 | chromatin silencing |
| GO:0006775 | 0.608 | 1.085 | 1 | 1 | 42 | fat-soluble vitamin metabolic process |
| GO:0009062 | 0.608 | 1.085 | 1 | 1 | 42 | fatty acid catabolic process |
| GO:0046847 | 0.608 | 1.085 | 1 | 1 | 42 | filopodium assembly |
| GO:0018105 | 0.608 | 1.085 | 1 | 1 | 42 | peptidyl-serine phosphorylation |
| GO:0044262 | 0.609 | 0.959 | 3 | 3 | 142 | cellular carbohydrate metabolic process |
| GO:1901292 | 0.610 | 0.947 | 7 | 7 | 335 | nucleoside phosphate catabolic process |
| GO:0044242 | 0.611 | 0.977 | 2 | 2 | 93 | cellular lipid catabolic process |
| GO:0009117 | 0.611 | 0.949 | 12 | 11 | 525 | nucleotide metabolic process |
| GO:0001508 | 0.617 | 0.966 | 2 | 2 | 94 | regulation of action potential |
| GO:0042384 | 0.617 | 1.059 | 1 | 1 | 43 | cilium assembly |
| GO:0006839 | 0.617 | 1.059 | 1 | 1 | 43 | mitochondrial transport |
| GO:0006661 | 0.617 | 1.059 | 1 | 1 | 43 | phosphatidylinositol biosynthetic process |
| GO:0022412 | 0.617 | 1.059 | 1 | 1 | 43 | cellular process involved in reproduction in multicellular organism |
| GO:0022407 | 0.617 | 1.059 | 1 | 1 | 43 | regulation of cell-cell adhesion |
| GO:0061333 | 0.617 | 1.059 | 1 | 1 | 43 | renal tubule morphogenesis |
| GO:0051495 | 0.617 | 1.059 | 1 | 1 | 43 | positive regulation of cytoskeleton organization |
| GO:0046883 | 0.617 | 1.059 | 1 | 1 | 43 | regulation of hormone secretion |
| GO:0031099 | 0.617 | 1.059 | 1 | 1 | 43 | regeneration |
| GO:0051028 | 0.622 | 0.956 | 2 | 2 | 95 | mRNA transport |
| GO:0034976 | 0.625 | 1.034 | 1 | 1 | 44 | response to endoplasmic reticulum stress |
| GO:0009156 | 0.625 | 1.034 | 1 | 1 | 44 | ribonucleoside monophosphate biosynthetic process |
| GO:0007368 | 0.625 | 1.034 | 1 | 1 | 44 | determination of left/right symmetry |
| GO:0007304 | 0.625 | 1.034 | 1 | 1 | 44 | chorion-containing eggshell formation |
| GO:0072002 | 0.625 | 1.034 | 1 | 1 | 44 | Malpighian tubule development |
| GO:0051224 | 0.625 | 1.034 | 1 | 1 | 44 | negative regulation of protein transport |
| GO:0046546 | 0.625 | 1.034 | 1 | 1 | 44 | development of primary male sexual characteristics |
| GO:0016079 | 0.625 | 1.034 | 1 | 1 | 44 | synaptic vesicle exocytosis |
| GO:0006812 | 0.627 | 0.928 | 5 | 5 | 244 | cation transport |
| GO:0006898 | 0.628 | 0.946 | 2 | 2 | 96 | receptor-mediated endocytosis |
| GO:0007498 | 0.628 | 0.946 | 2 | 2 | 96 | mesoderm development |
| GO:0034655 | 0.628 | 0.929 | 7 | 7 | 341 | nucleobase-containing compound catabolic process |
| GO:0006413 | 0.633 | 1.011 | 1 | 1 | 45 | translational initiation |
| GO:0019722 | 0.633 | 1.011 | 1 | 1 | 45 | calcium-mediated signaling |
| GO:0009124 | 0.633 | 1.011 | 1 | 1 | 45 | nucleoside monophosphate biosynthetic process |
| GO:0045814 | 0.633 | 1.011 | 1 | 1 | 45 | negative regulation of gene expression, epigenetic |
| GO:1901264 | 0.633 | 1.011 | 1 | 1 | 45 | carbohydrate derivative transport |
| GO:0050804 | 0.634 | 0.935 | 2 | 2 | 97 | regulation of synaptic transmission |
| GO:0050767 | 0.634 | 0.920 | 4 | 4 | 197 | regulation of neurogenesis |
| GO:2000602 | 0.639 | 0.926 | 2 | 2 | 98 | regulation of interphase of mitotic cell cycle |
| GO:0009161 | 0.641 | 0.988 | 1 | 1 | 46 | ribonucleoside monophosphate metabolic process |
| GO:0048645 | 0.641 | 0.988 | 1 | 1 | 46 | organ formation |
| GO:0018209 | 0.641 | 0.988 | 1 | 1 | 46 | peptidyl-serine modification |
| GO:0065004 | 0.641 | 0.988 | 1 | 1 | 46 | protein-DNA complex assembly |
| GO:0008587 | 0.641 | 0.988 | 1 | 1 | 46 | imaginal disc-derived wing margin morphogenesis |
| GO:0031572 | 0.641 | 0.988 | 1 | 1 | 46 | G2/M transition DNA damage checkpoint |
| GO:0031576 | 0.641 | 0.988 | 1 | 1 | 46 | G2/M transition checkpoint |
| GO:0046434 | 0.644 | 0.915 | 8 | 7 | 346 | organophosphate catabolic process |
| GO:0048749 | 0.645 | 0.909 | 5 | 5 | 249 | compound eye development |
| GO:0009953 | 0.646 | 0.906 | 3 | 3 | 150 | dorsal/ventral pattern formation |
| GO:0007548 | 0.646 | 0.905 | 4 | 4 | 200 | sex differentiation |
| GO:2001020 | 0.649 | 0.967 | 1 | 1 | 47 | regulation of response to DNA damage stimulus |
| GO:0009123 | 0.649 | 0.967 | 1 | 1 | 47 | nucleoside monophosphate metabolic process |
| GO:0097190 | 0.649 | 0.967 | 1 | 1 | 47 | apoptotic signaling pathway |
| GO:0035317 | 0.649 | 0.967 | 1 | 1 | 47 | imaginal disc-derived wing hair organization |
| GO:0030703 | 0.649 | 0.967 | 1 | 1 | 47 | eggshell formation |
| GO:0043279 | 0.649 | 0.967 | 1 | 1 | 47 | response to alkaloid |
| GO:0009725 | 0.652 | 0.901 | 6 | 5 | 251 | response to hormone stimulus |
| GO:0006333 | 0.657 | 0.946 | 1 | 1 | 48 | chromatin assembly or disassembly |
| GO:0046356 | 0.657 | 0.946 | 1 | 1 | 48 | acetyl-CoA catabolic process |
| GO:0006099 | 0.657 | 0.946 | 1 | 1 | 48 | tricarboxylic acid cycle |
| GO:0048732 | 0.661 | 0.887 | 4 | 4 | 204 | gland development |
| GO:0042391 | 0.663 | 0.882 | 3 | 3 | 154 | regulation of membrane potential |
| GO:0006720 | 0.665 | 0.926 | 1 | 1 | 49 | isoprenoid metabolic process |
| GO:0045454 | 0.665 | 0.926 | 1 | 1 | 49 | cell redox homeostasis |
| GO:0045197 | 0.665 | 0.926 | 1 | 1 | 49 | establishment or maintenance of epithelial cell apical/basal polarity |
| GO:0035316 | 0.665 | 0.926 | 1 | 1 | 49 | non-sensory hair organization |
| GO:0007088 | 0.665 | 0.926 | 1 | 1 | 49 | regulation of mitosis |
| GO:0030574 | 0.665 | 0.926 | 1 | 1 | 49 | collagen catabolic process |
| GO:0043583 | 0.665 | 0.926 | 1 | 1 | 49 | ear development |
| GO:0051783 | 0.665 | 0.926 | 1 | 1 | 49 | regulation of nuclear division |
| GO:0051648 | 0.665 | 0.926 | 1 | 1 | 49 | vesicle localization |
| GO:0009880 | 0.668 | 0.876 | 3 | 3 | 155 | embryonic pattern specification |
| GO:0006974 | 0.669 | 0.898 | 10 | 9 | 452 | response to DNA damage stimulus |
| GO:0009719 | 0.671 | 0.886 | 7 | 6 | 306 | response to endogenous stimulus |
| GO:0034637 | 0.672 | 0.907 | 1 | 1 | 50 | cellular carbohydrate biosynthetic process |
| GO:0009799 | 0.672 | 0.907 | 1 | 1 | 50 | specification of symmetry |
| GO:0009855 | 0.672 | 0.907 | 1 | 1 | 50 | determination of bilateral symmetry |
| GO:0035172 | 0.672 | 0.907 | 1 | 1 | 50 | hemocyte proliferation |
| GO:0000082 | 0.672 | 0.907 | 1 | 1 | 50 | G1/S transition of mitotic cell cycle |
| GO:0015074 | 0.676 | 0.864 | 3 | 3 | 157 | DNA integration |
| GO:0016579 | 0.679 | 0.889 | 1 | 1 | 51 | protein deubiquitination |
| GO:0050821 | 0.679 | 0.889 | 1 | 1 | 51 | protein stabilization |
| GO:0035315 | 0.679 | 0.889 | 1 | 1 | 51 | hair cell differentiation |
| GO:0017157 | 0.679 | 0.889 | 1 | 1 | 51 | regulation of exocytosis |
| GO:0072329 | 0.679 | 0.889 | 1 | 1 | 51 | monocarboxylic acid catabolic process |
| GO:0044243 | 0.679 | 0.889 | 1 | 1 | 51 | multicellular organismal catabolic process |
| GO:0010970 | 0.679 | 0.889 | 1 | 1 | 51 | microtubule-based transport |
| GO:0006959 | 0.682 | 0.854 | 2 | 2 | 106 | humoral immune response |
| GO:0009266 | 0.682 | 0.854 | 2 | 2 | 106 | response to temperature stimulus |
| GO:0007308 | 0.682 | 0.854 | 2 | 2 | 106 | oocyte construction |
| GO:0051969 | 0.682 | 0.854 | 2 | 2 | 106 | regulation of transmission of nerve impulse |
| GO:0035088 | 0.687 | 0.871 | 1 | 1 | 52 | establishment or maintenance of apical/basal cell polarity |
| GO:0009109 | 0.687 | 0.871 | 1 | 1 | 52 | coenzyme catabolic process |
| GO:0030168 | 0.687 | 0.871 | 1 | 1 | 52 | platelet activation |
| GO:0006631 | 0.687 | 0.856 | 5 | 4 | 211 | fatty acid metabolic process |
| GO:0071103 | 0.691 | 0.837 | 2 | 2 | 108 | DNA conformation change |
| GO:0031644 | 0.691 | 0.837 | 2 | 2 | 108 | regulation of neurological system process |
| GO:0034220 | 0.691 | 0.837 | 2 | 2 | 108 | ion transmembrane transport |
| GO:0019395 | 0.693 | 0.854 | 1 | 1 | 53 | fatty acid oxidation |
| GO:0009581 | 0.693 | 0.854 | 1 | 1 | 53 | detection of external stimulus |
| GO:0009582 | 0.693 | 0.854 | 1 | 1 | 53 | detection of abiotic stimulus |
| GO:0061245 | 0.693 | 0.854 | 1 | 1 | 53 | establishment or maintenance of bipolar cell polarity |
| GO:0030705 | 0.693 | 0.854 | 1 | 1 | 53 | cytoskeleton-dependent intracellular transport |
| GO:0007272 | 0.693 | 0.854 | 1 | 1 | 53 | ensheathment of neurons |
| GO:0002440 | 0.693 | 0.854 | 1 | 1 | 53 | production of molecular mediator of immune response |
| GO:0008366 | 0.693 | 0.854 | 1 | 1 | 53 | axon ensheathment |
| GO:0051188 | 0.696 | 0.829 | 2 | 2 | 109 | cofactor biosynthetic process |
| GO:0034762 | 0.696 | 0.829 | 2 | 2 | 109 | regulation of transmembrane transport |
| GO:0045664 | 0.697 | 0.836 | 4 | 3 | 162 | regulation of neuron differentiation |
| GO:0044282 | 0.697 | 0.836 | 4 | 3 | 162 | small molecule catabolic process |
| GO:0010927 | 0.697 | 0.836 | 4 | 3 | 162 | cellular component assembly involved in morphogenesis |
| GO:0001745 | 0.698 | 0.844 | 5 | 4 | 214 | compound eye morphogenesis |
| GO:0007310 | 0.700 | 0.838 | 1 | 1 | 54 | oocyte dorsal/ventral axis specification |
| GO:0030203 | 0.700 | 0.838 | 1 | 1 | 54 | glycosaminoglycan metabolic process |
| GO:0051187 | 0.700 | 0.838 | 1 | 1 | 54 | cofactor catabolic process |
| GO:0046661 | 0.700 | 0.838 | 1 | 1 | 54 | male sex differentiation |
| GO:0045137 | 0.704 | 0.826 | 4 | 3 | 164 | development of primary sexual characteristics |
| GO:0030261 | 0.707 | 0.823 | 1 | 1 | 55 | chromosome condensation |
| GO:0007442 | 0.707 | 0.823 | 1 | 1 | 55 | hindgut morphogenesis |
| GO:0043648 | 0.707 | 0.823 | 1 | 1 | 55 | dicarboxylic acid metabolic process |
| GO:0006816 | 0.713 | 0.807 | 1 | 1 | 56 | calcium ion transport |
| GO:0002119 | 0.713 | 0.807 | 1 | 1 | 56 | nematode larval development |
| GO:0002521 | 0.713 | 0.807 | 1 | 1 | 56 | leukocyte differentiation |
| GO:0010212 | 0.713 | 0.807 | 1 | 1 | 56 | response to ionizing radiation |
| GO:0032787 | 0.717 | 0.831 | 6 | 5 | 271 | monocarboxylic acid metabolic process |
| GO:0016050 | 0.720 | 0.793 | 1 | 1 | 57 | vesicle organization |
| GO:0070646 | 0.720 | 0.793 | 1 | 1 | 57 | protein modification by small protein removal |
| GO:0006399 | 0.720 | 0.792 | 3 | 2 | 114 | tRNA metabolic process |
| GO:0009060 | 0.726 | 0.779 | 1 | 1 | 58 | aerobic respiration |
| GO:0030178 | 0.726 | 0.779 | 1 | 1 | 58 | negative regulation of Wnt receptor signaling pathway |
| GO:0009798 | 0.727 | 0.795 | 4 | 3 | 170 | axis specification |
| GO:0061326 | 0.732 | 0.765 | 1 | 1 | 59 | renal tubule development |
| GO:0030239 | 0.732 | 0.765 | 1 | 1 | 59 | myofibril assembly |
| GO:0043297 | 0.732 | 0.765 | 1 | 1 | 59 | apical junction assembly |
| GO:0031123 | 0.732 | 0.765 | 1 | 1 | 59 | RNA 3'-end processing |
| GO:0042787 | 0.738 | 0.752 | 1 | 1 | 60 | protein ubiquitination involved in ubiquitin-dependent protein catabolic process |
| GO:0001708 | 0.738 | 0.752 | 1 | 1 | 60 | cell fate specification |
| GO:0045087 | 0.738 | 0.781 | 4 | 3 | 173 | innate immune response |
| GO:0045467 | 0.744 | 0.740 | 1 | 1 | 61 | R7 cell development |
| GO:0035214 | 0.744 | 0.740 | 1 | 1 | 61 | eye-antennal disc development |
| GO:0007224 | 0.744 | 0.740 | 1 | 1 | 61 | smoothened signaling pathway |
| GO:0007266 | 0.749 | 0.728 | 1 | 1 | 62 | Rho protein signal transduction |
| GO:0006873 | 0.759 | 0.772 | 5 | 4 | 233 | cellular ion homeostasis |
| GO:0060271 | 0.760 | 0.704 | 1 | 1 | 64 | cilium morphogenesis |
| GO:0046488 | 0.760 | 0.704 | 1 | 1 | 64 | phosphatidylinositol metabolic process |
| GO:0000070 | 0.760 | 0.704 | 1 | 1 | 64 | mitotic sister chromatid segregation |
| GO:0010001 | 0.766 | 0.693 | 1 | 1 | 65 | glial cell differentiation |
| GO:0000819 | 0.766 | 0.693 | 1 | 1 | 65 | sister chromatid segregation |
| GO:0007626 | 0.769 | 0.741 | 4 | 3 | 182 | locomotory behavior |
| GO:0070838 | 0.771 | 0.682 | 1 | 1 | 66 | divalent metal ion transport |
| GO:0006084 | 0.771 | 0.682 | 1 | 1 | 66 | acetyl-CoA metabolic process |
| GO:0006278 | 0.774 | 0.708 | 3 | 2 | 127 | RNA-dependent DNA replication |
| GO:0048545 | 0.774 | 0.708 | 3 | 2 | 127 | response to steroid hormone stimulus |
| GO:0007431 | 0.774 | 0.708 | 3 | 2 | 127 | salivary gland development |
| GO:0009913 | 0.776 | 0.672 | 1 | 1 | 67 | epidermal cell differentiation |
| GO:0042306 | 0.781 | 0.662 | 1 | 1 | 68 | regulation of protein import into nucleus |
| GO:0048546 | 0.781 | 0.662 | 1 | 1 | 68 | digestive tract morphogenesis |
| GO:0072511 | 0.781 | 0.662 | 1 | 1 | 68 | divalent inorganic cation transport |
| GO:0007346 | 0.781 | 0.724 | 4 | 3 | 186 | regulation of mitotic cell cycle |
| GO:0055082 | 0.782 | 0.745 | 5 | 4 | 241 | cellular chemical homeostasis |
| GO:0007350 | 0.785 | 0.691 | 3 | 2 | 130 | blastoderm segmentation |
| GO:0014070 | 0.786 | 0.652 | 2 | 1 | 69 | response to organic cyclic compound |
| GO:0008406 | 0.788 | 0.686 | 3 | 2 | 131 | gonad development |
| GO:0032196 | 0.791 | 0.643 | 2 | 1 | 70 | transposition |
| GO:1900180 | 0.791 | 0.643 | 2 | 1 | 70 | regulation of protein localization to nucleus |
| GO:0031647 | 0.791 | 0.643 | 2 | 1 | 70 | regulation of protein stability |
| GO:0051046 | 0.792 | 0.681 | 3 | 2 | 132 | regulation of secretion |
| GO:0006200 | 0.799 | 0.670 | 3 | 2 | 134 | ATP catabolic process |
| GO:0050817 | 0.799 | 0.670 | 3 | 2 | 134 | coagulation |
| GO:0007599 | 0.799 | 0.670 | 3 | 2 | 134 | hemostasis |
| GO:0046394 | 0.801 | 0.723 | 5 | 4 | 248 | carboxylic acid biosynthetic process |
| GO:0016053 | 0.801 | 0.723 | 5 | 4 | 248 | organic acid biosynthetic process |
| GO:0046395 | 0.802 | 0.665 | 3 | 2 | 135 | carboxylic acid catabolic process |
| GO:0016054 | 0.802 | 0.665 | 3 | 2 | 135 | organic acid catabolic process |
| GO:0030010 | 0.804 | 0.616 | 2 | 1 | 73 | establishment of cell polarity |
| GO:0016573 | 0.809 | 0.607 | 2 | 1 | 74 | histone acetylation |
| GO:0019228 | 0.809 | 0.607 | 2 | 1 | 74 | regulation of action potential in neuron |
| GO:0031032 | 0.809 | 0.607 | 2 | 1 | 74 | actomyosin structure organization |
| GO:0019725 | 0.809 | 0.731 | 7 | 5 | 306 | cellular homeostasis |
| GO:0007173 | 0.813 | 0.599 | 2 | 1 | 75 | epidermal growth factor receptor signaling pathway |
| GO:0038127 | 0.813 | 0.599 | 2 | 1 | 75 | ERBB signaling pathway |
| GO:0044283 | 0.814 | 0.726 | 7 | 5 | 308 | small molecule biosynthetic process |
| GO:0050801 | 0.818 | 0.702 | 6 | 4 | 255 | ion homeostasis |
| GO:0006814 | 0.821 | 0.583 | 2 | 1 | 77 | sodium ion transport |
| GO:0046331 | 0.821 | 0.583 | 2 | 1 | 77 | lateral inhibition |
| GO:0018393 | 0.821 | 0.583 | 2 | 1 | 77 | internal peptidyl-lysine acetylation |
| GO:0000956 | 0.821 | 0.583 | 2 | 1 | 77 | nuclear-transcribed mRNA catabolic process |
| GO:0060341 | 0.822 | 0.696 | 6 | 4 | 257 | regulation of cellular localization |
| GO:0032870 | 0.824 | 0.631 | 3 | 2 | 142 | cellular response to hormone stimulus |
| GO:0006811 | 0.827 | 0.712 | 7 | 5 | 314 | ion transport |
| GO:0006475 | 0.829 | 0.568 | 2 | 1 | 79 | internal protein amino acid acetylation |
| GO:0045466 | 0.833 | 0.560 | 2 | 1 | 80 | R7 cell differentiation |
| GO:0032844 | 0.833 | 0.560 | 2 | 1 | 80 | regulation of homeostatic process |
| GO:0048489 | 0.833 | 0.560 | 2 | 1 | 80 | synaptic vesicle transport |
| GO:0018394 | 0.833 | 0.560 | 2 | 1 | 80 | peptidyl-lysine acetylation |
| GO:0046822 | 0.836 | 0.553 | 2 | 1 | 81 | regulation of nucleocytoplasmic transport |
| GO:0006281 | 0.837 | 0.700 | 7 | 5 | 319 | DNA repair |
| GO:0006874 | 0.840 | 0.547 | 2 | 1 | 82 | cellular calcium ion homeostasis |
| GO:0030198 | 0.844 | 0.540 | 2 | 1 | 83 | extracellular matrix organization |
| GO:0051707 | 0.847 | 0.666 | 6 | 4 | 268 | response to other organism |
| GO:0009063 | 0.847 | 0.533 | 2 | 1 | 84 | cellular amino acid catabolic process |
| GO:0019730 | 0.847 | 0.533 | 2 | 1 | 84 | antimicrobial humoral response |
| GO:0006473 | 0.850 | 0.527 | 2 | 1 | 85 | protein acetylation |
| GO:0072503 | 0.850 | 0.527 | 2 | 1 | 85 | cellular divalent inorganic cation homeostasis |
| GO:0006402 | 0.854 | 0.521 | 2 | 1 | 86 | mRNA catabolic process |
| GO:0042067 | 0.854 | 0.521 | 2 | 1 | 86 | establishment of ommatidial planar polarity |
| GO:0009607 | 0.855 | 0.656 | 6 | 4 | 272 | response to biotic stimulus |
| GO:0055074 | 0.857 | 0.514 | 2 | 1 | 87 | calcium ion homeostasis |
| GO:0019058 | 0.860 | 0.508 | 2 | 1 | 88 | viral infectious cycle |
| GO:0033157 | 0.860 | 0.508 | 2 | 1 | 88 | regulation of intracellular protein transport |
| GO:0006259 | 0.861 | 0.760 | 17 | 13 | 757 | DNA metabolic process |
| GO:0009952 | 0.865 | 0.569 | 3 | 2 | 157 | anterior/posterior pattern specification |
| GO:0043543 | 0.866 | 0.497 | 2 | 1 | 90 | protein acylation |
| GO:0009948 | 0.872 | 0.486 | 2 | 1 | 92 | anterior/posterior axis specification |
| GO:0072507 | 0.875 | 0.480 | 2 | 1 | 93 | divalent inorganic cation homeostasis |
| GO:0050878 | 0.876 | 0.551 | 4 | 2 | 162 | regulation of body fluid levels |
| GO:0043062 | 0.878 | 0.475 | 2 | 1 | 94 | extracellular structure organization |
| GO:0090305 | 0.879 | 0.591 | 5 | 3 | 226 | nucleic acid phosphodiester bond hydrolysis |
| GO:0001754 | 0.882 | 0.541 | 4 | 2 | 165 | eye photoreceptor cell differentiation |
| GO:0006875 | 0.898 | 0.437 | 2 | 1 | 102 | cellular metal ion homeostasis |
| GO:0007264 | 0.900 | 0.560 | 5 | 3 | 238 | small GTPase mediated signal transduction |
| GO:0007309 | 0.900 | 0.433 | 2 | 1 | 103 | oocyte axis specification |
| GO:0008544 | 0.907 | 0.420 | 2 | 1 | 106 | epidermis development |
| GO:0055085 | 0.907 | 0.584 | 7 | 4 | 304 | transmembrane transport |
| GO:0071495 | 0.910 | 0.494 | 4 | 2 | 180 | cellular response to endogenous stimulus |
| GO:0006310 | 0.910 | 0.544 | 5 | 3 | 245 | DNA recombination |
| GO:0030855 | 0.915 | 0.405 | 2 | 1 | 110 | epithelial cell differentiation |
| GO:0060627 | 0.917 | 0.401 | 2 | 1 | 111 | regulation of vesicle-mediated transport |
| GO:0042051 | 0.919 | 0.397 | 2 | 1 | 112 | compound eye photoreceptor development |
| GO:0055065 | 0.922 | 0.390 | 3 | 1 | 114 | metal ion homeostasis |
| GO:0030003 | 0.924 | 0.387 | 3 | 1 | 115 | cellular cation homeostasis |
| GO:0042063 | 0.926 | 0.383 | 3 | 1 | 116 | gliogenesis |
| GO:0042462 | 0.932 | 0.370 | 3 | 1 | 120 | eye photoreceptor cell development |
| GO:0006633 | 0.932 | 0.370 | 3 | 1 | 120 | fatty acid biosynthetic process |
| GO:0032386 | 0.934 | 0.367 | 3 | 1 | 121 | regulation of intracellular transport |
| GO:0007596 | 0.939 | 0.355 | 3 | 1 | 125 | blood coagulation |
| GO:0001736 | 0.941 | 0.352 | 3 | 1 | 126 | establishment of planar polarity |
| GO:0007164 | 0.941 | 0.352 | 3 | 1 | 126 | establishment of tissue polarity |
| GO:0055080 | 0.960 | 0.307 | 3 | 1 | 144 | cation homeostasis |
| GO:0001738 | 0.962 | 0.303 | 3 | 1 | 146 | morphogenesis of a polarized epithelium |
| GO:0001751 | 0.968 | 0.287 | 3 | 1 | 154 | compound eye photoreceptor cell differentiation |
| GO:0007018 | 0.969 | 0.285 | 3 | 1 | 155 | microtubule-based movement |
| GO:0006260 | 0.972 | 0.419 | 7 | 3 | 314 | DNA replication |
