# Supplementary material for: Inducible Defenses Stay Up Late: Temporal Patterns of Immune Gene Expression in Tenebrio molitor
Source: G3 (Bethesda). 2014 Jun 1;4(6):947–55. doi: 10.1534/g3.113.008516 (PMC4065263; doi:10.1534/g3.113.008516)
Supplement: Supporting Information [file supp_g3.113.008516_TableS12.html]

TableS12 

# Inducible Defenses Stay Up Late: Temporal Patterns of Immune Gene Expression in *Tenebrio molitor*

Gene to GO MF test for over-representation

| GOMFID | Pvalue | OddsRatio | ExpCount | Count | Size | Term |
| GO:0005515 | 0.000 | 2.138 | 22 | 40 | 1102 | protein binding |
| GO:0004716 | 0.000 | Inf | 0 | 2 | 2 | receptor signaling protein tyrosine kinase activity |
| GO:0008142 | 0.000 | Inf | 0 | 2 | 2 | oxysterol binding |
| GO:0008170 | 0.000 | 5.684 | 1 | 7 | 70 | N-methyltransferase activity |
| GO:0015631 | 0.001 | 5.342 | 1 | 7 | 74 | tubulin binding |
| GO:0042054 | 0.001 | 5.990 | 1 | 6 | 57 | histone methyltransferase activity |
| GO:0003725 | 0.001 | 10.626 | 0 | 4 | 23 | double-stranded RNA binding |
| GO:0008092 | 0.001 | 2.958 | 5 | 13 | 242 | cytoskeletal protein binding |
| GO:0004022 | 0.001 | 99.913 | 0 | 2 | 3 | alcohol dehydrogenase (NAD) activity |
| GO:0045294 | 0.001 | 99.913 | 0 | 2 | 3 | alpha-catenin binding |
| GO:0000989 | 0.001 | 3.217 | 4 | 11 | 188 | transcription factor binding transcription factor activity |
| GO:0008017 | 0.001 | 5.355 | 1 | 6 | 63 | microtubule binding |
| GO:0003713 | 0.002 | 4.019 | 2 | 8 | 110 | transcription coactivator activity |
| GO:0032934 | 0.002 | 9.173 | 1 | 4 | 26 | sterol binding |
| GO:0000988 | 0.002 | 3.074 | 4 | 11 | 196 | protein binding transcription factor activity |
| GO:0003714 | 0.002 | 6.177 | 1 | 5 | 46 | transcription corepressor activity |
| GO:0030506 | 0.002 | 49.950 | 0 | 2 | 4 | ankyrin binding |
| GO:0018024 | 0.003 | 5.754 | 1 | 5 | 49 | histone-lysine N-methyltransferase activity |
| GO:0008276 | 0.003 | 4.691 | 1 | 6 | 71 | protein methyltransferase activity |
| GO:0003712 | 0.003 | 3.046 | 4 | 10 | 179 | transcription cofactor activity |
| GO:0016278 | 0.003 | 5.625 | 1 | 5 | 50 | lysine N-methyltransferase activity |
| GO:0016279 | 0.003 | 5.625 | 1 | 5 | 50 | protein-lysine N-methyltransferase activity |
| GO:0045296 | 0.004 | 11.583 | 0 | 3 | 16 | cadherin binding |
| GO:0008757 | 0.004 | 3.715 | 2 | 7 | 103 | S-adenosylmethionine-dependent methyltransferase activity |
| GO:0005496 | 0.005 | 6.299 | 1 | 4 | 36 | steroid binding |
| GO:0050839 | 0.007 | 4.514 | 1 | 5 | 61 | cell adhesion molecule binding |
| GO:0004525 | 0.008 | 19.973 | 0 | 2 | 7 | ribonuclease III activity |
| GO:0042800 | 0.008 | 8.360 | 0 | 3 | 21 | histone methyltransferase activity (H3-K4 specific) |
| GO:0003682 | 0.008 | 2.590 | 4 | 10 | 208 | chromatin binding |
| GO:0001871 | 0.009 | 3.207 | 2 | 7 | 118 | pattern binding |
| GO:0030247 | 0.009 | 3.207 | 2 | 7 | 118 | polysaccharide binding |
| GO:0003779 | 0.011 | 3.094 | 2 | 7 | 122 | actin binding |
| GO:0005319 | 0.012 | 4.911 | 1 | 4 | 45 | lipid transporter activity |
| GO:0016614 | 0.014 | 2.525 | 4 | 9 | 191 | oxidoreductase activity, acting on CH-OH group of donors |
| GO:0004725 | 0.015 | 3.712 | 1 | 5 | 73 | protein tyrosine phosphatase activity |
| GO:0005488 | 0.016 | 1.572 | 123 | 135 | 6201 | binding |
| GO:0043566 | 0.016 | 3.605 | 1 | 5 | 75 | structure-specific DNA binding |
| GO:0016811 | 0.017 | 4.374 | 1 | 4 | 50 | hydrolase activity, acting on carbon-nitrogen (but not peptide) bonds, in linear amides |
| GO:0050662 | 0.019 | 2.377 | 4 | 9 | 202 | coenzyme binding |
| GO:0019534 | 0.019 | 11.090 | 0 | 2 | 11 | toxin transporter activity |
| GO:0030246 | 0.020 | 2.365 | 4 | 9 | 203 | carbohydrate binding |
| GO:0008168 | 0.020 | 2.511 | 3 | 8 | 170 | methyltransferase activity |
| GO:0047800 | 0.020 | Inf | 0 | 1 | 1 | cysteamine dioxygenase activity |
| GO:0000009 | 0.020 | Inf | 0 | 1 | 1 | alpha-1,6-mannosyltransferase activity |
| GO:0008808 | 0.020 | Inf | 0 | 1 | 1 | cardiolipin synthase activity |
| GO:0005325 | 0.020 | Inf | 0 | 1 | 1 | peroxisomal fatty-acyl-CoA transporter activity |
| GO:0031210 | 0.020 | Inf | 0 | 1 | 1 | phosphatidylcholine binding |
| GO:0004034 | 0.020 | Inf | 0 | 1 | 1 | aldose 1-epimerase activity |
| GO:0071936 | 0.020 | Inf | 0 | 1 | 1 | coreceptor activity involved in Wnt receptor signaling pathway |
| GO:0015246 | 0.020 | Inf | 0 | 1 | 1 | fatty-acyl group transporter activity |
| GO:0004408 | 0.020 | Inf | 0 | 1 | 1 | holocytochrome-c synthase activity |
| GO:0051903 | 0.020 | Inf | 0 | 1 | 1 | S-(hydroxymethyl)glutathione dehydrogenase activity |
| GO:0042586 | 0.020 | Inf | 0 | 1 | 1 | peptide deformylase activity |
| GO:0004122 | 0.020 | Inf | 0 | 1 | 1 | cystathionine beta-synthase activity |
| GO:0003839 | 0.020 | Inf | 0 | 1 | 1 | gamma-glutamylcyclotransferase activity |
| GO:0052917 | 0.020 | Inf | 0 | 1 | 1 | dol-P-Man:Man(7)GlcNAc(2)-PP-Dol alpha-1,6-mannosyltransferase activity |
| GO:0004757 | 0.020 | Inf | 0 | 1 | 1 | sepiapterin reductase activity |
| GO:0030572 | 0.020 | Inf | 0 | 1 | 1 | phosphatidyltransferase activity |
| GO:0030523 | 0.020 | Inf | 0 | 1 | 1 | dihydrolipoamide S-acyltransferase activity |
| GO:0004742 | 0.020 | Inf | 0 | 1 | 1 | dihydrolipoyllysine-residue acetyltransferase activity |
| GO:0080007 | 0.020 | Inf | 0 | 1 | 1 | S-nitrosoglutathione reductase activity |
| GO:0015607 | 0.020 | Inf | 0 | 1 | 1 | fatty-acyl-CoA transporter activity |
| GO:0050072 | 0.020 | Inf | 0 | 1 | 1 | m7G(5')pppN diphosphatase activity |
| GO:0004552 | 0.020 | Inf | 0 | 1 | 1 | octanol dehydrogenase activity |
| GO:0042903 | 0.020 | Inf | 0 | 1 | 1 | tubulin deacetylase activity |
| GO:0047860 | 0.020 | Inf | 0 | 1 | 1 | diiodophenylpyruvate reductase activity |
| GO:0016810 | 0.020 | 3.408 | 2 | 5 | 79 | hydrolase activity, acting on carbon-nitrogen (but not peptide) bonds |
| GO:0008201 | 0.021 | 4.105 | 1 | 4 | 53 | heparin binding |
| GO:0016741 | 0.022 | 2.449 | 3 | 8 | 174 | transferase activity, transferring one-carbon groups |
| GO:0005520 | 0.023 | 9.980 | 0 | 2 | 12 | insulin-like growth factor binding |
| GO:0008270 | 0.024 | 1.537 | 23 | 32 | 1135 | zinc ion binding |
| GO:0045182 | 0.031 | 8.315 | 0 | 2 | 14 | translation regulator activity |
| GO:0050661 | 0.037 | 4.417 | 1 | 3 | 37 | NADP binding |
| GO:0015026 | 0.039 | 49.648 | 0 | 1 | 2 | coreceptor activity |
| GO:0031405 | 0.039 | 49.648 | 0 | 1 | 2 | lipoic acid binding |
| GO:0070410 | 0.039 | 49.648 | 0 | 1 | 2 | co-SMAD binding |
| GO:0005324 | 0.039 | 49.648 | 0 | 1 | 2 | long-chain fatty acid transporter activity |
| GO:0004097 | 0.039 | 49.648 | 0 | 1 | 2 | catechol oxidase activity |
| GO:0004044 | 0.039 | 49.648 | 0 | 1 | 2 | amidophosphoribosyltransferase activity |
| GO:0005219 | 0.039 | 49.648 | 0 | 1 | 2 | ryanodine-sensitive calcium-release channel activity |
| GO:0000213 | 0.039 | 49.648 | 0 | 1 | 2 | tRNA-intron endonuclease activity |
| GO:0030275 | 0.039 | 49.648 | 0 | 1 | 2 | LRR domain binding |
| GO:0015245 | 0.039 | 49.648 | 0 | 1 | 2 | fatty acid transporter activity |
| GO:0003726 | 0.039 | 49.648 | 0 | 1 | 2 | double-stranded RNA adenosine deaminase activity |
| GO:0003858 | 0.039 | 49.648 | 0 | 1 | 2 | 3-hydroxybutyrate dehydrogenase activity |
| GO:0036263 | 0.039 | 49.648 | 0 | 1 | 2 | L-DOPA monooxygenase activity |
| GO:0036264 | 0.039 | 49.648 | 0 | 1 | 2 | dopamine monooxygenase activity |
| GO:0046790 | 0.039 | 49.648 | 0 | 1 | 2 | virion binding |
| GO:0004711 | 0.039 | 49.648 | 0 | 1 | 2 | ribosomal protein S6 kinase activity |
| GO:0016716 | 0.039 | 49.648 | 0 | 1 | 2 | oxidoreductase activity, acting on paired donors, with incorporation or reduction of molecular oxygen, another compound as one donor, and incorporation of one atom of oxygen |
| GO:0017174 | 0.039 | 49.648 | 0 | 1 | 2 | glycine N-methyltransferase activity |
| GO:0004530 | 0.039 | 49.648 | 0 | 1 | 2 | deoxyribonuclease I activity |
| GO:0004503 | 0.039 | 49.648 | 0 | 1 | 2 | monophenol monooxygenase activity |
| GO:0005009 | 0.039 | 49.648 | 0 | 1 | 2 | insulin-activated receptor activity |
| GO:0016657 | 0.039 | 49.648 | 0 | 1 | 2 | oxidoreductase activity, acting on NADH or NADPH, nitrogenous group as acceptor |
| GO:0004672 | 0.040 | 1.808 | 8 | 13 | 381 | protein kinase activity |
| GO:0005539 | 0.040 | 3.292 | 1 | 4 | 65 | glycosaminoglycan binding |
| GO:0008289 | 0.041 | 1.846 | 7 | 12 | 344 | lipid binding |
| GO:0016616 | 0.041 | 2.299 | 3 | 7 | 161 | oxidoreductase activity, acting on the CH-OH group of donors, NAD or NADP as acceptor |
| GO:0003690 | 0.042 | 4.171 | 1 | 3 | 39 | double-stranded DNA binding |
| GO:0015485 | 0.044 | 6.649 | 0 | 2 | 17 | cholesterol binding |
| GO:0048037 | 0.047 | 1.919 | 5 | 10 | 275 | cofactor binding |
| GO:0043169 | 0.047 | 1.327 | 57 | 68 | 2887 | cation binding |
| GO:0043167 | 0.048 | 1.326 | 57 | 68 | 2888 | ion binding |
| GO:0046914 | 0.048 | 1.399 | 29 | 38 | 1474 | transition metal ion binding |
| GO:0004713 | 0.052 | 2.593 | 2 | 5 | 102 | protein tyrosine kinase activity |
| GO:0008138 | 0.054 | 5.866 | 0 | 2 | 19 | protein tyrosine/serine/threonine phosphatase activity |
| GO:0004521 | 0.055 | 2.951 | 1 | 4 | 72 | endoribonuclease activity |
| GO:0008061 | 0.056 | 3.660 | 1 | 3 | 44 | chitin binding |
| GO:0008134 | 0.057 | 2.908 | 1 | 4 | 73 | transcription factor binding |
| GO:0001129 | 0.058 | 24.821 | 0 | 1 | 3 | TBP-class protein binding RNA polymerase II transcription factor activity involved in preinitiation complex assembly |
| GO:0001132 | 0.058 | 24.821 | 0 | 1 | 3 | TBP-class protein binding RNA polymerase II transcription factor activity |
| GO:0001102 | 0.058 | 24.821 | 0 | 1 | 3 | RNA polymerase II activating transcription factor binding |
| GO:0070412 | 0.058 | 24.821 | 0 | 1 | 3 | R-SMAD binding |
| GO:0005159 | 0.058 | 24.821 | 0 | 1 | 3 | insulin-like growth factor receptor binding |
| GO:0070883 | 0.058 | 24.821 | 0 | 1 | 3 | pre-miRNA binding |
| GO:0017017 | 0.058 | 24.821 | 0 | 1 | 3 | MAP kinase tyrosine/serine/threonine phosphatase activity |
| GO:0004420 | 0.058 | 24.821 | 0 | 1 | 3 | hydroxymethylglutaryl-CoA reductase (NADPH) activity |
| GO:0016443 | 0.058 | 24.821 | 0 | 1 | 3 | bidentate ribonuclease III activity |
| GO:0016174 | 0.058 | 24.821 | 0 | 1 | 3 | NAD(P)H oxidase activity |
| GO:0001083 | 0.058 | 24.821 | 0 | 1 | 3 | RNA polymerase II basal transcription factor binding transcription factor activity |
| GO:0004772 | 0.058 | 24.821 | 0 | 1 | 3 | sterol O-acyltransferase activity |
| GO:0043560 | 0.058 | 24.821 | 0 | 1 | 3 | insulin receptor substrate binding |
| GO:0043548 | 0.058 | 24.821 | 0 | 1 | 3 | phosphatidylinositol 3-kinase binding |
| GO:0034736 | 0.058 | 24.821 | 0 | 1 | 3 | cholesterol O-acyltransferase activity |
| GO:0005010 | 0.058 | 24.821 | 0 | 1 | 3 | insulin-like growth factor-activated receptor activity |
| GO:0042043 | 0.058 | 24.821 | 0 | 1 | 3 | neurexin family protein binding |
| GO:0019838 | 0.059 | 5.539 | 0 | 2 | 20 | growth factor binding |
| GO:0042802 | 0.064 | 2.066 | 4 | 7 | 178 | identical protein binding |
| GO:0003729 | 0.064 | 2.785 | 2 | 4 | 76 | mRNA binding |
| GO:0004344 | 0.064 | 5.247 | 0 | 2 | 21 | glucose dehydrogenase activity |
| GO:0016651 | 0.066 | 3.409 | 1 | 3 | 47 | oxidoreductase activity, acting on NADH or NADPH |
| GO:0046872 | 0.067 | 1.293 | 55 | 65 | 2792 | metal ion binding |
| GO:0019198 | 0.070 | 4.984 | 0 | 2 | 22 | transmembrane receptor protein phosphatase activity |
| GO:0004715 | 0.070 | 4.984 | 0 | 2 | 22 | non-membrane spanning protein tyrosine kinase activity |
| GO:0005001 | 0.070 | 4.984 | 0 | 2 | 22 | transmembrane receptor protein tyrosine phosphatase activity |
| GO:0008812 | 0.076 | 4.746 | 0 | 2 | 23 | choline dehydrogenase activity |
| GO:0004721 | 0.076 | 2.304 | 2 | 5 | 114 | phosphoprotein phosphatase activity |
| GO:0003956 | 0.077 | 16.545 | 0 | 1 | 4 | NAD(P)+-protein-arginine ADP-ribosyltransferase activity |
| GO:0047522 | 0.077 | 16.545 | 0 | 1 | 4 | 15-oxoprostaglandin 13-oxidase activity |
| GO:0050327 | 0.077 | 16.545 | 0 | 1 | 4 | testosterone 17-beta-dehydrogenase (NAD+) activity |
| GO:0043014 | 0.077 | 16.545 | 0 | 1 | 4 | alpha-tubulin binding |
| GO:0004067 | 0.077 | 16.545 | 0 | 1 | 4 | asparaginase activity |
| GO:0032440 | 0.077 | 16.545 | 0 | 1 | 4 | 2-alkenal reductase [NAD(P)] activity |
| GO:0005178 | 0.077 | 16.545 | 0 | 1 | 4 | integrin binding |
| GO:0047035 | 0.077 | 16.545 | 0 | 1 | 4 | testosterone dehydrogenase (NAD+) activity |
| GO:0035174 | 0.077 | 16.545 | 0 | 1 | 4 | histone serine kinase activity |
| GO:0004303 | 0.077 | 16.545 | 0 | 1 | 4 | estradiol 17-beta-dehydrogenase activity |
| GO:0048027 | 0.077 | 16.545 | 0 | 1 | 4 | mRNA 5'-UTR binding |
| GO:0036132 | 0.077 | 16.545 | 0 | 1 | 4 | 13-prostaglandin reductase activity |
| GO:0034185 | 0.077 | 16.545 | 0 | 1 | 4 | apolipoprotein binding |
| GO:0043559 | 0.077 | 16.545 | 0 | 1 | 4 | insulin binding |
| GO:0017169 | 0.077 | 16.545 | 0 | 1 | 4 | CDP-alcohol phosphatidyltransferase activity |
| GO:0033549 | 0.077 | 16.545 | 0 | 1 | 4 | MAP kinase phosphatase activity |
| GO:0019789 | 0.077 | 16.545 | 0 | 1 | 4 | SUMO ligase activity |
| GO:0033613 | 0.077 | 16.545 | 0 | 1 | 4 | activating transcription factor binding |
| GO:0008294 | 0.077 | 16.545 | 0 | 1 | 4 | calcium- and calmodulin-responsive adenylate cyclase activity |
| GO:0005047 | 0.077 | 16.545 | 0 | 1 | 4 | signal recognition particle binding |
| GO:0008026 | 0.088 | 2.201 | 2 | 5 | 119 | ATP-dependent helicase activity |
| GO:0070035 | 0.088 | 2.201 | 2 | 5 | 119 | purine NTP-dependent helicase activity |
| GO:0031491 | 0.095 | 12.407 | 0 | 1 | 5 | nucleosome binding |
| GO:0047617 | 0.095 | 12.407 | 0 | 1 | 5 | acyl-CoA hydrolase activity |
| GO:0000340 | 0.095 | 12.407 | 0 | 1 | 5 | RNA 7-methylguanosine cap binding |
| GO:0070290 | 0.095 | 12.407 | 0 | 1 | 5 | NAPE-specific phospholipase D activity |
| GO:0000403 | 0.095 | 12.407 | 0 | 1 | 5 | Y-form DNA binding |
| GO:0051635 | 0.095 | 12.407 | 0 | 1 | 5 | bacterial cell surface binding |
| GO:0050750 | 0.095 | 12.407 | 0 | 1 | 5 | low-density lipoprotein particle receptor binding |
| GO:0050840 | 0.095 | 12.407 | 0 | 1 | 5 | extracellular matrix binding |
| GO:0035242 | 0.095 | 12.407 | 0 | 1 | 5 | protein-arginine omega-N asymmetric methyltransferase activity |
| GO:0043176 | 0.095 | 12.407 | 0 | 1 | 5 | amine binding |
| GO:0050664 | 0.095 | 12.407 | 0 | 1 | 5 | oxidoreductase activity, acting on NADH or NADPH, oxygen as acceptor |
| GO:0016727 | 0.095 | 12.407 | 0 | 1 | 5 | oxidoreductase activity, acting on CH or CH2 groups, oxygen as acceptor |
| GO:0004855 | 0.095 | 12.407 | 0 | 1 | 5 | xanthine oxidase activity |
| GO:0048487 | 0.095 | 12.407 | 0 | 1 | 5 | beta-tubulin binding |
| GO:0016594 | 0.095 | 12.407 | 0 | 1 | 5 | glycine binding |
| GO:0004579 | 0.095 | 12.407 | 0 | 1 | 5 | dolichyl-diphosphooligosaccharide-protein glycotransferase activity |
| GO:0005041 | 0.095 | 12.407 | 0 | 1 | 5 | low-density lipoprotein receptor activity |
| GO:0004630 | 0.095 | 12.407 | 0 | 1 | 5 | phospholipase D activity |
| GO:0016682 | 0.095 | 12.407 | 0 | 1 | 5 | oxidoreductase activity, acting on diphenols and related substances as donors, oxygen as acceptor |
| GO:0016891 | 0.104 | 2.774 | 1 | 3 | 57 | endoribonuclease activity, producing 5'-phosphomonoesters |
| GO:0005102 | 0.111 | 2.038 | 3 | 5 | 128 | receptor binding |
| GO:0016763 | 0.112 | 3.689 | 1 | 2 | 29 | transferase activity, transferring pentosyl groups |
| GO:0030060 | 0.113 | 9.925 | 0 | 1 | 6 | L-malate dehydrogenase activity |
| GO:0001105 | 0.113 | 9.925 | 0 | 1 | 6 | RNA polymerase II transcription coactivator activity |
| GO:0000400 | 0.113 | 9.925 | 0 | 1 | 6 | four-way junction DNA binding |
| GO:0005218 | 0.113 | 9.925 | 0 | 1 | 6 | intracellular ligand-gated calcium channel activity |
| GO:0035241 | 0.113 | 9.925 | 0 | 1 | 6 | protein-arginine omega-N monomethyltransferase activity |
| GO:0001085 | 0.113 | 9.925 | 0 | 1 | 6 | RNA polymerase II transcription factor binding |
| GO:0008469 | 0.113 | 9.925 | 0 | 1 | 6 | histone-arginine N-methyltransferase activity |
| GO:0034237 | 0.113 | 9.925 | 0 | 1 | 6 | protein kinase A regulatory subunit binding |
| GO:0050997 | 0.113 | 9.925 | 0 | 1 | 6 | quaternary ammonium group binding |
| GO:0016857 | 0.113 | 9.925 | 0 | 1 | 6 | racemase and epimerase activity, acting on carbohydrates and derivatives |
| GO:0016888 | 0.113 | 9.925 | 0 | 1 | 6 | endodeoxyribonuclease activity, producing 5'-phosphomonoesters |
| GO:0004576 | 0.113 | 9.925 | 0 | 1 | 6 | oligosaccharyl transferase activity |
| GO:0042803 | 0.115 | 1.871 | 3 | 6 | 167 | protein homodimerization activity |
| GO:0016893 | 0.121 | 2.582 | 1 | 3 | 61 | endonuclease activity, active with either ribo- or deoxyribonucleic acids and producing 5'-phosphomonoesters |
| GO:0019901 | 0.126 | 3.433 | 1 | 2 | 31 | protein kinase binding |
| GO:0004568 | 0.126 | 3.433 | 1 | 2 | 31 | chitinase activity |
| GO:0005543 | 0.126 | 1.657 | 5 | 8 | 251 | phospholipid binding |
| GO:0070325 | 0.131 | 8.270 | 0 | 1 | 7 | lipoprotein particle receptor binding |
| GO:0046969 | 0.131 | 8.270 | 0 | 1 | 7 | NAD-dependent histone deacetylase activity (H3-K9 specific) |
| GO:0046970 | 0.131 | 8.270 | 0 | 1 | 7 | NAD-dependent histone deacetylase activity (H4-K16 specific) |
| GO:0046975 | 0.131 | 8.270 | 0 | 1 | 7 | histone methyltransferase activity (H3-K36 specific) |
| GO:0000339 | 0.131 | 8.270 | 0 | 1 | 7 | RNA cap binding |
| GO:0004000 | 0.131 | 8.270 | 0 | 1 | 7 | adenosine deaminase activity |
| GO:0032129 | 0.131 | 8.270 | 0 | 1 | 7 | histone deacetylase activity (H3-K9 specific) |
| GO:0034041 | 0.131 | 8.270 | 0 | 1 | 7 | sterol-transporting ATPase activity |
| GO:0042799 | 0.131 | 8.270 | 0 | 1 | 7 | histone methyltransferase activity (H4-K20 specific) |
| GO:0042813 | 0.131 | 8.270 | 0 | 1 | 7 | Wnt-activated receptor activity |
| GO:0005542 | 0.131 | 8.270 | 0 | 1 | 7 | folic acid binding |
| GO:0016289 | 0.131 | 8.270 | 0 | 1 | 7 | CoA hydrolase activity |
| GO:0008312 | 0.131 | 8.270 | 0 | 1 | 7 | 7S RNA binding |
| GO:0008453 | 0.131 | 8.270 | 0 | 1 | 7 | alanine-glyoxylate transaminase activity |
| GO:0031078 | 0.131 | 8.270 | 0 | 1 | 7 | histone deacetylase activity (H3-K14 specific) |
| GO:0016726 | 0.131 | 8.270 | 0 | 1 | 7 | oxidoreductase activity, acting on CH or CH2 groups, NAD or NADP as acceptor |
| GO:0043546 | 0.131 | 8.270 | 0 | 1 | 7 | molybdopterin cofactor binding |
| GO:0004854 | 0.131 | 8.270 | 0 | 1 | 7 | xanthine dehydrogenase activity |
| GO:0016892 | 0.131 | 8.270 | 0 | 1 | 7 | endoribonuclease activity, producing 3'-phosphomonoesters |
| GO:0034739 | 0.131 | 8.270 | 0 | 1 | 7 | histone deacetylase activity (H4-K16 specific) |
| GO:0032041 | 0.131 | 8.270 | 0 | 1 | 7 | NAD-dependent histone deacetylase activity (H3-K14 specific) |
| GO:0003697 | 0.132 | 3.318 | 1 | 2 | 32 | single-stranded DNA binding |
| GO:0016702 | 0.132 | 3.318 | 1 | 2 | 32 | oxidoreductase activity, acting on single donors with incorporation of molecular oxygen, incorporation of two atoms of oxygen |
| GO:0016740 | 0.138 | 1.263 | 28 | 34 | 1423 | transferase activity |
| GO:0003727 | 0.139 | 3.211 | 1 | 2 | 33 | single-stranded RNA binding |
| GO:0004540 | 0.141 | 2.061 | 2 | 4 | 101 | ribonuclease activity |
| GO:0051213 | 0.146 | 3.110 | 1 | 2 | 34 | dioxygenase activity |
| GO:0001190 | 0.148 | 7.087 | 0 | 1 | 8 | RNA polymerase II transcription factor binding transcription factor activity involved in positive regulation of transcription |
| GO:0034979 | 0.148 | 7.087 | 0 | 1 | 8 | NAD-dependent protein deacetylase activity |
| GO:0035173 | 0.148 | 7.087 | 0 | 1 | 8 | histone kinase activity |
| GO:0015278 | 0.148 | 7.087 | 0 | 1 | 8 | calcium-release channel activity |
| GO:0004470 | 0.148 | 7.087 | 0 | 1 | 8 | malic enzyme activity |
| GO:0004407 | 0.148 | 7.087 | 0 | 1 | 8 | histone deacetylase activity |
| GO:0016418 | 0.148 | 7.087 | 0 | 1 | 8 | S-acetyltransferase activity |
| GO:0005544 | 0.148 | 7.087 | 0 | 1 | 8 | calcium-dependent phospholipid binding |
| GO:0033764 | 0.148 | 7.087 | 0 | 1 | 8 | steroid dehydrogenase activity, acting on the CH-OH group of donors, NAD or NADP as acceptor |
| GO:0017136 | 0.148 | 7.087 | 0 | 1 | 8 | NAD-dependent histone deacetylase activity |
| GO:0016791 | 0.154 | 1.708 | 4 | 6 | 182 | phosphatase activity |
| GO:0004519 | 0.154 | 1.708 | 4 | 6 | 182 | endonuclease activity |
| GO:0016773 | 0.156 | 1.409 | 10 | 13 | 479 | phosphotransferase activity, alcohol group as acceptor |
| GO:0005509 | 0.158 | 1.452 | 8 | 11 | 393 | calcium ion binding |
| GO:0004842 | 0.159 | 1.689 | 4 | 6 | 184 | ubiquitin-protein ligase activity |
| GO:0003723 | 0.164 | 1.306 | 16 | 20 | 798 | RNA binding |
| GO:0016679 | 0.165 | 6.201 | 0 | 1 | 9 | oxidoreductase activity, acting on diphenols and related substances as donors |
| GO:0034040 | 0.165 | 6.201 | 0 | 1 | 9 | lipid-transporting ATPase activity |
| GO:0005217 | 0.165 | 6.201 | 0 | 1 | 9 | intracellular ligand-gated ion channel activity |
| GO:0000217 | 0.165 | 6.201 | 0 | 1 | 9 | DNA secondary structure binding |
| GO:0031625 | 0.165 | 6.201 | 0 | 1 | 9 | ubiquitin protein ligase binding |
| GO:0044389 | 0.165 | 6.201 | 0 | 1 | 9 | small conjugating protein ligase binding |
| GO:0050681 | 0.165 | 6.201 | 0 | 1 | 9 | androgen receptor binding |
| GO:0016229 | 0.165 | 6.201 | 0 | 1 | 9 | steroid dehydrogenase activity |
| GO:0033558 | 0.165 | 6.201 | 0 | 1 | 9 | protein deacetylase activity |
| GO:0016854 | 0.165 | 6.201 | 0 | 1 | 9 | racemase and epimerase activity |
| GO:0017105 | 0.165 | 6.201 | 0 | 1 | 9 | acyl-CoA delta11-desaturase activity |
| GO:0038024 | 0.167 | 2.843 | 1 | 2 | 37 | cargo receptor activity |
| GO:0043021 | 0.174 | 2.763 | 1 | 2 | 38 | ribonucleoprotein complex binding |
| GO:0019900 | 0.174 | 2.763 | 1 | 2 | 38 | kinase binding |
| GO:0019787 | 0.180 | 1.623 | 4 | 6 | 191 | small conjugating protein ligase activity |
| GO:0004029 | 0.182 | 5.511 | 0 | 1 | 10 | aldehyde dehydrogenase (NAD) activity |
| GO:0030371 | 0.182 | 5.511 | 0 | 1 | 10 | translation repressor activity |
| GO:0003756 | 0.182 | 5.511 | 0 | 1 | 10 | protein disulfide isomerase activity |
| GO:0034437 | 0.182 | 5.511 | 0 | 1 | 10 | glycoprotein transporter activity |
| GO:0016273 | 0.182 | 5.511 | 0 | 1 | 10 | arginine N-methyltransferase activity |
| GO:0016274 | 0.182 | 5.511 | 0 | 1 | 10 | protein-arginine N-methyltransferase activity |
| GO:0008301 | 0.182 | 5.511 | 0 | 1 | 10 | DNA binding, bending |
| GO:0016780 | 0.182 | 5.511 | 0 | 1 | 10 | phosphotransferase activity, for other substituted phosphate groups |
| GO:0016894 | 0.182 | 5.511 | 0 | 1 | 10 | endonuclease activity, active with either ribo- or deoxyribonucleic acids and producing 3'-phosphomonoesters |
| GO:0016862 | 0.182 | 5.511 | 0 | 1 | 10 | intramolecular oxidoreductase activity, interconverting keto- and enol-groups |
| GO:0016864 | 0.182 | 5.511 | 0 | 1 | 10 | intramolecular oxidoreductase activity, transposing S-S bonds |
| GO:0016846 | 0.182 | 5.511 | 0 | 1 | 10 | carbon-sulfur lyase activity |
| GO:0004549 | 0.182 | 5.511 | 0 | 1 | 10 | tRNA-specific ribonuclease activity |
| GO:0004500 | 0.182 | 5.511 | 0 | 1 | 10 | dopamine beta-monooxygenase activity |
| GO:0004674 | 0.186 | 1.492 | 6 | 8 | 277 | protein serine/threonine kinase activity |
| GO:0042578 | 0.186 | 1.538 | 5 | 7 | 235 | phosphoric ester hydrolase activity |
| GO:0003676 | 0.192 | 1.181 | 43 | 48 | 2149 | nucleic acid binding |
| GO:0005057 | 0.195 | 2.550 | 1 | 2 | 41 | receptor signaling protein activity |
| GO:0008235 | 0.195 | 2.550 | 1 | 2 | 41 | metalloexopeptidase activity |
| GO:0016301 | 0.197 | 1.339 | 10 | 13 | 502 | kinase activity |
| GO:0043498 | 0.198 | 4.959 | 0 | 1 | 11 | cell surface binding |
| GO:0019210 | 0.198 | 4.959 | 0 | 1 | 11 | kinase inhibitor activity |
| GO:0016715 | 0.198 | 4.959 | 0 | 1 | 11 | oxidoreductase activity, acting on paired donors, with incorporation or reduction of molecular oxygen, reduced ascorbate as one donor, and incorporation of one atom of oxygen |
| GO:0017127 | 0.198 | 4.959 | 0 | 1 | 11 | cholesterol transporter activity |
| GO:0004563 | 0.198 | 4.959 | 0 | 1 | 11 | beta-N-acetylhexosaminidase activity |
| GO:0000030 | 0.198 | 4.959 | 0 | 1 | 11 | mannosyltransferase activity |
| GO:0005200 | 0.203 | 2.486 | 1 | 2 | 42 | structural constituent of cytoskeleton |
| GO:0005484 | 0.214 | 4.508 | 0 | 1 | 12 | SNAP receptor activity |
| GO:0046332 | 0.214 | 4.508 | 0 | 1 | 12 | SMAD binding |
| GO:0015248 | 0.214 | 4.508 | 0 | 1 | 12 | sterol transporter activity |
| GO:0051010 | 0.214 | 4.508 | 0 | 1 | 12 | microtubule plus-end binding |
| GO:0051018 | 0.214 | 4.508 | 0 | 1 | 12 | protein kinase A binding |
| GO:0005546 | 0.214 | 4.508 | 0 | 1 | 12 | phosphatidylinositol-4,5-bisphosphate binding |
| GO:0051540 | 0.228 | 1.867 | 2 | 3 | 83 | metal cluster binding |
| GO:0051536 | 0.228 | 1.867 | 2 | 3 | 83 | iron-sulfur cluster binding |
| GO:0046983 | 0.229 | 1.491 | 4 | 6 | 207 | protein dimerization activity |
| GO:0008553 | 0.230 | 4.132 | 0 | 1 | 13 | hydrogen-exporting ATPase activity, phosphorylative mechanism |
| GO:0046961 | 0.230 | 4.132 | 0 | 1 | 13 | proton-transporting ATPase activity, rotational mechanism |
| GO:0004016 | 0.230 | 4.132 | 0 | 1 | 13 | adenylate cyclase activity |
| GO:0043178 | 0.230 | 4.132 | 0 | 1 | 13 | alcohol binding |
| GO:0004181 | 0.230 | 4.132 | 0 | 1 | 13 | metallocarboxypeptidase activity |
| GO:0005545 | 0.230 | 4.132 | 0 | 1 | 13 | 1-phosphatidylinositol binding |
| GO:0016755 | 0.230 | 4.132 | 0 | 1 | 13 | transferase activity, transferring amino-acyl groups |
| GO:0019213 | 0.230 | 4.132 | 0 | 1 | 13 | deacetylase activity |
| GO:0016725 | 0.230 | 4.132 | 0 | 1 | 13 | oxidoreductase activity, acting on CH or CH2 groups |
| GO:0019239 | 0.230 | 4.132 | 0 | 1 | 13 | deaminase activity |
| GO:0043531 | 0.230 | 4.132 | 0 | 1 | 13 | ADP binding |
| GO:0005021 | 0.230 | 4.132 | 0 | 1 | 13 | vascular endothelial growth factor-activated receptor activity |
| GO:0008762 | 0.230 | 4.132 | 0 | 1 | 13 | UDP-N-acetylmuramate dehydrogenase activity |
| GO:0017046 | 0.245 | 3.813 | 0 | 1 | 14 | peptide hormone binding |
| GO:0035258 | 0.245 | 3.813 | 0 | 1 | 14 | steroid hormone receptor binding |
| GO:0016417 | 0.245 | 3.813 | 0 | 1 | 14 | S-acyltransferase activity |
| GO:0017112 | 0.245 | 3.813 | 0 | 1 | 14 | Rab guanyl-nucleotide exchange factor activity |
| GO:0004520 | 0.245 | 3.813 | 0 | 1 | 14 | endodeoxyribonuclease activity |
| GO:0016881 | 0.246 | 1.454 | 4 | 6 | 212 | acid-amino acid ligase activity |
| GO:0005085 | 0.258 | 1.581 | 3 | 4 | 130 | guanyl-nucleotide exchange factor activity |
| GO:0015459 | 0.260 | 3.541 | 0 | 1 | 15 | potassium channel regulator activity |
| GO:0016215 | 0.260 | 3.541 | 0 | 1 | 15 | acyl-CoA desaturase activity |
| GO:0004768 | 0.260 | 3.541 | 0 | 1 | 15 | stearoyl-CoA 9-desaturase activity |
| GO:0004745 | 0.260 | 3.541 | 0 | 1 | 15 | retinol dehydrogenase activity |
| GO:0000062 | 0.260 | 3.541 | 0 | 1 | 15 | fatty-acyl-CoA binding |
| GO:0030695 | 0.265 | 1.371 | 5 | 7 | 262 | GTPase regulator activity |
| GO:0035091 | 0.270 | 1.695 | 2 | 3 | 91 | phosphatidylinositol binding |
| GO:0016491 | 0.274 | 1.191 | 16 | 19 | 821 | oxidoreductase activity |
| GO:0060589 | 0.274 | 1.354 | 5 | 7 | 265 | nucleoside-triphosphatase regulator activity |
| GO:0051537 | 0.275 | 3.304 | 0 | 1 | 16 | 2 iron, 2 sulfur cluster binding |
| GO:0045735 | 0.275 | 3.304 | 0 | 1 | 16 | nutrient reservoir activity |
| GO:0001076 | 0.276 | 1.986 | 1 | 2 | 52 | RNA polymerase II transcription factor binding transcription factor activity |
| GO:0004714 | 0.284 | 1.947 | 1 | 2 | 53 | transmembrane receptor protein tyrosine kinase activity |
| GO:0016615 | 0.289 | 3.097 | 0 | 1 | 17 | malate dehydrogenase activity |
| GO:0030374 | 0.289 | 3.097 | 0 | 1 | 17 | ligand-dependent nuclear receptor transcription coactivator activity |
| GO:0030228 | 0.289 | 3.097 | 0 | 1 | 17 | lipoprotein particle receptor activity |
| GO:0004712 | 0.289 | 3.097 | 0 | 1 | 17 | protein serine/threonine/tyrosine kinase activity |
| GO:0016717 | 0.289 | 3.097 | 0 | 1 | 17 | oxidoreductase activity, acting on paired donors, with oxidation of a pair of donors resulting in the reduction of molecular oxygen to two molecules of water |
| GO:0016814 | 0.289 | 3.097 | 0 | 1 | 17 | hydrolase activity, acting on carbon-nitrogen (but not peptide) bonds, in cyclic amidines |
| GO:0003993 | 0.289 | 3.097 | 0 | 1 | 17 | acid phosphatase activity |
| GO:0016788 | 0.298 | 1.181 | 15 | 17 | 739 | hydrolase activity, acting on ester bonds |
| GO:0050136 | 0.303 | 2.915 | 0 | 1 | 18 | NADH dehydrogenase (quinone) activity |
| GO:0003730 | 0.303 | 2.915 | 0 | 1 | 18 | mRNA 3'-UTR binding |
| GO:0008137 | 0.303 | 2.915 | 0 | 1 | 18 | NADH dehydrogenase (ubiquinone) activity |
| GO:0004623 | 0.303 | 2.915 | 0 | 1 | 18 | phospholipase A2 activity |
| GO:0004620 | 0.306 | 1.838 | 1 | 2 | 56 | phospholipase activity |
| GO:0050660 | 0.314 | 1.552 | 2 | 3 | 99 | flavin adenine dinucleotide binding |
| GO:0003954 | 0.317 | 2.752 | 0 | 1 | 19 | NADH dehydrogenase activity |
| GO:0042562 | 0.317 | 2.752 | 0 | 1 | 19 | hormone binding |
| GO:0004536 | 0.317 | 2.752 | 0 | 1 | 19 | deoxyribonuclease activity |
| GO:0019199 | 0.328 | 1.741 | 1 | 2 | 59 | transmembrane receptor protein kinase activity |
| GO:0015929 | 0.331 | 2.607 | 0 | 1 | 20 | hexosaminidase activity |
| GO:0004004 | 0.331 | 2.607 | 0 | 1 | 20 | ATP-dependent RNA helicase activity |
| GO:0019829 | 0.331 | 2.607 | 0 | 1 | 20 | cation-transporting ATPase activity |
| GO:0008565 | 0.335 | 1.710 | 1 | 2 | 60 | protein transporter activity |
| GO:0051539 | 0.335 | 1.710 | 1 | 2 | 60 | 4 iron, 4 sulfur cluster binding |
| GO:0004386 | 0.342 | 1.315 | 4 | 5 | 194 | helicase activity |
| GO:0004177 | 0.343 | 1.681 | 1 | 2 | 61 | aminopeptidase activity |
| GO:0016701 | 0.343 | 1.681 | 1 | 2 | 61 | oxidoreductase activity, acting on single donors with incorporation of molecular oxygen |
| GO:0016628 | 0.344 | 2.477 | 0 | 1 | 21 | oxidoreductase activity, acting on the CH-CH group of donors, NAD or NADP as acceptor |
| GO:0005548 | 0.344 | 2.477 | 0 | 1 | 21 | phospholipid transporter activity |
| GO:0008186 | 0.344 | 2.477 | 0 | 1 | 21 | RNA-dependent ATPase activity |
| GO:0005086 | 0.344 | 2.477 | 0 | 1 | 21 | ARF guanyl-nucleotide exchange factor activity |
| GO:0005044 | 0.344 | 2.477 | 0 | 1 | 21 | scavenger receptor activity |
| GO:0016655 | 0.344 | 2.477 | 0 | 1 | 21 | oxidoreductase activity, acting on NADH or NADPH, quinone or similar compound as acceptor |
| GO:0019902 | 0.357 | 2.358 | 0 | 1 | 22 | phosphatase binding |
| GO:0035257 | 0.357 | 2.358 | 0 | 1 | 22 | nuclear hormone receptor binding |
| GO:0051427 | 0.357 | 2.358 | 0 | 1 | 22 | hormone receptor binding |
| GO:0032947 | 0.357 | 2.358 | 0 | 1 | 22 | protein complex scaffold |
| GO:0003677 | 0.368 | 1.095 | 25 | 27 | 1262 | DNA binding |
| GO:0004033 | 0.370 | 2.251 | 0 | 1 | 23 | aldo-keto reductase (NADP) activity |
| GO:0004003 | 0.370 | 2.251 | 0 | 1 | 23 | ATP-dependent DNA helicase activity |
| GO:0016860 | 0.370 | 2.251 | 0 | 1 | 23 | intramolecular oxidoreductase activity |
| GO:0019899 | 0.371 | 1.315 | 3 | 4 | 155 | enzyme binding |
| GO:0004601 | 0.383 | 2.153 | 0 | 1 | 24 | peroxidase activity |
| GO:0015035 | 0.383 | 2.153 | 0 | 1 | 24 | protein disulfide oxidoreductase activity |
| GO:0043022 | 0.383 | 2.153 | 0 | 1 | 24 | ribosome binding |
| GO:0016706 | 0.383 | 2.153 | 0 | 1 | 24 | oxidoreductase activity, acting on paired donors, with incorporation or reduction of molecular oxygen, 2-oxoglutarate as one donor, and incorporation of one atom each of oxygen into both donors |
| GO:0000049 | 0.383 | 2.153 | 0 | 1 | 24 | tRNA binding |
| GO:0016684 | 0.383 | 2.153 | 0 | 1 | 24 | oxidoreductase activity, acting on peroxide as acceptor |
| GO:0016790 | 0.386 | 1.525 | 1 | 2 | 67 | thiolester hydrolase activity |
| GO:0016879 | 0.388 | 1.206 | 5 | 6 | 253 | ligase activity, forming carbon-nitrogen bonds |
| GO:0031406 | 0.393 | 1.502 | 1 | 2 | 68 | carboxylic acid binding |
| GO:0008238 | 0.401 | 1.328 | 2 | 3 | 115 | exopeptidase activity |
| GO:0016757 | 0.407 | 1.248 | 3 | 4 | 163 | transferase activity, transferring glycosyl groups |
| GO:0016772 | 0.420 | 1.081 | 16 | 17 | 799 | transferase activity, transferring phosphorus-containing groups |
| GO:0009975 | 0.430 | 1.833 | 1 | 1 | 28 | cyclase activity |
| GO:0016769 | 0.430 | 1.833 | 1 | 1 | 28 | transferase activity, transferring nitrogenous groups |
| GO:0008483 | 0.430 | 1.833 | 1 | 1 | 28 | transaminase activity |
| GO:0016849 | 0.430 | 1.833 | 1 | 1 | 28 | phosphorus-oxygen lyase activity |
| GO:0042623 | 0.433 | 1.118 | 7 | 8 | 363 | ATPase activity, coupled |
| GO:0005088 | 0.434 | 1.375 | 1 | 2 | 74 | Ras guanyl-nucleotide exchange factor activity |
| GO:0008374 | 0.442 | 1.767 | 1 | 1 | 29 | O-acyltransferase activity |
| GO:0016247 | 0.442 | 1.767 | 1 | 1 | 29 | channel regulator activity |
| GO:0005198 | 0.446 | 1.105 | 7 | 8 | 367 | structural molecule activity |
| GO:0016298 | 0.448 | 1.338 | 2 | 2 | 76 | lipase activity |
| GO:0004518 | 0.449 | 1.126 | 5 | 6 | 270 | nuclease activity |
| GO:0004843 | 0.453 | 1.706 | 1 | 1 | 30 | ubiquitin-specific protease activity |
| GO:0004553 | 0.460 | 1.158 | 3 | 4 | 175 | hydrolase activity, hydrolyzing O-glycosyl compounds |
| GO:0015036 | 0.464 | 1.649 | 1 | 1 | 31 | disulfide oxidoreductase activity |
| GO:0043130 | 0.464 | 1.649 | 1 | 1 | 31 | ubiquitin binding |
| GO:0003724 | 0.464 | 1.649 | 1 | 1 | 31 | RNA helicase activity |
| GO:0043565 | 0.470 | 1.100 | 5 | 6 | 276 | sequence-specific DNA binding |
| GO:0003824 | 0.474 | 1.024 | 93 | 94 | 4686 | catalytic activity |
| GO:0051015 | 0.474 | 1.596 | 1 | 1 | 32 | actin filament binding |
| GO:0019783 | 0.474 | 1.596 | 1 | 1 | 32 | small conjugating protein-specific protease activity |
| GO:0005083 | 0.478 | 1.131 | 4 | 4 | 179 | small GTPase regulator activity |
| GO:0044212 | 0.495 | 1.141 | 3 | 3 | 133 | transcription regulatory region DNA binding |
| GO:0001077 | 0.495 | 1.499 | 1 | 1 | 34 | RNA polymerase II core promoter proximal region sequence-specific DNA binding transcription factor activity involved in positive regulation of transcription |
| GO:0005097 | 0.495 | 1.499 | 1 | 1 | 34 | Rab GTPase activator activity |
| GO:0004523 | 0.495 | 1.499 | 1 | 1 | 34 | ribonuclease H activity |
| GO:0001067 | 0.500 | 1.133 | 3 | 3 | 134 | regulatory region nucleic acid binding |
| GO:0000975 | 0.500 | 1.133 | 3 | 3 | 134 | regulatory region DNA binding |
| GO:0001228 | 0.505 | 1.454 | 1 | 1 | 35 | RNA polymerase II transcription regulatory region sequence-specific DNA binding transcription factor activity involved in positive regulation of transcription |
| GO:0003678 | 0.505 | 1.454 | 1 | 1 | 35 | DNA helicase activity |
| GO:0008237 | 0.512 | 1.081 | 4 | 4 | 187 | metallopeptidase activity |
| GO:0004872 | 0.522 | 1.025 | 9 | 9 | 443 | receptor activity |
| GO:0005254 | 0.525 | 1.373 | 1 | 1 | 37 | chloride channel activity |
| GO:0003705 | 0.525 | 1.373 | 1 | 1 | 37 | RNA polymerase II distal enhancer sequence-specific DNA binding transcription factor activity |
| GO:0016836 | 0.525 | 1.373 | 1 | 1 | 37 | hydro-lyase activity |
| GO:0004180 | 0.534 | 1.336 | 1 | 1 | 38 | carboxypeptidase activity |
| GO:0003899 | 0.534 | 1.336 | 1 | 1 | 38 | DNA-directed RNA polymerase activity |
| GO:0005506 | 0.535 | 1.025 | 6 | 6 | 295 | iron ion binding |
| GO:0004888 | 0.535 | 1.025 | 6 | 6 | 295 | transmembrane signaling receptor activity |
| GO:0016874 | 0.538 | 1.013 | 8 | 8 | 398 | ligase activity |
| GO:0016798 | 0.541 | 1.040 | 4 | 4 | 194 | hydrolase activity, acting on glycosyl bonds |
| GO:0005253 | 0.544 | 1.301 | 1 | 1 | 39 | anion channel activity |
| GO:0004871 | 0.544 | 1.005 | 9 | 9 | 451 | signal transducer activity |
| GO:0060089 | 0.544 | 1.005 | 9 | 9 | 451 | molecular transducer activity |
| GO:0036094 | 0.547 | 0.993 | 44 | 44 | 2227 | small molecule binding |
| GO:0032182 | 0.553 | 1.267 | 1 | 1 | 40 | small conjugating protein binding |
| GO:0016597 | 0.553 | 1.267 | 1 | 1 | 40 | amino acid binding |
| GO:0016209 | 0.562 | 1.235 | 1 | 1 | 41 | antioxidant activity |
| GO:0016829 | 0.567 | 1.021 | 3 | 3 | 148 | lyase activity |
| GO:0016705 | 0.572 | 0.991 | 5 | 5 | 254 | oxidoreductase activity, acting on paired donors, with incorporation or reduction of molecular oxygen |
| GO:0015108 | 0.579 | 1.176 | 1 | 1 | 43 | chloride transmembrane transporter activity |
| GO:0001104 | 0.587 | 1.149 | 1 | 1 | 44 | RNA polymerase II transcription cofactor activity |
| GO:0042302 | 0.587 | 1.149 | 1 | 1 | 44 | structural constituent of cuticle |
| GO:0000166 | 0.590 | 0.973 | 42 | 41 | 2106 | nucleotide binding |
| GO:0097159 | 0.590 | 0.973 | 42 | 41 | 2106 | organic cyclic compound binding |
| GO:1901265 | 0.590 | 0.973 | 42 | 41 | 2106 | nucleoside phosphate binding |
| GO:0042393 | 0.596 | 1.122 | 1 | 1 | 45 | histone binding |
| GO:0005244 | 0.600 | 0.997 | 2 | 2 | 101 | voltage-gated ion channel activity |
| GO:0022832 | 0.600 | 0.997 | 2 | 2 | 101 | voltage-gated channel activity |
| GO:0005524 | 0.605 | 0.963 | 29 | 28 | 1454 | ATP binding |
| GO:0019904 | 0.612 | 1.073 | 1 | 1 | 47 | protein domain specific binding |
| GO:0004221 | 0.612 | 1.073 | 1 | 1 | 47 | ubiquitin thiolesterase activity |
| GO:0080030 | 0.612 | 1.073 | 1 | 1 | 47 | methyl indole-3-acetate esterase activity |
| GO:0080031 | 0.612 | 1.073 | 1 | 1 | 47 | methyl salicylate esterase activity |
| GO:0080032 | 0.612 | 1.073 | 1 | 1 | 47 | methyl jasmonate esterase activity |
| GO:0004190 | 0.621 | 0.958 | 2 | 2 | 105 | aspartic-type endopeptidase activity |
| GO:0070001 | 0.621 | 0.958 | 2 | 2 | 105 | aspartic-type peptidase activity |
| GO:0016835 | 0.627 | 1.028 | 1 | 1 | 49 | carbon-oxygen lyase activity |
| GO:0032559 | 0.628 | 0.950 | 29 | 28 | 1469 | adenyl ribonucleotide binding |
| GO:0030554 | 0.628 | 0.950 | 29 | 28 | 1469 | adenyl nucleotide binding |
| GO:0019842 | 0.632 | 0.939 | 2 | 2 | 107 | vitamin binding |
| GO:0015662 | 0.634 | 1.007 | 1 | 1 | 50 | ATPase activity, coupled to transmembrane movement of ions, phosphorylative mechanism |
| GO:0016853 | 0.637 | 0.930 | 2 | 2 | 108 | isomerase activity |
| GO:0038023 | 0.637 | 0.919 | 6 | 6 | 327 | signaling receptor activity |
| GO:0022836 | 0.637 | 0.918 | 3 | 3 | 164 | gated channel activity |
| GO:0016627 | 0.642 | 0.987 | 1 | 1 | 51 | oxidoreductase activity, acting on the CH-CH group of donors |
| GO:0051287 | 0.642 | 0.987 | 1 | 1 | 51 | NAD binding |
| GO:0015078 | 0.642 | 0.987 | 1 | 1 | 51 | hydrogen ion transmembrane transporter activity |
| GO:0034062 | 0.642 | 0.987 | 1 | 1 | 51 | RNA polymerase activity |
| GO:0016746 | 0.645 | 0.907 | 3 | 3 | 166 | transferase activity, transferring acyl groups |
| GO:0030674 | 0.649 | 0.967 | 1 | 1 | 52 | protein binding, bridging |
| GO:0043492 | 0.649 | 0.901 | 3 | 3 | 167 | ATPase activity, coupled to movement of substances |
| GO:0042626 | 0.649 | 0.901 | 3 | 3 | 167 | ATPase activity, coupled to transmembrane movement of substances |
| GO:0008081 | 0.656 | 0.949 | 1 | 1 | 53 | phosphoric diester hydrolase activity |
| GO:0005089 | 0.656 | 0.949 | 1 | 1 | 53 | Rho guanyl-nucleotide exchange factor activity |
| GO:0016667 | 0.656 | 0.949 | 1 | 1 | 53 | oxidoreductase activity, acting on a sulfur group of donors |
| GO:0003743 | 0.663 | 0.931 | 1 | 1 | 54 | translation initiation factor activity |
| GO:0015399 | 0.665 | 0.879 | 3 | 3 | 171 | primary active transmembrane transporter activity |
| GO:0015405 | 0.665 | 0.879 | 3 | 3 | 171 | P-P-bond-hydrolysis-driven transmembrane transporter activity |
| GO:0016820 | 0.665 | 0.879 | 3 | 3 | 171 | hydrolase activity, acting on acid anhydrides, catalyzing transmembrane movement of substances |
| GO:0016620 | 0.669 | 0.913 | 1 | 1 | 55 | oxidoreductase activity, acting on the aldehyde or oxo group of donors, NAD or NADP as acceptor |
| GO:0019207 | 0.669 | 0.913 | 1 | 1 | 55 | kinase regulator activity |
| GO:0005099 | 0.669 | 0.913 | 1 | 1 | 55 | Ras GTPase activator activity |
| GO:0070279 | 0.676 | 0.897 | 1 | 1 | 56 | vitamin B6 binding |
| GO:0030170 | 0.676 | 0.897 | 1 | 1 | 56 | pyridoxal phosphate binding |
| GO:0032403 | 0.683 | 0.881 | 1 | 1 | 57 | protein complex binding |
| GO:0005262 | 0.683 | 0.881 | 1 | 1 | 57 | calcium channel activity |
| GO:0016765 | 0.683 | 0.881 | 1 | 1 | 57 | transferase activity, transferring alkyl or aryl (other than methyl) groups |
| GO:0060090 | 0.683 | 0.881 | 1 | 1 | 57 | binding, bridging |
| GO:0000981 | 0.684 | 0.849 | 2 | 2 | 118 | sequence-specific DNA binding RNA polymerase II transcription factor activity |
| GO:0042277 | 0.689 | 0.865 | 1 | 1 | 58 | peptide binding |
| GO:0008236 | 0.695 | 0.838 | 4 | 3 | 179 | serine-type peptidase activity |
| GO:0005267 | 0.701 | 0.835 | 1 | 1 | 60 | potassium channel activity |
| GO:0042625 | 0.701 | 0.835 | 1 | 1 | 60 | ATPase activity, coupled to transmembrane movement of ions |
| GO:0017171 | 0.702 | 0.829 | 4 | 3 | 181 | serine hydrolase activity |
| GO:0016407 | 0.707 | 0.821 | 1 | 1 | 61 | acetyltransferase activity |
| GO:0000982 | 0.707 | 0.821 | 1 | 1 | 61 | RNA polymerase II core promoter proximal region sequence-specific DNA binding transcription factor activity |
| GO:0004091 | 0.724 | 0.782 | 1 | 1 | 64 | carboxylesterase activity |
| GO:0050253 | 0.724 | 0.782 | 1 | 1 | 64 | retinyl-palmitate esterase activity |
| GO:0016758 | 0.727 | 0.781 | 3 | 2 | 128 | transferase activity, transferring hexosyl groups |
| GO:0015103 | 0.730 | 0.770 | 1 | 1 | 65 | inorganic anion transmembrane transporter activity |
| GO:0005096 | 0.731 | 0.774 | 3 | 2 | 129 | GTPase activator activity |
| GO:0016887 | 0.735 | 0.837 | 9 | 8 | 475 | ATPase activity |
| GO:0003887 | 0.735 | 0.758 | 1 | 1 | 66 | DNA-directed DNA polymerase activity |
| GO:0016747 | 0.739 | 0.762 | 3 | 2 | 131 | transferase activity, transferring acyl groups other than amino-acyl groups |
| GO:0030234 | 0.743 | 0.830 | 10 | 8 | 479 | enzyme regulator activity |
| GO:0008094 | 0.746 | 0.735 | 1 | 1 | 68 | DNA-dependent ATPase activity |
| GO:0070011 | 0.750 | 0.836 | 12 | 10 | 593 | peptidase activity, acting on L-amino acid peptides |
| GO:0015085 | 0.756 | 0.713 | 1 | 1 | 70 | calcium ion transmembrane transporter activity |
| GO:0052689 | 0.757 | 0.733 | 3 | 2 | 136 | carboxylic ester hydrolase activity |
| GO:0016903 | 0.761 | 0.703 | 1 | 1 | 71 | oxidoreductase activity, acting on the aldehyde or oxo group of donors |
| GO:0005261 | 0.771 | 0.712 | 3 | 2 | 140 | cation channel activity |
| GO:0008233 | 0.782 | 0.809 | 12 | 10 | 611 | peptidase activity |
| GO:0008509 | 0.784 | 0.656 | 2 | 1 | 76 | anion transmembrane transporter activity |
| GO:0000976 | 0.788 | 0.647 | 2 | 1 | 77 | transcription regulatory region sequence-specific DNA binding |
| GO:0015276 | 0.792 | 0.639 | 2 | 1 | 78 | ligand-gated ion channel activity |
| GO:0022834 | 0.792 | 0.639 | 2 | 1 | 78 | ligand-gated channel activity |
| GO:0015079 | 0.805 | 0.615 | 2 | 1 | 81 | potassium ion transmembrane transporter activity |
| GO:0072509 | 0.809 | 0.607 | 2 | 1 | 82 | divalent inorganic cation transmembrane transporter activity |
| GO:0003964 | 0.809 | 0.654 | 3 | 2 | 152 | RNA-directed DNA polymerase activity |
| GO:0008135 | 0.812 | 0.599 | 2 | 1 | 83 | translation factor activity, nucleic acid binding |
| GO:0004252 | 0.834 | 0.616 | 3 | 2 | 161 | serine-type endopeptidase activity |
| GO:0004222 | 0.837 | 0.552 | 2 | 1 | 90 | metalloendopeptidase activity |
| GO:0009055 | 0.841 | 0.646 | 5 | 3 | 230 | electron carrier activity |
| GO:0016787 | 0.843 | 0.845 | 45 | 40 | 2276 | hydrolase activity |
| GO:0005216 | 0.845 | 0.640 | 5 | 3 | 232 | ion channel activity |
| GO:0004497 | 0.845 | 0.640 | 5 | 3 | 232 | monooxygenase activity |
| GO:0022838 | 0.845 | 0.640 | 5 | 3 | 232 | substrate-specific channel activity |
| GO:0020037 | 0.853 | 0.629 | 5 | 3 | 236 | heme binding |
| GO:0015267 | 0.853 | 0.629 | 5 | 3 | 236 | channel activity |
| GO:0022803 | 0.853 | 0.629 | 5 | 3 | 236 | passive transmembrane transporter activity |
| GO:0035639 | 0.853 | 0.820 | 33 | 28 | 1652 | purine ribonucleoside triphosphate binding |
| GO:0004175 | 0.854 | 0.697 | 8 | 6 | 424 | endopeptidase activity |
| GO:0008047 | 0.861 | 0.575 | 3 | 2 | 172 | enzyme activator activity |
| GO:0046906 | 0.861 | 0.618 | 5 | 3 | 240 | tetrapyrrole binding |
| GO:0008234 | 0.867 | 0.495 | 2 | 1 | 100 | cysteine-type peptidase activity |
| GO:0017076 | 0.868 | 0.809 | 33 | 28 | 1669 | purine nucleotide binding |
| GO:0032553 | 0.868 | 0.809 | 33 | 28 | 1669 | ribonucleotide binding |
| GO:0032555 | 0.868 | 0.809 | 33 | 28 | 1669 | purine ribonucleotide binding |
| GO:0015077 | 0.869 | 0.562 | 3 | 2 | 176 | monovalent inorganic cation transmembrane transporter activity |
| GO:0022890 | 0.879 | 0.592 | 5 | 3 | 250 | inorganic cation transmembrane transporter activity |
| GO:0022892 | 0.885 | 0.702 | 12 | 9 | 627 | substrate-specific transporter activity |
| GO:0034061 | 0.890 | 0.528 | 4 | 2 | 187 | DNA polymerase activity |
| GO:0003735 | 0.898 | 0.437 | 2 | 1 | 113 | structural constituent of ribosome |
| GO:0000287 | 0.908 | 0.418 | 2 | 1 | 118 | magnesium ion binding |
| GO:0004857 | 0.913 | 0.408 | 2 | 1 | 121 | enzyme inhibitor activity |
| GO:0003700 | 0.915 | 0.598 | 8 | 5 | 409 | sequence-specific DNA binding transcription factor activity |
| GO:0001071 | 0.915 | 0.598 | 8 | 5 | 409 | nucleic acid binding transcription factor activity |
| GO:0016779 | 0.917 | 0.532 | 6 | 3 | 277 | nucleotidyltransferase activity |
| GO:0046873 | 0.918 | 0.480 | 4 | 2 | 205 | metal ion transmembrane transporter activity |
| GO:0004930 | 0.925 | 0.385 | 3 | 1 | 128 | G-protein coupled receptor activity |
| GO:0003924 | 0.928 | 0.379 | 3 | 1 | 130 | GTPase activity |
| GO:0008324 | 0.949 | 0.473 | 6 | 3 | 310 | cation transmembrane transporter activity |
| GO:0022804 | 0.959 | 0.453 | 6 | 3 | 323 | active transmembrane transporter activity |
| GO:0016462 | 0.965 | 0.597 | 16 | 10 | 804 | pyrophosphatase activity |
| GO:0016818 | 0.965 | 0.596 | 16 | 10 | 805 | hydrolase activity, acting on acid anhydrides, in phosphorus-containing anhydrides |
| GO:0016817 | 0.967 | 0.592 | 16 | 10 | 810 | hydrolase activity, acting on acid anhydrides |
| GO:0005215 | 0.971 | 0.594 | 18 | 11 | 885 | transporter activity |
| GO:0015075 | 0.977 | 0.442 | 9 | 4 | 437 | ion transmembrane transporter activity |
| GO:0017111 | 0.978 | 0.548 | 16 | 9 | 784 | nucleoside-triphosphatase activity |
| GO:0005525 | 0.989 | 0.220 | 4 | 1 | 221 | GTP binding |
| GO:0019001 | 0.991 | 0.207 | 5 | 1 | 234 | guanyl nucleotide binding |
| GO:0032561 | 0.991 | 0.207 | 5 | 1 | 234 | guanyl ribonucleotide binding |
| GO:0022891 | 0.996 | 0.350 | 11 | 4 | 543 | substrate-specific transmembrane transporter activity |
| GO:0022857 | 0.996 | 0.405 | 14 | 6 | 699 | transmembrane transporter activity |
