# Supplementary material for: Inducible Defenses Stay Up Late: Temporal Patterns of Immune Gene Expression in Tenebrio molitor
Source: G3 (Bethesda). 2014 Jun 1;4(6):947–55. doi: 10.1534/g3.113.008516 (PMC4065263; doi:10.1534/g3.113.008516)
Supplement: Supporting Information [file supp_g3.113.008516_TableS13.html]

TableS13 

# Inducible Defenses Stay Up Late: Temporal Patterns of Immune Gene Expression in *Tenebrio molitor*

Gene to GO BP test for over-representation

| GOBPID | Pvalue | OddsRatio | ExpCount | Count | Size | Term |
| GO:0045087 | 0.000 | 8.133 | 1 | 9 | 173 | innate immune response |
| GO:0006952 | 0.000 | 5.789 | 3 | 12 | 331 | defense response |
| GO:0042742 | 0.000 | 13.761 | 1 | 6 | 68 | defense response to bacterium |
| GO:0009617 | 0.000 | 8.108 | 1 | 7 | 131 | response to bacterium |
| GO:0009309 | 0.000 | 20.439 | 0 | 4 | 31 | amine biosynthetic process |
| GO:0051865 | 0.000 | 29.118 | 0 | 3 | 17 | protein autoubiquitination |
| GO:0042427 | 0.000 | 133.852 | 0 | 2 | 4 | serotonin biosynthetic process |
| GO:0014010 | 0.000 | 133.852 | 0 | 2 | 4 | Schwann cell proliferation |
| GO:0042430 | 0.001 | 22.636 | 0 | 3 | 21 | indole-containing compound metabolic process |
| GO:0006586 | 0.001 | 22.636 | 0 | 3 | 21 | indolalkylamine metabolic process |
| GO:0042416 | 0.001 | 89.224 | 0 | 2 | 5 | dopamine biosynthetic process |
| GO:0006955 | 0.001 | 4.267 | 2 | 9 | 316 | immune response |
| GO:0042435 | 0.001 | 66.910 | 0 | 2 | 6 | indole-containing compound biosynthetic process |
| GO:0046219 | 0.001 | 66.910 | 0 | 2 | 6 | indolalkylamine biosynthetic process |
| GO:0009072 | 0.001 | 17.704 | 0 | 3 | 26 | aromatic amino acid family metabolic process |
| GO:0035520 | 0.001 | 53.521 | 0 | 2 | 7 | monoubiquitinated protein deubiquitination |
| GO:0042401 | 0.001 | 16.284 | 0 | 3 | 28 | cellular biogenic amine biosynthetic process |
| GO:0008063 | 0.001 | 9.317 | 0 | 4 | 63 | Toll signaling pathway |
| GO:0060253 | 0.002 | 44.596 | 0 | 2 | 8 | negative regulation of glial cell proliferation |
| GO:0042423 | 0.002 | 44.596 | 0 | 2 | 8 | catecholamine biosynthetic process |
| GO:0009713 | 0.002 | 44.596 | 0 | 2 | 8 | catechol-containing compound biosynthetic process |
| GO:0009820 | 0.002 | 44.596 | 0 | 2 | 8 | alkaloid metabolic process |
| GO:0034312 | 0.002 | 44.596 | 0 | 2 | 8 | diol biosynthetic process |
| GO:0007311 | 0.002 | 14.031 | 0 | 3 | 32 | maternal specification of dorsal/ventral axis, oocyte, germ-line encoded |
| GO:0009308 | 0.002 | 6.257 | 1 | 5 | 116 | amine metabolic process |
| GO:0042428 | 0.002 | 38.220 | 0 | 2 | 9 | serotonin metabolic process |
| GO:0060251 | 0.002 | 33.439 | 0 | 2 | 10 | regulation of glial cell proliferation |
| GO:1901162 | 0.002 | 33.439 | 0 | 2 | 10 | primary amino compound biosynthetic process |
| GO:0051919 | 0.002 | 33.439 | 0 | 2 | 10 | positive regulation of fibrinolysis |
| GO:0009950 | 0.003 | 7.622 | 1 | 4 | 76 | dorsal/ventral axis specification |
| GO:0009994 | 0.003 | 5.638 | 1 | 5 | 128 | oocyte differentiation |
| GO:0014009 | 0.003 | 29.719 | 0 | 2 | 11 | glial cell proliferation |
| GO:0051917 | 0.003 | 29.719 | 0 | 2 | 11 | regulation of fibrinolysis |
| GO:0031639 | 0.003 | 29.719 | 0 | 2 | 11 | plasminogen activation |
| GO:0046165 | 0.003 | 10.986 | 0 | 3 | 40 | alcohol biosynthetic process |
| GO:0006508 | 0.004 | 2.529 | 6 | 14 | 843 | proteolysis |
| GO:0051707 | 0.004 | 3.786 | 2 | 7 | 268 | response to other organism |
| GO:0042417 | 0.004 | 24.310 | 0 | 2 | 13 | dopamine metabolic process |
| GO:0014014 | 0.004 | 24.310 | 0 | 2 | 13 | negative regulation of gliogenesis |
| GO:0009607 | 0.005 | 3.727 | 2 | 7 | 272 | response to biotic stimulus |
| GO:0002376 | 0.005 | 2.777 | 5 | 11 | 589 | immune system process |
| GO:0009074 | 0.005 | 22.281 | 0 | 2 | 14 | aromatic amino acid family catabolic process |
| GO:0030162 | 0.005 | 6.224 | 1 | 4 | 92 | regulation of proteolysis |
| GO:0009953 | 0.006 | 4.769 | 1 | 5 | 150 | dorsal/ventral pattern formation |
| GO:0019731 | 0.006 | 20.565 | 0 | 2 | 15 | antibacterial humoral response |
| GO:0006576 | 0.006 | 6.017 | 1 | 4 | 95 | cellular biogenic amine metabolic process |
| GO:0042730 | 0.006 | 19.094 | 0 | 2 | 16 | fibrinolysis |
| GO:1901160 | 0.006 | 19.094 | 0 | 2 | 16 | primary amino compound metabolic process |
| GO:0070973 | 0.008 | Inf | 0 | 1 | 1 | protein localization to endoplasmic reticulum exit site |
| GO:0014040 | 0.008 | Inf | 0 | 1 | 1 | positive regulation of Schwann cell differentiation |
| GO:0014038 | 0.008 | Inf | 0 | 1 | 1 | regulation of Schwann cell differentiation |
| GO:0042524 | 0.008 | Inf | 0 | 1 | 1 | negative regulation of tyrosine phosphorylation of Stat5 protein |
| GO:0032290 | 0.008 | Inf | 0 | 1 | 1 | peripheral nervous system myelin formation |
| GO:0035814 | 0.008 | Inf | 0 | 1 | 1 | negative regulation of renal sodium excretion |
| GO:0035519 | 0.008 | Inf | 0 | 1 | 1 | protein K29-linked ubiquitination |
| GO:0051319 | 0.008 | Inf | 0 | 1 | 1 | G2 phase |
| GO:0000085 | 0.008 | Inf | 0 | 1 | 1 | G2 phase of mitotic cell cycle |
| GO:0080167 | 0.008 | Inf | 0 | 1 | 1 | response to karrikin |
| GO:0044314 | 0.008 | Inf | 0 | 1 | 1 | protein K27-linked ubiquitination |
| GO:0007309 | 0.008 | 5.525 | 1 | 4 | 103 | oocyte axis specification |
| GO:0007310 | 0.008 | 7.957 | 0 | 3 | 54 | oocyte dorsal/ventral axis specification |
| GO:1900047 | 0.008 | 16.703 | 0 | 2 | 18 | negative regulation of hemostasis |
| GO:0030195 | 0.008 | 16.703 | 0 | 2 | 18 | negative regulation of blood coagulation |
| GO:0044106 | 0.008 | 5.414 | 1 | 4 | 105 | cellular amine metabolic process |
| GO:0007308 | 0.009 | 5.361 | 1 | 4 | 106 | oocyte construction |
| GO:0006950 | 0.009 | 2.062 | 11 | 19 | 1433 | response to stress |
| GO:0014013 | 0.009 | 15.718 | 0 | 2 | 19 | regulation of gliogenesis |
| GO:0050819 | 0.009 | 15.718 | 0 | 2 | 19 | negative regulation of coagulation |
| GO:0009798 | 0.009 | 4.181 | 1 | 5 | 170 | axis specification |
| GO:1900046 | 0.010 | 14.843 | 0 | 2 | 20 | regulation of hemostasis |
| GO:0030193 | 0.010 | 14.843 | 0 | 2 | 20 | regulation of blood coagulation |
| GO:0016485 | 0.010 | 5.107 | 1 | 4 | 111 | protein processing |
| GO:0006725 | 0.011 | 4.055 | 1 | 5 | 175 | cellular aromatic compound metabolic process |
| GO:0050818 | 0.011 | 14.060 | 0 | 2 | 21 | regulation of coagulation |
| GO:0051704 | 0.012 | 2.524 | 4 | 10 | 578 | multi-organism process |
| GO:0051604 | 0.012 | 4.832 | 1 | 4 | 117 | protein maturation |
| GO:0048599 | 0.013 | 4.705 | 1 | 4 | 120 | oocyte development |
| GO:0021700 | 0.015 | 3.719 | 1 | 5 | 190 | developmental maturation |
| GO:0042522 | 0.015 | 131.710 | 0 | 1 | 2 | regulation of tyrosine phosphorylation of Stat5 protein |
| GO:0042518 | 0.015 | 131.710 | 0 | 1 | 2 | negative regulation of tyrosine phosphorylation of Stat3 protein |
| GO:0042532 | 0.015 | 131.710 | 0 | 1 | 2 | negative regulation of tyrosine phosphorylation of STAT protein |
| GO:0017187 | 0.015 | 131.710 | 0 | 1 | 2 | peptidyl-glutamic acid carboxylation |
| GO:0033076 | 0.015 | 131.710 | 0 | 1 | 2 | isoquinoline alkaloid metabolic process |
| GO:0015842 | 0.015 | 131.710 | 0 | 1 | 2 | synaptic vesicle amine transport |
| GO:0018214 | 0.015 | 131.710 | 0 | 1 | 2 | protein carboxylation |
| GO:0052314 | 0.015 | 131.710 | 0 | 1 | 2 | phytoalexin metabolic process |
| GO:0031638 | 0.015 | 11.609 | 0 | 2 | 25 | zymogen activation |
| GO:0080134 | 0.016 | 3.176 | 2 | 6 | 268 | regulation of response to stress |
| GO:0010632 | 0.019 | 10.266 | 0 | 2 | 28 | regulation of epithelial cell migration |
| GO:0006584 | 0.021 | 9.885 | 0 | 2 | 29 | catecholamine metabolic process |
| GO:0009712 | 0.021 | 9.885 | 0 | 2 | 29 | catechol-containing compound metabolic process |
| GO:0034311 | 0.021 | 9.885 | 0 | 2 | 29 | diol metabolic process |
| GO:0048469 | 0.022 | 4.003 | 1 | 4 | 140 | cell maturation |
| GO:0042412 | 0.023 | 65.847 | 0 | 1 | 3 | taurine biosynthetic process |
| GO:0042506 | 0.023 | 65.847 | 0 | 1 | 3 | tyrosine phosphorylation of Stat5 protein |
| GO:0017000 | 0.023 | 65.847 | 0 | 1 | 3 | antibiotic biosynthetic process |
| GO:0032527 | 0.023 | 65.847 | 0 | 1 | 3 | protein exit from endoplasmic reticulum |
| GO:0045687 | 0.023 | 65.847 | 0 | 1 | 3 | positive regulation of glial cell differentiation |
| GO:0085020 | 0.023 | 65.847 | 0 | 1 | 3 | protein K6-linked ubiquitination |
| GO:0010624 | 0.023 | 65.847 | 0 | 1 | 3 | regulation of Schwann cell proliferation |
| GO:0010626 | 0.023 | 65.847 | 0 | 1 | 3 | negative regulation of Schwann cell proliferation |
| GO:0010633 | 0.023 | 65.847 | 0 | 1 | 3 | negative regulation of epithelial cell migration |
| GO:0070306 | 0.023 | 65.847 | 0 | 1 | 3 | lens fiber cell differentiation |
| GO:0031643 | 0.023 | 65.847 | 0 | 1 | 3 | positive regulation of myelination |
| GO:0044262 | 0.023 | 3.944 | 1 | 4 | 142 | cellular carbohydrate metabolic process |
| GO:0019439 | 0.023 | 9.201 | 0 | 2 | 31 | aromatic compound catabolic process |
| GO:0007281 | 0.025 | 3.235 | 2 | 5 | 217 | germ cell development |
| GO:0061041 | 0.026 | 8.605 | 0 | 2 | 33 | regulation of wound healing |
| GO:0009063 | 0.026 | 4.991 | 1 | 3 | 84 | cellular amino acid catabolic process |
| GO:0019441 | 0.030 | 43.892 | 0 | 1 | 4 | tryptophan catabolic process to kynurenine |
| GO:0042516 | 0.030 | 43.892 | 0 | 1 | 4 | regulation of tyrosine phosphorylation of Stat3 protein |
| GO:0071312 | 0.030 | 43.892 | 0 | 1 | 4 | cellular response to alkaloid |
| GO:0019530 | 0.030 | 43.892 | 0 | 1 | 4 | taurine metabolic process |
| GO:0035812 | 0.030 | 43.892 | 0 | 1 | 4 | renal sodium excretion |
| GO:0035813 | 0.030 | 43.892 | 0 | 1 | 4 | regulation of renal sodium excretion |
| GO:0055078 | 0.030 | 43.892 | 0 | 1 | 4 | sodium ion homeostasis |
| GO:0018958 | 0.031 | 7.843 | 0 | 2 | 36 | phenol-containing compound metabolic process |
| GO:0003002 | 0.032 | 2.473 | 3 | 7 | 400 | regionalization |
| GO:0019538 | 0.033 | 1.687 | 17 | 24 | 2207 | protein metabolic process |
| GO:0006520 | 0.035 | 2.624 | 2 | 6 | 321 | cellular amino acid metabolic process |
| GO:0051246 | 0.036 | 2.152 | 5 | 9 | 596 | regulation of protein metabolic process |
| GO:0016999 | 0.038 | 32.915 | 0 | 1 | 5 | antibiotic metabolic process |
| GO:0050732 | 0.038 | 32.915 | 0 | 1 | 5 | negative regulation of peptidyl-tyrosine phosphorylation |
| GO:0042503 | 0.038 | 32.915 | 0 | 1 | 5 | tyrosine phosphorylation of Stat3 protein |
| GO:0048082 | 0.038 | 32.915 | 0 | 1 | 5 | regulation of adult chitin-containing cuticle pigmentation |
| GO:0048079 | 0.038 | 32.915 | 0 | 1 | 5 | regulation of cuticle pigmentation |
| GO:0048026 | 0.038 | 32.915 | 0 | 1 | 5 | positive regulation of nuclear mRNA splicing, via spliceosome |
| GO:0035262 | 0.038 | 32.915 | 0 | 1 | 5 | gonad morphogenesis |
| GO:0007564 | 0.038 | 32.915 | 0 | 1 | 5 | regulation of chitin-based cuticle tanning |
| GO:0046426 | 0.038 | 32.915 | 0 | 1 | 5 | negative regulation of JAK-STAT cascade |
| GO:0046684 | 0.038 | 32.915 | 0 | 1 | 5 | response to pyrethroid |
| GO:0006021 | 0.038 | 32.915 | 0 | 1 | 5 | inositol biosynthetic process |
| GO:0019438 | 0.043 | 6.498 | 0 | 2 | 43 | aromatic compound biosynthetic process |
| GO:0006954 | 0.043 | 4.075 | 1 | 3 | 102 | inflammatory response |
| GO:0051017 | 0.044 | 6.343 | 0 | 2 | 44 | actin filament bundle assembly |
| GO:0031935 | 0.045 | 26.329 | 0 | 1 | 6 | regulation of chromatin silencing |
| GO:0021591 | 0.045 | 26.329 | 0 | 1 | 6 | ventricular system development |
| GO:0019474 | 0.045 | 26.329 | 0 | 1 | 6 | L-lysine catabolic process to acetyl-CoA |
| GO:0019477 | 0.045 | 26.329 | 0 | 1 | 6 | L-lysine catabolic process |
| GO:0042509 | 0.045 | 26.329 | 0 | 1 | 6 | regulation of tyrosine phosphorylation of STAT protein |
| GO:0001504 | 0.045 | 26.329 | 0 | 1 | 6 | neurotransmitter uptake |
| GO:0048251 | 0.045 | 26.329 | 0 | 1 | 6 | elastic fiber assembly |
| GO:0048207 | 0.045 | 26.329 | 0 | 1 | 6 | vesicle targeting, rough ER to cis-Golgi |
| GO:0048208 | 0.045 | 26.329 | 0 | 1 | 6 | COPII vesicle coating |
| GO:0030423 | 0.045 | 26.329 | 0 | 1 | 6 | targeting of mRNA for destruction involved in RNA interference |
| GO:0030727 | 0.045 | 26.329 | 0 | 1 | 6 | germarium-derived female germ-line cyst formation |
| GO:0030513 | 0.045 | 26.329 | 0 | 1 | 6 | positive regulation of BMP signaling pathway |
| GO:0085029 | 0.045 | 26.329 | 0 | 1 | 6 | extracellular matrix assembly |
| GO:0046440 | 0.045 | 26.329 | 0 | 1 | 6 | L-lysine metabolic process |
| GO:0033512 | 0.045 | 26.329 | 0 | 1 | 6 | L-lysine catabolic process to acetyl-CoA via saccharopine |
| GO:0046855 | 0.045 | 26.329 | 0 | 1 | 6 | inositol phosphate dephosphorylation |
| GO:0046838 | 0.045 | 26.329 | 0 | 1 | 6 | phosphorylated carbohydrate dephosphorylation |
| GO:0070189 | 0.045 | 26.329 | 0 | 1 | 6 | kynurenine metabolic process |
| GO:0006959 | 0.047 | 3.915 | 1 | 3 | 106 | humoral immune response |
| GO:0045088 | 0.048 | 6.053 | 0 | 2 | 46 | regulation of innate immune response |
| GO:0051241 | 0.050 | 3.839 | 1 | 3 | 108 | negative regulation of multicellular organismal process |
| GO:0031347 | 0.052 | 3.766 | 1 | 3 | 110 | regulation of defense response |
| GO:0070922 | 0.052 | 21.938 | 0 | 1 | 7 | small RNA loading onto RISC |
| GO:0042136 | 0.052 | 21.938 | 0 | 1 | 7 | neurotransmitter biosynthetic process |
| GO:0014015 | 0.052 | 21.938 | 0 | 1 | 7 | positive regulation of gliogenesis |
| GO:0040006 | 0.052 | 21.938 | 0 | 1 | 7 | protein-based cuticle attachment to epithelium |
| GO:0006558 | 0.052 | 21.938 | 0 | 1 | 7 | L-phenylalanine metabolic process |
| GO:0006559 | 0.052 | 21.938 | 0 | 1 | 7 | L-phenylalanine catabolic process |
| GO:0048085 | 0.052 | 21.938 | 0 | 1 | 7 | adult chitin-containing cuticle pigmentation |
| GO:0035087 | 0.052 | 21.938 | 0 | 1 | 7 | siRNA loading onto RISC involved in RNA interference |
| GO:0048135 | 0.052 | 21.938 | 0 | 1 | 7 | female germ-line cyst formation |
| GO:0071545 | 0.052 | 21.938 | 0 | 1 | 7 | inositol phosphate catabolic process |
| GO:0007260 | 0.052 | 21.938 | 0 | 1 | 7 | tyrosine phosphorylation of STAT protein |
| GO:0007593 | 0.052 | 21.938 | 0 | 1 | 7 | chitin-based cuticle tanning |
| GO:0090114 | 0.052 | 21.938 | 0 | 1 | 7 | COPII-coated vesicle budding |
| GO:0010216 | 0.052 | 21.938 | 0 | 1 | 7 | maintenance of DNA methylation |
| GO:0018200 | 0.052 | 21.938 | 0 | 1 | 7 | peptidyl-glutamic acid modification |
| GO:0008365 | 0.052 | 21.938 | 0 | 1 | 7 | adult chitin-based cuticle development |
| GO:0009411 | 0.054 | 5.664 | 0 | 2 | 49 | response to UV |
| GO:0030336 | 0.056 | 5.546 | 0 | 2 | 50 | negative regulation of cell migration |
| GO:0016579 | 0.058 | 5.432 | 0 | 2 | 51 | protein deubiquitination |
| GO:0016201 | 0.060 | 18.802 | 0 | 1 | 8 | synaptic target inhibition |
| GO:0040040 | 0.060 | 18.802 | 0 | 1 | 8 | thermosensory behavior |
| GO:0006569 | 0.060 | 18.802 | 0 | 1 | 8 | tryptophan catabolic process |
| GO:0050829 | 0.060 | 18.802 | 0 | 1 | 8 | defense response to Gram-negative bacterium |
| GO:0045685 | 0.060 | 18.802 | 0 | 1 | 8 | regulation of glial cell differentiation |
| GO:0035279 | 0.060 | 18.802 | 0 | 1 | 8 | mRNA cleavage involved in gene silencing by miRNA |
| GO:0046173 | 0.060 | 18.802 | 0 | 1 | 8 | polyol biosynthetic process |
| GO:2000146 | 0.060 | 5.323 | 0 | 2 | 52 | negative regulation of cell motility |
| GO:0002440 | 0.062 | 5.218 | 0 | 2 | 53 | production of molecular mediator of immune response |
| GO:0090132 | 0.062 | 5.218 | 0 | 2 | 53 | epithelium migration |
| GO:0010631 | 0.062 | 5.218 | 0 | 2 | 53 | epithelial cell migration |
| GO:0001558 | 0.063 | 3.470 | 1 | 3 | 119 | regulation of cell growth |
| GO:0007389 | 0.063 | 2.109 | 4 | 7 | 464 | pattern specification process |
| GO:0050776 | 0.064 | 3.440 | 1 | 3 | 120 | regulation of immune response |
| GO:2000021 | 0.064 | 5.117 | 0 | 2 | 54 | regulation of ion homeostasis |
| GO:0040029 | 0.065 | 3.411 | 1 | 3 | 121 | regulation of gene expression, epigenetic |
| GO:0006469 | 0.066 | 5.019 | 0 | 2 | 55 | negative regulation of protein kinase activity |
| GO:0043648 | 0.066 | 5.019 | 0 | 2 | 55 | dicarboxylic acid metabolic process |
| GO:0051271 | 0.066 | 5.019 | 0 | 2 | 55 | negative regulation of cellular component movement |
| GO:0090130 | 0.066 | 5.019 | 0 | 2 | 55 | tissue migration |
| GO:0021904 | 0.067 | 16.450 | 0 | 1 | 9 | dorsal/ventral neural tube patterning |
| GO:0006553 | 0.067 | 16.450 | 0 | 1 | 9 | lysine metabolic process |
| GO:0006554 | 0.067 | 16.450 | 0 | 1 | 9 | lysine catabolic process |
| GO:0032288 | 0.067 | 16.450 | 0 | 1 | 9 | myelin assembly |
| GO:0050685 | 0.067 | 16.450 | 0 | 1 | 9 | positive regulation of mRNA processing |
| GO:0009405 | 0.067 | 16.450 | 0 | 1 | 9 | pathogenesis |
| GO:0006953 | 0.067 | 16.450 | 0 | 1 | 9 | acute-phase response |
| GO:0035330 | 0.067 | 16.450 | 0 | 1 | 9 | regulation of hippo signaling cascade |
| GO:0030422 | 0.067 | 16.450 | 0 | 1 | 9 | production of siRNA involved in RNA interference |
| GO:0030212 | 0.067 | 16.450 | 0 | 1 | 9 | hyaluronan metabolic process |
| GO:0002088 | 0.067 | 16.450 | 0 | 1 | 9 | lens development in camera-type eye |
| GO:0033120 | 0.067 | 16.450 | 0 | 1 | 9 | positive regulation of RNA splicing |
| GO:0000098 | 0.067 | 16.450 | 0 | 1 | 9 | sulfur amino acid catabolic process |
| GO:0044062 | 0.067 | 16.450 | 0 | 1 | 9 | regulation of excretion |
| GO:0046669 | 0.067 | 16.450 | 0 | 1 | 9 | regulation of compound eye retinal cell programmed cell death |
| GO:0031054 | 0.067 | 16.450 | 0 | 1 | 9 | pre-miRNA processing |
| GO:0070646 | 0.070 | 4.836 | 0 | 2 | 57 | protein modification by small protein removal |
| GO:0007596 | 0.070 | 3.297 | 1 | 3 | 125 | blood coagulation |
| GO:0007349 | 0.073 | 4.749 | 0 | 2 | 58 | cellularization |
| GO:0022618 | 0.073 | 4.749 | 0 | 2 | 58 | ribonucleoprotein complex assembly |
| GO:0042436 | 0.074 | 14.620 | 0 | 1 | 10 | indole-containing compound catabolic process |
| GO:0006509 | 0.074 | 14.620 | 0 | 1 | 10 | membrane protein ectodomain proteolysis |
| GO:0048067 | 0.074 | 14.620 | 0 | 1 | 10 | cuticle pigmentation |
| GO:0048199 | 0.074 | 14.620 | 0 | 1 | 10 | vesicle targeting, to, from or within Golgi |
| GO:0017144 | 0.074 | 14.620 | 0 | 1 | 10 | drug metabolic process |
| GO:0001953 | 0.074 | 14.620 | 0 | 1 | 10 | negative regulation of cell-matrix adhesion |
| GO:0007352 | 0.074 | 14.620 | 0 | 1 | 10 | zygotic specification of dorsal/ventral axis |
| GO:0051496 | 0.074 | 14.620 | 0 | 1 | 10 | positive regulation of stress fiber assembly |
| GO:0046218 | 0.074 | 14.620 | 0 | 1 | 10 | indolalkylamine catabolic process |
| GO:0046668 | 0.074 | 14.620 | 0 | 1 | 10 | regulation of retinal cell programmed cell death |
| GO:0005991 | 0.074 | 14.620 | 0 | 1 | 10 | trehalose metabolic process |
| GO:0031641 | 0.074 | 14.620 | 0 | 1 | 10 | regulation of myelination |
| GO:0040013 | 0.075 | 4.665 | 0 | 2 | 59 | negative regulation of locomotion |
| GO:0033673 | 0.075 | 4.665 | 0 | 2 | 59 | negative regulation of kinase activity |
| GO:0051348 | 0.077 | 4.584 | 0 | 2 | 60 | negative regulation of transferase activity |
| GO:0006309 | 0.081 | 13.156 | 0 | 1 | 11 | apoptotic DNA fragmentation |
| GO:0006265 | 0.081 | 13.156 | 0 | 1 | 11 | DNA topological change |
| GO:0006826 | 0.081 | 13.156 | 0 | 1 | 11 | iron ion transport |
| GO:0040034 | 0.081 | 13.156 | 0 | 1 | 11 | regulation of development, heterochronic |
| GO:0032456 | 0.081 | 13.156 | 0 | 1 | 11 | endocytic recycling |
| GO:0032292 | 0.081 | 13.156 | 0 | 1 | 11 | peripheral nervous system axon ensheathment |
| GO:0022011 | 0.081 | 13.156 | 0 | 1 | 11 | myelination in peripheral nervous system |
| GO:0045793 | 0.081 | 13.156 | 0 | 1 | 11 | positive regulation of cell size |
| GO:0022408 | 0.081 | 13.156 | 0 | 1 | 11 | negative regulation of cell-cell adhesion |
| GO:0043052 | 0.081 | 13.156 | 0 | 1 | 11 | thermotaxis |
| GO:0000045 | 0.081 | 13.156 | 0 | 1 | 11 | autophagic vacuole assembly |
| GO:0016075 | 0.081 | 13.156 | 0 | 1 | 11 | rRNA catabolic process |
| GO:0071826 | 0.081 | 4.430 | 0 | 2 | 62 | ribonucleoprotein complex subunit organization |
| GO:0050817 | 0.083 | 3.067 | 1 | 3 | 134 | coagulation |
| GO:0007599 | 0.083 | 3.067 | 1 | 3 | 134 | hemostasis |
| GO:0006464 | 0.084 | 1.639 | 9 | 13 | 1131 | cellular protein modification process |
| GO:0036211 | 0.084 | 1.639 | 9 | 13 | 1131 | protein modification process |
| GO:0046395 | 0.084 | 3.044 | 1 | 3 | 135 | carboxylic acid catabolic process |
| GO:0016054 | 0.084 | 3.044 | 1 | 3 | 135 | organic acid catabolic process |
| GO:0046488 | 0.086 | 4.286 | 0 | 2 | 64 | phosphatidylinositol metabolic process |
| GO:0009611 | 0.087 | 2.230 | 2 | 5 | 309 | response to wounding |
| GO:0042381 | 0.088 | 11.959 | 0 | 1 | 12 | hemolymph coagulation |
| GO:0021532 | 0.088 | 11.959 | 0 | 1 | 12 | neural tube patterning |
| GO:0014044 | 0.088 | 11.959 | 0 | 1 | 12 | Schwann cell development |
| GO:0032233 | 0.088 | 11.959 | 0 | 1 | 12 | positive regulation of actin filament bundle assembly |
| GO:0035196 | 0.088 | 11.959 | 0 | 1 | 12 | production of miRNAs involved in gene silencing by miRNA |
| GO:0043154 | 0.088 | 11.959 | 0 | 1 | 12 | negative regulation of cysteine-type endopeptidase activity involved in apoptotic process |
| GO:0007112 | 0.088 | 11.959 | 0 | 1 | 12 | male meiosis cytokinesis |
| GO:0033619 | 0.088 | 11.959 | 0 | 1 | 12 | membrane protein proteolysis |
| GO:0003014 | 0.088 | 11.959 | 0 | 1 | 12 | renal system process |
| GO:0006020 | 0.088 | 11.959 | 0 | 1 | 12 | inositol metabolic process |
| GO:0006084 | 0.091 | 4.151 | 1 | 2 | 66 | acetyl-CoA metabolic process |
| GO:0006915 | 0.093 | 1.825 | 5 | 8 | 611 | apoptotic process |
| GO:0030334 | 0.095 | 2.888 | 1 | 3 | 142 | regulation of cell migration |
| GO:0070979 | 0.095 | 10.961 | 0 | 1 | 13 | protein K11-linked ubiquitination |
| GO:0014037 | 0.095 | 10.961 | 0 | 1 | 13 | Schwann cell differentiation |
| GO:0006817 | 0.095 | 10.961 | 0 | 1 | 13 | phosphate ion transport |
| GO:0009409 | 0.095 | 10.961 | 0 | 1 | 13 | response to cold |
| GO:0006963 | 0.095 | 10.961 | 0 | 1 | 13 | positive regulation of antibacterial peptide biosynthetic process |
| GO:0045736 | 0.095 | 10.961 | 0 | 1 | 13 | negative regulation of cyclin-dependent protein kinase activity |
| GO:0030262 | 0.095 | 10.961 | 0 | 1 | 13 | apoptotic nuclear change |
| GO:0002780 | 0.095 | 10.961 | 0 | 1 | 13 | antibacterial peptide biosynthetic process |
| GO:0002786 | 0.095 | 10.961 | 0 | 1 | 13 | regulation of antibacterial peptide production |
| GO:0002778 | 0.095 | 10.961 | 0 | 1 | 13 | antibacterial peptide production |
| GO:0002808 | 0.095 | 10.961 | 0 | 1 | 13 | regulation of antibacterial peptide biosynthetic process |
| GO:0018991 | 0.095 | 10.961 | 0 | 1 | 13 | oviposition |
| GO:0050768 | 0.100 | 3.905 | 1 | 2 | 70 | negative regulation of neurogenesis |
| GO:0001933 | 0.100 | 3.905 | 1 | 2 | 70 | negative regulation of protein phosphorylation |
| GO:0050896 | 0.101 | 1.427 | 26 | 31 | 3334 | response to stimulus |
| GO:0019752 | 0.101 | 1.786 | 5 | 8 | 623 | carboxylic acid metabolic process |
| GO:0043436 | 0.101 | 1.786 | 5 | 8 | 623 | oxoacid metabolic process |
| GO:0030716 | 0.102 | 10.117 | 0 | 1 | 14 | oocyte fate determination |
| GO:0007294 | 0.102 | 10.117 | 0 | 1 | 14 | germarium-derived oocyte fate determination |
| GO:0051492 | 0.102 | 10.117 | 0 | 1 | 14 | regulation of stress fiber assembly |
| GO:0043647 | 0.102 | 10.117 | 0 | 1 | 14 | inositol phosphate metabolic process |
| GO:0010972 | 0.102 | 10.117 | 0 | 1 | 14 | negative regulation of G2/M transition of mitotic cell cycle |
| GO:0005984 | 0.102 | 10.117 | 0 | 1 | 14 | disaccharide metabolic process |
| GO:2000145 | 0.102 | 2.786 | 1 | 3 | 147 | regulation of cell motility |
| GO:0043010 | 0.102 | 3.848 | 1 | 2 | 71 | camera-type eye development |
| GO:0006082 | 0.105 | 1.767 | 5 | 8 | 629 | organic acid metabolic process |
| GO:0034661 | 0.109 | 9.393 | 0 | 1 | 15 | ncRNA catabolic process |
| GO:0070918 | 0.109 | 9.393 | 0 | 1 | 15 | production of small RNA involved in gene silencing by RNA |
| GO:0070936 | 0.109 | 9.393 | 0 | 1 | 15 | protein K48-linked ubiquitination |
| GO:0060968 | 0.109 | 9.393 | 0 | 1 | 15 | regulation of gene silencing |
| GO:0006568 | 0.109 | 9.393 | 0 | 1 | 15 | tryptophan metabolic process |
| GO:0006967 | 0.109 | 9.393 | 0 | 1 | 15 | positive regulation of antifungal peptide biosynthetic process |
| GO:1900150 | 0.109 | 9.393 | 0 | 1 | 15 | regulation of defense response to fungus |
| GO:0043149 | 0.109 | 9.393 | 0 | 1 | 15 | stress fiber assembly |
| GO:0002781 | 0.109 | 9.393 | 0 | 1 | 15 | antifungal peptide production |
| GO:0002783 | 0.109 | 9.393 | 0 | 1 | 15 | antifungal peptide biosynthetic process |
| GO:0002788 | 0.109 | 9.393 | 0 | 1 | 15 | regulation of antifungal peptide production |
| GO:0002810 | 0.109 | 9.393 | 0 | 1 | 15 | regulation of antifungal peptide biosynthetic process |
| GO:0031050 | 0.109 | 9.393 | 0 | 1 | 15 | dsRNA fragmentation |
| GO:0010812 | 0.109 | 9.393 | 0 | 1 | 15 | negative regulation of cell-substrate adhesion |
| GO:0008156 | 0.109 | 9.393 | 0 | 1 | 15 | negative regulation of DNA replication |
| GO:2000274 | 0.109 | 9.393 | 0 | 1 | 15 | regulation of epithelial cell migration, open tracheal system |
| GO:0042326 | 0.110 | 3.686 | 1 | 2 | 74 | negative regulation of phosphorylation |
| GO:0001667 | 0.112 | 3.635 | 1 | 2 | 75 | ameboidal cell migration |
| GO:0009880 | 0.115 | 2.637 | 1 | 3 | 155 | embryonic pattern specification |
| GO:0043086 | 0.115 | 2.637 | 1 | 3 | 155 | negative regulation of catalytic activity |
| GO:0042475 | 0.116 | 8.766 | 0 | 1 | 16 | odontogenesis of dentin-containing tooth |
| GO:0048070 | 0.116 | 8.766 | 0 | 1 | 16 | regulation of developmental pigmentation |
| GO:0048009 | 0.116 | 8.766 | 0 | 1 | 16 | insulin-like growth factor receptor signaling pathway |
| GO:0071359 | 0.116 | 8.766 | 0 | 1 | 16 | cellular response to dsRNA |
| GO:1900424 | 0.116 | 8.766 | 0 | 1 | 16 | regulation of defense response to bacterium |
| GO:0009629 | 0.116 | 8.766 | 0 | 1 | 16 | response to gravity |
| GO:0030706 | 0.116 | 8.766 | 0 | 1 | 16 | germarium-derived oocyte differentiation |
| GO:0033206 | 0.116 | 8.766 | 0 | 1 | 16 | cytokinesis after meiosis |
| GO:0008101 | 0.116 | 8.766 | 0 | 1 | 16 | decapentaplegic signaling pathway |
| GO:0070647 | 0.116 | 2.028 | 3 | 5 | 338 | protein modification by small protein conjugation or removal |
| GO:0006814 | 0.117 | 3.537 | 1 | 2 | 77 | sodium ion transport |
| GO:0045936 | 0.117 | 3.537 | 1 | 2 | 77 | negative regulation of phosphate metabolic process |
| GO:0010563 | 0.117 | 3.537 | 1 | 2 | 77 | negative regulation of phosphorus metabolic process |
| GO:0043412 | 0.118 | 1.533 | 9 | 13 | 1197 | macromolecule modification |
| GO:0006400 | 0.123 | 8.217 | 0 | 1 | 17 | tRNA modification |
| GO:0006108 | 0.123 | 8.217 | 0 | 1 | 17 | malate metabolic process |
| GO:0009068 | 0.123 | 8.217 | 0 | 1 | 17 | aspartate family amino acid catabolic process |
| GO:0050830 | 0.123 | 8.217 | 0 | 1 | 17 | defense response to Gram-positive bacterium |
| GO:0019732 | 0.123 | 8.217 | 0 | 1 | 17 | antifungal humoral response |
| GO:0019853 | 0.123 | 8.217 | 0 | 1 | 17 | L-ascorbic acid biosynthetic process |
| GO:0032231 | 0.123 | 8.217 | 0 | 1 | 17 | regulation of actin filament bundle assembly |
| GO:0017085 | 0.123 | 8.217 | 0 | 1 | 17 | response to insecticide |
| GO:0043331 | 0.123 | 8.217 | 0 | 1 | 17 | response to dsRNA |
| GO:0046854 | 0.123 | 8.217 | 0 | 1 | 17 | phosphatidylinositol phosphorylation |
| GO:0000737 | 0.123 | 8.217 | 0 | 1 | 17 | DNA catabolic process, endonucleolytic |
| GO:2000117 | 0.123 | 8.217 | 0 | 1 | 17 | negative regulation of cysteine-type endopeptidase activity |
| GO:0070534 | 0.123 | 8.217 | 0 | 1 | 17 | protein K63-linked ubiquitination |
| GO:0055067 | 0.123 | 8.217 | 0 | 1 | 17 | monovalent inorganic cation homeostasis |
| GO:0032844 | 0.125 | 3.400 | 1 | 2 | 80 | regulation of homeostatic process |
| GO:0042180 | 0.125 | 1.691 | 5 | 8 | 655 | cellular ketone metabolic process |
| GO:0044271 | 0.127 | 2.165 | 2 | 4 | 252 | cellular nitrogen compound biosynthetic process |
| GO:0050878 | 0.127 | 2.518 | 1 | 3 | 162 | regulation of body fluid levels |
| GO:0044282 | 0.127 | 2.518 | 1 | 3 | 162 | small molecule catabolic process |
| GO:0005975 | 0.127 | 1.748 | 4 | 7 | 552 | carbohydrate metabolic process |
| GO:0048134 | 0.129 | 7.732 | 0 | 1 | 18 | germ-line cyst formation |
| GO:0033227 | 0.129 | 7.732 | 0 | 1 | 18 | dsRNA transport |
| GO:0016445 | 0.129 | 7.732 | 0 | 1 | 18 | somatic diversification of immunoglobulins |
| GO:0006575 | 0.130 | 3.314 | 1 | 2 | 82 | cellular modified amino acid metabolic process |
| GO:0012501 | 0.133 | 1.663 | 5 | 8 | 665 | programmed cell death |
| GO:0019730 | 0.135 | 3.233 | 1 | 2 | 84 | antimicrobial humoral response |
| GO:0006103 | 0.136 | 7.302 | 0 | 1 | 19 | 2-oxoglutarate metabolic process |
| GO:0006284 | 0.136 | 7.302 | 0 | 1 | 19 | base-excision repair |
| GO:0006513 | 0.136 | 7.302 | 0 | 1 | 19 | protein monoubiquitination |
| GO:0019852 | 0.136 | 7.302 | 0 | 1 | 19 | L-ascorbic acid metabolic process |
| GO:0002200 | 0.136 | 7.302 | 0 | 1 | 19 | somatic diversification of immune receptors |
| GO:0002807 | 0.136 | 7.302 | 0 | 1 | 19 | positive regulation of antimicrobial peptide biosynthetic process |
| GO:0002526 | 0.136 | 7.302 | 0 | 1 | 19 | acute inflammatory response |
| GO:0046834 | 0.136 | 7.302 | 0 | 1 | 19 | lipid phosphorylation |
| GO:0000289 | 0.136 | 7.302 | 0 | 1 | 19 | nuclear-transcribed mRNA poly(A) tail shortening |
| GO:0042078 | 0.136 | 7.302 | 0 | 1 | 19 | germ-line stem cell division |
| GO:0006013 | 0.136 | 7.302 | 0 | 1 | 19 | mannose metabolic process |
| GO:0016049 | 0.139 | 2.410 | 1 | 3 | 169 | cell growth |
| GO:0051270 | 0.141 | 2.395 | 1 | 3 | 170 | regulation of cellular component movement |
| GO:0006879 | 0.143 | 6.917 | 0 | 1 | 20 | cellular iron ion homeostasis |
| GO:0050832 | 0.143 | 6.917 | 0 | 1 | 20 | defense response to fungus |
| GO:0006901 | 0.143 | 6.917 | 0 | 1 | 20 | vesicle coating |
| GO:0007279 | 0.143 | 6.917 | 0 | 1 | 20 | pole cell formation |
| GO:0002377 | 0.143 | 6.917 | 0 | 1 | 20 | immunoglobulin production |
| GO:0051052 | 0.143 | 3.117 | 1 | 2 | 87 | regulation of DNA metabolic process |
| GO:0010721 | 0.143 | 3.117 | 1 | 2 | 87 | negative regulation of cell development |
| GO:0030001 | 0.144 | 2.366 | 1 | 3 | 172 | metal ion transport |
| GO:0006397 | 0.146 | 1.869 | 3 | 5 | 365 | mRNA processing |
| GO:0031399 | 0.146 | 1.869 | 3 | 5 | 365 | regulation of protein modification process |
| GO:0034644 | 0.149 | 6.570 | 0 | 1 | 21 | cellular response to UV |
| GO:0040035 | 0.149 | 6.570 | 0 | 1 | 21 | hermaphrodite genitalia development |
| GO:0045448 | 0.149 | 6.570 | 0 | 1 | 21 | mitotic cell cycle, embryonic |
| GO:0006921 | 0.149 | 6.570 | 0 | 1 | 21 | cellular component disassembly involved in apoptotic process |
| GO:0035329 | 0.149 | 6.570 | 0 | 1 | 21 | hippo signaling cascade |
| GO:0007029 | 0.149 | 6.570 | 0 | 1 | 21 | endoplasmic reticulum organization |
| GO:0035195 | 0.149 | 6.570 | 0 | 1 | 21 | gene silencing by miRNA |
| GO:0009620 | 0.149 | 6.570 | 0 | 1 | 21 | response to fungus |
| GO:0007291 | 0.149 | 6.570 | 0 | 1 | 21 | sperm individualization |
| GO:0007588 | 0.149 | 6.570 | 0 | 1 | 21 | excretion |
| GO:0044273 | 0.149 | 6.570 | 0 | 1 | 21 | sulfur compound catabolic process |
| GO:0033057 | 0.151 | 3.010 | 1 | 2 | 90 | multicellular organismal reproductive behavior |
| GO:0042402 | 0.156 | 6.257 | 0 | 1 | 22 | cellular biogenic amine catabolic process |
| GO:0021675 | 0.156 | 6.257 | 0 | 1 | 22 | nerve development |
| GO:0042133 | 0.156 | 6.257 | 0 | 1 | 22 | neurotransmitter metabolic process |
| GO:0009310 | 0.156 | 6.257 | 0 | 1 | 22 | amine catabolic process |
| GO:0090100 | 0.156 | 6.257 | 0 | 1 | 22 | positive regulation of transmembrane receptor protein serine/threonine kinase signaling pathway |
| GO:0001932 | 0.157 | 1.983 | 2 | 4 | 274 | regulation of protein phosphorylation |
| GO:0048477 | 0.161 | 1.806 | 3 | 5 | 377 | oogenesis |
| GO:0016137 | 0.162 | 5.971 | 0 | 1 | 23 | glycoside metabolic process |
| GO:0016236 | 0.162 | 5.971 | 0 | 1 | 23 | macroautophagy |
| GO:0006379 | 0.162 | 5.971 | 0 | 1 | 23 | mRNA cleavage |
| GO:0045766 | 0.162 | 5.971 | 0 | 1 | 23 | positive regulation of angiogenesis |
| GO:0035690 | 0.162 | 5.971 | 0 | 1 | 23 | cellular response to drug |
| GO:0007277 | 0.162 | 5.971 | 0 | 1 | 23 | pole cell development |
| GO:0000245 | 0.162 | 5.971 | 0 | 1 | 23 | spliceosomal complex assembly |
| GO:0008285 | 0.164 | 2.219 | 1 | 3 | 183 | negative regulation of cell proliferation |
| GO:0045927 | 0.167 | 2.816 | 1 | 2 | 96 | positive regulation of growth |
| GO:0000578 | 0.167 | 2.816 | 1 | 2 | 96 | embryonic axis specification |
| GO:0042981 | 0.167 | 1.781 | 3 | 5 | 382 | regulation of apoptotic process |
| GO:0042476 | 0.169 | 5.711 | 0 | 1 | 24 | odontogenesis |
| GO:0006305 | 0.169 | 5.711 | 0 | 1 | 24 | DNA alkylation |
| GO:0006306 | 0.169 | 5.711 | 0 | 1 | 24 | DNA methylation |
| GO:0016574 | 0.169 | 5.711 | 0 | 1 | 24 | histone ubiquitination |
| GO:0006536 | 0.169 | 5.711 | 0 | 1 | 24 | glutamate metabolic process |
| GO:0006900 | 0.169 | 5.711 | 0 | 1 | 24 | membrane budding |
| GO:0006903 | 0.169 | 5.711 | 0 | 1 | 24 | vesicle targeting |
| GO:0015837 | 0.169 | 5.711 | 0 | 1 | 24 | amine transport |
| GO:0019094 | 0.175 | 5.472 | 0 | 1 | 25 | pole plasm mRNA localization |
| GO:0060810 | 0.175 | 5.472 | 0 | 1 | 25 | intracellular mRNA localization involved in pattern specification process |
| GO:0060811 | 0.175 | 5.472 | 0 | 1 | 25 | intracellular mRNA localization involved in anterior/posterior axis specification |
| GO:0006506 | 0.175 | 5.472 | 0 | 1 | 25 | GPI anchor biosynthetic process |
| GO:0071482 | 0.175 | 5.472 | 0 | 1 | 25 | cellular response to light stimulus |
| GO:0006401 | 0.175 | 2.728 | 1 | 2 | 99 | RNA catabolic process |
| GO:0016458 | 0.175 | 2.728 | 1 | 2 | 99 | gene silencing |
| GO:0044265 | 0.176 | 1.747 | 3 | 5 | 389 | cellular macromolecule catabolic process |
| GO:0007292 | 0.179 | 1.738 | 3 | 5 | 391 | female gamete generation |
| GO:0007316 | 0.181 | 5.253 | 0 | 1 | 26 | pole plasm RNA localization |
| GO:0051053 | 0.181 | 5.253 | 0 | 1 | 26 | negative regulation of DNA metabolic process |
| GO:0010951 | 0.181 | 5.253 | 0 | 1 | 26 | negative regulation of endopeptidase activity |
| GO:0008219 | 0.183 | 1.523 | 6 | 8 | 720 | cell death |
| GO:0043066 | 0.183 | 2.099 | 1 | 3 | 193 | negative regulation of apoptotic process |
| GO:0016265 | 0.184 | 1.518 | 6 | 8 | 722 | death |
| GO:0016246 | 0.188 | 5.050 | 0 | 1 | 27 | RNA interference |
| GO:0001889 | 0.188 | 5.050 | 0 | 1 | 27 | liver development |
| GO:0001952 | 0.188 | 5.050 | 0 | 1 | 27 | regulation of cell-matrix adhesion |
| GO:0030510 | 0.188 | 5.050 | 0 | 1 | 27 | regulation of BMP signaling pathway |
| GO:0010466 | 0.188 | 5.050 | 0 | 1 | 27 | negative regulation of peptidase activity |
| GO:0000041 | 0.188 | 5.050 | 0 | 1 | 27 | transition metal ion transport |
| GO:0046667 | 0.188 | 5.050 | 0 | 1 | 27 | compound eye retinal cell programmed cell death |
| GO:0031646 | 0.188 | 5.050 | 0 | 1 | 27 | positive regulation of neurological system process |
| GO:0043067 | 0.188 | 1.705 | 3 | 5 | 398 | regulation of programmed cell death |
| GO:0043069 | 0.193 | 2.044 | 2 | 3 | 198 | negative regulation of programmed cell death |
| GO:0042364 | 0.194 | 4.863 | 0 | 1 | 28 | water-soluble vitamin biosynthetic process |
| GO:0050730 | 0.194 | 4.863 | 0 | 1 | 28 | regulation of peptidyl-tyrosine phosphorylation |
| GO:0006505 | 0.194 | 4.863 | 0 | 1 | 28 | GPI anchor metabolic process |
| GO:0061008 | 0.194 | 4.863 | 0 | 1 | 28 | hepaticobiliary system development |
| GO:0046666 | 0.194 | 4.863 | 0 | 1 | 28 | retinal cell programmed cell death |
| GO:0009266 | 0.195 | 2.542 | 1 | 2 | 106 | response to temperature stimulus |
| GO:0042325 | 0.197 | 1.803 | 2 | 4 | 300 | regulation of phosphorylation |
| GO:0042060 | 0.199 | 2.012 | 2 | 3 | 201 | wound healing |
| GO:0045610 | 0.200 | 4.688 | 0 | 1 | 29 | regulation of hemocyte differentiation |
| GO:0090090 | 0.200 | 4.688 | 0 | 1 | 29 | negative regulation of canonical Wnt receptor signaling pathway |
| GO:0002784 | 0.200 | 4.688 | 0 | 1 | 29 | regulation of antimicrobial peptide production |
| GO:0002775 | 0.200 | 4.688 | 0 | 1 | 29 | antimicrobial peptide production |
| GO:0002777 | 0.200 | 4.688 | 0 | 1 | 29 | antimicrobial peptide biosynthetic process |
| GO:0002805 | 0.200 | 4.688 | 0 | 1 | 29 | regulation of antimicrobial peptide biosynthetic process |
| GO:0031400 | 0.200 | 2.493 | 1 | 2 | 108 | negative regulation of protein modification process |
| GO:0044092 | 0.201 | 2.002 | 2 | 3 | 202 | negative regulation of molecular function |
| GO:0019098 | 0.203 | 2.470 | 1 | 2 | 109 | reproductive behavior |
| GO:0050769 | 0.206 | 4.526 | 0 | 1 | 30 | positive regulation of neurogenesis |
| GO:0040010 | 0.206 | 4.526 | 0 | 1 | 30 | positive regulation of growth rate |
| GO:0050680 | 0.206 | 4.526 | 0 | 1 | 30 | negative regulation of epithelial cell proliferation |
| GO:0030307 | 0.206 | 4.526 | 0 | 1 | 30 | positive regulation of cell growth |
| GO:0000096 | 0.206 | 4.526 | 0 | 1 | 30 | sulfur amino acid metabolic process |
| GO:0010941 | 0.211 | 1.631 | 3 | 5 | 415 | regulation of cell death |
| GO:0040012 | 0.211 | 1.952 | 2 | 3 | 207 | regulation of locomotion |
| GO:0009110 | 0.212 | 4.375 | 0 | 1 | 31 | vitamin biosynthetic process |
| GO:0002759 | 0.212 | 4.375 | 0 | 1 | 31 | regulation of antimicrobial humoral response |
| GO:0051650 | 0.212 | 4.375 | 0 | 1 | 31 | establishment of vesicle localization |
| GO:0008039 | 0.212 | 4.375 | 0 | 1 | 31 | synaptic target recognition |
| GO:0010506 | 0.212 | 4.375 | 0 | 1 | 31 | regulation of autophagy |
| GO:0055072 | 0.212 | 4.375 | 0 | 1 | 31 | iron ion homeostasis |
| GO:0045926 | 0.214 | 2.380 | 1 | 2 | 113 | negative regulation of growth |
| GO:0060548 | 0.217 | 1.923 | 2 | 3 | 210 | negative regulation of cell death |
| GO:0055065 | 0.217 | 2.358 | 1 | 2 | 114 | metal ion homeostasis |
| GO:0006282 | 0.218 | 4.233 | 0 | 1 | 32 | regulation of DNA repair |
| GO:0040009 | 0.218 | 4.233 | 0 | 1 | 32 | regulation of growth rate |
| GO:0050919 | 0.218 | 4.233 | 0 | 1 | 32 | negative chemotaxis |
| GO:0071705 | 0.218 | 4.233 | 0 | 1 | 32 | nitrogen compound transport |
| GO:0007398 | 0.218 | 4.233 | 0 | 1 | 32 | ectoderm development |
| GO:0007367 | 0.218 | 4.233 | 0 | 1 | 32 | segment polarity determination |
| GO:0045930 | 0.218 | 4.233 | 0 | 1 | 32 | negative regulation of mitotic cell cycle |
| GO:0051438 | 0.218 | 4.233 | 0 | 1 | 32 | regulation of ubiquitin-protein ligase activity |
| GO:0051289 | 0.218 | 4.233 | 0 | 1 | 32 | protein homotetramerization |
| GO:0046425 | 0.218 | 4.233 | 0 | 1 | 32 | regulation of JAK-STAT cascade |
| GO:0006259 | 0.220 | 1.441 | 6 | 8 | 757 | DNA metabolic process |
| GO:0006650 | 0.220 | 2.337 | 1 | 2 | 115 | glycerophospholipid metabolic process |
| GO:0042063 | 0.223 | 2.316 | 1 | 2 | 116 | gliogenesis |
| GO:0070972 | 0.224 | 4.100 | 0 | 1 | 33 | protein localization to endoplasmic reticulum |
| GO:0006308 | 0.224 | 4.100 | 0 | 1 | 33 | DNA catabolic process |
| GO:0007162 | 0.224 | 4.100 | 0 | 1 | 33 | negative regulation of cell adhesion |
| GO:0051340 | 0.224 | 4.100 | 0 | 1 | 33 | regulation of ligase activity |
| GO:0043687 | 0.224 | 4.100 | 0 | 1 | 33 | post-translational protein modification |
| GO:0007178 | 0.231 | 2.256 | 1 | 2 | 119 | transmembrane receptor protein serine/threonine kinase signaling pathway |
| GO:0007611 | 0.231 | 2.256 | 1 | 2 | 119 | learning or memory |
| GO:0045786 | 0.233 | 1.849 | 2 | 3 | 218 | negative regulation of cell cycle |
| GO:0006304 | 0.236 | 3.858 | 0 | 1 | 35 | DNA modification |
| GO:0006497 | 0.236 | 3.858 | 0 | 1 | 35 | protein lipidation |
| GO:0042158 | 0.236 | 3.858 | 0 | 1 | 35 | lipoprotein biosynthetic process |
| GO:0050900 | 0.236 | 3.858 | 0 | 1 | 35 | leukocyte migration |
| GO:0043409 | 0.236 | 3.858 | 0 | 1 | 35 | negative regulation of MAPK cascade |
| GO:0046621 | 0.236 | 3.858 | 0 | 1 | 35 | negative regulation of organ growth |
| GO:0002920 | 0.236 | 3.858 | 0 | 1 | 35 | regulation of humoral immune response |
| GO:0010623 | 0.236 | 3.858 | 0 | 1 | 35 | developmental programmed cell death |
| GO:0001505 | 0.237 | 2.217 | 1 | 2 | 121 | regulation of neurotransmitter levels |
| GO:0016071 | 0.239 | 1.551 | 3 | 5 | 435 | mRNA metabolic process |
| GO:0050890 | 0.240 | 2.199 | 1 | 2 | 122 | cognition |
| GO:0009066 | 0.242 | 3.747 | 0 | 1 | 36 | aspartate family amino acid metabolic process |
| GO:0001707 | 0.242 | 3.747 | 0 | 1 | 36 | mesoderm formation |
| GO:0048332 | 0.242 | 3.747 | 0 | 1 | 36 | mesoderm morphogenesis |
| GO:0030509 | 0.242 | 3.747 | 0 | 1 | 36 | BMP signaling pathway |
| GO:0015698 | 0.242 | 3.747 | 0 | 1 | 36 | inorganic anion transport |
| GO:0032101 | 0.242 | 2.180 | 1 | 2 | 123 | regulation of response to external stimulus |
| GO:0006275 | 0.248 | 3.643 | 0 | 1 | 37 | regulation of DNA replication |
| GO:0051048 | 0.248 | 3.643 | 0 | 1 | 37 | negative regulation of secretion |
| GO:0002700 | 0.248 | 3.643 | 0 | 1 | 37 | regulation of production of molecular mediator of immune response |
| GO:0000079 | 0.248 | 3.643 | 0 | 1 | 37 | regulation of cyclin-dependent protein kinase activity |
| GO:0005977 | 0.248 | 3.643 | 0 | 1 | 37 | glycogen metabolic process |
| GO:0048583 | 0.249 | 1.337 | 8 | 10 | 1020 | regulation of response to stimulus |
| GO:0016310 | 0.252 | 1.518 | 3 | 5 | 444 | phosphorylation |
| GO:0040008 | 0.252 | 1.610 | 3 | 4 | 334 | regulation of growth |
| GO:0006396 | 0.252 | 1.455 | 4 | 6 | 557 | RNA processing |
| GO:0006066 | 0.254 | 1.765 | 2 | 3 | 228 | alcohol metabolic process |
| GO:0042386 | 0.254 | 3.544 | 0 | 1 | 38 | hemocyte differentiation |
| GO:0007365 | 0.254 | 3.544 | 0 | 1 | 38 | periodic partitioning |
| GO:0000288 | 0.254 | 3.544 | 0 | 1 | 38 | nuclear-transcribed mRNA catabolic process, deadenylation-dependent decay |
| GO:0006073 | 0.254 | 3.544 | 0 | 1 | 38 | cellular glucan metabolic process |
| GO:0006091 | 0.256 | 1.757 | 2 | 3 | 229 | generation of precursor metabolites and energy |
| GO:0009416 | 0.257 | 2.092 | 1 | 2 | 128 | response to light stimulus |
| GO:0019751 | 0.259 | 3.450 | 0 | 1 | 39 | polyol metabolic process |
| GO:0045132 | 0.259 | 3.450 | 0 | 1 | 39 | meiotic chromosome segregation |
| GO:0071478 | 0.259 | 3.450 | 0 | 1 | 39 | cellular response to radiation |
| GO:0007315 | 0.259 | 3.450 | 0 | 1 | 39 | pole plasm assembly |
| GO:0007259 | 0.259 | 3.450 | 0 | 1 | 39 | JAK-STAT cascade |
| GO:0071901 | 0.259 | 3.450 | 0 | 1 | 39 | negative regulation of protein serine/threonine kinase activity |
| GO:0044042 | 0.259 | 3.450 | 0 | 1 | 39 | glucan metabolic process |
| GO:0010720 | 0.259 | 3.450 | 0 | 1 | 39 | positive regulation of cell development |
| GO:0008298 | 0.259 | 3.450 | 0 | 1 | 39 | intracellular mRNA localization |
| GO:0007350 | 0.262 | 2.059 | 1 | 2 | 130 | blastoderm segmentation |
| GO:0021782 | 0.265 | 3.361 | 0 | 1 | 40 | glial cell development |
| GO:0006888 | 0.265 | 3.361 | 0 | 1 | 40 | ER to Golgi vesicle-mediated transport |
| GO:0040003 | 0.265 | 3.361 | 0 | 1 | 40 | chitin-based cuticle development |
| GO:0009311 | 0.265 | 3.361 | 0 | 1 | 40 | oligosaccharide metabolic process |
| GO:0007427 | 0.265 | 3.361 | 0 | 1 | 40 | epithelial cell migration, open tracheal system |
| GO:0007507 | 0.268 | 1.710 | 2 | 3 | 235 | heart development |
| GO:0000910 | 0.268 | 2.027 | 1 | 2 | 132 | cytokinesis |
| GO:0042552 | 0.271 | 3.277 | 0 | 1 | 41 | myelination |
| GO:0019882 | 0.271 | 3.277 | 0 | 1 | 41 | antigen processing and presentation |
| GO:0000165 | 0.271 | 2.011 | 1 | 2 | 133 | MAPK cascade |
| GO:0002682 | 0.272 | 1.695 | 2 | 3 | 237 | regulation of immune system process |
| GO:0042493 | 0.274 | 1.996 | 1 | 2 | 134 | response to drug |
| GO:0035220 | 0.274 | 1.688 | 2 | 3 | 238 | wing disc development |
| GO:0019220 | 0.276 | 1.542 | 3 | 4 | 348 | regulation of phosphate metabolic process |
| GO:0006342 | 0.276 | 3.197 | 0 | 1 | 42 | chromatin silencing |
| GO:0048066 | 0.276 | 3.197 | 0 | 1 | 42 | developmental pigmentation |
| GO:0045765 | 0.276 | 3.197 | 0 | 1 | 42 | regulation of angiogenesis |
| GO:0007028 | 0.276 | 3.197 | 0 | 1 | 42 | cytoplasm organization |
| GO:0035194 | 0.276 | 3.197 | 0 | 1 | 42 | posttranscriptional gene silencing by RNA |
| GO:0010810 | 0.276 | 3.197 | 0 | 1 | 42 | regulation of cell-substrate adhesion |
| GO:0016441 | 0.276 | 3.197 | 0 | 1 | 42 | posttranscriptional gene silencing |
| GO:0015980 | 0.277 | 1.981 | 1 | 2 | 135 | energy derivation by oxidation of organic compounds |
| GO:0051174 | 0.278 | 1.537 | 3 | 4 | 349 | regulation of phosphorus metabolic process |
| GO:0006767 | 0.282 | 3.120 | 0 | 1 | 43 | water-soluble vitamin metabolic process |
| GO:0006661 | 0.282 | 3.120 | 0 | 1 | 43 | phosphatidylinositol biosynthetic process |
| GO:0022407 | 0.282 | 3.120 | 0 | 1 | 43 | regulation of cell-cell adhesion |
| GO:0017145 | 0.282 | 3.120 | 0 | 1 | 43 | stem cell division |
| GO:0051495 | 0.282 | 3.120 | 0 | 1 | 43 | positive regulation of cytoskeleton organization |
| GO:0072358 | 0.283 | 1.523 | 3 | 4 | 352 | cardiovascular system development |
| GO:0072359 | 0.283 | 1.523 | 3 | 4 | 352 | circulatory system development |
| GO:0006511 | 0.285 | 1.651 | 2 | 3 | 243 | ubiquitin-dependent protein catabolic process |
| GO:0006812 | 0.287 | 1.644 | 2 | 3 | 244 | cation transport |
| GO:0019941 | 0.287 | 1.644 | 2 | 3 | 244 | modification-dependent protein catabolic process |
| GO:0007166 | 0.287 | 1.299 | 7 | 9 | 938 | cell surface receptor signaling pathway |
| GO:0018988 | 0.288 | 3.047 | 0 | 1 | 44 | molting cycle, protein-based cuticle |
| GO:0009628 | 0.292 | 1.425 | 4 | 5 | 471 | response to abiotic stimulus |
| GO:0001704 | 0.293 | 2.978 | 0 | 1 | 45 | formation of primary germ layer |
| GO:0045814 | 0.293 | 2.978 | 0 | 1 | 45 | negative regulation of gene expression, epigenetic |
| GO:0007293 | 0.293 | 2.978 | 0 | 1 | 45 | germarium-derived egg chamber formation |
| GO:0009267 | 0.298 | 2.911 | 0 | 1 | 46 | cellular response to starvation |
| GO:0010741 | 0.298 | 2.911 | 0 | 1 | 46 | negative regulation of intracellular protein kinase cascade |
| GO:0043632 | 0.299 | 1.603 | 2 | 3 | 250 | modification-dependent macromolecule catabolic process |
| GO:0055080 | 0.302 | 1.853 | 1 | 2 | 144 | cation homeostasis |
| GO:2001020 | 0.304 | 2.847 | 0 | 1 | 47 | regulation of response to DNA damage stimulus |
| GO:0009451 | 0.304 | 2.847 | 0 | 1 | 47 | RNA modification |
| GO:0050678 | 0.304 | 2.847 | 0 | 1 | 47 | regulation of epithelial cell proliferation |
| GO:0043279 | 0.304 | 2.847 | 0 | 1 | 47 | response to alkaloid |
| GO:0043043 | 0.309 | 2.787 | 0 | 1 | 48 | peptide biosynthetic process |
| GO:0046356 | 0.309 | 2.787 | 0 | 1 | 48 | acetyl-CoA catabolic process |
| GO:0006099 | 0.309 | 2.787 | 0 | 1 | 48 | tricarboxylic acid cycle |
| GO:0050801 | 0.310 | 1.570 | 2 | 3 | 255 | ion homeostasis |
| GO:0030308 | 0.315 | 2.728 | 0 | 1 | 49 | negative regulation of cell growth |
| GO:0051648 | 0.315 | 2.728 | 0 | 1 | 49 | vesicle localization |
| GO:0031124 | 0.315 | 2.728 | 0 | 1 | 49 | mRNA 3'-end processing |
| GO:0046486 | 0.316 | 1.789 | 1 | 2 | 149 | glycerolipid metabolic process |
| GO:0015672 | 0.316 | 1.789 | 1 | 2 | 149 | monovalent inorganic cation transport |
| GO:0034637 | 0.320 | 2.672 | 0 | 1 | 50 | cellular carbohydrate biosynthetic process |
| GO:0006112 | 0.320 | 2.672 | 0 | 1 | 50 | energy reserve metabolic process |
| GO:0035172 | 0.320 | 2.672 | 0 | 1 | 50 | hemocyte proliferation |
| GO:0010389 | 0.320 | 2.672 | 0 | 1 | 50 | regulation of G2/M transition of mitotic cell cycle |
| GO:0051603 | 0.321 | 1.539 | 2 | 3 | 260 | proteolysis involved in cellular protein catabolic process |
| GO:0006468 | 0.321 | 1.429 | 3 | 4 | 374 | protein phosphorylation |
| GO:0055114 | 0.323 | 1.533 | 2 | 3 | 261 | oxidation-reduction process |
| GO:0050821 | 0.325 | 2.618 | 0 | 1 | 51 | protein stabilization |
| GO:0022404 | 0.325 | 2.618 | 0 | 1 | 51 | molting cycle process |
| GO:0007616 | 0.325 | 2.618 | 0 | 1 | 51 | long-term memory |
| GO:0018108 | 0.325 | 2.618 | 0 | 1 | 51 | peptidyl-tyrosine phosphorylation |
| GO:0007155 | 0.328 | 1.351 | 4 | 5 | 495 | cell adhesion |
| GO:0044257 | 0.329 | 1.515 | 2 | 3 | 264 | cellular protein catabolic process |
| GO:0051239 | 0.330 | 1.257 | 7 | 8 | 855 | regulation of multicellular organismal process |
| GO:0060828 | 0.330 | 2.567 | 0 | 1 | 52 | regulation of canonical Wnt receptor signaling pathway |
| GO:0021915 | 0.330 | 2.567 | 0 | 1 | 52 | neural tube development |
| GO:0009109 | 0.330 | 2.567 | 0 | 1 | 52 | coenzyme catabolic process |
| GO:0007314 | 0.330 | 2.567 | 0 | 1 | 52 | oocyte anterior/posterior axis specification |
| GO:0043281 | 0.330 | 2.567 | 0 | 1 | 52 | regulation of cysteine-type endopeptidase activity involved in apoptotic process |
| GO:0002831 | 0.330 | 2.567 | 0 | 1 | 52 | regulation of response to biotic stimulus |
| GO:0018212 | 0.330 | 2.567 | 0 | 1 | 52 | peptidyl-tyrosine modification |
| GO:0007272 | 0.335 | 2.517 | 0 | 1 | 53 | ensheathment of neurons |
| GO:0008366 | 0.335 | 2.517 | 0 | 1 | 53 | axon ensheathment |
| GO:0008358 | 0.335 | 2.517 | 0 | 1 | 53 | maternal determination of anterior/posterior axis, embryo |
| GO:0016567 | 0.337 | 1.491 | 2 | 3 | 268 | protein ubiquitination |
| GO:0044248 | 0.338 | 1.216 | 8 | 10 | 1107 | cellular catabolic process |
| GO:0048024 | 0.341 | 2.469 | 0 | 1 | 54 | regulation of nuclear mRNA splicing, via spliceosome |
| GO:0030203 | 0.341 | 2.469 | 0 | 1 | 54 | glycosaminoglycan metabolic process |
| GO:0051187 | 0.341 | 2.469 | 0 | 1 | 54 | cofactor catabolic process |
| GO:0051262 | 0.341 | 2.469 | 0 | 1 | 54 | protein tetramerization |
| GO:0022610 | 0.341 | 1.328 | 4 | 5 | 503 | biological adhesion |
| GO:0006403 | 0.342 | 1.684 | 1 | 2 | 158 | RNA localization |
| GO:0046620 | 0.346 | 2.423 | 0 | 1 | 55 | regulation of organ growth |
| GO:0019226 | 0.346 | 1.374 | 3 | 4 | 388 | transmission of nerve impulse |
| GO:0016477 | 0.346 | 1.374 | 3 | 4 | 388 | cell migration |
| GO:0007610 | 0.347 | 1.316 | 4 | 5 | 507 | behavior |
| GO:0032269 | 0.350 | 1.651 | 1 | 2 | 161 | negative regulation of cellular protein metabolic process |
| GO:0035264 | 0.351 | 2.379 | 0 | 1 | 56 | multicellular organism growth |
| GO:0002119 | 0.351 | 2.379 | 0 | 1 | 56 | nematode larval development |
| GO:0008033 | 0.351 | 2.379 | 0 | 1 | 56 | tRNA processing |
| GO:0031047 | 0.351 | 2.379 | 0 | 1 | 56 | gene silencing by RNA |
| GO:0044264 | 0.351 | 2.379 | 0 | 1 | 56 | cellular polysaccharide metabolic process |
| GO:0044267 | 0.354 | 1.166 | 12 | 14 | 1621 | cellular protein metabolic process |
| GO:0048806 | 0.356 | 2.336 | 0 | 1 | 57 | genitalia development |
| GO:0044272 | 0.356 | 2.336 | 0 | 1 | 57 | sulfur compound biosynthetic process |
| GO:0016050 | 0.356 | 2.336 | 0 | 1 | 57 | vesicle organization |
| GO:2000116 | 0.356 | 2.336 | 0 | 1 | 57 | regulation of cysteine-type endopeptidase activity |
| GO:0040007 | 0.358 | 1.297 | 4 | 5 | 514 | growth |
| GO:0009060 | 0.361 | 2.295 | 0 | 1 | 58 | aerobic respiration |
| GO:0030178 | 0.361 | 2.295 | 0 | 1 | 58 | negative regulation of Wnt receptor signaling pathway |
| GO:0033205 | 0.361 | 2.295 | 0 | 1 | 58 | cell cycle cytokinesis |
| GO:0043900 | 0.361 | 2.295 | 0 | 1 | 58 | regulation of multi-organism process |
| GO:0008356 | 0.361 | 2.295 | 0 | 1 | 58 | asymmetric cell division |
| GO:0042048 | 0.361 | 2.295 | 0 | 1 | 58 | olfactory behavior |
| GO:0009057 | 0.364 | 1.286 | 4 | 5 | 518 | macromolecule catabolic process |
| GO:0000398 | 0.364 | 1.600 | 1 | 2 | 166 | nuclear mRNA splicing, via spliceosome |
| GO:0000377 | 0.364 | 1.600 | 1 | 2 | 166 | RNA splicing, via transesterification reactions with bulged adenosine as nucleophile |
| GO:0042335 | 0.366 | 2.255 | 0 | 1 | 59 | cuticle development |
| GO:0006820 | 0.366 | 2.255 | 0 | 1 | 59 | anion transport |
| GO:0007286 | 0.366 | 2.255 | 0 | 1 | 59 | spermatid development |
| GO:0031123 | 0.366 | 2.255 | 0 | 1 | 59 | RNA 3'-end processing |
| GO:0032268 | 0.366 | 1.284 | 4 | 5 | 519 | regulation of cellular protein metabolic process |
| GO:2000113 | 0.367 | 1.330 | 3 | 4 | 400 | negative regulation of cellular macromolecule biosynthetic process |
| GO:0032446 | 0.369 | 1.408 | 2 | 3 | 283 | protein modification by small protein conjugation |
| GO:0042787 | 0.370 | 2.217 | 0 | 1 | 60 | protein ubiquitination involved in ubiquitin-dependent protein catabolic process |
| GO:0001708 | 0.370 | 2.217 | 0 | 1 | 60 | cell fate specification |
| GO:0007635 | 0.370 | 2.217 | 0 | 1 | 60 | chemosensory behavior |
| GO:0008344 | 0.370 | 2.217 | 0 | 1 | 60 | adult locomotory behavior |
| GO:0035637 | 0.370 | 1.323 | 3 | 4 | 402 | multicellular organismal signaling |
| GO:0010558 | 0.372 | 1.320 | 3 | 4 | 403 | negative regulation of macromolecule biosynthetic process |
| GO:0007015 | 0.372 | 1.571 | 1 | 2 | 169 | actin filament organization |
| GO:0000375 | 0.372 | 1.571 | 1 | 2 | 169 | RNA splicing, via transesterification reactions |
| GO:0007224 | 0.375 | 2.179 | 0 | 1 | 61 | smoothened signaling pathway |
| GO:0045934 | 0.378 | 1.310 | 3 | 4 | 406 | negative regulation of nucleobase-containing compound metabolic process |
| GO:0051172 | 0.378 | 1.310 | 3 | 4 | 406 | negative regulation of nitrogen compound metabolic process |
| GO:0008361 | 0.380 | 2.143 | 0 | 1 | 62 | regulation of cell size |
| GO:0008380 | 0.382 | 1.378 | 2 | 3 | 289 | RNA splicing |
| GO:0060255 | 0.382 | 1.131 | 15 | 16 | 1905 | regulation of macromolecule metabolic process |
| GO:0051248 | 0.383 | 1.533 | 1 | 2 | 173 | negative regulation of protein metabolic process |
| GO:0070925 | 0.385 | 2.108 | 0 | 1 | 63 | organelle assembly |
| GO:0009064 | 0.385 | 2.108 | 0 | 1 | 63 | glutamine family amino acid metabolic process |
| GO:0071363 | 0.385 | 2.108 | 0 | 1 | 63 | cellular response to growth factor stimulus |
| GO:0051329 | 0.389 | 1.515 | 1 | 2 | 175 | interphase of mitotic cell cycle |
| GO:0042303 | 0.390 | 2.075 | 0 | 1 | 64 | molting cycle |
| GO:0042157 | 0.390 | 2.075 | 0 | 1 | 64 | lipoprotein metabolic process |
| GO:0048515 | 0.390 | 2.075 | 0 | 1 | 64 | spermatid differentiation |
| GO:0090092 | 0.390 | 2.075 | 0 | 1 | 64 | regulation of transmembrane receptor protein serine/threonine kinase signaling pathway |
| GO:0031396 | 0.390 | 2.075 | 0 | 1 | 64 | regulation of protein ubiquitination |
| GO:0070848 | 0.390 | 2.075 | 0 | 1 | 64 | response to growth factor stimulus |
| GO:0031669 | 0.390 | 2.075 | 0 | 1 | 64 | cellular response to nutrient levels |
| GO:0042327 | 0.394 | 1.497 | 1 | 2 | 177 | positive regulation of phosphorylation |
| GO:0010001 | 0.394 | 2.042 | 0 | 1 | 65 | glial cell differentiation |
| GO:0002697 | 0.394 | 2.042 | 0 | 1 | 65 | regulation of immune effector process |
| GO:0010948 | 0.394 | 2.042 | 0 | 1 | 65 | negative regulation of cell cycle process |
| GO:0051325 | 0.397 | 1.489 | 1 | 2 | 178 | interphase |
| GO:0007033 | 0.399 | 2.010 | 1 | 1 | 66 | vacuole organization |
| GO:0043484 | 0.399 | 2.010 | 1 | 1 | 66 | regulation of RNA splicing |
| GO:0043473 | 0.399 | 2.010 | 1 | 1 | 66 | pigmentation |
| GO:0052548 | 0.404 | 1.980 | 1 | 1 | 67 | regulation of endopeptidase activity |
| GO:0050684 | 0.408 | 1.950 | 1 | 1 | 68 | regulation of mRNA processing |
| GO:0009636 | 0.408 | 1.950 | 1 | 1 | 68 | response to toxin |
| GO:0006732 | 0.411 | 1.447 | 1 | 2 | 183 | coenzyme metabolic process |
| GO:0031327 | 0.411 | 1.247 | 3 | 4 | 425 | negative regulation of cellular biosynthetic process |
| GO:0051301 | 0.413 | 1.244 | 3 | 4 | 426 | cell division |
| GO:0052547 | 0.413 | 1.921 | 1 | 1 | 69 | regulation of peptidase activity |
| GO:0008593 | 0.413 | 1.921 | 1 | 1 | 69 | regulation of Notch signaling pathway |
| GO:0031668 | 0.413 | 1.921 | 1 | 1 | 69 | cellular response to extracellular stimulus |
| GO:0009314 | 0.413 | 1.438 | 1 | 2 | 184 | response to radiation |
| GO:0035282 | 0.413 | 1.438 | 1 | 2 | 184 | segmentation |
| GO:0007476 | 0.413 | 1.438 | 1 | 2 | 184 | imaginal disc-derived wing morphogenesis |
| GO:0002520 | 0.413 | 1.438 | 1 | 2 | 184 | immune system development |
| GO:0031324 | 0.415 | 1.203 | 4 | 5 | 551 | negative regulation of cellular metabolic process |
| GO:0034470 | 0.416 | 1.430 | 1 | 2 | 185 | ncRNA processing |
| GO:0009890 | 0.416 | 1.238 | 3 | 4 | 428 | negative regulation of biosynthetic process |
| GO:0031647 | 0.417 | 1.893 | 1 | 1 | 70 | regulation of protein stability |
| GO:0006644 | 0.419 | 1.422 | 1 | 2 | 186 | phospholipid metabolic process |
| GO:0007472 | 0.419 | 1.422 | 1 | 2 | 186 | wing disc morphogenesis |
| GO:0044283 | 0.421 | 1.289 | 2 | 3 | 308 | small molecule biosynthetic process |
| GO:0051346 | 0.422 | 1.866 | 1 | 1 | 71 | negative regulation of hydrolase activity |
| GO:0008049 | 0.422 | 1.866 | 1 | 1 | 71 | male courtship behavior |
| GO:0044057 | 0.424 | 1.407 | 1 | 2 | 188 | regulation of system process |
| GO:0003006 | 0.424 | 1.189 | 4 | 5 | 557 | developmental process involved in reproduction |
| GO:0000209 | 0.426 | 1.839 | 1 | 1 | 72 | protein polyubiquitination |
| GO:0051094 | 0.427 | 1.399 | 1 | 2 | 189 | positive regulation of developmental process |
| GO:0048870 | 0.428 | 1.217 | 3 | 4 | 435 | cell motility |
| GO:0050673 | 0.431 | 1.813 | 1 | 1 | 73 | epithelial cell proliferation |
| GO:0071496 | 0.431 | 1.813 | 1 | 1 | 73 | cellular response to external stimulus |
| GO:0006911 | 0.431 | 1.813 | 1 | 1 | 73 | phagocytosis, engulfment |
| GO:0060070 | 0.431 | 1.813 | 1 | 1 | 73 | canonical Wnt receptor signaling pathway |
| GO:0006811 | 0.434 | 1.263 | 2 | 3 | 314 | ion transport |
| GO:0008152 | 0.434 | 1.097 | 45 | 46 | 5857 | metabolic process |
| GO:0045937 | 0.434 | 1.377 | 1 | 2 | 192 | positive regulation of phosphate metabolic process |
| GO:0010562 | 0.434 | 1.377 | 1 | 2 | 192 | positive regulation of phosphorus metabolic process |
| GO:0019228 | 0.435 | 1.788 | 1 | 1 | 74 | regulation of action potential in neuron |
| GO:0051674 | 0.435 | 1.205 | 3 | 4 | 439 | localization of cell |
| GO:0045596 | 0.437 | 1.369 | 1 | 2 | 193 | negative regulation of cell differentiation |
| GO:0018130 | 0.437 | 1.369 | 1 | 2 | 193 | heterocycle biosynthetic process |
| GO:0060179 | 0.440 | 1.764 | 1 | 1 | 75 | male mating behavior |
| GO:0007613 | 0.440 | 1.764 | 1 | 1 | 75 | memory |
| GO:0016197 | 0.440 | 1.764 | 1 | 1 | 75 | endosomal transport |
| GO:0007160 | 0.444 | 1.740 | 1 | 1 | 76 | cell-matrix adhesion |
| GO:0051260 | 0.444 | 1.740 | 1 | 1 | 76 | protein homooligomerization |
| GO:2000027 | 0.444 | 1.740 | 1 | 1 | 76 | regulation of organ morphogenesis |
| GO:0006281 | 0.444 | 1.242 | 2 | 3 | 319 | DNA repair |
| GO:0048610 | 0.444 | 1.160 | 4 | 5 | 570 | cellular process involved in reproduction |
| GO:0048609 | 0.445 | 1.139 | 5 | 6 | 697 | multicellular organismal reproductive process |
| GO:0030163 | 0.446 | 1.238 | 2 | 3 | 320 | protein catabolic process |
| GO:0050767 | 0.448 | 1.340 | 2 | 2 | 197 | regulation of neurogenesis |
| GO:0035120 | 0.448 | 1.340 | 2 | 2 | 197 | post-embryonic appendage morphogenesis |
| GO:0007444 | 0.448 | 1.234 | 2 | 3 | 321 | imaginal disc development |
| GO:0071214 | 0.448 | 1.717 | 1 | 1 | 77 | cellular response to abiotic stimulus |
| GO:0035265 | 0.448 | 1.717 | 1 | 1 | 77 | organ growth |
| GO:0022900 | 0.448 | 1.717 | 1 | 1 | 77 | electron transport chain |
| GO:0046474 | 0.448 | 1.717 | 1 | 1 | 77 | glycerophospholipid biosynthetic process |
| GO:0000956 | 0.448 | 1.717 | 1 | 1 | 77 | nuclear-transcribed mRNA catabolic process |
| GO:0032504 | 0.450 | 1.133 | 5 | 6 | 700 | multicellular organism reproduction |
| GO:0010324 | 0.452 | 1.695 | 1 | 1 | 78 | membrane invagination |
| GO:0035114 | 0.455 | 1.320 | 2 | 2 | 200 | imaginal disc-derived appendage morphogenesis |
| GO:0000122 | 0.455 | 1.320 | 2 | 2 | 200 | negative regulation of transcription from RNA polymerase II promoter |
| GO:0048878 | 0.456 | 1.218 | 2 | 3 | 325 | chemical homeostasis |
| GO:0006475 | 0.457 | 1.673 | 1 | 1 | 79 | internal protein amino acid acetylation |
| GO:0007351 | 0.457 | 1.673 | 1 | 1 | 79 | tripartite regional subdivision |
| GO:0007619 | 0.457 | 1.673 | 1 | 1 | 79 | courtship behavior |
| GO:0008595 | 0.457 | 1.673 | 1 | 1 | 79 | anterior/posterior axis specification, embryo |
| GO:0045859 | 0.460 | 1.306 | 2 | 2 | 202 | regulation of protein kinase activity |
| GO:0048489 | 0.461 | 1.651 | 1 | 1 | 80 | synaptic vesicle transport |
| GO:0007254 | 0.461 | 1.651 | 1 | 1 | 80 | JNK cascade |
| GO:0007268 | 0.462 | 1.206 | 3 | 3 | 328 | synaptic transmission |
| GO:0048737 | 0.463 | 1.299 | 2 | 2 | 203 | imaginal disc-derived appendage development |
| GO:0006914 | 0.465 | 1.630 | 1 | 1 | 81 | autophagy |
| GO:0007276 | 0.467 | 1.128 | 4 | 5 | 585 | gamete generation |
| GO:0071844 | 0.468 | 1.099 | 6 | 7 | 841 | cellular component assembly at cellular level |
| GO:0050877 | 0.469 | 1.109 | 5 | 6 | 714 | neurological system process |
| GO:0045333 | 0.473 | 1.590 | 1 | 1 | 83 | cellular respiration |
| GO:0030198 | 0.473 | 1.590 | 1 | 1 | 83 | extracellular matrix organization |
| GO:0009056 | 0.477 | 1.065 | 10 | 11 | 1364 | catabolic process |
| GO:0043414 | 0.477 | 1.571 | 1 | 1 | 84 | macromolecule methylation |
| GO:0051403 | 0.477 | 1.571 | 1 | 1 | 84 | stress-activated MAPK cascade |
| GO:0006796 | 0.480 | 1.111 | 5 | 5 | 593 | phosphate-containing compound metabolic process |
| GO:0006793 | 0.481 | 1.109 | 5 | 5 | 594 | phosphorus metabolic process |
| GO:0006473 | 0.481 | 1.552 | 1 | 1 | 85 | protein acetylation |
| GO:0006402 | 0.485 | 1.534 | 1 | 1 | 86 | mRNA catabolic process |
| GO:0045216 | 0.485 | 1.534 | 1 | 1 | 86 | cell-cell junction organization |
| GO:0007422 | 0.485 | 1.534 | 1 | 1 | 86 | peripheral nervous system development |
| GO:0031098 | 0.485 | 1.534 | 1 | 1 | 86 | stress-activated protein kinase signaling cascade |
| GO:0043549 | 0.486 | 1.242 | 2 | 2 | 212 | regulation of kinase activity |
| GO:0006766 | 0.489 | 1.516 | 1 | 1 | 87 | vitamin metabolic process |
| GO:0006518 | 0.489 | 1.516 | 1 | 1 | 87 | peptide metabolic process |
| GO:0007617 | 0.489 | 1.516 | 1 | 1 | 87 | mating behavior |
| GO:0000086 | 0.489 | 1.516 | 1 | 1 | 87 | G2/M transition of mitotic cell cycle |
| GO:0051186 | 0.491 | 1.230 | 2 | 2 | 214 | cofactor metabolic process |
| GO:0003008 | 0.492 | 1.072 | 7 | 7 | 860 | system process |
| GO:0022613 | 0.493 | 1.224 | 2 | 2 | 215 | ribonucleoprotein complex biogenesis |
| GO:0032259 | 0.493 | 1.498 | 1 | 1 | 88 | methylation |
| GO:0051338 | 0.496 | 1.218 | 2 | 2 | 216 | regulation of transferase activity |
| GO:0010259 | 0.497 | 1.481 | 1 | 1 | 89 | multicellular organismal aging |
| GO:0022607 | 0.500 | 1.057 | 8 | 8 | 996 | cellular component assembly |
| GO:0048518 | 0.500 | 1.042 | 12 | 12 | 1516 | positive regulation of biological process |
| GO:0043543 | 0.501 | 1.464 | 1 | 1 | 90 | protein acylation |
| GO:0010638 | 0.501 | 1.464 | 1 | 1 | 90 | positive regulation of organelle organization |
| GO:0019953 | 0.503 | 1.079 | 5 | 5 | 609 | sexual reproduction |
| GO:0045892 | 0.504 | 1.130 | 3 | 3 | 349 | negative regulation of transcription, DNA-dependent |
| GO:0001709 | 0.505 | 1.447 | 1 | 1 | 91 | cell fate determination |
| GO:0007623 | 0.505 | 1.447 | 1 | 1 | 91 | circadian rhythm |
| GO:0007369 | 0.509 | 1.431 | 1 | 1 | 92 | gastrulation |
| GO:0009948 | 0.509 | 1.431 | 1 | 1 | 92 | anterior/posterior axis specification |
| GO:0010605 | 0.512 | 1.068 | 5 | 5 | 615 | negative regulation of macromolecule metabolic process |
| GO:0001508 | 0.516 | 1.400 | 1 | 1 | 94 | regulation of action potential |
| GO:0043062 | 0.516 | 1.400 | 1 | 1 | 94 | extracellular structure organization |
| GO:0030258 | 0.516 | 1.400 | 1 | 1 | 94 | lipid modification |
| GO:0010608 | 0.518 | 1.168 | 2 | 2 | 225 | posttranscriptional regulation of gene expression |
| GO:0090305 | 0.520 | 1.163 | 2 | 2 | 226 | nucleic acid phosphodiester bond hydrolysis |
| GO:0051051 | 0.520 | 1.385 | 1 | 1 | 95 | negative regulation of transport |
| GO:0007498 | 0.524 | 1.370 | 1 | 1 | 96 | mesoderm development |
| GO:0007167 | 0.527 | 1.091 | 3 | 3 | 361 | enzyme linked receptor protein signaling pathway |
| GO:0007267 | 0.527 | 1.065 | 4 | 4 | 493 | cell-cell signaling |
| GO:2000602 | 0.531 | 1.342 | 1 | 1 | 98 | regulation of interphase of mitotic cell cycle |
| GO:0001525 | 0.531 | 1.342 | 1 | 1 | 98 | angiogenesis |
| GO:0031589 | 0.531 | 1.342 | 1 | 1 | 98 | cell-substrate adhesion |
| GO:0007416 | 0.535 | 1.328 | 1 | 1 | 99 | synapse assembly |
| GO:0007618 | 0.535 | 1.328 | 1 | 1 | 99 | mating |
| GO:0006873 | 0.537 | 1.126 | 2 | 2 | 233 | cellular ion homeostasis |
| GO:0048563 | 0.537 | 1.126 | 2 | 2 | 233 | post-embryonic organ morphogenesis |
| GO:0007560 | 0.537 | 1.126 | 2 | 2 | 233 | imaginal disc morphogenesis |
| GO:0032956 | 0.538 | 1.314 | 1 | 1 | 100 | regulation of actin cytoskeleton organization |
| GO:0043408 | 0.538 | 1.314 | 1 | 1 | 100 | regulation of MAPK cascade |
| GO:0016051 | 0.542 | 1.301 | 1 | 1 | 101 | carbohydrate biosynthetic process |
| GO:0045017 | 0.546 | 1.288 | 1 | 1 | 102 | glycerolipid biosynthetic process |
| GO:0006875 | 0.546 | 1.288 | 1 | 1 | 102 | cellular metal ion homeostasis |
| GO:0051656 | 0.546 | 1.288 | 1 | 1 | 102 | establishment of organelle localization |
| GO:0051960 | 0.548 | 1.102 | 2 | 2 | 238 | regulation of nervous system development |
| GO:0009892 | 0.549 | 1.023 | 5 | 5 | 640 | negative regulation of metabolic process |
| GO:0030111 | 0.549 | 1.275 | 1 | 1 | 103 | regulation of Wnt receptor signaling pathway |
| GO:0048519 | 0.551 | 1.001 | 11 | 11 | 1436 | negative regulation of biological process |
| GO:0006022 | 0.553 | 1.263 | 1 | 1 | 104 | aminoglycan metabolic process |
| GO:0051253 | 0.555 | 1.045 | 3 | 3 | 376 | negative regulation of RNA metabolic process |
| GO:0055082 | 0.555 | 1.088 | 2 | 2 | 241 | cellular chemical homeostasis |
| GO:0019222 | 0.558 | 0.993 | 17 | 17 | 2232 | regulation of metabolic process |
| GO:0071845 | 0.559 | 1.238 | 1 | 1 | 106 | cellular component disassembly at cellular level |
| GO:0051969 | 0.559 | 1.238 | 1 | 1 | 106 | regulation of transmission of nerve impulse |
| GO:0007420 | 0.562 | 1.074 | 2 | 2 | 244 | brain development |
| GO:0051726 | 0.564 | 1.030 | 3 | 3 | 381 | regulation of cell cycle |
| GO:0042127 | 0.565 | 1.027 | 3 | 3 | 382 | regulation of cell proliferation |
| GO:0035107 | 0.566 | 1.065 | 2 | 2 | 246 | appendage morphogenesis |
| GO:0071103 | 0.566 | 1.215 | 1 | 1 | 108 | DNA conformation change |
| GO:0022411 | 0.566 | 1.215 | 1 | 1 | 108 | cellular component disassembly |
| GO:0007219 | 0.566 | 1.215 | 1 | 1 | 108 | Notch signaling pathway |
| GO:0031644 | 0.566 | 1.215 | 1 | 1 | 108 | regulation of neurological system process |
| GO:0042594 | 0.570 | 1.204 | 1 | 1 | 109 | response to starvation |
| GO:0048193 | 0.570 | 1.204 | 1 | 1 | 109 | Golgi vesicle transport |
| GO:0043434 | 0.573 | 1.192 | 1 | 1 | 110 | response to peptide hormone stimulus |
| GO:0008654 | 0.573 | 1.192 | 1 | 1 | 110 | phospholipid biosynthetic process |
| GO:0019048 | 0.576 | 1.181 | 1 | 1 | 111 | virus-host interaction |
| GO:0006836 | 0.580 | 1.171 | 1 | 1 | 112 | neurotransmitter transport |
| GO:0006909 | 0.580 | 1.171 | 1 | 1 | 112 | phagocytosis |
| GO:0002164 | 0.580 | 1.171 | 1 | 1 | 112 | larval development |
| GO:0071843 | 0.582 | 1.034 | 2 | 2 | 253 | cellular component biogenesis at cellular level |
| GO:0048736 | 0.582 | 1.034 | 2 | 2 | 253 | appendage development |
| GO:0048569 | 0.586 | 1.026 | 2 | 2 | 255 | post-embryonic organ development |
| GO:0006399 | 0.586 | 1.150 | 1 | 1 | 114 | tRNA metabolic process |
| GO:0034660 | 0.588 | 1.021 | 2 | 2 | 256 | ncRNA metabolic process |
| GO:0030003 | 0.589 | 1.139 | 1 | 1 | 115 | cellular cation homeostasis |
| GO:0045597 | 0.589 | 1.139 | 1 | 1 | 115 | positive regulation of cell differentiation |
| GO:0009791 | 0.590 | 0.989 | 3 | 3 | 396 | post-embryonic development |
| GO:0031325 | 0.594 | 0.967 | 6 | 6 | 808 | positive regulation of cellular metabolic process |
| GO:0032970 | 0.596 | 1.119 | 1 | 1 | 117 | regulation of actin filament-based process |
| GO:0016072 | 0.596 | 1.119 | 1 | 1 | 117 | rRNA metabolic process |
| GO:0030155 | 0.599 | 1.110 | 1 | 1 | 118 | regulation of cell adhesion |
| GO:0030534 | 0.599 | 1.110 | 1 | 1 | 118 | adult behavior |
| GO:0050790 | 0.600 | 0.965 | 4 | 4 | 540 | regulation of catalytic activity |
| GO:0006790 | 0.602 | 1.100 | 1 | 1 | 119 | sulfur compound metabolic process |
| GO:0051701 | 0.602 | 1.100 | 1 | 1 | 119 | interaction with host |
| GO:0007243 | 0.603 | 0.993 | 2 | 2 | 263 | intracellular protein kinase cascade |
| GO:2000026 | 0.603 | 0.961 | 4 | 4 | 542 | regulation of multicellular organismal development |
| GO:0048707 | 0.607 | 0.985 | 2 | 2 | 265 | instar larval or pupal morphogenesis |
| GO:0071900 | 0.611 | 1.073 | 1 | 1 | 122 | regulation of protein serine/threonine kinase activity |
| GO:0007049 | 0.611 | 0.948 | 7 | 7 | 958 | cell cycle |
| GO:0051093 | 0.615 | 0.970 | 2 | 2 | 269 | negative regulation of developmental process |
| GO:0009886 | 0.617 | 0.966 | 2 | 2 | 270 | post-embryonic morphogenesis |
| GO:0040011 | 0.620 | 0.939 | 6 | 6 | 829 | locomotion |
| GO:0050657 | 0.620 | 1.046 | 1 | 1 | 125 | nucleic acid transport |
| GO:0050658 | 0.620 | 1.046 | 1 | 1 | 125 | RNA transport |
| GO:0051236 | 0.620 | 1.046 | 1 | 1 | 125 | establishment of RNA localization |
| GO:0000278 | 0.622 | 0.937 | 4 | 4 | 555 | mitotic cell cycle |
| GO:0034097 | 0.623 | 1.038 | 1 | 1 | 126 | response to cytokine stimulus |
| GO:0007552 | 0.624 | 0.955 | 2 | 2 | 273 | metamorphosis |
| GO:0048813 | 0.626 | 1.029 | 1 | 1 | 127 | dendrite morphogenesis |
| GO:0034330 | 0.626 | 1.029 | 1 | 1 | 127 | cell junction organization |
| GO:0060284 | 0.628 | 0.948 | 2 | 2 | 275 | regulation of cell development |
| GO:0051259 | 0.629 | 1.021 | 1 | 1 | 128 | protein oligomerization |
| GO:0080135 | 0.632 | 1.013 | 1 | 1 | 129 | regulation of cellular response to stress |
| GO:0048514 | 0.635 | 1.005 | 1 | 1 | 130 | blood vessel morphogenesis |
| GO:0018193 | 0.637 | 0.930 | 2 | 2 | 280 | peptidyl-amino acid modification |
| GO:0048511 | 0.637 | 0.997 | 1 | 1 | 131 | rhythmic process |
| GO:0008406 | 0.637 | 0.997 | 1 | 1 | 131 | gonad development |
| GO:0008283 | 0.639 | 0.916 | 4 | 4 | 567 | cell proliferation |
| GO:0051046 | 0.640 | 0.989 | 1 | 1 | 132 | regulation of secretion |
| GO:0008038 | 0.643 | 0.982 | 1 | 1 | 133 | neuron recognition |
| GO:0044238 | 0.646 | 0.941 | 41 | 40 | 5339 | primary metabolic process |
| GO:0005976 | 0.646 | 0.974 | 1 | 1 | 134 | polysaccharide metabolic process |
| GO:0065008 | 0.651 | 0.914 | 10 | 9 | 1268 | regulation of biological quality |
| GO:0080090 | 0.651 | 0.923 | 15 | 14 | 1944 | regulation of primary metabolic process |
| GO:0032535 | 0.651 | 0.960 | 1 | 1 | 136 | regulation of cellular component size |
| GO:0015931 | 0.651 | 0.960 | 1 | 1 | 136 | nucleobase-containing compound transport |
| GO:0045165 | 0.653 | 0.903 | 2 | 2 | 288 | cell fate commitment |
| GO:0000003 | 0.653 | 0.910 | 9 | 8 | 1133 | reproduction |
| GO:0010629 | 0.653 | 0.897 | 3 | 3 | 434 | negative regulation of gene expression |
| GO:0032879 | 0.653 | 0.899 | 4 | 4 | 577 | regulation of localization |
| GO:0048523 | 0.656 | 0.910 | 10 | 9 | 1273 | negative regulation of cellular process |
| GO:0009893 | 0.656 | 0.901 | 7 | 6 | 860 | positive regulation of metabolic process |
| GO:0016568 | 0.657 | 0.897 | 2 | 2 | 290 | chromatin modification |
| GO:0044419 | 0.665 | 0.925 | 1 | 1 | 141 | interspecies interaction between organisms |
| GO:0044403 | 0.665 | 0.925 | 1 | 1 | 141 | symbiosis, encompassing mutualism through parasitism |
| GO:0023052 | 0.665 | 0.913 | 15 | 14 | 1961 | signaling |
| GO:0008037 | 0.670 | 0.912 | 1 | 1 | 143 | cell recognition |
| GO:0001568 | 0.673 | 0.905 | 1 | 1 | 144 | blood vessel development |
| GO:0051640 | 0.673 | 0.905 | 1 | 1 | 144 | organelle localization |
| GO:0045595 | 0.675 | 0.868 | 3 | 3 | 448 | regulation of cell differentiation |
| GO:0009968 | 0.677 | 0.863 | 2 | 2 | 301 | negative regulation of signal transduction |
| GO:0022403 | 0.679 | 0.867 | 5 | 4 | 596 | cell cycle phase |
| GO:0007126 | 0.680 | 0.886 | 1 | 1 | 147 | meiosis |
| GO:0051327 | 0.680 | 0.886 | 1 | 1 | 147 | M phase of meiotic cell cycle |
| GO:0009605 | 0.680 | 0.872 | 6 | 5 | 740 | response to external stimulus |
| GO:0006974 | 0.680 | 0.859 | 3 | 3 | 452 | response to DNA damage stimulus |
| GO:0022402 | 0.683 | 0.869 | 6 | 5 | 742 | cell cycle process |
| GO:0002252 | 0.683 | 0.880 | 1 | 1 | 148 | immune effector process |
| GO:0009790 | 0.685 | 0.867 | 6 | 5 | 744 | embryo development |
| GO:0019725 | 0.686 | 0.848 | 2 | 2 | 306 | cellular homeostasis |
| GO:0006457 | 0.688 | 0.868 | 1 | 1 | 150 | protein folding |
| GO:0051240 | 0.688 | 0.868 | 1 | 1 | 150 | positive regulation of multicellular organismal process |
| GO:0044085 | 0.689 | 0.878 | 9 | 8 | 1169 | cellular component biogenesis |
| GO:0007059 | 0.690 | 0.862 | 1 | 1 | 151 | chromosome segregation |
| GO:0002165 | 0.691 | 0.839 | 2 | 2 | 309 | instar larval or pupal development |
| GO:0019318 | 0.692 | 0.856 | 1 | 1 | 152 | hexose metabolic process |
| GO:0001944 | 0.692 | 0.856 | 1 | 1 | 152 | vasculature development |
| GO:0050789 | 0.695 | 0.907 | 30 | 28 | 3856 | regulation of biological process |
| GO:0051254 | 0.695 | 0.840 | 4 | 3 | 462 | positive regulation of RNA metabolic process |
| GO:0016044 | 0.696 | 0.831 | 2 | 2 | 312 | cellular membrane organization |
| GO:0042391 | 0.697 | 0.845 | 1 | 1 | 154 | regulation of membrane potential |
| GO:0001751 | 0.697 | 0.845 | 1 | 1 | 154 | compound eye photoreceptor cell differentiation |
| GO:0016358 | 0.697 | 0.845 | 1 | 1 | 154 | dendrite development |
| GO:0061024 | 0.698 | 0.828 | 2 | 2 | 313 | membrane organization |
| GO:0006260 | 0.699 | 0.825 | 2 | 2 | 314 | DNA replication |
| GO:0006928 | 0.700 | 0.841 | 5 | 4 | 613 | cellular component movement |
| GO:0006897 | 0.701 | 0.823 | 2 | 2 | 315 | endocytosis |
| GO:0022603 | 0.701 | 0.823 | 2 | 2 | 315 | regulation of anatomical structure morphogenesis |
| GO:0051321 | 0.702 | 0.834 | 1 | 1 | 156 | meiotic cell cycle |
| GO:0016311 | 0.702 | 0.834 | 1 | 1 | 156 | dephosphorylation |
| GO:0050793 | 0.703 | 0.846 | 6 | 5 | 760 | regulation of developmental process |
| GO:0009952 | 0.704 | 0.828 | 1 | 1 | 157 | anterior/posterior pattern specification |
| GO:0030097 | 0.707 | 0.823 | 1 | 1 | 158 | hemopoiesis |
| GO:0007154 | 0.708 | 0.880 | 15 | 14 | 2015 | cell communication |
| GO:0007568 | 0.709 | 0.818 | 1 | 1 | 159 | aging |
| GO:0006325 | 0.713 | 0.804 | 2 | 2 | 322 | chromatin organization |
| GO:0034622 | 0.714 | 0.801 | 2 | 2 | 323 | cellular macromolecular complex assembly |
| GO:0065007 | 0.715 | 0.894 | 32 | 30 | 4147 | biological regulation |
| GO:0010927 | 0.716 | 0.802 | 1 | 1 | 162 | cellular component assembly involved in morphogenesis |
| GO:0030036 | 0.719 | 0.794 | 2 | 2 | 326 | actin cytoskeleton organization |
| GO:0043170 | 0.719 | 0.891 | 32 | 30 | 4154 | macromolecule metabolic process |
| GO:0045137 | 0.720 | 0.792 | 1 | 1 | 164 | development of primary sexual characteristics |
| GO:0022415 | 0.720 | 0.792 | 1 | 1 | 164 | viral reproductive process |
| GO:0048522 | 0.721 | 0.854 | 10 | 9 | 1343 | positive regulation of cellular process |
| GO:0001754 | 0.722 | 0.787 | 1 | 1 | 165 | eye photoreceptor cell differentiation |
| GO:0010467 | 0.723 | 0.876 | 19 | 17 | 2440 | gene expression |
| GO:0001934 | 0.724 | 0.782 | 1 | 1 | 166 | positive regulation of protein phosphorylation |
| GO:0051493 | 0.724 | 0.782 | 1 | 1 | 166 | regulation of cytoskeleton organization |
| GO:0010646 | 0.725 | 0.812 | 5 | 4 | 633 | regulation of cell communication |
| GO:0023057 | 0.725 | 0.784 | 3 | 2 | 330 | negative regulation of signaling |
| GO:0005996 | 0.731 | 0.768 | 1 | 1 | 169 | monosaccharide metabolic process |
| GO:0051705 | 0.733 | 0.763 | 1 | 1 | 170 | behavioral interaction between organisms |
| GO:0050808 | 0.735 | 0.759 | 1 | 1 | 171 | synapse organization |
| GO:0010648 | 0.736 | 0.767 | 3 | 2 | 337 | negative regulation of cell communication |
| GO:0048534 | 0.737 | 0.754 | 1 | 1 | 172 | hemopoietic or lymphoid organ development |
| GO:0001654 | 0.738 | 0.764 | 3 | 2 | 338 | eye development |
| GO:0030029 | 0.741 | 0.759 | 3 | 2 | 340 | actin filament-based process |
| GO:0065003 | 0.742 | 0.777 | 4 | 3 | 497 | macromolecular complex assembly |
| GO:0007050 | 0.743 | 0.741 | 1 | 1 | 175 | cell cycle arrest |
| GO:0016055 | 0.743 | 0.741 | 1 | 1 | 175 | Wnt receptor signaling pathway |
| GO:0048585 | 0.747 | 0.750 | 3 | 2 | 344 | negative regulation of response to stimulus |
| GO:0022414 | 0.747 | 0.817 | 8 | 7 | 1091 | reproductive process |
| GO:0042592 | 0.748 | 0.768 | 4 | 3 | 502 | homeostatic process |
| GO:0016570 | 0.749 | 0.728 | 1 | 1 | 178 | histone modification |
| GO:0043065 | 0.749 | 0.728 | 1 | 1 | 178 | positive regulation of apoptotic process |
| GO:0016569 | 0.751 | 0.724 | 1 | 1 | 179 | covalent chromatin modification |
| GO:0045935 | 0.752 | 0.763 | 4 | 3 | 505 | positive regulation of nucleobase-containing compound metabolic process |
| GO:0010468 | 0.756 | 0.827 | 12 | 10 | 1527 | regulation of gene expression |
| GO:0007626 | 0.757 | 0.712 | 1 | 1 | 182 | locomotory behavior |
| GO:0051173 | 0.758 | 0.755 | 4 | 3 | 510 | positive regulation of nitrogen compound metabolic process |
| GO:0046530 | 0.761 | 0.704 | 1 | 1 | 184 | photoreceptor cell differentiation |
| GO:0016192 | 0.761 | 0.769 | 5 | 4 | 666 | vesicle-mediated transport |
| GO:0007346 | 0.764 | 0.696 | 1 | 1 | 186 | regulation of mitotic cell cycle |
| GO:0007424 | 0.764 | 0.696 | 1 | 1 | 186 | open tracheal system development |
| GO:0007165 | 0.765 | 0.825 | 13 | 11 | 1678 | signal transduction |
| GO:0071310 | 0.767 | 0.719 | 3 | 2 | 358 | cellular response to organic substance |
| GO:0010627 | 0.770 | 0.685 | 1 | 1 | 189 | regulation of intracellular protein kinase cascade |
| GO:0048646 | 0.771 | 0.738 | 4 | 3 | 521 | anatomical structure formation involved in morphogenesis |
| GO:0048232 | 0.772 | 0.681 | 1 | 1 | 190 | male gamete generation |
| GO:0007283 | 0.772 | 0.681 | 1 | 1 | 190 | spermatogenesis |
| GO:0065009 | 0.773 | 0.755 | 5 | 4 | 677 | regulation of molecular function |
| GO:0043068 | 0.775 | 0.674 | 1 | 1 | 192 | positive regulation of programmed cell death |
| GO:0010942 | 0.777 | 0.670 | 1 | 1 | 193 | positive regulation of cell death |
| GO:0016337 | 0.779 | 0.666 | 1 | 1 | 194 | cell-cell adhesion |
| GO:0048729 | 0.786 | 0.689 | 3 | 2 | 373 | tissue morphogenesis |
| GO:0007548 | 0.789 | 0.646 | 2 | 1 | 200 | sex differentiation |
| GO:0031328 | 0.792 | 0.710 | 4 | 3 | 540 | positive regulation of cellular biosynthetic process |
| GO:0007417 | 0.793 | 0.679 | 3 | 2 | 378 | central nervous system development |
| GO:0051716 | 0.794 | 0.816 | 17 | 15 | 2277 | cellular response to stimulus |
| GO:0034621 | 0.798 | 0.672 | 3 | 2 | 382 | cellular macromolecular complex subunit organization |
| GO:0051252 | 0.798 | 0.775 | 10 | 8 | 1298 | regulation of RNA metabolic process |
| GO:0090066 | 0.800 | 0.623 | 2 | 1 | 207 | regulation of anatomical structure size |
| GO:0016032 | 0.800 | 0.623 | 2 | 1 | 207 | viral reproduction |
| GO:0031667 | 0.800 | 0.623 | 2 | 1 | 207 | response to nutrient levels |
| GO:0061458 | 0.803 | 0.617 | 2 | 1 | 209 | reproductive system development |
| GO:0048608 | 0.803 | 0.617 | 2 | 1 | 209 | reproductive structure development |
| GO:0006886 | 0.803 | 0.663 | 3 | 2 | 387 | intracellular protein transport |
| GO:0071704 | 0.805 | 0.716 | 5 | 4 | 710 | organic substance metabolic process |
| GO:0051130 | 0.805 | 0.614 | 2 | 1 | 210 | positive regulation of cellular component organization |
| GO:0009891 | 0.807 | 0.690 | 4 | 3 | 555 | positive regulation of biosynthetic process |
| GO:0043009 | 0.808 | 0.608 | 2 | 1 | 212 | chordate embryonic development |
| GO:0009991 | 0.808 | 0.608 | 2 | 1 | 212 | response to extracellular stimulus |
| GO:0001745 | 0.811 | 0.602 | 2 | 1 | 214 | compound eye morphogenesis |
| GO:0070727 | 0.816 | 0.678 | 4 | 3 | 564 | cellular macromolecule localization |
| GO:0033554 | 0.818 | 0.700 | 6 | 4 | 725 | cellular response to stress |
| GO:0044087 | 0.818 | 0.588 | 2 | 1 | 219 | regulation of cellular component biogenesis |
| GO:0042330 | 0.822 | 0.635 | 3 | 2 | 403 | taxis |
| GO:0043933 | 0.824 | 0.666 | 4 | 3 | 573 | macromolecular complex subunit organization |
| GO:0009792 | 0.828 | 0.625 | 3 | 2 | 409 | embryo development ending in birth or egg hatching |
| GO:0006810 | 0.830 | 0.766 | 14 | 11 | 1778 | transport |
| GO:0071702 | 0.835 | 0.557 | 2 | 1 | 231 | organic substance transport |
| GO:0007517 | 0.841 | 0.544 | 2 | 1 | 236 | muscle organ development |
| GO:0031401 | 0.842 | 0.542 | 2 | 1 | 237 | positive regulation of protein modification process |
| GO:0031329 | 0.845 | 0.537 | 2 | 1 | 239 | regulation of cellular catabolic process |
| GO:0019219 | 0.846 | 0.736 | 12 | 9 | 1517 | regulation of nucleobase-containing compound metabolic process |
| GO:0060541 | 0.846 | 0.535 | 2 | 1 | 240 | respiratory system development |
| GO:0010564 | 0.849 | 0.530 | 2 | 1 | 242 | regulation of cell cycle process |
| GO:0007423 | 0.849 | 0.593 | 3 | 2 | 430 | sensory organ development |
| GO:0010604 | 0.852 | 0.657 | 6 | 4 | 768 | positive regulation of macromolecule metabolic process |
| GO:0051234 | 0.853 | 0.743 | 14 | 11 | 1820 | establishment of localization |
| GO:0045944 | 0.853 | 0.522 | 2 | 1 | 246 | positive regulation of transcription from RNA polymerase II promoter |
| GO:0045893 | 0.853 | 0.586 | 3 | 2 | 435 | positive regulation of transcription, DNA-dependent |
| GO:0046394 | 0.856 | 0.517 | 2 | 1 | 248 | carboxylic acid biosynthetic process |
| GO:0016053 | 0.856 | 0.517 | 2 | 1 | 248 | organic acid biosynthetic process |
| GO:0048749 | 0.857 | 0.515 | 2 | 1 | 249 | compound eye development |
| GO:0006355 | 0.857 | 0.702 | 9 | 7 | 1241 | regulation of transcription, DNA-dependent |
| GO:0051171 | 0.857 | 0.724 | 12 | 9 | 1538 | regulation of nitrogen compound metabolic process |
| GO:2001141 | 0.859 | 0.701 | 10 | 7 | 1243 | regulation of RNA biosynthetic process |
| GO:0009725 | 0.859 | 0.511 | 2 | 1 | 251 | response to hormone stimulus |
| GO:0010033 | 0.861 | 0.616 | 5 | 3 | 616 | response to organic substance |
| GO:0035556 | 0.861 | 0.645 | 6 | 4 | 781 | intracellular signal transduction |
| GO:0007169 | 0.862 | 0.505 | 2 | 1 | 254 | transmembrane receptor protein tyrosine kinase signaling pathway |
| GO:0048592 | 0.863 | 0.502 | 2 | 1 | 255 | eye morphogenesis |
| GO:0033365 | 0.863 | 0.502 | 2 | 1 | 255 | protein localization to organelle |
| GO:0048513 | 0.864 | 0.706 | 11 | 8 | 1403 | organ development |
| GO:0006357 | 0.866 | 0.566 | 3 | 2 | 449 | regulation of transcription from RNA polymerase II promoter |
| GO:0051179 | 0.867 | 0.746 | 17 | 14 | 2275 | localization |
| GO:0009887 | 0.871 | 0.601 | 5 | 3 | 630 | organ morphogenesis |
| GO:0006807 | 0.872 | 0.765 | 25 | 21 | 3249 | nitrogen compound metabolic process |
| GO:0009966 | 0.872 | 0.630 | 6 | 4 | 797 | regulation of signal transduction |
| GO:0009967 | 0.873 | 0.485 | 2 | 1 | 264 | positive regulation of signal transduction |
| GO:0046907 | 0.873 | 0.598 | 5 | 3 | 633 | intracellular transport |
| GO:0046483 | 0.876 | 0.594 | 5 | 3 | 637 | heterocycle metabolic process |
| GO:0006351 | 0.876 | 0.692 | 11 | 8 | 1426 | transcription, DNA-dependent |
| GO:0000279 | 0.876 | 0.549 | 4 | 2 | 462 | M phase |
| GO:0032774 | 0.878 | 0.690 | 11 | 8 | 1430 | RNA biosynthetic process |
| GO:0009894 | 0.879 | 0.474 | 2 | 1 | 270 | regulation of catabolic process |
| GO:0051276 | 0.879 | 0.544 | 4 | 2 | 466 | chromosome organization |
| GO:0010628 | 0.879 | 0.544 | 4 | 2 | 466 | positive regulation of gene expression |
| GO:0032270 | 0.880 | 0.472 | 2 | 1 | 271 | positive regulation of cellular protein metabolic process |
| GO:0033043 | 0.885 | 0.461 | 2 | 1 | 277 | regulation of organelle organization |
| GO:0010557 | 0.887 | 0.532 | 4 | 2 | 476 | positive regulation of macromolecule biosynthetic process |
| GO:0031326 | 0.891 | 0.674 | 11 | 8 | 1457 | regulation of cellular biosynthetic process |
| GO:0048468 | 0.892 | 0.646 | 9 | 6 | 1151 | cell development |
| GO:0023056 | 0.892 | 0.448 | 2 | 1 | 285 | positive regulation of signaling |
| GO:0051247 | 0.893 | 0.446 | 2 | 1 | 286 | positive regulation of protein metabolic process |
| GO:0010647 | 0.893 | 0.446 | 2 | 1 | 286 | positive regulation of cell communication |
| GO:0009889 | 0.896 | 0.669 | 11 | 8 | 1467 | regulation of biosynthetic process |
| GO:0051336 | 0.901 | 0.432 | 2 | 1 | 295 | regulation of hydrolase activity |
| GO:0007067 | 0.901 | 0.430 | 2 | 1 | 296 | mitosis |
| GO:0044281 | 0.902 | 0.660 | 11 | 8 | 1482 | small molecule metabolic process |
| GO:0000280 | 0.904 | 0.426 | 2 | 1 | 299 | nuclear division |
| GO:0000087 | 0.907 | 0.420 | 2 | 1 | 303 | M phase of mitotic cell cycle |
| GO:0055085 | 0.907 | 0.419 | 2 | 1 | 304 | transmembrane transport |
| GO:0009719 | 0.909 | 0.416 | 2 | 1 | 306 | response to endogenous stimulus |
| GO:0070887 | 0.910 | 0.493 | 4 | 2 | 511 | cellular response to chemical stimulus |
| GO:0044255 | 0.911 | 0.491 | 4 | 2 | 513 | cellular lipid metabolic process |
| GO:0048285 | 0.913 | 0.409 | 2 | 1 | 311 | organelle fission |
| GO:0034641 | 0.913 | 0.719 | 25 | 20 | 3228 | cellular nitrogen compound metabolic process |
| GO:0048598 | 0.915 | 0.405 | 2 | 1 | 314 | embryonic morphogenesis |
| GO:0009058 | 0.916 | 0.706 | 22 | 17 | 2823 | biosynthetic process |
| GO:0051128 | 0.917 | 0.529 | 5 | 3 | 708 | regulation of cellular component organization |
| GO:0016070 | 0.918 | 0.669 | 15 | 11 | 1973 | RNA metabolic process |
| GO:0061061 | 0.918 | 0.398 | 2 | 1 | 319 | muscle structure development |
| GO:0006366 | 0.919 | 0.477 | 4 | 2 | 527 | transcription from RNA polymerase II promoter |
| GO:0090304 | 0.920 | 0.692 | 20 | 15 | 2556 | nucleic acid metabolic process |
| GO:0034613 | 0.922 | 0.471 | 4 | 2 | 533 | cellular protein localization |
| GO:2000112 | 0.922 | 0.618 | 11 | 7 | 1381 | regulation of cellular macromolecule biosynthetic process |
| GO:0002009 | 0.923 | 0.388 | 3 | 1 | 327 | morphogenesis of an epithelium |
| GO:0032501 | 0.926 | 0.703 | 25 | 20 | 3272 | multicellular organismal process |
| GO:0010556 | 0.926 | 0.612 | 11 | 7 | 1392 | regulation of macromolecule biosynthetic process |
| GO:0042221 | 0.927 | 0.595 | 9 | 6 | 1234 | response to chemical stimulus |
| GO:0031323 | 0.927 | 0.656 | 15 | 11 | 2002 | regulation of cellular metabolic process |
| GO:0023051 | 0.928 | 0.545 | 7 | 4 | 907 | regulation of signaling |
| GO:0030182 | 0.928 | 0.510 | 6 | 3 | 732 | neuron differentiation |
| GO:0046434 | 0.934 | 0.366 | 3 | 1 | 346 | organophosphate catabolic process |
| GO:0044249 | 0.936 | 0.674 | 21 | 16 | 2757 | cellular biosynthetic process |
| GO:0046903 | 0.937 | 0.358 | 3 | 1 | 353 | secretion |
| GO:0008610 | 0.938 | 0.356 | 3 | 1 | 355 | lipid biosynthetic process |
| GO:0048584 | 0.940 | 0.352 | 3 | 1 | 359 | positive regulation of response to stimulus |
| GO:0046700 | 0.941 | 0.351 | 3 | 1 | 360 | heterocycle catabolic process |
| GO:0044270 | 0.943 | 0.346 | 3 | 1 | 365 | cellular nitrogen compound catabolic process |
| GO:0048699 | 0.945 | 0.477 | 6 | 3 | 778 | generation of neurons |
| GO:0006935 | 0.945 | 0.341 | 3 | 1 | 370 | chemotaxis |
| GO:0006996 | 0.953 | 0.566 | 11 | 7 | 1485 | organelle organization |
| GO:0060429 | 0.954 | 0.321 | 3 | 1 | 392 | epithelium development |
| GO:0051049 | 0.955 | 0.318 | 3 | 1 | 395 | regulation of transport |
| GO:0006461 | 0.957 | 0.314 | 3 | 1 | 400 | protein complex assembly |
| GO:0016043 | 0.958 | 0.626 | 20 | 14 | 2573 | cellular component organization |
| GO:0070271 | 0.958 | 0.312 | 3 | 1 | 403 | protein complex biogenesis |
| GO:0048666 | 0.960 | 0.392 | 5 | 2 | 632 | neuron development |
| GO:0007275 | 0.960 | 0.622 | 20 | 14 | 2584 | multicellular organismal development |
| GO:0050794 | 0.964 | 0.644 | 27 | 21 | 3590 | regulation of cellular process |
| GO:0007010 | 0.965 | 0.380 | 5 | 2 | 651 | cytoskeleton organization |
| GO:0048856 | 0.965 | 0.605 | 19 | 13 | 2468 | anatomical structure development |
| GO:0071822 | 0.966 | 0.292 | 3 | 1 | 429 | protein complex subunit organization |
| GO:0007399 | 0.967 | 0.493 | 9 | 5 | 1220 | nervous system development |
| GO:0030154 | 0.967 | 0.549 | 13 | 8 | 1719 | cell differentiation |
| GO:0019637 | 0.970 | 0.366 | 5 | 2 | 674 | organophosphate metabolic process |
| GO:1901135 | 0.973 | 0.358 | 5 | 2 | 687 | carbohydrate derivative metabolic process |
| GO:0015031 | 0.973 | 0.356 | 5 | 2 | 691 | protein transport |
| GO:0071842 | 0.975 | 0.565 | 17 | 11 | 2236 | cellular component organization at cellular level |
| GO:0045184 | 0.977 | 0.345 | 5 | 2 | 710 | establishment of protein localization |
| GO:0048667 | 0.977 | 0.261 | 4 | 1 | 476 | cell morphogenesis involved in neuron differentiation |
| GO:0006629 | 0.977 | 0.344 | 5 | 2 | 712 | lipid metabolic process |
| GO:0071840 | 0.978 | 0.579 | 21 | 14 | 2711 | cellular component organization or biogenesis |
| GO:0048812 | 0.978 | 0.257 | 4 | 1 | 483 | neuron projection morphogenesis |
| GO:0051649 | 0.978 | 0.393 | 7 | 3 | 925 | establishment of localization in cell |
| GO:0022008 | 0.981 | 0.384 | 7 | 3 | 943 | neurogenesis |
| GO:0048869 | 0.982 | 0.506 | 14 | 8 | 1831 | cellular developmental process |
| GO:0033036 | 0.983 | 0.413 | 9 | 4 | 1155 | macromolecule localization |
| GO:0048731 | 0.984 | 0.513 | 15 | 9 | 2013 | system development |
| GO:0000904 | 0.984 | 0.237 | 4 | 1 | 522 | cell morphogenesis involved in differentiation |
| GO:0031175 | 0.985 | 0.233 | 4 | 1 | 530 | neuron projection development |
| GO:0044237 | 0.986 | 0.592 | 39 | 31 | 5100 | cellular metabolic process |
| GO:0009888 | 0.986 | 0.311 | 6 | 2 | 782 | tissue development |
| GO:0048858 | 0.987 | 0.225 | 4 | 1 | 547 | cell projection morphogenesis |
| GO:0071841 | 0.987 | 0.520 | 18 | 11 | 2374 | cellular component organization or biogenesis at cellular level |
| GO:0032990 | 0.988 | 0.222 | 4 | 1 | 555 | cell part morphogenesis |
| GO:0032502 | 0.990 | 0.530 | 22 | 14 | 2873 | developmental process |
| GO:0044260 | 0.991 | 0.548 | 28 | 19 | 3617 | cellular macromolecule metabolic process |
| GO:0034645 | 0.992 | 0.473 | 16 | 9 | 2137 | cellular macromolecule biosynthetic process |
| GO:0051641 | 0.992 | 0.332 | 8 | 3 | 1073 | cellular localization |
| GO:0006139 | 0.992 | 0.523 | 24 | 15 | 3070 | nucleobase-containing compound metabolic process |
| GO:0009059 | 0.993 | 0.467 | 17 | 9 | 2156 | macromolecule biosynthetic process |
| GO:0008104 | 0.996 | 0.249 | 7 | 2 | 953 | protein localization |
| GO:0000902 | 0.996 | 0.173 | 5 | 1 | 697 | cell morphogenesis |
| GO:0030030 | 0.996 | 0.173 | 5 | 1 | 698 | cell projection organization |
| GO:0009653 | 0.998 | 0.306 | 11 | 4 | 1484 | anatomical structure morphogenesis |
| GO:0032989 | 0.998 | 0.150 | 6 | 1 | 792 | cellular component morphogenesis |
| GO:0009987 | 0.999 | 0.394 | 55 | 46 | 7174 | cellular process |
